# Identification of Blood Circular RNAs as Potential Biomarkers for Acute Ischemic Stroke

**DOI:** 10.3389/fnins.2020.00081

**Published:** 2020-02-06

**Authors:** Dan Lu, Eric S. Ho, Hongcheng Mai, Jiankun Zang, Yanfang Liu, Yufeng Li, Bing Yang, Yan Ding, Chi Kwan Tsang, Anding Xu

**Affiliations:** ^1^Department of Neurology and Stroke Center, The First Affiliated Hospital of Jinan University, Guangzhou, China; ^2^Clinical Neuroscience Institute, The First Affiliated Hospital of Jinan University, Guangzhou, China; ^3^Department of Biology, Lafayette College, Easton, PA, United States; ^4^Department of Computer Science, Lafayette College, Easton, PA, United States

**Keywords:** circular RNA, acute ischemic stroke, biomarkers, cerebral ischemia, blood, bioinformatics analysis

## Abstract

Many hospitals lack facilities for accurate diagnosis of acute ischemic stroke (AIS). Circular RNA (circRNA) is highly expressed in the brain and is closely associated with stroke. In this study, we examined whether the blood-borne circRNAs could be promising candidates as adjunctive diagnostic biomarkers and their pathophysiological roles after stroke. We profiled the blood circRNA expression in mice subjected to experimental focal cerebral ischemia and validated the selected circRNAs in AIS patients. We demonstrated that 128, 198, and 789 circRNAs were significantly altered at 5 min, 3 h, and 24 h after ischemic stroke, respectively. Our bioinformatics analysis revealed that the circRNA-targeted genes were associated with the Hippo signaling pathway, extracellular matrix-receptor interaction, and fatty acid metabolism at 5 min, 3 h and 24 h after ischemic stroke, respectively. We verified that many of these circRNAs existed in the mouse brain. Furthermore, we found that most of the predicted circRNA-miRNA interactions apparently exhibited functional roles in terms of regulation of their target gene expression in the brain. We also verified that many of these mouse circRNAs were conserved in human. Finally, we found that circBBS2 and circPHKA2 were differentially expressed in the blood of AIS patients. These results demonstrate that blood circRNAs may serve as potential biomarkers for AIS diagnosis and reveal the pathophysiological responses in the brain after ischemic stroke.

## Introduction

Stroke is the second most common cause of death worldwide, and many stroke survivors suffer from long-term disability, severely affecting the well-being of the patients and family members (GBD 2015 Mortality and Causes of Death Collaborators, 2016; Bazan et al., 2017). The two major types of stroke are acute ischemic stroke (AIS), which occurs when blood flow through the artery that brings oxygen-rich blood to the brain is obstructed (usually due to a blood clot), and hemorrhagic stroke, which occurs when an artery in the brain ruptures (CDC, 2014). Because ischemic stroke is more common than hemorrhagic stroke (87% of cases), most stroke prevention investigations are focused on AIS (Meschia et al., 2014).

The current standard treatment for stroke involves the administration of tissue plasminogen activator (t-PA) (Collen and Lijnen, 2009). t-PA activates the proteolytic enzyme plasmin, which subsequently breaks down stroke-causing blood clots. Although the American Heart Association 2018 Guideline for Management of Acute Ischemic Stroke strongly recommends the use of t-PA for patients who meet certain criteria (Meschia et al., 2014), only about 2–5% of patients with AIS in the United States received t-PA (Miller et al., 2011). Effective treatment requires administration within four and a half hours after stroke onset – a timeframe that is often too narrow for patients to be diagnosed and/or properly screened for treatment eligibility. Complications with t-PA administration include risk of major intracranial hemorrhage and angioedema. A recent clinical trial showed that t-PA administration only restored blood flow in 37% of patients (Campbell et al., 2015). Thus, there is a need for the development of safer and more universally applicable diagnostic tools and therapeutics to improve the quality and safety of available stroke treatments. As speedy diagnosis is critical in preventing permanent damage to brain tissues, however, numerous hospitals, especially those in rural areas, lack the required imaging facilities for diagnosis of AIS. Therefore, a more accessible, non-invasive diagnostic method, such as a blood-based biomarker, would be of great value for the diagnosis of AIS, swiftly assisting clinicians in choosing the most effective treatment strategy for stroke patients and hence leading to better outcomes.

Circular RNAs (circRNAs) are a class of RNA molecules that may hold the keys to understanding and properly manipulating the tightly regulated gene expression patterns, as they are more highly expressed in brain tissues than in other tissues (Rybak-Wolf et al., 2015; Zhang et al., 2016). CircRNAs are produced by back-splicing of mRNAs or lncRNAs during post-transcriptional modification. Because of their closed covalent structure and resistance to exonuclease, circRNAs are much more stable than their linear RNA counterparts (Lu and Xu, 2016). Although the functions of circRNAs remain largely unknown, some circRNAs can upregulate their parental genes in different cell types (Luo et al., 2017; Pamudurti et al., 2017). Moreover, emerging evidence has shown that some of them, termed microRNA “sponges,” can sequester miRNAs for the regulation of their downstream target genes (Hansen et al., 2013). This proposed functionality is especially relevant for this present study, because numerous miRNAs that play roles in the post-stroke brain have been identified (Li et al., 2018). miRNAs play roles in a range of neurological responses that are triggered in the post-stroke brain, including excitotoxicity, oxidative stress, apoptosis, blood-brain barrier damage, edema, and inflammation. For example, miR-223 and miR-125b both affect levels of specific NMDA receptor subunits (NR2B and NR2A, respectively), which subsequently impact the functionality of glutamate receptors and affect the chances of excitotoxic neuronal death (Li et al., 2010).

Accumulating evidence has shown that the brain-specific circRNAs are associated with their pathophysiological functions. For instance, animal studies have revealed that circRNA expression in brain tissues is closely associated with ischemic stroke (Lin et al., 2016; Liu et al., 2017; Mehta et al., 2017). Notably, circRNAs expressed in brain tissues have been reproducibly detected in human peripheral blood samples (Memczak et al., 2015). Moreover, expressions of circRNAs are much higher than their corresponding linear mRNA counterparts (Memczak et al., 2015). These findings suggest that the blood-borne circRNAs may be potential diagnostic biomarkers and reveal the pathophysiology for ischemic stroke. The goal of this study was to determine the potential of circRNAs in the blood as diagnostic biomarkers for acute ischemic stroke. We initially profiled the blood circRNA expression in mice subjected to experimental focal cerebral stroke. We then used bioinformatics analysis to determine whether the blood circRNAs were associated with cerebral ischemic stroke and to determine the potentially novel responses after stroke. Finally, we validated the circRNA candidates in the blood of acute ischemic stroke patients.

## Materials and Methods

### Animal Experiments

Adult male Balb/c mice (10–12 weeks old) were purchased from the Institute of Laboratory Animal Science of the Chinese Academy of Medical Sciences (Guangzhou, China). Food and water were freely available throughout the experiments. All experiments were carried out in accordance with the guidelines for Animal Experimentation of Jinan University and were approved by the Committee for Animal Experimentation. The mice used for experiments had similar body weight (22∼25 g) and normal physiological index values. All mice were assigned with a computer-generated random number and were allocated to experimental groups randomly.

### Focal Ischemia

Focal ischemia was induced by intraluminal middle cerebral artery occlusion (MCAO) for 5 min, 3 h and 24 h using a monofilament nylon string coated with silicon (MSMC23B104PK100, RWD Life Science, Shenzhan, China) in male Balb/c mice under isoflurane anesthesia as described earlier (Wang et al., 2004; Krawiec et al., 2009). Physiological parameters (PaO_2_, PaCO_2_, blood pressure) were monitored, and rectal temperature was controlled at 37.0 ± 0.5°C. Cortical cerebral blood flow was monitored using a blood perfusion imager (PeriCam PSI System, Perimed AB, Stockholm, Sweden). None of the animals showed any adverse effects before euthanasia. Animal blood was extracted after different time points of surgery. Sham-operated mice underwent the same surgical procedure except that MCAO was used for the 0 min time point.

### Blood RNA Extraction

Prior to blood collection, the PAXgene RNA stabilizer solution was removed from the BD Vacutainer-style PAXgene blood RNA tubes. Whole blood obtained from the cardiac puncture of mice under anesthesia was collected into heparinized tubes (Wang et al., 2004; Krawiec et al., 2009). All tubes were gently inverted for 5 min directly after collection and incubated for 2 h at room temperature and then stored at −80°C until processing for RNA extraction. Total RNA was extracted from blood samples using the PAXgene Blood RNA kit (Qiagen) according to the manufacturer’s protocol.

### Brain RNA Extraction

After being deeply anesthetized and saline perfused, the animals were dissected and brain tissue from the infarct, peri-infarct (penumbra), contralateral ischemic hemisphere (con), and SHAM (the zones corresponding to the infarct and peri-infarct in sham-operation mice) zones were collected. Total RNA was isolated using Trizol reagent (Invitrogen) according to the manufacturer’s protocol. The RNA sample concentrations were determined by OD260/280 using a Nanodrop ND-1000 spectrophotometer (Thermo Fisher Scientific) instrument.

### CircRNA Microarray

Circular RNAs were characterized using circRNA microarray analyses of ribosomal RNA-depleted total RNA from blood samples. Triplicate samples were used for each time point. Total RNA from each sample was quantified using the NanoDrop 2000. The sample labeling, preparation and microarray hybridization were performed based on the manufacturer’s standard protocols (Arraystar Inc.) Briefly, total RNAs were digested with RNase R (Epicentre, Inc.) to remove linear RNAs and enrich circular RNAs. Then, the enriched circular RNAs were amplified and transcribed to fluorescent cRNA utilizing a random priming method (Arraystar Super RNA Labeling Kit; Arraystar). The labeled cRNAs were purified by RNeasy Mini Kit (Qiagen). The concentration and specific activity of the labeled cRNAs (pmol Cy3/μg cRNA) were measured by NanoDrop ND-1000. One μg of each labeled cRNA was fragmented by adding 5 μl of 10 × blocking agent and 1 μl of 25 × fragmentation buffer. The mixture was then heated at 60°C for 30 min. Twenty-five microliters of 2 × hybridization buffer was added to dilute the labeled cRNA. Fifty microliters of hybridization solution was dispensed into the gasket slide and assembled on the circRNA expression microarray slide [Arraystar Mouse circRNA Array v2 (8 × 15K, Arraystar)]. The slides were incubated for 17 h at 65°C in an Agilent Hybridization Oven. The hybridized arrays were washed, fixed, and scanned using the Agilent G2505C Scanner.

### Identification of Differentially Expressed circRNAs

The Arraystar Mouse Microarray was used in this study. The probe definitions, however, were not based on the commonly used NCBI RefSeq database. To facilitate downstream bioinformatics analysis, we developed an internal pipeline to map proprietary probe IDs to RefSeq accession numbers. First, several proprietary probe IDs were used as search terms to search for array definitions in the NCBI GEO database. circRNA chips GPL21826 and GPL7173 showed the highest identity to our data. The corresponding definition files (GPL21826_family.soft and GPL7173_family.soft) were downloaded. Next, the probe sequences provided by the definition files were used to uncover the RefSeq accession numbers associated with the probes by using our locally installed NCBI BLASTN for the mouse (mm10) genome.

Regular probes that were not at least 10% brighter than the control probes in all twelve arrays were removed, resulting in 10324 high-quality probes out of 15712 (66%). Bioconductor’s limma package (Ritchie et al., 2015) was used to identify differently expressed circRNAs at four different time points, i.e., time 0, 5 min, 3 h, and 24 h, each with three replicates, resulting in twelve samples in total. Time 0 is the control group (SHAM), resulting in three comparisons: 5 min (MCAO 5 m) versus 0 h (SHAM), 3 h (MCAO 3 h) versus 0 h (SHAM), and 24 h (MCAO 24 h) versus 0 h (SHAM). Agilent Feature Extraction software (version 11.0.1.1) was used to analyze acquired array images. Quantile normalization and subsequent data processing were performed using the limma package in R software. Briefly, the single-channel Agilent data collection model was used where only the green signal was considered. Intra-array variation was normalized by the adaptive background correction recommended by limma. Inter-array background signals were further corrected by the quantile method. Differentially expressed circRNAs with statistical significance between two groups were identified through Volcano Plot filtering (absolute log fold-change ≥2, adjusted *P* value ≤0.05). Differentially expressed circRNAs between two samples were identified through fold-change filtering. Hierarchical clustering was performed to show the distinguishable circRNA expression pattern among samples. Differentially expressed circRNAs were ranked by log fold-change and were considered significant if the fold-change was ≥2.

### Verification of circRNAs by RT-qPCR

Ischemia-induced differentially expressed circRNAs were verified by RT-qPCR. Briefly, total RNA was extracted using an RNeasy Mini Kit (Qiagen), and 1 μg of RNA was reverse-transcribed into cDNA with SuperScript III First-Strand Synthesis System (Thermo Fisher Scientific) after being digested with RNase R. The circRNAs were amplified with circRNA specific outward-facing divergent primers (Supplementary Table S1). The amplification reaction and detection were conducted in a QuantStudio 3 real-time PCR machine (Bio-Rad, CA, United States) using a miScript SYBR^®^ Green PCR Kit (Roche, United States) as described earlier 7,18. GAPDH was used as internal control.

### Validation of Patient Blood circRNAs

Venous blood (2.5 ml) was withdrawn from each participant into a PAXgene Blood RNA tube. Total RNA was extracted from samples using the RNAiso Blood (TAKARA), followed by RNase R digestion. Reverse transcription was performed by using the PrimeScriptTMRT reagent Kit (TAKARA) according to the manufacturer’s instructions with minor modification. Briefly, the reaction mixture, with gDNA eraser added, was incubated for 2 min at 42°C, followed by incubation with reverse transcriptase for 15 min at 37°C. The reaction mixtures were then incubated for 5 s at 85°C to inactivate miScript Reverse Transcriptase. Quantitative PCR was carried out using the miScript SYBR^®^ Green PCR Kit (Roche, United States). The primer sequences used for the detection of human circRNAs were designed based on circRNA conservation analysis using the Primer Assay Program (Biosen, China). GAPDH was used as a control. Primer sequences for human circRNAs are shown as Supplementary Table S1.

### CircRNA Conservation Analysis

Conservation of mouse circRNAs was measured by aligning sequences of their human homologies through BLASTN. Human (hg19) circRNA sequences were downloaded from circBase (Glazar et al., 2014). After filtering emptied sequences, 140732 out of 140790 human circRNAs were indexed in a BLASTN database. We queried mouse circRNA sequences against the human circRNA database by BLASTN using default parameter values except that the maximum *E*-value was reduced to 1e-10.

### Infarct Size Measurement

The infarct size was evaluated after different time points of MCAO. Mice were anesthetized with 5% isoflurane and perfused transcardially with cold saline. The brains were quickly removed and sectioned coronally into 1-mm slices using stainless steel brain matrices (RWD Life Science, Shenzhan, China). The brain slices were stained with 2% 2,3,5-triphenyltetrazolium chloride (TTC) in a dark chamber at 37°C for 20 min and then fixed with 4% paraformaldehyde. Slice images were captured using a digital camera. Measurements of the hemispheric volumes were performed using the Image-Pro^®^ Plus (Version 6.0, NIH). The infarct volumes were calculated according to the following formula: Infarct volume (%) = (Contralateral hemispheric volume - Ipsilateral hemispheric volume + Infarct volume) × 100%/contralateral hemisphere volume (Lu et al., 2018; Zhang et al., 2018).

### Pathway Analysis of circRNA Parental Genes

The functional classification and enrichment analysis of parental genes originating from the ischemia-responsive circRNAs were conducted using the ontology of the terms generated with gene ontology curation through the publicly available GeneCoDIS3^1^ (Tabas-Madrid et al., 2012). GeneCoDIS3 uses a hypergeometric model to identify pathways with an over-represented presence of queried genes to provide a Modular Enrichment Analysis (MEA) in which enrichment analysis can be done by sourcing annotations from PANTHER and KEGG sources. A key advantage of GeneCodis3 over other tools, such as DAVID (Huang da et al., 2009), is that GeneCodis3 sources pathway information from multiple databases. In this study, we used pathway databases PANTHER (Mi et al., 2016) and KEGG (Kanehisa et al., 2012). The 22,601 genes probed by the microarray used in this study were used as the reference genes required by GeneCoDIS3.

### Prediction of circRNA-Interacting miRNAs and Gene Ontology Annotations of miRNA-Target Genes

CircRNA-miRNA interactions were predicted by a two-step analysis. The first analysis was performed by proprietary miRNA target prediction software developed based on RegRNA 2.0 (Huang et al., 2006)^2^, in which sequence matching is one of the major conditions used by the method. The second analysis was performed by functional RNA motifs with default parameters (Chang et al., 2013). The PANTHER Classification System was used to classify the comprehensive functions of proteins and their genes. MiRNA-targeted genes were predicted by the Gene Ontology Phylogenetic Annotation.

### Pathway Analysis of the circRNA-miRNA Targeted Genes

Shortlisted circRNA-interacting miRNAs were further analyzed by mirPath v.3 (Vlachos et al., 2015) and TargetScan (Shi et al., 2017), leading to the discovery of pathways that may be mediated by circRNA-miRNA interactions. Pathway/category union was predicted by mirPath v.3 (Vlachos et al., 2015) and TargetScan (Shi et al., 2017), which identified all the pathways significantly targeted by the selected microRNAs. Fisher’s meta-analysis method was used to calculate the significance levels and extract the merged *p*-values for each pathway. The resulting *p*-values depicted the probability that the examined pathway was significantly enriched with gene targets of at least one selected microRNA. The merged *p*-values were determined by *a posteriori* Analysis Method (Vlachos et al., 2015).

### Characterization of Patient Population

All subjects were recruited from the Department of Neurology and Stroke Center of The First Affiliated Hospital of Jinan University from November 2017 to January 2018. The clinical data were collected from case-controlled studies. Acute ischemic stroke patients and control subjects included in the validation samples were matched with respect to all variables, including demographic, vascular risk factors, and previous use of medication. The average time from symptom onset to blood sampling was 179 min. Stroke etiologies in the IS patients included large-artery atherosclerosis (three patients), cardioembolism (two patients), and small-vessel occlusion (three patients). Patients who showed acute major artery occlusion within 24 h from acute ischemic stroke onset were examined with MRI, DWI, and MRA immediately after hospital arrival. MRI examinations were performed using a 3.0-T unit (Achieva 3T, Philips Medical Systems, Best, Netherlands) with an eight-channel phased-array head coil. DWI was performed by two-dimensional single-shot, spin-echo, echo-planar imaging DWI of the entire brain with the following parameters: DWI: TR = 4,300∼5,000 millisecond (msec), TE = 81.7∼90.3 msec, vision FOV: 24 × 24 cm, NEX was 2, flip angle, 90o, imaging matrix, 128 × 128, thickness of layer, 6.0 mm, spacing between layers, 1.0 mm. MRA adopted the three-dimensional time-of-flight method and TR = 22∼25 msec, TE = 2.6∼3.6 msec, a layer thickness of 6.0 mm, spacing between layers of 1.0 mm, vision FOV of 24 × 24 cm, and NEX of 2. The study was conducted in accordance with the guidelines of the First Affiliated Hospital of Jinan University. Written and informed consent was obtained from all subjects.

### Statistical Analysis

All statistical analyses were performed by the Statistical Product and Service Solutions (SPSS) 22.0 software package (IBM Corporation, Armonk, NY, United States) and Graph-Pad Prism 6.0 (GraphPad Software, Inc., La Jolla, CA, United States). Values are expressed as mean ± SEM. The qPCR verified differences in the levels of circRNAs between the sham group and MCAO groups or control subject group and paired AIS patient group were assessed using the Student’s *t*-test (if homogeneity of variance was determined) or Tamhane’s T2 *post hoc* test (if homogeneity of variance was not determined) for paired data. Two-sided *p*-values < 0.05 were considered statistically significant. Statistical differences among more than two groups were assessed using one-way ANOVA with the least significant difference test and determination of homogeneity of variance.

## Results

### CircRNA Expression Profiles in the Blood of Mice Subjected to MCAO

**FIGURE 1:**
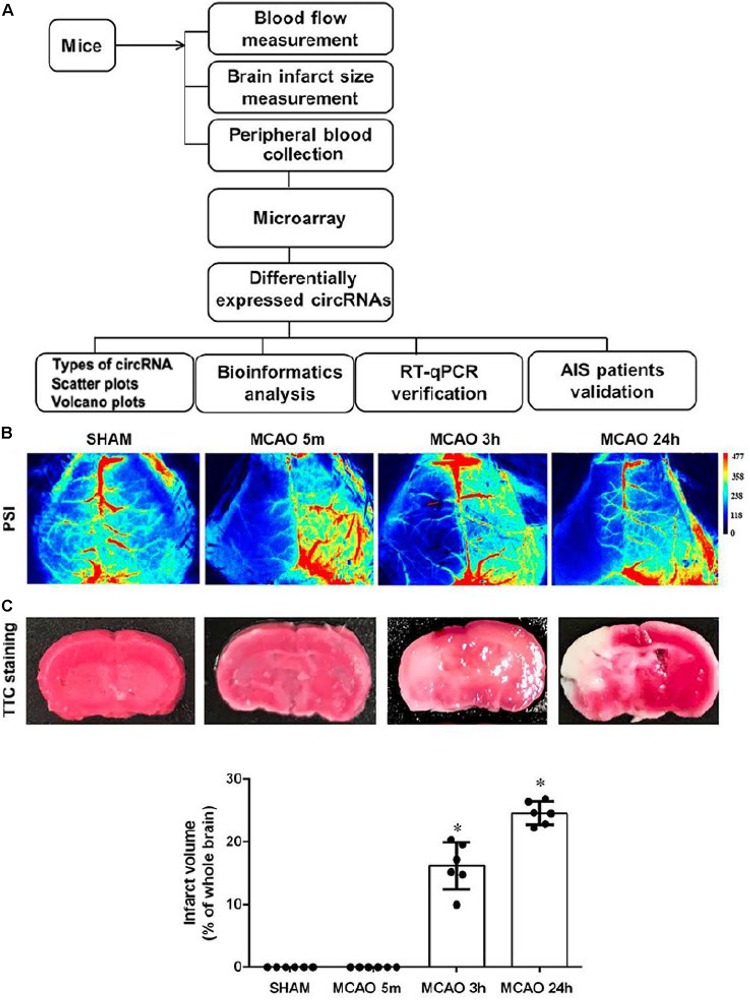
Experimental procedure of this study. **(A)** Schematic diagram of the experimental procedure. **(B)** Representative images of cerebral blood flow in SHAM and 5-min, 3-h, and 24-h MCAO-treated mice captured by a blood perfusion imager. PSI, Perfusion speckle imaging. **(C)** Representative images of TTC-stained histology of brain sections at different time points of MCAO. The survival and infarct tissues are stained red and white, respectively. Lower panel shows the quantification of infarct volume. Error bars represent mean ± SEM; *n* = 6; ^∗^*p* < 0.05 compared with sham, Student’s *t*-test.

We used the experimental procedure described in Figure 1A to profile the blood circRNA expression in mice at different time points of focal cerebral ischemic stroke by middle cerebral artery occlusion (MCAO) (Figure 1B).

**FIGURE 2:**
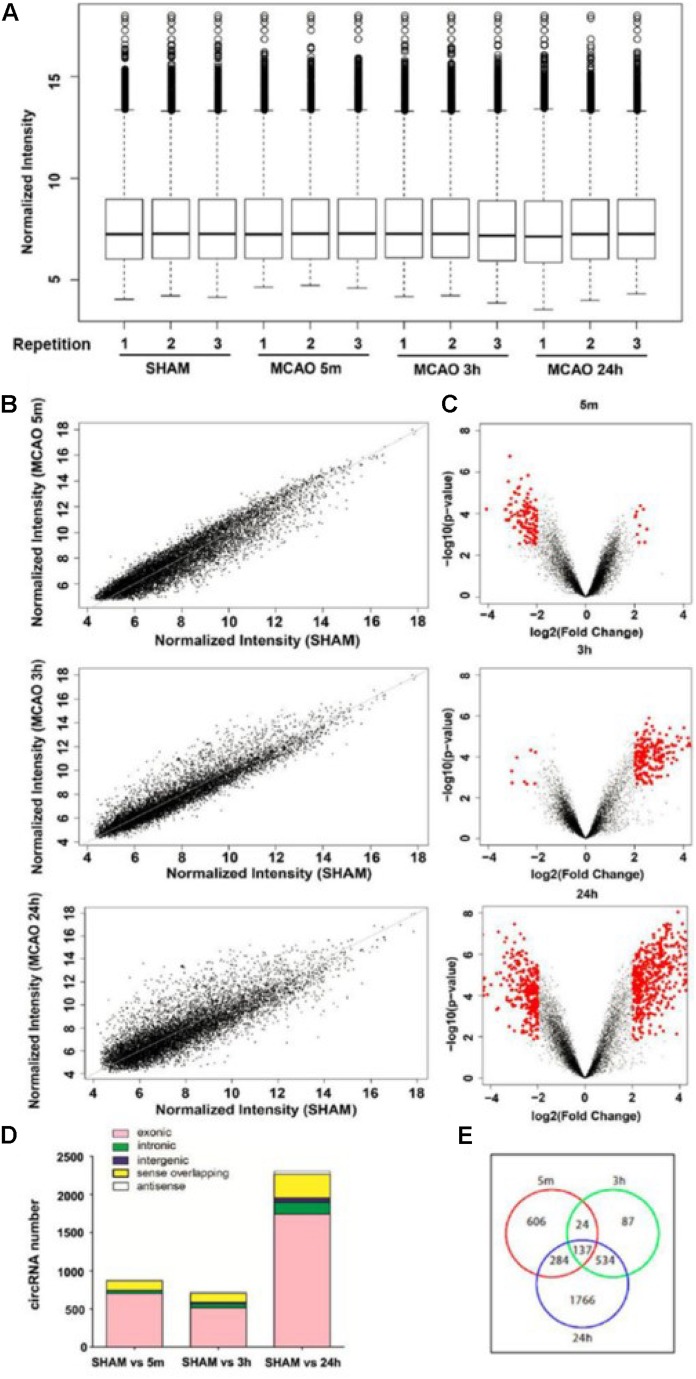
Characterization of the expression profile of circular RNAs in blood samples of MCAO-treated mice. **(A)** Normalized intensities of all circular RNAs expressed in the blood in sham and 5 min, 3-h, and 24-h MCAO-treated mice; *n* = 3 per group. **(B)** The scatter plots show the differentially expressed circRNAs in the 5-min, 3-h, and 24-h MCAO groups compared with sham. circRNAs in the scatter plot above and below the diagonal line indicate upregulation and downregulation, respectively. **(C)** Volcano plots show circRNA expression profiles in the 5-min, 3-h, and 24-h MCAO groups compared with sham control. Red dots represent differentially expressed circRNAs (*p* < 0.05 and fold-change ≥ 2.0). **(D)** Distribution of different types of differentially expressed circRNAs, including those consisting of exon, intron, intergenic region, sense, and antisense sequences. **(E)** Venn diagram shows the overlapping differentially expressed circRNA probes among the three groups compared with sham control. The total numbers of probes exhibiting differential expression in 5 min, 3 h, and 24 h are 1051, 782, and 2721, respectively.

Brain ischemia for 5 min was used as the time for inducing brain adaptive responses against ischemia before permanent brain damage. Three hours was used as the time for the presence of salvageable ischemic penumbra, whereas, at 24 h, the infarct size was maximized (Figure 1C; Lo, 2008). Occlusion of blood flow of all animals was confirmed by blood perfusion imaging (Figure 1B). 2,3,5-triphenyl-tetrazolim chloride (TTC) staining was used to verify that MCAO for 5 min had not caused detectable brain damage whereas, at 3 h, MCAO had already initiated signs of brain infarction, as shown by the pale pink staining. At 24 h of MCAO, significant enlargement of brain damage (white region) was detected (Figure 1C). Blood samples were then subjected to Arraystar Mouse circRNA Array v2 analysis. Our results showed that 10,739 circRNA probes were of high quality and were used in the microarray analysis (Supplementary Figure S1A). Among them, 10,324 (96%) probes were used for profiling the differential expression of blood circRNA (Supplementary Figure S1B). The distribution of circRNA expression patterns in these three groups had no significant difference (Figure 2A), demonstrating that the circRNA alterations were not random.

Scatter and volcano plots between the sham and different MCAO time points show the significant variation and the fold change of circRNA expression, respectively (Figures 2B,C). The UCSC mm10 database was then used to annotate these circRNAs based on their genomic locations. As shown in Figure 2D, most of the circRNAs consisted of protein-coding exons, whereas a smaller fraction aligned with intronic, intergenic, sense-overlapping, and antisense regions to known transcripts. In the blood samples, 1051, 782, and 2721 circRNA probes exhibited differential expression at 5 min, 3 h, and 24 h of MCAO respectively, as shown in the Venn diagram (Figure 2E).

**FIGURE 3:**
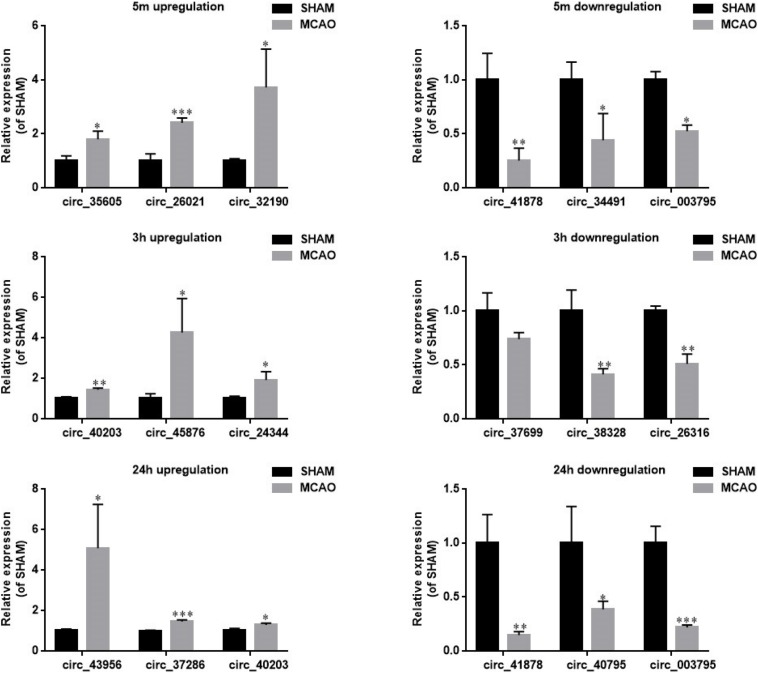
RT-qPCR verification of the microarray data from mouse blood. Representative circRNAs with significant differential expression at 5 min (upper panel), 3 h (middle panel), and 24 h of MCAO (lower panel) were verified by RT-qPCR. Left and right panels show the circRNAs with upregulation and downregulation, respectively. Values are mean ± SEM (*n* = 3 per group). ^∗^*p* < 0.05, ^∗∗^*p* < 0.05, and ^∗∗∗^*p* < 0.001 compared with sham (independent samples *t*-test, single-tailed).

Next, we defined those circRNAs with more than two-fold changes of expression as significant. The numbers of these differentially expressed circRNAs (≥2.0-fold, *p* < 0.05) at each time point are summarized in the Venn diagram (Supplementary Figure S2). We identified 128, 198, and 789 circRNAs with significant differential expression at 5 min, 3 h and 24 h, respectively. The top 10 most altered circRNAs are listed in Supplementary Table S2. The full list at each time point is shown in Supplementary Table S3. The MCAO-induced alteration of the representative circRNA was then verified by RT-qPCR (Figure 3). We showed that the changes in circRNAs were mostly independent of their corresponding mRNA expression (Supplementary Figure S3). These results demonstrate that the circRNAs may play specific functional roles after different durations of MCAO.

To further verify whether the source of blood circRNAs was the brain, we collected ischemic brain tissue at the corresponding time points after MCAO and verified the representative circRNAs by RT-qPCR. As shown in Supplementary Figure S5, most of the circRNAs could be detected in the brain tissues. Therefore, these results suggest that the ischemic brain is apparently the source of circRNAs detected in the blood.

### Pathway Analysis of the Parental Genes of circRNAs

CircRNAs are known to be capable of regulating their parental genes (Li et al., 2015). We used GeneCoDIS3 to perform a pathway analysis of the parental genes of the differentially expressed circRNAs in an attempt to better understand their potential pathophysiological roles. GeneCoDIS3 provides a Modular Enrichment Analysis (MEA) in which enrichment analysis can be done by sourcing annotations from PANTHER and KEGG analysis (Tabas-Madrid et al., 2012). At 5 min of MCAO, the major pathways associated with the enriched parental genes of circRNAs include platelet-derived growth factor (PDGF), insulin, focal adhesion, and chemokine signaling pathways. At 3 h of MCAO, the major pathways include glutamatergic synapse, calcium signaling, and GnRH signaling. At 24 h of MCAO, the identified pathways include glutamatergic synapse, calcium signaling, and vascular smooth muscle contraction. The full list of annotations is summarized in Supplementary Table S4.

### CircRNA-miRNA-Target Gene Network, GO, and KEGG Pathway Analyses

**FIGURE 4:**
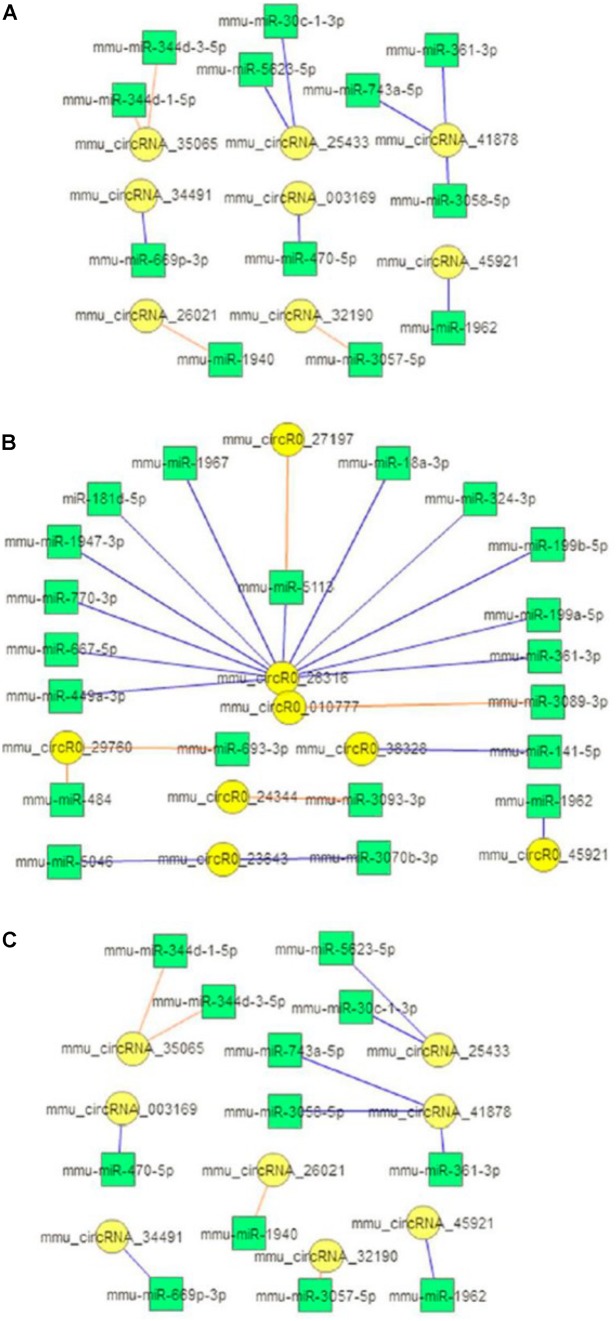
circRNA-miRNA interaction. Diagrams show the predicted miRNAs (square boxes) that bind to the verified differentially expressed circRNAs (round circles) at the 5-min **(A)**, 3-h **(B)**, and 24-h **(C)** time points of MCAO in mice. Blue lines represent upregulation; red lines represent downregulation.

A number of circRNAs can act as miRNA sponges for regulating gene expression through a circRNA-associated miRNA-regulated targeted gene network. To explore the possible pathophysiological involvement of brain ischemia-responsive circRNAs, we conducted miRNA sponge analysis using RegRNA 2.0 databases to predict the miRNA binding of the verified circRNAs in this study. RegRNA 2.0 is widely used to comprehensively predict interacting miRNAs based on the sequence of circRNAs and their internal structures together with the use of miRBase (Griffiths-Jones et al., 2008), which contains a central repository database for miRNA nomenclature, sequence data annotation, and target prediction (Huang et al., 2006; Chang et al., 2013). As shown in Figure 4, 24 out of 60 circRNAs highly altered after ischemia at the three time points showed miRNA binding sites. These circRNAs contained one to four miRNA binding sites, with the exception that mmu_circRNA_26316 contained 12 miRNA binding sites (Figure 4).

**FIGURE 5:**
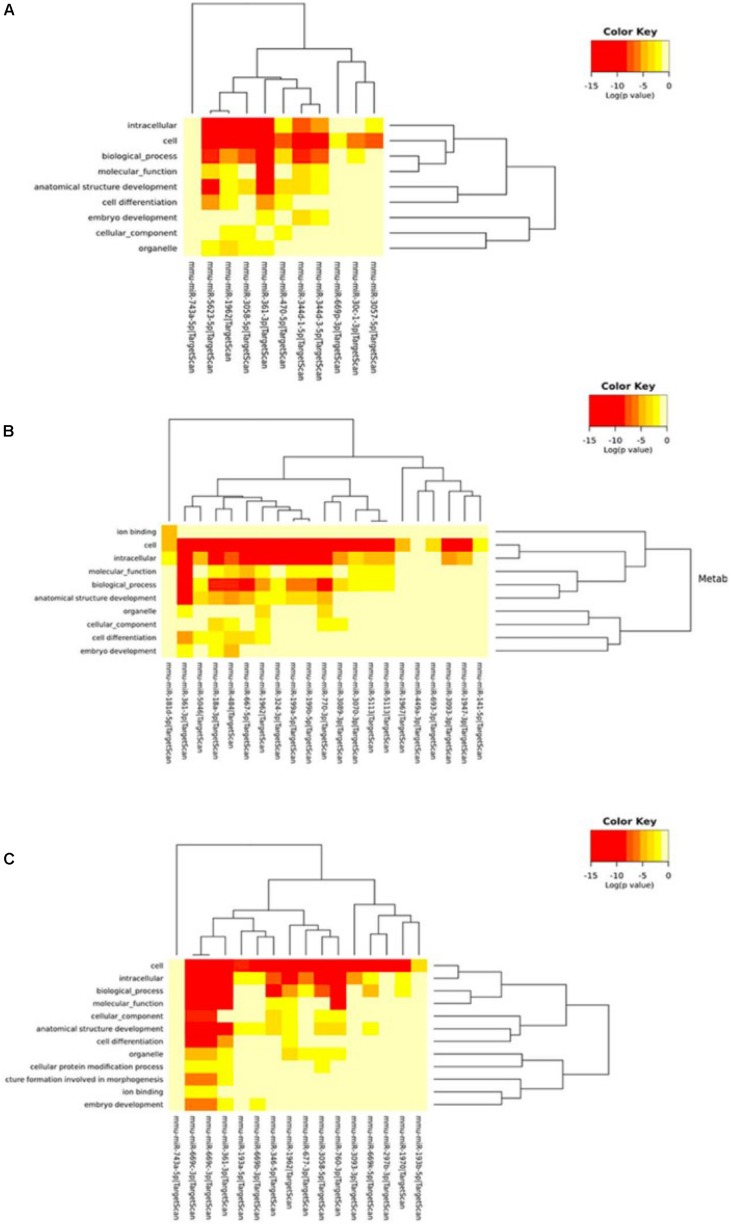
Gene ontology analysis. Gene ontology classifications of the circRNA-miRNA target genes at the **(A)** 5-min, **(B)** 3-h, and **(C)** 24-h time points of MCAO. Color key represents log(*p*-value).

Next, we performed Gene Ontology using the DIANA tool to analyze the functional enrichment of predicted circRNA-miRNA target genes (Figure 5 and Supplementary Tables S5, S6). The results of GO Panther analysis further indicated that at 5 min of MCAO, the major biological functions were antioxidant, catalytic, receptor, signal transducer, and transporter activities. At 3 h of MCAO, most gene functions were involved in the regulation of catalytic activity, while genes involved in antioxidant activity were prominently increased. At 24 h of MCAO, most gene functions were associated with binding and catalytic activity. Among them, the stroke-related biological processes included the immune process, metabolic process, and biological adhesion.

**FIGURE 6:**
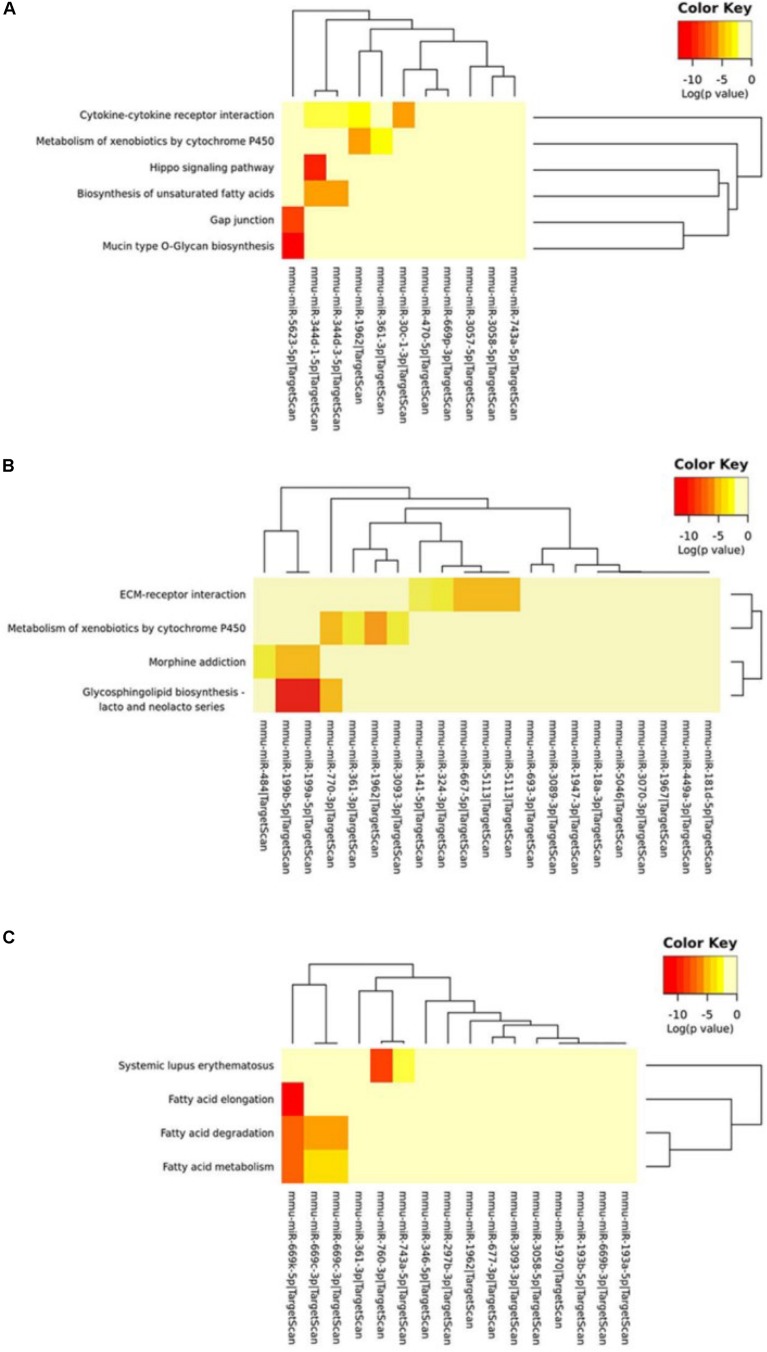
KEGG pathway analysis of circRNA-miRNA target genes. **(A)** KEGG pathway analysis of the circRNA-miRNA target genes at the **(A)** 5-min, **(B)** 3-h and **(C)** 24-h time points of MCAO. Color key represents log(*p* value).

We further used the MirPath and TargetScan databases to predict the pathways involved in circRNA-miRNA regulated genes (Supplementary Table S7). Based on the enrichment analysis and Fisher’s meta-analysis using the Posteriori Analysis Method, we found that the significant pathways after 5 min MCAO were associated with the Hippo signaling pathway, cytokine-cytokine receptor interaction, metabolism of xenobiotics by cytochrome P450, biosynthesis of unsaturated fatty acids, gap junction, and mucin type O-glycan biosynthesis (Figure 6A).

At 3 h, extracellular matrix (ECM)-receptor interaction, metabolism of xenobiotics by cytochrome P450, and glycosphingolipid biosynthesis of lacto- and neolacto-series were identified (Figure 6B). Interestingly, at 24 h, most of the identified significant pathways were associated with fatty acid metabolism (Figure 6C).

If circRNAs serve as miRNA sponges, circRNA-miRNA interactions should be verifiable by examining the expression of genes targeted by those miRNAs. Therefore, to verify the regulation of potential circRNA-miRNA target gene expression, we selected the representative target genes by KEGG analysis according to Figure 6 and Supplementary Table S7 and collected the ischemic brain tissues at different time points. We then verified their circRNA-miRNA target gene expression by RT-qPCR. As shown in Supplementary Figure S6, the expression of circRNA-miRNA target genes, at least in part, were differentially regulated, especially in the peri-infarct penumbra brain tissue (Supplementary Figure S6). These results suggest that the circRNAs could interact with their miRNAs and that the resultant circRNA-miRNA interactions may have a functional role in the regulation of their target gene expression.

### Identification of Differentially Expressed circRNAs in Stroke Patients

**FIGURE 7:**
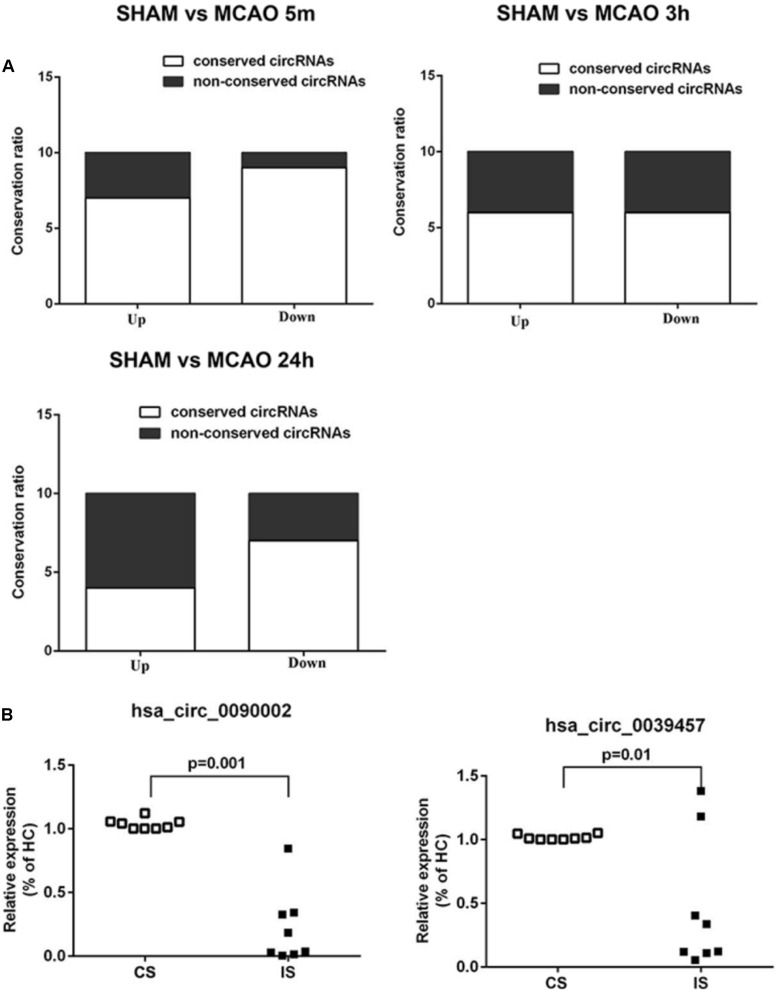
Conservation analysis and validation of the blood circRNAs in stroke patients. **(A)** Mouse-human conservation analysis of the verified blood circRNAs. Results show the percentage of conservation of the top 10 most differentially expressed circRNAs at different time points of MCAO (left, 5 min; middle, 3 h, right, 24 h). **(B)** Validation of circRNAs in acute ischemic stroke patients (IS) versus control subjects (CS) by RT-qPCR. hsa_circ_0090002 and hsa_circ_0039457 are circRNAs of PHKA2 and BBS2, respectively. Values are expressed as mean ± SEM, Student’s *t*-test value. *p* < 0.05 was considered statistically significant compared to sham group.

We first performed conservation analysis of the mouse ischemia-responsive blood circRNAs identified in our study with those in human. Our results showed that 65% of the highly differentially expressed circRNAs in all three time points were conserved in the corresponding human circRNAs (Figure 7A). This result is in line with the notion that circRNAs are highly evolutionarily conserved (Jeck et al., 2013), especially the exonic circRNAs (Guo et al., 2014).

To determine whether these conserved circRNAs were present in acute ischemic stroke patients, we collected blood samples from patients and the matched control subjects (Supplementary Table S8). Samples were matched for demographic and vascular risk factors as well as history of previous use of anti-platelet medications. The time from symptom onset to blood sample collection ranged from 60 to 280 min, with a mean time of 179 min. Stroke etiologies in the patients included large-artery atherosclerosis (44.4%), cardioembolism (22.2%), and small-vessel occlusion (33.3%). We performed diffusion-weighted imaging (DWI) and magnetic resonance angiography (MRA) to confirm brain infarction and cerebral blood vessel occlusion in the patients (Supplementary Figure S5). We identified seven conserved circRNAs that were also found in the ischemia-responsive brain tissues in mice (Memczak et al., 2015; Mehta et al., 2017) (Supplementary Tables S2, S5, S7). We then performed validation analysis in the blood of stroke patients. As shown in Figure 7B and Supplementary Figure 4B, circPHKA2 (hsa_circ_0090002) and circBBS2 (hsa_circ_0039457) showed significant differential expression (Figure 7B). Since we found that circPHKA2 exhibited an interacting sequence with miR-1962, we used TargetScan software to predict its target genes (Supplementary Table S10) and verified the expression of top-ranked potential target genes in the brain tissue by RT-qPCR. As shown in Supplementary Figure S8, SH3PXD2A, NNT, VPS26A, and TMUB2 were differentially expressed, especially in the peri-infarct brain tissue. These data suggest that circPHKA2-miR1962 interaction is likely to have a functional role in the regulation of its target genes in the ischemic brain. Taken together, all these analyses suggest that circPHKA2 and circBBS2 are diagnostic biomarkers for acute ischemic stroke.

## Discussion

In this study, we profiled the differentially expressed blood circRNAs in a mouse model of focal ischemic stroke. Through the combination of experimental study and bioinformatics analysis, circPHKA2 and circBBS2 were identified to be differentially expressed specifically in ischemic stroke patients, making these two circRNAs potential diagnostic biomarkers for acute ischemic stroke. The PHKA2 gene expresses the alpha subunit of phosphorylase b kinase (PBK) (Brushia and Walsh, 1999). This kinase is found in various tissues, with particularly high expression in the liver and muscles (Mehta et al., 2017). It plays an important role in cellular energy metabolism, as PBK activates glycogen phosphorylase, which catabolizes glycogen when glucose is deprived (Brushia and Walsh, 1999). During ischemic stroke conditions, glucose deprivation in the brain tissues is unlikely to influence glycogen metabolism, as it is not considered to be the energy source in the brain tissues. Interestingly, we found that circPHKA2 may interact with miR-1962 and subsequently regulate the expression of the circPHKA2-miR-1962 target genes. Thus, it will be interesting to further delineate the functional role of circPHKA2 after stroke in future research. Regarding BBS2, it is a member of the Bardet-Biedl syndrome (BBS) gene family. It has been reported that BBS proteins cooperate with GTPase Rab8 to promote ciliary membrane biogenesis and are involved in intracellular trafficking via microtubule-related transport (Nachury et al., 2007). Currently, the exact function of BBS2 in the brain cells after acute ischemic stroke remains unclear, and we did not identify any potential miRNA interacting with this circRNA according to our bioinformatics analysis. Further work will be required to identify the pathophysiological role of circPHKA2 and circBBS2 in acute ischemic stroke. In addition, given the statistically significant differential expression of their circular isoforms in stroke patients (Figure 7B), the results warrant further research into the clinical application of these two circRNAs as biomarkers in acute ischemic stroke.

It has been reported that circRNAs can pass through the blood–brain barrier (BBB) and enter the bloodstream (Memczak et al., 2015). Acute ischemic stroke could further facilitate this process because the BBB becomes leakier during the acute phase of stroke. Our study further supported this notion by showing that most of the differentially expressed circRNAs we identified in blood were also detected in the brain at the respective time points. In addition, we used RNA-sequencing analysis of circRNAs in the brain tissues and found that among the 20 top-ranked differentially expressed circRNAs we identified in the brain tissue, 12 of them were detected in the blood (data not shown). Furthermore, our bioinformatics analyses of the blood circRNAs reveal many pathways that are highly related to brain pathophysiology, including glutamate synapse and Alzheimer’s disease. We also showed that the predicted circRNA-miRNA interactions are likely to have a functional role in terms of regulation of their target gene expression in the brain. Taken together, these lines of evidence strongly suggest that brain circRNAs are one of the major sources of blood circRNAs. This study also reveals that the blood circRNAs are informative surrogates for brain circRNAs after acute ischemic stroke. Future studies will be required to demonstrate the direct causal relationship between the brain and blood circRNAs.

In addition to showing them to be biomarkers, the results on the circRNAs and their associated pathways identified in this study may provide insight into their roles in stroke pathophysiology. Our bioinformatics analysis of the parental genes of the identified circRNAs at 5 min of MCAO demonstrated that the PDGF and chemokine pathways, among others, are responsive to the non-damaging ischemic stimulus. Consistently, it has been reported that PDGF and its receptor are expressed in microvascular endothelial cells (Beitz et al., 1991). The PDGF pathway also affects the BBB integrity and influx of inflammatory cells into the brain (Folestad et al., 2018). Microglia are known to mediate PDGF activation and increase cerebrovascular permeability (Su et al., 2017). These observations raise the possibility that the PDGF pathway may play multiple roles in the regulation of angiogenesis, immune response, and neurovascular protection. With respect to the analysis of circRNA-miRNA target genes after 5 min of MCAO, we found that they are associated with the base excision repair, Hippo, and PPAR pathways. Gene Ontology analysis revealed that these circRNA-regulated genes are also associated with antioxidant activity. These results suggest that they may play important roles in adaptive responses to the subsequent stroke.

Penumbra is typically formed during the first 3 h after ischemia (Lo, 2008). The GeneCoDIS3 pathway analysis of the differentially expressed circRNAs at the 3 h time point reveals that the annotated enriched pathways are associated with the glutamatergic synapse and calcium signaling pathways. It is known that stroke causes excitotoxicity to neurons (Brassai et al., 2015) mainly caused by activation of calcium-dependent mechanisms at the glutamatergic synapses (Curcio et al., 2016). Our results further indicated that the blood circRNAs can reflect the pathophysiological responses in the brain penumbra tissues. Interestingly, our KEGG analysis of the circRNA-regulated genes reveals that glycosphingolipid biosynthesis, extracellular matrix-receptor interaction, and protein processing in endoplasmic reticulum might take place in the penumbra tissues. Whether these previously unknown changes to molecular events in the brain lead to subsequent excitotoxic brain cell death or salvation of penumbra tissues requires detailed follow-up studies in the future.

For the blood circRNAs identified at 24 h, we found that fatty acid metabolism and calcium signaling are enriched, as revealed by our bioinformatics analysis. This is consistent with the known pathological change in the brain damage induced by ischemic stroke (Broughton et al., 2009). Therefore, our results suggest that the blood circRNA expression profiles can reflect brain responses after ischemic stroke. Interestingly, we also identified pathways that are potentially involved in repair and regeneration after stroke. For example, we identified the Wnt and cadherin signaling pathways, which are known to play a role in axonal repair and regeneration in adult optic nerve and spinal cord injury (Garcia et al., 2018). However, whether and how the repair responses can be initiated as early as 24 h after stroke remains to be determined in future studies.

## Data Availability Statement

The datasets analyzed for this study can be found in the GEO Repository (https://www.ncbi.nlm.nih.gov/geo/query/acc.cgi?acc =GSE138849).

## Ethics Statement

The studies involving human participants were reviewed and approved by The First Affiliated Hospital, Jinan University.

## Author Contributions

DL designed and performed experiments, prepared figures, analyzed data, and wrote, edited, and proofread the manuscript. EH analyzed and interpreted data, prepared figures, and wrote, edited, and proofread the manuscript. HM, JZ, YaL, and YuL performed experiments. BY and YD assisted with patient studies. CT conceptualized the project and wrote, edited, and proofread the manuscript. AX conceptualized and directed the overall project.

## Conflict of Interest

The authors declare that the research was conducted in the absence of any commercial or financial relationships that could be construed as a potential conflict of interest.

## Footnotes

1 http://bio.tools/genecodis3

2 http://regrna2.mbc.nctu.edu.tw/
