# Supplementary material for: Identification of Blood Circular RNAs as Potential Biomarkers for Acute Ischemic Stroke
Source: Front Neurosci. 2020 Feb 6;14:81. doi: 10.3389/fnins.2020.00081 (PMC7015875; doi:10.3389/fnins.2020.00081)
Supplement: Supplementary file 1 [file Data_Sheet_1.pdf]

Supplementary Material

1 Supplementary Figures

Supplementary Figure S1

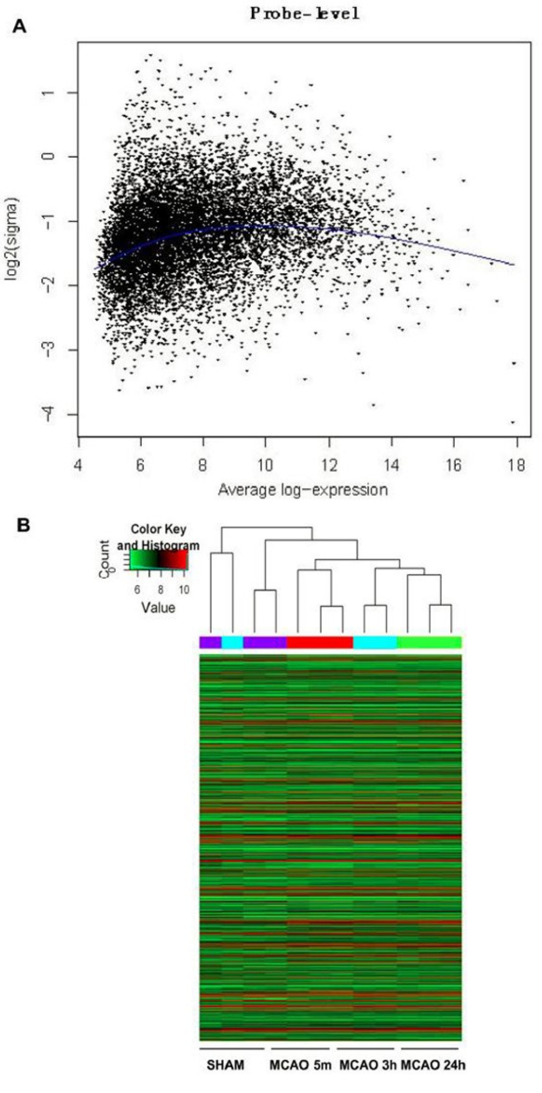

Figure supplementary 1. Quality control of the microarray probes and the heatmap of distinguishable circRNA expression profiling. A. Each dot represents a probe. Y-axis shows the log of variance of a probe intensity among replicates. X-axis shows the average log intensity of the probe's brightness (i.e. expression level). B. The result of hierarchical clustering shows distinguishable circRNA expression profiles among 12 samples of three biological replicates containing SHAM, MCAO 5min, MCAO 3h and MCAO 24h groups.

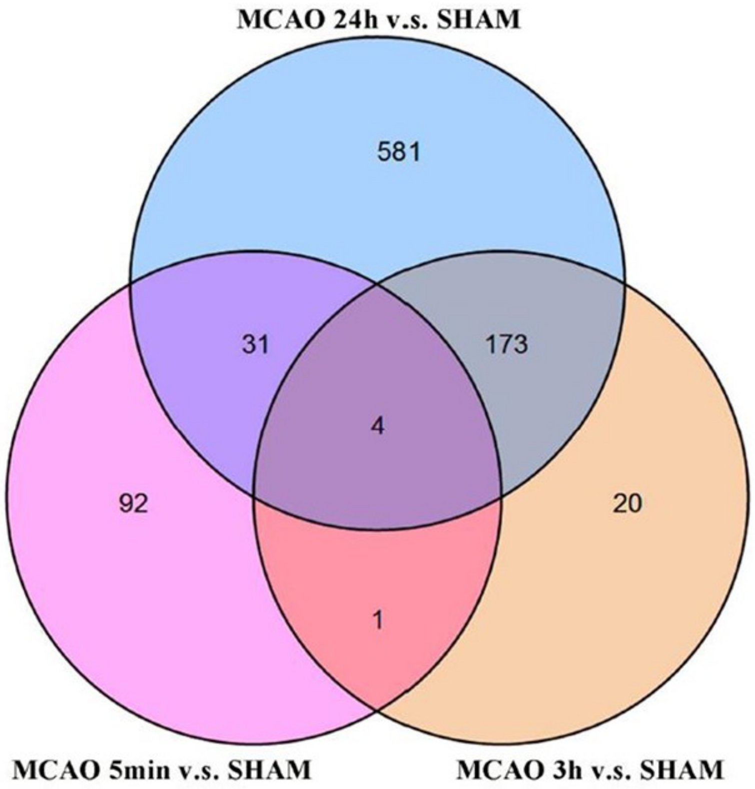

Figure supplementary 2. Venn diagram. The Venn diagram of the significant differentially expressed circRNAs (≥2.0-fold, $p<0.05$) in the MCAO groups at 5 min, 3 h and 24 h, respectively.

Supplementary Figure S3

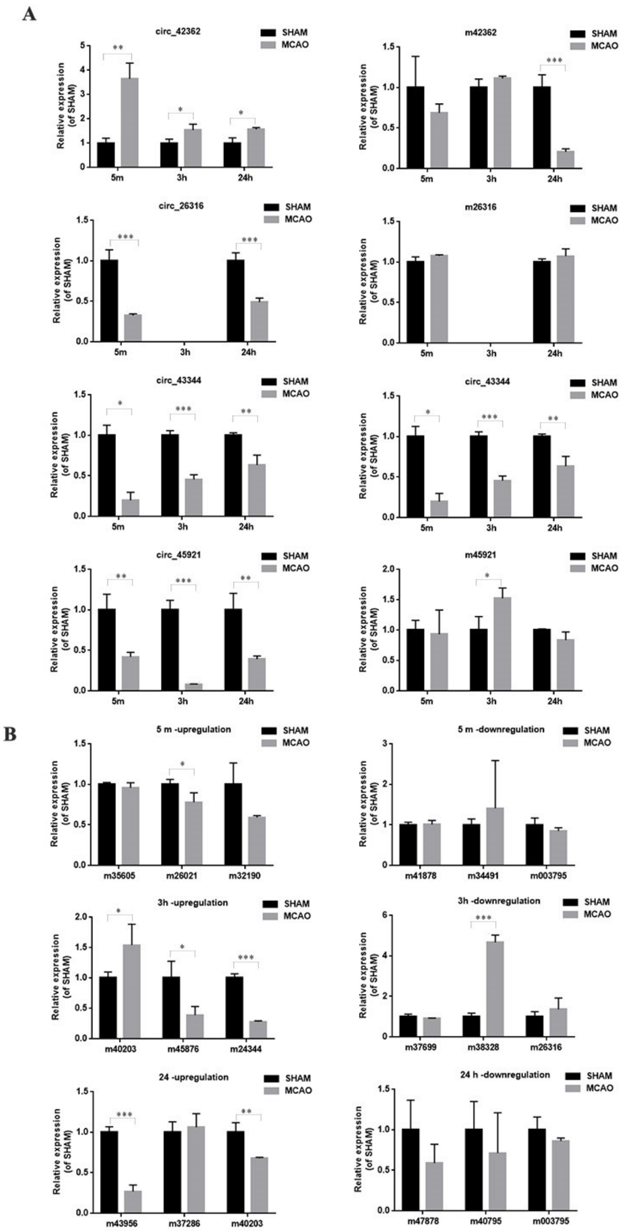

Figure supplementary 3. Changes of circRNA expression is independent to their corresponding mRNA. (A) The expression of 4 overlapped (circRNAs mmu_circRNA_42362, mmu_circRNA_26316, mmu_circRNA_43344 and mmu_circRNA_45921) and their respective mRNAs in all three time points of MCAO. Since the data of mmu_circRNA_26316 of MCAO 3h has been shown in the Figure3, this data are omitted. (B) Corresponding mRNA expression of the indicated circRNAs determined by RT-qPCR. Values are expressed as the means±S.E.M., *P<0.05,P<0.01, and ***p<0.001 were considered as statistically significant compared to SHAM contro**

Supplementary Figure S4

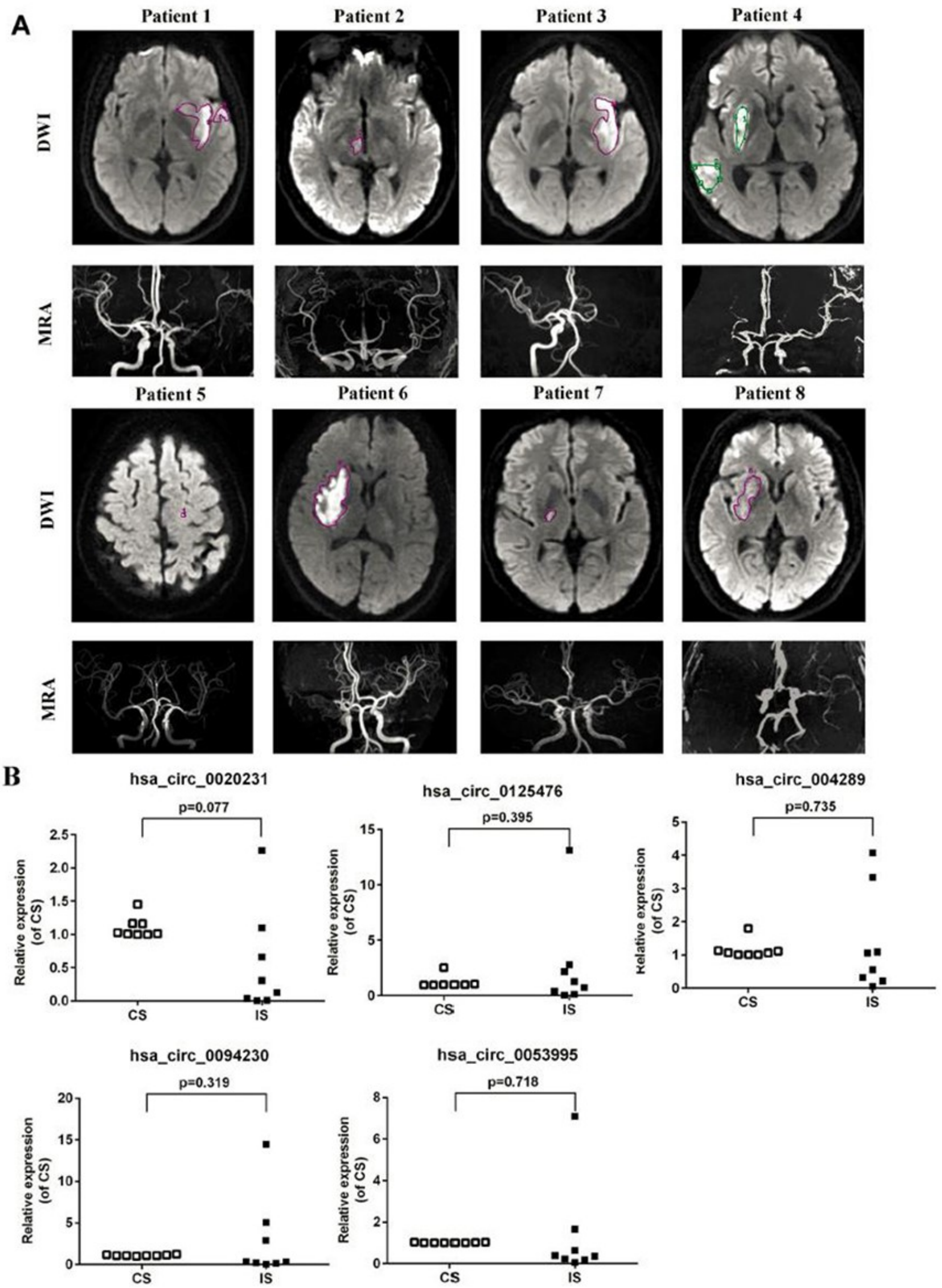

Figure supplementary 4. A. The Diffusion weighted imaging (DWI) and Magnetic Resonance Angiography (MRA) images of eight acute ischemic stroke patients. B. Validation of circRNAs in acute ischemic stroke patients by RT-qPCR. P>0.05 was considered no statistical significance.

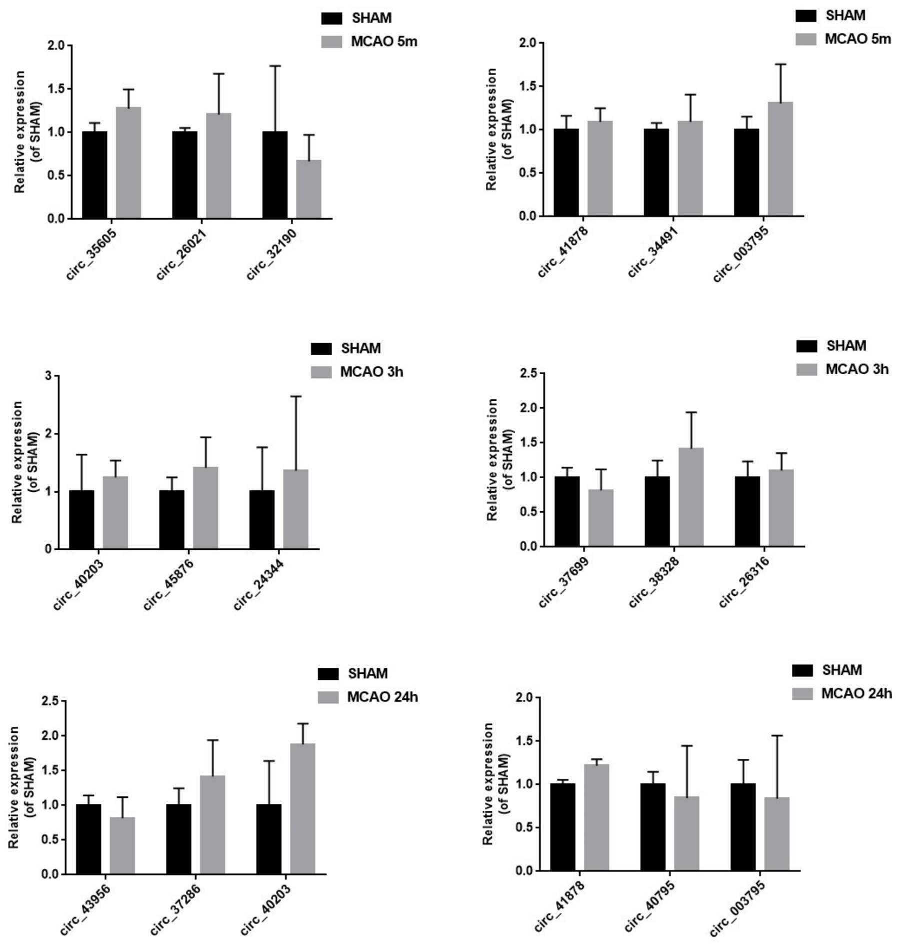

Figure Supplementary 5. RT-qPCR verification of the representative circRNAs in mouse brain tissues. Mice were subjected to MCAO for 5 min (upper panel), 3h (middle panel) and 24h (lower panel), and the respective brain tissues were collected as described in the Experimental Section. CircRNAs in the brain tissues were verified by RT-qPCR. Left and right panels show the circRNAs with upregulation and down-regulation, respectively. Values are mean ± SEM (n=3 per group).

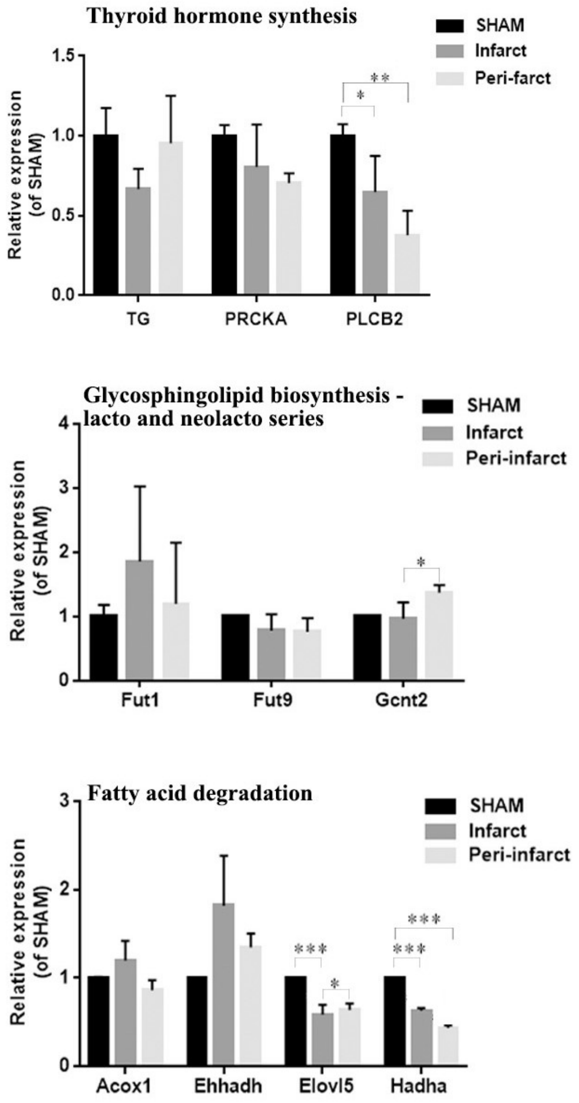

Supplementary Figure S6. Verification of circRNA-miRNA target gene expression. Mice were subjected to MCAO and the brain tissues were collected from sham, infarct core, and peri-infarct region. Expression of the representative circRNA-miRNA target genes in different brain tissues were determined by RT-qPCR. Values are mean ± SEM (n=3 per group). *$p<0.05$, **$p<0.01$, ***$p<0.001$ compared with sham (Student's t test).

Supplementary Figure S7

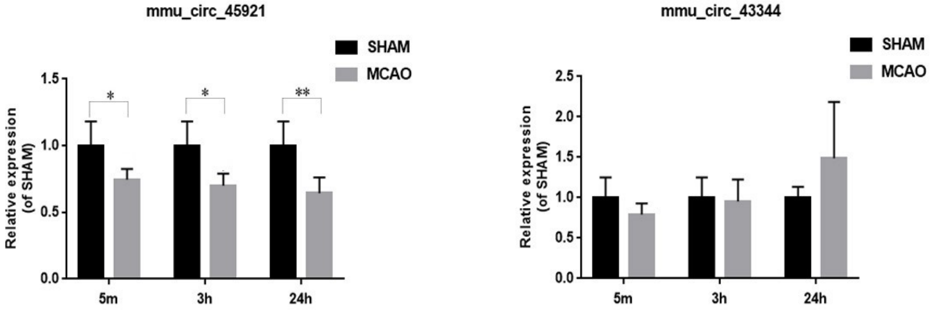

Figure Supplementary 7. Expression of circPHKA2 and circBBS2 in the mouse brain tissues. Mice were subjected to MCAO for the indicated time points, and the respective brain tissues were collected as described in the Experimental Section. CircRNAs expression in the brain tissues were verified by RT-qPCR. Left and right panels show the expression of circPHKA2 (mmu_circ_45921) and circBBS2 (mmu_circ_43344), respectively. Values are mean ± SEM (n=3 per group). *$p< 0.05$, **$p<0.01$ compared with sham (Student's t test).

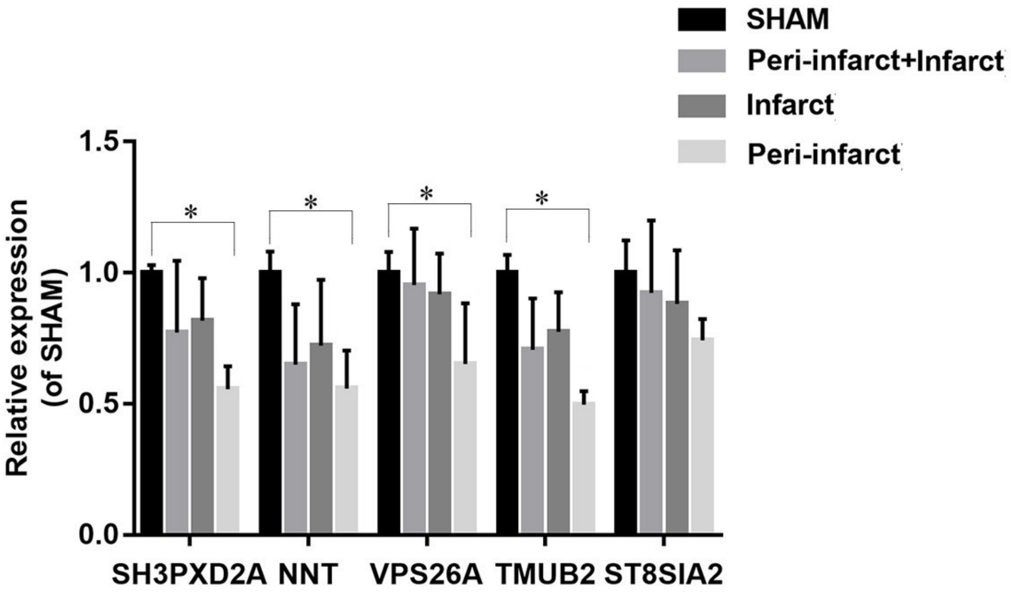

Supplementary Figure S8. Expression of circPHKA2-miR1962 target genes in brain tissues. Mice were subjected to MCAO for 24h and brain tissues were collected from sham, infarct, and peri-infarct regions. The circPHKA2-miR1962 target gene candidates were predicted by TargetScan and indicted in the x-axis. Their expression was determined by RT-qPCR. Values are mean ± SEM (n=3 per group). *$p<0.05$ compared with sham (Student's t test).

2 Supplementary Tables

Supplementary Table S1. List of primers used for qPCR.

name	Forward primer	Reverse Primer
mmu_circ_35605	CTGGCTTCCAGGAGTGAGAC	CGAACAACCAAAGGGACAG
mmu_circ_26021	CACTGTCCCTTCAGGAGAGC	TCCCCTGTGGTATCCTCTTG
mmu_circ_32190	TTGCCCGTTACTACCTGACC	CCAAGGACATGAGCATAGCA
mmu_circ_41878	GCGGCTATGGAAGATGACTC	CCACGCTCCACTATGTCTGA
mmu_circ_34491	TGCTGTTGTGTGGGCTGTAT	ACCACCTTCCTGTGCAATTC
mmu_circ_003795	CCTTAGCACCTGCCTTCTTG	CAGCTGCATCTTCACACACA
mmu_mrna_35605	AGGATTGAGAAGGCAGCAAA	TCTGGCTTCGTCTTGACCTT
mmu_mrna_26021	GCTGGTGGACCATACATCCT	ATCACCGCCAATGAGATAGC
mmu_mrna_32190	GCTGGGTACCATGCCTTCTA	CCACACAGGGTGTGATGAAG
mmu_mrna_41878	GCCTTCACTGGCTACTCTGG	CACTCCACTTCGCAGCATTA
mmu_mrna_34491	GAATTGCACAGGAAGGTGGT	TCAAAGTTAGGCCAGGATGG
mmu_mrna_003795	CTTTGGGCACCACCTAAAAA	AACGTTTGAAGGCCAAAATG
mmu_circ_40203	AGTGGCCATCATCCTCTTTG	GGGATGGTTGCTGAGGAGTA
mmu_circ_45876	CCAGCCCACTACTCATTGCT	CCAGGTGAGCAGACTGTGAA
mmu_circ_24344	AAATGGCGATGTGAAAGAGG	TGACTCCGTCGTTTTGACAG
mmu_circ_37699	AAGAACGCGTGCTGATACCT	GGGGGTCCAAAGACTTCAAT
mmu_circ_38328	GGAATGGAAACCCTGGAACT	CCTCGTCAGCAGCTCTTCTT
mmu_circ_26316	GCAGCAAGGCTATCTCCAAG	ACCTCTGTGTGGAGGTGGAC
mmu_mrna_40203	GACGTGTGGTTTCCCTCACT	CATCCCAGGGAAAGAGATGA
mmu_mrna_45876	GGGTGGGGGACTATGAAGAT	TTGCAAACTAGGCAGCAATG
mmu_mrna_24344	CTGTCAAAACGACGGAGTCA	GAGCACGCTCAATTTCAACA

mmu_mrna_37699	AAGAACGCGTGCTGATACCT	GGGGGTCCAAAGACTTCAAT
mmu_mrna_38328	ATGACCAATCCGGAGAACAG	TCTGGCTCATCCCCATATTC
mmu_mrna_26316	GCAAAGAAGGCTTTGTCCTG	TTCAATGCTTGCCTCAAGTG
mmu_circ_43956	GGGGAAGAGAGAGGGAGAGA	CCTGCTCAGTGCAACCAGTA
mmu_circ_37286	TTGCAGGAGTCCTTGCTTCT	GGGTTTCCTCTTCCCATCTC
mmu_circ_40795	AGGGAAGTCCCCAAATCAAC	AGGCTTCATGCCTACCTTCA
mmu_circ_003795	CCTTAGCACCTGCCTTCTTG	CAGCTGCATCTTCACACACA
mmu_mrna_43956	CCCTCAAATAGCCACGTGTT	TGCATTGCCTTTAAGCCTCT
mmu_mrna_37286	CTGTGGCCTTACCGTGTTTT	GGCTTCACGAGGACAGAGAC
mmu_mrna_40795	CAGAACGCATGTGCTGAGAT	ACCTCTTGGCCAGATTTCCT
mmu_circ_42362	CTGGCAGAGGGTGTTCAGTT	AGGCACCTCTGCAAGAGAGA
mmu_circ_43344	GTATGGCAGTCGGTTTGGTT	GAGGGCTCTGGAACACTCTG
mmu_circ_45921	ATCGGTGATCCACGTCCTAC	TATCCTCATCACGGTCAGCA
hsa_circ_0020231	CTGGCAGAGGGTGTTCAGTT	AGGCACCTCTGCAAGAGAGA
hsa_circ_0039457	ATGGCAGTCGGTTTGGTTAC	GAGGGCTCTGGAACACTCTG
has_circ_0090002	ATCGGTGATCCACGTCCTAC	TATCCTCATCACGGTCAGCA
Fgf1	CAGAGCAGACAGAGTGCTGAG	GCTGCAGTAGAGCAGTTTGG
Nf2	ACCTTTCACTTCCTGGCCAAA	GGAGGAATTCCTCTTGGGCT
Ppp2a	AAGGTTCGTTACCGAGAGCG	GCTCGGATGTGATCCAGTGT
Trp73	CTAGCCACAGAGCCTCAACC	CATGGGGTCACCGACGTAAA
Yap1	CCCTCGTTTTGCCATGAACC	TCCGTATTGCCTGCCGAAAT
Bmp2	CACCCCAAGACACAGTTCCC	AACACTAGAAGACAGCGGGTC
Gli2	GCCTGAATAGACTGTGGGTGAA	TGCACCAAATTTACTGCCTGGA
Gcnt2	AATGGAGCTGGGATGGAGGA	TAGGCGTGTGAAGGGTTTCC

Fut9	ATCCCATGCGGTCCTGATTC	AAGGGATTTGTGCTCACCGT
Fut1	ACCAGCAAGGAGCTCAGACTA	GGCATGCATCTCAGGTTGGA
Hadha	ATTGTGGTCAAGGACGGACC	ACATCCACACCCACTTCGTC
Elovl5	CAGCTTGCTTCTGTTCCCG	GTCCAGGAGGAACCATCCTTT
Acox1	ATTTTGTGGAACCTGTTGGCCT	CCTCGAAGATGAGTTCCGTGG
Ehhadh	ACATCAGAGGCGCAGGATAC	ATGGCTCTAACCGTATGGTCC
VPS26A	CGGCGGTGACAATGAGTTTT	TGAAAAGCTCTTTCCTGAGACA
ST6SIA2	AATCGGGAATTCTGGAGGCAG	TCCTCAGAGAGAGXGTCTGG
TMUB2	GACTGTCCTGGCGAAGTCAA	TCCACACCCCTCAAACTCG
SH3PXD2A	CCAAGACTGGGGAAGAAGAGAA	GGCCTTCTTCAGGTAGGACG
NNT	GCTGTAAGCGTGCAGGACG	GCTGTAAGCGTGCATCTGGT
Prkca	AATCTGGGACTGGGATCGGA	GGCCTAGCTTHHCTTTCTCA
Tg	ATGCAGGTAATGGAGGCCAAGG	AAGATGGGAGGTCTCAGGT
Plcb2	GGTTTGCCCAACCTCTCCAA	CCGGAGTTTCTGGCTCTGATT
β-actin (mouse)	CGTTGACATCCGTAAAGACC	AACAGTCCGCCTAGAAGCAC
β-actin (human)	CGACAGGATGCAGAAGGAG	ACATCTGCTGGAAGGTGGA

Supplementary Table S2. List of top 10 up- and down-regulated differentially expressed circRNAs in the three time points.

Group	ProbeName	adj.P.Val	GeneSymbol	Type	logFC
MCAO 5 min	mmu_circRNA_35065	0.0287	Dido1	exonic	2.48
MCAO 5 min	mmu_circRNA_42362	0.0468	Wdr11	exonic	2.4
MCAO 5 min	mmu_circRNA_011174	0.0145	NA	sense overlapping	2.35
MCAO 5 min	mmu_circRNA_35587	0.0144	Gm20754	intronic	2.22
MCAO 5 min	mmu_circRNA_35588	0.0246	Gm20754	intronic	2.19
MCAO 5 min	mmu_circRNA_43955	0.047	Ets1	exonic	2.17
MCAO 5 min	mmu_circRNA_26021	0.0353	Ryr2	exonic	2.09
MCAO 5 min	mmu_circRNA_32190	0.0145	Slc29a2	exonic	2.07
MCAO 5 min	mmu_circRNA_20066	0.0157	Gm553	sense overlapping	2.03
MCAO 5 min	mmu_circRNA_35589	0.0167	Gm20754	intronic	2.01
MCAO 5 min	mmu_circRNA_010803	0.0151	Raf1	exonic	-3.08
MCAO 5 min	mmu_circRNA_43867	0.0018	Bbs9	exonic	-3.12
MCAO 5 min	mmu_circRNA_25433	0.0195	Pcnx	exonic	-3.15
MCAO 5 min	mmu_circRNA_45921	0.0144	Phka2	exonic	-3.16
MCAO 5 min	mmu_circRNA_22898	0.0198	Zpbp	exonic	-3.18
MCAO 5 min	mmu_circRNA_003169	0.0075	Arhgap10	exonic	-3.18
MCAO 5 min	mmu_circRNA_43344	0.0197	Bbs2	exonic	-3.27
MCAO 5 min	mmu_circRNA_003795	0.0145	Cep350	sense overlapping	-3.28
MCAO 5 min	mmu_circRNA_34491	0.0145	Sppl2a	exonic	-3.3
MCAO 5 min	mmu_circRNA_41878	0.0145	Ankrd42	exonic	-4.07
MCAO 3 h	mmu_circRNA_40203	0.005	Tmem178b	sense overlapping	4.49
MCAO 3 h	mmu_circRNA_36481	0.005	Rad54b	exonic	4.27

MCAO 3 h	mmu_circRNA_45876	0.005	Cnksr2	exonic	4.21
MCAO 3 h	mmu_circRNA_24344	0.005	Psmd12	exonic	4.17
MCAO 3 h	mmu_circRNA_27197	0.005	Samd8	exonic	4.15
MCAO 3 h	mmu_circRNA_010777	0.005	Kcnma1	exonic	4.01
MCAO 3 h	mmu_circRNA_27565	0.006	Tm9sf1	exonic	3.93
MCAO 3 h	mmu_circRNA_43956	0.005	Kirrel3	intronic	3.89
MCAO 3 h	mmu_circRNA_19178	0.005	Ermard	sense overlapping	3.79
MCAO 3 h	mmu_circRNA_29760	0.005	Spice1	exonic	3.79
MCAO 3 h	mmu_circRNA_38234	0.012	5031425E22Rik	exonic	-2
MCAO 3 h	mmu_circRNA_38234	0.012	LOC102635844	exonic	-2
MCAO 3 h	mmu_circRNA_38760	0.007	Corin	exonic	-2.07
MCAO 3 h	mmu_circRNA_26316	0.045	LOC102631805	intergenic	-2.1
MCAO 3 h	mmu_circRNA_38328	0.006	Rbm33	exonic	-2.26
MCAO 3 h	mmu_circRNA_23643	0.047	Spns2	exonic	-2.42
MCAO 3 h	mmu_circRNA_43344	0.041	Bbs2	exonic	-2.52
MCAO 3 h	mmu_circRNA_45921	0.008	Phka2	exonic	-2.83
MCAO 3 h	mmu_circRNA_37699	0.043	Khdrbs1	exonic	-3.03
MCAO 3 h	mmu_circRNA_002246	0.018	Igf2	sense overlapping	-3.04
MCAO 24 h	mmu_circRNA_43956	0	Kirrel3	intronic	7.461
MCAO 24 h	mmu_circRNA_37286	0	Dab1	intronic	6.79
MCAO 24 h	mmu_circRNA_40203	0	Tmem178b	sense overlapping	6.28
MCAO 24 h	mmu_circRNA_31169	0	NA	intergenic	5.85
MCAO 24 h	mmu_circRNA_19077	0	Adck1	intronic	5.79
MCAO 24 h	mmu_circRNA_45876	0	Cnksr2	exonic	5.72

MCAO 24 h	mmu_circRNA_45047	0	Manf	antisense	5.59
MCAO 24 h	mmu_circRNA_24344	0	Psmd12	exonic	5.55
MCAO 24 h	mmu_circRNA_22526	0	NA	sense overlapping	5.51
MCAO 24 h	mmu_circRNA_30884	0	Xdh	exonic	5.38
MCAO 24 h	mmu_circRNA_37699	0.001	Khdrbs1	exonic	-4.29
MCAO 24 h	mmu_circRNA_35175	0.001	Spata16	exonic	-4.3
MCAO 24 h	mmu_circRNA_39121	0	Evi5	exonic	-4.35
MCAO 24 h	mmu_circRNA_45921	0	Phka2	exonic	-4.42
MCAO 24 h	mmu_circRNA_003795	0	Cep350	sense overlapping	-4.51
MCAO 24 h	mmu_circRNA_002246	0	Igf2	sense overlapping	-4.67
MCAO 24 h	mmu_circRNA_010803	0	Raf1	exonic	-4.73
MCAO 24 h	mmu_circRNA_40795	0	Tmcc1	exonic	-4.77
MCAO 24 h	mmu_circRNA_015506	0	Crim1	exonic	-5.7
MCAO 24 h	mmu_circRNA_41878	0	Ankrd42	exonic	-6.57

Supplementary Table S3. List of significnatly differentially expressed circRNAs.

Groups	circRNA	chrom	strand	txStart	txEnd	circRNA_type	logFC	AveExpr	t	P.Value	adj.P.Val	GeneSymbol
MCAO 5m *v.s.* SHAM	mmu_circRNA_001149	chr12	+	52937134	52938506	exonic	-2.887	9.67614	-6.89	1.01E-05	0.011299	Akap6
MCAO 5m *v.s.* SHAM	mmu_circRNA_001579	chr3	-	56079858	56091120	exonic	-2.954	7.59162	-4.61	0.00046898	0.026424	Nbea
MCAO 5m *v.s.* SHAM	mmu_circRNA_001729	chr9	-	96591949	96611499	exonic	-2.054	11.2543	-5.05	0.0002133	0.019755	Rasa2
MCAO 5m v.s. SHAM	mmu_circRNA_001848	chr11	+	70874745	70884968	exonic	-2.106	10.3305	-3.78	0.0022453	0.046084	Rabep1
MCAO 5m v.s. SHAM	mmu_circRNA_002352	chr1	+	139039999	139062971	exonic	-2.003	7.85573	-4.33	0.00078792	0.032538	Dennd1b
MCAO 5m v.s. SHAM	mmu_circRNA_002644	chrX	-	98626913	98644994	exonic	-2.021	7.007	-5.41	0.00011272	0.015405	Ophn1
MCAO 5m v.s. SHAM	mmu_circRNA_003169	chr8	-	77310730	77365160	exonic	-3.181	10.5202	-7.74	2.89E-06	0.007452	Arhgap10
MCAO 5m v.s. SHAM	mmu_circRNA_003795	chr1	-	155847109	155848727	sense overlapping	-3.276	11.5818	-5.76	6.25E-05	0.014544	Cep350
MCAO 5m v.s. SHAM	mmu_circRNA_004072	chr10	+	84947411	84948020	exonic	-3.071	10.2347	-6.08	3.65E-05	0.014378	Ric8b
MCAO 5m v.s. SHAM	mmu_circRNA_004868	chr10	-	12439626	12455564	exonic	-2.535	10.1668	-5.49	9.74E-05	0.015375	Utrn
MCAO 5m v.s. SHAM	mmu_circRNA_004975	chr15	+	95912304	95920378	exonic	-2.393	8.56951	-5.82	5.57E-05	0.014544	Ano6
MCAO 5m v.s. SHAM	mmu_circRNA_005547	chr1	-	53256628	53290423	exonic	-2.021	8.77203	-4	0.0014534	0.038915	Pms1
MCAO 5m v.s. SHAM	mmu_circRNA_006097	chr2	+	92214048	92230724	exonic	-2.523	11.7045	-5.31	0.00013308	0.016192	Phf21a
MCAO 5m v.s. SHAM	mmu_circRNA_006404	chr9	+	22643744	22679064	exonic	-2.39	9.96291	-5.84	5.42E-05	0.014544	Bbs9
MCAO 5m v.s. SHAM	mmu_circRNA_007853	chr10	+	14004200	14086082	sense overlapping	-2.285	12.3794	-4.8	0.00032944	0.023034	Hivep2
MCAO 5m v.s. SHAM	mmu_circRNA_007906	chr3	+	122747888	122748477	exonic	-2.403	11.1222	-4.05	0.00133141	0.038322	Pde5a
MCAO 5m v.s. SHAM	mmu_circRNA_008057	chr9	-	66540061	66566903	exonic	-2.686	8.84259	-4.34	0.0007715	0.032274	Usp3
MCAO 5m v.s. SHAM	mmu_circRNA_009056	chr10	-	12436280	12455564	exonic	-2.378	11.0566	-4.38	0.00072002	0.031661	Utrn
MCAO 5m v.s. SHAM	mmu_circRNA_009396	chr6	+	31418930	31433131	exonic	-2.774	11.6581	-5.02	0.00022486	0.019786	Mkln1

MCAO 5m v.s. SHAM	mmu_circRNA_009558	chr1	+	139053345	139062971	exonic	-2.092	9.44197	-3.94	0.00163453	0.040578	Dennd1b
MCAO 5m v.s. SHAM	mmu_circRNA_009674	chr5	+	34342521	34350838	exonic	-2.063	10.0777	-3.65	0.00285335	0.04976	Rnf4
MCAO 5m v.s. SHAM	mmu_circRNA_010337	chr3	-	153411461	153411871	exonic	-2.056	11.2999	-5.29	0.00013802	0.016192	St6galnac3
MCAO 5m v.s. SHAM	mmu_circRNA_010803	chr6	-	115634552	115644642	exonic	-3.079	11.5318	-5.56	8.65E-05	0.015139	Raf1
MCAO 5m v.s. SHAM	mmu_circRNA_011174	chr17	-	39846503	39846663	sense overlapping	2.345	6.2875	5.753	6.27E-05	0.014544	NA
MCAO 5m v.s. SHAM	mmu_circRNA_012978	chr17	-	24624819	24625014	intronic	-2.502	13.128	-3.84	0.00200336	0.044575	Tsc2
MCAO 5m v.s. SHAM	mmu_circRNA_013636	chr9	+	8634046	8658377	exonic	-2.797	11.3865	-5.55	8.89E-05	0.015142	Trpc6
MCAO 5m v.s. SHAM	mmu_circRNA_013908	chr3	+	122794259	122818067	exonic	-2.506	9.68296	-5.93	4.68E-05	0.014378	4930447N08Rik
MCAO 5m v.s. SHAM	mmu_circRNA_013908	chr3	+	122794259	122818067	exonic	-2.506	9.68296	-5.93	4.68E-05	0.014378	Pde5a
MCAO 5m v.s. SHAM	mmu_circRNA_014005	chr3	+	59037559	59093695	exonic	-2.681	12.0878	-4.38	0.00072101	0.031661	Med12l
MCAO 5m v.s. SHAM	mmu_circRNA_015239	chr12	+	4961217	5006791	exonic	-2.452	9.42344	-4.93	0.0002648	0.021192	Atad2b
MCAO 5m v.s. SHAM	mmu_circRNA_015986	chr8	-	77344639	77365160	exonic	-2.153	10.3786	-5.46	0.000103	0.015375	Arhgap10
MCAO 5m v.s. SHAM	mmu_circRNA_016182	chr9	+	80087429	80103764	exonic	-2.011	10.4465	-5.18	0.00016713	0.018163	Senp6
MCAO 5m v.s. SHAM	mmu_circRNA_016331	chr2	+	92356863	92359209	exonic	-2.439	7.66482	-5.64	7.63E-05	0.014544	Phf21a
MCAO 5m v.s. SHAM	mmu_circRNA_017784	chr6	+	31426713	31433131	exonic	-2.245	9.69551	-4.56	0.0005152	0.027703	Mkln1
MCAO 5m v.s. SHAM	mmu_circRNA_18953	chr1	-	119741351	119765923	sense overlapping	-2.065	9.77486	-4.35	0.00076299	0.032274	Ptpn4
MCAO 5m v.s. SHAM	mmu_circRNA_18967	chr10	-	12436280	12455565	sense overlapping	-2.363	10.9141	-4.34	0.00077528	0.032274	Utrn
MCAO 5m v.s. SHAM	mmu_circRNA_18979	chr10	-	52544603	52545267	sense overlapping	-3.069	10.788	-6.49	1.88E-05	0.011608	Zfx
MCAO 5m v.s. SHAM	mmu_circRNA_19038	chr11	+	95319176	95332371	sense overlapping	-2.244	8.91513	-5.16	0.00017416	0.018506	NA
MCAO 5m v.s. SHAM	mmu_circRNA_19252	chr2	+	92214045	92230725	sense overlapping	-2.576	11.7163	-5.66	7.31E-05	0.014544	Phf21a
MCAO 5m v.s. SHAM	mmu_circRNA_19295	chr3	-	94519045	94531392	sense overlapping	-2.113	8.59456	-4.14	0.00112962	0.03662	Snx27

MCAO 5m v.s. SHAM	mmu_circRNA_19311	chr3	-	153411461	153411874	sense overlapping	-2.096	11.059	-5.69	6.93E-05	0.014544	St6galnac3
MCAO 5m v.s. SHAM	mmu_circRNA_19390	chr5	+	130187399	130198117	sense overlapping	-2.014	9.175	-4.68	0.00041207	0.025818	Rabgef1
MCAO 5m v.s. SHAM	mmu_circRNA_19411	chr6	+	31418929	31433132	sense overlapping	-2.78	11.6827	-4.91	0.00027016	0.021253	Mkln1
MCAO 5m v.s. SHAM	mmu_circRNA_19525	chr9	-	66540061	66566907	sense overlapping	-2.623	7.86135	-4.84	0.00030801	0.022856	Usp3
MCAO 5m v.s. SHAM	mmu_circRNA_19573	chr1	+	5089008	5150061	exonic	-2.123	8.21024	-4.14	0.00113459	0.03662	Atp6v1h
MCAO 5m v.s. SHAM	mmu_circRNA_19729	chr1	-	21424048	21469564	exonic	-3.038	8.21758	-5.31	0.00013356	0.016192	Kcnq5
MCAO 5m v.s. SHAM	mmu_circRNA_20066	chr1	+	52522221	52523151	sense overlapping	2.034	12.4253	5.384	0.00011771	0.015687	Gm553
MCAO 5m v.s. SHAM	mmu_circRNA_20290	chr1	-	66721804	66778108	exonic	-2.057	12.1603	-4.32	0.00079969	0.032693	Kansl1l
MCAO 5m v.s. SHAM	mmu_circRNA_20764	chr1	-	124633364	124639139	intronic	-2.375	8.72334	-4.58	0.00050033	0.02733	NA
MCAO 5m v.s. SHAM	mmu_circRNA_21247	chr1	+	167830545	167832894	exonic	-2.781	7.25257	-5.35	0.00012476	0.015901	Lmx1a
MCAO 5m v.s. SHAM	mmu_circRNA_21668	chr10	-	12455362	12688427	exonic	-2.181	11.1013	-5.2	0.00016214	0.017993	Utrn
MCAO 5m v.s. SHAM	mmu_circRNA_22800	chr10	-	128578099	128579092	exonic	-2.513	11.4194	-5.43	0.00010836	0.015375	Erbb3
MCAO 5m v.s. SHAM	mmu_circRNA_22898	chr11	-	11352521	11418287	exonic	-3.179	8.3935	-5.04	0.00021431	0.019755	Zpbp
MCAO 5m v.s. SHAM	mmu_circRNA_23166	chr11	+	31055457	31058999	exonic	-2.164	8.8165	-5.8	5.79E-05	0.014544	Asb3
MCAO 5m v.s. SHAM	mmu_circRNA_23845	chr11	+	80220257	80227332	exonic	-2.022	8.92519	-4.35	0.00076153	0.032274	Rhot1
MCAO 5m v.s. SHAM	mmu_circRNA_24828	chr12	+	28847556	28867153	sense overlapping	-2.024	7.80646	-5.01	0.00022628	0.019786	Tssc1
MCAO 5m v.s. SHAM	mmu_circRNA_25433	chr12	+	81946930	81967148	exonic	-3.15	7.14595	-5.12	0.00018673	0.019473	Pcnx
MCAO 5m v.s. SHAM	mmu_circRNA_26021	chr13	-	11868116	11885538	exonic	2.088	9.09262	4.205	0.00099527	0.035258	Ryr2
MCAO 5m v.s. SHAM	mmu_circRNA_26033	chr13	+	13651576	13696788	exonic	-2.729	8.77711	-7.33	5.24E-06	0.009839	Lyst
MCAO 5m v.s. SHAM	mmu_circRNA_26040	chr13	+	13725813	13761201	exonic	-2.515	7.61351	-4.67	0.00041955	0.025818	Lyst

MCAO 5m v.s. SHAM	mmu_circRNA_26205	chr13	-	36877742	36918635	exonic	-2.283	8.25587	-5.95	4.52E-05	0.014378	Gm30489
MCAO 5m v.s. SHAM	mmu_circRNA_26205	chr13	-	36877742	36918635	exonic	-2.283	8.25587	-5.95	4.52E-05	0.014378	F13a1
MCAO 5m v.s. SHAM	mmu_circRNA_26206	chr13	-	36892805	36918635	exonic	-2.412	8.9522	-6.53	1.78E-05	0.011608	F13a1
MCAO 5m v.s. SHAM	mmu_circRNA_26206	chr13	-	36892805	36918635	exonic	-2.412	8.9522	-6.53	1.78E-05	0.011608	Gm30489
MCAO 5m v.s. SHAM	mmu_circRNA_26316	chr13	+	49820612	49830616	intergenic	-2.533	8.79094	-4.62	0.00046426	0.026424	LOC102631805
MCAO 5m v.s. SHAM	mmu_circRNA_26948	chr13	-	107769559	107788009	exonic	-2.934	7.4385	-6.94	9.42E-06	0.011299	Zswim6
MCAO 5m v.s. SHAM	mmu_circRNA_27715	chr14	-	64896649	64897126	exonic	-2.304	7.57109	-3.73	0.00244005	0.046838	Hmbox1
MCAO 5m v.s. SHAM	mmu_circRNA_27968	chr14	-	103247746	103282802	exonic	-2.264	6.91609	-7.17	6.67E-06	0.009839	Mycbp2
MCAO 5m v.s. SHAM	mmu_circRNA_29302	chr16	+	15662982	15690411	exonic	-3.7	9.07667	-4.06	0.00144573	0.048506	Prkdc
MCAO 5m v.s. SHAM	mmu_circRNA_29357	chr16	+	17257334	17259073	sense overlapping	-2.523	9.29371	-6.31	2.52E-05	0.012943	Hic2
MCAO 5m v.s. SHAM	mmu_circRNA_29619	chr16	+	35267179	35283609	exonic	-2.415	12.8924	-6.17	3.17E-05	0.014244	Adcy5
MCAO 5m v.s. SHAM	mmu_circRNA_29699	chr16	+	38170663	38193994	exonic	-2.795	8.57752	-7.22	6.19E-06	0.009839	Gsk3b
MCAO 5m v.s. SHAM	mmu_circRNA_29747	chr16	-	44005459	44013037	exonic	-2.376	7.10252	-8.22	1.49E-06	0.007323	Gramd1c
MCAO 5m v.s. SHAM	mmu_circRNA_29750	chr16	-	44028678	44040158	intergenic	-2.352	6.54705	-5.92	4.74E-05	0.014378	NA
MCAO 5m v.s. SHAM	mmu_circRNA_30386	chr17	-	27596723	27596834	exonic	-2.43	7.11418	-3.7	0.00262187	0.047805	Nudt3
MCAO 5m v.s. SHAM	mmu_circRNA_30941	chr17	+	75225155	75227241	exonic	-2.6	11.6544	-4.65	0.00043997	0.026209	Ltbp1
MCAO 5m v.s. SHAM	mmu_circRNA_32017	chr18	+	77873360	77875919	exonic	-2.057	9.4629	-5.02	0.00022411	0.019786	Pstpip2
MCAO 5m v.s. SHAM	mmu_circRNA_32190	chr19	+	5027024	5027764	exonic	2.068	11.2703	5.608	8.02E-05	0.014544	Slc29a2
MCAO 5m v.s. SHAM	mmu_circRNA_32545	chr19	+	37576935	37609105	exonic	-2.096	8.04767	-4.53	0.0005451	0.028279	Exoc6
MCAO 5m v.s. SHAM	mmu_circRNA_32884	chr2	-	5354668	5362127	exonic	-2.919	11.6701	-5.42	0.00011021	0.015375	Camk1d
MCAO 5m v.s. SHAM	mmu_circRNA_33003	chr2	+	14981305	14984439	exonic	-2.866	11.6408	-6.26	2.71E-05	0.012943	Cacnb2

MCAO 5m v.s. SHAM	mmu_circRNA_33850	chr2	+	71875405	71888994	exonic	-2.397	10.0574	-6.51	1.84E-05	0.011608	Pdk1
MCAO 5m v.s. SHAM	mmu_circRNA_33851	chr2	+	71880038	71883932	exonic	-2.671	10.0236	-5.67	7.17E-05	0.014544	Pdk1
MCAO 5m v.s. SHAM	mmu_circRNA_33860	chr2	-	73141897	73156913	exonic	-2.13	6.49504	-5.84	5.41E-05	0.014544	Ola1
MCAO 5m v.s. SHAM	mmu_circRNA_34079	chr2	+	92221471	92230724	exonic	-2.454	8.97882	-5.65	7.41E-05	0.014544	Phf21a
MCAO 5m v.s. SHAM	mmu_circRNA_34491	chr2	-	126919638	126926999	exonic	-3.301	9.25167	-5.74	6.47E-05	0.014544	Sppl2a
MCAO 5m v.s. SHAM	mmu_circRNA_35065	chr2	-	180683808	180692048	exonic	2.481	11.3105	4.504	0.00056988	0.028734	Dido1
MCAO 5m v.s. SHAM	mmu_circRNA_35175	chr3	+	26878562	26927389	exonic	-2.718	7.13891	-3.67	0.00278057	0.04958	Spata16
MCAO 5m v.s. SHAM	mmu_circRNA_35587	chr3	+	73277865	73333103	intronic	2.217	8.97047	5.991	4.22E-05	0.014378	Gm20754
MCAO 5m v.s. SHAM	mmu_circRNA_35588	chr3	+	73277865	73339144	intronic	2.19	9.01322	4.743	0.00036788	0.024632	Gm20754
MCAO 5m v.s. SHAM	mmu_circRNA_35589	chr3	+	73277865	73341175	intronic	2.013	9.34021	5.261	0.00014587	0.016733	Gm20754
MCAO 5m v.s. SHAM	mmu_circRNA_35991	chr3	+	109508783	109563204	exonic	-2.063	10.4304	-4.41	0.00067361	0.031401	Vav3
MCAO 5m v.s. SHAM	mmu_circRNA_36096	chr3	+	122774552	122795321	exonic	-2.547	10.7618	-6.04	3.88E-05	0.014378	LOC108168896
MCAO 5m v.s. SHAM	mmu_circRNA_36096	chr3	+	122774552	122795321	exonic	-2.547	10.7618	-6.04	3.88E-05	0.014378	4930447N08Rik
MCAO 5m v.s. SHAM	mmu_circRNA_36096	chr3	+	122774552	122795321	exonic	-2.547	10.7618	-6.04	3.88E-05	0.014378	Pde5a
MCAO 5m v.s. SHAM	mmu_circRNA_36265	chr3	+	145124210	145132817	exonic	-2.116	8.05362	-4.08	0.00126226	0.037773	Odf2l
MCAO 5m v.s. SHAM	mmu_circRNA_36266	chr3	+	145124210	145144416	exonic	-2.242	8.143	-4.35	0.00076182	0.032274	Odf2l
MCAO 5m v.s. SHAM	mmu_circRNA_36800	chr4	+	53716779	53737659	sense overlapping	-2.381	11.9328	-6.82	1.12E-05	0.011299	Fktn
MCAO 5m v.s. SHAM	mmu_circRNA_37370	chr4	+	108486453	108503035	exonic	-2.014	7.09874	-5.68	7.11E-05	0.014544	Zcchc11
MCAO 5m v.s. SHAM	mmu_circRNA_37852	chr4	-	140067136	140157573	exonic	-2.653	11.488	-7.96	2.13E-06	0.007323	Igsf21
MCAO 5m v.s. SHAM	mmu_circRNA_38199	chr5	-	21029775	21031725	intronic	-2.172	7.91492	-4.19	0.00102735	0.035316	Ptpn12

MCAO 5m v.s. SHAM	mmu_circRNA_38721	chr5	-	67398199	67400301	sense overlapping	-2.312	5.91948	-4.83	0.00031417	0.022967	Bend4
MCAO 5m v.s. SHAM	mmu_circRNA_38722	chr5	-	67398199	67405519	sense overlapping	-2.48	11.0972	-4.1	0.00121193	0.037349	Bend4
MCAO 5m v.s. SHAM	mmu_circRNA_38722	chr5	-	67398199	67405519	sense overlapping	-2.48	11.0972	-4.1	0.00121193	0.037349	Gm33345
MCAO 5m v.s. SHAM	mmu_circRNA_38764	chr5	-	72782002	72789244	exonic	-2.411	7.53191	-6.1	3.51E-05	0.014378	Tec
MCAO 5m v.s. SHAM	mmu_circRNA_38765	chr5	-	72823455	72825130	exonic	-2.687	8.7828	-4.98	0.00023848	0.020139	Tec
MCAO 5m v.s. SHAM	mmu_circRNA_38889	chr5	-	84233582	84265639	intronic	-2.453	6.79666	-4.59	0.00048524	0.026881	Epha5
MCAO 5m v.s. SHAM	mmu_circRNA_38977	chr5	+	92170672	92194175	exonic	-2.324	7.83906	-5.59	8.20E-05	0.0146	Uso1
MCAO 5m v.s. SHAM	mmu_circRNA_38999	chr5	-	96091583	96134132	exonic	-2.13	7.77935	-4.45	0.00063439	0.0306	Cnot6l
MCAO 5m v.s. SHAM	mmu_circRNA_40539	chr6	+	87904087	87905267	exonic	-2.915	6.6404	-4.64	0.00044417	0.026209	Copg1
MCAO 5m v.s. SHAM	mmu_circRNA_40598	chr6	-	97323654	97329564	exonic	-2.75	9.04445	-6.78	1.20E-05	0.011299	Frmd4b
MCAO 5m v.s. SHAM	mmu_circRNA_40722	chr6	+	108813626	108818613	exonic	-2.104	9.06452	-4.07	0.00127397	0.037903	Arl8b
MCAO 5m v.s. SHAM	mmu_circRNA_40722	chr6	+	108813626	108818613	exonic	-2.104	9.06452	-4.07	0.00127397	0.037903	Gm35417
MCAO 5m v.s. SHAM	mmu_circRNA_40924	chr6	+	125793556	125815599	exonic	-2.141	6.92836	-5.3	0.00013567	0.016192	Ano2
MCAO 5m v.s. SHAM	mmu_circRNA_41210	chr7	+	19186101	19186371	exonic	-2.174	12.8742	-3.85	0.00193966	0.043819	Eml2
MCAO 5m v.s. SHAM	mmu_circRNA_41589	chr7	+	64512980	64553771	sense overlapping	-2.158	6.89811	-3.69	0.00263475	0.047805	Apba2
MCAO 5m v.s. SHAM	mmu_circRNA_41878	chr7	-	92584465	92631662	exonic	-4.071	10.4762	-5.77	6.09E-05	0.014544	Ankrd42
MCAO 5m v.s. SHAM	mmu_circRNA_42362	chr7	+	129629917	129632421	exonic	2.401	10.6303	3.738	0.00241912	0.046838	Wdr11
MCAO 5m v.s. SHAM	mmu_circRNA_42666	chr8	-	24878720	24902884	exonic	-2.665	7.23952	-5.34	0.0001265	0.015927	Adam32
MCAO 5m v.s. SHAM	mmu_circRNA_43161	chr8	+	81715200	81770924	exonic	-2.343	11.0896	-5.54	9.05E-05	0.015142	Inpp4b
MCAO 5m v.s. SHAM	mmu_circRNA_43168	chr8	+	82010538	82071835	exonic	-2.347	8.50999	-4.55	0.00052843	0.027834	Inpp4b
MCAO 5m v.s. SHAM	mmu_circRNA_43246	chr8	+	85946051	85957654	exonic	-2.273	7.35741	-6.25	2.76E-05	0.012943	Phkb

MCAO 5m v.s. SHAM	mmu_circRNA_43344	chr8	-	94086655	94092622	exonic	-3.271	6.19788	-5.07	0.00020278	0.019681	Bbs2
MCAO 5m v.s. SHAM	mmu_circRNA_43741	chr9	+	8609699	8610474	exonic	-2.402	6.71172	-5.5	9.69E-05	0.015375	Trpc6
MCAO 5m v.s. SHAM	mmu_circRNA_43742	chr9	+	8643505	8658377	exonic	-2.455	10.7079	-4.89	0.00028424	0.021737	Trpc6
MCAO 5m v.s. SHAM	mmu_circRNA_43867	chr9	+	22638729	22670957	exonic	-3.117	8.76555	-9.9	1.77E-07	0.001828	Bbs9
MCAO 5m v.s. SHAM	mmu_circRNA_43955	chr9	+	32732948	32738359	exonic	2.168	9.77374	3.728	0.00246539	0.04696	Ets1
MCAO 5m v.s. SHAM	mmu_circRNA_44092	chr9	+	46123216	46155540	exonic	-2.062	7.47221	-6.4	2.19E-05	0.011873	Sik3
MCAO 5m v.s. SHAM	mmu_circRNA_44440	chr9	-	64725501	64728429	exonic	-2.274	10.1907	-5.87	5.16E-05	0.014544	Rab11a
MCAO 5m v.s. SHAM	mmu_circRNA_44528	chr9	-	66540061	66547955	exonic	-2.286	7.871	-4.3	0.00083796	0.03302	Usp3
MCAO 5m v.s. SHAM	mmu_circRNA_44742	chr9	+	83569568	83577621	sense overlapping	-2.163	8.28082	-4.84	0.00030849	0.022856	Sh3bgrl2
MCAO 5m v.s. SHAM	mmu_circRNA_44743	chr9	+	83577435	83597526	sense overlapping	-2.424	8.28884	-5.1	0.00019353	0.019478	Sh3bgrl2
MCAO 5m v.s. SHAM	mmu_circRNA_44966	chr9	+	101100570	101124015	sense overlapping	-2.112	10.7835	-4.74	0.00036816	0.024632	Ppp2r3a
MCAO 5m v.s. SHAM	mmu_circRNA_45392	chrX	+	20376880	20377546	exonic	-2.662	8.78223	-4.77	0.00035269	0.023955	Rp2
MCAO 5m v.s. SHAM	mmu_circRNA_45393	chrX	+	20376880	20381819	exonic	-2.285	8.85704	-3.96	0.0015981	0.040143	Rp2
MCAO 5m v.s. SHAM	mmu_circRNA_45921	chrX	+	160523271	160533077	exonic	-3.157	12.4019	-6.06	3.78E-05	0.014378	Phka2
MCAO 3h v.s. SHAM	mmu_circRNA_39116	chr5	+	107490662	107496053	sense overlapping	2.317	9.44385	5.077	0.00020188	0.010743	NA
MCAO 3h v.s. SHAM	mmu_circRNA_30518	chr17	+	45562001	45562400	sense overlapping	2.695	10.6643	4.733	0.00037458	0.015797	Nfkbie
MCAO 3h v.s. SHAM	mmu_circRNA_19217	chr19	-	4288591	4294928	sense overlapping	3.17	11.0486	5.515	9.40E-05	0.007856	Grk2
MCAO 3h v.s. SHAM	mmu_circRNA_23522	chr11	-	65053098	65067156	exonic	2.042	7.0033	5.633	7.69E-05	0.007353	Arhgap44
MCAO 3h v.s. SHAM	mmu_circRNA_19178	chr17	-	14988387	15044490	sense overlapping	3.786	13.1034	6.543	1.73E-05	0.005435	Ermard
MCAO 3h v.s. SHAM	mmu_circRNA_19178	chr17	-	14988387	15044490	sense overlapping	3.786	13.1034	6.543	1.73E-05	0.005435	Gm35343

MCAO 3h v.s. SHAM	mmu_circRNA_43833	chr9	+	21923439	21923863	antisense	2.413	8.15057	5.399	0.00011485	0.008099	Tmem205
MCAO 3h v.s. SHAM	mmu_circRNA_27242	chr14	-	26422420	26470400	exonic	2.212	8.65704	4.677	0.0004147	0.0164	Slmap
MCAO 3h v.s. SHAM	mmu_circRNA_36726	chr4	+	45992196	46018616	exonic	2.712	10.2689	6.142	3.30E-05	0.005435	Tdrd7
MCAO 3h v.s. SHAM	mmu_circRNA_31307	chr18	+	11905924	11926364	exonic	2.383	10.0866	6.489	1.89E-05	0.005435	Cables1
MCAO 3h v.s. SHAM	mmu_circRNA_37769	chr4	-	134148009	134156371	exonic	2.631	10.1983	6.091	3.58E-05	0.005435	Cep85
MCAO 3h v.s. SHAM	mmu_circRNA_40916	chr6	+	124703226	124703627	exonic	2.149	7.83643	6.295	2.57E-05	0.005435	Lpcat3
MCAO 3h v.s. SHAM	mmu_circRNA_37267	chr4	-	103048209	103096775	exonic	2.41	10.0703	4.92	0.00026703	0.013004	Wdr78
MCAO 3h v.s. SHAM	mmu_circRNA_41362	chr7	+	46427345	46428279	exonic	2.282	10.3476	5.805	5.75E-05	0.006637	Kcnc1
MCAO 3h v.s. SHAM	mmu_circRNA_39875	chr6	-	18378366	18423981	exonic	2.081	9.81301	4.102	0.00120953	0.032948	Cttnbp2
MCAO 3h v.s. SHAM	mmu_circRNA_38639	chr5	-	62466745	62467049	intergenic	2.015	5.82599	5.11	0.00019049	0.010406	NA
MCAO 3h v.s. SHAM	mmu_circRNA_31230	chr18	-	7265000	7285792	exonic	2.275	8.901	6.662	1.44E-05	0.005435	Armc4
MCAO 3h v.s. SHAM	mmu_circRNA_43900	chr9	+	25594560	25634351	sense overlapping	2.466	7.75305	4.958	0.00024964	0.012332	Eepd1
MCAO 3h v.s. SHAM	mmu_circRNA_45876	chrX	-	157888397	157945416	exonic	4.214	9.30168	6.374	2.27E-05	0.005435	Cnksr2
MCAO 3h v.s. SHAM	mmu_circRNA_010498	chr2	-	143938457	143938574	antisense	3.316	12.0558	5.712	6.72E-05	0.006805	Dstn
MCAO 3h v.s. SHAM	mmu_circRNA_32218	chr19	-	7456299	7467867	exonic	3.004	11.3789	6.071	3.70E-05	0.005435	Rtn3
MCAO 3h v.s. SHAM	mmu_circRNA_26394	chr13	-	57512691	57513356	sense overlapping	2.511	11.9974	6.675	1.41E-05	0.005435	Spock1
MCAO 3h v.s. SHAM	mmu_circRNA_38328	chr5	+	28331110	28352643	exonic	-2.263	7.20658	-5.89	4.96E-05	0.006294	Rbm33
MCAO 3h v.s. SHAM	mmu_circRNA_45000	chr9	+	103160819	103167192	sense overlapping	2.465	7.88522	7.845	2.50E-06	0.005435	Rab6b
MCAO 3h v.s. SHAM	mmu_circRNA_30190	chr17	+	6016265	6019478	exonic	2.902	9.77447	6.367	2.29E-05	0.005435	Synj2
MCAO 3h v.s. SHAM	mmu_circRNA_40075	chr6	+	35189854	35224217	exonic	2.033	10.6563	4.353	0.00075476	0.024124	Nup205
MCAO 3h v.s. SHAM	mmu_circRNA_43988	chr9	-	36923583	36987272	exonic	2.079	11.5488	3.834	0.00201362	0.044707	Pknox2

MCAO 3h v.s. SHAM	mmu_circRNA_37766	chr4	-	133535509	133541216	exonic	2.575	8.53074	4.211	0.00098522	0.028571	Nudc
MCAO 3h v.s. SHAM	mmu_circRNA_30189	chr17	+	5990135	5996961	exonic	2.599	6.80978	7.079	7.61E-06	0.005435	Synj2
MCAO 3h v.s. SHAM	mmu_circRNA_36522	chr4	-	20469439	20505935	sense overlapping	2.306	9.03996	5.252	0.00014834	0.009453	Nkain3
MCAO 3h v.s. SHAM	mmu_circRNA_30666	chr17	-	57218564	57218671	intronic	2.079	10.6724	7.151	6.83E-06	0.005435	C3
MCAO 3h v.s. SHAM	mmu_circRNA_35606	chr3	-	75298105	75339532	intergenic	2.682	10.5375	6.388	2.22E-05	0.005435	Wdr49
MCAO 3h v.s. SHAM	mmu_circRNA_25821	chr12	-	108862802	108867777	exonic	2.003	7.83539	4.905	0.00027458	0.013309	Wars
MCAO 3h v.s. SHAM	mmu_circRNA_44586	chr9	-	71515068	71555737	exonic	2.881	8.69819	4.213	0.0009802	0.028571	Myzap
MCAO 3h v.s. SHAM	mmu_circRNA_22930	chr11	-	20306465	20320234	exonic	2.747	12.5212	6.222	2.89E-05	0.005435	Slc1a4
MCAO 3h v.s. SHAM	mmu_circRNA_26594	chr13	+	77245200	77281152	exonic	2.084	9.87178	4.689	0.000406	0.016328	2210408I21Rik
MCAO 3h v.s. SHAM	mmu_circRNA_27197	chr14	+	21774962	21780181	exonic	4.146	12.5543	6.206	2.97E-05	0.005435	Samd8
MCAO 3h v.s. SHAM	mmu_circRNA_22817	chr11	-	3711693	3712053	exonic	2.445	8.43872	4.394	0.00069939	0.022706	Osbp2
MCAO 3h v.s. SHAM	mmu_circRNA_45523	chrX	-	74035347	74080032	sense overlapping	2.068	8.21785	4.277	0.00086943	0.026374	Mecp2
MCAO 3h v.s. SHAM	mmu_circRNA_21880	chr10	-	41922121	41951746	exonic	2.609	10.4083	6.666	1.43E-05	0.005435	Armc2
MCAO 3h v.s. SHAM	mmu_circRNA_45149	chr9	+	110571946	110604231	exonic	2.457	8.1666	4.315	0.0008104	0.025171	Setd2
MCAO 3h v.s. SHAM	mmu_circRNA_41166	chr7	-	4430393	4435098	exonic	2.388	11.0901	6.679	1.40E-05	0.005435	Rdh13
MCAO 3h v.s. SHAM	mmu_circRNA_31317	chr18	+	12684836	12698059	exonic	2.25	10.9156	5.135	0.00018232	0.010214	Ttc39c
MCAO 3h v.s. SHAM	mmu_circRNA_36481	chr4	+	11593620	11605005	exonic	4.267	12.1502	6.207	2.96E-05	0.005435	Rad54b
MCAO 3h v.s. SHAM	mmu_circRNA_24344	chr11	+	107485549	107489072	exonic	4.168	9.89977	6.77	1.22E-05	0.005435	Psmd12
MCAO 3h v.s. SHAM	mmu_circRNA_38760	chr5	-	72329611	72343117	exonic	-2.069	7.15788	-5.77	6.14E-05	0.006637	Corin
MCAO 3h v.s. SHAM	mmu_circRNA_21120	chr1	+	159861209	159868285	exonic	2.239	9.45099	4.798	0.00033288	0.01475	Tnr

MCAO 3h v.s. SHAM	mmu_circRNA_43201	chr8	-	84707659	84713857	sense overlapping	2.057	7.28274	6.205	2.97E-05	0.005435	Nfix
MCAO 3h v.s. SHAM	mmu_circRNA_38590	chr5	-	51472597	51474411	exonic	2.341	11.8476	6.042	3.88E-05	0.005483	Ppargc1a
MCAO 3h v.s. SHAM	mmu_circRNA_29760	chr16	+	44355439	44377022	exonic	3.73	11.9177	6.856	1.07E-05	0.005435	Spice1
MCAO 3h v.s. SHAM	mmu_circRNA_40611	chr6	-	99162838	99669567	sense overlapping	2.519	11.8573	5.889	5.00E-05	0.006294	NA
MCAO 3h v.s. SHAM	mmu_circRNA_43263	chr8	-	86724205	86725856	sense overlapping	2.426	13.3906	5.784	5.96E-05	0.006637	Siah1a
MCAO 3h v.s. SHAM	mmu_circRNA_30884	chr17	-	73906009	73907745	exonic	3.334	9.06227	5.332	0.00012889	0.008697	Xdh
MCAO 3h v.s. SHAM	mmu_circRNA_35962	chr3	-	107445429	107448440	exonic	2.967	12.5675	6.647	1.47E-05	0.005435	Kcnc4
MCAO 3h v.s. SHAM	mmu_circRNA_26316	chr13	+	49820612	49830616	intergenic	-2.096	8.79094	-3.82	0.00207082	0.045383	LOC102631805
MCAO 3h v.s. SHAM	mmu_circRNA_23881	chr11	+	83321938	83336861	exonic	2.964	11.8823	6.502	1.85E-05	0.005435	Ap2b1
MCAO 3h v.s. SHAM	mmu_circRNA_010147	chr19	-	37068172	37088587	exonic	2.864	11.8228	6.234	2.84E-05	0.005435	Cpeb3
MCAO 3h v.s. SHAM	mmu_circRNA_44088	chr9	-	45943050	45944486	exonic	3.094	11.2441	6.581	1.63E-05	0.005435	Sidt2
MCAO 3h v.s. SHAM	mmu_circRNA_22088	chr10	-	62274446	62286652	exonic	2.263	9.9047	4.377	0.00072147	0.023132	Hk1
MCAO 3h v.s. SHAM	mmu_circRNA_42273	chr7	+	122754505	122762323	exonic	3.032	9.65921	6.056	3.79E-05	0.005435	Cacng3
MCAO 3h v.s. SHAM	mmu_circRNA_42362	chr7	+	129629917	129632421	exonic	3.133	10.6303	4.878	0.00028844	0.013682	Wdr11
MCAO 3h v.s. SHAM	mmu_circRNA_26137	chr13	-	24036184	24094570	exonic	2.25	9.85198	5.552	8.82E-05	0.007856	Carmil1
MCAO 3h v.s. SHAM	mmu_circRNA_41406	chr7	+	49780308	49829030	exonic	3.031	9.0492	6.457	1.99E-05	0.005435	Prmt3
MCAO 3h v.s. SHAM	mmu_circRNA_29484	chr16	-	30545125	30547469	exonic	2.236	10.6491	4.275	0.00087275	0.026374	Tmem44
MCAO 3h v.s. SHAM	mmu_circRNA_40590	chr6	-	94654846	94663998	exonic	3.286	12.122	6.991	8.69E-06	0.005435	Lrig1
MCAO 3h v.s. SHAM	mmu_circRNA_42581	chr8	+	14305203	14314292	intronic	2.204	7.54848	5.423	0.00011005	0.008021	Dlgap2
MCAO 3h v.s. SHAM	mmu_circRNA_27287	chr14	+	27449945	27459851	exonic	3.562	12.2679	6.218	2.91E-05	0.005435	Fam208a
MCAO 3h v.s. SHAM	mmu_circRNA_27821	chr14	+	77268806	77441899	exonic	3.51	10.955	6.304	2.54E-05	0.005435	Enox1

MCAO 3h v.s. SHAM	mmu_circRNA_31671	chr18	+	42288107	42305814	exonic	2.354	10.5446	5.493	9.76E-05	0.007856	Rbm27
MCAO 3h v.s. SHAM	mmu_circRNA_30586	chr17	+	50711410	50718783	intergenic	3.708	10.1033	5.26	0.00014624	0.009378	NA
MCAO 3h v.s. SHAM	mmu_circRNA_27546	chr14	-	51905612	51905724	sense overlapping	3.088	8.02883	4.747	0.00036527	0.015521	Ndrg2
MCAO 3h v.s. SHAM	mmu_circRNA_38545	chr5	+	43709043	43714601	exonic	2.365	10.5431	5.821	5.60E-05	0.006637	Cc2d2a
MCAO 3h v.s. SHAM	mmu_circRNA_22663	chr10	-	116343842	116346937	antisense	2.987	9.56817	5.756	6.25E-05	0.006637	Ptprb
MCAO 3h v.s. SHAM	mmu_circRNA_27893	chr14	+	79596747	79597466	exonic	2.456	10.324	5.523	9.28E-05	0.007856	Sugt1
MCAO 3h v.s. SHAM	mmu_circRNA_27264	chr14	+	26900422	26934532	sense overlapping	2.789	7.76428	5.308	0.00013438	0.008887	Appl1
MCAO 3h v.s. SHAM	mmu_circRNA_27026	chr14	+	8028236	8039904	exonic	2.74	8.79435	4.66	0.00042802	0.016607	Abhd6
MCAO 3h v.s. SHAM	mmu_circRNA_19110	chr14	-	19904158	19904277	intronic	3.125	11.1538	6.453	2.00E-05	0.005435	Gng2
MCAO 3h v.s. SHAM	mmu_circRNA_28026	chr14	+	118153568	118160184	exonic	2.356	8.81652	5.198	0.00016307	0.00972	Gpr180
MCAO 3h v.s. SHAM	mmu_circRNA_22790	chr10	+	128207297	128208667	exonic	2.709	7.95375	4.289	0.00085048	0.025977	Gls2
MCAO 3h v.s. SHAM	mmu_circRNA_41481	chr7	-	59284025	59317973	sense overlapping	2.13	9.50828	5.718	6.65E-05	0.006802	Snhg14
MCAO 3h v.s. SHAM	mmu_circRNA_002246	chr7	-	142651460	142651527	sense overlapping	-3.042	7.52559	-4.57	0.00050509	0.018169	Igf2
MCAO 3h v.s. SHAM	mmu_circRNA_19314	chr4	-	3080977	3084320	intergenic	2.804	10.6166	5.923	4.72E-05	0.006166	NA
MCAO 3h v.s. SHAM	mmu_circRNA_006741	chr6	-	119052600	119057515	exonic	2.013	8.357	5.728	6.55E-05	0.006759	Cacna1c
MCAO 3h v.s. SHAM	mmu_circRNA_22526	chr10	+	99402065	99402880	sense overlapping	2.957	8.75673	5.959	4.45E-05	0.005893	NA
MCAO 3h v.s. SHAM	mmu_circRNA_41238	chr7	-	27541084	27542181	exonic	2.132	10.0306	5.49	9.80E-05	0.007856	Pld3
MCAO 3h v.s. SHAM	mmu_circRNA_34860	chr2	+	156664798	156711261	exonic	2.289	9.01726	6.355	2.34E-05	0.005435	Dlgap4
MCAO 3h v.s. SHAM	mmu_circRNA_19015	chr11	+	53893644	53981397	sense overlapping	3.571	10.4419	5.749	6.31E-05	0.006637	Gm30927
MCAO 3h v.s. SHAM	mmu_circRNA_45137	chr9	-	110432433	110439058	exonic	2.081	10.1696	4.663	0.0004257	0.016585	Klhl18

MCAO 3h v.s. SHAM	mmu_circRNA_28632	chr15	+	62054793	62107465	intronic	2.169	10.8688	4.723	0.00038143	0.016008	Pvt1
MCAO 3h v.s. SHAM	mmu_circRNA_23261	chr11	-	46221335	46224205	exonic	3.01	10.9546	7.094	7.43E-06	0.005435	Cyfip2
MCAO 3h v.s. SHAM	mmu_circRNA_41378	chr7	-	46743993	46750514	intergenic	2.517	9.74998	6.251	2.76E-05	0.005435	NA
MCAO 3h v.s. SHAM	mmu_circRNA_39371	chr5	+	123934071	123936555	exonic	3.133	9.61083	5.123	0.00018608	0.010273	Ccdc62
MCAO 3h v.s. SHAM	mmu_circRNA_37770	chr4	-	134148009	134172880	exonic	2.706	9.51011	4.671	0.00041955	0.016407	Cep85
MCAO 3h v.s. SHAM	mmu_circRNA_20054	chr1	-	52189422	52207359	exonic	2.018	7.59221	4.572	0.0005031	0.018161	Gls
MCAO 3h v.s. SHAM	mmu_circRNA_001874	chr9	+	120453526	120464961	exonic	2.474	9.54261	5.011	0.00022694	0.011656	Myrip
MCAO 3h v.s. SHAM	mmu_circRNA_013002	chr16	+	94403313	94468060	sense overlapping	2.35	10.6294	5.078	0.00020148	0.010743	Ttc3
MCAO 3h v.s. SHAM	mmu_circRNA_40913	chr6	+	122828156	122833876	exonic	2.033	12.9587	6.281	2.63E-05	0.005435	Foxj2
MCAO 3h v.s. SHAM	mmu_circRNA_38234	chr5	-	23384145	23385172	exonic	2.001	12.1188	4.975	0.00024214	0.012135	5031425E22Rik
MCAO 3h v.s. SHAM	mmu_circRNA_38234	chr5	-	23384145	23385172	exonic	2.001	12.1188	4.975	0.00024214	0.012135	LOC102635844
MCAO 3h v.s. SHAM	mmu_circRNA_003170	chr2	+	18101457	18126229	exonic	2.621	11.6332	5.392	0.00011611	0.008099	Mllt10
MCAO 3h v.s. SHAM	mmu_circRNA_31773	chr18	+	56728400	56733337	exonic	2.115	12.9694	4.861	0.00029715	0.013925	Lmnb1
MCAO 3h v.s. SHAM	mmu_circRNA_002314	chr13	+	8672404	8731971	exonic	2.828	8.09707	4.647	0.00043864	0.016772	Adarb2
MCAO 3h v.s. SHAM	mmu_circRNA_29156	chr16	+	5234965	5239166	exonic	2.654	11.4155	6.114	3.45E-05	0.005435	Alg1
MCAO 3h v.s. SHAM	mmu_circRNA_015541	chr2	+	37508927	37541176	exonic	3.498	9.68183	5.052	0.00021104	0.011047	Strbp
MCAO 3h v.s. SHAM	mmu_circRNA_015541	chr2	+	37508927	37541176	exonic	3.498	9.68183	5.052	0.00021104	0.011047	Rabgap1
MCAO 3h v.s. SHAM	mmu_circRNA_38761	chr5	-	72353869	72360758	exonic	2.827	9.91956	5.335	0.00012832	0.008697	Corin
MCAO 3h v.s. SHAM	mmu_circRNA_28834	chr15	-	81323609	81338087	exonic	2.099	13.0963	4.245	0.00092305	0.027463	Slc25a17
MCAO 3h v.s. SHAM	mmu_circRNA_013526	chr17	-	90560740	90597629	exonic	2.374	7.42759	3.843	0.00198022	0.044155	Nrxn1
MCAO 3h v.s. SHAM	mmu_circRNA_011301	chr14	+	13995006	14013655	sense overlapping	2.227	10.4371	4.332	0.00078497	0.024707	NA

MCAO 3h v.s. SHAM	mmu_circRNA_27565	chr14	-	55640384	55641588	exonic	3.925	10.9835	5.961	4.44E-05	0.005893	Tm9sf1
MCAO 3h v.s. SHAM	mmu_circRNA_27592	chr14	+	56887794	56919342	exonic	2.873	7.43915	4.828	0.00031522	0.014336	Zmym2
MCAO 3h v.s. SHAM	mmu_circRNA_28652	chr15	-	64783723	64815418	exonic	2.47	8.14097	4.454	0.00062551	0.021454	Adcy8
MCAO 3h v.s. SHAM	mmu_circRNA_25585	chr12	+	88850278	88853112	intronic	2.169	12.3907	4.751	0.0003624	0.015521	Nrxn3
MCAO 3h v.s. SHAM	mmu_circRNA_002563	chr18	+	22375132	22434675	exonic	2.604	11.7048	5.393	0.00011593	0.008099	Asxl3
MCAO 3h v.s. SHAM	mmu_circRNA_24567	chr12	+	3899192	3900361	exonic	2.307	12.9486	5.598	8.15E-05	0.007579	Dnmt3a
MCAO 3h v.s. SHAM	mmu_circRNA_30337	chr17	-	25380224	25381573	exonic	2.333	9.03534	5.807	5.73E-05	0.006637	Cacna1h
MCAO 3h v.s. SHAM	mmu_circRNA_27181	chr14	-	21476670	21483639	antisense	2.216	12.1141	4.688	0.00040646	0.016328	Gm30363
MCAO 3h v.s. SHAM	mmu_circRNA_27181	chr14	-	21476670	21483639	antisense	2.216	12.1141	4.688	0.00040646	0.016328	Kat6b
MCAO 3h v.s. SHAM	mmu_circRNA_24789	chr12	+	21230822	21247373	exonic	2.546	10.8049	5.826	5.55E-05	0.006637	Asap2
MCAO 3h v.s. SHAM	mmu_circRNA_24789	chr12	+	21230822	21247373	exonic	2.546	10.8049	5.826	5.55E-05	0.006637	Gm40855
MCAO 3h v.s. SHAM	mmu_circRNA_42048	chr7	+	105682778	105687385	exonic	2.27	12.6057	6.099	3.53E-05	0.005435	Dnhd1
MCAO 3h v.s. SHAM	mmu_circRNA_34777	chr2	+	152677541	152704941	sense overlapping	2.375	13.5676	5.65	7.47E-05	0.007353	H13
MCAO 3h v.s. SHAM	mmu_circRNA_008457	chr16	-	89877037	89885125	exonic	2.057	12.2258	5.749	6.31E-05	0.006637	Tiam1
MCAO 3h v.s. SHAM	mmu_circRNA_35586	chr3	+	73269612	73341175	intronic	2.356	8.29045	4.971	0.00024367	0.012153	Gm20754
MCAO 3h v.s. SHAM	mmu_circRNA_42423	chr7	+	133650500	133667691	exonic	3.089	11.0595	5.559	8.72E-05	0.007856	Edrf1
MCAO 3h v.s. SHAM	mmu_circRNA_40203	chr6	+	40004144	40207533	sense overlapping	4.494	10.0608	7.275	5.69E-06	0.005435	Tmem178b
MCAO 3h v.s. SHAM	mmu_circRNA_43956	chr9	+	34536915	34538449	intronic	3.887	9.26161	6.78	1.20E-05	0.005435	Kirrel3
MCAO 3h v.s. SHAM	mmu_circRNA_33499	chr2	-	48087107	48095756	sense overlapping	2.662	9.1881	3.899	0.00177806	0.041468	Gm13481
MCAO 3h v.s. SHAM	mmu_circRNA_34968	chr2	-	166617832	166621333	exonic	3.655	11.8013	6.226	2.88E-05	0.005435	Prex1

MCAO 3h v.s. SHAM	mmu_circRNA_41052	chr6	-	144155096	144625686	exonic	2.313	10.6932	6.157	3.22E-05	0.005435	NA
MCAO 3h v.s. SHAM	mmu_circRNA_19470	chr7	-	104315409	104348404	sense overlapping	2.778	12.3217	5.572	8.52E-05	0.007786	Trim5
MCAO 3h v.s. SHAM	mmu_circRNA_36544	chr4	+	22025119	22040595	intergenic	2.523	7.03666	5.062	0.00020722	0.010915	Gm42260
MCAO 3h v.s. SHAM	mmu_circRNA_015492	chr4	+	101612861	101616865	exonic	2.227	12.8653	5.325	0.00013058	0.008754	Dnajc6
MCAO 3h v.s. SHAM	mmu_circRNA_39830	chr6	-	8643972	8658314	exonic	2.185	7.55356	5.838	5.44E-05	0.006637	Ica1
MCAO 3h v.s. SHAM	mmu_circRNA_32047	chr18	-	80808611	80891923	exonic	2.483	12.7347	6.094	3.56E-05	0.005435	Atp9b
MCAO 3h v.s. SHAM	mmu_circRNA_28307	chr15	-	25027225	25364918	sense overlapping	2.367	10.2107	3.933	0.0016677	0.039907	Gm2824
MCAO 3h v.s. SHAM	mmu_circRNA_43907	chr9	+	28039050	28045364	intronic	2.841	11.0118	6.092	3.57E-05	0.005435	Opcml
MCAO 3h v.s. SHAM	mmu_circRNA_37176	chr4	+	95696959	95769529	exonic	2.594	10.5223	4.713	0.00038878	0.01606	Fggy
MCAO 3h v.s. SHAM	mmu_circRNA_45837	chrX	+	152033698	152047968	exonic	2.703	10.4481	7.657	3.26E-06	0.005435	Smc1a
MCAO 3h v.s. SHAM	mmu_circRNA_26486	chr13	+	63156545	63240331	exonic	2.682	8.743	3.812	0.00209816	0.045606	2010111I01Rik
MCAO 3h v.s. SHAM	mmu_circRNA_38907	chr5	+	86891227	86905024	antisense	2.044	13.7169	6.995	8.64E-06	0.005435	Ugt2b34
MCAO 3h v.s. SHAM	mmu_circRNA_29553	chr16	+	32884123	32898470	exonic	2.27	7.95559	4.089	0.00124055	0.033353	Fyttd1
MCAO 3h v.s. SHAM	mmu_circRNA_38525	chr5	+	37278084	37278931	exonic	2.843	8.0808	5.302	0.00013585	0.008887	Crmp1
MCAO 3h v.s. SHAM	mmu_circRNA_42572	chr8	-	13605861	13631892	exonic	3.42	11.1948	5.764	6.16E-05	0.006637	Rasa3
MCAO 3h v.s. SHAM	mmu_circRNA_44389	chr9	-	61924631	61926518	exonic	2.417	11.5111	6.057	3.79E-05	0.005435	Kif23
MCAO 3h v.s. SHAM	mmu_circRNA_22421	chr10	+	90510595	90644556	exonic	2.955	8.96316	4.236	0.00093972	0.027719	Anks1b
MCAO 3h v.s. SHAM	mmu_circRNA_23643	chr11	-	72456302	72474770	exonic	-2.416	8.98581	-3.79	0.00219011	0.046813	Spns2
MCAO 3h v.s. SHAM	mmu_circRNA_001832	chr6	-	82730767	82739674	exonic	3.423	12.3907	7.498	4.10E-06	0.005435	Hk2
MCAO 3h v.s. SHAM	mmu_circRNA_37286	chr4	+	103754180	103764967	intronic	3.067	8.22307	4.234	0.00094287	0.027733	Dab1
MCAO 3h v.s. SHAM	mmu_circRNA_32308	chr19	+	21797326	21818011	exonic	2.384	10.4919	7.236	6.02E-06	0.005435	Tmem2

MCAO 3h v.s. SHAM	mmu_circRNA_20259	chr1	-	64034531	64042437	sense overlapping	2.475	10.5876	5.53	9.16E-05	0.007856	Klf7
MCAO 3h v.s. SHAM	mmu_circRNA_003057	chr18	+	11923359	11926364	exonic	2.269	9.66942	6.78	1.20E-05	0.005435	Cables1
MCAO 3h v.s. SHAM	mmu_circRNA_45921	chrX	+	160523271	160533077	exonic	-2.827	12.4019	-5.42	0.00010991	0.008021	Phka2
MCAO 3h v.s. SHAM	mmu_circRNA_20456	chr1	+	86086029	86090190	exonic	2.759	9.70433	7.33	5.24E-06	0.005435	Psmd1
MCAO 3h v.s. SHAM	mmu_circRNA_22970	chr11	-	22137837	22151892	exonic	3.338	9.42401	4.296	0.00083893	0.025701	Ehbp1
MCAO 3h v.s. SHAM	mmu_circRNA_36483	chr4	-	12057315	12063830	exonic	2.8	13.9213	5.744	6.36E-05	0.006637	Tmem67
MCAO 3h v.s. SHAM	mmu_circRNA_36632	chr4	+	34647785	34656262	exonic	2.305	9.40609	4.016	0.00142342	0.036018	Rars2
MCAO 3h v.s. SHAM	mmu_circRNA_37699	chr4	-	129712103	129730325	exonic	-3.028	12.3654	-3.86	0.00191273	0.042835	Khdrbs1
MCAO 3h v.s. SHAM	mmu_circRNA_010777	chr14	-	23494538	23509900	exonic	4.007	9.39607	7.527	3.93E-06	0.005435	Kcnma1
MCAO 3h v.s. SHAM	mmu_circRNA_41390	chr7	+	48799828	48818720	exonic	2.362	11.2406	7.006	8.49E-06	0.005435	Zdhhc13
MCAO 3h v.s. SHAM	mmu_circRNA_33862	chr2	-	73373614	73374014	exonic	2.473	9.08106	3.855	0.00193565	0.043255	Gm13709
MCAO 3h v.s. SHAM	mmu_circRNA_33862	chr2	-	73373614	73374014	exonic	2.473	9.08106	3.855	0.00193565	0.043255	Gpr155
MCAO 3h v.s. SHAM	mmu_circRNA_43955	chr9	+	32732948	32738359	exonic	2.488	9.77374	4.279	0.00086687	0.026374	Ets1
MCAO 3h v.s. SHAM	mmu_circRNA_010789	chr15	-	96609855	96624169	exonic	2.145	11.0649	5.844	5.38E-05	0.006637	Slc38a1
MCAO 3h v.s. SHAM	mmu_circRNA_017685	chr16	+	5953418	5956863	intronic	2.279	11.6724	5.235	0.00015274	0.009499	Rbfox1
MCAO 3h v.s. SHAM	mmu_circRNA_29001	chr15	-	95229083	95241677	exonic	2.107	10.047	4.148	0.00110784	0.030705	Nell2
MCAO 3h v.s. SHAM	mmu_circRNA_43010	chr8	-	70405258	70409011	exonic	2.208	10.4832	6.163	3.19E-05	0.005435	Crtc1
MCAO 3h v.s. SHAM	mmu_circRNA_30482	chr17	+	34416466	34421987	exonic	2.124	12.0496	6.618	1.54E-05	0.005435	BC051142
MCAO 3h v.s. SHAM	mmu_circRNA_37006	chr4	-	83260881	83271218	exonic	2.254	9.54519	4.841	0.00030788	0.014082	Ttc39b
MCAO 3h v.s. SHAM	mmu_circRNA_38874	chr5	-	84067359	84156536	exonic	2.374	10.7494	5.639	7.61E-05	0.007353	Epha5

MCAO 3h v.s. SHAM	mmu_circRNA_44220	chr9	+	55397740	55400647	exonic	2.292	9.17902	5.444	0.00010616	0.007896	Tmem266
MCAO 3h v.s. SHAM	mmu_circRNA_014406	chr15	+	85359453	85365359	exonic	2.397	9.10438	4.414	0.00067355	0.022472	Atxn10
MCAO 3h v.s. SHAM	mmu_circRNA_21750	chr10	+	21928341	21928836	sense overlapping	2.718	12.9902	6.269	2.68E-05	0.005435	Sgk1
MCAO 3h v.s. SHAM	mmu_circRNA_19780	chr1	+	24383303	24390303	antisense	2.573	6.42785	8.316	1.31E-06	0.005435	Col19a1
MCAO 3h v.s. SHAM	mmu_circRNA_41537	chr7	-	61813261	61916736	exonic	2.045	10.3586	5.514	9.42E-05	0.007856	A230057D06Rik
MCAO 3h v.s. SHAM	mmu_circRNA_32634	chr19	+	43810470	43814814	exonic	2.339	6.89627	4.882	0.00028623	0.013681	Abcc2
MCAO 3h v.s. SHAM	mmu_circRNA_21730	chr10	+	20158760	20159555	intronic	3.373	12.6282	6.154	3.23E-05	0.005435	Map7
MCAO 3h v.s. SHAM	mmu_circRNA_42246	chr7	+	121032747	121057399	sense overlapping	3.265	11.8786	6.121	3.41E-05	0.005435	NA
MCAO 3h v.s. SHAM	mmu_circRNA_001686	chr6	+	61310813	61313835	exonic	2.024	13.6931	6.071	3.70E-05	0.005435	Ccser1
MCAO 3h v.s. SHAM	mmu_circRNA_21755	chr10	-	23138190	23159154	exonic	2.306	13.2622	5.607	8.04E-05	0.007543	Eya4
MCAO 3h v.s. SHAM	mmu_circRNA_19073	chr12	+	81894979	81913476	sense overlapping	2.317	8.00463	5.05	0.00021187	0.011047	Pcnx
MCAO 3h v.s. SHAM	mmu_circRNA_45629	chrX	-	98695998	98714211	exonic	2.776	10.5693	5.591	8.25E-05	0.007607	Ophn1
MCAO 3h v.s. SHAM	mmu_circRNA_20164	chr1	-	59066149	59076722	exonic	2.222	8.62554	5.443	0.00010632	0.007896	Als2cr11
MCAO 3h v.s. SHAM	mmu_circRNA_37285	chr4	+	103754180	103755895	intronic	2.598	9.65288	4.85	0.00030326	0.013977	Dab1
MCAO 3h v.s. SHAM	mmu_circRNA_23477	chr11	+	62283596	62285885	exonic	2.613	9.10737	3.997	0.00147425	0.036442	Ttc19
MCAO 3h v.s. SHAM	mmu_circRNA_22246	chr10	+	75966350	75986140	exonic	3.102	10.6644	5.967	4.39E-05	0.005893	Gm5134
MCAO 3h v.s. SHAM	mmu_circRNA_43344	chr8	-	94086655	94092622	exonic	-2.523	6.19788	-3.91	0.00173021	0.041064	Bbs2
MCAO 3h v.s. SHAM	mmu_circRNA_27734	chr14	-	66187442	66190234	exonic	2.101	9.4989	5.823	5.58E-05	0.006637	Ptk2b
MCAO 3h v.s. SHAM	mmu_circRNA_30556	chr17	-	46882732	46883949	sense overlapping	2.368	9.65751	5.909	4.83E-05	0.006234	NA
MCAO 3h v.s. SHAM	mmu_circRNA_36399	chr4	+	3589146	3598663	exonic	2.614	10.4637	4.01	0.00144039	0.036119	Tgs1
MCAO 3h v.s. SHAM	mmu_circRNA_25951	chr13	+	8559103	8570574	exonic	2.88	9.2851	5.755	6.25E-05	0.006637	Adarb2

MCAO 3h v.s. SHAM	mmu_circRNA_33227	chr2	-	29247827	29248878	sense overlapping	2.966	11.6832	5.482	9.95E-05	0.007856	6530402F18Rik
MCAO 3h v.s. SHAM	mmu_circRNA_27500	chr14	-	40907705	40934285	sense overlapping	2.246	10.731	5.75	6.31E-05	0.006637	Tspan14
MCAO 3h v.s. SHAM	mmu_circRNA_31359	chr18	-	16627567	16629712	exonic	2.196	11.9233	5.08	0.00020069	0.010743	Cdh2
MCAO 3h v.s. SHAM	mmu_circRNA_24243	chr11	+	104456946	104459039	antisense	2.151	11.0241	5.213	0.00015884	0.00959	NA
MCAO 3h v.s. SHAM	mmu_circRNA_26219	chr13	-	38934737	38944654	exonic	2.178	11.0949	4.321	0.00080111	0.024987	Slc35b3
MCAO 3h v.s. SHAM	mmu_circRNA_26526	chr13	-	68668469	68678615	exonic	2.927	12.1565	6.383	2.23E-05	0.005435	Adcy2
MCAO 3h v.s. SHAM	mmu_circRNA_32900	chr2	-	5914809	5923023	sense overlapping	2.853	7.94835	5.215	0.00015813	0.00959	Dhtkd1
MCAO 3h v.s. SHAM	mmu_circRNA_33228	chr2	+	29679132	29689263	exonic	3.025	9.78871	4.721	0.00038299	0.016008	Rapgef1
MCAO 3h v.s. SHAM	mmu_circRNA_008226	chr18	+	22375132	22392852	exonic	2.782	10.9977	5.542	8.98E-05	0.007856	Asxl3
MCAO 3h v.s. SHAM	mmu_circRNA_25584	chr12	+	88431020	88459203	exonic	2.977	12.9812	5.393	0.00011606	0.008099	Adck1
MCAO 3h v.s. SHAM	mmu_circRNA_011505	chr15	+	79114654	79115171	exonic	3.572	10.6939	5.397	0.00011524	0.008099	Micall1
MCAO 3h v.s. SHAM	mmu_circRNA_19995	chr1	-	42660742	42667003	sense overlapping	2.478	7.71634	7.32	5.32E-06	0.005435	Pantr1
MCAO 3h v.s. SHAM	mmu_circRNA_42102	chr7	+	111046762	111050505	exonic	2.414	8.4655	6.611	1.56E-05	0.005435	Ctr9
MCAO 24h v.s. SHAM	mmu_circRNA_21485	chr1	-	182278975	182279718	exonic	-2.036	10.7546	-3.99	0.00150908	0.008777	Degs1
MCAO 24h v.s. SHAM	mmu_circRNA_29986	chr16	-	87420172	87420982	exonic	2.228	10.0205	4.755	0.0003599	0.003188	Ltn1
MCAO 24h v.s. SHAM	mmu_circRNA_39116	chr5	+	107490662	107496053	sense overlapping	4.447	9.44385	9.744	2.12E-07	4.58E-05	NA
MCAO 24h v.s. SHAM	mmu_circRNA_34867	chr2	-	157120481	157126844	exonic	-2.297	6.94402	-5.79	5.92E-05	0.000932	Samhd1
MCAO 24h v.s. SHAM	mmu_circRNA_30518	chr17	+	45562001	45562400	sense overlapping	3.297	10.6643	5.79	5.90E-05	0.000932	Nfkbie
MCAO 24h v.s. SHAM	mmu_circRNA_45522	chrX	-	74035347	74037332	sense overlapping	3.566	8.11784	7.047	7.98E-06	0.000259	Mecp2
MCAO 24h v.s. SHAM	mmu_circRNA_002888	chr12	-	51759218	51761170	exonic	-2.39	11.4477	-4.69	0.00040854	0.003472	Hectd1

MCAO 24h v.s. SHAM	mmu_circRNA_006013	chrX	+	69544408	69545269	exonic	2.427	5.81716	3.203	0.00680652	0.026233	Aff2
MCAO 24h v.s. SHAM	mmu_circRNA_008748	chr17	+	65810537	65816050	exonic	-2.013	7.82485	-6.53	1.78E-05	0.000423	Ppp4r1
MCAO 24h v.s. SHAM	mmu_circRNA_19531	chr9	-	107812210	107812329	intronic	-2.093	6.27046	-4.42	0.00066806	0.004942	Rbm6
MCAO 24h v.s. SHAM	mmu_circRNA_012164	chr13	+	23739382	23739448	sense overlapping	-2.196	9.8743	-4.83	0.00031411	0.002923	Hist1h1c
MCAO 24h v.s. SHAM	mmu_circRNA_31561	chr18	-	34614447	34614797	exonic	-2.86	10.1297	-5.38	0.00011872	0.001524	Brd8
MCAO 24h v.s. SHAM	mmu_circRNA_28074	chr14	+	121973608	121994470	exonic	-2.057	10.3144	-4.65	0.0004398	0.003659	Ubac2
MCAO 24h v.s. SHAM	mmu_circRNA_19217	chr19	-	4288591	4294928	sense overlapping	3.989	11.0486	6.94	9.38E-06	0.000279	Grk2
MCAO 24h v.s. SHAM	mmu_circRNA_21799	chr10	+	29196225	29199280	sense overlapping	2.937	11.2096	8.202	1.53E-06	9.37E-05	Soga3
MCAO 24h v.s. SHAM	mmu_circRNA_32778	chr19	+	55628787	55631286	intergenic	2.066	8.37006	6.008	4.10E-05	0.000734	NA
MCAO 24h v.s. SHAM	mmu_circRNA_35360	chr3	+	54701486	54710472	exonic	-2.824	9.29439	-7.79	2.71E-06	0.000137	Supt20
MCAO 24h v.s. SHAM	mmu_circRNA_27614	chr14	+	57472974	57496279	exonic	-4.131	6.91049	-5.52	9.27E-05	0.001288	Ift88
MCAO 24h v.s. SHAM	mmu_circRNA_008982	chr13	+	18810431	18830793	exonic	2.234	6.51841	5.477	0.00010037	0.001358	Vps41
MCAO 24h v.s. SHAM	mmu_circRNA_40001	chr6	+	30443988	30447477	sense overlapping	-3.22	9.14632	-3.58	0.00327517	0.015412	Klhdc10
MCAO 24h v.s. SHAM	mmu_circRNA_26717	chr13	+	93790038	93807703	exonic	3.332	8.31656	5.759	6.21E-05	0.000962	Arsb
MCAO 24h v.s. SHAM	mmu_circRNA_34233	chr2	-	112886491	112912403	exonic	3.461	8.23847	6.785	1.19E-05	0.000327	Ryr3
MCAO 24h v.s. SHAM	mmu_circRNA_23522	chr11	-	65053098	65067156	exonic	2.704	7.0033	7.46	4.34E-06	0.000173	Arhgap44
MCAO 24h v.s. SHAM	mmu_circRNA_19178	chr17	-	14988387	15044490	sense overlapping	4.424	13.1034	7.645	3.32E-06	0.00015	Ermard
MCAO 24h v.s. SHAM	mmu_circRNA_19178	chr17	-	14988387	15044490	sense overlapping	4.424	13.1034	7.645	3.32E-06	0.00015	Gm35343
MCAO 24h v.s. SHAM	mmu_circRNA_43833	chr9	+	21923439	21923863	antisense	2.649	8.15057	5.927	4.69E-05	0.000802	Tmem205
MCAO 24h v.s. SHAM	mmu_circRNA_27242	chr14	-	26422420	26470400	exonic	2.007	8.65704	4.244	0.00092637	0.006247	Slmap
MCAO 24h v.s. SHAM	mmu_circRNA_37644	chr4	+	127025854	127026227	exonic	-2.26	8.17618	-5.03	0.00022076	0.002297	Sfpq

MCAO 24h v.s. SHAM	mmu_circRNA_36102	chr3	-	122947360	122969249	exonic	-3.202	7.15776	-6.76	1.24E-05	0.000336	Usp53
MCAO 24h v.s. SHAM	mmu_circRNA_41233	chr7	-	27212311	27213061	exonic	2.268	8.76204	5	0.0002316	0.002358	Itpkc
MCAO 24h v.s. SHAM	mmu_circRNA_25332	chr12	+	73967346	73975161	sense overlapping	-2.035	9.99379	-2.91	0.01193313	0.039613	Snapc1
MCAO 24h v.s. SHAM	mmu_circRNA_26221	chr13	+	41008210	41009364	exonic	2.603	9.46183	7.883	2.37E-06	0.000125	Pak1ip1
MCAO 24h v.s. SHAM	mmu_circRNA_21913	chr10	+	43345527	43345974	sense overlapping	-2.043	8.49264	-3.82	0.00207403	0.011026	Pdss2
MCAO 24h v.s. SHAM	mmu_circRNA_013251	chr9	-	89601041	89603394	exonic	-2.338	6.91191	-6.13	3.34E-05	0.000637	AF529169
MCAO 24h v.s. SHAM	mmu_circRNA_37780	chr4	+	134934931	134935464	exonic	-2.273	10.3658	-4.82	0.00031977	0.002952	Syf2
MCAO 24h v.s. SHAM	mmu_circRNA_016017	chr9	+	64178285	64178345	sense overlapping	-3.4	11.05	-9.58	2.60E-07	4.97E-05	Rpl4
MCAO 24h v.s. SHAM	mmu_circRNA_007217	chr13	+	23739400	23739466	sense overlapping	-2.364	10.8611	-5.71	6.75E-05	0.001023	Hist1h1c
MCAO 24h v.s. SHAM	mmu_circRNA_31307	chr18	+	11905924	11926364	exonic	3.415	10.0866	9.299	3.65E-07	5.17E-05	Cables1
MCAO 24h v.s. SHAM	mmu_circRNA_21531	chr1	+	190216581	190218968	sense overlapping	3.411	7.02368	4.707	0.000393	0.00337	NA
MCAO 24h v.s. SHAM	mmu_circRNA_001121	chr6	+	17850356	17855028	exonic	-2.548	9.46733	-8.1	1.75E-06	0.000103	St7
MCAO 24h v.s. SHAM	mmu_circRNA_41718	chr7	+	76318079	76335324	exonic	2.131	10.3335	5.917	4.77E-05	0.000809	Agbl1
MCAO 24h v.s. SHAM	mmu_circRNA_29113	chr15	+	102473284	102478747	exonic	-2.048	7.54428	-5.27	0.00014271	0.001715	Pcbp2
MCAO 24h v.s. SHAM	mmu_circRNA_015638	chr19	-	4784723	4788042	intronic	-2.063	10.7685	-5.12	0.00018622	0.002026	Rbm4
MCAO 24h v.s. SHAM	mmu_circRNA_015638	chr19	-	4784723	4788042	intronic	-2.063	10.7685	-5.12	0.00018622	0.002026	Gm21992
MCAO 24h v.s. SHAM	mmu_circRNA_40180	chr6	+	38559169	38591431	sense overlapping	-2.752	9.14523	-5.96	4.45E-05	0.000776	Luc7l2
MCAO 24h v.s. SHAM	mmu_circRNA_37769	chr4	-	134148009	134156371	exonic	3.941	10.1983	9.124	4.55E-07	5.38E-05	Cep85
MCAO 24h v.s. SHAM	mmu_circRNA_29200	chr16	-	9653395	9663607	exonic	-2.02	7.11965	-3.43	0.00437341	0.019108	Grin2a
MCAO 24h v.s. SHAM	mmu_circRNA_016901	chr17	+	83221789	83222230	exonic	4.253	6.14526	5.814	5.66E-05	0.000906	Pkdcc

MCAO 24h v.s. SHAM	mmu_circRNA_40916	chr6	+	124703226	124703627	exonic	3.136	7.83643	9.188	4.20E-07	5.26E-05	Lpcat3
MCAO 24h v.s. SHAM	mmu_circRNA_010432	chr4	-	151830417	151835883	exonic	-2.997	12.5229	-11.3	3.56E-08	3.37E-05	Camta1
MCAO 24h v.s. SHAM	mmu_circRNA_30848	chr17	-	71273373	71275287	exonic	-2.497	9.01996	-8.57	9.33E-07	7.61E-05	Emilin2
MCAO 24h v.s. SHAM	mmu_circRNA_016773	chr17	+	35834970	35835050	antisense	2.392	14.0589	8.888	6.15E-07	6.28E-05	Tubb5
MCAO 24h v.s. SHAM	mmu_circRNA_36156	chr3	+	132694382	132752669	exonic	2.879	12.6197	10.27	1.15E-07	4.18E-05	Tbck
MCAO 24h v.s. SHAM	mmu_circRNA_41362	chr7	+	46427345	46428279	exonic	3.637	10.3476	9.252	3.88E-07	5.20E-05	Kcnc1
MCAO 24h v.s. SHAM	mmu_circRNA_009970	chr13	-	48590286	48606360	exonic	2.355	7.50204	3.988	0.00150118	0.008736	Ptpdc1
MCAO 24h v.s. SHAM	mmu_circRNA_23349	chr11	+	53397831	53402566	exonic	-2.91	7.25684	-5.82	5.57E-05	0.000898	Aff4
MCAO 24h v.s. SHAM	mmu_circRNA_32258	chr19	+	16176757	16219772	sense overlapping	3.347	7.14132	7.779	2.74E-06	0.000137	Gnaq
MCAO 24h v.s. SHAM	mmu_circRNA_26258	chr13	-	44970045	44993738	exonic	-2.41	6.34477	-6.52	1.80E-05	0.000427	Dtnbp1
MCAO 24h v.s. SHAM	mmu_circRNA_21208	chr1	-	165357428	165429315	exonic	-2.99	10.8925	-9.53	2.73E-07	5.03E-05	Dcaf6
MCAO 24h v.s. SHAM	mmu_circRNA_20282	chr1	+	66521454	66530853	exonic	-3.065	9.39285	-4.9	0.00027932	0.0027	Unc80
MCAO 24h v.s. SHAM	mmu_circRNA_39875	chr6	-	18378366	18423981	exonic	2.031	9.81301	4.003	0.00145753	0.008559	Cttnbp2
MCAO 24h v.s. SHAM	mmu_circRNA_19300	chr3	-	116348451	116366708	sense overlapping	-2.014	9.01274	-5.18	0.00016749	0.001881	Cdc14a
MCAO 24h v.s. SHAM	mmu_circRNA_42809	chr8	+	40454497	40474807	exonic	3.587	6.07654	5.259	0.00014642	0.001736	Zdhhc2
MCAO 24h v.s. SHAM	mmu_circRNA_44100	chr9	-	47129526	47192564	intergenic	2.172	7.46507	6.74	1.28E-05	0.000342	Gm31698
MCAO 24h v.s. SHAM	mmu_circRNA_21244	chr1	+	167314109	167326020	intronic	-2.655	7.73059	-8.75	7.31E-07	6.93E-05	Tmco1
MCAO 24h v.s. SHAM	mmu_circRNA_34924	chr2	-	162135211	162278319	exonic	2.221	6.40497	6.796	1.17E-05	0.000324	Ptprt
MCAO 24h v.s. SHAM	mmu_circRNA_33179	chr2	-	25354467	25354822	exonic	2.074	9.0531	6.363	2.31E-05	0.000495	Dpp7
MCAO 24h v.s. SHAM	mmu_circRNA_003208	chr7	-	116358635	116368189	exonic	3.37	7.06679	9.288	3.70E-07	5.17E-05	Pik3c2a
MCAO 24h v.s. SHAM	mmu_circRNA_30227	chr17	-	10576464	10597281	exonic	2.24	7.88214	5.148	0.0001779	0.001958	Pacrg

MCAO 24h v.s. SHAM	mmu_circRNA_31230	chr18	-	7265000	7285792	exonic	3.379	8.901	9.894	1.77E-07	4.46E-05	Armc4
MCAO 24h v.s. SHAM	mmu_circRNA_32020	chr18	-	78584582	78644801	sense overlapping	2.254	8.11116	4.34	0.00077267	0.00552	NA
MCAO 24h v.s. SHAM	mmu_circRNA_34988	chr2	+	166921969	166941492	exonic	-2.232	12.3804	-5.57	8.60E-05	0.00122	Cse1l
MCAO 24h v.s. SHAM	mmu_circRNA_43806	chr9	-	18617594	18620536	intergenic	2.08	8.68243	4.05	0.00133435	0.008005	Muc16
MCAO 24h v.s. SHAM	mmu_circRNA_33285	chr2	+	32216045	32217335	exonic	-2.062	7.72768	-4.14	0.00112507	0.00712	Prrc2b
MCAO 24h v.s. SHAM	mmu_circRNA_38374	chr5	+	30247462	30257820	exonic	3.133	10.2574	5.859	5.25E-05	0.000865	Selenoi
MCAO 24h v.s. SHAM	mmu_circRNA_32294	chr19	-	18581111	18596401	sense overlapping	-2.1	6.33154	-7.15	6.87E-06	0.000237	Ostf1
MCAO 24h v.s. SHAM	mmu_circRNA_43900	chr9	+	25594560	25634351	sense overlapping	4.713	7.75305	9.474	2.94E-07	5.05E-05	Eepd1
MCAO 24h v.s. SHAM	mmu_circRNA_33288	chr2	-	32802753	32808062	exonic	3.15	7.24823	3.572	0.00333415	0.01559	Stxbp1
MCAO 24h v.s. SHAM	mmu_circRNA_015174	chr7	-	116333180	116333258	intronic	2.976	7.42622	5.538	9.03E-05	0.001265	Rps13
MCAO 24h v.s. SHAM	mmu_circRNA_015174	chr7	-	116333180	116333258	intronic	2.976	7.42622	5.538	9.03E-05	0.001265	Snord14a
MCAO 24h v.s. SHAM	mmu_circRNA_45876	chrX	-	157888397	157945416	exonic	5.717	9.30168	8.647	8.41E-07	7.48E-05	Cnksr2
MCAO 24h v.s. SHAM	mmu_circRNA_40599	chr6	-	97396192	97423524	exonic	-2.191	10.0346	-5.77	6.12E-05	0.000955	Frmd4b
MCAO 24h v.s. SHAM	mmu_circRNA_31968	chr18	+	74569766	74580534	exonic	-2.439	5.77255	-5.73	6.49E-05	0.000993	Myo5b
MCAO 24h v.s. SHAM	mmu_circRNA_26922	chr13	-	106822955	106860937	exonic	-2.18	7.22371	-6.41	2.14E-05	0.000472	Ipo11
MCAO 24h v.s. SHAM	mmu_circRNA_36806	chr4	+	55007532	55023606	exonic	-2.44	8.09533	-5.61	8.03E-05	0.001157	Zfp462
MCAO 24h v.s. SHAM	mmu_circRNA_20105	chr1	-	55014868	55016182	intronic	-2.155	8.67914	-5.27	0.00014415	0.001723	Sf3b1
MCAO 24h v.s. SHAM	mmu_circRNA_010498	chr2	-	143938457	143938574	antisense	4.5	12.0558	7.751	2.86E-06	0.00014	Dstn
MCAO 24h v.s. SHAM	mmu_circRNA_38691	chr5	-	65634171	65666311	exonic	-2.025	7.28969	-5.67	7.24E-05	0.001067	Pds5a
MCAO 24h v.s. SHAM	mmu_circRNA_33808	chr2	-	70738420	70757549	exonic	-2.778	9.62562	-8.65	8.35E-07	7.48E-05	Tlk1

MCAO 24h v.s. SHAM	mmu_circRNA_32218	chr19	-	7456299	7467867	exonic	4.509	11.3789	9.112	4.62E-07	5.38E-05	Rtn3
MCAO 24h v.s. SHAM	mmu_circRNA_26394	chr13	-	57512691	57513356	sense overlapping	3.931	11.9974	10.45	9.28E-08	3.99E-05	Spock1
MCAO 24h v.s. SHAM	mmu_circRNA_38328	chr5	+	28331110	28352643	exonic	-3.15	7.20658	-8.2	1.52E-06	9.37E-05	Rbm33
MCAO 24h v.s. SHAM	mmu_circRNA_45000	chr9	+	103160819	103167192	sense overlapping	4.611	7.88522	14.67	1.47E-09	1.49E-05	Rab6b
MCAO 24h v.s. SHAM	mmu_circRNA_19476	chr7	+	123143745	123162878	sense overlapping	-2.601	9.91528	-6.26	2.74E-05	0.000559	Tnrc6a
MCAO 24h v.s. SHAM	mmu_circRNA_44680	chr9	+	78452763	78465002	exonic	-2.256	6.28311	-5.66	7.32E-05	0.001075	Mto1
MCAO 24h v.s. SHAM	mmu_circRNA_30190	chr17	+	6016265	6019478	exonic	4.462	9.77447	9.789	2.01E-07	4.51E-05	Synj2
MCAO 24h v.s. SHAM	mmu_circRNA_000233	chr6	-	135922871	135923470	exonic	2.048	6.34287	3.386	0.00477187	0.020331	Grin2b
MCAO 24h v.s. SHAM	mmu_circRNA_35991	chr3	+	109508783	109563204	exonic	-2.363	10.4304	-5.06	0.00020976	0.002217	Vav3
MCAO 24h v.s. SHAM	mmu_circRNA_22898	chr11	-	11352521	11418287	exonic	-3.098	8.3935	-4.92	0.0002694	0.002639	Zpbp
MCAO 24h v.s. SHAM	mmu_circRNA_015316	chr14	-	78651680	78653796	exonic	-2.082	8.30615	-4.78	0.0003421	0.003093	Gm30716
MCAO 24h v.s. SHAM	mmu_circRNA_015316	chr14	-	78651680	78653796	exonic	-2.082	8.30615	-4.78	0.0003421	0.003093	Dgkh
MCAO 24h v.s. SHAM	mmu_circRNA_45879	chrX	-	157906669	157996684	exonic	-3.048	8.2722	-4.78	0.00034624	0.003111	Cnksr2
MCAO 24h v.s. SHAM	mmu_circRNA_30683	chr17	-	58066717	58067505	antisense	2.672	6.75121	9.308	3.61E-07	5.17E-05	Cntnap5c
MCAO 24h v.s. SHAM	mmu_circRNA_39766	chr5	+	150825533	150825960	intronic	-2.219	8.34115	-4.52	0.00055377	0.0043	Gm36447
MCAO 24h v.s. SHAM	mmu_circRNA_33661	chr2	+	61720443	61785559	exonic	-2.061	5.26327	-6.6	1.58E-05	0.000393	Psmd14
MCAO 24h v.s. SHAM	mmu_circRNA_40764	chr6	-	113416788	113417411	exonic	2.875	9.96391	8.459	1.08E-06	8.06E-05	Rpusd3
MCAO 24h v.s. SHAM	mmu_circRNA_25428	chr12	+	81894982	81919380	exonic	2.325	12.5121	7.487	4.17E-06	0.00017	Pcnx
MCAO 24h v.s. SHAM	mmu_circRNA_43988	chr9	-	36923583	36987272	exonic	2.681	11.5488	4.944	0.00025588	0.002538	Pknox2
MCAO 24h v.s. SHAM	mmu_circRNA_24855	chr12	+	29866827	29867876	intronic	2.277	6.38462	8.572	9.28E-07	7.61E-05	Myt1l
MCAO 24h v.s. SHAM	mmu_circRNA_43812	chr9	-	20444258	20445897	exonic	-2.345	6.8752	-4.74	0.00037117	0.003247	Zfp26

MCAO 24h v.s. SHAM	mmu_circRNA_25974	chr13	-	8985208	8992815	exonic	-2.509	8.60964	-5.45	0.00010443	0.001398	Gtpbp4
MCAO 24h v.s. SHAM	mmu_circRNA_25973	chr13	-	8985208	8992035	exonic	-3.194	8.28853	-6.13	3.35E-05	0.000639	Gtpbp4
MCAO 24h v.s. SHAM	mmu_circRNA_27674	chr14	+	62346567	62353637	exonic	-2.149	7.92899	-3.65	0.00288145	0.014092	Rnaseh2b
MCAO 24h v.s. SHAM	mmu_circRNA_38897	chr5	-	86016201	86052241	exonic	-2.19	8.45987	-4.54	0.00052889	0.00415	Gtdc1
MCAO 24h v.s. SHAM	mmu_circRNA_35175	chr3	+	26878562	26927389	exonic	-4.304	7.13891	-5.81	5.74E-05	0.000917	Spata16
MCAO 24h v.s. SHAM	mmu_circRNA_37766	chr4	-	133535509	133541216	exonic	5.062	8.53074	8.277	1.38E-06	8.93E-05	Nudc
MCAO 24h v.s. SHAM	mmu_circRNA_26255	chr13	+	44848279	44884880	exonic	-2.09	8.52968	-7.37	4.96E-06	0.000188	Jarid2
MCAO 24h v.s. SHAM	mmu_circRNA_003371	chr6	+	134062063	134066465	intronic	-2.104	12.7065	-5.85	5.37E-05	0.000877	Etv6
MCAO 24h v.s. SHAM	mmu_circRNA_19280	chr3	-	51308049	51326037	sense overlapping	-2.845	12.3806	-7.96	2.12E-06	0.000117	Elf2
MCAO 24h v.s. SHAM	mmu_circRNA_005039	chr3	-	51308049	51326035	exonic	-2.781	12.7021	-7.76	2.84E-06	0.00014	Elf2
MCAO 24h v.s. SHAM	mmu_circRNA_43925	chr9	+	31053239	31054313	exonic	2.195	8.09254	2.819	0.01429942	0.045634	Zbtb44
MCAO 24h v.s. SHAM	mmu_circRNA_22440	chr10	-	91158747	91166069	exonic	-2.172	7.55131	-4.91	0.00027133	0.002652	Tmpo
MCAO 24h v.s. SHAM	mmu_circRNA_30189	chr17	+	5990135	5996961	exonic	3.255	6.80978	8.864	6.34E-07	6.42E-05	Synj2
MCAO 24h v.s. SHAM	mmu_circRNA_45157	chr9	+	111190167	111199835	exonic	-2.739	6.37005	-10.3	1.05E-07	4.16E-05	Lrrfip2
MCAO 24h v.s. SHAM	mmu_circRNA_29958	chr16	+	81432708	81490394	exonic	3.974	5.82206	5.745	6.36E-05	0.000979	Ncam2
MCAO 24h v.s. SHAM	mmu_circRNA_42485	chr7	-	143258341	143259384	sense overlapping	-2.058	5.74412	-5.62	7.91E-05	0.001146	Kcnq1ot1
MCAO 24h v.s. SHAM	mmu_circRNA_42485	chr7	-	143258341	143259384	sense overlapping	-2.058	5.74412	-5.62	7.91E-05	0.001146	Kcnq1
MCAO 24h v.s. SHAM	mmu_circRNA_42579	chr8	+	14305203	14305966	intronic	2.312	7.54871	5.377	0.00011918	0.001529	Dlgap2
MCAO 24h v.s. SHAM	mmu_circRNA_19033	chr11	+	87146931	87149364	intronic	-2.749	10.0048	-7.12	7.14E-06	0.000243	Trim37
MCAO 24h v.s. SHAM	mmu_circRNA_41943	chr7	+	97854435	97871612	exonic	2.716	6.21876	6.286	2.61E-05	0.000543	Pak1

MCAO 24h v.s. SHAM	mmu_circRNA_000113	chr12	-	109603917	109603990	antisense	2.05	8.53112	5.062	0.00020718	0.002196	Rian
MCAO 24h v.s. SHAM	mmu_circRNA_29699	chr16	+	38170663	38193994	exonic	-2.76	8.57752	-7.13	7.08E-06	0.000242	Gsk3b
MCAO 24h v.s. SHAM	mmu_circRNA_40985	chr6	-	136634430	136651950	exonic	-2.8	7.52008	-5.69	6.97E-05	0.001044	Plbd1
MCAO 24h v.s. SHAM	mmu_circRNA_44266	chr9	-	56653599	56653832	exonic	3.642	7.46004	10.35	1.05E-07	4.16E-05	Lingo1
MCAO 24h v.s. SHAM	mmu_circRNA_30550	chr17	-	46824449	46830996	exonic	2.372	8.09717	6.849	1.08E-05	0.000304	Bicral
MCAO 24h v.s. SHAM	mmu_circRNA_20182	chr1	-	60006233	60014018	exonic	2.112	8.74103	6.057	3.79E-05	0.000694	Ica1l
MCAO 24h v.s. SHAM	mmu_circRNA_34829	chr2	-	155708931	155726674	exonic	-2.05	12.4543	-5.28	0.00014233	0.001713	Edem2
MCAO 24h v.s. SHAM	mmu_circRNA_010337	chr3	-	153411461	153411871	exonic	-2.172	11.2999	-5.59	8.24E-05	0.001177	St6galnac3
MCAO 24h v.s. SHAM	mmu_circRNA_012978	chr17	-	24624819	24625014	intronic	-4.292	13.128	-6.58	1.63E-05	0.000402	Tsc2
MCAO 24h v.s. SHAM	mmu_circRNA_39718	chr5	+	150186772	150196735	intronic	2.9	9.76747	8.429	1.12E-06	8.21E-05	Fry
MCAO 24h v.s. SHAM	mmu_circRNA_23135	chr11	-	29831098	29906331	exonic	2.195	6.43987	8.266	1.40E-06	8.93E-05	Eml6
MCAO 24h v.s. SHAM	mmu_circRNA_30666	chr17	-	57218564	57218671	intronic	3.119	10.6724	10.73	6.79E-08	3.89E-05	C3
MCAO 24h v.s. SHAM	mmu_circRNA_016119	chr11	+	93959411	93960695	antisense	2.454	7.25485	6.407	2.15E-05	0.000473	Nme1
MCAO 24h v.s. SHAM	mmu_circRNA_29342	chr16	-	16461593	16490606	exonic	2.077	6.61347	6.248	2.77E-05	0.000564	Fgd4
MCAO 24h v.s. SHAM	mmu_circRNA_018522	chr7	+	126864319	126864661	exonic	2.149	6.66072	8.114	1.72E-06	0.000102	Hirip3
MCAO 24h v.s. SHAM	mmu_circRNA_19277	chr3	-	27620601	27643091	sense overlapping	-3.587	11.2137	-7.04	8.13E-06	0.00026	Fndc3b
MCAO 24h v.s. SHAM	mmu_circRNA_35606	chr3	-	75298105	75339532	intergenic	3.296	10.5375	7.85	2.48E-06	0.00013	Wdr49
MCAO 24h v.s. SHAM	mmu_circRNA_39338	chr5	+	123101610	123104511	exonic	2.188	11.2006	6.487	1.89E-05	0.000437	Tmem120b
MCAO 24h v.s. SHAM	mmu_circRNA_25821	chr12	-	108862802	108867777	exonic	3.896	7.83539	9.539	2.72E-07	5.03E-05	Wars
MCAO 24h v.s. SHAM	mmu_circRNA_28998	chr15	+	94638060	94714994	exonic	3.689	8.20649	4.681	0.000412	0.003495	Tmem117
MCAO 24h v.s. SHAM	mmu_circRNA_44586	chr9	-	71515068	71555737	exonic	4.474	8.69819	6.543	1.73E-05	0.000417	Myzap

MCAO 24h v.s. SHAM	mmu_circRNA_32442	chr19	-	32223508	32352023	exonic	-2.14	10.5569	-6.04	3.91E-05	0.000709	Sgms1
MCAO 24h v.s. SHAM	mmu_circRNA_23218	chr11	-	36063088	36139761	exonic	2.263	9.75593	4.155	0.00109348	0.007003	Tenm2
MCAO 24h v.s. SHAM	mmu_circRNA_25572	chr12	-	86705266	86720663	exonic	2.199	7.91941	4.052	0.00133023	0.007984	Angel1
MCAO 24h v.s. SHAM	mmu_circRNA_22930	chr11	-	20306465	20320234	exonic	3.147	12.5212	7.129	7.05E-06	0.000242	Slc1a4
MCAO 24h v.s. SHAM	mmu_circRNA_26086	chr13	+	15477999	15613884	exonic	2.559	8.12528	5.771	6.09E-05	0.000953	Gli3
MCAO 24h v.s. SHAM	mmu_circRNA_38143	chr5	-	17811180	17835671	exonic	-2.462	8.21369	-3.28	0.00581741	0.02336	Cd36
MCAO 24h v.s. SHAM	mmu_circRNA_37532	chr4	+	120463074	120508212	exonic	-2.226	10.5524	-5.13	0.00018421	0.002015	Scmh1
MCAO 24h v.s. SHAM	mmu_circRNA_015481	chr2	+	4431198	4498073	exonic	2.942	8.0864	6.433	2.06E-05	0.000464	Gm39751
MCAO 24h v.s. SHAM	mmu_circRNA_015481	chr2	+	4431198	4498073	exonic	2.942	8.0864	6.433	2.06E-05	0.000464	Frmd4a
MCAO 24h v.s. SHAM	mmu_circRNA_36149	chr3	+	130475592	130496442	exonic	3.107	7.57463	4.556	0.00051811	0.004096	Col25a1
MCAO 24h v.s. SHAM	mmu_circRNA_33280	chr2	+	32191508	32194996	exonic	2.283	8.94716	5.62	7.85E-05	0.00114	Prrc2b
MCAO 24h v.s. SHAM	mmu_circRNA_31309	chr18	-	12058925	12070131	exonic	2.249	10.647	7.577	3.66E-06	0.000157	Tmem241
MCAO 24h v.s. SHAM	mmu_circRNA_42666	chr8	-	24878720	24902884	exonic	-2.294	7.23952	-4.6	0.00047748	0.003878	Adam32
MCAO 24h v.s. SHAM	mmu_circRNA_018406	chr5	+	16264257	16267475	exonic	2.421	6.48268	5.506	9.54E-05	0.001308	Cacna2d1
MCAO 24h v.s. SHAM	mmu_circRNA_26594	chr13	+	77245200	77281152	exonic	3.409	9.87178	7.672	3.19E-06	0.000146	2210408I21Rik
MCAO 24h v.s. SHAM	mmu_circRNA_43546	chr8	+	117555974	117558116	exonic	-2.266	10.0478	-5.69	6.99E-05	0.001044	Plcg2
MCAO 24h v.s. SHAM	mmu_circRNA_35765	chr3	+	89007141	89026506	exonic	2.602	9.31604	5.549	8.87E-05	0.00125	Ash1l
MCAO 24h v.s. SHAM	mmu_circRNA_38661	chr5	+	64311058	64324609	exonic	2.683	9.55217	7.058	7.85E-06	0.000258	Tbc1d1
MCAO 24h v.s. SHAM	mmu_circRNA_24895	chr12	-	32874945	32875233	intergenic	2.558	10.8179	7.382	4.85E-06	0.000186	NA
MCAO 24h v.s. SHAM	mmu_circRNA_003119	chr2	+	104608870	104609186	exonic	-2.196	11.3148	-6.41	2.14E-05	0.000472	Cstf3

MCAO 24h v.s. SHAM	mmu_circRNA_24053	chr11	+	88049411	88050095	exonic	-2.865	10.0034	-6.19	3.03E-05	0.000603	Srsf1
MCAO 24h v.s. SHAM	mmu_circRNA_27197	chr14	+	21774962	21780181	exonic	4.622	12.5543	6.919	9.69E-06	0.000285	Samd8
MCAO 24h v.s. SHAM	mmu_circRNA_41532	chr7	-	61786069	61814190	intronic	2.575	6.94104	9.109	4.64E-07	5.38E-05	A230057D06Rik
MCAO 24h v.s. SHAM	mmu_circRNA_009934	chr5	-	8399687	8405838	exonic	-2.047	8.03429	-6.92	9.63E-06	0.000284	Dbf4
MCAO 24h v.s. SHAM	mmu_circRNA_23058	chr11	+	24652459	24723339	sense overlapping	2.877	7.94183	7.466	4.29E-06	0.000172	NA
MCAO 24h v.s. SHAM	mmu_circRNA_41104	chr6	-	148782597	148812156	exonic	2.556	6.24501	8.243	1.44E-06	9.08E-05	Ipo8
MCAO 24h v.s. SHAM	mmu_circRNA_42073	chr7	+	110052710	110054116	exonic	-2.238	7.55347	-6.01	4.06E-05	0.000729	Ipo7
MCAO 24h v.s. SHAM	mmu_circRNA_39334	chr5	-	122947726	122961588	exonic	2.07	6.03478	6.821	1.13E-05	0.000315	Kdm2b
MCAO 24h v.s. SHAM	mmu_circRNA_39334	chr5	-	122947726	122961588	exonic	2.07	6.03478	6.821	1.13E-05	0.000315	Gm33118
MCAO 24h v.s. SHAM	mmu_circRNA_30491	chr17	+	35846301	35848665	exonic	-2.312	7.16074	-3.43	0.0043662	0.019092	Mdc1
MCAO 24h v.s. SHAM	mmu_circRNA_45523	chrX	-	74035347	74080032	sense overlapping	3.841	8.21785	7.945	2.17E-06	0.000119	Mecp2
MCAO 24h v.s. SHAM	mmu_circRNA_42792	chr8	-	36850163	36938758	exonic	2.955	7.30131	6.482	1.91E-05	0.000439	Dlc1
MCAO 24h v.s. SHAM	mmu_circRNA_34737	chr2	+	146870765	146891284	exonic	2.048	9.34661	7.004	8.52E-06	0.000267	Kiz
MCAO 24h v.s. SHAM	mmu_circRNA_43073	chr8	+	76906592	77085666	sense overlapping	3.827	6.54018	6.301	2.55E-05	0.000535	Nr3c2
MCAO 24h v.s. SHAM	mmu_circRNA_000692	chr9	-	86372340	86452010	exonic	2.768	6.20701	6.663	1.44E-05	0.000371	Ube2cbp
MCAO 24h v.s. SHAM	mmu_circRNA_21880	chr10	-	41922121	41951746	exonic	3.365	10.4083	8.597	8.98E-07	7.59E-05	Armc2
MCAO 24h v.s. SHAM	mmu_circRNA_40710	chr6	+	108369010	108440687	exonic	-2.067	6.60166	-4.37	0.00072929	0.005265	Itpr1
MCAO 24h v.s. SHAM	mmu_circRNA_45251	chr9	-	119489748	119492186	exonic	2.411	8.30375	7.53	3.91E-06	0.000164	Scn5a
MCAO 24h v.s. SHAM	mmu_circRNA_006917	chr8	+	82990346	83006412	exonic	2.349	6.76574	7.984	2.06E-06	0.000115	Rnf150
MCAO 24h v.s. SHAM	mmu_circRNA_41166	chr7	-	4430393	4435098	exonic	2.211	11.0901	6.185	3.07E-05	0.000606	Rdh13
MCAO 24h v.s. SHAM	mmu_circRNA_31317	chr18	+	12684836	12698059	exonic	3.086	10.9156	7.041	8.05E-06	0.00026	Ttc39c

MCAO 24h v.s. SHAM	mmu_circRNA_009247	chr19	-	37044521	37088587	exonic	2.082	11.656	5.961	4.43E-05	0.000774	Cpeb3
MCAO 24h v.s. SHAM	mmu_circRNA_38102	chr5	-	9569918	9590184	exonic	3.12	7.74002	8.439	1.11E-06	8.16E-05	Grm3
MCAO 24h v.s. SHAM	mmu_circRNA_008308	chr2	-	93012626	93013889	exonic	2.487	6.985	4.247	0.0009209	0.00623	Prdm11
MCAO 24h v.s. SHAM	mmu_circRNA_36481	chr4	+	11593620	11605005	exonic	5.2	12.1502	7.565	3.72E-06	0.000159	Rad54b
MCAO 24h v.s. SHAM	mmu_circRNA_24344	chr11	+	107485549	107489072	exonic	5.554	9.89977	9.022	5.18E-07	5.81E-05	Psmd12
MCAO 24h v.s. SHAM	mmu_circRNA_39856	chr6	+	17654105	17662169	exonic	-3.3	11.1066	-6.49	1.89E-05	0.000437	Capza2
MCAO 24h v.s. SHAM	mmu_circRNA_38760	chr5	-	72329611	72343117	exonic	-2.589	7.15788	-7.22	6.21E-06	0.000219	Corin
MCAO 24h v.s. SHAM	mmu_circRNA_38483	chr5	+	34834260	34854791	exonic	-2.769	9.96792	-5.26	0.00014707	0.001739	Htt
MCAO 24h v.s. SHAM	mmu_circRNA_30783	chr17	-	65858972	65867830	exonic	-2.816	6.9715	-7.62	3.45E-06	0.000152	Ralbp1
MCAO 24h v.s. SHAM	mmu_circRNA_30778	chr17	+	65837683	65838024	exonic	2.113	7.59016	7.091	7.48E-06	0.000248	Ppp4r1
MCAO 24h v.s. SHAM	mmu_circRNA_39443	chr5	-	128988801	128999587	exonic	-2.018	9.21534	-4.32	0.00080337	0.005681	Stx2
MCAO 24h v.s. SHAM	mmu_circRNA_41768	chr7	-	80923100	80927886	exonic	-2.174	8.255	-5.83	5.54E-05	0.000894	Sec11a
MCAO 24h v.s. SHAM	mmu_circRNA_017159	chr3	+	133320935	133330512	exonic	2.639	9.29524	8.76	7.25E-07	6.93E-05	Ppa2
MCAO 24h v.s. SHAM	mmu_circRNA_24439	chr11	-	116545833	116546968	exonic	-2.34	11.736	-7.33	5.20E-06	0.000195	Ube2o
MCAO 24h v.s. SHAM	mmu_circRNA_000704	chr7	+	96729348	96737561	exonic	2.714	5.77833	3.197	0.00689063	0.026485	Tenm4
MCAO 24h v.s. SHAM	mmu_circRNA_43649	chr8	-	128199297	128207761	sense overlapping	2.391	6.68362	6.992	8.67E-06	0.000269	NA
MCAO 24h v.s. SHAM	mmu_circRNA_21120	chr1	+	159861209	159868285	exonic	3.004	9.45099	6.437	2.05E-05	0.000462	Tnr
MCAO 24h v.s. SHAM	mmu_circRNA_26097	chr13	-	16002457	16003880	intronic	-4.265	8.40068	-6.02	4.03E-05	0.000725	NA
MCAO 24h v.s. SHAM	mmu_circRNA_23636	chr11	-	72070302	72075035	exonic	2.254	7.53127	8.517	9.98E-07	7.81E-05	Pitpnm3
MCAO 24h v.s. SHAM	mmu_circRNA_24642	chr12	-	5137422	5140708	exonic	-2.413	11.9372	-4.37	0.00073412	0.005296	Klhl29

MCAO 24h v.s. SHAM	mmu_circRNA_43201	chr8	-	84707659	84713857	sense overlapping	3.58	7.28274	10.8	6.28E-08	3.81E-05	Nfix
MCAO 24h v.s. SHAM	mmu_circRNA_24450	chr11	+	117735971	117740009	exonic	4.25	7.00855	9.707	2.22E-07	4.58E-05	Tnrc6c
MCAO 24h v.s. SHAM	mmu_circRNA_24450	chr11	+	117735971	117740009	exonic	4.25	7.00855	9.707	2.22E-07	4.58E-05	Gm11734
MCAO 24h v.s. SHAM	mmu_circRNA_44643	chr9	+	75193938	75215259	exonic	-2.561	8.55	-5.67	7.17E-05	0.001064	Myo5a
MCAO 24h v.s. SHAM	mmu_circRNA_24798	chr12	-	21353882	21361791	exonic	2.376	8.06477	7.292	5.54E-06	0.000202	Adam17
MCAO 24h v.s. SHAM	mmu_circRNA_29599	chr16	-	34116221	34220247	exonic	2.495	8.29917	7.875	2.40E-06	0.000126	Kalrn
MCAO 24h v.s. SHAM	mmu_circRNA_014144	chr8	+	108856721	108856953	exonic	-2.216	8.59907	-4.64	0.00044076	0.003664	Zfhx3
MCAO 24h v.s. SHAM	mmu_circRNA_26563	chr13	+	76641711	76759820	exonic	-2.004	8.7906	-5.93	4.68E-05	0.000802	Mctp1
MCAO 24h v.s. SHAM	mmu_circRNA_38590	chr5	-	51472597	51474411	exonic	3.241	11.8476	8.363	1.23E-06	8.55E-05	Ppargc1a
MCAO 24h v.s. SHAM	mmu_circRNA_33119	chr2	+	23075489	23087619	exonic	-2.461	7.14529	-5.49	9.81E-05	0.001335	Acbd5
MCAO 24h v.s. SHAM	mmu_circRNA_20913	chr1	-	135532348	135537299	exonic	-2.21	6.5197	-6.22	2.89E-05	0.000581	Nav1
MCAO 24h v.s. SHAM	mmu_circRNA_29760	chr16	+	44355439	44377022	exonic	4.412	11.9177	8.111	1.73E-06	0.000102	Spice1
MCAO 24h v.s. SHAM	mmu_circRNA_42216	chr7	+	118872820	118899886	exonic	2.14	6.06002	6.751	1.25E-05	0.000338	Iqck
MCAO 24h v.s. SHAM	mmu_circRNA_23911	chr11	-	84839980	84841624	exonic	-2.433	6.428	-5.89	4.97E-05	0.000829	Ggnbp2
MCAO 24h v.s. SHAM	mmu_circRNA_31938	chr18	-	71446336	71588027	exonic	2.219	11.3606	6.866	1.05E-05	0.0003	Dcc
MCAO 24h v.s. SHAM	mmu_circRNA_44832	chr9	+	95998215	96006847	exonic	-2.339	7.54576	-4.49	0.00058623	0.004481	Xrn1
MCAO 24h v.s. SHAM	mmu_circRNA_23290	chr11	-	50301757	50308929	exonic	-2.282	10.6565	-7.03	8.14E-06	0.00026	Canx
MCAO 24h v.s. SHAM	mmu_circRNA_32945	chr2	-	6721416	6747950	intronic	2.789	7.96055	4.881	0.00028659	0.002755	Celf2
MCAO 24h v.s. SHAM	mmu_circRNA_20389	chr1	-	79713857	79715000	exonic	-2.011	6.13515	-7.04	8.10E-06	0.00026	Wdfy1
MCAO 24h v.s. SHAM	mmu_circRNA_40611	chr6	-	99162838	99669567	sense overlapping	3.391	11.8573	7.926	2.23E-06	0.000121	NA
MCAO 24h v.s. SHAM	mmu_circRNA_32069	chr18	-	82618742	82624486	exonic	-2.51	6.93891	-6.39	2.21E-05	0.000481	Zfp236

MCAO 24h v.s. SHAM	mmu_circRNA_25329	chr12	+	73926556	73937800	exonic	-3	10.6729	-3.33	0.00532311	0.022033	Hif1a
MCAO 24h v.s. SHAM	mmu_circRNA_25329	chr12	+	73926556	73937800	exonic	-3	10.6729	-3.33	0.00532311	0.022033	Gm15283
MCAO 24h v.s. SHAM	mmu_circRNA_25607	chr12	-	91213564	91299077	exonic	-2.95	9.15897	-7.37	4.94E-06	0.000188	Cep128
MCAO 24h v.s. SHAM	mmu_circRNA_43263	chr8	-	86724205	86725856	sense overlapping	2.253	13.3906	5.371	0.00012045	0.001537	Siah1a
MCAO 24h v.s. SHAM	mmu_circRNA_43998	chr9	-	37311317	37316527	exonic	-2.068	6.53499	-4.2	0.00099991	0.006588	Ccdc15
MCAO 24h v.s. SHAM	mmu_circRNA_32884	chr2	-	5354668	5362127	exonic	-3.75	11.6701	-6.97	8.99E-06	0.000273	Camk1d
MCAO 24h v.s. SHAM	mmu_circRNA_28610	chr15	+	55208730	55218273	exonic	2.373	6.0862	6.087	3.60E-05	0.00067	Deptor
MCAO 24h v.s. SHAM	mmu_circRNA_21349	chr1	-	177050037	177109738	exonic	-2.026	8.20921	-6.06	3.77E-05	0.000693	Akt3
MCAO 24h v.s. SHAM	mmu_circRNA_37971	chr4	+	150508829	150570296	exonic	2.595	7.5097	4.848	0.0003042	0.00286	Rere
MCAO 24h v.s. SHAM	mmu_circRNA_35751	chr3	-	88910313	88910810	exonic	-3.144	9.14707	-5.83	5.54E-05	0.000894	Msto1
MCAO 24h v.s. SHAM	mmu_circRNA_19193	chr17	+	57419319	57420525	sense overlapping	2.525	6.1426	6.354	2.34E-05	0.0005	Adgre1
MCAO 24h v.s. SHAM	mmu_circRNA_39827	chr6	+	8280220	8291914	intronic	3.334	6.41158	6.918	9.71E-06	0.000285	Umad1
MCAO 24h v.s. SHAM	mmu_circRNA_30884	chr17	-	73906009	73907745	exonic	5.382	9.06227	8.609	8.84E-07	7.59E-05	Xdh
MCAO 24h v.s. SHAM	mmu_circRNA_41079	chr6	+	146866736	146868264	exonic	-2.515	6.50603	-4.91	0.00027068	0.002649	Smco2
MCAO 24h v.s. SHAM	mmu_circRNA_015601	chr5	-	99240822	99243246	exonic	2.051	9.1555	5.212	0.00015907	0.001829	A930011G23Rik
MCAO 24h v.s. SHAM	mmu_circRNA_015601	chr5	-	99240822	99243246	exonic	2.051	9.1555	5.212	0.00015907	0.001829	Rasgef1b
MCAO 24h v.s. SHAM	mmu_circRNA_35962	chr3	-	107445429	107448440	exonic	3.738	12.5675	8.372	1.21E-06	8.51E-05	Kcnc4
MCAO 24h v.s. SHAM	mmu_circRNA_26316	chr13	+	49820612	49830616	intergenic	-2.672	8.79094	-4.87	0.00029359	0.002804	LOC102631805
MCAO 24h v.s. SHAM	mmu_circRNA_013699	chr10	-	9841870	9843361	exonic	-2.35	10.4659	-7.25	5.86E-06	0.000211	Stxbp5
MCAO 24h v.s. SHAM	mmu_circRNA_23961	chr11	-	86108766	86157878	exonic	2.168	8.36419	7.284	5.61E-06	0.000203	Brip1

MCAO 24h v.s. SHAM	mmu_circRNA_25530	chr12	+	85826452	85933674	exonic	2.08	9.72457	5.896	4.94E-05	0.000828	Ttll5
MCAO 24h v.s. SHAM	mmu_circRNA_43896	chr9	-	25124871	25142311	sense overlapping	-2.449	6.55179	-6.01	4.12E-05	0.000735	Herpud2
MCAO 24h v.s. SHAM	mmu_circRNA_23881	chr11	+	83321938	83336861	exonic	3.509	11.8823	7.697	3.08E-06	0.000143	Ap2b1
MCAO 24h v.s. SHAM	mmu_circRNA_43831	chr9	+	21655626	21660871	exonic	2.062	9.29955	5.063	0.00020694	0.002196	Smarca4
MCAO 24h v.s. SHAM	mmu_circRNA_010147	chr19	-	37068172	37088587	exonic	3.579	11.8228	7.791	2.70E-06	0.000137	Cpeb3
MCAO 24h v.s. SHAM	mmu_circRNA_31790	chr18	+	60546220	60556370	sense overlapping	-2.017	6.19422	-6.04	3.92E-05	0.000709	Dctn4
MCAO 24h v.s. SHAM	mmu_circRNA_44088	chr9	-	45943050	45944486	exonic	4.716	11.2441	10.03	1.51E-07	4.38E-05	Sidt2
MCAO 24h v.s. SHAM	mmu_circRNA_22088	chr10	-	62274446	62286652	exonic	3.973	9.9047	7.684	3.14E-06	0.000144	Hk1
MCAO 24h v.s. SHAM	mmu_circRNA_42738	chr8	+	31114602	31118587	sense overlapping	3.518	8.6777	7.432	4.51E-06	0.000177	Rnf122
MCAO 24h v.s. SHAM	mmu_circRNA_42273	chr7	+	122754505	122762323	exonic	5.272	9.65921	10.53	8.48E-08	3.91E-05	Cacng3
MCAO 24h v.s. SHAM	mmu_circRNA_27031	chr14	-	10729724	10751694	exonic	-3.145	6.63967	-7.6	3.53E-06	0.000154	Fhit
MCAO 24h v.s. SHAM	mmu_circRNA_27031	chr14	-	10729724	10751694	exonic	-3.145	6.63967	-7.6	3.53E-06	0.000154	Fhitos
MCAO 24h v.s. SHAM	mmu_circRNA_37727	chr4	+	130727997	130774604	exonic	-3.306	12.7307	-8.61	8.82E-07	7.59E-05	Pum1
MCAO 24h v.s. SHAM	mmu_circRNA_42362	chr7	+	129629917	129632421	exonic	4.181	10.6303	6.51	1.83E-05	0.000429	Wdr11
MCAO 24h v.s. SHAM	mmu_circRNA_41406	chr7	+	49780308	49829030	exonic	2.889	9.0492	6.153	3.23E-05	0.000621	Prmt3
MCAO 24h v.s. SHAM	mmu_circRNA_29484	chr16	-	30545125	30547469	exonic	2.329	10.6491	4.453	0.00062729	0.004703	Tmem44
MCAO 24h v.s. SHAM	mmu_circRNA_30990	chr17	-	80251231	80267639	exonic	2.067	9.2142	6.559	1.69E-05	0.000409	Dhx57
MCAO 24h v.s. SHAM	mmu_circRNA_017949	chr6	-	37029762	37067385	exonic	2.027	6.6825	3.012	0.00986069	0.034419	Dgki
MCAO 24h v.s. SHAM	mmu_circRNA_38782	chr5	+	73510885	73544155	exonic	3.756	7.02379	5.405	0.00011365	0.001483	Dcun1d4
MCAO 24h v.s. SHAM	mmu_circRNA_41054	chr6	-	145032782	145039635	exonic	-3.744	13.9357	-4.59	0.00048663	0.003928	Bcat1
MCAO 24h v.s. SHAM	mmu_circRNA_24514	chr11	-	121623431	121630867	exonic	-2.061	14.2614	-7.63	3.39E-06	0.000152	B3gntl1

MCAO 24h v.s. SHAM	mmu_circRNA_40842	chr6	-	119361827	119365380	exonic	-2.604	10.7779	-6.17	3.15E-05	0.000613	Adipor2
MCAO 24h v.s. SHAM	mmu_circRNA_31621	chr18	+	37020094	37159353	intronic	2.43	6.29889	5.442	0.00010664	0.001419	Gm38666
MCAO 24h v.s. SHAM	mmu_circRNA_31621	chr18	+	37020094	37159353	intronic	2.43	6.29889	5.442	0.00010664	0.001419	Pcdha1
MCAO 24h v.s. SHAM	mmu_circRNA_31621	chr18	+	37020094	37159353	intronic	2.43	6.29889	5.442	0.00010664	0.001419	Pcdha5
MCAO 24h v.s. SHAM	mmu_circRNA_31621	chr18	+	37020094	37159353	intronic	2.43	6.29889	5.442	0.00010664	0.001419	Pcdha7
MCAO 24h v.s. SHAM	mmu_circRNA_31621	chr18	+	37020094	37159353	intronic	2.43	6.29889	5.442	0.00010664	0.001419	Gm38667
MCAO 24h v.s. SHAM	mmu_circRNA_31621	chr18	+	37020094	37159353	intronic	2.43	6.29889	5.442	0.00010664	0.001419	Pcdha4
MCAO 24h v.s. SHAM	mmu_circRNA_31621	chr18	+	37020094	37159353	intronic	2.43	6.29889	5.442	0.00010664	0.001419	Pcdha9
MCAO 24h v.s. SHAM	mmu_circRNA_31621	chr18	+	37020094	37159353	intronic	2.43	6.29889	5.442	0.00010664	0.001419	Gm37013
MCAO 24h v.s. SHAM	mmu_circRNA_31621	chr18	+	37020094	37159353	intronic	2.43	6.29889	5.442	0.00010664	0.001419	Pcdha10
MCAO 24h v.s. SHAM	mmu_circRNA_31621	chr18	+	37020094	37159353	intronic	2.43	6.29889	5.442	0.00010664	0.001419	Pcdha6
MCAO 24h v.s. SHAM	mmu_circRNA_31621	chr18	+	37020094	37159353	intronic	2.43	6.29889	5.442	0.00010664	0.001419	Pcdha3
MCAO 24h v.s. SHAM	mmu_circRNA_31621	chr18	+	37020094	37159353	intronic	2.43	6.29889	5.442	0.00010664	0.001419	Pcdha8
MCAO 24h v.s. SHAM	mmu_circRNA_31621	chr18	+	37020094	37159353	intronic	2.43	6.29889	5.442	0.00010664	0.001419	Pcdha11
MCAO 24h v.s. SHAM	mmu_circRNA_31621	chr18	+	37020094	37159353	intronic	2.43	6.29889	5.442	0.00010664	0.001419	Pcdha2
MCAO 24h v.s. SHAM	mmu_circRNA_45613	chrX	-	94535859	94535994	sense overlapping	3.41	6.13883	5.996	4.18E-05	0.000742	Maged1
MCAO 24h v.s. SHAM	mmu_circRNA_34837	chr2	-	156096417	156103397	exonic	2.407	6.71151	9.506	2.83E-07	5.03E-05	Cpne1
MCAO 24h v.s. SHAM	mmu_circRNA_34837	chr2	-	156096417	156103397	exonic	2.407	6.71151	9.506	2.83E-07	5.03E-05	Rbm12
MCAO 24h v.s. SHAM	mmu_circRNA_29464	chr16	+	24559591	24608317	exonic	-2.065	6.90893	-5.21	0.00015994	0.001831	Lpp
MCAO 24h v.s. SHAM	mmu_circRNA_36877	chr4	-	59514273	59517755	exonic	-2.093	13.7324	-4.63	0.00044943	0.003712	Ptbp3

MCAO 24h v.s. SHAM	mmu_circRNA_43751	chr9	-	9583050	9634385	intergenic	-2.632	10.5206	-4.83	0.00031327	0.002919	LOC108167638
MCAO 24h v.s. SHAM	mmu_circRNA_34013	chr2	+	90751067	90753190	exonic	-2.033	7.04717	-5.58	8.39E-05	0.001192	Fnbp4
MCAO 24h v.s. SHAM	mmu_circRNA_38722	chr5	-	67398199	67405519	sense overlapping	-3.131	11.0972	-5.18	0.00016941	0.001895	Gm33345
MCAO 24h v.s. SHAM	mmu_circRNA_38722	chr5	-	67398199	67405519	sense overlapping	-3.131	11.0972	-5.18	0.00016941	0.001895	Bend4
MCAO 24h v.s. SHAM	mmu_circRNA_28019	chr14	+	116116298	116183082	intronic	3.238	5.33604	4.268	0.00088425	0.006074	Gpc5
MCAO 24h v.s. SHAM	mmu_circRNA_002520	chr7	-	59507338	59568724	sense overlapping	4.989	8.05372	3.114	0.00809263	0.029775	Snhg14
MCAO 24h v.s. SHAM	mmu_circRNA_40590	chr6	-	94654846	94663998	exonic	3.862	12.122	8.218	1.49E-06	9.34E-05	Lrig1
MCAO 24h v.s. SHAM	mmu_circRNA_29063	chr15	-	99278688	99285665	exonic	-2.006	9.37541	-4.35	0.00076583	0.005479	Fam186b
MCAO 24h v.s. SHAM	mmu_circRNA_33110	chr2	-	20887105	20914811	exonic	-2.38	10.0237	-5.93	4.70E-05	0.000802	Arhgap21
MCAO 24h v.s. SHAM	mmu_circRNA_013226	chr2	-	144255339	144257195	exonic	-2.326	9.21968	-7.06	7.88E-06	0.000258	Snx5
MCAO 24h v.s. SHAM	mmu_circRNA_42581	chr8	+	14305203	14314292	intronic	2.626	7.54848	6.46	1.98E-05	0.000452	Dlgap2
MCAO 24h v.s. SHAM	mmu_circRNA_22847	chr11	-	4782186	4820493	exonic	-2.727	6.87177	-5.34	0.00012782	0.001598	Nf2
MCAO 24h v.s. SHAM	mmu_circRNA_24939	chr12	-	36118986	36134955	exonic	-2.12	12.5723	-5.87	5.14E-05	0.000852	Bzw2
MCAO 24h v.s. SHAM	mmu_circRNA_44014	chr9	-	42000243	42014574	exonic	3.219	8.5422	8.741	7.44E-07	6.98E-05	Sorl1
MCAO 24h v.s. SHAM	mmu_circRNA_004260	chr6	+	17834616	17855028	exonic	-2.107	9.83718	-7	8.52E-06	0.000267	St7
MCAO 24h v.s. SHAM	mmu_circRNA_21090	chr1	-	156869440	156901890	exonic	-2.234	10.1622	-5.68	7.04E-05	0.001048	Ralgps2
MCAO 24h v.s. SHAM	mmu_circRNA_32405	chr19	-	28938231	28944454	exonic	2.663	8.13973	5.281	0.00014088	0.001697	4430402I18Rik
MCAO 24h v.s. SHAM	mmu_circRNA_41535	chr7	-	61813261	61913335	exonic	2.367	6.64059	7.092	7.46E-06	0.000248	A230057D06Rik
MCAO 24h v.s. SHAM	mmu_circRNA_42207	chr7	+	118638128	118640007	exonic	2.705	7.98536	6.95	9.25E-06	0.000277	Tmc5
MCAO 24h v.s. SHAM	mmu_circRNA_27287	chr14	+	27449945	27459851	exonic	4.866	12.2679	8.495	1.03E-06	7.91E-05	Fam208a
MCAO 24h v.s. SHAM	mmu_circRNA_21469	chr1	-	181184250	181186067	exonic	-2.095	10.7396	-4.19	0.00102907	0.006707	Wdr26

MCAO 24h v.s. SHAM	mmu_circRNA_27821	chr14	+	77268806	77441899	exonic	4.988	10.955	8.959	5.61E-07	6.03E-05	Enox1
MCAO 24h v.s. SHAM	mmu_circRNA_37824	chr4	+	138057948	138105436	exonic	2.143	12.6063	6.999	8.59E-06	0.000268	Eif4g3
MCAO 24h v.s. SHAM	mmu_circRNA_26177	chr13	-	30856653	30911250	exonic	-2.061	8.06958	-5.14	0.00018002	0.001975	Exoc2
MCAO 24h v.s. SHAM	mmu_circRNA_33806	chr2	-	70721557	70749327	exonic	2.144	8.43554	3.887	0.001819	0.010064	Tlk1
MCAO 24h v.s. SHAM	mmu_circRNA_31671	chr18	+	42288107	42305814	exonic	2.968	10.5446	6.926	9.59E-06	0.000284	Rbm27
MCAO 24h v.s. SHAM	mmu_circRNA_24944	chr12	+	36501977	36522051	exonic	2.838	10.1168	8.027	1.94E-06	0.00011	Ispd
MCAO 24h v.s. SHAM	mmu_circRNA_33003	chr2	+	14981305	14984439	exonic	-2.305	11.6408	-5.04	0.00021657	0.00227	Cacnb2
MCAO 24h v.s. SHAM	mmu_circRNA_30586	chr17	+	50711410	50718783	intergenic	4.423	10.1033	6.275	2.66E-05	0.000549	NA
MCAO 24h v.s. SHAM	mmu_circRNA_27546	chr14	-	51905612	51905724	sense overlapping	2.275	8.02883	3.497	0.0038475	0.017338	Ndrg2
MCAO 24h v.s. SHAM	mmu_circRNA_31169	chr17	-	94694281	94695119	intergenic	5.854	7.9631	9.671	2.31E-07	4.60E-05	NA
MCAO 24h v.s. SHAM	mmu_circRNA_24167	chr11	+	98257346	98267858	sense overlapping	-3.232	10.0477	-7.51	4.05E-06	0.000168	NA
MCAO 24h v.s. SHAM	mmu_circRNA_38545	chr5	+	43709043	43714601	exonic	3.74	10.5431	9.204	4.11E-07	5.26E-05	Cc2d2a
MCAO 24h v.s. SHAM	mmu_circRNA_37781	chr4	+	134934931	134936081	exonic	2.407	7.54487	6.076	3.67E-05	0.00068	Syf2
MCAO 24h v.s. SHAM	mmu_circRNA_38573	chr5	-	45735730	45795358	exonic	3.997	9.57818	6.909	9.83E-06	0.000287	Lcorl
MCAO 24h v.s. SHAM	mmu_circRNA_38350	chr5	-	29323801	29346906	exonic	2	10.9308	6.296	2.57E-05	0.000538	Lmbr1
MCAO 24h v.s. SHAM	mmu_circRNA_22663	chr10	-	116343842	116346937	antisense	4.969	9.56817	9.576	2.60E-07	4.97E-05	Ptprb
MCAO 24h v.s. SHAM	mmu_circRNA_45608	chrX	-	93800113	93814068	exonic	-2.377	9.58389	-5.16	0.00017389	0.001928	Pdk3
MCAO 24h v.s. SHAM	mmu_circRNA_38258	chr5	-	23979487	24000019	exonic	-2.139	10.2283	-4.18	0.00103464	0.006731	Fam126a
MCAO 24h v.s. SHAM	mmu_circRNA_40915	chr6	-	124436817	124438985	exonic	2.146	8.94444	5.491	9.80E-05	0.001335	Clstn3
MCAO 24h v.s. SHAM	mmu_circRNA_27264	chr14	+	26900422	26934532	sense overlapping	4.53	7.76428	8.622	8.69E-07	7.59E-05	Appl1

MCAO 24h v.s. SHAM	mmu_circRNA_003795	chr1	-	155847109	155848727	sense overlapping	-4.507	11.5818	-7.92	2.25E-06	0.000121	Cep350
MCAO 24h v.s. SHAM	mmu_circRNA_36100	chr3	+	122794259	122843108	exonic	-2.173	8.82627	-6.05	3.84E-05	0.000701	4930447N08Rik
MCAO 24h v.s. SHAM	mmu_circRNA_36100	chr3	+	122794259	122843108	exonic	-2.173	8.82627	-6.05	3.84E-05	0.000701	Pde5a
MCAO 24h v.s. SHAM	mmu_circRNA_008640	chr6	+	55460156	55461614	exonic	2.11	8.75301	2.859	0.01324814	0.043048	Adcyap1r1
MCAO 24h v.s. SHAM	mmu_circRNA_004960	chr6	-	115969875	115970248	exonic	2.851	8.8388	8.209	1.51E-06	9.37E-05	Plxnd1
MCAO 24h v.s. SHAM	mmu_circRNA_19110	chr14	-	19904158	19904277	intronic	4.006	11.1538	8.272	1.39E-06	8.93E-05	Gng2
MCAO 24h v.s. SHAM	mmu_circRNA_009044	chr7	-	67529585	67559969	exonic	2.062	8.2101	5.632	7.70E-05	0.001125	Gm34079
MCAO 24h v.s. SHAM	mmu_circRNA_009044	chr7	-	67529585	67559969	exonic	2.062	8.2101	5.632	7.70E-05	0.001125	Lrrc28
MCAO 24h v.s. SHAM	mmu_circRNA_45033	chr9	-	106239531	106241785	exonic	-2.698	12.3737	-6.43	2.08E-05	0.000465	Alas1
MCAO 24h v.s. SHAM	mmu_circRNA_34423	chr2	-	121394346	121413480	sense overlapping	2.288	8.23884	5.672	7.19E-05	0.001064	Catsper2
MCAO 24h v.s. SHAM	mmu_circRNA_28026	chr14	+	118153568	118160184	exonic	4.135	8.81652	9.123	4.55E-07	5.38E-05	Gpr180
MCAO 24h v.s. SHAM	mmu_circRNA_25460	chr12	+	82677352	82681408	intronic	-2.414	7.13749	-6.1	3.53E-05	0.00066	Rgs6
MCAO 24h v.s. SHAM	mmu_circRNA_22790	chr10	+	128207297	128208667	exonic	3.222	7.95375	5.101	0.0001936	0.002092	Gls2
MCAO 24h v.s. SHAM	mmu_circRNA_41481	chr7	-	59284025	59317973	sense overlapping	3.228	9.50828	8.666	8.20E-07	7.43E-05	Snhg14
MCAO 24h v.s. SHAM	mmu_circRNA_43465	chr8	+	110645757	110671182	exonic	2.188	8.49979	7.455	4.37E-06	0.000173	Vac14
MCAO 24h v.s. SHAM	mmu_circRNA_26040	chr13	+	13725813	13761201	exonic	-2.312	7.61351	-4.29	0.00084276	0.005887	Lyst
MCAO 24h v.s. SHAM	mmu_circRNA_41647	chr7	+	70474179	70494757	sense overlapping	2.138	6.27192	8.498	1.02E-06	7.91E-05	NA
MCAO 24h v.s. SHAM	mmu_circRNA_33249	chr2	-	30110204	30111332	exonic	-2.002	8.18039	-6.73	1.29E-05	0.000344	Zer1
MCAO 24h v.s. SHAM	mmu_circRNA_002246	chr7	-	142651460	142651527	sense overlapping	-4.67	7.52559	-7.01	8.38E-06	0.000265	Igf2
MCAO 24h v.s. SHAM	mmu_circRNA_19314	chr4	-	3080977	3084320	intergenic	4.22	10.6166	8.915	5.94E-07	6.22E-05	NA
MCAO 24h v.s. SHAM	mmu_circRNA_006741	chr6	-	119052600	119057515	exonic	3.192	8.357	9.08	4.81E-07	5.51E-05	Cacna1c

MCAO 24h v.s. SHAM	mmu_circRNA_22526	chr10	+	99402065	99402880	sense overlapping	5.507	8.75673	11.1	4.55E-08	3.37E-05	NA
MCAO 24h v.s. SHAM	mmu_circRNA_009489	chr6	+	29372580	29372670	intronic	2.199	8.22179	7.437	4.48E-06	0.000177	Calu
MCAO 24h v.s. SHAM	mmu_circRNA_38919	chr5	+	88934748	88954957	exonic	3.286	8.87298	7.778	2.75E-06	0.000137	Slc4a4
MCAO 24h v.s. SHAM	mmu_circRNA_30520	chr17	-	45642467	45642962	exonic	3.29	7.24195	3.325	0.00537598	0.022158	Capn11
MCAO 24h v.s. SHAM	mmu_circRNA_24456	chr11	-	118182219	118182913	exonic	2.105	8.78585	6.854	1.07E-05	0.000304	Cyth1
MCAO 24h v.s. SHAM	mmu_circRNA_41238	chr7	-	27541084	27542181	exonic	2.957	10.0306	7.616	3.46E-06	0.000152	Pld3
MCAO 24h v.s. SHAM	mmu_circRNA_30332	chr17	+	25145568	25155537	exonic	3.131	9.76241	9.025	5.16E-07	5.81E-05	Clcn7
MCAO 24h v.s. SHAM	mmu_circRNA_34860	chr2	+	156664798	156711261	exonic	3.16	9.01726	8.773	7.14E-07	6.91E-05	Dlgap4
MCAO 24h v.s. SHAM	mmu_circRNA_23585	chr11	+	69026069	69026579	sense overlapping	2.084	8.40521	6.393	2.20E-05	0.000479	Ctc1
MCAO 24h v.s. SHAM	mmu_circRNA_19015	chr11	+	53893644	53981397	sense overlapping	3.761	10.4419	6.055	3.80E-05	0.000695	Gm30927
MCAO 24h v.s. SHAM	mmu_circRNA_42567	chr8	+	12806649	12813155	exonic	2.595	11.152	6.065	3.74E-05	0.00069	Atp11a
MCAO 24h v.s. SHAM	mmu_circRNA_19198	chr17	+	68455690	68463021	sense overlapping	2.354	5.61195	7.241	5.97E-06	0.000213	L3mbtl4
MCAO 24h v.s. SHAM	mmu_circRNA_38255	chr5	+	23807120	23819951	sense overlapping	-2.633	9.32812	-3.36	0.0050436	0.021115	Rint1
MCAO 24h v.s. SHAM	mmu_circRNA_45137	chr9	-	110432433	110439058	exonic	2.324	10.1696	5.209	0.00015997	0.001831	Klhl18
MCAO 24h v.s. SHAM	mmu_circRNA_28632	chr15	+	62054793	62107465	intronic	2.308	10.8688	5.025	0.00022147	0.002303	Pvt1
MCAO 24h v.s. SHAM	mmu_circRNA_30869	chr17	+	71695696	71697996	exonic	2.323	6.15365	6.23	2.85E-05	0.000577	Togaram2
MCAO 24h v.s. SHAM	mmu_circRNA_36895	chr4	+	65176208	65237931	exonic	2.259	9.9772	5.941	4.58E-05	0.000792	Pappa
MCAO 24h v.s. SHAM	mmu_circRNA_23261	chr11	-	46221335	46224205	exonic	3.172	10.9546	7.476	4.23E-06	0.000171	Cyfip2
MCAO 24h v.s. SHAM	mmu_circRNA_41378	chr7	-	46743993	46750514	intergenic	3.717	9.74998	9.23	3.98E-07	5.20E-05	NA
MCAO 24h v.s. SHAM	mmu_circRNA_20836	chr1	+	128252202	128269971	exonic	2.386	7.37815	3.565	0.00337643	0.015729	Ubxn4

MCAO 24h v.s. SHAM	mmu_circRNA_39371	chr5	+	123934071	123936555	exonic	3.283	9.61083	5.369	0.00012091	0.001539	Ccdc62
MCAO 24h v.s. SHAM	mmu_circRNA_37770	chr4	-	134148009	134172880	exonic	2.674	9.51011	4.615	0.00046458	0.003801	Cep85
MCAO 24h v.s. SHAM	mmu_circRNA_20054	chr1	-	52189422	52207359	exonic	3.31	7.59221	7.499	4.09E-06	0.000169	Gls
MCAO 24h v.s. SHAM	mmu_circRNA_001874	chr9	+	120453526	120464961	exonic	2.561	9.54261	5.188	0.00016594	0.001874	Myrip
MCAO 24h v.s. SHAM	mmu_circRNA_013002	chr16	+	94403313	94468060	sense overlapping	3.127	10.6294	6.757	1.24E-05	0.000336	Ttc3
MCAO 24h v.s. SHAM	mmu_circRNA_011696	chr17	+	39844601	39844697	sense overlapping	2.404	13.7386	4.38	0.00071769	0.005203	NA
MCAO 24h v.s. SHAM	mmu_circRNA_24446	chr11	+	117700724	117723550	exonic	-3.673	11.8964	-10.5	8.52E-08	3.91E-05	Tnrc6c
MCAO 24h v.s. SHAM	mmu_circRNA_24446	chr11	+	117700724	117723550	exonic	-3.673	11.8964	-10.5	8.52E-08	3.91E-05	Gm11734
MCAO 24h v.s. SHAM	mmu_circRNA_40913	chr6	+	122828156	122833876	exonic	3.079	12.9587	9.513	2.81E-07	5.03E-05	Foxj2
MCAO 24h v.s. SHAM	mmu_circRNA_39558	chr5	-	138150606	138152969	exonic	-2.202	7.52635	-7.3	5.44E-06	0.000199	Zfp113
MCAO 24h v.s. SHAM	mmu_circRNA_011703	chr15	+	39555064	39616510	sense overlapping	2.356	6.25351	2.962	0.01085035	0.036946	Rims2
MCAO 24h v.s. SHAM	mmu_circRNA_34392	chr2	-	120929385	120955720	exonic	2.323	8.40392	3.268	0.00599631	0.023902	Ubr1
MCAO 24h v.s. SHAM	mmu_circRNA_003170	chr2	+	18101457	18126229	exonic	3.256	11.6332	6.698	1.36E-05	0.000355	Mllt10
MCAO 24h v.s. SHAM	mmu_circRNA_002314	chr13	+	8672404	8731971	exonic	3.052	8.09707	5.014	0.00022601	0.002328	Adarb2
MCAO 24h v.s. SHAM	mmu_circRNA_29156	chr16	+	5234965	5239166	exonic	3.023	11.4155	6.964	9.04E-06	0.000274	Alg1
MCAO 24h v.s. SHAM	mmu_circRNA_015541	chr2	+	37508927	37541176	exonic	3.638	9.68183	5.255	0.00014746	0.001739	Rabgap1
MCAO 24h v.s. SHAM	mmu_circRNA_015541	chr2	+	37508927	37541176	exonic	3.638	9.68183	5.255	0.00014746	0.001739	Strbp
MCAO 24h v.s. SHAM	mmu_circRNA_42495	chr7	-	143584647	143588583	exonic	2.948	6.76258	4.273	0.00087582	0.006041	Cars
MCAO 24h v.s. SHAM	mmu_circRNA_38761	chr5	-	72353869	72360758	exonic	3.005	9.91956	5.671	7.20E-05	0.001064	Corin
MCAO 24h v.s. SHAM	mmu_circRNA_013794	chr4	+	152112107	152112700	exonic	3.523	8.21143	8.282	1.37E-06	8.93E-05	Plekhg5
MCAO 24h v.s. SHAM	mmu_circRNA_19080	chr12	+	104237731	104247974	intronic	2.166	7.82583	5.029	0.00022002	0.002294	Serpina3g

MCAO 24h v.s. SHAM	mmu_circRNA_34309	chr2	+	119701117	119705643	exonic	-2.176	6.00367	-6.22	2.89E-05	0.000581	Rtf1
MCAO 24h v.s. SHAM	mmu_circRNA_27565	chr14	-	55640384	55641588	exonic	4.306	10.9835	6.539	1.74E-05	0.000419	Tm9sf1
MCAO 24h v.s. SHAM	mmu_circRNA_29807	chr16	+	52047198	52047449	exonic	-2.201	7.87873	-6.83	1.12E-05	0.000314	Cblb
MCAO 24h v.s. SHAM	mmu_circRNA_27592	chr14	+	56887794	56919342	exonic	3.265	7.43915	5.488	9.84E-05	0.001337	Zmym2
MCAO 24h v.s. SHAM	mmu_circRNA_23299	chr11	-	50910435	50912404	exonic	2.464	7.77346	4.356	0.00075085	0.005391	Zfp2
MCAO 24h v.s. SHAM	mmu_circRNA_39090	chr5	-	106600275	106666845	sense overlapping	-2.077	7.9969	-4.6	0.00047533	0.003867	Zfp644
MCAO 24h v.s. SHAM	mmu_circRNA_43037	chr8	+	72725536	72731278	exonic	3.613	6.7959	7.514	4.00E-06	0.000167	Sin3b
MCAO 24h v.s. SHAM	mmu_circRNA_24114	chr11	-	95289771	95294645	exonic	-2.07	8.19932	-5.26	0.00014731	0.001739	Kat7
MCAO 24h v.s. SHAM	mmu_circRNA_19044	chr11	-	104368678	104378827	sense overlapping	-2.494	11.2665	-8.07	1.82E-06	0.000106	Kansl1
MCAO 24h v.s. SHAM	mmu_circRNA_31098	chr17	+	86559150	86559724	sense overlapping	3.574	8.31396	5.97	4.37E-05	0.000766	Prkce
MCAO 24h v.s. SHAM	mmu_circRNA_36263	chr3	-	144705512	144709361	exonic	-3.434	10.2896	-8.27	1.38E-06	8.93E-05	NA
MCAO 24h v.s. SHAM	mmu_circRNA_40285	chr6	-	52777534	52894071	exonic	-2.092	8.5611	-3.39	0.00475541	0.020287	Jazf1
MCAO 24h v.s. SHAM	mmu_circRNA_19186	chr17	-	33977770	33997565	sense overlapping	2.122	8.2004	6.255	2.74E-05	0.000559	H2-K1
MCAO 24h v.s. SHAM	mmu_circRNA_30283	chr17	+	20355630	20360447	exonic	2.357	6.15027	9.001	5.32E-07	5.84E-05	Vmn2r107
MCAO 24h v.s. SHAM	mmu_circRNA_34106	chr2	-	101758509	101772408	exonic	-2.113	11.5585	-4.75	0.00036081	0.003188	Prr5l
MCAO 24h v.s. SHAM	mmu_circRNA_23070	chr11	+	26407547	26434500	exonic	-2.172	9.35834	-4.83	0.00031423	0.002923	Fancl
MCAO 24h v.s. SHAM	mmu_circRNA_25585	chr12	+	88850278	88853112	intronic	2.654	12.3907	5.814	5.66E-05	0.000906	Nrxn3
MCAO 24h v.s. SHAM	mmu_circRNA_39113	chr5	-	107137529	107149958	exonic	2.446	10.2656	6.954	9.19E-06	0.000277	Tgfbr3
MCAO 24h v.s. SHAM	mmu_circRNA_36559	chr4	+	24505344	24578903	exonic	-2.293	9.21446	-3.11	0.00815552	0.029932	Mms22l
MCAO 24h v.s. SHAM	mmu_circRNA_41329	chr7	-	37961387	37969697	sense overlapping	3.496	7.86328	5.014	0.00022603	0.002328	Uri1

MCAO 24h v.s. SHAM	mmu_circRNA_002563	chr18	+	22375132	22434675	exonic	2.451	11.7048	5.078	0.00020164	0.002153	Asxl3
MCAO 24h v.s. SHAM	mmu_circRNA_22776	chr10	-	127299948	127305857	exonic	2.494	7.28734	7.329	5.24E-06	0.000195	Mars
MCAO 24h v.s. SHAM	mmu_circRNA_29625	chr16	-	35422865	35449463	exonic	-2.133	14.0795	-6.28	2.63E-05	0.000546	Pdia5
MCAO 24h v.s. SHAM	mmu_circRNA_32658	chr19	+	44952068	44979229	exonic	-2.15	9.08455	-6.01	4.10E-05	0.000734	NA
MCAO 24h v.s. SHAM	mmu_circRNA_25573	chr12	+	86952743	86961903	sense overlapping	-2	10.3291	-4.87	0.00028978	0.002773	Cipc
MCAO 24h v.s. SHAM	mmu_circRNA_30337	chr17	-	25380224	25381573	exonic	2.193	9.03534	5.458	0.00010374	0.001393	Cacna1h
MCAO 24h v.s. SHAM	mmu_circRNA_38721	chr5	-	67398199	67400301	sense overlapping	-2.022	5.91948	-4.22	0.00096091	0.006399	Bend4
MCAO 24h v.s. SHAM	mmu_circRNA_36945	chr4	+	74187313	74187769	exonic	2.161	13.6928	8.271	1.39E-06	8.93E-05	Frmd3
MCAO 24h v.s. SHAM	mmu_circRNA_24544	chr12	+	3591905	3632964	exonic	-2.72	11.4848	-6.57	1.65E-05	0.000403	Dtnb
MCAO 24h v.s. SHAM	mmu_circRNA_25566	chr12	+	86253847	86256932	sense overlapping	-2.505	6.26677	-8.07	1.84E-06	0.000106	Gpatch2l
MCAO 24h v.s. SHAM	mmu_circRNA_27181	chr14	-	21476670	21483639	antisense	2.2	12.1141	4.655	0.00043191	0.003615	Gm30363
MCAO 24h v.s. SHAM	mmu_circRNA_27181	chr14	-	21476670	21483639	antisense	2.2	12.1141	4.655	0.00043191	0.003615	Kat6b
MCAO 24h v.s. SHAM	mmu_circRNA_34504	chr2	+	127237460	127238820	exonic	-3.403	10.8092	-7.79	2.70E-06	0.000137	Snrnp200
MCAO 24h v.s. SHAM	mmu_circRNA_24789	chr12	+	21230822	21247373	exonic	2.989	10.8049	6.84	1.09E-05	0.000308	Gm40855
MCAO 24h v.s. SHAM	mmu_circRNA_24789	chr12	+	21230822	21247373	exonic	2.989	10.8049	6.84	1.09E-05	0.000308	Asap2
MCAO 24h v.s. SHAM	mmu_circRNA_19387	chr5	-	124089585	124090263	sense overlapping	2.44	10.502	6.511	1.82E-05	0.000429	Abcb9
MCAO 24h v.s. SHAM	mmu_circRNA_39375	chr5	-	124089587	124090262	exonic	2.363	10.6066	6.51	1.83E-05	0.000429	Abcb9
MCAO 24h v.s. SHAM	mmu_circRNA_42048	chr7	+	105682778	105687385	exonic	2.649	12.6057	7.118	7.17E-06	0.000244	Dnhd1
MCAO 24h v.s. SHAM	mmu_circRNA_36666	chr4	+	40990792	40994180	exonic	-2.045	8.24512	-3.9	0.00178848	0.009948	Nfx1
MCAO 24h v.s. SHAM	mmu_circRNA_34777	chr2	+	152677541	152704941	sense overlapping	2.285	13.5676	5.435	0.00010784	0.00143	H13
MCAO 24h v.s. SHAM	mmu_circRNA_19180	chr17	+	15021913	15051640	sense overlapping	2.021	9.85052	4.598	0.00047995	0.003891	Ermard

MCAO 24h v.s. SHAM	mmu_circRNA_19180	chr17	+	15021913	15051640	sense overlapping	2.021	9.85052	4.598	0.00047995	0.003891	9030025P20Rik
MCAO 24h v.s. SHAM	mmu_circRNA_19180	chr17	+	15021913	15051640	sense overlapping	2.021	9.85052	4.598	0.00047995	0.003891	Gm3255
MCAO 24h v.s. SHAM	mmu_circRNA_008457	chr16	-	89877037	89885125	exonic	2.3	12.2258	6.429	2.08E-05	0.000465	Tiam1
MCAO 24h v.s. SHAM	mmu_circRNA_25839	chr12	+	109729766	109730488	sense overlapping	3.464	7.25567	8.132	1.68E-06	0.000101	Mirg
MCAO 24h v.s. SHAM	mmu_circRNA_35586	chr3	+	73269612	73341175	intronic	3.132	8.29045	6.61	1.56E-05	0.00039	Gm20754
MCAO 24h v.s. SHAM	mmu_circRNA_42423	chr7	+	133650500	133667691	exonic	3.549	11.0595	6.386	2.22E-05	0.000482	Edrf1
MCAO 24h v.s. SHAM	mmu_circRNA_32099	chr18	+	86883565	86911148	intergenic	-2.284	9.05156	-4.56	0.00051557	0.004092	NA
MCAO 24h v.s. SHAM	mmu_circRNA_36886	chr4	-	62329347	62336547	exonic	2.221	10.7924	4.183	0.00103828	0.00675	Fkbp15
MCAO 24h v.s. SHAM	mmu_circRNA_40203	chr6	+	40004144	40207533	sense overlapping	6.279	10.0608	10.16	1.29E-07	4.31E-05	Tmem178b
MCAO 24h v.s. SHAM	mmu_circRNA_013053	chr6	-	86710514	86711679	exonic	2.453	6.86638	4.717	0.00038567	0.003329	Gmcl1
MCAO 24h v.s. SHAM	mmu_circRNA_43956	chr9	+	34536915	34538449	intronic	7.462	9.26161	13.02	6.51E-09	2.24E-05	Kirrel3
MCAO 24h v.s. SHAM	mmu_circRNA_19677	chr1	-	13284813	13286025	intronic	3.49	8.44883	4.852	0.00030219	0.002851	Ncoa2
MCAO 24h v.s. SHAM	mmu_circRNA_31339	chr18	+	12976468	12988385	exonic	4.514	6.90663	6.634	1.50E-05	0.000383	Impact
MCAO 24h v.s. SHAM	mmu_circRNA_33499	chr2	-	48087107	48095756	sense overlapping	4.923	9.1881	7.211	6.24E-06	0.000219	Gm13481
MCAO 24h v.s. SHAM	mmu_circRNA_27547	chr14	+	51994879	51996407	exonic	2.912	6.47832	5.92	4.74E-05	0.000806	Arhgef40
MCAO 24h v.s. SHAM	mmu_circRNA_34968	chr2	-	166617832	166621333	exonic	4.335	11.8013	7.383	4.85E-06	0.000186	Prex1
MCAO 24h v.s. SHAM	mmu_circRNA_20613	chr1	-	105546663	105566250	exonic	3.045	8.66184	7.545	3.83E-06	0.000161	Pign
MCAO 24h v.s. SHAM	mmu_circRNA_20613	chr1	-	105546663	105566250	exonic	3.045	8.66184	7.545	3.83E-06	0.000161	Gm28403
MCAO 24h v.s. SHAM	mmu_circRNA_28615	chr15	-	55749187	55792250	exonic	-2.338	7.26177	-6.41	2.15E-05	0.000473	Sntb1
MCAO 24h v.s. SHAM	mmu_circRNA_19077	chr12	+	88455628	88456716	intronic	5.785	6.53	8.069	1.83E-06	0.000106	Adck1

MCAO 24h v.s. SHAM	mmu_circRNA_000614	chr15	-	35088387	35115645	sense overlapping	2.476	8.16913	6.268	2.69E-05	0.000553	Stk3
MCAO 24h v.s. SHAM	mmu_circRNA_27543	chr14	+	49230063	49237629	antisense	2.276	6.57241	8.264	1.40E-06	8.93E-05	1700011H14Rik
MCAO 24h v.s. SHAM	mmu_circRNA_41052	chr6	-	144155096	144625686	exonic	2.909	10.6932	7.741	2.90E-06	0.000141	NA
MCAO 24h v.s. SHAM	mmu_circRNA_19469	chr7	+	102563267	102563363	antisense	2.097	12.3503	6.375	2.26E-05	0.000488	Trim21
MCAO 24h v.s. SHAM	mmu_circRNA_19608	chr1	-	8624778	8682029	exonic	2.006	6.16824	3.119	0.00800813	0.029538	Sntg1
MCAO 24h v.s. SHAM	mmu_circRNA_45753	chrX	-	139954906	139968599	exonic	2.065	11.6766	6.441	2.04E-05	0.00046	Rbm41
MCAO 24h v.s. SHAM	mmu_circRNA_19470	chr7	-	104315409	104348404	sense overlapping	3.638	12.3217	7.298	5.49E-06	0.0002	Trim5
MCAO 24h v.s. SHAM	mmu_circRNA_31499	chr18	+	32070106	32080577	exonic	-2.017	6.71854	-3.81	0.00208876	0.011081	Iws1
MCAO 24h v.s. SHAM	mmu_circRNA_36544	chr4	+	22025119	22040595	intergenic	3.725	7.03666	7.474	4.25E-06	0.000171	Gm42260
MCAO 24h v.s. SHAM	mmu_circRNA_24051	chr11	-	88001831	88003875	sense overlapping	3.189	9.61851	9.134	4.49E-07	5.38E-05	LOC108168720
MCAO 24h v.s. SHAM	mmu_circRNA_24051	chr11	-	88001831	88003875	sense overlapping	3.189	9.61851	9.134	4.49E-07	5.38E-05	LOC105247666
MCAO 24h v.s. SHAM	mmu_circRNA_23895	chr11	+	83987912	84009550	exonic	2.032	7.03068	6.302	2.54E-05	0.000535	Synrg
MCAO 24h v.s. SHAM	mmu_circRNA_22210	chr10	+	74324357	74396479	exonic	2.59	7.4128	6.456	1.99E-05	0.000453	Pcdh15
MCAO 24h v.s. SHAM	mmu_circRNA_39830	chr6	-	8643972	8658314	exonic	3.437	7.55356	9.183	4.23E-07	5.26E-05	Ica1
MCAO 24h v.s. SHAM	mmu_circRNA_20370	chr1	-	77398493	77426889	exonic	2.031	9.75193	6.107	3.49E-05	0.000657	Epha4
MCAO 24h v.s. SHAM	mmu_circRNA_32047	chr18	-	80808611	80891923	exonic	3.112	12.7347	7.638	3.35E-06	0.000151	Atp9b
MCAO 24h v.s. SHAM	mmu_circRNA_28307	chr15	-	25027225	25364918	sense overlapping	3.573	10.2107	5.935	4.63E-05	0.000797	Gm2824
MCAO 24h v.s. SHAM	mmu_circRNA_19502	chr8	+	122305354	122307643	intronic	-2.007	8.57794	-3.72	0.00252574	0.01277	Zfpm1
MCAO 24h v.s. SHAM	mmu_circRNA_42386	chr7	+	130739811	130753661	exonic	-2.6	7.40021	-3.24	0.00629593	0.024809	Tacc2
MCAO 24h v.s. SHAM	mmu_circRNA_43907	chr9	+	28039050	28045364	intronic	4.307	11.0118	9.238	3.95E-07	5.20E-05	Opcml
MCAO 24h v.s. SHAM	mmu_circRNA_32399	chr19	-	28283651	28317413	exonic	2.146	6.18751	5.912	4.81E-05	0.000816	Glis3

MCAO 24h v.s. SHAM	mmu_circRNA_21095	chr1	-	157230038	157299264	exonic	2.925	8.53058	7.052	7.92E-06	0.000258	Rasal2
MCAO 24h v.s. SHAM	mmu_circRNA_19930	chr1	+	37035636	37063549	exonic	3.045	7.41129	9.449	3.03E-07	5.05E-05	Vwa3b
MCAO 24h v.s. SHAM	mmu_circRNA_25708	chr12	-	101043442	101049818	exonic	-2.129	9.0035	-5.72	6.61E-05	0.001007	Ppp4r3a
MCAO 24h v.s. SHAM	mmu_circRNA_33553	chr2	+	52105434	52120368	exonic	-2.02	6.04643	-4.23	0.00094772	0.006341	Rif1
MCAO 24h v.s. SHAM	mmu_circRNA_31163	chr17	-	90991378	90992144	intronic	3.856	7.0874	3.151	0.00752139	0.028329	Nrxn1
MCAO 24h v.s. SHAM	mmu_circRNA_19351	chr4	-	151830417	151835885	sense overlapping	-2.59	12.4454	-9.88	1.81E-07	4.46E-05	Camta1
MCAO 24h v.s. SHAM	mmu_circRNA_30261	chr17	+	14336546	14348778	exonic	-2.299	7.94725	-3.65	0.00288757	0.014115	Smoc2
MCAO 24h v.s. SHAM	mmu_circRNA_28060	chr14	-	121297134	121308071	exonic	-2.115	6.30441	-4.54	0.00053688	0.004202	Stk24
MCAO 24h v.s. SHAM	mmu_circRNA_19472	chr7	+	108167494	108184865	antisense	2.143	5.73533	8.404	1.16E-06	8.32E-05	Gm39066
MCAO 24h v.s. SHAM	mmu_circRNA_37176	chr4	+	95696959	95769529	exonic	3.428	10.5223	6.228	2.86E-05	0.000578	Fggy
MCAO 24h v.s. SHAM	mmu_circRNA_23788	chr11	+	78283058	78284282	exonic	-3.037	7.11824	-8.31	1.31E-06	8.92E-05	2610507B11Rik
MCAO 24h v.s. SHAM	mmu_circRNA_41355	chr7	+	45575430	45588528	exonic	2.348	6.8683	6.503	1.85E-05	0.000431	Bcat2
MCAO 24h v.s. SHAM	mmu_circRNA_44596	chr9	-	71917004	72006762	exonic	2.849	8.00539	6.814	1.14E-05	0.000318	Tcf12
MCAO 24h v.s. SHAM	mmu_circRNA_003023	chr3	-	51308049	51326032	sense overlapping	-3.336	12.2892	-9.94	1.68E-07	4.38E-05	Elf2
MCAO 24h v.s. SHAM	mmu_circRNA_40266	chr6	+	51562017	51588428	exonic	-2.646	6.35362	-6.14	3.29E-05	0.000629	Snx10
MCAO 24h v.s. SHAM	mmu_circRNA_31992	chr18	+	75894633	75900761	sense overlapping	-2.055	10.9458	-3.76	0.00230622	0.011923	Zbtb7c
MCAO 24h v.s. SHAM	mmu_circRNA_26920	chr13	-	106812207	106886757	exonic	-2.093	9.6158	-4.22	0.00096164	0.006399	Ipo11
MCAO 24h v.s. SHAM	mmu_circRNA_001009	chr19	-	4784723	4794009	sense overlapping	-2.134	11.2561	-5.12	0.00018543	0.002024	Gm21992
MCAO 24h v.s. SHAM	mmu_circRNA_001009	chr19	-	4784723	4794009	sense overlapping	-2.134	11.2561	-5.12	0.00018543	0.002024	Rbm4
MCAO 24h v.s. SHAM	mmu_circRNA_45837	chrX	+	152033698	152047968	exonic	2.799	10.4481	7.93	2.22E-06	0.000121	Smc1a

MCAO 24h v.s. SHAM	mmu_circRNA_38346	chr5	-	29287390	29363955	exonic	2.494	6.81944	7.599	3.55E-06	0.000154	Lmbr1
MCAO 24h v.s. SHAM	mmu_circRNA_19255	chr2	+	104608871	104609187	sense overlapping	-2.253	11.5541	-6.8	1.16E-05	0.000322	Cstf3
MCAO 24h v.s. SHAM	mmu_circRNA_40039	chr6	-	32892291	32968318	exonic	-2.016	6.68128	-7.21	6.26E-06	0.000219	Chchd3
MCAO 24h v.s. SHAM	mmu_circRNA_36353	chr3	+	157960983	157962382	exonic	-2.222	9.79016	-4.71	0.00039381	0.003374	Ankrd13c
MCAO 24h v.s. SHAM	mmu_circRNA_26486	chr13	+	63156545	63240331	exonic	4.655	8.743	6.617	1.55E-05	0.000389	2010111I01Rik
MCAO 24h v.s. SHAM	mmu_circRNA_30333	chr17	+	25167296	25168665	exonic	-2.259	9.46242	-4.47	0.0006101	0.004614	Ccdc154
MCAO 24h v.s. SHAM	mmu_circRNA_37263	chr4	+	102760442	102870724	exonic	2.217	8.74199	7.017	8.35E-06	0.000264	Sgip1
MCAO 24h v.s. SHAM	mmu_circRNA_000673	chr2	+	31778333	31779216	exonic	2.28	7.32182	6.109	3.48E-05	0.000657	Abl1
MCAO 24h v.s. SHAM	mmu_circRNA_22800	chr10	-	128578099	128579092	exonic	-2.241	11.4194	-4.84	0.00030624	0.002869	Erbb3
MCAO 24h v.s. SHAM	mmu_circRNA_013935	chr19	-	4784723	4785567	intronic	-2.006	11.1684	-4.36	0.00075019	0.00539	Gm21992
MCAO 24h v.s. SHAM	mmu_circRNA_013935	chr19	-	4784723	4785567	intronic	-2.006	11.1684	-4.36	0.00075019	0.00539	Rbm4
MCAO 24h v.s. SHAM	mmu_circRNA_21656	chr10	+	11289519	11291572	exonic	-2.889	9.68067	-7.03	8.22E-06	0.000261	Fbxo30
MCAO 24h v.s. SHAM	mmu_circRNA_001219	chr3	+	28532887	28542098	exonic	-2.025	10.3558	-5.15	0.00017841	0.001962	Tnik
MCAO 24h v.s. SHAM	mmu_circRNA_44888	chr9	-	96576285	96602807	exonic	-2.257	8.0109	-6.05	3.82E-05	0.000698	Rasa2
MCAO 24h v.s. SHAM	mmu_circRNA_44888	chr9	-	96576285	96602807	exonic	-2.257	8.0109	-6.05	3.82E-05	0.000698	LOC108167675
MCAO 24h v.s. SHAM	mmu_circRNA_30386	chr17	-	27596723	27596834	exonic	-2.493	7.11418	-3.79	0.00217838	0.011413	Nudt3
MCAO 24h v.s. SHAM	mmu_circRNA_42040	chr7	+	102354500	102421530	exonic	-2.493	6.90883	-5.91	4.85E-05	0.000819	Stim1
MCAO 24h v.s. SHAM	mmu_circRNA_40806	chr6	+	116313844	116315642	exonic	-2.275	7.4558	-6.22	2.92E-05	0.000585	Zfand4
MCAO 24h v.s. SHAM	mmu_circRNA_38907	chr5	+	86891227	86905024	antisense	2.307	13.7169	7.895	2.33E-06	0.000125	Ugt2b34
MCAO 24h v.s. SHAM	mmu_circRNA_24227	chr11	+	104153520	104163889	exonic	3.141	9.24502	7.211	6.25E-06	0.000219	Crhr1
MCAO 24h v.s. SHAM	mmu_circRNA_29634	chr16	-	35844727	35853758	exonic	2.314	6.98088	3.861	0.00191374	0.01044	Parp14

MCAO 24h v.s. SHAM	mmu_circRNA_30420	chr17	+	29282059	29282891	exonic	3.236	8.54857	9.793	2.00E-07	4.51E-05	BC004004
MCAO 24h v.s. SHAM	mmu_circRNA_30419	chr17	+	29277856	29282891	exonic	4.271	7.19489	13.9	2.88E-09	1.49E-05	BC004004
MCAO 24h v.s. SHAM	mmu_circRNA_42243	chr7	+	120899367	120899546	exonic	-2.244	7.39012	-5.14	0.00018183	0.001993	Eef2k
MCAO 24h v.s. SHAM	mmu_circRNA_25314	chr12	+	72783682	72786789	exonic	-2.482	6.82624	-5.43	0.000109	0.001443	Ppm1a
MCAO 24h v.s. SHAM	mmu_circRNA_29553	chr16	+	32884123	32898470	exonic	2.87	7.95559	5.17	0.00017125	0.001905	Fyttd1
MCAO 24h v.s. SHAM	mmu_circRNA_21094	chr1	-	157192746	157299264	exonic	2.173	6.70972	7.193	6.42E-06	0.000224	Rasal2
MCAO 24h v.s. SHAM	mmu_circRNA_010637	chr8	+	110632029	110637033	exonic	-2.055	7.09314	-4.29	0.00084184	0.005887	Vac14
MCAO 24h v.s. SHAM	mmu_circRNA_45396	chrX	+	20429022	20502512	exonic	3.189	8.11075	7.582	3.63E-06	0.000156	Jade3
MCAO 24h v.s. SHAM	mmu_circRNA_27645	chr14	-	59771638	59860313	exonic	2.23	7.65353	7.422	4.58E-06	0.000178	Atp8a2
MCAO 24h v.s. SHAM	mmu_circRNA_38525	chr5	+	37278084	37278931	exonic	4.446	8.0808	8.291	1.35E-06	8.93E-05	Crmp1
MCAO 24h v.s. SHAM	mmu_circRNA_33115	chr2	+	22901280	22915528	sense overlapping	-2.308	7.58996	-6.89	1.01E-05	0.000291	Pdss1
MCAO 24h v.s. SHAM	mmu_circRNA_42572	chr8	-	13605861	13631892	exonic	4.745	11.1948	7.997	2.02E-06	0.000114	Rasa3
MCAO 24h v.s. SHAM	mmu_circRNA_40598	chr6	-	97323654	97329564	exonic	-2.297	9.04445	-5.66	7.34E-05	0.001077	Frmd4b
MCAO 24h v.s. SHAM	mmu_circRNA_42191	chr7	+	117548583	117593739	exonic	2.776	8.13329	5.324	0.00013078	0.001619	Xylt1
MCAO 24h v.s. SHAM	mmu_circRNA_44389	chr9	-	61924631	61926518	exonic	2.769	11.5111	6.938	9.42E-06	0.000279	Kif23
MCAO 24h v.s. SHAM	mmu_circRNA_40047	chr6	+	33296732	33442039	exonic	-2.046	11.0256	-4.81	0.00032378	0.002975	Exoc4
MCAO 24h v.s. SHAM	mmu_circRNA_19238	chr2	-	30110202	30111334	sense overlapping	-2.461	8.70043	-7.69	3.11E-06	0.000144	Zer1
MCAO 24h v.s. SHAM	mmu_circRNA_36129	chr3	-	126976050	127023390	exonic	2.726	7.38496	6.636	1.50E-05	0.000383	Ank2
MCAO 24h v.s. SHAM	mmu_circRNA_28251	chr15	+	12150172	12156408	exonic	-2.851	6.54151	-8.57	9.33E-07	7.61E-05	Zfr
MCAO 24h v.s. SHAM	mmu_circRNA_25527	chr12	-	85320713	85336485	exonic	-2.459	9.31009	-6.77	1.22E-05	0.000332	Nek9

MCAO 24h v.s. SHAM	mmu_circRNA_39331	chr5	+	122714398	122719226	exonic	-2.454	6.64769	-4.87	0.00029453	0.00281	P2rx4
MCAO 24h v.s. SHAM	mmu_circRNA_22421	chr10	+	90510595	90644556	exonic	4.587	8.96316	6.574	1.65E-05	0.000403	Anks1b
MCAO 24h v.s. SHAM	mmu_circRNA_43389	chr8	+	105178592	105202547	exonic	-2.889	8.21995	-8.48	1.04E-06	7.91E-05	Cbfb
MCAO 24h v.s. SHAM	mmu_circRNA_22066	chr10	+	58983920	59007137	exonic	2.712	11.2729	8.671	8.15E-07	7.43E-05	Sh3rf3
MCAO 24h v.s. SHAM	mmu_circRNA_35066	chr2	-	181081454	181114729	exonic	2.294	8.86149	6.901	9.96E-06	0.00029	Kcnq2
MCAO 24h v.s. SHAM	mmu_circRNA_23643	chr11	-	72456302	72474770	exonic	-4.25	8.98581	-6.67	1.43E-05	0.000371	Spns2
MCAO 24h v.s. SHAM	mmu_circRNA_23253	chr11	+	44531275	44551819	exonic	2.71	11.7915	7.106	7.31E-06	0.000246	Rnf145
MCAO 24h v.s. SHAM	mmu_circRNA_44128	chr9	-	49543010	49569900	exonic	2.364	7.20637	6.163	3.18E-05	0.000615	Ncam1
MCAO 24h v.s. SHAM	mmu_circRNA_44128	chr9	-	49543010	49569900	exonic	2.364	7.20637	6.163	3.18E-05	0.000615	Gm11149
MCAO 24h v.s. SHAM	mmu_circRNA_39121	chr5	-	107795698	107799260	exonic	-4.347	10.891	-10.2	1.17E-07	4.18E-05	Evi5
MCAO 24h v.s. SHAM	mmu_circRNA_22574	chr10	-	107563134	107608453	exonic	2.438	7.472	7.733	2.93E-06	0.000141	Gm36177
MCAO 24h v.s. SHAM	mmu_circRNA_22574	chr10	-	107563134	107608453	exonic	2.438	7.472	7.733	2.93E-06	0.000141	Ptprq
MCAO 24h v.s. SHAM	mmu_circRNA_19729	chr1	-	21424048	21469564	exonic	-2.697	8.21758	-4.72	0.00038687	0.003334	Kcnq5
MCAO 24h v.s. SHAM	mmu_circRNA_31064	chr17	-	85065631	85076825	exonic	3.061	6.80618	7.391	4.79E-06	0.000185	Prepl
MCAO 24h v.s. SHAM	mmu_circRNA_43320	chr8	-	91299228	91310429	exonic	4.147	8.47509	6.183	3.08E-05	0.000606	Rpgrip1l
MCAO 24h v.s. SHAM	mmu_circRNA_43320	chr8	-	91299228	91310429	exonic	4.147	8.47509	6.183	3.08E-05	0.000606	Gm36163
MCAO 24h v.s. SHAM	mmu_circRNA_001832	chr6	-	82730767	82739674	exonic	3.986	12.3907	8.732	7.53E-07	7.00E-05	Hk2
MCAO 24h v.s. SHAM	mmu_circRNA_32153	chr19	-	3602234	3605494	exonic	2.162	10.6637	4.412	0.00067601	0.004985	Lrp5
MCAO 24h v.s. SHAM	mmu_circRNA_009370	chr17	+	39844697	39845062	sense overlapping	-2.35	12.5074	-3.09	0.00842264	0.030629	NA
MCAO 24h v.s. SHAM	mmu_circRNA_20530	chr1	+	91088633	91112297	exonic	2.585	9.31812	7.621	3.43E-06	0.000152	Lrrfip1
MCAO 24h v.s. SHAM	mmu_circRNA_41232	chr7	-	27174421	27176238	exonic	2.211	7.84351	8.343	1.26E-06	8.67E-05	Rab4b

MCAO 24h v.s. SHAM	mmu_circRNA_37286	chr4	+	103754180	103764967	intronic	6.789	8.22307	9.372	3.34E-07	5.15E-05	Dab1
MCAO 24h v.s. SHAM	mmu_circRNA_32308	chr19	+	21797326	21818011	exonic	2.03	10.4919	6.164	3.18E-05	0.000615	Tmem2
MCAO 24h v.s. SHAM	mmu_circRNA_005949	chr1	-	190885333	190899571	exonic	-2.25	10.5752	-5.95	4.53E-05	0.000787	Rps6kc1
MCAO 24h v.s. SHAM	mmu_circRNA_004682	chr18	-	61140790	61141790	exonic	-3.44	9.3288	-6.63	1.50E-05	0.000383	Hmgxb3
MCAO 24h v.s. SHAM	mmu_circRNA_29140	chr16	-	4417852	4419588	exonic	-2.628	7.29825	-4.83	0.00031237	0.002916	Adcy9
MCAO 24h v.s. SHAM	mmu_circRNA_20259	chr1	-	64034531	64042437	sense overlapping	3.462	10.5876	7.736	2.91E-06	0.000141	Klf7
MCAO 24h v.s. SHAM	mmu_circRNA_003057	chr18	+	11923359	11926364	exonic	3.419	9.66942	10.22	1.22E-07	4.19E-05	Cables1
MCAO 24h v.s. SHAM	mmu_circRNA_23281	chr11	+	49818579	49819254	exonic	4.772	6.99354	6.614	1.55E-05	0.000389	Gm36507
MCAO 24h v.s. SHAM	mmu_circRNA_23281	chr11	+	49818579	49819254	exonic	4.772	6.99354	6.614	1.55E-05	0.000389	Gfpt2
MCAO 24h v.s. SHAM	mmu_circRNA_26660	chr13	-	91022569	91040988	exonic	-2.236	7.40292	-4.82	0.00031786	0.002941	Atg10
MCAO 24h v.s. SHAM	mmu_circRNA_001643	chr11	+	72712156	72714483	exonic	-2.161	7.72653	-4.66	0.00042706	0.003587	Ankfy1
MCAO 24h v.s. SHAM	mmu_circRNA_19311	chr3	-	153411461	153411874	sense overlapping	-2.232	11.059	-6.06	3.76E-05	0.000693	St6galnac3
MCAO 24h v.s. SHAM	mmu_circRNA_45921	chrX	+	160523271	160533077	exonic	-4.418	12.4019	-8.48	1.05E-06	7.93E-05	Phka2
MCAO 24h v.s. SHAM	mmu_circRNA_19441	chr6	+	134062060	134066465	intronic	-2.895	11.7909	-7.71	3.05E-06	0.000142	Etv6
MCAO 24h v.s. SHAM	mmu_circRNA_38278	chr5	-	24866078	24889188	exonic	-2.457	8.14273	-5.61	7.98E-05	0.001152	Prkag2
MCAO 24h v.s. SHAM	mmu_circRNA_33594	chr2	-	58500483	58537873	exonic	2.031	12.2897	7.003	8.53E-06	0.000267	Acvr1
MCAO 24h v.s. SHAM	mmu_circRNA_002280	chr19	-	60764031	60764233	exonic	-3.018	10.7955	-7.44	4.49E-06	0.000177	Eif3a
MCAO 24h v.s. SHAM	mmu_circRNA_34709	chr2	-	144259056	144260835	exonic	-2.042	8.47027	-4.71	0.00038915	0.003344	Snx5
MCAO 24h v.s. SHAM	mmu_circRNA_45731	chrX	+	129902899	129972609	exonic	3.248	7.41611	6.767	1.22E-05	0.000332	Diaph2
MCAO 24h v.s. SHAM	mmu_circRNA_45047	chr9	+	106889019	106889196	antisense	5.586	8.9381	8.949	5.68E-07	6.05E-05	Manf

MCAO 24h v.s. SHAM	mmu_circRNA_30480	chr17	-	33658387	33677440	exonic	-2.515	7.68585	-6.45	2.01E-05	0.000455	Hnrnpm
MCAO 24h v.s. SHAM	mmu_circRNA_42655	chr8	-	24687881	24697228	exonic	-2.033	9.31941	-4.12	0.00117517	0.0073	Adam3
MCAO 24h v.s. SHAM	mmu_circRNA_23947	chr11	+	85326863	85339796	exonic	2.418	10.0317	4.393	0.00070004	0.005093	Ppm1d
MCAO 24h v.s. SHAM	mmu_circRNA_20456	chr1	+	86086029	86090190	exonic	3.453	9.70433	9.173	4.28E-07	5.26E-05	Psmd1
MCAO 24h v.s. SHAM	mmu_circRNA_32211	chr19	-	7094329	7097229	sense overlapping	2.892	7.08832	9.956	1.65E-07	4.38E-05	Macrod1
MCAO 24h v.s. SHAM	mmu_circRNA_32211	chr19	-	7094329	7097229	sense overlapping	2.892	7.08832	9.956	1.65E-07	4.38E-05	Flrt1
MCAO 24h v.s. SHAM	mmu_circRNA_015637	chr1	-	156900585	156907778	exonic	-2.169	11.7772	-6.1	3.54E-05	0.00066	Ralgps2
MCAO 24h v.s. SHAM	mmu_circRNA_002466	chr7	-	133025712	133081860	exonic	-2.059	7.19414	-5.85	5.38E-05	0.000877	Ctbp2
MCAO 24h v.s. SHAM	mmu_circRNA_40539	chr6	+	87904087	87905267	exonic	-3.204	6.6404	-5.1	0.00019372	0.002092	Copg1
MCAO 24h v.s. SHAM	mmu_circRNA_013298	chr17	+	74668758	74670382	exonic	-2.964	10.652	-7.96	2.14E-06	0.000117	Birc6
MCAO 24h v.s. SHAM	mmu_circRNA_24983	chr12	+	40626336	40710901	exonic	3.104	6.23783	6.069	3.71E-05	0.000687	Dock4
MCAO 24h v.s. SHAM	mmu_circRNA_37675	chr4	+	128393489	128419598	exonic	2.353	6.84072	6.878	1.03E-05	0.000297	Csmd2
MCAO 24h v.s. SHAM	mmu_circRNA_007649	chr14	-	52161680	52184020	sense overlapping	-2.321	9.00605	-4.06	0.0013075	0.007876	Supt16
MCAO 24h v.s. SHAM	mmu_circRNA_27348	chr14	-	30045214	30047371	exonic	-3.68	9.68138	-4.66	0.00042588	0.003586	Cacna1d
MCAO 24h v.s. SHAM	mmu_circRNA_31588	chr18	+	35571842	35588453	exonic	-2.249	9.7815	-4.78	0.00034117	0.003087	Matr3
MCAO 24h v.s. SHAM	mmu_circRNA_23083	chr11	+	29182446	29197209	exonic	-2.444	8.04744	-5.22	0.00015612	0.001805	Ppp4r3b
MCAO 24h v.s. SHAM	mmu_circRNA_22301	chr10	+	82709008	82724396	exonic	-2.42	7.89281	-5.64	7.55E-05	0.001105	Hcfc2
MCAO 24h v.s. SHAM	mmu_circRNA_22970	chr11	-	22137837	22151892	exonic	4.435	9.42401	5.707	6.78E-05	0.001025	Ehbp1
MCAO 24h v.s. SHAM	mmu_circRNA_37158	chr4	-	94575165	94584002	exonic	-3.111	7.01303	-7.32	5.30E-06	0.000197	Plaa
MCAO 24h v.s. SHAM	mmu_circRNA_26317	chr13	+	49828935	49830616	intergenic	2.725	7.87637	4.387	0.00070797	0.005144	LOC102631805
MCAO 24h v.s. SHAM	mmu_circRNA_40898	chr6	+	120402505	120428377	exonic	4.234	6.54009	6.911	9.81E-06	0.000287	Kdm5a

MCAO 24h v.s. SHAM	mmu_circRNA_21073	chr1	-	156365615	156382477	exonic	-2.495	10.8483	-4.66	0.00042691	0.003587	Axdnd1
MCAO 24h v.s. SHAM	mmu_circRNA_35735	chr3	-	88447990	88450112	exonic	2.701	8.73501	3.338	0.0052414	0.021779	Sema4a
MCAO 24h v.s. SHAM	mmu_circRNA_43789	chr9	-	15297842	15306619	sense overlapping	3.265	6.82082	7.846	2.50E-06	0.00013	4931406C07Rik
MCAO 24h v.s. SHAM	mmu_circRNA_24141	chr11	-	97240928	97267679	exonic	-2.149	7.88897	-5.99	4.25E-05	0.000751	Npepps
MCAO 24h v.s. SHAM	mmu_circRNA_36483	chr4	-	12057315	12063830	exonic	2.863	13.9213	5.873	5.13E-05	0.000851	Tmem67
MCAO 24h v.s. SHAM	mmu_circRNA_23142	chr11	-	30219578	30219772	exonic	-2.631	7.8774	-3.64	0.00290183	0.014158	Sptbn1
MCAO 24h v.s. SHAM	mmu_circRNA_23334	chr11	-	53262263	53271079	exonic	2.966	8.51314	3.136	0.00774978	0.028905	Hspa4
MCAO 24h v.s. SHAM	mmu_circRNA_23275	chr11	+	49260570	49261813	sense overlapping	2.007	6.93632	6.851	1.07E-05	0.000304	Mgat1
MCAO 24h v.s. SHAM	mmu_circRNA_006731	chr15	+	76904375	76904450	sense overlapping	-2.739	6.87196	-8.41	1.16E-06	8.32E-05	Rpl8
MCAO 24h v.s. SHAM	mmu_circRNA_37699	chr4	-	129712103	129730325	exonic	-4.294	12.3654	-5.47	0.00010082	0.001362	Khdrbs1
MCAO 24h v.s. SHAM	mmu_circRNA_32678	chr19	-	45757904	45777017	exonic	2.244	6.84811	7.046	7.99E-06	0.000259	Mgea5
MCAO 24h v.s. SHAM	mmu_circRNA_45217	chr9	-	114610644	114622962	exonic	2.817	6.80411	9.331	3.51E-07	5.17E-05	Cnot10
MCAO 24h v.s. SHAM	mmu_circRNA_36037	chr3	+	118781835	118999385	exonic	4.279	8.9245	6.394	2.19E-05	0.000479	Dpyd
MCAO 24h v.s. SHAM	mmu_circRNA_18935	chr1	-	52708163	52709758	sense overlapping	2.29	8.5999	3.4	0.00464428	0.019958	Mfsd6
MCAO 24h v.s. SHAM	mmu_circRNA_010777	chr14	-	23494538	23509900	exonic	5.3	9.39607	9.957	1.64E-07	4.38E-05	Kcnma1
MCAO 24h v.s. SHAM	mmu_circRNA_41390	chr7	+	48799828	48818720	exonic	3.155	11.2406	9.359	3.39E-07	5.15E-05	Zdhhc13
MCAO 24h v.s. SHAM	mmu_circRNA_23573	chr11	+	68011314	68020998	exonic	-2.259	6.66079	-7.82	2.60E-06	0.000134	Stx8
MCAO 24h v.s. SHAM	mmu_circRNA_22124	chr10	-	63016079	63019527	exonic	-2.351	7.1404	-5.52	9.25E-05	0.001288	Hnrnph3
MCAO 24h v.s. SHAM	mmu_circRNA_22124	chr10	-	63016079	63019527	exonic	-2.351	7.1404	-5.52	9.25E-05	0.001288	Rufy2
MCAO 24h v.s. SHAM	mmu_circRNA_39080	chr5	-	104085009	104094767	exonic	2.636	7.17418	4.311	0.00081633	0.005741	Sparcl1

MCAO 24h v.s. SHAM	mmu_circRNA_34179	chr2	+	106699287	106744881	exonic	-2.252	8.84819	-3.59	0.00323087	0.015273	Mpped2
MCAO 24h v.s. SHAM	mmu_circRNA_24038	chr11	-	87388247	87397757	exonic	-3.183	6.88192	-6.88	1.02E-05	0.000295	Rad51c
MCAO 24h v.s. SHAM	mmu_circRNA_018929	chr5	-	67729791	67751085	exonic	-2.142	7.73358	-7.05	7.93E-06	0.000258	Atp8a1
MCAO 24h v.s. SHAM	mmu_circRNA_20395	chr1	-	80290081	80323056	exonic	-2.735	9.39239	-6.18	3.11E-05	0.000609	Cul3
MCAO 24h v.s. SHAM	mmu_circRNA_33674	chr2	+	62158205	62257605	exonic	3.191	9.27946	8.283	1.37E-06	8.93E-05	Slc4a10
MCAO 24h v.s. SHAM	mmu_circRNA_33961	chr2	-	77719450	77720699	exonic	-2.472	7.80619	-4.02	0.00141326	0.008371	Zfp385b
MCAO 24h v.s. SHAM	mmu_circRNA_43608	chr8	-	124694829	124714283	exonic	2.256	8.66781	5.425	0.00010975	0.001451	Ttc13
MCAO 24h v.s. SHAM	mmu_circRNA_007853	chr10	+	14004200	14086082	sense overlapping	-3.055	12.3794	-6.42	2.10E-05	0.000465	Hivep2
MCAO 24h v.s. SHAM	mmu_circRNA_33862	chr2	-	73373614	73374014	exonic	3.393	9.08106	5.289	0.00013898	0.00168	Gpr155
MCAO 24h v.s. SHAM	mmu_circRNA_33862	chr2	-	73373614	73374014	exonic	3.393	9.08106	5.289	0.00013898	0.00168	Gm13709
MCAO 24h v.s. SHAM	mmu_circRNA_19052	chr12	+	3442471	3457211	sense overlapping	-2.605	7.86494	-6.56	1.68E-05	0.000408	Asxl2
MCAO 24h v.s. SHAM	mmu_circRNA_31462	chr18	-	25486793	25486965	exonic	-2.743	8.13496	-5.72	6.58E-05	0.001005	Celf4
MCAO 24h v.s. SHAM	mmu_circRNA_44487	chr9	-	65858245	65881060	exonic	2.277	9.42551	5.517	9.37E-05	0.001293	Trip4
MCAO 24h v.s. SHAM	mmu_circRNA_010789	chr15	-	96609855	96624169	exonic	3.126	11.0649	8.52	9.95E-07	7.81E-05	Slc38a1
MCAO 24h v.s. SHAM	mmu_circRNA_30673	chr17	+	57419321	57450101	exonic	3.339	7.19761	7.773	2.77E-06	0.000137	Adgre1
MCAO 24h v.s. SHAM	mmu_circRNA_37524	chr4	+	119872036	119872942	intronic	2.105	11.0006	6.452	2.00E-05	0.000455	Hivep3
MCAO 24h v.s. SHAM	mmu_circRNA_32230	chr19	+	8873513	8873846	exonic	-2.613	8.22412	-7.24	5.98E-06	0.000213	Ubxn1
MCAO 24h v.s. SHAM	mmu_circRNA_41526	chr7	-	61276438	61282828	intronic	3.545	9.4282	7.092	7.46E-06	0.000248	Gm31445
MCAO 24h v.s. SHAM	mmu_circRNA_41526	chr7	-	61276438	61282828	intronic	3.545	9.4282	7.092	7.46E-06	0.000248	A230006K03Rik
MCAO 24h v.s. SHAM	mmu_circRNA_33702	chr2	-	65482150	65495396	exonic	2.057	6.63896	7.318	5.33E-06	0.000197	Scn3a
MCAO 24h v.s. SHAM	mmu_circRNA_36472	chr4	-	11338721	11353463	exonic	-2.127	6.53488	-4.98	0.00023974	0.002427	Esrp1

MCAO 24h v.s. SHAM	mmu_circRNA_18974	chr10	+	40394322	40408366	intronic	-2.722	10.8707	-6.75	1.26E-05	0.000339	Cdk19
MCAO 24h v.s. SHAM	mmu_circRNA_35932	chr3	+	104455486	104456848	antisense	2.869	5.93977	7.707	3.04E-06	0.000142	Lrig2
MCAO 24h v.s. SHAM	mmu_circRNA_017685	chr16	+	5953418	5956863	intronic	3.025	11.6724	6.948	9.27E-06	0.000277	Rbfox1
MCAO 24h v.s. SHAM	mmu_circRNA_39229	chr5	+	117209553	117228079	exonic	-2.449	7.66799	-6.6	1.57E-05	0.000392	Taok3
MCAO 24h v.s. SHAM	mmu_circRNA_43010	chr8	-	70405258	70409011	exonic	2.139	10.4832	5.972	4.36E-05	0.000765	Crtc1
MCAO 24h v.s. SHAM	mmu_circRNA_002219	chr14	-	36918433	36921652	sense overlapping	-2.188	6.96671	-7.41	4.68E-06	0.000181	Ccser2
MCAO 24h v.s. SHAM	mmu_circRNA_25612	chr12	-	91266735	91348876	exonic	-2.445	7.00466	-5.92	4.72E-05	0.000805	Cep128
MCAO 24h v.s. SHAM	mmu_circRNA_20241	chr1	-	63078952	63093730	exonic	2.749	9.37306	3.663	0.00279307	0.01379	Ino80d
MCAO 24h v.s. SHAM	mmu_circRNA_39553	chr5	+	137795490	137801096	exonic	-2.557	7.97895	-3.28	0.00590651	0.023626	Zcwpw1
MCAO 24h v.s. SHAM	mmu_circRNA_22771	chr10	+	127092733	127092873	sense overlapping	3.208	11.4638	9.93	1.70E-07	4.38E-05	Agap2
MCAO 24h v.s. SHAM	mmu_circRNA_37852	chr4	-	140067136	140157573	exonic	-2.405	11.488	-7.22	6.20E-06	0.000219	Igsf21
MCAO 24h v.s. SHAM	mmu_circRNA_26826	chr13	-	98884514	98890790	exonic	3.029	10.3785	8.525	9.87E-07	7.81E-05	Kng2
MCAO 24h v.s. SHAM	mmu_circRNA_30482	chr17	+	34416466	34421987	exonic	3.025	12.0496	9.428	3.12E-07	5.11E-05	BC051142
MCAO 24h v.s. SHAM	mmu_circRNA_013924	chr4	-	136770955	136774644	exonic	-2.183	7.9129	-2.86	0.01332667	0.043198	Ephb2
MCAO 24h v.s. SHAM	mmu_circRNA_20290	chr1	-	66721804	66778108	exonic	-3.105	12.1603	-6.52	1.78E-05	0.000423	Kansl1l
MCAO 24h v.s. SHAM	mmu_circRNA_38185	chr5	-	20801901	20855201	exonic	2.276	7.96403	5.671	7.21E-05	0.001064	Phtf2
MCAO 24h v.s. SHAM	mmu_circRNA_19014	chr11	-	53891146	53969605	sense overlapping	2.369	7.83329	5.319	0.00013183	0.001626	Slc22a5
MCAO 24h v.s. SHAM	mmu_circRNA_37006	chr4	-	83260881	83271218	exonic	2.776	9.54519	5.963	4.42E-05	0.000773	Ttc39b
MCAO 24h v.s. SHAM	mmu_circRNA_38874	chr5	-	84067359	84156536	exonic	3.207	10.7494	7.618	3.45E-06	0.000152	Epha5
MCAO 24h v.s. SHAM	mmu_circRNA_36730	chr4	+	46167301	46170073	exonic	2.315	11.0736	5.884	5.04E-05	0.000838	Ncbp1

MCAO 24h v.s. SHAM	mmu_circRNA_36730	chr4	+	46167301	46170073	exonic	2.315	11.0736	5.884	5.04E-05	0.000838	Xpa
MCAO 24h v.s. SHAM	mmu_circRNA_25808	chr12	+	106042836	106062808	exonic	-2.147	8.58433	-5.7	6.87E-05	0.001035	Vrk1
MCAO 24h v.s. SHAM	mmu_circRNA_42057	chr7	+	109020516	109021029	exonic	-2.312	9.01553	-5.05	0.00021143	0.00223	Tub
MCAO 24h v.s. SHAM	mmu_circRNA_44966	chr9	+	101100570	101124015	sense overlapping	-2.19	10.7835	-4.92	0.00026832	0.002633	Ppp2r3a
MCAO 24h v.s. SHAM	mmu_circRNA_38627	chr5	+	53846200	53883694	exonic	2.081	8.10199	6.189	3.05E-05	0.000605	Tbc1d19
MCAO 24h v.s. SHAM	mmu_circRNA_44220	chr9	+	55397740	55400647	exonic	2.982	9.17902	7.082	7.57E-06	0.00025	Tmem266
MCAO 24h v.s. SHAM	mmu_circRNA_27768	chr14	+	70895771	70926072	exonic	2.028	7.55474	5.599	8.15E-05	0.001167	Gfra2
MCAO 24h v.s. SHAM	mmu_circRNA_28661	chr15	-	66156315	66161889	intronic	3.216	8.5502	7.887	2.36E-06	0.000125	Kcnq3
MCAO 24h v.s. SHAM	mmu_circRNA_25433	chr12	+	81946930	81967148	exonic	-2.964	7.14595	-4.82	0.00032043	0.002952	Pcnx
MCAO 24h v.s. SHAM	mmu_circRNA_21247	chr1	+	167830545	167832894	exonic	-2.703	7.25257	-5.2	0.00016256	0.001854	Lmx1a
MCAO 24h v.s. SHAM	mmu_circRNA_015506	chr17	+	78313123	78315746	exonic	-5.696	9.65211	-6.48	1.92E-05	0.000441	Crim1
MCAO 24h v.s. SHAM	mmu_circRNA_41878	chr7	-	92584465	92631662	exonic	-6.572	10.4762	-9.31	3.58E-07	5.17E-05	Ankrd42
MCAO 24h v.s. SHAM	mmu_circRNA_004941	chr13	-	64243633	64248499	sense overlapping	2.367	8.75373	7.029	8.21E-06	0.000261	Cdc14b
MCAO 24h v.s. SHAM	mmu_circRNA_36337	chr3	-	153411461	153509497	exonic	-3.115	8.97146	-8.54	9.71E-07	7.77E-05	St6galnac3
MCAO 24h v.s. SHAM	mmu_circRNA_32224	chr19	+	8749665	8750027	exonic	-2.422	10.1673	-5.31	0.00013503	0.001648	Stx5a
MCAO 24h v.s. SHAM	mmu_circRNA_014406	chr15	+	85359453	85365359	exonic	3.047	9.10438	5.61	7.99E-05	0.001152	Atxn10
MCAO 24h v.s. SHAM	mmu_circRNA_21750	chr10	+	21928341	21928836	sense overlapping	3.25	12.9902	7.496	4.11E-06	0.000169	Sgk1
MCAO 24h v.s. SHAM	mmu_circRNA_35996	chr3	+	109574105	109628167	exonic	-2.635	6.84934	-7.15	6.84E-06	0.000237	Vav3
MCAO 24h v.s. SHAM	mmu_circRNA_31039	chr17	-	83587656	83588379	sense overlapping	3.071	10.9519	6.6	1.59E-05	0.000393	Kcng3
MCAO 24h v.s. SHAM	mmu_circRNA_22664	chr10	+	116349965	116361307	exonic	2.14	7.27645	4.066	0.00129552	0.007812	Ptprb
MCAO 24h v.s. SHAM	mmu_circRNA_015839	chr16	-	73956487	73985712	exonic	2.267	5.96801	6.205	2.97E-05	0.000594	Robo2

MCAO 24h v.s. SHAM	mmu_circRNA_32300	chr19	-	20856674	20870488	exonic	-2.157	6.17278	-4.87	0.00029213	0.002793	Tmc1
MCAO 24h v.s. SHAM	mmu_circRNA_42683	chr8	+	25640576	25673434	exonic	-3.085	8.19164	-8.9	6.04E-07	6.23E-05	Nsd3
MCAO 24h v.s. SHAM	mmu_circRNA_20390	chr1	-	79796825	79821557	exonic	2.518	7.7575	8.793	6.95E-07	6.83E-05	Serpine2
MCAO 24h v.s. SHAM	mmu_circRNA_29357	chr16	+	17257334	17259073	sense overlapping	-2.002	9.29371	-5.01	0.00022884	0.002337	Hic2
MCAO 24h v.s. SHAM	mmu_circRNA_19780	chr1	+	24383303	24390303	antisense	3.913	6.42785	12.64	9.30E-09	2.40E-05	Col19a1
MCAO 24h v.s. SHAM	mmu_circRNA_30343	chr17	-	25841051	25841482	exonic	4.352	8.30124	11.33	3.52E-08	3.37E-05	Rhot2
MCAO 24h v.s. SHAM	mmu_circRNA_24936	chr12	-	36020489	36024072	exonic	2.188	8.25874	4.558	0.00051594	0.004092	Tspan13
MCAO 24h v.s. SHAM	mmu_circRNA_35559	chr3	+	69009483	69019325	exonic	2.047	6.57712	7.183	6.51E-06	0.000226	Smc4
MCAO 24h v.s. SHAM	mmu_circRNA_20093	chr1	-	54486469	54497627	exonic	2.387	8.6377	6.311	2.51E-05	0.000529	Pgap1
MCAO 24h v.s. SHAM	mmu_circRNA_30514	chr17	-	44551374	44555457	intronic	2.079	6.65745	6.533	1.76E-05	0.000422	Runx2
MCAO 24h v.s. SHAM	mmu_circRNA_36074	chr3	-	120797195	120799747	exonic	2.326	6.0088	6.791	1.18E-05	0.000325	6530403H02Rik
MCAO 24h v.s. SHAM	mmu_circRNA_29158	chr16	+	5952576	5956863	intronic	3.26	10.8036	7.714	3.01E-06	0.000142	Rbfox1
MCAO 24h v.s. SHAM	mmu_circRNA_23191	chr11	-	33283907	33452549	exonic	3.267	6.6821	6.398	2.18E-05	0.000478	Ranbp17
MCAO 24h v.s. SHAM	mmu_circRNA_21952	chr10	-	49355908	49523855	exonic	2.221	6.87361	6.058	3.78E-05	0.000694	Grik2
MCAO 24h v.s. SHAM	mmu_circRNA_004768	chr19	+	8874187	8874255	sense overlapping	3.176	9.4591	6.722	1.31E-05	0.000345	Ubxn1
MCAO 24h v.s. SHAM	mmu_circRNA_19355	chr5	+	3968576	3981215	sense overlapping	-3.306	8.0641	-5.35	0.00012555	0.001582	Akap9
MCAO 24h v.s. SHAM	mmu_circRNA_22867	chr11	+	5596713	5635387	exonic	-3.31	7.68831	-5.17	0.00017103	0.001905	Ankrd36
MCAO 24h v.s. SHAM	mmu_circRNA_007681	chr9	-	21757116	21757390	sense overlapping	-2.027	11.7998	-4.11	0.00119555	0.007393	Spc24
MCAO 24h v.s. SHAM	mmu_circRNA_006229	chr12	-	79083615	79085856	exonic	2.179	10.5818	6.715	1.33E-05	0.000347	Pigh
MCAO 24h v.s. SHAM	mmu_circRNA_018772	chr16	-	46436353	46438068	exonic	2.153	12.9806	7.082	7.58E-06	0.00025	Nectin3

MCAO 24h v.s. SHAM	mmu_circRNA_26004	chr13	-	10224644	10228621	sense overlapping	2.839	10.0926	7.352	5.07E-06	0.000192	Chrm3
MCAO 24h v.s. SHAM	mmu_circRNA_32779	chr19	-	56542632	56545646	exonic	2.696	6.6536	6.256	2.74E-05	0.000559	Dclre1a
MCAO 24h v.s. SHAM	mmu_circRNA_45692	chrX	+	106097389	106102846	exonic	3.964	7.42572	9.246	3.90E-07	5.20E-05	Atp7a
MCAO 24h v.s. SHAM	mmu_circRNA_41537	chr7	-	61813261	61916736	exonic	2.115	10.3586	5.701	6.85E-05	0.001034	A230057D06Rik
MCAO 24h v.s. SHAM	mmu_circRNA_39349	chr5	-	123590792	123642616	exonic	3.076	8.93705	7.598	3.55E-06	0.000154	Clip1
MCAO 24h v.s. SHAM	mmu_circRNA_32634	chr19	+	43810470	43814814	exonic	4.044	6.89627	8.441	1.10E-06	8.16E-05	Abcc2
MCAO 24h v.s. SHAM	mmu_circRNA_22146	chr10	-	66176233	66205600	antisense	2.213	9.6147	5.809	5.71E-05	0.000913	Gm31056
MCAO 24h v.s. SHAM	mmu_circRNA_22146	chr10	-	66176233	66205600	antisense	2.213	9.6147	5.809	5.71E-05	0.000913	4930407I19Rik
MCAO 24h v.s. SHAM	mmu_circRNA_21730	chr10	+	20158760	20159555	intronic	4.229	12.6282	7.717	2.99E-06	0.000142	Map7
MCAO 24h v.s. SHAM	mmu_circRNA_37893	chr4	-	145016734	145100067	exonic	-2.465	9.1335	-7.99	2.03E-06	0.000114	Vps13d
MCAO 24h v.s. SHAM	mmu_circRNA_41211	chr7	+	19368541	19368859	exonic	2.767	7.1614	8.678	8.07E-07	7.43E-05	Ppp1r13l
MCAO 24h v.s. SHAM	mmu_circRNA_22909	chr11	-	12306957	12309752	exonic	3.173	8.74306	9.413	3.17E-07	5.12E-05	Cobl
MCAO 24h v.s. SHAM	mmu_circRNA_44431	chr9	-	64149398	64153650	exonic	-2.06	10.8032	-3.02	0.00968764	0.033961	Zwilch
MCAO 24h v.s. SHAM	mmu_circRNA_31562	chr18	-	34614447	34616734	exonic	-2.477	7.18692	-6.19	3.03E-05	0.000603	Brd8
MCAO 24h v.s. SHAM	mmu_circRNA_42611	chr8	-	16346570	16501392	exonic	2.247	8.41874	5.9	4.90E-05	0.000823	Csmd1
MCAO 24h v.s. SHAM	mmu_circRNA_21761	chr10	-	25239625	25289730	exonic	-2.445	9.91409	-6.61	1.55E-05	0.000389	Akap7
MCAO 24h v.s. SHAM	mmu_circRNA_36929	chr4	-	70298819	70380301	exonic	-2.207	8.69443	-5.12	0.00018779	0.002039	Cdk5rap2
MCAO 24h v.s. SHAM	mmu_circRNA_42246	chr7	+	121032747	121057399	sense overlapping	3.9	11.8786	7.31	5.40E-06	0.000198	NA
MCAO 24h v.s. SHAM	mmu_circRNA_44758	chr9	-	86372340	86448853	exonic	2.077	9.30834	5.112	0.00018983	0.002059	Ube2cbp
MCAO 24h v.s. SHAM	mmu_circRNA_018441	chr1	+	163992593	163992680	antisense	4.033	6.37665	10.04	1.49E-07	4.38E-05	BC055324
MCAO 24h v.s. SHAM	mmu_circRNA_001686	chr6	+	61310813	61313835	exonic	2.086	13.6931	6.258	2.73E-05	0.000559	Ccser1

MCAO 24h v.s. SHAM	mmu_circRNA_29705	chr16	-	38498860	38518532	sense overlapping	-2.143	8.77763	-4.4	0.00069306	0.005075	Timmdc1
MCAO 24h v.s. SHAM	mmu_circRNA_28306	chr15	+	23436449	23460567	exonic	3.174	7.69871	4.954	0.00025125	0.002501	Cdh18
MCAO 24h v.s. SHAM	mmu_circRNA_21755	chr10	-	23138190	23159154	exonic	2.374	13.2622	5.771	6.09E-05	0.000953	Eya4
MCAO 24h v.s. SHAM	mmu_circRNA_40808	chr6	-	117702621	117702723	exonic	2.851	6.967	8.982	5.45E-07	5.92E-05	1700030F04Rik
MCAO 24h v.s. SHAM	mmu_circRNA_011784	chr8	-	77450692	77450923	exonic	-2.43	12.2097	-5.69	6.94E-05	0.001041	Arhgap10
MCAO 24h v.s. SHAM	mmu_circRNA_19073	chr12	+	81894979	81913476	sense overlapping	5.2	8.00463	11.33	3.53E-08	3.37E-05	Pcnx
MCAO 24h v.s. SHAM	mmu_circRNA_45629	chrX	-	98695998	98714211	exonic	3.604	10.5693	7.258	5.83E-06	0.00021	Ophn1
MCAO 24h v.s. SHAM	mmu_circRNA_20164	chr1	-	59066149	59076722	exonic	3.835	8.62554	9.394	3.25E-07	5.13E-05	Als2cr11
MCAO 24h v.s. SHAM	mmu_circRNA_22031	chr10	-	56064473	56129177	exonic	3.348	6.07152	9.947	1.66E-07	4.38E-05	Tbc1d32
MCAO 24h v.s. SHAM	mmu_circRNA_29261	chr16	-	13603027	13648499	exonic	-2.703	10.275	-6.15	3.24E-05	0.000621	Parn
MCAO 24h v.s. SHAM	mmu_circRNA_24681	chr12	+	8238442	8275890	exonic	2.179	6.57156	4.26	0.00089759	0.006125	Ldah
MCAO 24h v.s. SHAM	mmu_circRNA_37285	chr4	+	103754180	103755895	intronic	2.651	9.65288	4.948	0.00025396	0.002523	Dab1
MCAO 24h v.s. SHAM	mmu_circRNA_20201	chr1	+	60181462	60206416	exonic	3.063	6.89202	7.588	3.60E-06	0.000156	Nbeal1
MCAO 24h v.s. SHAM	mmu_circRNA_23477	chr11	+	62283596	62285885	exonic	2.83	9.10737	4.33	0.00078851	0.005598	Ttc19
MCAO 24h v.s. SHAM	mmu_circRNA_22246	chr10	+	75966350	75986140	exonic	3.081	10.6644	5.927	4.69E-05	0.000802	Gm5134
MCAO 24h v.s. SHAM	mmu_circRNA_29893	chr16	-	63566578	63603633	exonic	2.266	6.88765	6.626	1.52E-05	0.000386	Epha3
MCAO 24h v.s. SHAM	mmu_circRNA_43344	chr8	-	94086655	94092622	exonic	-3.988	6.19788	-6.19	3.07E-05	0.000606	Bbs2
MCAO 24h v.s. SHAM	mmu_circRNA_27734	chr14	-	66187442	66190234	exonic	4.1	9.4989	11.36	3.42E-08	3.37E-05	Ptk2b
MCAO 24h v.s. SHAM	mmu_circRNA_23240	chr11	-	41912311	41920550	sense overlapping	-2.344	8.62501	-3.79	0.00218052	0.011416	Gabrg2
MCAO 24h v.s. SHAM	mmu_circRNA_23240	chr11	-	41912311	41920550	sense overlapping	-2.344	8.62501	-3.79	0.00218052	0.011416	LOC102637814

MCAO 24h v.s. SHAM	mmu_circRNA_37352	chr4	-	108250494	108252216	exonic	2.231	9.00138	5.536	9.07E-05	0.001268	Zyg11b
MCAO 24h v.s. SHAM	mmu_circRNA_30556	chr17	-	46882732	46883949	sense overlapping	4.223	9.65751	10.54	8.40E-08	3.91E-05	NA
MCAO 24h v.s. SHAM	mmu_circRNA_42042	chr7	+	104571920	104588022	sense overlapping	-2.85	11.4005	-5.27	0.00014417	0.001723	NA
MCAO 24h v.s. SHAM	mmu_circRNA_36399	chr4	+	3589146	3598663	exonic	3.045	10.4637	4.671	0.00041922	0.003539	Tgs1
MCAO 24h v.s. SHAM	mmu_circRNA_25951	chr13	+	8559103	8570574	exonic	4.249	9.2851	8.49	1.03E-06	7.91E-05	Adarb2
MCAO 24h v.s. SHAM	mmu_circRNA_015986	chr8	-	77344639	77365160	exonic	-2.269	10.3786	-5.76	6.24E-05	0.000965	Arhgap10
MCAO 24h v.s. SHAM	mmu_circRNA_19084	chr13	+	13725811	13761203	sense overlapping	-2.292	5.71198	-4.28	0.00085744	0.005953	Lyst
MCAO 24h v.s. SHAM	mmu_circRNA_33227	chr2	-	29247827	29248878	sense overlapping	4.138	11.6832	7.649	3.30E-06	0.00015	6530402F18Rik
MCAO 24h v.s. SHAM	mmu_circRNA_37527	chr4	+	120447032	120478090	exonic	-2.148	8.27686	-5.24	0.00015173	0.001766	Scmh1
MCAO 24h v.s. SHAM	mmu_circRNA_44903	chr9	-	99101174	99122376	exonic	-3.077	7.25337	-5.83	5.47E-05	0.000888	Pik3cb
MCAO 24h v.s. SHAM	mmu_circRNA_40693	chr6	+	103700404	103706636	exonic	-2.332	8.98752	-4.38	0.00071706	0.005203	Chl1
MCAO 24h v.s. SHAM	mmu_circRNA_36755	chr4	-	48218080	48243549	exonic	2.079	12.5819	5.787	5.92E-05	0.000932	Erp44
MCAO 24h v.s. SHAM	mmu_circRNA_43615	chr8	+	125124492	125135509	exonic	-2.418	6.13057	-5.35	0.00012457	0.001576	Disc1
MCAO 24h v.s. SHAM	mmu_circRNA_25129	chr12	-	55671300	55677265	intronic	4.213	7.77317	7.332	5.22E-06	0.000195	Ralgapa1
MCAO 24h v.s. SHAM	mmu_circRNA_21394	chr1	-	178333886	178336158	exonic	-2.621	6.90231	-7.09	7.46E-06	0.000248	Hnrnpu
MCAO 24h v.s. SHAM	mmu_circRNA_36669	chr4	+	41017971	41018489	exonic	-2.249	10.0884	-3.3	0.00560116	0.022768	Nfx1
MCAO 24h v.s. SHAM	mmu_circRNA_006502	chr11	-	104368679	104378826	exonic	-2.696	10.6323	-8.37	1.21E-06	8.51E-05	Kansl1
MCAO 24h v.s. SHAM	mmu_circRNA_37478	chr4	-	116611971	116622814	exonic	2.907	10.5718	9.299	3.65E-07	5.17E-05	Nasp
MCAO 24h v.s. SHAM	mmu_circRNA_007835	chr13	-	64243633	64248466	sense overlapping	2.449	7.86489	6.976	8.88E-06	0.000273	Cdc14b
MCAO 24h v.s. SHAM	mmu_circRNA_21193	chr1	-	164439298	164441641	exonic	2.18	7.36698	4.987	0.00023719	0.002405	Atp1b1
MCAO 24h v.s. SHAM	mmu_circRNA_35381	chr3	-	55786440	56009643	exonic	-2.674	9.13842	-3.24	0.00634602	0.024921	Nbea

MCAO 24h v.s. SHAM	mmu_circRNA_37994	chr4	+	154018369	154019032	exonic	2.015	9.62179	4.76	0.00035695	0.00318	Lrrc47
MCAO 24h v.s. SHAM	mmu_circRNA_26972	chr13	+	112669586	112714247	exonic	-2.136	8.61299	-4.18	0.00104356	0.006776	Slc38a9
MCAO 24h v.s. SHAM	mmu_circRNA_29131	chr16	-	4137365	4180133	exonic	-2.307	11.6915	-4.77	0.0003483	0.003124	Crebbp
MCAO 24h v.s. SHAM	mmu_circRNA_27500	chr14	-	40907705	40934285	sense overlapping	3.28	10.731	8.397	1.17E-06	8.34E-05	Tspan14
MCAO 24h v.s. SHAM	mmu_circRNA_31359	chr18	-	16627567	16629712	exonic	2.564	11.9233	5.93	4.66E-05	0.000801	Cdh2
MCAO 24h v.s. SHAM	mmu_circRNA_30327	chr17	-	24974704	24984025	exonic	2.285	13.1057	8.77	7.17E-07	6.91E-05	Cramp1l
MCAO 24h v.s. SHAM	mmu_circRNA_34491	chr2	-	126919638	126926999	exonic	-3.557	9.25167	-6.18	3.10E-05	0.000608	Sppl2a
MCAO 24h v.s. SHAM	mmu_circRNA_32568	chr19	+	38940470	38945573	exonic	-2.56	6.48579	-3.92	0.00172081	0.009645	Hells
MCAO 24h v.s. SHAM	mmu_circRNA_36304	chr3	-	152285201	152320743	exonic	2.17	7.32744	5.262	0.00014561	0.001731	Miga1
MCAO 24h v.s. SHAM	mmu_circRNA_002959	chr8	+	85875633	85922242	exonic	-2.229	7.28717	-4.71	0.00038796	0.003341	Phkb
MCAO 24h v.s. SHAM	mmu_circRNA_21287	chr1	+	173348412	173394033	sense overlapping	-2.145	8.22131	-4.44	0.0006433	0.004799	Aim2
MCAO 24h v.s. SHAM	mmu_circRNA_43349	chr8	-	94828276	94829230	exonic	2.425	6.95634	4.081	0.0012574	0.007668	Ciapin1
MCAO 24h v.s. SHAM	mmu_circRNA_010792	chr5	+	33843111	33868878	exonic	2.191	8.71653	5.252	0.00014829	0.001742	Nsd2
MCAO 24h v.s. SHAM	mmu_circRNA_35899	chr3	+	101425450	101427440	exonic	-2.197	6.55771	-4.99	0.00023395	0.002375	Igsf3
MCAO 24h v.s. SHAM	mmu_circRNA_22174	chr10	+	69607154	69608960	intronic	2.874	10.003	10.28	1.12E-07	4.18E-05	Ank3
MCAO 24h v.s. SHAM	mmu_circRNA_31510	chr18	+	32419842	32432425	exonic	2.202	7.71473	5.211	0.00015935	0.00183	Bin1
MCAO 24h v.s. SHAM	mmu_circRNA_24243	chr11	+	104456946	104459039	antisense	2.562	11.0241	6.21	2.95E-05	0.00059	NA
MCAO 24h v.s. SHAM	mmu_circRNA_44176	chr9	+	53552249	53566832	exonic	2.165	10.6458	7.48	4.21E-06	0.00017	Npat
MCAO 24h v.s. SHAM	mmu_circRNA_26219	chr13	-	38934737	38944654	exonic	2.328	11.0949	4.619	0.00046129	0.003781	Slc35b3
MCAO 24h v.s. SHAM	mmu_circRNA_21246	chr1	+	167454466	167455198	intronic	-2.451	10.4473	-3.83	0.00201975	0.01081	Gm34207

MCAO 24h v.s. SHAM	mmu_circRNA_21246	chr1	+	167454466	167455198	intronic	-2.451	10.4473	-3.83	0.00201975	0.01081	Lrrc52
MCAO 24h v.s. SHAM	mmu_circRNA_26502	chr13	-	64104732	64113001	exonic	3.603	9.62939	9.194	4.17E-07	5.26E-05	Slc35d2
MCAO 24h v.s. SHAM	mmu_circRNA_40444	chr6	+	80947553	80953743	intergenic	4.136	5.72681	9.853	1.86E-07	4.47E-05	NA
MCAO 24h v.s. SHAM	mmu_circRNA_010803	chr6	-	115634552	115644642	exonic	-4.725	11.5318	-8.54	9.70E-07	7.77E-05	Raf1
MCAO 24h v.s. SHAM	mmu_circRNA_40795	chr6	-	116133766	116138250	exonic	-4.765	12.4267	-6.49	1.88E-05	0.000437	Tmcc1
MCAO 24h v.s. SHAM	mmu_circRNA_26526	chr13	-	68668469	68678615	exonic	3.715	12.1565	8.102	1.75E-06	0.000103	Adcy2
MCAO 24h v.s. SHAM	mmu_circRNA_35512	chr3	+	65669089	65784025	exonic	2.664	7.97222	4.058	0.00131314	0.007896	Lekr1
MCAO 24h v.s. SHAM	mmu_circRNA_20627	chr1	+	105719451	105727988	exonic	2.329	7.92264	5.718	6.66E-05	0.001014	2310035C23Rik
MCAO 24h v.s. SHAM	mmu_circRNA_41969	chr7	-	98610666	98637520	exonic	3.581	9.96169	6.969	8.98E-06	0.000273	Emsy
MCAO 24h v.s. SHAM	mmu_circRNA_34628	chr2	+	135805548	135910244	exonic	-2.026	5.98454	-6.53	1.78E-05	0.000423	Plcb4
MCAO 24h v.s. SHAM	mmu_circRNA_35656	chr3	+	83346159	83351824	intergenic	2.063	10.7672	7.558	3.76E-06	0.00016	NA
MCAO 24h v.s. SHAM	mmu_circRNA_32900	chr2	-	5914809	5923023	sense overlapping	4.465	7.94835	8.162	1.61E-06	9.72E-05	Dhtkd1
MCAO 24h v.s. SHAM	mmu_circRNA_32430	chr19	-	30781294	30993162	exonic	-2.79	6.66892	-7.71	3.04E-06	0.000142	Prkg1
MCAO 24h v.s. SHAM	mmu_circRNA_19057	chr12	+	8698087	8713939	sense overlapping	-2.561	9.4294	-5.77	6.05E-05	0.000951	Pum2
MCAO 24h v.s. SHAM	mmu_circRNA_33228	chr2	+	29679132	29689263	exonic	4.447	9.78871	6.941	9.38E-06	0.000279	Rapgef1
MCAO 24h v.s. SHAM	mmu_circRNA_39663	chr5	+	145211745	145218883	exonic	2.597	10.7123	7.131	7.04E-06	0.000242	Zkscan5
MCAO 24h v.s. SHAM	mmu_circRNA_44206	chr9	-	54423229	54447065	exonic	2.026	5.99297	7.958	2.14E-06	0.000117	Dmxl2
MCAO 24h v.s. SHAM	mmu_circRNA_008226	chr18	+	22375132	22392852	exonic	2.943	10.9977	5.862	5.23E-05	0.000863	Asxl3
MCAO 24h v.s. SHAM	mmu_circRNA_34199	chr2	+	110386528	110395382	intergenic	2.57	10.1918	8.591	9.05E-07	7.59E-05	NA
MCAO 24h v.s. SHAM	mmu_circRNA_28801	chr15	+	78044129	78047183	antisense	2.105	7.00582	6.17	3.15E-05	0.000613	LOC108168245
MCAO 24h v.s. SHAM	mmu_circRNA_28801	chr15	+	78044129	78047183	antisense	2.105	7.00582	6.17	3.15E-05	0.000613	Cacng2

MCAO 24h v.s. SHAM	mmu_circRNA_27766	chr14	-	70612667	70613420	exonic	-2.032	7.73081	-7.55	3.81E-06	0.000161	Dmtn
MCAO 24h v.s. SHAM	mmu_circRNA_33006	chr2	+	15837315	15871582	exonic	2.409	10.2742	6.623	1.53E-05	0.000387	Malrd1
MCAO 24h v.s. SHAM	mmu_circRNA_28919	chr15	+	89018149	89026636	exonic	3.324	8.81054	11.2	4.07E-08	3.37E-05	Mov10l1
MCAO 24h v.s. SHAM	mmu_circRNA_25584	chr12	+	88431020	88459203	exonic	2.951	12.9812	5.345	0.00012603	0.001585	Adck1
MCAO 24h v.s. SHAM	mmu_circRNA_40506	chr6	-	85256342	85289788	exonic	3.182	9.84178	9.386	3.28E-07	5.13E-05	Sfxn5
MCAO 24h v.s. SHAM	mmu_circRNA_011505	chr15	+	79114654	79115171	exonic	4.285	10.6939	6.473	1.94E-05	0.000444	Micall1
MCAO 24h v.s. SHAM	mmu_circRNA_20272	chr1	+	65356127	65384001	sense overlapping	2.842	7.50971	6.335	2.41E-05	0.000513	Pth2r
MCAO 24h v.s. SHAM	mmu_circRNA_27942	chr14	+	99686558	99687284	sense overlapping	2.102	6.88632	7.486	4.17E-06	0.00017	Gm34061
MCAO 24h v.s. SHAM	mmu_circRNA_31691	chr18	-	42645431	42645533	intronic	2.36	9.20963	3.809	0.00211249	0.011179	Ppp2r2b
MCAO 24h v.s. SHAM	mmu_circRNA_003739	chr14	-	121592393	121597756	sense overlapping	2.945	6.95357	8.912	5.96E-07	6.22E-05	Dock9
MCAO 24h v.s. SHAM	mmu_circRNA_26971	chr13	-	112604486	112622307	exonic	2.813	10.3028	7.708	3.03E-06	0.000142	Ddx4
MCAO 24h v.s. SHAM	mmu_circRNA_24679	chr12	-	8068097	8083726	intergenic	3.675	8.12083	9.992	1.58E-07	4.38E-05	Gm33037
MCAO 24h v.s. SHAM	mmu_circRNA_004736	chr15	-	38498668	38498768	sense overlapping	-2.054	11.6549	-6.11	3.49E-05	0.000657	Azin1
MCAO 24h v.s. SHAM	mmu_circRNA_33824	chr2	-	70786876	70795010	intronic	-2.222	11.7243	-4.82	0.00032051	0.002952	Tlk1
MCAO 24h v.s. SHAM	mmu_circRNA_19995	chr1	-	42660742	42667003	sense overlapping	2.724	7.71634	8.047	1.89E-06	0.000108	Pantr1
MCAO 24h v.s. SHAM	mmu_circRNA_42102	chr7	+	111046762	111050505	exonic	2.861	8.4655	7.836	2.53E-06	0.000131	Ctr9
MCAO 24h v.s. SHAM	mmu_circRNA_33184	chr2	-	25937945	25941469	exonic	5.079	8.60169	6.993	8.66E-06	0.000269	Camsap1
MCAO 24h v.s. SHAM	mmu_circRNA_41957	chr7	-	98230449	98259457	exonic	2.182	9.34598	4.334	0.00078274	0.005569	Acer3
MCAO 24h v.s. SHAM	mmu_circRNA_42622	chr8	+	18629545	18641665	exonic	-2.042	8.50076	-5.48	0.00010017	0.001357	Mcph1
MCAO 24h v.s. SHAM	mmu_circRNA_38427	chr5	+	33821658	33868878	exonic	2.521	8.28324	5.752	6.28E-05	0.000971	Nsd2

MCAO 24h v.s. SHAM	mmu_circRNA_29190	chr16	-	8700045	8715919	exonic	-2.197	7.1865	-4.73	0.00037664	0.003279	Usp7
MCAO 24h v.s. SHAM	mmu_circRNA_40766	chr6	-	113522313	113522807	exonic	2.265	8.05894	5.87	5.16E-05	0.000853	Emc3
MCAO 24h v.s. SHAM	mmu_circRNA_19041	chr11	-	103386956	103387349	intronic	2.675	11.6018	7.735	2.92E-06	0.000141	Plekhm1
MCAO 24h v.s. SHAM	mmu_circRNA_013131	chr4	+	24505344	24536448	exonic	2.587	8.6949	6.646	1.48E-05	0.000379	Mms22l
MCAO 24h v.s. SHAM	mmu_circRNA_009923	chr5	+	132226987	132227061	antisense	4.209	6.97919	6.295	2.57E-05	0.000538	Auts2
MCAO 24h v.s. SHAM	mmu_circRNA_20394	chr1	-	80283675	80304193	exonic	-2.158	6.96447	-5.22	0.00015691	0.001812	Cul3
MCAO 24h v.s. SHAM	mmu_circRNA_19372	chr5	-	110692047	110692115	intronic	2.397	9.74876	6.423	2.09E-05	0.000465	Ep400
MCAO 24h v.s. SHAM	mmu_circRNA_41210	chr7	+	19186101	19186371	exonic	-2.953	12.8742	-5.23	0.00015283	0.001775	Eml2
MCAO 24h v.s. SHAM	mmu_circRNA_43328	chr8	-	93104118	93108573	exonic	2.604	9.00022	8.073	1.82E-06	0.000106	Ces1c
MCAO 24h v.s. SHAM	mmu_circRNA_40341	chr6	-	60815616	60818365	exonic	-2.615	10.4939	-5.76	6.18E-05	0.000961	Snca
MCAO 24h v.s. SHAM	mmu_circRNA_37932	chr4	-	149251740	149267283	exonic	2.575	6.65868	6.729	1.30E-05	0.000344	Kif1b
MCAO 24h v.s. SHAM	mmu_circRNA_40441	chr6	+	80231810	80232745	intronic	2.001	8.76116	5.904	4.87E-05	0.000821	Lrrtm4
MCAO 24h v.s. SHAM	mmu_circRNA_19339	chr4	+	130727992	130730549	intronic	-2.384	10.5404	-5.08	0.0002014	0.002152	Pum1
MCAO 24h v.s. SHAM	mmu_circRNA_22461	chr10	-	92902014	92927839	exonic	2.005	6.25072	6.969	8.98E-06	0.000273	Cfap54

Supplementary Table S4. Parenral gene enrichment pathway analysis by GeneCoDIS3.

Groups	Pathway ID	Name	Combined Hypgeometric	Gene list
MCAO 5 min	Panther:P00047	PDGF signaling pathway	0.000206694	94190,57257,56637,110157,78514, 23871
MCAO 5 min	Kegg:04910,Panther:P00026	Insulin signaling pathway,Heterotrimeric G-protein signaling pathway-Gi alpha and Gs alpha mediated pathway	0.000548558	56637,110094,102093
MCAO 5 min	Panther:P00047,Panther:P00005,Panther:P04393	PDGF signaling pathway,Angiogenesis,Ras Pathway	0.000550474	56637,110157,23871
MCAO 5 min	Kegg:04062,Kegg:04510,Panther:P00047,Kegg:04660,Kegg:04662	Chemokine signaling pathway,Focal adhesion,PDGF signaling pathway,T cell receptor signaling pathway,B cell receptor signaling pathway	0.00062609	57257,56637,110157
MCAO 5 min	Kegg:04660	T cell receptor signaling pathway	0.000683869	21682,57257,56637,110157,228026
MCAO 5 min	Kegg:04062,Kegg:04916	Chemokine signaling pathway,Melanogenesis	0.00102507	224129,56637,110157
MCAO 5 min	Kegg:05160,Kegg:04722,Kegg:04660	Hepatitis C,Neurotrophin signaling pathway,T cell receptor signaling pathway	0.00104372	56637,110157,228026
MCAO 5 min	Kegg:04910	Insulin signaling pathway	0.00111268	56637,110094,110157,102093,22084
MCAO 5 min	Kegg:04660,Kegg:04664	T cell receptor signaling pathway,Fc epsilon RI signaling pathway	0.00197523	57257,110157,228026
MCAO 5 min	Panther:P00026	Heterotrimeric G-protein signaling pathway-Gi alpha and Gs alpha mediated pathway	0.00635568	224129,56637,110094,102093
MCAO 5 min	Kegg:04062	Chemokine signaling pathway	0.0130995	57257,224129,56637,110157
MCAO 5 min	Kegg:04020	Calcium signaling pathway	0.0130995	110094,13867,102093,20191
MCAO 5 min	Kegg:05414	Dilated cardiomyopathy	0.0138963	224129,20191,12296
MCAO 5 min	Kegg:04012	ErbB signaling pathway	0.0142372	56637,13867,110157
MCAO 5 min	Kegg:04972	Pancreatic secretion	0.0152902	224129,53869,20191
MCAO 5 min	Panther:P00034	Integrin signalling pathway	0.0287739	19248,110157,78514
MCAO 3 h	Kegg:04724	Glutamatergic synapse	0.000336807	216456,12288,14702,11514,110355,210044,105727,14660
MCAO 3 h	Kegg:04020,Kegg:04912	Calcium signaling pathway,GnRH signaling pathway	0.00144652	12288,11514,210044,19229

MCAO 3 h	Panther:P00026,Kegg:04724,Kegg:04062,Panther:P00031	Heterotrimeric G-protein signaling pathway-Gi alpha and Gs alpha mediated pathway,Glutamatergic synapse,Chemokine signaling pathway,Inflammation mediated by chemokine and cytokine signaling pathway	0.0015781	14702,110355,210044
MCAO 3 h	Kegg:04724,Kegg:05414,Kegg:04020,Kegg:04270,Kegg:04912	Glutamatergic synapse,Dilated cardiomyopathy,Calcium signaling pathway,Vascular smooth muscle contraction,GnRH signaling pathway	0.0015781	12288,11514,210044
MCAO 3 h	Kegg:04062,Kegg:04020,Kegg:04912	Chemokine signaling pathway,Calcium signaling pathway,GnRH signaling pathway	0.00181815	11514,210044,19229
MCAO 3 h	Kegg:04724,Kegg:04062	Glutamatergic synapse,Chemokine signaling pathway	0.0018236	14702,11514,110355,210044
MCAO 3 h	Kegg:04724,Panther:P04377,Panther:P04378	Glutamatergic synapse,Beta1 adrenergic receptor signaling pathway,Beta2 adrenergic receptor signaling pathway	0.00267155	12288,14702,210044
MCAO 3 h	Kegg:04972,Kegg:04970,Kegg:04270	Pancreatic secretion,Salivary secretion,Vascular smooth muscle contraction	0.00267155	16531,11514,210044
MCAO 3 h	Kegg:04062	Chemokine signaling pathway	0.00319366	14702,21844,11514,110355,210044,19229,277360
MCAO 3 h	Kegg:04020	Calcium signaling pathway	0.011213	12288,58226,110094,11514,210044,19229
MCAO 3 h	Kegg:04062,Panther:P00031	Chemokine signaling pathway,Inflammation mediated by chemokine and cytokine signaling pathway	0.0114385	14702,110355,210044,277360
MCAO 3 h	Kegg:04910	Insulin signaling pathway	0.0126031	19017,15277,110094,107746,15275
MCAO 3 h	Kegg:00500	Starch and sucrose metabolism	0.0144994	100727,15277,15275
MCAO 3 h	Kegg:05414	Dilated cardiomyopathy	0.0154781	12288,11514,210044,54376
MCAO 3 h	Kegg:04930	Type II diabetes mellitus	0.0161115	12288,15277,15275
MCAO 3 h	Kegg:04270	Vascular smooth muscle contraction	0.0268361	12288,16531,11514,210044
MCAO 3 h	Panther:P00031	Inflammation mediated by chemokine and cytokine signaling pathway	0.0379845	14702,110355,210044,18037,277360
MCAO 3 h	Panther:P00026	Heterotrimeric G-protein signaling pathway-Gi alpha and Gs alpha mediated pathway	0.0380963	14702,110094,110355,210044
MCAO 3 h	Kegg:05412	Arrhythmogenic right ventricular cardiomyopathy (ARVC)	0.0381573	12288,12558,54376
MCAO 3 h	Kegg:04976	Bile secretion	0.0383239	11514,210044,12780

MCAO 24 h	Kegg:04724,Kegg:05010	Glutamatergic synapse,Alzheimer's disease	0.0011369	12288,16438,12289,14682,18798,14812,14811
MCAO 24 h	Kegg:04020,Kegg:04912	Calcium signaling pathway,GnRH signaling pathway	0.0012582	12288,11515,16438,12289,210044,14682,19229,18798
MCAO 24 h	Kegg:04724,Kegg:05010,Kegg:04020,Kegg:04270,Kegg:04912	Glutamatergic synapse,Alzheimer's disease,Calcium signaling pathway,Vascular smooth muscle contraction,GnRH signaling pathway	0.00128962	12288,16438,12289,14682,18798
MCAO 24 h	Kegg:04972,Kegg:04971	Pancreatic secretion,Gastric acid secretion	0.00131696	11515,16438,16535,210044,14682,11931,12671,18798
MCAO 24 h	Kegg:05016,Kegg:04720	Huntington's disease,Long-term potentiation	0.0016364	12914,16438,14682,18798,14812
MCAO 24 h	Kegg:04724,Kegg:04020	Glutamatergic synapse,Calcium signaling pathway	0.00165541	12288,11515,16438,12289,210044,14682,18798,14811
MCAO 24 h	Kegg:04724,Kegg:04020,Kegg:04270,Kegg:04912	Glutamatergic synapse,Calcium signaling pathway,Vascular smooth muscle contraction,GnRH signaling pathway	0.00166159	12288,11515,16438,12289,210044,14682,18798
MCAO 24 h	Kegg:05010,Kegg:04020	Alzheimer's disease,Calcium signaling pathway	0.0017073	12288,16438,12289,14682,18798,20192,14811
MCAO 24 h	Kegg:04010,Kegg:04260,Kegg:05410,Kegg:05412,Kegg:05414	MAPK signaling pathway,Cardiac muscle contraction,Hypertrophic cardiomyopathy (HCM),Arrhythmogenic right ventricular cardiomyopathy (ARVC),Dilated cardiomyopathy	0.00177136	12288,12300,12289,54376,12296,12293
MCAO 24 h	Kegg:04972,Kegg:04970,Kegg:04020,Kegg:04971	Pancreatic secretion,Salivary secretion,Calcium signaling pathway,Gastric acid secretion	0.00177136	11515,16438,210044,14682,12671,18798
MCAO 24 h	Kegg:04724,Kegg:05010,Kegg:04020	Glutamatergic synapse,Alzheimer's disease,Calcium signaling pathway	0.00180401	12288,16438,12289,14682,18798,14811
MCAO 24 h	Panther:P00031,Panther:P00005,Kegg:04664,Kegg:04666,Panther:P00018,Panther:P00021	Inflammation mediated by chemokine and cytokine signaling pathway,Angiogenesis,Fc epsilon RI signaling pathway,Fc gamma R-mediated phagocytosis,EGF receptor signaling pathway,FGF signaling pathway	0.00180676	23797,234779,74769,110157,18754
MCAO 24 h	Kegg:04910,Kegg:05160,Kegg:04722,Kegg:05200,Kegg:04062,Kegg:04510,Kegg:05215,Panther:P00005,Panther:P00036,Kegg:05210,Kegg:05213,Kegg:04012,Kegg:04660,Panther:P04393,Kegg:04662	Insulin signaling pathway,Hepatitis C,Neurotrophin signaling pathway,Pathways in cancer,Chemokine signaling pathway,Focal adhesion,Prostate cancer,Angiogenesis,Interleukin signaling pathway,Colorectal cancer,Endometrial cancer,ErbB signaling pathway,T cell receptor signaling pathway,Ras Pathway,B cell receptor signaling pathway	0.00184384	23797,74769,56637,110157

MCAO 24 h	Kegg:04724,Kegg:05010,Kegg:05016,Kegg:04720	Glutamatergic synapse,Alzheimer's disease,Huntington's disease,Long-term potentiation	0.00184384	16438,14682,18798,14812
MCAO 24 h	Kegg:04972,Kegg:04970	Pancreatic secretion,Salivary secretion	0.00189677	11515,16438,16531,210044,14682,11931,12671,18798
MCAO 24 h	Kegg:04972,Kegg:04970,Kegg:04971	Pancreatic secretion,Salivary secretion,Gastric acid secretion	0.00191593	11515,16438,210044,14682,11931,12671,18798
MCAO 24 h	Kegg:04972,Kegg:04970,Kegg:04270	Pancreatic secretion,Salivary secretion,Vascular smooth muscle contraction	0.00192478	11515,16438,16531,210044,14682,18798
MCAO 24 h	Kegg:04724,Kegg:05010,Kegg:04720	Glutamatergic synapse,Alzheimer's disease,Long-term potentiation	0.00199415	12288,16438,14682,18798,14812,14811
MCAO 24 h	Kegg:04724	Glutamatergic synapse	0.00221888	108069,216456,12288,14702,11515,16438,12289,110355,210044,14682,105727,18798,14812,14806,14811,14660
MCAO 24 h	Kegg:04020	Calcium signaling pathway	0.002306	12288,234779,11515,16438,12289,58226,110094,13867,102093,210044,14682,12671,19229,18798,20192,18438,14811
MCAO 24 h	Kegg:04010,Kegg:04260,Kegg:05410,Kegg:05412,Kegg:05414,Panther:P04377,Panther:P04391,Panther:P04378,Panther:P00044,Panther:P04374,Panther:P00003	MAPK signaling pathway,Cardiac muscle contraction,Hypertrophic cardiomyopathy (HCM),Arrhythmogenic right ventricular cardiomyopathy (ARVC),Dilated cardiomyopathy,Beta1 adrenergic receptor signaling pathway,Oxytocin receptor mediated signaling pathway,Beta2 adrenergic receptor signaling pathway,Nicotinic acetylcholine receptor signaling pathway,5HT2 type receptor mediated signaling pathway,Alzheimer disease-amyloid secretase pathway	0.00239246	12288,12289,12296
MCAO 24 h	Kegg:04930,Panther:P04391,Panther:P04374,Kegg:04270,Panther:P00003	Type II diabetes mellitus,Oxytocin receptor mediated signaling pathway,5HT2 type receptor mediated signaling pathway,Vascular smooth muscle contraction,Alzheimer disease-amyloid secretase pathway	0.00239246	12288,12289,18754
MCAO 24 h	Kegg:04540,Kegg:04270	Gap junction,Vascular smooth muscle contraction	0.00251125	11515,16438,110157,210044,14682,19091,18798
MCAO 24 h	Kegg:04970,Kegg:04270	Salivary secretion,Vascular smooth muscle contraction	0.00251125	11515,16438,16531,210044,14682,19091,18798
MCAO 24 h	Kegg:04724,Kegg:05010,Kegg:04020,Kegg:04270,Kegg:04720,Kegg:04912	Glutamatergic synapse,Alzheimer's disease,Calcium signaling pathway,Vascular smooth muscle contraction,Long-term potentiation,GnRH signaling pathway	0.00261081	12288,16438,14682,18798

MCAO 24 h	Kegg:04970	Salivary secretion	0.00274413	11515,16438,16531,210044,14682,11931,12671,19091,18798,20192
MCAO 24 h	Kegg:04062,Kegg:04510,Kegg:04660	Chemokine signaling pathway,Focal adhesion,T cell receptor signaling pathway	0.00292113	23797,18479,74769,57257,56637,110157
MCAO 24 h	Kegg:04722,Kegg:05200,Panther:P00031,Kegg:05214,Panther:P00005,Kegg:04012,Kegg:04370,Kegg:04662,Kegg:04664,Kegg:04666,Panther:P00018,Panther:P00021,Kegg:05223	Neurotrophin signaling pathway,Pathways in cancer,Inflammation mediated by chemokine and cytokine signaling pathway,Glioma,Angiogenesis,ErbB signaling pathway,VEGF signaling pathway,B cell receptor signaling pathway,Fc epsilon RI signaling pathway,Fc gamma R-mediated phagocytosis,EGF receptor signaling pathway,FGF signaling pathway,Non-small cell lung cancer	0.0031714	23797,234779,74769,110157
MCAO 24 h	Panther:P00019,Panther:P00031	Endothelin signaling pathway,Inflammation mediated by chemokine and cytokine signaling pathway	0.00339202	23797,74769,16438,110157,210044,18754
MCAO 24 h	Kegg:04724,Kegg:04540,Kegg:04972,Kegg:04970,Kegg:04020,Kegg:04270,Kegg:04971,Kegg:04912	Glutamatergic synapse,Gap junction,Pancreatic secretion,Salivary secretion,Calcium signaling pathway,Vascular smooth muscle contraction,Gastric acid secretion,GnRH signaling pathway	0.00345573	11515,16438,210044,14682,18798
MCAO 24 h	Kegg:04722,Kegg:05200,Panther:P00005,Kegg:04012,Kegg:04662	Neurotrophin signaling pathway,Pathways in cancer,Angiogenesis,ErbB signaling pathway,B cell receptor signaling pathway	0.00359443	23797,234779,74769,56637,110157
MCAO 24 h	Kegg:04270,Kegg:04912	Vascular smooth muscle contraction,GnRH signaling pathway	0.00362859	12288,11515,16438,12289,110157,210044,14682,18798
MCAO 24 h	Panther:P00019,Panther:P00031,Panther:P00005,Kegg:04664,Kegg:04666,Panther:P00018,Panther:P00021	Endothelin signaling pathway,Inflammation mediated by chemokine and cytokine signaling pathway,Angiogenesis,Fc epsilon RI signaling pathway,Fc gamma R-mediated phagocytosis,EGF receptor signaling pathway,FGF signaling pathway	0.00366507	23797,74769,110157,18754
MCAO 24 h	Kegg:05414,Panther:P04377,Panther:P04378	Dilated cardiomyopathy,Beta1 adrenergic receptor signaling pathway,Beta2 adrenergic receptor signaling pathway	0.00366507	12288,12289,210044,12296
MCAO 24 h	Panther:P04391,Panther:P04374,Panther:P00003	Oxytocin receptor mediated signaling pathway,5HT2 type receptor mediated signaling pathway,Alzheimer disease-amyloid secretase pathway	0.00366507	12288,12289,18754,12296
MCAO 24 h	Panther:P00019	Endothelin signaling pathway	0.00367356	23797,11515,74769,16438,110157,210044,14682,18754,56529

MCAO 24 h	Kegg:04540,Kegg:04970,Kegg:04270	Gap junction,Salivary secretion,Vascular smooth muscle contraction	0.00371384	11515,16438,210044,14682,19091,18798
MCAO 24 h	Kegg:04540,Kegg:04270,Kegg:04912	Gap junction,Vascular smooth muscle contraction,GnRH signaling pathway	0.00371384	11515,16438,110157,210044,14682,18798
MCAO 24 h	Kegg:04970,Kegg:04020	Salivary secretion,Calcium signaling pathway	0.00384499	11515,16438,210044,14682,12671,18798,20192
MCAO 24 h	Kegg:04910,Kegg:05160,Kegg:04722,Kegg:05200,Kegg:04062,Kegg:05162,Kegg:04510,Kegg:05215,Panther:P00005,Panther:P00036,Kegg:05210,Kegg:05213,Kegg:04012,Kegg:04660,Panther:P04393,Kegg:04662,Panther:P00048	Insulin signaling pathway,Hepatitis C,Neurotrophin signaling pathway,Pathways in cancer,Chemokine signaling pathway,Measles,Focal adhesion,Prostate cancer,Angiogenesis,Interleukin signaling pathway,Colorectal cancer,Endometrial cancer,ErbB signaling pathway,T cell receptor signaling pathway,Ras Pathway,B cell receptor signaling pathway,PI3 kinase pathway	0.0039384	23797,74769,56637
MCAO 24 h	Panther:P00019,Panther:P00031,Panther:P00053	Endothelin signaling pathway,Inflammation mediated by chemokine and cytokine signaling pathway,T cell activation	0.00395368	23797,74769,16438,110157
MCAO 24 h	Panther:P00031,Panther:P00005,Panther:P00056,Kegg:04664,Kegg:04666,Panther:P00018,Panther:P00021	Inflammation mediated by chemokine and cytokine signaling pathway,Angiogenesis,VEGF signaling pathway,Fc epsilon RI signaling pathway,Fc gamma R-mediated phagocytosis,EGF receptor signaling pathway,FGF signaling pathway	0.00395368	234779,74769,110157,18754
MCAO 24 h	Panther:P00047,Kegg:04650,Kegg:04662,Kegg:04664,Kegg:04666,Panther:P00010	PDGF signaling pathway,Natural killer cell mediated cytotoxicity,B cell receptor signaling pathway,Fc epsilon RI signaling pathway,Fc gamma R-mediated phagocytosis,B cell activation	0.00395368	234779,74769,57257,110157
MCAO 24 h	Kegg:05010,Kegg:04970,Kegg:04020	Alzheimer's disease,Salivary secretion,Calcium signaling pathway	0.00395368	16438,14682,18798,20192
MCAO 24 h	Panther:P04391,Panther:P04374,Kegg:04270	Oxytocin receptor mediated signaling pathway,5HT2 type receptor mediated signaling pathway,Vascular smooth muscle contraction	0.00395368	12288,12289,14682,18754
MCAO 24 h	Kegg:04020,Panther:P00042	Calcium signaling pathway,Muscarinic acetylcholine receptor 1 and 3 signaling pathway	0.00395368	16438,14682,12671,14811
MCAO 24 h	Panther:P00019,Kegg:04270	Endothelin signaling pathway,Vascular smooth muscle contraction	0.00409416	11515,16438,110157,210044,14682,18754
MCAO 24 h	Kegg:04724,Kegg:05010,Kegg:04020,Kegg:04720	Glutamatergic synapse,Alzheimer's disease,Calcium signaling pathway,Long-term potentiation	0.00411172	12288,16438,14682,18798,14811

MCAO 24 h	Kegg:04722,Panther:P00031,Kegg:04662	Neurotrophin signaling pathway,Inflammation mediated by chemokine and cytokine signaling pathway,B cell receptor signaling pathway	0.00411172	23797,234779,74769,110157,18037
MCAO 24 h	Kegg:04062,Kegg:04510,Kegg:04660,Kegg:04666	Chemokine signaling pathway,Focal adhesion,T cell receptor signaling pathway,Fc gamma R-mediated phagocytosis	0.00411172	23797,18479,74769,57257,110157
MCAO 24 h	Kegg:04062,Kegg:04510,Kegg:04012,Kegg:04660	Chemokine signaling pathway,Focal adhesion,ErbB signaling pathway,T cell receptor signaling pathway	0.0043325	23797,18479,74769,56637,110157
MCAO 24 h	Kegg:04910,Kegg:05160,Kegg:04722,Kegg:05200,Kegg:04062,Kegg:04510,Kegg:05215,Panther:P00047,Panther:P00005,Panther:P00036,Kegg:05210,Kegg:05213,Kegg:04012,Kegg:04660,Panther:P04393,Kegg:04662	Insulin signaling pathway,Hepatitis C,Neurotrophin signaling pathway,Pathways in cancer,Chemokine signaling pathway,Focal adhesion,Prostate cancer,PDGF signaling pathway,Angiogenesis,Interleukin signaling pathway,Colorectal cancer,Endometrial cancer,ErbB signaling pathway,T cell receptor signaling pathway,Ras Pathway,B cell receptor signaling pathway	0.00434496	74769,56637,110157
MCAO 24 h	Panther:P00041,Kegg:04724,Kegg:05010,Kegg:04020,Kegg:04720,Panther:P00042	Metabotropic glutamate receptor group I pathway,Glutamatergic synapse,Alzheimer's disease,Calcium signaling pathway,Long-term potentiation,Muscarinic acetylcholine receptor 1 and 3 signaling pathway	0.00434496	16438,14682,14811
MCAO 24 h	Panther:P00025,Kegg:05200	Hedgehog signaling pathway,Pathways in cancer	0.00434496	12914,56637,14634
MCAO 24 h	Kegg:04724,Kegg:04540,Kegg:05010,Kegg:04972,Kegg:05016,Kegg:04970,Kegg:04020,Kegg:04270,Kegg:04971,Kegg:04720,Kegg:04730,Kegg:04912	Glutamatergic synapse,Gap junction,Alzheimer's disease,Pancreatic secretion,Huntington's disease,Salivary secretion,Calcium signaling pathway,Vascular smooth muscle contraction,Gastric acid secretion,Long-term potentiation,Long-term depression,GnRH signaling pathway	0.00434496	16438,14682,18798
MCAO 24 h	Kegg:04724,Kegg:05010,Panther:P04391,Panther:P04374,Kegg:04020,Kegg:04270,Kegg:04912	Glutamatergic synapse,Alzheimer's disease,Oxytocin receptor mediated signaling pathway,5HT2 type receptor mediated signaling pathway,Calcium signaling pathway,Vascular smooth muscle contraction,GnRH signaling pathway	0.00434496	12288,12289,14682
MCAO 24 h	Panther:P00027,Kegg:04972,Kegg:04970,Kegg:04020,Kegg:04971,Panther:P00042	Heterotrimeric G-protein signaling pathway-Gq alpha and Go alpha mediated pathway,Pancreatic secretion,Salivary secretion,Calcium signaling pathway,Gastric acid secretion,Muscarinic acetylcholine receptor 1 and 3 signaling pathway	0.00434496	16438,14682,12671

MCAO 24 h	Kegg:05016,Kegg:04720,Panther:P00057	Huntington's disease,Long-term potentiation,Wnt signaling pathway	0.00434496	12914,16438,14682
MCAO 24 h	Kegg:04910	Insulin signaling pathway	0.00439515	19017,23797,15277,74769,56637,110094,110157,102093,107746,108099,208650,22084,15275
MCAO 24 h	Kegg:04910,Kegg:05200,Kegg:05162,Kegg:04012,Kegg:04660	Insulin signaling pathway,Pathways in cancer,Measles,ErbB signaling pathway,T cell receptor signaling pathway	0.00447602	23797,74769,56637,208650
MCAO 24 h	Kegg:04724,Kegg:05414,Kegg:04020,Kegg:04270,Kegg:04912	Glutamatergic synapse,Dilated cardiomyopathy,Calcium signaling pathway,Vascular smooth muscle contraction,GnRH signaling pathway	0.00447602	12288,11515,12289,210044
MCAO 24 h	Kegg:04722,Kegg:04662	Neurotrophin signaling pathway,B cell receptor signaling pathway	0.00488223	23797,234779,74769,56637,110157,18037
MCAO 24 h	Panther:P00019,Kegg:04540,Kegg:04270,Kegg:04912	Endothelin signaling pathway,Gap junction,Vascular smooth muscle contraction,GnRH signaling pathway	0.00524124	11515,16438,110157,210044,14682
MCAO 24 h	Kegg:04062,Kegg:04510,Kegg:04660,Kegg:04662	Chemokine signaling pathway,Focal adhesion,T cell receptor signaling pathway,B cell receptor signaling pathway	0.00524124	23797,74769,57257,56637,110157
MCAO 24 h	Kegg:04540,Kegg:04270,Kegg:04730	Gap junction,Vascular smooth muscle contraction,Long-term depression	0.00524124	16438,110157,14682,19091,18798
MCAO 24 h	Panther:P00047,Panther:P00010	PDGF signaling pathway,B cell activation	0.00524124	234779,74769,16438,57257,110157
MCAO 24 h	Kegg:04270,Kegg:04720,Kegg:04912	Vascular smooth muscle contraction,Long-term potentiation,GnRH signaling pathway	0.00524124	12288,16438,110157,14682,18798
MCAO 24 h	Kegg:04664,Kegg:04666	Fc epsilon RI signaling pathway,Fc gamma R-mediated phagocytosis	0.00531586	23797,234779,74769,57257,110157,18754
MCAO 24 h	Panther:P00019,Kegg:04724,Kegg:04540,Kegg:04972,Kegg:04970,Kegg:04020,Kegg:04270,Kegg:04971,Kegg:04912	Endothelin signaling pathway,Glutamatergic synapse,Gap junction,Pancreatic secretion,Salivary secretion,Calcium signaling pathway,Vascular smooth muscle contraction,Gastric acid secretion,GnRH signaling pathway	0.0053184	11515,16438,210044,14682
MCAO 24 h	Panther:P00019,Kegg:04062,Panther:P00031,Kegg:04914	Endothelin signaling pathway,Chemokine signaling pathway,Inflammation mediated by chemokine and cytokine signaling pathway,Progesterone-mediated oocyte maturation	0.0053184	23797,74769,110157,210044
MCAO 24 h	Kegg:04930,Kegg:04973	Type II diabetes mellitus,Carbohydrate digestion and absorption	0.0053184	15277,74769,12289,15275

MCAO 24 h	Kegg:04910,Panther:P00019,Kegg:05160,Kegg:04722,Kegg:05200,Kegg:04062,Kegg:05212,Panther:P00031,Kegg:04510,Kegg:05211,Kegg:05214,Kegg:05215,Kegg:05218,Panther:P00005,Kegg:05221,Panther:P00036,Kegg:04914,Kegg:05210,Kegg:05213,Kegg:04012,Kegg:04660,Panther:P04393,Panther:P00053,Kegg:04370,Kegg:04662,Kegg:04664,Kegg:04666,Panther:P00018,Panther:P00021,Kegg:05220,Kegg:05223	Insulin signaling pathway,Endothelin signaling pathway,Hepatitis C,Neurotrophin signaling pathway,Pathways in cancer,Chemokine signaling pathway,Pancreatic cancer,Inflammation mediated by chemokine and cytokine signaling pathway,Focal adhesion,Renal cell carcinoma,Glioma,Prostate cancer,Melanoma,Angiogenesis,Acute myeloid leukemia,Interleukin signaling pathway,Progesterone-mediated oocyte maturation,Colorectal cancer,Endometrial cancer,ErbB signaling pathway,T cell receptor signaling pathway,Ras Pathway,T cell activation,VEGF signaling pathway,B cell receptor signaling pathway,Fc epsilon RI signaling pathway,Fc gamma R-mediated phagocytosis,EGF receptor signaling pathway,FGF signaling pathway,Chronic myeloid leukemia,Non-small cell lung cancer	0.00576784	23797,74769,110157
MCAO 24 h	Panther:P00026,Kegg:04972,Kegg:04970,Kegg:04020,Kegg:04971	Heterotrimeric G-protein signaling pathway-Gi alpha and Gs alpha mediated pathway,Pancreatic secretion,Salivary secretion,Calcium signaling pathway,Gastric acid secretion	0.00576784	11515,210044,12671
MCAO 24 h	Kegg:04916,Kegg:05016,Kegg:04720	Melanogenesis,Huntington's disease,Long-term potentiation	0.00576784	12914,14682,18798
MCAO 24 h	Kegg:04020,Panther:P00003	Calcium signaling pathway,Alzheimer disease-amyloid secretase pathway	0.00576784	12288,12289,12671
MCAO 24 h	Panther:P00019,Kegg:04062,Kegg:04914	Endothelin signaling pathway,Chemokine signaling pathway,Progesterone-mediated oocyte maturation	0.00589972	23797,11515,74769,110157,210044
MCAO 24 h	Panther:P00047,Kegg:04662	PDGF signaling pathway,B cell receptor signaling pathway	0.00589972	234779,74769,57257,56637,110157
MCAO 24 h	Kegg:04540,Kegg:04916,Kegg:04270,Kegg:04912	Gap junction,Melanogenesis,Vascular smooth muscle contraction,GnRH signaling pathway	0.00622278	11515,110157,210044,14682,18798
MCAO 24 h	Kegg:04650,Kegg:04666	Natural killer cell mediated cytotoxicity,Fc gamma R-mediated phagocytosis	0.00622278	18479,234779,74769,57257,110157
MCAO 24 h	Kegg:04012,Kegg:04666	ErbB signaling pathway,Fc gamma R-mediated phagocytosis	0.00622278	23797,18479,234779,74769,110157
MCAO 24 h	Kegg:04662,Kegg:04664,Kegg:04666	B cell receptor signaling pathway,Fc epsilon RI signaling pathway,Fc gamma R-mediated phagocytosis	0.00622278	23797,234779,74769,57257,110157

MCAO 24 h	Kegg:04724,Kegg:04540,Kegg:04916,Kegg:04972,Kegg:04970,Kegg:04020,Kegg:04270,Kegg:04971,Kegg:04912	Glutamatergic synapse,Gap junction,Melanogenesis,Pancreatic secretion,Salivary secretion,Calcium signaling pathway,Vascular smooth muscle contraction,Gastric acid secretion,GnRH signaling pathway	0.00625592	11515,210044,14682,18798
MCAO 24 h	Kegg:05200,Kegg:05211,Panther:P00030	Pathways in cancer,Renal cell carcinoma,Hypoxia response via HIF activation	0.00625592	23797,12914,74769,15251
MCAO 24 h	Kegg:04540,Kegg:04270,Kegg:04720,Kegg:04730,Kegg:04912	Gap junction,Vascular smooth muscle contraction,Long-term potentiation,Long-term depression,GnRH signaling pathway	0.00625592	16438,110157,14682,18798
MCAO 24 h	Kegg:04670,Panther:P00047,Kegg:04650,Kegg:04662,Kegg:04664,Kegg:04666,Panther:P00010	Leukocyte transendothelial migration,PDGF signaling pathway,Natural killer cell mediated cytotoxicity,B cell receptor signaling pathway,Fc epsilon RI signaling pathway,Fc gamma R-mediated phagocytosis,B cell activation	0.00678226	234779,74769,57257
MCAO 24 h	Kegg:04724,Kegg:05414,Panther:P04377,Panther:P04378,Kegg:04020,Kegg:04270,Kegg:04912	Glutamatergic synapse,Dilated cardiomyopathy,Beta1 adrenergic receptor signaling pathway,Beta2 adrenergic receptor signaling pathway,Calcium signaling pathway,Vascular smooth muscle contraction,GnRH signaling pathway	0.00678226	12288,12289,210044
MCAO 24 h	Kegg:04010,Kegg:04062,Kegg:04510,Kegg:05211,Kegg:04012,Kegg:04660,Kegg:04666	MAPK signaling pathway,Chemokine signaling pathway,Focal adhesion,Renal cell carcinoma,ErbB signaling pathway,T cell receptor signaling pathway,Fc gamma R-mediated phagocytosis	0.00678226	23797,18479,110157
MCAO 24 h	Kegg:04722,Kegg:05200,Panther:P00031,Kegg:05214,Panther:P00047,Panther:P00005,Kegg:04650,Kegg:04012,Panther:P00056,Kegg:04370,Kegg:04662,Kegg:04664,Kegg:04666,Panther:P00018,Panther:P00021,Panther:P00010,Kegg:05223	Neurotrophin signaling pathway,Pathways in cancer,Inflammation mediated by chemokine and cytokine signaling pathway,Glioma,PDGF signaling pathway,Angiogenesis,Natural killer cell mediated cytotoxicity,ErbB signaling pathway,VEGF signaling pathway,VEGF signaling pathway,B cell receptor signaling pathway,Fc epsilon RI signaling pathway,Fc gamma R-mediated phagocytosis,EGF receptor signaling pathway,FGF signaling pathway,B cell activation,Non-small cell lung cancer	0.00678226	234779,74769,110157
MCAO 24 h	Kegg:05200,Kegg:05152,Kegg:05211,Kegg:05215	Pathways in cancer,Tuberculosis,Renal cell carcinoma,Prostate cancer	0.00678226	23797,12914,110157
MCAO 24 h	Panther:P00031,Panther:P00047,Panther:P00010,Kegg:04070	Inflammation mediated by chemokine and cytokine signaling pathway,PDGF signaling pathway,B cell activation,Phosphatidylinositol signaling system	0.00678226	234779,74769,16438

MCAO 24 h	Kegg:04720	Long-term potentiation	0.00680694	12288,12914,16438,110157,14682,18798,14812,14811
MCAO 24 h	Kegg:04910,Kegg:04973	Insulin signaling pathway,Carbohydrate digestion and absorption	0.0068274	23797,15277,74769,15275
MCAO 24 h	Panther:P00026,Kegg:04916	Heterotrimeric G-protein signaling pathway-Gi alpha and Gs alpha mediated pathway,Melanogenesis	0.0068274	12914,11515,56637,210044
MCAO 24 h	Kegg:04062,Kegg:04510,Kegg:04810,Kegg:04650,Kegg:04660,Kegg:04666	Chemokine signaling pathway,Focal adhesion,Regulation of actin cytoskeleton,Natural killer cell mediated cytotoxicity,T cell receptor signaling pathway,Fc gamma R-mediated phagocytosis	0.0068274	18479,74769,57257,110157
MCAO 24 h	Kegg:04062,Kegg:04510,Kegg:05211,Kegg:04012,Kegg:04660,Kegg:04666	Chemokine signaling pathway,Focal adhesion,Renal cell carcinoma,ErbB signaling pathway,T cell receptor signaling pathway,Fc gamma R-mediated phagocytosis	0.0068274	23797,18479,74769,110157
MCAO 24 h	Kegg:04062,Kegg:04020,Kegg:04912	Chemokine signaling pathway,Calcium signaling pathway,GnRH signaling pathway	0.0068274	11515,210044,19229,18798
MCAO 24 h	Panther:P04391,Panther:P04374,Kegg:04020	Oxytocin receptor mediated signaling pathway,5HT2 type receptor mediated signaling pathway,Calcium signaling pathway	0.0068274	12288,234779,12289,14682
MCAO 24 h	Panther:P04391,Panther:P04374	Oxytocin receptor mediated signaling pathway,5HT2 type receptor mediated signaling pathway	0.00682776	12288,14702,234779,12289,14682,18754,12296
MCAO 24 h	Panther:P00042	Muscarinic acetylcholine receptor 1 and 3 signaling pathway	0.00682776	16536,14702,16438,14682,18754,12671,14811
MCAO 24 h	Kegg:04012,Kegg:04660	ErbB signaling pathway,T cell receptor signaling pathway	0.00684996	23797,18479,74769,56637,110157,208650
MCAO 24 h	Kegg:04270	Vascular smooth muscle contraction	0.00687392	12288,11515,16438,16531,12289,110157,210044,14682,18754,19091,18798
MCAO 24 h	Panther:P00027,Panther:P00042	Heterotrimeric G-protein signaling pathway-Gq alpha and Go alpha mediated pathway,Muscarinic acetylcholine receptor 1 and 3 signaling pathway	0.00706056	14702,16438,14682,18754,12671
MCAO 24 h	Kegg:04973	Carbohydrate digestion and absorption	0.00725085	23797,15277,74769,12289,11931,15275
MCAO 24 h	Kegg:04910,Kegg:05200,Kegg:04012,Kegg:04660	Insulin signaling pathway,Pathways in cancer,ErbB signaling pathway,T cell receptor signaling pathway	0.00727038	23797,74769,56637,110157,208650
MCAO 24 h	Kegg:04722,Kegg:05200,Panther:P00047,Panther:P00005,Kegg:04012,Kegg:04662	Neurotrophin signaling pathway,Pathways in cancer,PDGF signaling pathway,Angiogenesis,ErbB signaling pathway,B cell receptor signaling pathway	0.00763372	234779,74769,56637,110157

MCAO 24 h	Kegg:04062,Kegg:04510,Kegg:04660,Panther:P00053,Kegg:04662,Kegg:04664,Kegg:04666	Chemokine signaling pathway,Focal adhesion,T cell receptor signaling pathway,T cell activation,B cell receptor signaling pathway,Fc epsilon RI signaling pathway,Fc gamma R-mediated phagocytosis	0.00763372	23797,74769,57257,110157
MCAO 24 h	Panther:P00031,Panther:P00047,Panther:P00010	Inflammation mediated by chemokine and cytokine signaling pathway,PDGF signaling pathway,B cell activation	0.00763372	234779,74769,16438,110157
MCAO 24 h	Kegg:04540,Kegg:04970,Kegg:04270,Kegg:04730	Gap junction,Salivary secretion,Vascular smooth muscle contraction,Long-term depression	0.00763372	16438,14682,19091,18798
MCAO 24 h	Kegg:04062,Kegg:04916	Chemokine signaling pathway,Melanogenesis	0.00804764	11515,56637,110157,210044,18798
MCAO 24 h	Kegg:04972	Pancreatic secretion	0.00805769	11515,16438,16531,16535,210044,14682,11931,12671,18798,54403
MCAO 24 h	Panther:P00019,Panther:P00031,Kegg:04270	Endothelin signaling pathway,Inflammation mediated by chemokine and cytokine signaling pathway,Vascular smooth muscle contraction	0.00858646	16438,110157,210044,18754
MCAO 24 h	Kegg:04722,Panther:P00031,Kegg:04660,Kegg:04662	Neurotrophin signaling pathway,Inflammation mediated by chemokine and cytokine signaling pathway,T cell receptor signaling pathway,B cell receptor signaling pathway	0.00858646	23797,74769,110157,18037
MCAO 24 h	Kegg:04650,Kegg:04012,Kegg:04666	Natural killer cell mediated cytotoxicity,ErbB signaling pathway,Fc gamma R-mediated phagocytosis	0.00858646	18479,234779,74769,110157
MCAO 24 h	Kegg:04910,Kegg:05200,Kegg:04630,Kegg:05162,Kegg:04012,Kegg:04660,Panther:P00018,Kegg:05220	Insulin signaling pathway,Pathways in cancer,Jak-STAT signaling pathway,Measles,ErbB signaling pathway,T cell receptor signaling pathway,EGF receptor signaling pathway,Chronic myeloid leukemia	0.00863481	23797,74769,208650
MCAO 24 h	Panther:P00019,Kegg:04724,Kegg:04540,Kegg:04114,Kegg:04972,Kegg:04970,Kegg:04020,Kegg:04270,Kegg:04971,Kegg:04912	Endothelin signaling pathway,Glutamatergic synapse,Gap junction,Oocyte meiosis,Pancreatic secretion,Salivary secretion,Calcium signaling pathway,Vascular smooth muscle contraction,Gastric acid secretion,GnRH signaling pathway	0.00863481	11515,16438,210044
MCAO 24 h	Panther:P00019,Panther:P00027,Kegg:04270,Panther:P00057,Panther:P00042,Panther:P04385	Endothelin signaling pathway,Heterotrimeric G-protein signaling pathway-Gq alpha and Go alpha mediated pathway,Vascular smooth muscle contraction,Wnt signaling pathway,Muscarinic acetylcholine receptor 1 and 3 signaling pathway,Histamine H1 receptor mediated signaling pathway	0.00863481	16438,14682,18754

MCAO 24 h	Panther:P00019,Panther:P00031,Panther:P00047,Panther:P00053,Panther:P00010	Endothelin signaling pathway,Inflammation mediated by chemokine and cytokine signaling pathway,PDGF signaling pathway,T cell activation,B cell activation	0.00863481	74769,16438,110157
MCAO 24 h	Kegg:04724,Kegg:04540,Kegg:04916,Kegg:04972,Kegg:04970,Kegg:04020,Kegg:04270,Kegg:04971,Kegg:04912,Kegg:04961	Glutamatergic synapse,Gap junction,Melanogenesis,Pancreatic secretion,Salivary secretion,Calcium signaling pathway,Vascular smooth muscle contraction,Gastric acid secretion,GnRH signaling pathway,Endocrine and other factor-regulated calcium reabsorption	0.00863481	11515,14682,18798
MCAO 24 h	Kegg:04722,Kegg:05200,Kegg:04380,Panther:P00031,Kegg:05214,Panther:P00005,Kegg:04012,Kegg:04370,Kegg:04662,Kegg:04664,Kegg:04666,Panther:P00018,Panther:P00021,Kegg:05223	Neurotrophin signaling pathway,Pathways in cancer,Osteoclast differentiation,Inflammation mediated by chemokine and cytokine signaling pathway,Glioma,Angiogenesis,ErbB signaling pathway,VEGF signaling pathway,B cell receptor signaling pathway,Fc epsilon RI signaling pathway,Fc gamma R-mediated phagocytosis,EGF receptor signaling pathway,FGF signaling pathway,Non-small cell lung cancer	0.00863481	23797,234779,74769
MCAO 24 h	Kegg:04012,Panther:P00018	ErbB signaling pathway,EGF receptor signaling pathway	0.0088156	23797,234779,74769,13867,110157,208650
MCAO 24 h	Kegg:04062	Chemokine signaling pathway	0.00911687	14702,23797,18479,11515,74769,57257,56637,21844,110355,110157,210044,19229,18798,277360
MCAO 24 h	Panther:P00047	PDGF signaling pathway	0.00960617	94190,69257,50768,234779,74769,16438,57257,56637,110157,78514
MCAO 24 h	Kegg:04722,Kegg:04660,Kegg:04662	Neurotrophin signaling pathway,T cell receptor signaling pathway,B cell receptor signaling pathway	0.00968345	23797,74769,56637,110157,18037
MCAO 24 h	Kegg:05200,Kegg:04012,Panther:P00018	Pathways in cancer,ErbB signaling pathway,EGF receptor signaling pathway	0.00968345	23797,234779,74769,110157,208650
MCAO 24 h	Kegg:04062,Kegg:04912	Chemokine signaling pathway,GnRH signaling pathway	0.00968345	11515,110157,210044,19229,18798
MCAO 24 h	Panther:P00019,Kegg:04540,Kegg:04916,Kegg:04270,Kegg:04912	Endothelin signaling pathway,Gap junction,Melanogenesis,Vascular smooth muscle contraction,GnRH signaling pathway	0.00975937	11515,110157,210044,14682
MCAO 24 h	Kegg:04964	Proximal tubule bicarbonate reclamation	0.00975937	216456,11931,54403,14660
MCAO 24 h	Kegg:04062,Kegg:04510,Panther:P00047,Kegg:04660,Kegg:04662	Chemokine signaling pathway,Focal adhesion,PDGF signaling pathway,T cell receptor signaling pathway,B cell receptor signaling pathway	0.00975937	74769,57257,56637,110157
MCAO 24 h	Panther:P00047,Panther:P00053,Panther:P00010	PDGF signaling pathway,T cell activation,B cell activation	0.00975937	74769,16438,57257,110157

MCAO 24 h	Kegg:04972,Kegg:04970,Kegg:04971,Kegg:04961	Pancreatic secretion,Salivary secretion,Gastric acid secretion,Endocrine and other factor-regulated calcium reabsorption	0.00975937	11515,14682,11931,18798
MCAO 24 h	Panther:P00019,Panther:P00031,Panther:P00005,Panther:P00006,Kegg:04664,Kegg:04666,Panther:P00018,Panther:P00021	Endothelin signaling pathway,Inflammation mediated by chemokine and cytokine signaling pathway,Angiogenesis,Apoptosis signaling pathway,Fc epsilon RI signaling pathway,Fc gamma R-mediated phagocytosis,EGF receptor signaling pathway,FGF signaling pathway	0.0103323	23797,74769,18754
MCAO 24 h	Panther:P00019,Panther:P00031,Panther:P00005,Panther:P00056,Kegg:04664,Kegg:04666,Panther:P00018,Panther:P00021	Endothelin signaling pathway,Inflammation mediated by chemokine and cytokine signaling pathway,Angiogenesis,VEGF signaling pathway,Fc epsilon RI signaling pathway,Fc gamma R-mediated phagocytosis,EGF receptor signaling pathway,FGF signaling pathway	0.0103323	74769,110157,18754
MCAO 24 h	Panther:P00019,Kegg:05142,Panther:P00048	Endothelin signaling pathway,Chagas disease (American trypanosomiasis),PI3 kinase pathway	0.0103323	23797,74769,14682
MCAO 24 h	Kegg:05200,Kegg:05211,Panther:P00005,Kegg:04150,Panther:P00030	Pathways in cancer,Renal cell carcinoma,Angiogenesis,mTOR signaling pathway,Hypoxia response via HIF activation	0.0103323	23797,74769,15251
MCAO 24 h	Kegg:05010,Kegg:04916	Alzheimer's disease,Melanogenesis	0.0103323	56637,14682,18798
MCAO 24 h	Kegg:04062,Kegg:04650	Chemokine signaling pathway,Natural killer cell mediated cytotoxicity	0.0106987	18479,74769,57257,110157,19229
MCAO 24 h	Kegg:04070	Phosphatidylinositol signaling system	0.0109167	234779,74769,16438,18704,20975,380921,18798,320127
MCAO 24 h	Kegg:04910,Kegg:05200,Kegg:04012,Kegg:04660,Panther:P00018,Kegg:05220	Insulin signaling pathway,Pathways in cancer,ErbB signaling pathway,T cell receptor signaling pathway,EGF receptor signaling pathway,Chronic myeloid leukemia	0.0110913	23797,74769,110157,208650
MCAO 24 h	Kegg:05200,Panther:P00005,Panther:P00056	Pathways in cancer,Angiogenesis,VEGF signaling pathway	0.0110913	234779,74769,15251,110157
MCAO 24 h	Kegg:04910,Kegg:04722,Kegg:04510	Insulin signaling pathway,Neurotrophin signaling pathway,Focal adhesion	0.0115226	23797,74769,56637,110157,107746
MCAO 24 h	Kegg:04912	GnRH signaling pathway	0.0122924	12288,11515,16438,12289,110157,210044,14682,19229,18798
MCAO 24 h	Panther:P00026	Heterotrimeric G-protein signaling pathway-Gi alpha and Gs alpha mediated pathway	0.0127886	108069,50779,14702,12914,11515,56637,110094,110355,102093,210044,12671

MCAO 24 h	Panther:P00019,Kegg:04724,Kegg:04540,Kegg:04916,Kegg:04972,Kegg:04970,Kegg:04020,Kegg:04270,Kegg:04971,Kegg:04912	Endothelin signaling pathway,Glutamatergic synapse,Gap junction,Melanogenesis,Pancreatic secretion,Salivary secretion,Calcium signaling pathway,Vascular smooth muscle contraction,Gastric acid secretion,GnRH signaling pathway	0.0127978	11515,210044,14682
MCAO 24 h	Kegg:05200,Kegg:04630,Kegg:05211,Kegg:05215,Panther:P00059,Panther:P00030	Pathways in cancer,Jak-STAT signaling pathway,Renal cell carcinoma,Prostate cancer,p53 pathway,Hypoxia response via HIF activation	0.0127978	23797,12914,74769
MCAO 24 h	Kegg:04062,Kegg:04510,Kegg:04810,Panther:P00047,Kegg:04650,Kegg:04660,Panther:P00053,Kegg:04662,Kegg:04664,Kegg:04666,Panther:P00010	Chemokine signaling pathway,Focal adhesion,Regulation of actin cytoskeleton,PDGF signaling pathway,Natural killer cell mediated cytotoxicity,T cell receptor signaling pathway,T cell activation,B cell receptor signaling pathway,Fc epsilon RI signaling pathway,Fc gamma R-mediated phagocytosis,B cell activation	0.0127978	74769,57257,110157
MCAO 24 h	Kegg:04724,Panther:P04391,Panther:P04374	Glutamatergic synapse,Oxytocin receptor mediated signaling pathway,5HT2 type receptor mediated signaling pathway	0.0128572	12288,14702,12289,14682
MCAO 24 h	Kegg:04062,Kegg:04540,Kegg:04916,Kegg:04270,Kegg:04912	Chemokine signaling pathway,Gap junction,Melanogenesis,Vascular smooth muscle contraction,GnRH signaling pathway	0.0128572	11515,110157,210044,18798
MCAO 24 h	Panther:P00026,Kegg:04724	Heterotrimeric G-protein signaling pathway-Gi alpha and Gs alpha mediated pathway,Glutamatergic synapse	0.0140164	108069,14702,11515,110355,210044
MCAO 24 h	Panther:P00026,Kegg:04062	Heterotrimeric G-protein signaling pathway-Gi alpha and Gs alpha mediated pathway,Chemokine signaling pathway	0.0140164	14702,11515,56637,110355,210044
MCAO 24 h	Panther:P00026,Kegg:04020	Heterotrimeric G-protein signaling pathway-Gi alpha and Gs alpha mediated pathway,Calcium signaling pathway	0.0140164	11515,110094,102093,210044,12671
MCAO 24 h	Kegg:04724,Panther:P04377,Panther:P04378	Glutamatergic synapse,Beta1 adrenergic receptor signaling pathway,Beta2 adrenergic receptor signaling pathway	0.0145693	12288,14702,12289,210044
MCAO 24 h	Kegg:04910,Kegg:04930,Kegg:04973	Insulin signaling pathway,Type II diabetes mellitus,Carbohydrate digestion and absorption	0.0155572	15277,74769,15275
MCAO 24 h	Panther:P00026,Kegg:04062,Kegg:04916	Heterotrimeric G-protein signaling pathway-Gi alpha and Gs alpha mediated pathway,Chemokine signaling pathway,Melanogenesis	0.0155572	11515,56637,210044

MCAO 24 h	Kegg:04724,Kegg:04062,Kegg:04540,Kegg:04916,Kegg:04972,Kegg:04970,Kegg:04020,Kegg:04270,Kegg:04971,Kegg:04912	Glutamatergic synapse,Chemokine signaling pathway,Gap junction,Melanogenesis,Pancreatic secretion,Salivary secretion,Calcium signaling pathway,Vascular smooth muscle contraction,Gastric acid secretion,GnRH signaling pathway	0.0155572	11515,210044,18798
MCAO 24 h	Kegg:04540,Kegg:04916,Kegg:04270,Kegg:04720,Kegg:04730,Kegg:04912	Gap junction,Melanogenesis,Vascular smooth muscle contraction,Long-term potentiation,Long-term depression,GnRH signaling pathway	0.0155572	110157,14682,18798
MCAO 24 h	Kegg:04930	Type II diabetes mellitus	0.0156488	12288,15277,74769,12289,18754,15275
MCAO 24 h	Panther:P04377,Panther:P04378	Beta1 adrenergic receptor signaling pathway,Beta2 adrenergic receptor signaling pathway	0.0156732	12288,14702,12289,210044,12296
MCAO 24 h	Kegg:04910,Kegg:04722,Kegg:04510,Kegg:05211	Insulin signaling pathway,Neurotrophin signaling pathway,Focal adhesion,Renal cell carcinoma	0.0161824	23797,74769,110157,107746
MCAO 24 h	Kegg:05200,Kegg:05211,Panther:P00005	Pathways in cancer,Renal cell carcinoma,Angiogenesis	0.0161824	23797,74769,15251,110157
MCAO 24 h	Panther:P00027,Panther:P00057,Panther:P00042,Panther:P04385	Heterotrimeric G-protein signaling pathway-Gq alpha and Go alpha mediated pathway,Wnt signaling pathway,Muscarinic acetylcholine receptor 1 and 3 signaling pathway,Histamine H1 receptor mediated signaling pathway	0.0161824	14702,16438,14682,18754
MCAO 24 h	Kegg:04540	Gap junction	0.01716	11515,16438,110157,22154,210044,14682,19091,18798
MCAO 24 h	Kegg:05200,Kegg:04012	Pathways in cancer,ErbB signaling pathway	0.0179307	23797,234779,74769,56637,110157,208650
MCAO 24 h	Panther:P00019,Kegg:04062,Kegg:04540,Kegg:04914,Kegg:04916,Kegg:04270,Kegg:04912	Endothelin signaling pathway,Chemokine signaling pathway,Gap junction,Progesterone-mediated oocyte maturation,Melanogenesis,Vascular smooth muscle contraction,GnRH signaling pathway	0.0181881	11515,110157,210044
MCAO 24 h	Panther:P00019,Kegg:04540,Kegg:04270,Kegg:04720,Kegg:04730,Kegg:04912	Endothelin signaling pathway,Gap junction,Vascular smooth muscle contraction,Long-term potentiation,Long-term depression,GnRH signaling pathway	0.0181881	16438,110157,14682
MCAO 24 h	Panther:P00026,Kegg:04724,Kegg:04062,Panther:P00031	Heterotrimeric G-protein signaling pathway-Gi alpha and Gs alpha mediated pathway,Glutamatergic synapse,Chemokine signaling pathway,Inflammation mediated by chemokine and cytokine signaling pathway	0.0181881	14702,110355,210044

MCAO 24 h	Kegg:04724,Kegg:04080,Panther:P00037	Glutamatergic synapse,Neuroactive ligand-receptor interaction,Ionotropic glutamate receptor pathway	0.0181881	108069,14806,14811
MCAO 24 h	Kegg:04062,Kegg:04510,Kegg:04810,Kegg:05211,Kegg:04650,Kegg:04012,Kegg:04660,Kegg:04666	Chemokine signaling pathway,Focal adhesion,Regulation of actin cytoskeleton,Renal cell carcinoma,Natural killer cell mediated cytotoxicity,ErbB signaling pathway,T cell receptor signaling pathway,Fc gamma R-mediated phagocytosis	0.0181881	18479,74769,110157
MCAO 24 h	Kegg:05016,Kegg:04961	Huntington's disease,Endocrine and other factor-regulated calcium reabsorption	0.0181881	14682,71770,18798
MCAO 24 h	Kegg:04670,Kegg:04650	Leukocyte transendothelial migration,Natural killer cell mediated cytotoxicity	0.0184201	234779,74769,57257,19229
MCAO 24 h	Kegg:04724,Kegg:04080	Glutamatergic synapse,Neuroactive ligand-receptor interaction	0.0184201	108069,14812,14806,14811
MCAO 24 h	Kegg:04660,Kegg:04662	T cell receptor signaling pathway,B cell receptor signaling pathway	0.0186385	23797,74769,57257,56637,110157,18037
MCAO 24 h	Panther:P00026,Kegg:04724,Kegg:04062	Heterotrimeric G-protein signaling pathway-Gi alpha and Gs alpha mediated pathway,Glutamatergic synapse,Chemokine signaling pathway	0.0199814	14702,11515,110355,210044
MCAO 24 h	Kegg:04976,Kegg:04972	Bile secretion,Pancreatic secretion	0.0199814	11515,210044,11931,54403
MCAO 24 h	Panther:P00059,Panther:P04398	p53 pathway,p53 pathway feedback loops 2	0.0199814	23797,74769,20437,53892
MCAO 24 h	Panther:P00003	Alzheimer disease-amyloid secretase pathway	0.0202822	12288,12289,11491,18754,12671,12296
MCAO 24 h	Kegg:04012	ErbB signaling pathway	0.0206631	23797,18479,234779,74769,56637,13867,110157,208650
MCAO 24 h	Kegg:04062,Kegg:04810	Chemokine signaling pathway,Regulation of actin cytoskeleton	0.0211283	18479,74769,57257,21844,110157
MCAO 24 h	Panther:P04385	Histamine H1 receptor mediated signaling pathway	0.0211283	14702,234779,16438,14682,18754
MCAO 24 h	Kegg:05010,Panther:P00057	Alzheimer's disease,Wnt signaling pathway	0.0211727	16438,56637,14682
MCAO 24 h	Kegg:04260	Cardiac muscle contraction	0.021272	12288,12300,12289,11931,54376,12296,12293
MCAO 24 h	Kegg:05200,Panther:P00005	Pathways in cancer,Angiogenesis	0.0212821	23797,234779,74769,56637,15251,110157
MCAO 24 h	Kegg:04724,Panther:P00031	Glutamatergic synapse,Inflammation mediated by chemokine and cytokine signaling pathway	0.0218383	14702,16438,110355,210044

MCAO 24 h	Kegg:04724,Panther:P00042	Glutamatergic synapse,Muscarinic acetylcholine receptor 1 and 3 signaling pathway	0.0218383	14702,16438,14682,14811
MCAO 24 h	Panther:P00031,Panther:P04385	Inflammation mediated by chemokine and cytokine signaling pathway,Histamine H1 receptor mediated signaling pathway	0.0218383	14702,234779,16438,18754
MCAO 24 h	Kegg:05414	Dilated cardiomyopathy	0.0222743	12288,12300,11515,12289,210044,54376,12296,12293
MCAO 24 h	Kegg:05211	Renal cell carcinoma	0.0222759	23797,12914,18479,74769,15251,110157,107746
MCAO 24 h	Kegg:04724,Kegg:04062	Glutamatergic synapse,Chemokine signaling pathway	0.0223269	14702,11515,110355,210044,18798
MCAO 24 h	Panther:P00019,Panther:P00031,Kegg:04540,Kegg:04270,Kegg:04912	Endothelin signaling pathway,Inflammation mediated by chemokine and cytokine signaling pathway,Gap junction,Vascular smooth muscle contraction,GnRH signaling pathway	0.0240474	16438,110157,210044
MCAO 24 h	Panther:P00026,Panther:P00057	Heterotrimeric G-protein signaling pathway-Gi alpha and Gs alpha mediated pathway,Wnt signaling pathway	0.0240474	14702,12914,56637
MCAO 24 h	Kegg:04670,Kegg:04062,Kegg:04650	Leukocyte transendothelial migration,Chemokine signaling pathway,Natural killer cell mediated cytotoxicity	0.0240474	74769,57257,19229
MCAO 24 h	Kegg:05200,Kegg:05211,Panther:P00005,Panther:P00056	Pathways in cancer,Renal cell carcinoma,Angiogenesis,VEGF signaling pathway	0.0240474	74769,15251,110157
MCAO 24 h	Panther:P00027,Panther:P00031,Panther:P00057,Panther:P00042,Panther:P04385	Heterotrimeric G-protein signaling pathway-Gq alpha and Go alpha mediated pathway,Inflammation mediated by chemokine and cytokine signaling pathway,Wnt signaling pathway,Muscarinic acetylcholine receptor 1 and 3 signaling pathway,Histamine H1 receptor mediated signaling pathway	0.0240474	14702,16438,18754
MCAO 24 h	Kegg:05010,Panther:P00003	Alzheimer's disease,Alzheimer disease-amyloid secretase pathway	0.0240474	12288,12289,11491
MCAO 24 h	Panther:P04377,Panther:P04391,Panther:P04378,Panther:P04374	Beta1 adrenergic receptor signaling pathway,Oxytocin receptor mediated signaling pathway,Beta2 adrenergic receptor signaling pathway,5HT2 type receptor mediated signaling pathway	0.0243981	12288,14702,12289,12296
MCAO 24 h	Kegg:05412	Arrhythmogenic right ventricular cardiomyopathy (ARVC)	0.0261172	12288,12300,12558,12289,54376,12296,12293
MCAO 24 h	Kegg:05160,Panther:P00021	Hepatitis C,FGF signaling pathway	0.0265499	23797,74769,110157,72930
MCAO 24 h	Kegg:04510,Kegg:05211	Focal adhesion,Renal cell carcinoma	0.0275833	23797,18479,74769,110157,107746

MCAO 24 h	Kegg:04662	B cell receptor signaling pathway	0.0276651	23797,234779,74769,57257,56637,110157,18037
MCAO 24 h	Kegg:04910,Panther:P00026	Insulin signaling pathway,Heterotrimeric G-protein signaling pathway-Gi alpha and Gs alpha mediated pathway	0.0276837	56637,110094,102093
MCAO 24 h	Kegg:04724,Panther:P04377,Panther:P04391,Panther:P04378,Panther:P04374	Glutamatergic synapse,Beta1 adrenergic receptor signaling pathway,Oxytocin receptor mediated signaling pathway,Beta2 adrenergic receptor signaling pathway,5HT2 type receptor mediated signaling pathway	0.0276837	12288,14702,12289
MCAO 24 h	Kegg:05200,Kegg:05215,Kegg:04916	Pathways in cancer,Prostate cancer,Melanogenesis	0.0276837	12914,56637,110157
MCAO 24 h	Kegg:04020,Panther:P04385	Calcium signaling pathway,Histamine H1 receptor mediated signaling pathway	0.0276837	234779,16438,14682
MCAO 24 h	Kegg:00770	Pantothenate and CoA biosynthesis	0.0276837	99586,12036,12035
MCAO 24 h	Kegg:05010	Alzheimer's disease	0.0285209	12288,16438,20617,12289,56637,14682,11491,18798,14812,20192,14811
MCAO 24 h	Kegg:05200,Kegg:05211,Kegg:05215	Pathways in cancer,Renal cell carcinoma,Prostate cancer	0.0286351	23797,12914,74769,110157
MCAO 24 h	Kegg:04916,Kegg:04720	Melanogenesis,Long-term potentiation	0.0286351	12914,110157,14682,18798
MCAO 24 h	Kegg:04062,Panther:P00031	Chemokine signaling pathway,Inflammation mediated by chemokine and cytokine signaling pathway	0.030032	14702,23797,74769,110355,110157,210044,277360
MCAO 24 h	Kegg:04724,Panther:P00027,Panther:P00057,Panther:P00042,Panther:P04385	Glutamatergic synapse,Heterotrimeric G-protein signaling pathway-Gq alpha and Go alpha mediated pathway,Wnt signaling pathway,Muscarinic acetylcholine receptor 1 and 3 signaling pathway,Histamine H1 receptor mediated signaling pathway	0.0317969	14702,16438,14682
MCAO 24 h	Kegg:05200,Kegg:05210	Pathways in cancer,Colorectal cancer	0.0342221	23797,74769,56637,110157,72993,13176
MCAO 24 h	Kegg:05410	Hypertrophic cardiomyopathy (HCM)	0.0402631	12288,12300,12289,54376,108099,12296,12293
MCAO 24 h	Panther:P04394,Panther:P04391,Panther:P04374	Thyrotropin-releasing hormone receptor signaling pathway,Oxytocin receptor mediated signaling pathway,5HT2 type receptor mediated signaling pathway	0.0408669	14702,234779,14682,18754,12296
MCAO 24 h	Kegg:04530,Panther:P00021	Tight junction,FGF signaling pathway	0.0421792	23797,18754,72930
MCAO 24 h	Panther:P00005,Panther:P00056	Angiogenesis,VEGF signaling pathway	0.0438062	234779,74769,15251,110157,18754

MCAO 24 h	Kegg:04724,Panther:P00027	Glutamatergic synapse,Heterotrimeric G-protein signaling pathway-Gq alpha and Go alpha mediated pathway	0.0463942	108069,14702,16438,14682
MCAO 24 h	Kegg:05200,Kegg:04630	Pathways in cancer,Jak-STAT signaling pathway	0.0463942	23797,12914,74769,208650
MCAO 24 h	Kegg:00562,Kegg:04070	Inositol phosphate metabolism,Phosphatidylinositol signaling system	0.0466392	234779,74769,18704,20975,18798
MCAO 24 h	Panther:P00026,Panther:P05731,Kegg:04724,Kegg:04062	Heterotrimeric G-protein signaling pathway-Gi alpha and Gs alpha mediated pathway,GABA-B receptor II signaling,Glutamatergic synapse,Chemokine signaling pathway	0.0467942	14702,11515,210044
MCAO 24 h	Panther:P00027,Panther:P04394,Panther:P04391,Panther:P04374,Panther:P00057,Panther:P00042,Panther:P04385	Heterotrimeric G-protein signaling pathway-Gq alpha and Go alpha mediated pathway,Thyrotropin-releasing hormone receptor signaling pathway,Oxytocin receptor mediated signaling pathway,5HT2 type receptor mediated signaling pathway,Wnt signaling pathway,Muscarinic acetylcholine receptor 1 and 3 signaling pathway,Histamine H1 receptor mediated signaling pathway	0.0467942	14702,14682,18754
MCAO 24 h	Kegg:04976,Kegg:04972,Kegg:04970,Kegg:04971	Bile secretion,Pancreatic secretion,Salivary secretion,Gastric acid secretion	0.0467942	11515,210044,11931
MCAO 24 h	Kegg:04666	Fc gamma R-mediated phagocytosis	0.0481799	23797,18479,234779,74769,57257,110157,18754
MCAO 24 h	Kegg:04920	Adipocytokine signaling pathway	0.0482842	68465,19017,12491,23797,18037,108099
MCAO 24 h	Panther:P04394,Panther:P04391,Panther:P04374,Panther:P04385	Thyrotropin-releasing hormone receptor signaling pathway,Oxytocin receptor mediated signaling pathway,5HT2 type receptor mediated signaling pathway,Histamine H1 receptor mediated signaling pathway	0.0493443	14702,234779,14682,18754

Supplementary Table S5. miRNA binding sites of the significantly differentially expressed circRNAs.

Group	circRNA	miRNA
MCAO 5min	mmu_circRNA_29699	mmu-miR-433-3p

MCAO 5min	mmu_circRNA_013636	
MCAO 5min	mmu_circRNA_33003	mmu-miR-31-5p
MCAO 5min	mmu_circRNA_001149	mmu-miR-1247-5p
MCAO 5min	mmu_circRNA_40539	mmu-miR-1896
MCAO 5min	mmu_circRNA_32884	NA
MCAO 5min	mmu_circRNA_26948	NA
MCAO 5min	mmu_circRNA_001579	NA
MCAO 5min	mmu_circRNA_19729	mmu-miR-542-5p
MCAO 5min	mmu_circRNA_18979	NA
MCAO 5min	mmu_circRNA_004072	NA
MCAO 5min	mmu_circRNA_010803	NA
MCAO 5min	mmu_circRNA_43867	NA
MCAO 5min	mmu_circRNA_25433	mmu-miR-30c-1-3p/mmu-miR-5623-5p
MCAO 5min	mmu_circRNA_45921	mmu-miR-1962
MCAO 5min	mmu_circRNA_22898	NA
MCAO 5min	mmu_circRNA_003169	mmu-miR-470-5p
MCAO 5min	mmu_circRNA_43344	NA
MCAO 5min	mmu_circRNA_003795	NA
MCAO 5min	mmu_circRNA_34491	mmu-miR-669p-3p
MCAO 5min	mmu_circRNA_41878	mmu-miR-361-3p,mmu-miR-3058-5p,mmu-miR-743a-5p
MCAO 5min	mmu_circRNA_35065	mmu-miR-344d-3-5p， mmu-miR-344d-1-5p
MCAO 5min	mmu_circRNA_42362	NA
MCAO 5min	mmu_circRNA_011174	NA
MCAO 5min	mmu_circRNA_35587	NA

MCAO 5min	mmu_circRNA_35588	NA
MCAO 5min	mmu_circRNA_43955	NA
MCAO 5min	mmu_circRNA_26021	mmu-miR-1940
MCAO 5min	mmu_circRNA_32190	mmu-miR-3057-5p
MCAO 5min	mmu_circRNA_20066	NA
MCAO 5min	mmu_circRNA_35589	NA
MCAO 3h	mmu_circRNA_40203	NA
MCAO 3h	mmu_circRNA_36481	NA
MCAO 3h	mmu_circRNA_45876	NA
MCAO 3h	mmu_circRNA_24344	mmu-miR-3093-3p
MCAO 3h	mmu_circRNA_27197	mmu-miR-5113
MCAO 3h	mmu_circRNA_010777	mmu-miR-3089-3p
MCAO 3h	mmu_circRNA_27565	NA
MCAO 3h	mmu_circRNA_43956	NA
MCAO 3h	mmu_circRNA_19178	NA
MCAO 3h	mmu_circRNA_19178	NA
MCAO 3h	mmu_circRNA_29760	mmu-miR-484，mmu-miR-693-3p
MCAO 3h	mmu_circRNA_30586	NA
MCAO 3h	mmu_circRNA_34968	NA
MCAO 3h	mmu_circRNA_011505	NA
MCAO 3h	mmu_circRNA_19015	NA
MCAO 3h	mmu_circRNA_27287	NA
MCAO 3h	mmu_circRNA_27821	NA
MCAO 3h	mmu_circRNA_015541	NA

MCAO 3h	mmu_circRNA_015541	NA
MCAO 3h	mmu_circRNA_001832	NA
MCAO 3h	mmu_circRNA_38760	NA
MCAO 3h	mmu_circRNA_26316	NA
MCAO 3h	mmu_circRNA_38328	mmu-miR-141-5p
MCAO 3h	mmu_circRNA_23643	mmu-miR-3070b-3p，mmu-miR-5046
MCAO 3h	mmu_circRNA_43344	NA
MCAO 3h	mmu_circRNA_45921	mmu-miR-1962
MCAO 3h	mmu_circRNA_37699	NA
MCAO 3h	mmu_circRNA_002246	NA
MCAO 24h	mmu_circRNA_43956	NA
MCAO 24h	mmu_circRNA_37286	mmu-miR-149-3p,mmu-miR-199a-5p,mmu-miR-214-3p,mmu-miR-466a-3p,mmu-miR-467a-3p,mmu-miR-467a-4p,mmu-miR-467a-5p,mmu-miR-467a-6p,mmu-miR-467a-7p,mmu-miR-467a-8p,mmu-miR-467a-9p,mmu-miR-467a-10p,mmu-miR-467a-11p,mmu-miR-467a-12p,mmu-miR-467a-13p,mmu-miR-467a-14p,mmu-miR-467a-15p,mmu-miR-467a-16p,mmu-miR-467a-17p,mmu-miR-467a-20p,mmu-miR-467a-21p,mmu-miR-467a-22p,mmu-miR-467a-23p,mmu-miR-467a-24p,mmu-miR-467a-25p,mmu-miR-467a-26p,mmu-miR-467a-27p,mmu-miR-467a-28p,mmu-miR-467a-29p,mmu-miR-467a-30p,mmu-miR-467a-31p,mmu-miR-467a-32p,mmu-miR-467a-33p,mmu-miR-467a-34p,mmu-miR-467a-35p,mmu-miR-467a-36p,mmu-miR-467a-37p,mmu-miR-467a-38p,mmu-miR-467a-39p,mmu-miR-467a-40p,mmu-miR-467a-41p,mmu-miR-467a-42p,mmu-miR-467a-43p,mmu-miR-467a-44p,mmu-miR-467a-45p,mmu-miR-467a-46p,mmu-miR-467a-47p,mmu-miR-467a-48p,mmu-miR-467a-49p,mmu-miR-467a-50p,mmu-miR-467a-51p,mmu-miR-467a-52p,mmu-miR-467a-53p,mmu-miR-467a-54p,mmu-miR-467a-55p,mmu-miR-467a-56p,mmu-miR-467a-57p,mmu-miR-467a-58p,mmu-miR-467a-59p,mmu-miR-467a-60p,mmu-miR-467a-61p,mmu-miR-467a-62p,mmu-miR-467a-63p,mmu-miR-467a-64p,mmu-miR-467a-65p,mmu-miR-467a-66p,mmu-miR-467a-67p,mmu-miR-467a-68p,mmu-miR-467a-69p,mmu-miR-467a-70p,mmu-miR-467a-71p,mmu-miR-467a-72p,mmu-miR-467a-73p,mmu-miR-467a-74p,mmu-miR-467a-75p,mmu-miR-467a-76p,mmu-miR-467a-77p,mmu-miR-467a-78p,mmu-miR-467a-79p,mmu-miR-467a-80p,mmu-miR-467a-81p,mmu-miR-467a-82p,mmu-miR-467a-83p,mmu-miR-467a-84p,mmu-miR-467a-85p,mmu-miR-467a-86p,mmu-miR-467a-87p,mmu-miR-467a-88p,mmu-miR-467a-89p,mmu-miR-467a-90p,mmu-miR-467a-91p,mmu-miR-467a-92p,mmu-miR-467a-93p,mmu-miR-467a-94p,mmu-miR-467a-95p,mmu-miR-467a-96p,mmu-miR-467a-97p,mmu-miR-467a-98p,mmu-miR-467a-99p,mmu-miR-467a-100p,mmu-miR-467a-101p,mmu-miR-467a-102p,mmu-miR-467a-103p,mmu-miR-467a-104p,mmu-miR-467a-105p,mmu-miR-467a-106p,mmu-miR-467a-107p,mmu-miR-467a-108p,mmu-miR-467a-109p,mmu-miR-467a-110p,mmu-miR-467a-111p,mmu-miR-467a-112p,mmu-miR-467a-113p,mmu-miR-467a-114p,mmu-miR-467a-115p,mmu-miR-

		467a-116p,mmu-miR-467a-117p,mmu-miR-467a-118p,mmu-miR-467a-119p,mmu-miR-467a-120p,mmu-miR-467a-121p,mmu-miR-467a-122p,mmu-miR-467a-123p,mmu-miR-467a-124p,mmu-miR-467a-125p,mmu-miR-467a-126p,mmu-miR-467a-127p,mmu-miR-467a-128p,mmu-miR-467a-129p,mmu-miR-467a-130p,mmu-miR-467a-131p,mmu-miR-467a-132p,mmu-miR-467a-133p,mmu-miR-467a-134p,mmu-miR-467a-135p,mmu-miR-467a-136p,mmu-miR-467a-137p,mmu-miR-467a-138p,mmu-miR-467a-139p,mmu-miR-467a-140p,mmu-miR-467a-141p,mmu-miR-467a-142p,mmu-miR-467a-143p,mmu-miR-467a-144p,mmu-miR-467a-145p,mmu-miR-467a-146p,mmu-miR-467a-147p,mmu-miR-467a-148p,mmu-miR-467a-149p,mmu-miR-467a-149p
MCAO 24h	mmu_circRNA_40203	NA
MCAO 24h	mmu_circRNA_31169	NA
MCAO 24h	mmu_circRNA_19077	mmu-miR-669k-5p，mmu-miR-1970
MCAO 24h	mmu_circRNA_45876	NA
MCAO 24h	mmu_circRNA_45047	mmu-miR-346-5p
MCAO 24h	mmu_circRNA_24344	mmu-miR-3093-3p
MCAO 24h	mmu_circRNA_22526	NA
MCAO 24h	mmu_circRNA_30884	NA
MCAO 24h	mmu_circRNA_010777	mmu-miR-3089-3p
MCAO 24h	mmu_circRNA_42273	mmu-miR-370-3p
MCAO 24h	mmu_circRNA_19073	NA
MCAO 24h	mmu_circRNA_36481	NA

MCAO 24h	mmu_circRNA_33184	mmu-miR-346-3p，mmu-miR-365-2-5p，mmu-miR-693-3p，mmu-miR-1956，mmu-miR-3075-3p，mmu-miR-3083-5p，mmu-miR-3096-3p，mmu-miR-5102
MCAO 24h	mmu_circRNA_37766	mmu-miR-3104-3p
MCAO 24h	mmu_circRNA_002520	NA
MCAO 24h	mmu_circRNA_27821	NA
MCAO 24h	mmu_circRNA_22663	mmu-miR-669c-3p，mmu-miR-676-5p，mmu-miR-466c-5p，mmu-miR-466e-5p，mmu-miR-1951，mmu-miR-3074-5p
MCAO 24h	mmu_circRNA_33499	mmu-miR-15a-3p，mmu-miR-706，mmu-miR-709，mmu-miR-717，mmu-miR-466i-5p，mmu-miR-466i-5p，mmu-miR-1187，mmu-miR-466k，mmu-miR-1935，mmu-miR-1946a，mmu-miR-1950，mmu-miR-669n，mmu-miR-3473c
MCAO 24h	mmu_circRNA_19355	NA
MCAO 24h	mmu_circRNA_22867	mmu-miR-346-3p,mmu-miR-377-5p
MCAO 24h	mmu_circRNA_003023	NA
MCAO 24h	mmu_circRNA_016017	NA
MCAO 24h	mmu_circRNA_34504	NA
MCAO 24h	mmu_circRNA_36263	NA
MCAO 24h	mmu_circRNA_004682	NA
MCAO 24h	mmu_circRNA_34491	mmu-miR-669p-3p
MCAO 24h	mmu_circRNA_19277	NA
MCAO 24h	mmu_circRNA_24446	mmu-miR-299-5p，mmu-miR-324-3p，mmu-miR-340-3p，mmu-miR-346-3p，mmu-miR-383-3p，mmu-miR-433-3p，mmu-miR-1198-5p
MCAO 24h	mmu_circRNA_24446	mmu-miR-299-5p，mmu-miR-324-3p，mmu-miR-340-3p，mmu-miR-346-3p，mmu-miR-383-3p，mmu-miR-433-3p，mmu-miR-1198-5p
MCAO 24h	mmu_circRNA_27348	NA
MCAO 24h	mmu_circRNA_41054	NA
MCAO 24h	mmu_circRNA_32884	NA

MCAO 24h	mmu_circRNA_43344	NA
MCAO 24h	mmu_circRNA_27614	NA
MCAO 24h	mmu_circRNA_23643	mmu-miR-3070b-3p,mmu-miR-5046
MCAO 24h	mmu_circRNA_26097	NA
MCAO 24h	mmu_circRNA_012978	NA
MCAO 24h	mmu_circRNA_37699	NA
MCAO 24h	mmu_circRNA_35175	NA
MCAO 24h	mmu_circRNA_39121	NA
MCAO 24h	mmu_circRNA_45921	mmu-miR-1962
MCAO 24h	mmu_circRNA_003795	NA
MCAO 24h	mmu_circRNA_002246	NA
MCAO 24h	mmu_circRNA_010803	NA
MCAO 24h	mmu_circRNA_40795	mmu-miR-760-3p,mmu-miR-669c-3p
MCAO 24h	mmu_circRNA_015506	NA
MCAO 24h	mmu_circRNA_41878	mmu-miR-361-3p,mmu-miR-3058-5p,mmu-miR-743a-5p

Supplementary Table S6. Gene Ontology analysis of the predicted circRNA-miRNA target genes.

Group	GO category	p-value	genes	miRNAs
MCAO 5 m vs SHAM	anatomical structure development	0	332	7
MCAO 5 m vs SHAM	intracellular	0	889	8
MCAO 5 m vs SHAM	biological_process	0	1463	8
MCAO 5 m vs SHAM	cell	0	1090	10
MCAO 5 m vs SHAM	molecular_function	1.72E-14	1092	5
MCAO 5 m vs SHAM	cell differentiation	4.45E-11	183	4
MCAO 5 m vs SHAM	organelle	0.0000382	591	4
MCAO 5 m vs SHAM	embyo development	0.000173629	46	3
MCAO 5 m vs SHAM	cellular_component	0.02759663	458	3
MCAO 3 h vs SHAM	molecular_function	0	1852	11
MCAO 3 h vs SHAM	anatomical structure development	0	431	10
MCAO 3 h vs SHAM	biological_process	0	2202	14
MCAO 3 h vs SHAM	intracellular	0	1470	17
MCAO 3 h vs SHAM	cell	0	1769	20
MCAO 3 h vs SHAM	cell differentiation	4.63E-09	239	6
MCAO 3 h vs SHAM	cellular_component	0.0000904	1005	5

MCAO 3 h vs SHAM	organelle	0.000145454	486	3
MCAO 3 h vs SHAM	ion binding	0.001951403	23	1
MCAO 3 h vs SHAM	embyo development	0.002613401	65	3
MCAO 24 h vs SHAM	cell	0	1913	15
MCAO 24 h vs SHAM	intracellular	7.35E-158	1626	15
MCAO 24 h vs SHAM	biological_process	5.97E-92	2767	15
MCAO 24 h vs SHAM	anatomical structure development	8.93E-66	594	14
MCAO 24 h vs SHAM	molecular_function	4.24E-47	2701	15
MCAO 24 h vs SHAM	cell differentiation	3.28E-28	426	14
MCAO 24 h vs SHAM	cellular_component	1.68E-22	2653	15
MCAO 24 h vs SHAM	organelle	7.52E-19	1364	15
MCAO 24 h vs SHAM	embyo development	3.24E-16	156	14
MCAO 24 h vs SHAM	anatomical structure formation involved in morphogenesis	8.44E-14	146	14
MCAO 24 h vs SHAM	ion binding	1.87E-11	842	15
MCAO 24 h vs SHAM	cellular protein modification process	1.17E-09	353	15
MCAO 24 h vs SHAM	cell morphogenesis	1.99E-08	115	14
MCAO 24 h vs SHAM	chromosome organization	0.00000216	94	13
MCAO 24 h vs SHAM	homeostatic process	0.00000226	147	14

MCAO 24 h vs SHAM	nucleic acid binding transcription factor activity	0.000231647	151	14
MCAO 24 h vs SHAM	developmental maturation	0.000535648	31	9
MCAO 24 h vs SHAM	biosynthetic process	0.000797789	525	15
MCAO 24 h vs SHAM	growth	0.00178368	73	14
MCAO 24 h vs SHAM	cell motility	0.005024911	94	14
MCAO 24 h vs SHAM	cytoplasmic membrane-bounded vesicle	0.032080963	82	14
MCAO 24 h vs SHAM	chromosome	0.04118051	102	14

Supplementary Table S7. KEGG pathway analysis of the differentially expressed circRNAs in different time points of MCAO

Group	KEGG pathway	p-value	genes	miRNAs
Upregulation circRNAs in MCAO 5 m vs SHAM	Hippo signaling pathway	6.5497E-08	Fgf1, Nf2, Ppp2ca, Trp73, Yap1, Bmp2, Gli	mmu-miR-344d-3-5p,mmu-miR-344d-1-5
Upregulation circRNAs in MCAO 5 m vs SHAM	Biosynthesis of unsaturated fatty acids	3.62879E-05	Acot2	mmu-miR-344d-3-5p,mmu-miR-344d-1-5
Upregulation circRNAs in MCAO 5 m vs SHAM	Folate biosynthesis	0.003002272	Gch1	mmu-miR-344d-3-5p,mmu-miR-344d-1-5
Upregulation circRNAs in MCAO 5 m vs SHAM	Vitamin B6 metabolism	0.030929924	Pdxk	mmu-miR-344d-3-5p,mmu-miR-344d-1-5
Downregulation circRNAs in MCAO 5 m vs SHAM	Mucin type O-Glycan biosynthesis	1.8699E-05	St6galnac1, Galnt5, Galnt7	mmu-miR-5623-5p
Downregulation circRNAs in MCAO 5 m vs SHAM	Cytokine-cytokine receptor interaction	0.00106912	Gm13304, Il1r1, Tnfrsf12a, Flt4, Gm21541, Il6st	mmu-miR-30c-1-3p,mmu-miR-1962
Downregulation circRNAs in MCAO 5 m vs SHAM	Gap junction	0.001832478	Gjd2, Mapk3, Csnk1d, Gja1, Prkg1	mmu-miR-5623-5p
Downregulation circRNAs in MCAO 5 m vs SHAM	Metabolism of xenobiotics by cytochrome P450	0.003354516	Ugt2b5, Mgst3, Gstm2, Ephx1, Ugt2b37	mmu-miR-1962,mmu-miR-361-3p
Upregulation circRNAs in MCAO 3 h vs SHAM	Thyroid hormone synthesis	0.002103258	Prkca,Tg,Plcb2	mmu-miR-3089-3p, mmu-miR-3093-3p,mmu-miR-484
Downregulation circRNAs in MCAO 3 h vs SHAM	Glycosphingolipid biosynthesis - lacto and neolacto series	2.78494E-10	Gcnt2, Fut9,Fut1	mmu-miR-199a-5p,mmu-miR-199b-5p,mmu-miR-770-3p
Downregulation circRNAs in MCAO 3 h vs SHAM	ECM-receptor interaction	8.102E-05	Col6a3, Itgb3, Lamc2, Thbs2, Hmmr, Itgav, Tnxb, Lamc1	mmu-miR-324-3p,mmu-miR-667-5p,mmu-miR-5113,mmu-miR-141-5p
Downregulation circRNAs in MCAO 3 h vs SHAM	Metabolism of xenobiotics by cytochrome P450	0.007447632	Cyp1a1, Gstm5, Ugt2b5, Mgst3, Gstm2, Ephx1, Ugt2b37	mmu-miR-361-3p,mmu-miR-770-3p,mmu-miR-1962
Upregulation circRNAs in MCAO 24 h vs SHAM	Fatty acid degradation	3.14963E-06	Hadha, Acox1, Ehhadh	mmu-miR-669c-3p,mmu-miR-669k-5p
Upregulation circRNAs in MCAO 24 h vs SHAM	Fatty acid metabolism	0.00056093	Hadha, Elovl5, Acox1, Ehhadh, Ppt1	mmu-miR-346-5p,mmu-miR-669c-3p,mmu-miR-669k-5p
Upregulation circRNAs in MCAO 24 h vs SHAM	Type II diabetes mellitus	0.000585123	Pik3r1, Pik3r3, Irs4, Ikbkb, Cacna1b, Insr, Irs1, Mapk9, Prkcd, Hkdc1, Pik3cd, Mapk8, Hk2, Adipoq	mmu-miR-1970,mmu-miR-669c-3p,mmu-miR-669b-3p,mmu-miR-297b-3p,mmu-miR-3093-3p,mmu-miR-193a-5p,mmu-miR-346-5p,mmu-miR-677-3p
Upregulation circRNAs in MCAO 24 h vs SHAM	Fatty acid elongation	0.001239343	Hadha, Elovl5, Elovl1, Ppt1	mmu-miR-346-5p,mmu-miR-669c-3p,mmu-miR-669k-5p
Upregulation circRNAs in MCAO 24 h vs SHAM	Phosphatidylinositol signaling system	0.00477227	Inpp1, Pik3r1, Pik3r3, Itpr2, Pik3c2g, Inpp5j, Pik3c3, Cds2, Prkca, Plcb1, Itpk1, Pten, Pik3cd, Plcb2	mmu-miR-669k-5p,mmu-miR-669c-3p,mmu-miR-346-5p,mmu-miR-193b-5p,mmu-miR-3093-3p,mmu-miR-1970
Upregulation circRNAs in MCAO 24 h vs SHAM	mTOR signaling pathway	0.020401333	Pik3r1, Pik3r3, Eif4e2, Ikbkb, Ulk3, Rps6ka6, Irs1, Vegfa, Prkaa1, Eif4ebp1, Prkca, Rraga, Pten, Pik3cd, Eif4e1b	mmu-miR-677-3p,mmu-miR-669c-3p,mmu-miR-193b-5p,mmu-miR-346-5p,mmu-miR-3093-3p,mmu-miR-669b-3p
Upregulation circRNAs in MCAO 24 h vs SHAM	TNF signaling pathway	0.025829681	Mapk14, Nfkbia, Pik3r1, Pik3r3, Ikbkb, Lif, Junb, Creb5, Casp3, Tnfrsf1b, Il18r1, Mapk9, Sele, Tab3,	mmu-miR-346-5p,mmu-miR-669c-3p,mmu-miR-3093-

			Tradd, Ripk1, Pik3cd, Mapk8	3p,mmu-miR-677-3p,mmu-miR-669b-3p
Upregulation circRNAs in MCAO 24 h vs SHAM	Adipocytokine signaling pathway	0.025829681	Nfkbia, Irs4, Ikbkb, Camkk2, Irs1, Tnfrsf1b, Prkcq, Prkaa1, Mapk9, Ppargc1a, Prkag2, Tradd, Pck1, Mapk8, Adipoq	mmu-miR-1970,mmu-miR-669c-3p,mmu-miR-193a-5p,mmu-miR-346-5p,mmu-miR-3093-3p,mmu-miR-677-3p,mmu-miR-669b-3p
Upregulation circRNAs in MCAO 24 h vs SHAM	Thyroid hormone synthesis	0.048874445	Itpr2, Creb5, Adcy1, Prkca, Tg, Plcb1, Plcb2	mmu-miR-3093-3p,mmu-miR-346-5p,mmu-miR-669c-3p,mmu-miR-193b-5p,mmu-miR-669k-5p,mmu-miR-677-3p
Downregulation circRNAs in MCAO 24 h vs SHAM	Systemic lupus erythematosus	1.19826E-06	Tnf, Hist3h2ba, Hist3h2a, Hist2h3c2, Hist2h3c1, Hist1h3d, Hist1h3b, Hist1h3a, Hist1h2bj, Hist1h2bg, Hist1h2bf, Hist1h2ap, Hist1h2ao, Hist1h2ag, Hist1h2ae, Hist1h2ad, Hist1h2ab, H2bfm, Cd28	mmu-miR-760-3p, mmu-miR-1962
Downregulation circRNAs in MCAO 24 h vs SHAM	Alcoholism	0.000446567	Ppp1r1b, Hist3h2ba, Hist3h2a, Hist2h3c2, Hist2h3c1, Hist1h3d, Hist1h3b, Hist1h3a, Hist1h2bj, Hist1h2bg, Hist1h2bf, Hist1h2ap, Hist1h2ao, Hist1h2ag, Hist1h2ae, Hist1h2ad, Hist1h2ab, H2bfm	mmu-miR-760-3p
Downregulation circRNAs in MCAO 24 h vs SHAM	Other types of O-glycan biosynthesis	0.000446567	Ugt2b5, Hsd17b6, Ugt2b37	mmu-miR-1962,mmu-miR-760-3p
Downregulation circRNAs in MCAO 24 h vs SHAM	Steroid hormone biosynthesis	0.008920821	Ugt2b5, Mgst3, Ugt2b37	mmu-miR-1962,mmu-miR-760-3p
Downregulation circRNAs in MCAO 24 h vs SHAM	Metabolism of xenobiotics by cytochrome P450	0.036022081	Ugt2b5, Mgst3, Ugt2b37	mmu-miR-1962
Downregulation circRNAs in MCAO 24 h vs SHAM	Inositol phosphate metabolism	0.036022081	Pi4k2a, Synj1, Plcg2, Pip5kl1, Impa2	mmu-miR-1962,mmu-miR-760-3p
Downregulation circRNAs in MCAO 24 h vs SHAM	Cytokine-cytokine receptor interaction	0.036022081	Cxcl9, Gm13304, Il2ra, Flt4, Gm21541, Il6st, Ngfr, Tnf, Flt3	mmu-miR-1962,mmu-miR-760-3p
Downregulation circRNAs in MCAO 24 h vs SHAM	Phosphatidylinositol signaling system	0.036022081	Pi4k2a, Cds2, Synj1, Plcg2, Impa2	mmu-miR-1962,mmu-miR-760-3p

Supplementary Table S8. Baseline characteristics of stroke patients and control subjects.

Demographic and clinicopathological variables	Ischemic stroke n (%)	Control subjects n (%)
Total, n	8	8
Age (Mean±SD)	55.4±12.3	54.11±10.1
Male	7(77.8%)	7(77.8%)
**Vascular risk factors, n (%)**		
Hypertension	3(33.3%)	2(22.2%)
Smoking history	4(44.4%)	2(22.2%)
Hypercholesterolemia	0(0.0%)	0(0.0%)
Diabetes mellitus	0(0.0%)	0(0.0%)
Previous TIA/stroke/MI	0(0.0%)	0(0.0%)
Atrial fibrillation	2(22.2%)	2(22.2%)
Chronic kidney disease	0(0.0%)	1(11.1%)
Family history	0(0.0%)	0(0.0%)
**Laboratory parameters, mean±SD**		
Glucose [mmol/l]	5.53±1.1	4.99±0.4
Creatinine [mg/dl]	207.9±394.5*	73.37±9.6
hs-CRP [mg/dl]	7.82±12.2*	3.29±0.9
D-dimer [ug/mL]	865.00±922.8*	289.00±70.9
Total Cholesterol [mg/dl]	9.95±10.8*	4.70±0.8
Triglycerides [mg/dl]*	1.13±0.4	1.77±0.7

LDL [mg/dl]	2.49±1.0	2.85±0.4
HDL [mg/dl]	1.19±0.6	1.62±.1.2
AST [U/l]*	22.00±6.2	18.67±6.1
ALT [U/l]	24.78±17.4	17.89±4.6
Uric acid [umol/L]	320.20±88.7	402.1±93.6
Albumin [g/L]	37.78±2.7	40.7±1.6
**Medication, n (%)**		
Statins	0(0.0%)	0(0.0%)
ACE I / ARBs	0(0.0%)	2(22.2%)
Beta-Blockers	1(11.1%)	0(0.0%)
Calcium channel blockers	2(22.2%)	0(0.0%)
Anticoagulant	0(0.0%)	0(0.0%)
Antiplatelet therapy	0(0.0%)	1(11.1%)
**Imaging findings, n (%)**		
ICA occlusion	1(11.1%)	n/a
MCA occlusion	7(77.8%)	n/a
Other occlusion	1(11.1%)	n/a
DWI lesion volume at baseline, mean±SD [mL]	32.9±36.3*	n/a
ΔT, mean±SD [minutes]	178.80±68.7	n/a
**Stroke subtype, n (%)**		n/a
CE	2(22.2%)	n/a
LAA	4(44.4%)	n/a
SVO	3(33.3%)	n/a

Other etiology	0(0.0%)	n/a
**Score, mean±SD**		n/a
NIHSS	8.67±7.0	n/a
mRS	1.89±1.85	n/a
ASPECT	7.33±2.11	n/a

* s.e.m >Mean,as for one of the patients was massive cerebral infarction, the ICA occlusion;

Abbreviation: SD, standard deviation; TIA, transient ischemic attack; MI, myocardial infarction; AST,aspartate transaminase; ALT, alanine transaminase; LDL, low-density lipoprotein; HDL, high-density lipoprotein; ACE I, angiotensin-converting enzyme inhibitor; ARBs, angiotensin II receptor blockers; ΔT, time from symptom onset until hospital arrival; n/a, not available; ASPECT,Alberta Stroke Program Early CT Score, 8of patients were obseved by MRI, one of patients was observed by CT in hyperacute phase without significant damage.

Figure supplementary 1

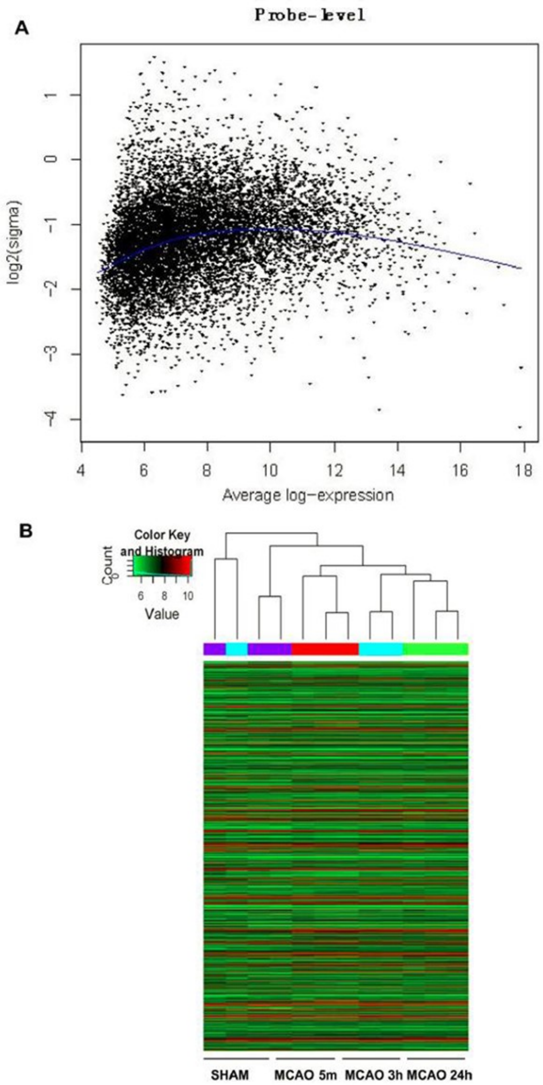

Figure supplementary 1. Quality control of the microarray probes and the heatmap of distinguishable circRNA expression profiling. A. Each dot represents a probe. Y-axis shows the log of variance of a probe intensity among replicates. X-axis shows the average log intensity of the probe's brightness (i.e. expression level). B. The result of hierarchical clustering shows distinguishable circRNA expression profiles among 12 samples of three biological replicates containing SHAM, MCAO 5min, MCAO 3h and MCAO 24h groups.

3 Supplementary Figures and Tables

Supplementary Table S1. List of primers used for qPCR.

name	Forward primer	Reverse Primer
mmu_circ_35605	CTGGCTTCCAGGAGTGAGAC	CGAACAACCAAAGGGACAG
mmu_circ_26021	CACTGTCCCTTCAGGAGAGC	TCCCCTGTGGTATCCTCTTG
mmu_circ_32190	TTGCCCGTTACTACCTGACC	CCAAGGACATGAGCATAGCA
mmu_circ_41878	GCGGCTATGGAAGATGACTC	CCACGCTCCACTATGTCTGA
mmu_circ_34491	TGCTGTTGTGTGGGCTGTAT	ACCACCTTCCTGTGCAATTC
mmu_circ_003795	CCTTAGCACCTGCCTTCTTG	CAGCTGCATCTTCACACACA
mmu_mrna_35605	AGGATTGAGAAGGCAGCAAA	TCTGGCTTCGTCTTGACCTT
mmu_mrna_26021	GCTGGTGGACCATACATCCT	ATCACCGCCAATGAGATAGC
mmu_mrna_32190	GCTGGGTACCATGCCTTCTA	CCACACAGGGTGTGATGAAG
mmu_mrna_41878	GCCTTCACTGGCTACTCTGG	CACTCCACTTCGCAGCATTA
mmu_mrna_34491	GAATTGCACAGGAAGGTGGT	TCAAAGTTAGGCCAGGATGG
mmu_mrna_003795	CTTTGGGCACCACCTAAAAA	AACGTTTGAAGGCCAAAATG
mmu_circ_40203	AGTGGCCATCATCCTCTTTG	GGGATGGTTGCTGAGGAGTA
mmu_circ_45876	CCAGCCCACTACTCATTGCT	CCAGGTGAGCAGACTGTGAA
mmu_circ_24344	AAATGGCGATGTGAAAGAGG	TGACTCCGTCGTTTTGACAG
mmu_circ_37699	AAGAACGCGTGCTGATACCT	GGGGGTCCAAAGACTTCAAT
mmu_circ_38328	GGAATGGAAACCCTGGAACT	CCTCGTCAGCAGCTCTTCTT
mmu_circ_26316	GCAGCAAGGCTATCTCCAAG	ACCTCTGTGTGGAGGTGGAC
mmu_mrna_40203	GACGTGTGGTTTCCCTCACT	CATCCCAGGGAAAGAGATGA
mmu_mrna_45876	GGGTGGGGGACTATGAAGAT	TTGCAAACTAGGCAGCAATG
mmu_mrna_24344	CTGTCAAAACGACGGAGTCA	GAGCACGCTCAATTTCAACA
mmu_mrna_37699	AAGAACGCGTGCTGATACCT	GGGGGTCCAAAGACTTCAAT

mmu_mrna_38328	ATGACCAATCCGGAGAACAG	TCTGGCTCATCCCCATATTC
mmu_mrna_26316	GCAAAGAAGGCTTTGTCCTG	TTCAATGCTTGCCTCAAGTG
mmu_circ_43956	GGGGAAGAGAGAGGGAGAGA	CCTGCTCAGTGCAACCAGTA
mmu_circ_37286	TTGCAGGAGTCCTTGCTTCT	GGGTTTCCTCTTCCCATCTC
mmu_circ_40795	AGGGAAGTCCCCAAATCAAC	AGGCTTCATGCCTACCTTCA
mmu_circ_003795	CCTTAGCACCTGCCTTCTTG	CAGCTGCATCTTCACACACA
mmu_mrna_43956	CCCTCAAATAGCCACGTGTT	TGCATTGCCTTTAAGCCTCT
mmu_mrna_37286	CTGTGGCCTTACCGTGTTTT	GGCTTCACGAGGACAGAGAC
mmu_mrna_40795	CAGAACGCATGTGCTGAGAT	ACCTCTTGGCCAGATTTCCT
mmu_circ_42362	CTGGCAGAGGGTGTTCAGTT	AGGCACCTCTGCAAGAGAGA
mmu_circ_43344	GTATGGCAGTCGGTTTGGTT	GAGGGCTCTGGAACACTCTG
mmu_circ_45921	ATCGGTGATCCACGTCCTAC	TATCCTCATCACGGTCAGCA
hsa_circ_0020231	CTGGCAGAGGGTGTTCAGTT	AGGCACCTCTGCAAGAGAGA
hsa_circ_0039457	ATGGCAGTCGGTTTGGTTAC	GAGGGCTCTGGAACACTCTG
has_circ_0090002	ATCGGTGATCCACGTCCTAC	TATCCTCATCACGGTCAGCA
Fgf1	CAGAGCAGACAGAGTGCTGAG	GCTGCAGTAGAGCAGTTTGG
Nf2	ACCTTTCACTTCCTGGCCAAA	GGAGGAATTCCTCTTGGGCT
Ppp2a	AAGGTTCGTTACCGAGAGCG	GCTCGGATGTGATCCAGTGT
Trp73	CTAGCCACAGAGCCTCAACC	CATGGGGTCACCGACGTAAA
Yap1	CCCTCGTTTTGCCATGAACC	TCCGTATTGCCTGCCGAAAT
Bmp2	CACCCCAAGACACAGTTCCC	AACACTAGAAGACAGCGGGTC
Gli2	GCCTGAATAGACTGTGGGTGAA	TGCACCAAATTTACTGCCTGGA
Gcnt2	AATGGAGCTGGGATGGAGGA	TAGGCGTGTGAAGGGTTTCC
Fut9	ATCCCATGCGGTCCTGATTC	AAGGGATTTGTGCTCACCGT

Fut1	ACCAGCAAGGAGCTCAGACTA	GGCATGCATCTCAGGTTGGA
Hadha	ATTGTGGTCAAGGACGGACC	ACATCCACACCCACTTCGTC
Elovl5	CAGCTTGCTTCTGTTCCCG	GTCCAGGAGGAACCATCCTTT
Acox1	ATTTTGTGGAACCTGTTGGCCT	CCTCGAAGATGAGTTCCGTGG
Ehhadh	ACATCAGAGGCGCAGGATAC	ATGGCTCTAACCGTATGGTCC
β-actin (mouse)	CGTTGACATCCGTAAAGACC	AACAGTCCGCCTAGAAGCAC
β-actin (human)	CGACAGGATGCAGAAGGAG	ACATCTGCTGGAAGGTGGA

Supplementary Table S2. List of top 10 up- and down-regulated differentially expressed circRNAs in the three time points.

Group	ProbeName	adj.P.Val	GeneSymbol	Type	logFC
MCAO 5 min	mmu_circRNA_35065	0.0287	Dido1	exonic	2.48
MCAO 5 min	mmu_circRNA_42362	0.0468	Wdr11	exonic	2.4
MCAO 5 min	mmu_circRNA_011174	0.0145	NA	sense overlapping	2.35
MCAO 5 min	mmu_circRNA_35587	0.0144	Gm20754	intronic	2.22
MCAO 5 min	mmu_circRNA_35588	0.0246	Gm20754	intronic	2.19
MCAO 5 min	mmu_circRNA_43955	0.047	Ets1	exonic	2.17
MCAO 5 min	mmu_circRNA_26021	0.0353	Ryr2	exonic	2.09
MCAO 5 min	mmu_circRNA_32190	0.0145	Slc29a2	exonic	2.07
MCAO 5 min	mmu_circRNA_20066	0.0157	Gm553	sense overlapping	2.03
MCAO 5 min	mmu_circRNA_35589	0.0167	Gm20754	intronic	2.01
MCAO 5 min	mmu_circRNA_010803	0.0151	Raf1	exonic	-3.08
MCAO 5 min	mmu_circRNA_43867	0.0018	Bbs9	exonic	-3.12
MCAO 5 min	mmu_circRNA_25433	0.0195	Pcnx	exonic	-3.15
MCAO 5 min	mmu_circRNA_45921	0.0144	Phka2	exonic	-3.16
MCAO 5 min	mmu_circRNA_22898	0.0198	Zpbp	exonic	-3.18
MCAO 5 min	mmu_circRNA_003169	0.0075	Arhgap10	exonic	-3.18
MCAO 5 min	mmu_circRNA_43344	0.0197	Bbs2	exonic	-3.27
MCAO 5 min	mmu_circRNA_003795	0.0145	Cep350	sense overlapping	-3.28
MCAO 5 min	mmu_circRNA_34491	0.0145	Sppl2a	exonic	-3.3
MCAO 5 min	mmu_circRNA_41878	0.0145	Ankrd42	exonic	-4.07
MCAO 3 h	mmu_circRNA_40203	0.005	Tmem178b	sense overlapping	4.49
MCAO 3 h	mmu_circRNA_36481	0.005	Rad54b	exonic	4.27

MCAO 3 h	mmu_circRNA_45876	0.005	Cnksr2	exonic	4.21
MCAO 3 h	mmu_circRNA_24344	0.005	Psmd12	exonic	4.17
MCAO 3 h	mmu_circRNA_27197	0.005	Samd8	exonic	4.15
MCAO 3 h	mmu_circRNA_010777	0.005	Kcnma1	exonic	4.01
MCAO 3 h	mmu_circRNA_27565	0.006	Tm9sf1	exonic	3.93
MCAO 3 h	mmu_circRNA_43956	0.005	Kirrel3	intronic	3.89
MCAO 3 h	mmu_circRNA_19178	0.005	Ermard	sense overlapping	3.79
MCAO 3 h	mmu_circRNA_29760	0.005	Spice1	exonic	3.79
MCAO 3 h	mmu_circRNA_38234	0.012	5031425E22Rik	exonic	-2
MCAO 3 h	mmu_circRNA_38234	0.012	LOC102635844	exonic	-2
MCAO 3 h	mmu_circRNA_38760	0.007	Corin	exonic	-2.07
MCAO 3 h	mmu_circRNA_26316	0.045	LOC102631805	intergenic	-2.1
MCAO 3 h	mmu_circRNA_38328	0.006	Rbm33	exonic	-2.26
MCAO 3 h	mmu_circRNA_23643	0.047	Spns2	exonic	-2.42
MCAO 3 h	mmu_circRNA_43344	0.041	Bbs2	exonic	-2.52
MCAO 3 h	mmu_circRNA_45921	0.008	Phka2	exonic	-2.83
MCAO 3 h	mmu_circRNA_37699	0.043	Khdrbs1	exonic	-3.03
MCAO 3 h	mmu_circRNA_002246	0.018	Igf2	sense overlapping	-3.04
MCAO 24 h	mmu_circRNA_43956	0	Kirrel3	intronic	7.461
MCAO 24 h	mmu_circRNA_37286	0	Dab1	intronic	6.79
MCAO 24 h	mmu_circRNA_40203	0	Tmem178b	sense overlapping	6.28
MCAO 24 h	mmu_circRNA_31169	0	NA	intergenic	5.85
MCAO 24 h	mmu_circRNA_19077	0	Adck1	intronic	5.79
MCAO 24 h	mmu_circRNA_45876	0	Cnksr2	exonic	5.72

MCAO 24 h	mmu_circRNA_45047	0	Manf	antisense	5.59
MCAO 24 h	mmu_circRNA_24344	0	Psmd12	exonic	5.55
MCAO 24 h	mmu_circRNA_22526	0	NA	sense overlapping	5.51
MCAO 24 h	mmu_circRNA_30884	0	Xdh	exonic	5.38
MCAO 24 h	mmu_circRNA_37699	0.001	Khdrbs1	exonic	-4.29
MCAO 24 h	mmu_circRNA_35175	0.001	Spata16	exonic	-4.3
MCAO 24 h	mmu_circRNA_39121	0	Evi5	exonic	-4.35
MCAO 24 h	mmu_circRNA_45921	0	Phka2	exonic	-4.42
MCAO 24 h	mmu_circRNA_003795	0	Cep350	sense overlapping	-4.51
MCAO 24 h	mmu_circRNA_002246	0	Igf2	sense overlapping	-4.67
MCAO 24 h	mmu_circRNA_010803	0	Raf1	exonic	-4.73
MCAO 24 h	mmu_circRNA_40795	0	Tmcc1	exonic	-4.77
MCAO 24 h	mmu_circRNA_015506	0	Crim1	exonic	-5.7
MCAO 24 h	mmu_circRNA_41878	0	Ankrd42	exonic	-6.57

Supplementary Table S3. List of significnatly differentially expressed circRNAs.

Groups	circRNA	chrom	strand	txStart	txEnd	circRNA_type	logFC	AveExpr	t	P.Value	adj.P.Val	GeneSymbol
MCAO 5m *v.s.* SHAM	mmu_circRNA_001149	chr12	+	52937134	52938506	exonic	-2.887	9.67614	-6.89	1.01E-05	0.011299	Akap6
MCAO 5m *v.s.* SHAM	mmu_circRNA_001579	chr3	-	56079858	56091120	exonic	-2.954	7.59162	-4.61	0.00046898	0.026424	Nbea
MCAO 5m *v.s.* SHAM	mmu_circRNA_001729	chr9	-	96591949	96611499	exonic	-2.054	11.2543	-5.05	0.0002133	0.019755	Rasa2
MCAO 5m v.s. SHAM	mmu_circRNA_001848	chr11	+	70874745	70884968	exonic	-2.106	10.3305	-3.78	0.0022453	0.046084	Rabep1
MCAO 5m v.s. SHAM	mmu_circRNA_002352	chr1	+	139039999	139062971	exonic	-2.003	7.85573	-4.33	0.00078792	0.032538	Dennd1b
MCAO 5m v.s. SHAM	mmu_circRNA_002644	chrX	-	98626913	98644994	exonic	-2.021	7.007	-5.41	0.00011272	0.015405	Ophn1
MCAO 5m v.s. SHAM	mmu_circRNA_003169	chr8	-	77310730	77365160	exonic	-3.181	10.5202	-7.74	2.89E-06	0.007452	Arhgap10
MCAO 5m v.s. SHAM	mmu_circRNA_003795	chr1	-	155847109	155848727	sense overlapping	-3.276	11.5818	-5.76	6.25E-05	0.014544	Cep350
MCAO 5m v.s. SHAM	mmu_circRNA_004072	chr10	+	84947411	84948020	exonic	-3.071	10.2347	-6.08	3.65E-05	0.014378	Ric8b
MCAO 5m v.s. SHAM	mmu_circRNA_004868	chr10	-	12439626	12455564	exonic	-2.535	10.1668	-5.49	9.74E-05	0.015375	Utrn
MCAO 5m v.s. SHAM	mmu_circRNA_004975	chr15	+	95912304	95920378	exonic	-2.393	8.56951	-5.82	5.57E-05	0.014544	Ano6
MCAO 5m v.s. SHAM	mmu_circRNA_005547	chr1	-	53256628	53290423	exonic	-2.021	8.77203	-4	0.0014534	0.038915	Pms1
MCAO 5m v.s. SHAM	mmu_circRNA_006097	chr2	+	92214048	92230724	exonic	-2.523	11.7045	-5.31	0.00013308	0.016192	Phf21a
MCAO 5m v.s. SHAM	mmu_circRNA_006404	chr9	+	22643744	22679064	exonic	-2.39	9.96291	-5.84	5.42E-05	0.014544	Bbs9
MCAO 5m v.s. SHAM	mmu_circRNA_007853	chr10	+	14004200	14086082	sense overlapping	-2.285	12.3794	-4.8	0.00032944	0.023034	Hivep2
MCAO 5m v.s. SHAM	mmu_circRNA_007906	chr3	+	122747888	122748477	exonic	-2.403	11.1222	-4.05	0.00133141	0.038322	Pde5a
MCAO 5m v.s. SHAM	mmu_circRNA_008057	chr9	-	66540061	66566903	exonic	-2.686	8.84259	-4.34	0.0007715	0.032274	Usp3
MCAO 5m v.s. SHAM	mmu_circRNA_009056	chr10	-	12436280	12455564	exonic	-2.378	11.0566	-4.38	0.00072002	0.031661	Utrn
MCAO 5m v.s. SHAM	mmu_circRNA_009396	chr6	+	31418930	31433131	exonic	-2.774	11.6581	-5.02	0.00022486	0.019786	Mkln1

MCAO 5m v.s. SHAM	mmu_circRNA_009558	chr1	+	139053345	139062971	exonic	-2.092	9.44197	-3.94	0.00163453	0.040578	Dennd1b
MCAO 5m v.s. SHAM	mmu_circRNA_009674	chr5	+	34342521	34350838	exonic	-2.063	10.0777	-3.65	0.00285335	0.04976	Rnf4
MCAO 5m v.s. SHAM	mmu_circRNA_010337	chr3	-	153411461	153411871	exonic	-2.056	11.2999	-5.29	0.00013802	0.016192	St6galnac3
MCAO 5m v.s. SHAM	mmu_circRNA_010803	chr6	-	115634552	115644642	exonic	-3.079	11.5318	-5.56	8.65E-05	0.015139	Raf1
MCAO 5m v.s. SHAM	mmu_circRNA_011174	chr17	-	39846503	39846663	sense overlapping	2.345	6.2875	5.753	6.27E-05	0.014544	NA
MCAO 5m v.s. SHAM	mmu_circRNA_012978	chr17	-	24624819	24625014	intronic	-2.502	13.128	-3.84	0.00200336	0.044575	Tsc2
MCAO 5m v.s. SHAM	mmu_circRNA_013636	chr9	+	8634046	8658377	exonic	-2.797	11.3865	-5.55	8.89E-05	0.015142	Trpc6
MCAO 5m v.s. SHAM	mmu_circRNA_013908	chr3	+	122794259	122818067	exonic	-2.506	9.68296	-5.93	4.68E-05	0.014378	4930447N08Rik
MCAO 5m v.s. SHAM	mmu_circRNA_013908	chr3	+	122794259	122818067	exonic	-2.506	9.68296	-5.93	4.68E-05	0.014378	Pde5a
MCAO 5m v.s. SHAM	mmu_circRNA_014005	chr3	+	59037559	59093695	exonic	-2.681	12.0878	-4.38	0.00072101	0.031661	Med12l
MCAO 5m v.s. SHAM	mmu_circRNA_015239	chr12	+	4961217	5006791	exonic	-2.452	9.42344	-4.93	0.0002648	0.021192	Atad2b
MCAO 5m v.s. SHAM	mmu_circRNA_015986	chr8	-	77344639	77365160	exonic	-2.153	10.3786	-5.46	0.000103	0.015375	Arhgap10
MCAO 5m v.s. SHAM	mmu_circRNA_016182	chr9	+	80087429	80103764	exonic	-2.011	10.4465	-5.18	0.00016713	0.018163	Senp6
MCAO 5m v.s. SHAM	mmu_circRNA_016331	chr2	+	92356863	92359209	exonic	-2.439	7.66482	-5.64	7.63E-05	0.014544	Phf21a
MCAO 5m v.s. SHAM	mmu_circRNA_017784	chr6	+	31426713	31433131	exonic	-2.245	9.69551	-4.56	0.0005152	0.027703	Mkln1
MCAO 5m v.s. SHAM	mmu_circRNA_18953	chr1	-	119741351	119765923	sense overlapping	-2.065	9.77486	-4.35	0.00076299	0.032274	Ptpn4
MCAO 5m v.s. SHAM	mmu_circRNA_18967	chr10	-	12436280	12455565	sense overlapping	-2.363	10.9141	-4.34	0.00077528	0.032274	Utrn
MCAO 5m v.s. SHAM	mmu_circRNA_18979	chr10	-	52544603	52545267	sense overlapping	-3.069	10.788	-6.49	1.88E-05	0.011608	Zfx
MCAO 5m v.s. SHAM	mmu_circRNA_19038	chr11	+	95319176	95332371	sense overlapping	-2.244	8.91513	-5.16	0.00017416	0.018506	NA
MCAO 5m v.s. SHAM	mmu_circRNA_19252	chr2	+	92214045	92230725	sense overlapping	-2.576	11.7163	-5.66	7.31E-05	0.014544	Phf21a

MCAO 5m v.s. SHAM	mmu_circRNA_19295	chr3	-	94519045	94531392	sense overlapping	-2.113	8.59456	-4.14	0.00112962	0.03662	Snx27
MCAO 5m v.s. SHAM	mmu_circRNA_19311	chr3	-	153411461	153411874	sense overlapping	-2.096	11.059	-5.69	6.93E-05	0.014544	St6galnac3
MCAO 5m v.s. SHAM	mmu_circRNA_19390	chr5	+	130187399	130198117	sense overlapping	-2.014	9.175	-4.68	0.00041207	0.025818	Rabgef1
MCAO 5m v.s. SHAM	mmu_circRNA_19411	chr6	+	31418929	31433132	sense overlapping	-2.78	11.6827	-4.91	0.00027016	0.021253	Mkln1
MCAO 5m v.s. SHAM	mmu_circRNA_19525	chr9	-	66540061	66566907	sense overlapping	-2.623	7.86135	-4.84	0.00030801	0.022856	Usp3
MCAO 5m v.s. SHAM	mmu_circRNA_19573	chr1	+	5089008	5150061	exonic	-2.123	8.21024	-4.14	0.00113459	0.03662	Atp6v1h
MCAO 5m v.s. SHAM	mmu_circRNA_19729	chr1	-	21424048	21469564	exonic	-3.038	8.21758	-5.31	0.00013356	0.016192	Kcnq5
MCAO 5m v.s. SHAM	mmu_circRNA_20066	chr1	+	52522221	52523151	sense overlapping	2.034	12.4253	5.384	0.00011771	0.015687	Gm553
MCAO 5m v.s. SHAM	mmu_circRNA_20290	chr1	-	66721804	66778108	exonic	-2.057	12.1603	-4.32	0.00079969	0.032693	Kansl1l
MCAO 5m v.s. SHAM	mmu_circRNA_20764	chr1	-	124633364	124639139	intronic	-2.375	8.72334	-4.58	0.00050033	0.02733	NA
MCAO 5m v.s. SHAM	mmu_circRNA_21247	chr1	+	167830545	167832894	exonic	-2.781	7.25257	-5.35	0.00012476	0.015901	Lmx1a
MCAO 5m v.s. SHAM	mmu_circRNA_21668	chr10	-	12455362	12688427	exonic	-2.181	11.1013	-5.2	0.00016214	0.017993	Utrn
MCAO 5m v.s. SHAM	mmu_circRNA_22800	chr10	-	128578099	128579092	exonic	-2.513	11.4194	-5.43	0.00010836	0.015375	Erbb3
MCAO 5m v.s. SHAM	mmu_circRNA_22898	chr11	-	11352521	11418287	exonic	-3.179	8.3935	-5.04	0.00021431	0.019755	Zpbp
MCAO 5m v.s. SHAM	mmu_circRNA_23166	chr11	+	31055457	31058999	exonic	-2.164	8.8165	-5.8	5.79E-05	0.014544	Asb3
MCAO 5m v.s. SHAM	mmu_circRNA_23845	chr11	+	80220257	80227332	exonic	-2.022	8.92519	-4.35	0.00076153	0.032274	Rhot1
MCAO 5m v.s. SHAM	mmu_circRNA_24828	chr12	+	28847556	28867153	sense overlapping	-2.024	7.80646	-5.01	0.00022628	0.019786	Tssc1
MCAO 5m v.s. SHAM	mmu_circRNA_25433	chr12	+	81946930	81967148	exonic	-3.15	7.14595	-5.12	0.00018673	0.019473	Pcnx
MCAO 5m v.s. SHAM	mmu_circRNA_26021	chr13	-	11868116	11885538	exonic	2.088	9.09262	4.205	0.00099527	0.035258	Ryr2
MCAO 5m v.s. SHAM	mmu_circRNA_26033	chr13	+	13651576	13696788	exonic	-2.729	8.77711	-7.33	5.24E-06	0.009839	Lyst
MCAO 5m v.s. SHAM	mmu_circRNA_26040	chr13	+	13725813	13761201	exonic	-2.515	7.61351	-4.67	0.00041955	0.025818	Lyst

MCAO 5m v.s. SHAM	mmu_circRNA_26205	chr13	-	36877742	36918635	exonic	-2.283	8.25587	-5.95	4.52E-05	0.014378	Gm30489
MCAO 5m v.s. SHAM	mmu_circRNA_26205	chr13	-	36877742	36918635	exonic	-2.283	8.25587	-5.95	4.52E-05	0.014378	F13a1
MCAO 5m v.s. SHAM	mmu_circRNA_26206	chr13	-	36892805	36918635	exonic	-2.412	8.9522	-6.53	1.78E-05	0.011608	F13a1
MCAO 5m v.s. SHAM	mmu_circRNA_26206	chr13	-	36892805	36918635	exonic	-2.412	8.9522	-6.53	1.78E-05	0.011608	Gm30489
MCAO 5m v.s. SHAM	mmu_circRNA_26316	chr13	+	49820612	49830616	intergenic	-2.533	8.79094	-4.62	0.00046426	0.026424	LOC102631805
MCAO 5m v.s. SHAM	mmu_circRNA_26948	chr13	-	107769559	107788009	exonic	-2.934	7.4385	-6.94	9.42E-06	0.011299	Zswim6
MCAO 5m v.s. SHAM	mmu_circRNA_27715	chr14	-	64896649	64897126	exonic	-2.304	7.57109	-3.73	0.00244005	0.046838	Hmbox1
MCAO 5m v.s. SHAM	mmu_circRNA_27968	chr14	-	103247746	103282802	exonic	-2.264	6.91609	-7.17	6.67E-06	0.009839	Mycbp2
MCAO 5m v.s. SHAM	mmu_circRNA_29302	chr16	+	15662982	15690411	exonic	-3.7	9.07667	-4.06	0.00144573	0.048506	Prkdc
MCAO 5m v.s. SHAM	mmu_circRNA_29357	chr16	+	17257334	17259073	sense overlapping	-2.523	9.29371	-6.31	2.52E-05	0.012943	Hic2
MCAO 5m v.s. SHAM	mmu_circRNA_29619	chr16	+	35267179	35283609	exonic	-2.415	12.8924	-6.17	3.17E-05	0.014244	Adcy5
MCAO 5m v.s. SHAM	mmu_circRNA_29699	chr16	+	38170663	38193994	exonic	-2.795	8.57752	-7.22	6.19E-06	0.009839	Gsk3b
MCAO 5m v.s. SHAM	mmu_circRNA_29747	chr16	-	44005459	44013037	exonic	-2.376	7.10252	-8.22	1.49E-06	0.007323	Gramd1c
MCAO 5m v.s. SHAM	mmu_circRNA_29750	chr16	-	44028678	44040158	intergenic	-2.352	6.54705	-5.92	4.74E-05	0.014378	NA
MCAO 5m v.s. SHAM	mmu_circRNA_30386	chr17	-	27596723	27596834	exonic	-2.43	7.11418	-3.7	0.00262187	0.047805	Nudt3
MCAO 5m v.s. SHAM	mmu_circRNA_30941	chr17	+	75225155	75227241	exonic	-2.6	11.6544	-4.65	0.00043997	0.026209	Ltbp1
MCAO 5m v.s. SHAM	mmu_circRNA_32017	chr18	+	77873360	77875919	exonic	-2.057	9.4629	-5.02	0.00022411	0.019786	Pstpip2
MCAO 5m v.s. SHAM	mmu_circRNA_32190	chr19	+	5027024	5027764	exonic	2.068	11.2703	5.608	8.02E-05	0.014544	Slc29a2
MCAO 5m v.s. SHAM	mmu_circRNA_32545	chr19	+	37576935	37609105	exonic	-2.096	8.04767	-4.53	0.0005451	0.028279	Exoc6
MCAO 5m v.s. SHAM	mmu_circRNA_32884	chr2	-	5354668	5362127	exonic	-2.919	11.6701	-5.42	0.00011021	0.015375	Camk1d

MCAO 5m v.s. SHAM	mmu_circRNA_33003	chr2	+	14981305	14984439	exonic	-2.866	11.6408	-6.26	2.71E-05	0.012943	Cacnb2
MCAO 5m v.s. SHAM	mmu_circRNA_33850	chr2	+	71875405	71888994	exonic	-2.397	10.0574	-6.51	1.84E-05	0.011608	Pdk1
MCAO 5m v.s. SHAM	mmu_circRNA_33851	chr2	+	71880038	71883932	exonic	-2.671	10.0236	-5.67	7.17E-05	0.014544	Pdk1
MCAO 5m v.s. SHAM	mmu_circRNA_33860	chr2	-	73141897	73156913	exonic	-2.13	6.49504	-5.84	5.41E-05	0.014544	Ola1
MCAO 5m v.s. SHAM	mmu_circRNA_34079	chr2	+	92221471	92230724	exonic	-2.454	8.97882	-5.65	7.41E-05	0.014544	Phf21a
MCAO 5m v.s. SHAM	mmu_circRNA_34491	chr2	-	126919638	126926999	exonic	-3.301	9.25167	-5.74	6.47E-05	0.014544	Sppl2a
MCAO 5m v.s. SHAM	mmu_circRNA_35065	chr2	-	180683808	180692048	exonic	2.481	11.3105	4.504	0.00056988	0.028734	Dido1
MCAO 5m v.s. SHAM	mmu_circRNA_35175	chr3	+	26878562	26927389	exonic	-2.718	7.13891	-3.67	0.00278057	0.04958	Spata16
MCAO 5m v.s. SHAM	mmu_circRNA_35587	chr3	+	73277865	73333103	intronic	2.217	8.97047	5.991	4.22E-05	0.014378	Gm20754
MCAO 5m v.s. SHAM	mmu_circRNA_35588	chr3	+	73277865	73339144	intronic	2.19	9.01322	4.743	0.00036788	0.024632	Gm20754
MCAO 5m v.s. SHAM	mmu_circRNA_35589	chr3	+	73277865	73341175	intronic	2.013	9.34021	5.261	0.00014587	0.016733	Gm20754
MCAO 5m v.s. SHAM	mmu_circRNA_35991	chr3	+	109508783	109563204	exonic	-2.063	10.4304	-4.41	0.00067361	0.031401	Vav3
MCAO 5m v.s. SHAM	mmu_circRNA_36096	chr3	+	122774552	122795321	exonic	-2.547	10.7618	-6.04	3.88E-05	0.014378	LOC108168896
MCAO 5m v.s. SHAM	mmu_circRNA_36096	chr3	+	122774552	122795321	exonic	-2.547	10.7618	-6.04	3.88E-05	0.014378	4930447N08Rik
MCAO 5m v.s. SHAM	mmu_circRNA_36096	chr3	+	122774552	122795321	exonic	-2.547	10.7618	-6.04	3.88E-05	0.014378	Pde5a
MCAO 5m v.s. SHAM	mmu_circRNA_36265	chr3	+	145124210	145132817	exonic	-2.116	8.05362	-4.08	0.00126226	0.037773	Odf2l
MCAO 5m v.s. SHAM	mmu_circRNA_36266	chr3	+	145124210	145144416	exonic	-2.242	8.143	-4.35	0.00076182	0.032274	Odf2l
MCAO 5m v.s. SHAM	mmu_circRNA_36800	chr4	+	53716779	53737659	sense overlapping	-2.381	11.9328	-6.82	1.12E-05	0.011299	Fktn
MCAO 5m v.s. SHAM	mmu_circRNA_37370	chr4	+	108486453	108503035	exonic	-2.014	7.09874	-5.68	7.11E-05	0.014544	Zcchc11
MCAO 5m v.s. SHAM	mmu_circRNA_37852	chr4	-	140067136	140157573	exonic	-2.653	11.488	-7.96	2.13E-06	0.007323	Igsf21
MCAO 5m v.s. SHAM	mmu_circRNA_38199	chr5	-	21029775	21031725	intronic	-2.172	7.91492	-4.19	0.00102735	0.035316	Ptpn12

MCAO 5m v.s. SHAM	mmu_circRNA_38721	chr5	-	67398199	67400301	sense overlapping	-2.312	5.91948	-4.83	0.00031417	0.022967	Bend4
MCAO 5m v.s. SHAM	mmu_circRNA_38722	chr5	-	67398199	67405519	sense overlapping	-2.48	11.0972	-4.1	0.00121193	0.037349	Bend4
MCAO 5m v.s. SHAM	mmu_circRNA_38722	chr5	-	67398199	67405519	sense overlapping	-2.48	11.0972	-4.1	0.00121193	0.037349	Gm33345
MCAO 5m v.s. SHAM	mmu_circRNA_38764	chr5	-	72782002	72789244	exonic	-2.411	7.53191	-6.1	3.51E-05	0.014378	Tec
MCAO 5m v.s. SHAM	mmu_circRNA_38765	chr5	-	72823455	72825130	exonic	-2.687	8.7828	-4.98	0.00023848	0.020139	Tec
MCAO 5m v.s. SHAM	mmu_circRNA_38889	chr5	-	84233582	84265639	intronic	-2.453	6.79666	-4.59	0.00048524	0.026881	Epha5
MCAO 5m v.s. SHAM	mmu_circRNA_38977	chr5	+	92170672	92194175	exonic	-2.324	7.83906	-5.59	8.20E-05	0.0146	Uso1
MCAO 5m v.s. SHAM	mmu_circRNA_38999	chr5	-	96091583	96134132	exonic	-2.13	7.77935	-4.45	0.00063439	0.0306	Cnot6l
MCAO 5m v.s. SHAM	mmu_circRNA_40539	chr6	+	87904087	87905267	exonic	-2.915	6.6404	-4.64	0.00044417	0.026209	Copg1
MCAO 5m v.s. SHAM	mmu_circRNA_40598	chr6	-	97323654	97329564	exonic	-2.75	9.04445	-6.78	1.20E-05	0.011299	Frmd4b
MCAO 5m v.s. SHAM	mmu_circRNA_40722	chr6	+	108813626	108818613	exonic	-2.104	9.06452	-4.07	0.00127397	0.037903	Arl8b
MCAO 5m v.s. SHAM	mmu_circRNA_40722	chr6	+	108813626	108818613	exonic	-2.104	9.06452	-4.07	0.00127397	0.037903	Gm35417
MCAO 5m v.s. SHAM	mmu_circRNA_40924	chr6	+	125793556	125815599	exonic	-2.141	6.92836	-5.3	0.00013567	0.016192	Ano2
MCAO 5m v.s. SHAM	mmu_circRNA_41210	chr7	+	19186101	19186371	exonic	-2.174	12.8742	-3.85	0.00193966	0.043819	Eml2
MCAO 5m v.s. SHAM	mmu_circRNA_41589	chr7	+	64512980	64553771	sense overlapping	-2.158	6.89811	-3.69	0.00263475	0.047805	Apba2
MCAO 5m v.s. SHAM	mmu_circRNA_41878	chr7	-	92584465	92631662	exonic	-4.071	10.4762	-5.77	6.09E-05	0.014544	Ankrd42
MCAO 5m v.s. SHAM	mmu_circRNA_42362	chr7	+	129629917	129632421	exonic	2.401	10.6303	3.738	0.00241912	0.046838	Wdr11
MCAO 5m v.s. SHAM	mmu_circRNA_42666	chr8	-	24878720	24902884	exonic	-2.665	7.23952	-5.34	0.0001265	0.015927	Adam32
MCAO 5m v.s. SHAM	mmu_circRNA_43161	chr8	+	81715200	81770924	exonic	-2.343	11.0896	-5.54	9.05E-05	0.015142	Inpp4b
MCAO 5m v.s. SHAM	mmu_circRNA_43168	chr8	+	82010538	82071835	exonic	-2.347	8.50999	-4.55	0.00052843	0.027834	Inpp4b

MCAO 5m v.s. SHAM	mmu_circRNA_43246	chr8	+	85946051	85957654	exonic	-2.273	7.35741	-6.25	2.76E-05	0.012943	Phkb
MCAO 5m v.s. SHAM	mmu_circRNA_43344	chr8	-	94086655	94092622	exonic	-3.271	6.19788	-5.07	0.00020278	0.019681	Bbs2
MCAO 5m v.s. SHAM	mmu_circRNA_43741	chr9	+	8609699	8610474	exonic	-2.402	6.71172	-5.5	9.69E-05	0.015375	Trpc6
MCAO 5m v.s. SHAM	mmu_circRNA_43742	chr9	+	8643505	8658377	exonic	-2.455	10.7079	-4.89	0.00028424	0.021737	Trpc6
MCAO 5m v.s. SHAM	mmu_circRNA_43867	chr9	+	22638729	22670957	exonic	-3.117	8.76555	-9.9	1.77E-07	0.001828	Bbs9
MCAO 5m v.s. SHAM	mmu_circRNA_43955	chr9	+	32732948	32738359	exonic	2.168	9.77374	3.728	0.00246539	0.04696	Ets1
MCAO 5m v.s. SHAM	mmu_circRNA_44092	chr9	+	46123216	46155540	exonic	-2.062	7.47221	-6.4	2.19E-05	0.011873	Sik3
MCAO 5m v.s. SHAM	mmu_circRNA_44440	chr9	-	64725501	64728429	exonic	-2.274	10.1907	-5.87	5.16E-05	0.014544	Rab11a
MCAO 5m v.s. SHAM	mmu_circRNA_44528	chr9	-	66540061	66547955	exonic	-2.286	7.871	-4.3	0.00083796	0.03302	Usp3
MCAO 5m v.s. SHAM	mmu_circRNA_44742	chr9	+	83569568	83577621	sense overlapping	-2.163	8.28082	-4.84	0.00030849	0.022856	Sh3bgrl2
MCAO 5m v.s. SHAM	mmu_circRNA_44743	chr9	+	83577435	83597526	sense overlapping	-2.424	8.28884	-5.1	0.00019353	0.019478	Sh3bgrl2
MCAO 5m v.s. SHAM	mmu_circRNA_44966	chr9	+	101100570	101124015	sense overlapping	-2.112	10.7835	-4.74	0.00036816	0.024632	Ppp2r3a
MCAO 5m v.s. SHAM	mmu_circRNA_45392	chrX	+	20376880	20377546	exonic	-2.662	8.78223	-4.77	0.00035269	0.023955	Rp2
MCAO 5m v.s. SHAM	mmu_circRNA_45393	chrX	+	20376880	20381819	exonic	-2.285	8.85704	-3.96	0.0015981	0.040143	Rp2
MCAO 5m v.s. SHAM	mmu_circRNA_45921	chrX	+	160523271	160533077	exonic	-3.157	12.4019	-6.06	3.78E-05	0.014378	Phka2
MCAO 3h v.s. SHAM	mmu_circRNA_39116	chr5	+	107490662	107496053	sense overlapping	2.317	9.44385	5.077	0.00020188	0.010743	NA
MCAO 3h v.s. SHAM	mmu_circRNA_30518	chr17	+	45562001	45562400	sense overlapping	2.695	10.6643	4.733	0.00037458	0.015797	Nfkbie
MCAO 3h v.s. SHAM	mmu_circRNA_19217	chr19	-	4288591	4294928	sense overlapping	3.17	11.0486	5.515	9.40E-05	0.007856	Grk2
MCAO 3h v.s. SHAM	mmu_circRNA_23522	chr11	-	65053098	65067156	exonic	2.042	7.0033	5.633	7.69E-05	0.007353	Arhgap44
MCAO 3h v.s. SHAM	mmu_circRNA_19178	chr17	-	14988387	15044490	sense overlapping	3.786	13.1034	6.543	1.73E-05	0.005435	Ermard
MCAO 3h v.s. SHAM	mmu_circRNA_19178	chr17	-	14988387	15044490	sense overlapping	3.786	13.1034	6.543	1.73E-05	0.005435	Gm35343

MCAO 3h v.s. SHAM	mmu_circRNA_43833	chr9	+	21923439	21923863	antisense	2.413	8.15057	5.399	0.00011485	0.008099	Tmem205
MCAO 3h v.s. SHAM	mmu_circRNA_27242	chr14	-	26422420	26470400	exonic	2.212	8.65704	4.677	0.0004147	0.0164	Slmap
MCAO 3h v.s. SHAM	mmu_circRNA_36726	chr4	+	45992196	46018616	exonic	2.712	10.2689	6.142	3.30E-05	0.005435	Tdrd7
MCAO 3h v.s. SHAM	mmu_circRNA_31307	chr18	+	11905924	11926364	exonic	2.383	10.0866	6.489	1.89E-05	0.005435	Cables1
MCAO 3h v.s. SHAM	mmu_circRNA_37769	chr4	-	134148009	134156371	exonic	2.631	10.1983	6.091	3.58E-05	0.005435	Cep85
MCAO 3h v.s. SHAM	mmu_circRNA_40916	chr6	+	124703226	124703627	exonic	2.149	7.83643	6.295	2.57E-05	0.005435	Lpcat3
MCAO 3h v.s. SHAM	mmu_circRNA_37267	chr4	-	103048209	103096775	exonic	2.41	10.0703	4.92	0.00026703	0.013004	Wdr78
MCAO 3h v.s. SHAM	mmu_circRNA_41362	chr7	+	46427345	46428279	exonic	2.282	10.3476	5.805	5.75E-05	0.006637	Kcnc1
MCAO 3h v.s. SHAM	mmu_circRNA_39875	chr6	-	18378366	18423981	exonic	2.081	9.81301	4.102	0.00120953	0.032948	Cttnbp2
MCAO 3h v.s. SHAM	mmu_circRNA_38639	chr5	-	62466745	62467049	intergenic	2.015	5.82599	5.11	0.00019049	0.010406	NA
MCAO 3h v.s. SHAM	mmu_circRNA_31230	chr18	-	7265000	7285792	exonic	2.275	8.901	6.662	1.44E-05	0.005435	Armc4
MCAO 3h v.s. SHAM	mmu_circRNA_43900	chr9	+	25594560	25634351	sense overlapping	2.466	7.75305	4.958	0.00024964	0.012332	Eepd1
MCAO 3h v.s. SHAM	mmu_circRNA_45876	chrX	-	157888397	157945416	exonic	4.214	9.30168	6.374	2.27E-05	0.005435	Cnksr2
MCAO 3h v.s. SHAM	mmu_circRNA_010498	chr2	-	143938457	143938574	antisense	3.316	12.0558	5.712	6.72E-05	0.006805	Dstn
MCAO 3h v.s. SHAM	mmu_circRNA_32218	chr19	-	7456299	7467867	exonic	3.004	11.3789	6.071	3.70E-05	0.005435	Rtn3
MCAO 3h v.s. SHAM	mmu_circRNA_26394	chr13	-	57512691	57513356	sense overlapping	2.511	11.9974	6.675	1.41E-05	0.005435	Spock1
MCAO 3h v.s. SHAM	mmu_circRNA_38328	chr5	+	28331110	28352643	exonic	-2.263	7.20658	-5.89	4.96E-05	0.006294	Rbm33
MCAO 3h v.s. SHAM	mmu_circRNA_45000	chr9	+	103160819	103167192	sense overlapping	2.465	7.88522	7.845	2.50E-06	0.005435	Rab6b
MCAO 3h v.s. SHAM	mmu_circRNA_30190	chr17	+	6016265	6019478	exonic	2.902	9.77447	6.367	2.29E-05	0.005435	Synj2
MCAO 3h v.s. SHAM	mmu_circRNA_40075	chr6	+	35189854	35224217	exonic	2.033	10.6563	4.353	0.00075476	0.024124	Nup205

MCAO 3h v.s. SHAM	mmu_circRNA_43988	chr9	-	36923583	36987272	exonic	2.079	11.5488	3.834	0.00201362	0.044707	Pknox2
MCAO 3h v.s. SHAM	mmu_circRNA_37766	chr4	-	133535509	133541216	exonic	2.575	8.53074	4.211	0.00098522	0.028571	Nudc
MCAO 3h v.s. SHAM	mmu_circRNA_30189	chr17	+	5990135	5996961	exonic	2.599	6.80978	7.079	7.61E-06	0.005435	Synj2
MCAO 3h v.s. SHAM	mmu_circRNA_36522	chr4	-	20469439	20505935	sense overlapping	2.306	9.03996	5.252	0.00014834	0.009453	Nkain3
MCAO 3h v.s. SHAM	mmu_circRNA_30666	chr17	-	57218564	57218671	intronic	2.079	10.6724	7.151	6.83E-06	0.005435	C3
MCAO 3h v.s. SHAM	mmu_circRNA_35606	chr3	-	75298105	75339532	intergenic	2.682	10.5375	6.388	2.22E-05	0.005435	Wdr49
MCAO 3h v.s. SHAM	mmu_circRNA_25821	chr12	-	108862802	108867777	exonic	2.003	7.83539	4.905	0.00027458	0.013309	Wars
MCAO 3h v.s. SHAM	mmu_circRNA_44586	chr9	-	71515068	71555737	exonic	2.881	8.69819	4.213	0.0009802	0.028571	Myzap
MCAO 3h v.s. SHAM	mmu_circRNA_22930	chr11	-	20306465	20320234	exonic	2.747	12.5212	6.222	2.89E-05	0.005435	Slc1a4
MCAO 3h v.s. SHAM	mmu_circRNA_26594	chr13	+	77245200	77281152	exonic	2.084	9.87178	4.689	0.000406	0.016328	2210408I21Rik
MCAO 3h v.s. SHAM	mmu_circRNA_27197	chr14	+	21774962	21780181	exonic	4.146	12.5543	6.206	2.97E-05	0.005435	Samd8
MCAO 3h v.s. SHAM	mmu_circRNA_22817	chr11	-	3711693	3712053	exonic	2.445	8.43872	4.394	0.00069939	0.022706	Osbp2
MCAO 3h v.s. SHAM	mmu_circRNA_45523	chrX	-	74035347	74080032	sense overlapping	2.068	8.21785	4.277	0.00086943	0.026374	Mecp2
MCAO 3h v.s. SHAM	mmu_circRNA_21880	chr10	-	41922121	41951746	exonic	2.609	10.4083	6.666	1.43E-05	0.005435	Armc2
MCAO 3h v.s. SHAM	mmu_circRNA_45149	chr9	+	110571946	110604231	exonic	2.457	8.1666	4.315	0.0008104	0.025171	Setd2
MCAO 3h v.s. SHAM	mmu_circRNA_41166	chr7	-	4430393	4435098	exonic	2.388	11.0901	6.679	1.40E-05	0.005435	Rdh13
MCAO 3h v.s. SHAM	mmu_circRNA_31317	chr18	+	12684836	12698059	exonic	2.25	10.9156	5.135	0.00018232	0.010214	Ttc39c
MCAO 3h v.s. SHAM	mmu_circRNA_36481	chr4	+	11593620	11605005	exonic	4.267	12.1502	6.207	2.96E-05	0.005435	Rad54b
MCAO 3h v.s. SHAM	mmu_circRNA_24344	chr11	+	107485549	107489072	exonic	4.168	9.89977	6.77	1.22E-05	0.005435	Psmd12
MCAO 3h v.s. SHAM	mmu_circRNA_38760	chr5	-	72329611	72343117	exonic	-2.069	7.15788	-5.77	6.14E-05	0.006637	Corin
MCAO 3h v.s. SHAM	mmu_circRNA_21120	chr1	+	159861209	159868285	exonic	2.239	9.45099	4.798	0.00033288	0.01475	Tnr

MCAO 3h v.s. SHAM	mmu_circRNA_43201	chr8	-	84707659	84713857	sense overlapping	2.057	7.28274	6.205	2.97E-05	0.005435	Nfix
MCAO 3h v.s. SHAM	mmu_circRNA_38590	chr5	-	51472597	51474411	exonic	2.341	11.8476	6.042	3.88E-05	0.005483	Ppargc1a
MCAO 3h v.s. SHAM	mmu_circRNA_29760	chr16	+	44355439	44377022	exonic	3.73	11.9177	6.856	1.07E-05	0.005435	Spice1
MCAO 3h v.s. SHAM	mmu_circRNA_40611	chr6	-	99162838	99669567	sense overlapping	2.519	11.8573	5.889	5.00E-05	0.006294	NA
MCAO 3h v.s. SHAM	mmu_circRNA_43263	chr8	-	86724205	86725856	sense overlapping	2.426	13.3906	5.784	5.96E-05	0.006637	Siah1a
MCAO 3h v.s. SHAM	mmu_circRNA_30884	chr17	-	73906009	73907745	exonic	3.334	9.06227	5.332	0.00012889	0.008697	Xdh
MCAO 3h v.s. SHAM	mmu_circRNA_35962	chr3	-	107445429	107448440	exonic	2.967	12.5675	6.647	1.47E-05	0.005435	Kcnc4
MCAO 3h v.s. SHAM	mmu_circRNA_26316	chr13	+	49820612	49830616	intergenic	-2.096	8.79094	-3.82	0.00207082	0.045383	LOC102631805
MCAO 3h v.s. SHAM	mmu_circRNA_23881	chr11	+	83321938	83336861	exonic	2.964	11.8823	6.502	1.85E-05	0.005435	Ap2b1
MCAO 3h v.s. SHAM	mmu_circRNA_010147	chr19	-	37068172	37088587	exonic	2.864	11.8228	6.234	2.84E-05	0.005435	Cpeb3
MCAO 3h v.s. SHAM	mmu_circRNA_44088	chr9	-	45943050	45944486	exonic	3.094	11.2441	6.581	1.63E-05	0.005435	Sidt2
MCAO 3h v.s. SHAM	mmu_circRNA_22088	chr10	-	62274446	62286652	exonic	2.263	9.9047	4.377	0.00072147	0.023132	Hk1
MCAO 3h v.s. SHAM	mmu_circRNA_42273	chr7	+	122754505	122762323	exonic	3.032	9.65921	6.056	3.79E-05	0.005435	Cacng3
MCAO 3h v.s. SHAM	mmu_circRNA_42362	chr7	+	129629917	129632421	exonic	3.133	10.6303	4.878	0.00028844	0.013682	Wdr11
MCAO 3h v.s. SHAM	mmu_circRNA_26137	chr13	-	24036184	24094570	exonic	2.25	9.85198	5.552	8.82E-05	0.007856	Carmil1
MCAO 3h v.s. SHAM	mmu_circRNA_41406	chr7	+	49780308	49829030	exonic	3.031	9.0492	6.457	1.99E-05	0.005435	Prmt3
MCAO 3h v.s. SHAM	mmu_circRNA_29484	chr16	-	30545125	30547469	exonic	2.236	10.6491	4.275	0.00087275	0.026374	Tmem44
MCAO 3h v.s. SHAM	mmu_circRNA_40590	chr6	-	94654846	94663998	exonic	3.286	12.122	6.991	8.69E-06	0.005435	Lrig1
MCAO 3h v.s. SHAM	mmu_circRNA_42581	chr8	+	14305203	14314292	intronic	2.204	7.54848	5.423	0.00011005	0.008021	Dlgap2
MCAO 3h v.s. SHAM	mmu_circRNA_27287	chr14	+	27449945	27459851	exonic	3.562	12.2679	6.218	2.91E-05	0.005435	Fam208a

MCAO 3h v.s. SHAM	mmu_circRNA_27821	chr14	+	77268806	77441899	exonic	3.51	10.955	6.304	2.54E-05	0.005435	Enox1
MCAO 3h v.s. SHAM	mmu_circRNA_31671	chr18	+	42288107	42305814	exonic	2.354	10.5446	5.493	9.76E-05	0.007856	Rbm27
MCAO 3h v.s. SHAM	mmu_circRNA_30586	chr17	+	50711410	50718783	intergenic	3.708	10.1033	5.26	0.00014624	0.009378	NA
MCAO 3h v.s. SHAM	mmu_circRNA_27546	chr14	-	51905612	51905724	sense overlapping	3.088	8.02883	4.747	0.00036527	0.015521	Ndrg2
MCAO 3h v.s. SHAM	mmu_circRNA_38545	chr5	+	43709043	43714601	exonic	2.365	10.5431	5.821	5.60E-05	0.006637	Cc2d2a
MCAO 3h v.s. SHAM	mmu_circRNA_22663	chr10	-	116343842	116346937	antisense	2.987	9.56817	5.756	6.25E-05	0.006637	Ptprb
MCAO 3h v.s. SHAM	mmu_circRNA_27893	chr14	+	79596747	79597466	exonic	2.456	10.324	5.523	9.28E-05	0.007856	Sugt1
MCAO 3h v.s. SHAM	mmu_circRNA_27264	chr14	+	26900422	26934532	sense overlapping	2.789	7.76428	5.308	0.00013438	0.008887	Appl1
MCAO 3h v.s. SHAM	mmu_circRNA_27026	chr14	+	8028236	8039904	exonic	2.74	8.79435	4.66	0.00042802	0.016607	Abhd6
MCAO 3h v.s. SHAM	mmu_circRNA_19110	chr14	-	19904158	19904277	intronic	3.125	11.1538	6.453	2.00E-05	0.005435	Gng2
MCAO 3h v.s. SHAM	mmu_circRNA_28026	chr14	+	118153568	118160184	exonic	2.356	8.81652	5.198	0.00016307	0.00972	Gpr180
MCAO 3h v.s. SHAM	mmu_circRNA_22790	chr10	+	128207297	128208667	exonic	2.709	7.95375	4.289	0.00085048	0.025977	Gls2
MCAO 3h v.s. SHAM	mmu_circRNA_41481	chr7	-	59284025	59317973	sense overlapping	2.13	9.50828	5.718	6.65E-05	0.006802	Snhg14
MCAO 3h v.s. SHAM	mmu_circRNA_002246	chr7	-	142651460	142651527	sense overlapping	-3.042	7.52559	-4.57	0.00050509	0.018169	Igf2
MCAO 3h v.s. SHAM	mmu_circRNA_19314	chr4	-	3080977	3084320	intergenic	2.804	10.6166	5.923	4.72E-05	0.006166	NA
MCAO 3h v.s. SHAM	mmu_circRNA_006741	chr6	-	119052600	119057515	exonic	2.013	8.357	5.728	6.55E-05	0.006759	Cacna1c
MCAO 3h v.s. SHAM	mmu_circRNA_22526	chr10	+	99402065	99402880	sense overlapping	2.957	8.75673	5.959	4.45E-05	0.005893	NA
MCAO 3h v.s. SHAM	mmu_circRNA_41238	chr7	-	27541084	27542181	exonic	2.132	10.0306	5.49	9.80E-05	0.007856	Pld3
MCAO 3h v.s. SHAM	mmu_circRNA_34860	chr2	+	156664798	156711261	exonic	2.289	9.01726	6.355	2.34E-05	0.005435	Dlgap4
MCAO 3h v.s. SHAM	mmu_circRNA_19015	chr11	+	53893644	53981397	sense overlapping	3.571	10.4419	5.749	6.31E-05	0.006637	Gm30927
MCAO 3h v.s. SHAM	mmu_circRNA_45137	chr9	-	110432433	110439058	exonic	2.081	10.1696	4.663	0.0004257	0.016585	Klhl18

MCAO 3h v.s. SHAM	mmu_circRNA_28632	chr15	+	62054793	62107465	intronic	2.169	10.8688	4.723	0.00038143	0.016008	Pvt1
MCAO 3h v.s. SHAM	mmu_circRNA_23261	chr11	-	46221335	46224205	exonic	3.01	10.9546	7.094	7.43E-06	0.005435	Cyfip2
MCAO 3h v.s. SHAM	mmu_circRNA_41378	chr7	-	46743993	46750514	intergenic	2.517	9.74998	6.251	2.76E-05	0.005435	NA
MCAO 3h v.s. SHAM	mmu_circRNA_39371	chr5	+	123934071	123936555	exonic	3.133	9.61083	5.123	0.00018608	0.010273	Ccdc62
MCAO 3h v.s. SHAM	mmu_circRNA_37770	chr4	-	134148009	134172880	exonic	2.706	9.51011	4.671	0.00041955	0.016407	Cep85
MCAO 3h v.s. SHAM	mmu_circRNA_20054	chr1	-	52189422	52207359	exonic	2.018	7.59221	4.572	0.0005031	0.018161	Gls
MCAO 3h v.s. SHAM	mmu_circRNA_001874	chr9	+	120453526	120464961	exonic	2.474	9.54261	5.011	0.00022694	0.011656	Myrip
MCAO 3h v.s. SHAM	mmu_circRNA_013002	chr16	+	94403313	94468060	sense overlapping	2.35	10.6294	5.078	0.00020148	0.010743	Ttc3
MCAO 3h v.s. SHAM	mmu_circRNA_40913	chr6	+	122828156	122833876	exonic	2.033	12.9587	6.281	2.63E-05	0.005435	Foxj2
MCAO 3h v.s. SHAM	mmu_circRNA_38234	chr5	-	23384145	23385172	exonic	2.001	12.1188	4.975	0.00024214	0.012135	5031425E22Rik
MCAO 3h v.s. SHAM	mmu_circRNA_38234	chr5	-	23384145	23385172	exonic	2.001	12.1188	4.975	0.00024214	0.012135	LOC102635844
MCAO 3h v.s. SHAM	mmu_circRNA_003170	chr2	+	18101457	18126229	exonic	2.621	11.6332	5.392	0.00011611	0.008099	Mllt10
MCAO 3h v.s. SHAM	mmu_circRNA_31773	chr18	+	56728400	56733337	exonic	2.115	12.9694	4.861	0.00029715	0.013925	Lmnb1
MCAO 3h v.s. SHAM	mmu_circRNA_002314	chr13	+	8672404	8731971	exonic	2.828	8.09707	4.647	0.00043864	0.016772	Adarb2
MCAO 3h v.s. SHAM	mmu_circRNA_29156	chr16	+	5234965	5239166	exonic	2.654	11.4155	6.114	3.45E-05	0.005435	Alg1
MCAO 3h v.s. SHAM	mmu_circRNA_015541	chr2	+	37508927	37541176	exonic	3.498	9.68183	5.052	0.00021104	0.011047	Strbp
MCAO 3h v.s. SHAM	mmu_circRNA_015541	chr2	+	37508927	37541176	exonic	3.498	9.68183	5.052	0.00021104	0.011047	Rabgap1
MCAO 3h v.s. SHAM	mmu_circRNA_38761	chr5	-	72353869	72360758	exonic	2.827	9.91956	5.335	0.00012832	0.008697	Corin
MCAO 3h v.s. SHAM	mmu_circRNA_28834	chr15	-	81323609	81338087	exonic	2.099	13.0963	4.245	0.00092305	0.027463	Slc25a17
MCAO 3h v.s. SHAM	mmu_circRNA_013526	chr17	-	90560740	90597629	exonic	2.374	7.42759	3.843	0.00198022	0.044155	Nrxn1

MCAO 3h v.s. SHAM	mmu_circRNA_011301	chr14	+	13995006	14013655	sense overlapping	2.227	10.4371	4.332	0.00078497	0.024707	NA
MCAO 3h v.s. SHAM	mmu_circRNA_27565	chr14	-	55640384	55641588	exonic	3.925	10.9835	5.961	4.44E-05	0.005893	Tm9sf1
MCAO 3h v.s. SHAM	mmu_circRNA_27592	chr14	+	56887794	56919342	exonic	2.873	7.43915	4.828	0.00031522	0.014336	Zmym2
MCAO 3h v.s. SHAM	mmu_circRNA_28652	chr15	-	64783723	64815418	exonic	2.47	8.14097	4.454	0.00062551	0.021454	Adcy8
MCAO 3h v.s. SHAM	mmu_circRNA_25585	chr12	+	88850278	88853112	intronic	2.169	12.3907	4.751	0.0003624	0.015521	Nrxn3
MCAO 3h v.s. SHAM	mmu_circRNA_002563	chr18	+	22375132	22434675	exonic	2.604	11.7048	5.393	0.00011593	0.008099	Asxl3
MCAO 3h v.s. SHAM	mmu_circRNA_24567	chr12	+	3899192	3900361	exonic	2.307	12.9486	5.598	8.15E-05	0.007579	Dnmt3a
MCAO 3h v.s. SHAM	mmu_circRNA_30337	chr17	-	25380224	25381573	exonic	2.333	9.03534	5.807	5.73E-05	0.006637	Cacna1h
MCAO 3h v.s. SHAM	mmu_circRNA_27181	chr14	-	21476670	21483639	antisense	2.216	12.1141	4.688	0.00040646	0.016328	Gm30363
MCAO 3h v.s. SHAM	mmu_circRNA_27181	chr14	-	21476670	21483639	antisense	2.216	12.1141	4.688	0.00040646	0.016328	Kat6b
MCAO 3h v.s. SHAM	mmu_circRNA_24789	chr12	+	21230822	21247373	exonic	2.546	10.8049	5.826	5.55E-05	0.006637	Asap2
MCAO 3h v.s. SHAM	mmu_circRNA_24789	chr12	+	21230822	21247373	exonic	2.546	10.8049	5.826	5.55E-05	0.006637	Gm40855
MCAO 3h v.s. SHAM	mmu_circRNA_42048	chr7	+	105682778	105687385	exonic	2.27	12.6057	6.099	3.53E-05	0.005435	Dnhd1
MCAO 3h v.s. SHAM	mmu_circRNA_34777	chr2	+	152677541	152704941	sense overlapping	2.375	13.5676	5.65	7.47E-05	0.007353	H13
MCAO 3h v.s. SHAM	mmu_circRNA_008457	chr16	-	89877037	89885125	exonic	2.057	12.2258	5.749	6.31E-05	0.006637	Tiam1
MCAO 3h v.s. SHAM	mmu_circRNA_35586	chr3	+	73269612	73341175	intronic	2.356	8.29045	4.971	0.00024367	0.012153	Gm20754
MCAO 3h v.s. SHAM	mmu_circRNA_42423	chr7	+	133650500	133667691	exonic	3.089	11.0595	5.559	8.72E-05	0.007856	Edrf1
MCAO 3h v.s. SHAM	mmu_circRNA_40203	chr6	+	40004144	40207533	sense overlapping	4.494	10.0608	7.275	5.69E-06	0.005435	Tmem178b
MCAO 3h v.s. SHAM	mmu_circRNA_43956	chr9	+	34536915	34538449	intronic	3.887	9.26161	6.78	1.20E-05	0.005435	Kirrel3
MCAO 3h v.s. SHAM	mmu_circRNA_33499	chr2	-	48087107	48095756	sense overlapping	2.662	9.1881	3.899	0.00177806	0.041468	Gm13481
MCAO 3h v.s. SHAM	mmu_circRNA_34968	chr2	-	166617832	166621333	exonic	3.655	11.8013	6.226	2.88E-05	0.005435	Prex1

MCAO 3h v.s. SHAM	mmu_circRNA_41052	chr6	-	144155096	144625686	exonic	2.313	10.6932	6.157	3.22E-05	0.005435	NA
MCAO 3h v.s. SHAM	mmu_circRNA_19470	chr7	-	104315409	104348404	sense overlapping	2.778	12.3217	5.572	8.52E-05	0.007786	Trim5
MCAO 3h v.s. SHAM	mmu_circRNA_36544	chr4	+	22025119	22040595	intergenic	2.523	7.03666	5.062	0.00020722	0.010915	Gm42260
MCAO 3h v.s. SHAM	mmu_circRNA_015492	chr4	+	101612861	101616865	exonic	2.227	12.8653	5.325	0.00013058	0.008754	Dnajc6
MCAO 3h v.s. SHAM	mmu_circRNA_39830	chr6	-	8643972	8658314	exonic	2.185	7.55356	5.838	5.44E-05	0.006637	Ica1
MCAO 3h v.s. SHAM	mmu_circRNA_32047	chr18	-	80808611	80891923	exonic	2.483	12.7347	6.094	3.56E-05	0.005435	Atp9b
MCAO 3h v.s. SHAM	mmu_circRNA_28307	chr15	-	25027225	25364918	sense overlapping	2.367	10.2107	3.933	0.0016677	0.039907	Gm2824
MCAO 3h v.s. SHAM	mmu_circRNA_43907	chr9	+	28039050	28045364	intronic	2.841	11.0118	6.092	3.57E-05	0.005435	Opcml
MCAO 3h v.s. SHAM	mmu_circRNA_37176	chr4	+	95696959	95769529	exonic	2.594	10.5223	4.713	0.00038878	0.01606	Fggy
MCAO 3h v.s. SHAM	mmu_circRNA_45837	chrX	+	152033698	152047968	exonic	2.703	10.4481	7.657	3.26E-06	0.005435	Smc1a
MCAO 3h v.s. SHAM	mmu_circRNA_26486	chr13	+	63156545	63240331	exonic	2.682	8.743	3.812	0.00209816	0.045606	2010111I01Rik
MCAO 3h v.s. SHAM	mmu_circRNA_38907	chr5	+	86891227	86905024	antisense	2.044	13.7169	6.995	8.64E-06	0.005435	Ugt2b34
MCAO 3h v.s. SHAM	mmu_circRNA_29553	chr16	+	32884123	32898470	exonic	2.27	7.95559	4.089	0.00124055	0.033353	Fyttd1
MCAO 3h v.s. SHAM	mmu_circRNA_38525	chr5	+	37278084	37278931	exonic	2.843	8.0808	5.302	0.00013585	0.008887	Crmp1
MCAO 3h v.s. SHAM	mmu_circRNA_42572	chr8	-	13605861	13631892	exonic	3.42	11.1948	5.764	6.16E-05	0.006637	Rasa3
MCAO 3h v.s. SHAM	mmu_circRNA_44389	chr9	-	61924631	61926518	exonic	2.417	11.5111	6.057	3.79E-05	0.005435	Kif23
MCAO 3h v.s. SHAM	mmu_circRNA_22421	chr10	+	90510595	90644556	exonic	2.955	8.96316	4.236	0.00093972	0.027719	Anks1b
MCAO 3h v.s. SHAM	mmu_circRNA_23643	chr11	-	72456302	72474770	exonic	-2.416	8.98581	-3.79	0.00219011	0.046813	Spns2
MCAO 3h v.s. SHAM	mmu_circRNA_001832	chr6	-	82730767	82739674	exonic	3.423	12.3907	7.498	4.10E-06	0.005435	Hk2
MCAO 3h v.s. SHAM	mmu_circRNA_37286	chr4	+	103754180	103764967	intronic	3.067	8.22307	4.234	0.00094287	0.027733	Dab1

MCAO 3h v.s. SHAM	mmu_circRNA_32308	chr19	+	21797326	21818011	exonic	2.384	10.4919	7.236	6.02E-06	0.005435	Tmem2
MCAO 3h v.s. SHAM	mmu_circRNA_20259	chr1	-	64034531	64042437	sense overlapping	2.475	10.5876	5.53	9.16E-05	0.007856	Klf7
MCAO 3h v.s. SHAM	mmu_circRNA_003057	chr18	+	11923359	11926364	exonic	2.269	9.66942	6.78	1.20E-05	0.005435	Cables1
MCAO 3h v.s. SHAM	mmu_circRNA_45921	chrX	+	160523271	160533077	exonic	-2.827	12.4019	-5.42	0.00010991	0.008021	Phka2
MCAO 3h v.s. SHAM	mmu_circRNA_20456	chr1	+	86086029	86090190	exonic	2.759	9.70433	7.33	5.24E-06	0.005435	Psmd1
MCAO 3h v.s. SHAM	mmu_circRNA_22970	chr11	-	22137837	22151892	exonic	3.338	9.42401	4.296	0.00083893	0.025701	Ehbp1
MCAO 3h v.s. SHAM	mmu_circRNA_36483	chr4	-	12057315	12063830	exonic	2.8	13.9213	5.744	6.36E-05	0.006637	Tmem67
MCAO 3h v.s. SHAM	mmu_circRNA_36632	chr4	+	34647785	34656262	exonic	2.305	9.40609	4.016	0.00142342	0.036018	Rars2
MCAO 3h v.s. SHAM	mmu_circRNA_37699	chr4	-	129712103	129730325	exonic	-3.028	12.3654	-3.86	0.00191273	0.042835	Khdrbs1
MCAO 3h v.s. SHAM	mmu_circRNA_010777	chr14	-	23494538	23509900	exonic	4.007	9.39607	7.527	3.93E-06	0.005435	Kcnma1
MCAO 3h v.s. SHAM	mmu_circRNA_41390	chr7	+	48799828	48818720	exonic	2.362	11.2406	7.006	8.49E-06	0.005435	Zdhhc13
MCAO 3h v.s. SHAM	mmu_circRNA_33862	chr2	-	73373614	73374014	exonic	2.473	9.08106	3.855	0.00193565	0.043255	Gm13709
MCAO 3h v.s. SHAM	mmu_circRNA_33862	chr2	-	73373614	73374014	exonic	2.473	9.08106	3.855	0.00193565	0.043255	Gpr155
MCAO 3h v.s. SHAM	mmu_circRNA_43955	chr9	+	32732948	32738359	exonic	2.488	9.77374	4.279	0.00086687	0.026374	Ets1
MCAO 3h v.s. SHAM	mmu_circRNA_010789	chr15	-	96609855	96624169	exonic	2.145	11.0649	5.844	5.38E-05	0.006637	Slc38a1
MCAO 3h v.s. SHAM	mmu_circRNA_017685	chr16	+	5953418	5956863	intronic	2.279	11.6724	5.235	0.00015274	0.009499	Rbfox1
MCAO 3h v.s. SHAM	mmu_circRNA_29001	chr15	-	95229083	95241677	exonic	2.107	10.047	4.148	0.00110784	0.030705	Nell2
MCAO 3h v.s. SHAM	mmu_circRNA_43010	chr8	-	70405258	70409011	exonic	2.208	10.4832	6.163	3.19E-05	0.005435	Crtc1
MCAO 3h v.s. SHAM	mmu_circRNA_30482	chr17	+	34416466	34421987	exonic	2.124	12.0496	6.618	1.54E-05	0.005435	BC051142
MCAO 3h v.s. SHAM	mmu_circRNA_37006	chr4	-	83260881	83271218	exonic	2.254	9.54519	4.841	0.00030788	0.014082	Ttc39b
MCAO 3h v.s. SHAM	mmu_circRNA_38874	chr5	-	84067359	84156536	exonic	2.374	10.7494	5.639	7.61E-05	0.007353	Epha5

MCAO 3h v.s. SHAM	mmu_circRNA_44220	chr9	+	55397740	55400647	exonic	2.292	9.17902	5.444	0.00010616	0.007896	Tmem266
MCAO 3h v.s. SHAM	mmu_circRNA_014406	chr15	+	85359453	85365359	exonic	2.397	9.10438	4.414	0.00067355	0.022472	Atxn10
MCAO 3h v.s. SHAM	mmu_circRNA_21750	chr10	+	21928341	21928836	sense overlapping	2.718	12.9902	6.269	2.68E-05	0.005435	Sgk1
MCAO 3h v.s. SHAM	mmu_circRNA_19780	chr1	+	24383303	24390303	antisense	2.573	6.42785	8.316	1.31E-06	0.005435	Col19a1
MCAO 3h v.s. SHAM	mmu_circRNA_41537	chr7	-	61813261	61916736	exonic	2.045	10.3586	5.514	9.42E-05	0.007856	A230057D06Rik
MCAO 3h v.s. SHAM	mmu_circRNA_32634	chr19	+	43810470	43814814	exonic	2.339	6.89627	4.882	0.00028623	0.013681	Abcc2
MCAO 3h v.s. SHAM	mmu_circRNA_21730	chr10	+	20158760	20159555	intronic	3.373	12.6282	6.154	3.23E-05	0.005435	Map7
MCAO 3h v.s. SHAM	mmu_circRNA_42246	chr7	+	121032747	121057399	sense overlapping	3.265	11.8786	6.121	3.41E-05	0.005435	NA
MCAO 3h v.s. SHAM	mmu_circRNA_001686	chr6	+	61310813	61313835	exonic	2.024	13.6931	6.071	3.70E-05	0.005435	Ccser1
MCAO 3h v.s. SHAM	mmu_circRNA_21755	chr10	-	23138190	23159154	exonic	2.306	13.2622	5.607	8.04E-05	0.007543	Eya4
MCAO 3h v.s. SHAM	mmu_circRNA_19073	chr12	+	81894979	81913476	sense overlapping	2.317	8.00463	5.05	0.00021187	0.011047	Pcnx
MCAO 3h v.s. SHAM	mmu_circRNA_45629	chrX	-	98695998	98714211	exonic	2.776	10.5693	5.591	8.25E-05	0.007607	Ophn1
MCAO 3h v.s. SHAM	mmu_circRNA_20164	chr1	-	59066149	59076722	exonic	2.222	8.62554	5.443	0.00010632	0.007896	Als2cr11
MCAO 3h v.s. SHAM	mmu_circRNA_37285	chr4	+	103754180	103755895	intronic	2.598	9.65288	4.85	0.00030326	0.013977	Dab1
MCAO 3h v.s. SHAM	mmu_circRNA_23477	chr11	+	62283596	62285885	exonic	2.613	9.10737	3.997	0.00147425	0.036442	Ttc19
MCAO 3h v.s. SHAM	mmu_circRNA_22246	chr10	+	75966350	75986140	exonic	3.102	10.6644	5.967	4.39E-05	0.005893	Gm5134
MCAO 3h v.s. SHAM	mmu_circRNA_43344	chr8	-	94086655	94092622	exonic	-2.523	6.19788	-3.91	0.00173021	0.041064	Bbs2
MCAO 3h v.s. SHAM	mmu_circRNA_27734	chr14	-	66187442	66190234	exonic	2.101	9.4989	5.823	5.58E-05	0.006637	Ptk2b
MCAO 3h v.s. SHAM	mmu_circRNA_30556	chr17	-	46882732	46883949	sense overlapping	2.368	9.65751	5.909	4.83E-05	0.006234	NA
MCAO 3h v.s. SHAM	mmu_circRNA_36399	chr4	+	3589146	3598663	exonic	2.614	10.4637	4.01	0.00144039	0.036119	Tgs1

MCAO 3h v.s. SHAM	mmu_circRNA_25951	chr13	+	8559103	8570574	exonic	2.88	9.2851	5.755	6.25E-05	0.006637	Adarb2
MCAO 3h v.s. SHAM	mmu_circRNA_33227	chr2	-	29247827	29248878	sense overlapping	2.966	11.6832	5.482	9.95E-05	0.007856	6530402F18Rik
MCAO 3h v.s. SHAM	mmu_circRNA_27500	chr14	-	40907705	40934285	sense overlapping	2.246	10.731	5.75	6.31E-05	0.006637	Tspan14
MCAO 3h v.s. SHAM	mmu_circRNA_31359	chr18	-	16627567	16629712	exonic	2.196	11.9233	5.08	0.00020069	0.010743	Cdh2
MCAO 3h v.s. SHAM	mmu_circRNA_24243	chr11	+	104456946	104459039	antisense	2.151	11.0241	5.213	0.00015884	0.00959	NA
MCAO 3h v.s. SHAM	mmu_circRNA_26219	chr13	-	38934737	38944654	exonic	2.178	11.0949	4.321	0.00080111	0.024987	Slc35b3
MCAO 3h v.s. SHAM	mmu_circRNA_26526	chr13	-	68668469	68678615	exonic	2.927	12.1565	6.383	2.23E-05	0.005435	Adcy2
MCAO 3h v.s. SHAM	mmu_circRNA_32900	chr2	-	5914809	5923023	sense overlapping	2.853	7.94835	5.215	0.00015813	0.00959	Dhtkd1
MCAO 3h v.s. SHAM	mmu_circRNA_33228	chr2	+	29679132	29689263	exonic	3.025	9.78871	4.721	0.00038299	0.016008	Rapgef1
MCAO 3h v.s. SHAM	mmu_circRNA_008226	chr18	+	22375132	22392852	exonic	2.782	10.9977	5.542	8.98E-05	0.007856	Asxl3
MCAO 3h v.s. SHAM	mmu_circRNA_25584	chr12	+	88431020	88459203	exonic	2.977	12.9812	5.393	0.00011606	0.008099	Adck1
MCAO 3h v.s. SHAM	mmu_circRNA_011505	chr15	+	79114654	79115171	exonic	3.572	10.6939	5.397	0.00011524	0.008099	Micall1
MCAO 3h v.s. SHAM	mmu_circRNA_19995	chr1	-	42660742	42667003	sense overlapping	2.478	7.71634	7.32	5.32E-06	0.005435	Pantr1
MCAO 3h v.s. SHAM	mmu_circRNA_42102	chr7	+	111046762	111050505	exonic	2.414	8.4655	6.611	1.56E-05	0.005435	Ctr9
MCAO 24h v.s. SHAM	mmu_circRNA_21485	chr1	-	182278975	182279718	exonic	-2.036	10.7546	-3.99	0.00150908	0.008777	Degs1
MCAO 24h v.s. SHAM	mmu_circRNA_29986	chr16	-	87420172	87420982	exonic	2.228	10.0205	4.755	0.0003599	0.003188	Ltn1
MCAO 24h v.s. SHAM	mmu_circRNA_39116	chr5	+	107490662	107496053	sense overlapping	4.447	9.44385	9.744	2.12E-07	4.58E-05	NA
MCAO 24h v.s. SHAM	mmu_circRNA_34867	chr2	-	157120481	157126844	exonic	-2.297	6.94402	-5.79	5.92E-05	0.000932	Samhd1
MCAO 24h v.s. SHAM	mmu_circRNA_30518	chr17	+	45562001	45562400	sense overlapping	3.297	10.6643	5.79	5.90E-05	0.000932	Nfkbie
MCAO 24h v.s. SHAM	mmu_circRNA_45522	chrX	-	74035347	74037332	sense overlapping	3.566	8.11784	7.047	7.98E-06	0.000259	Mecp2
MCAO 24h v.s. SHAM	mmu_circRNA_002888	chr12	-	51759218	51761170	exonic	-2.39	11.4477	-4.69	0.00040854	0.003472	Hectd1

MCAO 24h v.s. SHAM	mmu_circRNA_006013	chrX	+	69544408	69545269	exonic	2.427	5.81716	3.203	0.00680652	0.026233	Aff2
MCAO 24h v.s. SHAM	mmu_circRNA_008748	chr17	+	65810537	65816050	exonic	-2.013	7.82485	-6.53	1.78E-05	0.000423	Ppp4r1
MCAO 24h v.s. SHAM	mmu_circRNA_19531	chr9	-	107812210	107812329	intronic	-2.093	6.27046	-4.42	0.00066806	0.004942	Rbm6
MCAO 24h v.s. SHAM	mmu_circRNA_012164	chr13	+	23739382	23739448	sense overlapping	-2.196	9.8743	-4.83	0.00031411	0.002923	Hist1h1c
MCAO 24h v.s. SHAM	mmu_circRNA_31561	chr18	-	34614447	34614797	exonic	-2.86	10.1297	-5.38	0.00011872	0.001524	Brd8
MCAO 24h v.s. SHAM	mmu_circRNA_28074	chr14	+	121973608	121994470	exonic	-2.057	10.3144	-4.65	0.0004398	0.003659	Ubac2
MCAO 24h v.s. SHAM	mmu_circRNA_19217	chr19	-	4288591	4294928	sense overlapping	3.989	11.0486	6.94	9.38E-06	0.000279	Grk2
MCAO 24h v.s. SHAM	mmu_circRNA_21799	chr10	+	29196225	29199280	sense overlapping	2.937	11.2096	8.202	1.53E-06	9.37E-05	Soga3
MCAO 24h v.s. SHAM	mmu_circRNA_32778	chr19	+	55628787	55631286	intergenic	2.066	8.37006	6.008	4.10E-05	0.000734	NA
MCAO 24h v.s. SHAM	mmu_circRNA_35360	chr3	+	54701486	54710472	exonic	-2.824	9.29439	-7.79	2.71E-06	0.000137	Supt20
MCAO 24h v.s. SHAM	mmu_circRNA_27614	chr14	+	57472974	57496279	exonic	-4.131	6.91049	-5.52	9.27E-05	0.001288	Ift88
MCAO 24h v.s. SHAM	mmu_circRNA_008982	chr13	+	18810431	18830793	exonic	2.234	6.51841	5.477	0.00010037	0.001358	Vps41
MCAO 24h v.s. SHAM	mmu_circRNA_40001	chr6	+	30443988	30447477	sense overlapping	-3.22	9.14632	-3.58	0.00327517	0.015412	Klhdc10
MCAO 24h v.s. SHAM	mmu_circRNA_26717	chr13	+	93790038	93807703	exonic	3.332	8.31656	5.759	6.21E-05	0.000962	Arsb
MCAO 24h v.s. SHAM	mmu_circRNA_34233	chr2	-	112886491	112912403	exonic	3.461	8.23847	6.785	1.19E-05	0.000327	Ryr3
MCAO 24h v.s. SHAM	mmu_circRNA_23522	chr11	-	65053098	65067156	exonic	2.704	7.0033	7.46	4.34E-06	0.000173	Arhgap44
MCAO 24h v.s. SHAM	mmu_circRNA_19178	chr17	-	14988387	15044490	sense overlapping	4.424	13.1034	7.645	3.32E-06	0.00015	Ermard
MCAO 24h v.s. SHAM	mmu_circRNA_19178	chr17	-	14988387	15044490	sense overlapping	4.424	13.1034	7.645	3.32E-06	0.00015	Gm35343
MCAO 24h v.s. SHAM	mmu_circRNA_43833	chr9	+	21923439	21923863	antisense	2.649	8.15057	5.927	4.69E-05	0.000802	Tmem205
MCAO 24h v.s. SHAM	mmu_circRNA_27242	chr14	-	26422420	26470400	exonic	2.007	8.65704	4.244	0.00092637	0.006247	Slmap

MCAO 24h v.s. SHAM	mmu_circRNA_37644	chr4	+	127025854	127026227	exonic	-2.26	8.17618	-5.03	0.00022076	0.002297	Sfpq
MCAO 24h v.s. SHAM	mmu_circRNA_36102	chr3	-	122947360	122969249	exonic	-3.202	7.15776	-6.76	1.24E-05	0.000336	Usp53
MCAO 24h v.s. SHAM	mmu_circRNA_41233	chr7	-	27212311	27213061	exonic	2.268	8.76204	5	0.0002316	0.002358	Itpkc
MCAO 24h v.s. SHAM	mmu_circRNA_25332	chr12	+	73967346	73975161	sense overlapping	-2.035	9.99379	-2.91	0.01193313	0.039613	Snapc1
MCAO 24h v.s. SHAM	mmu_circRNA_26221	chr13	+	41008210	41009364	exonic	2.603	9.46183	7.883	2.37E-06	0.000125	Pak1ip1
MCAO 24h v.s. SHAM	mmu_circRNA_21913	chr10	+	43345527	43345974	sense overlapping	-2.043	8.49264	-3.82	0.00207403	0.011026	Pdss2
MCAO 24h v.s. SHAM	mmu_circRNA_013251	chr9	-	89601041	89603394	exonic	-2.338	6.91191	-6.13	3.34E-05	0.000637	AF529169
MCAO 24h v.s. SHAM	mmu_circRNA_37780	chr4	+	134934931	134935464	exonic	-2.273	10.3658	-4.82	0.00031977	0.002952	Syf2
MCAO 24h v.s. SHAM	mmu_circRNA_016017	chr9	+	64178285	64178345	sense overlapping	-3.4	11.05	-9.58	2.60E-07	4.97E-05	Rpl4
MCAO 24h v.s. SHAM	mmu_circRNA_007217	chr13	+	23739400	23739466	sense overlapping	-2.364	10.8611	-5.71	6.75E-05	0.001023	Hist1h1c
MCAO 24h v.s. SHAM	mmu_circRNA_31307	chr18	+	11905924	11926364	exonic	3.415	10.0866	9.299	3.65E-07	5.17E-05	Cables1
MCAO 24h v.s. SHAM	mmu_circRNA_21531	chr1	+	190216581	190218968	sense overlapping	3.411	7.02368	4.707	0.000393	0.00337	NA
MCAO 24h v.s. SHAM	mmu_circRNA_001121	chr6	+	17850356	17855028	exonic	-2.548	9.46733	-8.1	1.75E-06	0.000103	St7
MCAO 24h v.s. SHAM	mmu_circRNA_41718	chr7	+	76318079	76335324	exonic	2.131	10.3335	5.917	4.77E-05	0.000809	Agbl1
MCAO 24h v.s. SHAM	mmu_circRNA_29113	chr15	+	102473284	102478747	exonic	-2.048	7.54428	-5.27	0.00014271	0.001715	Pcbp2
MCAO 24h v.s. SHAM	mmu_circRNA_015638	chr19	-	4784723	4788042	intronic	-2.063	10.7685	-5.12	0.00018622	0.002026	Rbm4
MCAO 24h v.s. SHAM	mmu_circRNA_015638	chr19	-	4784723	4788042	intronic	-2.063	10.7685	-5.12	0.00018622	0.002026	Gm21992
MCAO 24h v.s. SHAM	mmu_circRNA_40180	chr6	+	38559169	38591431	sense overlapping	-2.752	9.14523	-5.96	4.45E-05	0.000776	Luc7l2
MCAO 24h v.s. SHAM	mmu_circRNA_37769	chr4	-	134148009	134156371	exonic	3.941	10.1983	9.124	4.55E-07	5.38E-05	Cep85
MCAO 24h v.s. SHAM	mmu_circRNA_29200	chr16	-	9653395	9663607	exonic	-2.02	7.11965	-3.43	0.00437341	0.019108	Grin2a
MCAO 24h v.s. SHAM	mmu_circRNA_016901	chr17	+	83221789	83222230	exonic	4.253	6.14526	5.814	5.66E-05	0.000906	Pkdcc

MCAO 24h v.s. SHAM	mmu_circRNA_40916	chr6	+	124703226	124703627	exonic	3.136	7.83643	9.188	4.20E-07	5.26E-05	Lpcat3
MCAO 24h v.s. SHAM	mmu_circRNA_010432	chr4	-	151830417	151835883	exonic	-2.997	12.5229	-11.3	3.56E-08	3.37E-05	Camta1
MCAO 24h v.s. SHAM	mmu_circRNA_30848	chr17	-	71273373	71275287	exonic	-2.497	9.01996	-8.57	9.33E-07	7.61E-05	Emilin2
MCAO 24h v.s. SHAM	mmu_circRNA_016773	chr17	+	35834970	35835050	antisense	2.392	14.0589	8.888	6.15E-07	6.28E-05	Tubb5
MCAO 24h v.s. SHAM	mmu_circRNA_36156	chr3	+	132694382	132752669	exonic	2.879	12.6197	10.27	1.15E-07	4.18E-05	Tbck
MCAO 24h v.s. SHAM	mmu_circRNA_41362	chr7	+	46427345	46428279	exonic	3.637	10.3476	9.252	3.88E-07	5.20E-05	Kcnc1
MCAO 24h v.s. SHAM	mmu_circRNA_009970	chr13	-	48590286	48606360	exonic	2.355	7.50204	3.988	0.00150118	0.008736	Ptpdc1
MCAO 24h v.s. SHAM	mmu_circRNA_23349	chr11	+	53397831	53402566	exonic	-2.91	7.25684	-5.82	5.57E-05	0.000898	Aff4
MCAO 24h v.s. SHAM	mmu_circRNA_32258	chr19	+	16176757	16219772	sense overlapping	3.347	7.14132	7.779	2.74E-06	0.000137	Gnaq
MCAO 24h v.s. SHAM	mmu_circRNA_26258	chr13	-	44970045	44993738	exonic	-2.41	6.34477	-6.52	1.80E-05	0.000427	Dtnbp1
MCAO 24h v.s. SHAM	mmu_circRNA_21208	chr1	-	165357428	165429315	exonic	-2.99	10.8925	-9.53	2.73E-07	5.03E-05	Dcaf6
MCAO 24h v.s. SHAM	mmu_circRNA_20282	chr1	+	66521454	66530853	exonic	-3.065	9.39285	-4.9	0.00027932	0.0027	Unc80
MCAO 24h v.s. SHAM	mmu_circRNA_39875	chr6	-	18378366	18423981	exonic	2.031	9.81301	4.003	0.00145753	0.008559	Cttnbp2
MCAO 24h v.s. SHAM	mmu_circRNA_19300	chr3	-	116348451	116366708	sense overlapping	-2.014	9.01274	-5.18	0.00016749	0.001881	Cdc14a
MCAO 24h v.s. SHAM	mmu_circRNA_42809	chr8	+	40454497	40474807	exonic	3.587	6.07654	5.259	0.00014642	0.001736	Zdhhc2
MCAO 24h v.s. SHAM	mmu_circRNA_44100	chr9	-	47129526	47192564	intergenic	2.172	7.46507	6.74	1.28E-05	0.000342	Gm31698
MCAO 24h v.s. SHAM	mmu_circRNA_21244	chr1	+	167314109	167326020	intronic	-2.655	7.73059	-8.75	7.31E-07	6.93E-05	Tmco1
MCAO 24h v.s. SHAM	mmu_circRNA_34924	chr2	-	162135211	162278319	exonic	2.221	6.40497	6.796	1.17E-05	0.000324	Ptprt
MCAO 24h v.s. SHAM	mmu_circRNA_33179	chr2	-	25354467	25354822	exonic	2.074	9.0531	6.363	2.31E-05	0.000495	Dpp7
MCAO 24h v.s. SHAM	mmu_circRNA_003208	chr7	-	116358635	116368189	exonic	3.37	7.06679	9.288	3.70E-07	5.17E-05	Pik3c2a

MCAO 24h v.s. SHAM	mmu_circRNA_30227	chr17	-	10576464	10597281	exonic	2.24	7.88214	5.148	0.0001779	0.001958	Pacrg
MCAO 24h v.s. SHAM	mmu_circRNA_31230	chr18	-	7265000	7285792	exonic	3.379	8.901	9.894	1.77E-07	4.46E-05	Armc4
MCAO 24h v.s. SHAM	mmu_circRNA_32020	chr18	-	78584582	78644801	sense overlapping	2.254	8.11116	4.34	0.00077267	0.00552	NA
MCAO 24h v.s. SHAM	mmu_circRNA_34988	chr2	+	166921969	166941492	exonic	-2.232	12.3804	-5.57	8.60E-05	0.00122	Cse1l
MCAO 24h v.s. SHAM	mmu_circRNA_43806	chr9	-	18617594	18620536	intergenic	2.08	8.68243	4.05	0.00133435	0.008005	Muc16
MCAO 24h v.s. SHAM	mmu_circRNA_33285	chr2	+	32216045	32217335	exonic	-2.062	7.72768	-4.14	0.00112507	0.00712	Prrc2b
MCAO 24h v.s. SHAM	mmu_circRNA_38374	chr5	+	30247462	30257820	exonic	3.133	10.2574	5.859	5.25E-05	0.000865	Selenoi
MCAO 24h v.s. SHAM	mmu_circRNA_32294	chr19	-	18581111	18596401	sense overlapping	-2.1	6.33154	-7.15	6.87E-06	0.000237	Ostf1
MCAO 24h v.s. SHAM	mmu_circRNA_43900	chr9	+	25594560	25634351	sense overlapping	4.713	7.75305	9.474	2.94E-07	5.05E-05	Eepd1
MCAO 24h v.s. SHAM	mmu_circRNA_33288	chr2	-	32802753	32808062	exonic	3.15	7.24823	3.572	0.00333415	0.01559	Stxbp1
MCAO 24h v.s. SHAM	mmu_circRNA_015174	chr7	-	116333180	116333258	intronic	2.976	7.42622	5.538	9.03E-05	0.001265	Rps13
MCAO 24h v.s. SHAM	mmu_circRNA_015174	chr7	-	116333180	116333258	intronic	2.976	7.42622	5.538	9.03E-05	0.001265	Snord14a
MCAO 24h v.s. SHAM	mmu_circRNA_45876	chrX	-	157888397	157945416	exonic	5.717	9.30168	8.647	8.41E-07	7.48E-05	Cnksr2
MCAO 24h v.s. SHAM	mmu_circRNA_40599	chr6	-	97396192	97423524	exonic	-2.191	10.0346	-5.77	6.12E-05	0.000955	Frmd4b
MCAO 24h v.s. SHAM	mmu_circRNA_31968	chr18	+	74569766	74580534	exonic	-2.439	5.77255	-5.73	6.49E-05	0.000993	Myo5b
MCAO 24h v.s. SHAM	mmu_circRNA_26922	chr13	-	106822955	106860937	exonic	-2.18	7.22371	-6.41	2.14E-05	0.000472	Ipo11
MCAO 24h v.s. SHAM	mmu_circRNA_36806	chr4	+	55007532	55023606	exonic	-2.44	8.09533	-5.61	8.03E-05	0.001157	Zfp462
MCAO 24h v.s. SHAM	mmu_circRNA_20105	chr1	-	55014868	55016182	intronic	-2.155	8.67914	-5.27	0.00014415	0.001723	Sf3b1
MCAO 24h v.s. SHAM	mmu_circRNA_010498	chr2	-	143938457	143938574	antisense	4.5	12.0558	7.751	2.86E-06	0.00014	Dstn
MCAO 24h v.s. SHAM	mmu_circRNA_38691	chr5	-	65634171	65666311	exonic	-2.025	7.28969	-5.67	7.24E-05	0.001067	Pds5a
MCAO 24h v.s. SHAM	mmu_circRNA_33808	chr2	-	70738420	70757549	exonic	-2.778	9.62562	-8.65	8.35E-07	7.48E-05	Tlk1

MCAO 24h v.s. SHAM	mmu_circRNA_32218	chr19	-	7456299	7467867	exonic	4.509	11.3789	9.112	4.62E-07	5.38E-05	Rtn3
MCAO 24h v.s. SHAM	mmu_circRNA_26394	chr13	-	57512691	57513356	sense overlapping	3.931	11.9974	10.45	9.28E-08	3.99E-05	Spock1
MCAO 24h v.s. SHAM	mmu_circRNA_38328	chr5	+	28331110	28352643	exonic	-3.15	7.20658	-8.2	1.52E-06	9.37E-05	Rbm33
MCAO 24h v.s. SHAM	mmu_circRNA_45000	chr9	+	103160819	103167192	sense overlapping	4.611	7.88522	14.67	1.47E-09	1.49E-05	Rab6b
MCAO 24h v.s. SHAM	mmu_circRNA_19476	chr7	+	123143745	123162878	sense overlapping	-2.601	9.91528	-6.26	2.74E-05	0.000559	Tnrc6a
MCAO 24h v.s. SHAM	mmu_circRNA_44680	chr9	+	78452763	78465002	exonic	-2.256	6.28311	-5.66	7.32E-05	0.001075	Mto1
MCAO 24h v.s. SHAM	mmu_circRNA_30190	chr17	+	6016265	6019478	exonic	4.462	9.77447	9.789	2.01E-07	4.51E-05	Synj2
MCAO 24h v.s. SHAM	mmu_circRNA_000233	chr6	-	135922871	135923470	exonic	2.048	6.34287	3.386	0.00477187	0.020331	Grin2b
MCAO 24h v.s. SHAM	mmu_circRNA_35991	chr3	+	109508783	109563204	exonic	-2.363	10.4304	-5.06	0.00020976	0.002217	Vav3
MCAO 24h v.s. SHAM	mmu_circRNA_22898	chr11	-	11352521	11418287	exonic	-3.098	8.3935	-4.92	0.0002694	0.002639	Zpbp
MCAO 24h v.s. SHAM	mmu_circRNA_015316	chr14	-	78651680	78653796	exonic	-2.082	8.30615	-4.78	0.0003421	0.003093	Gm30716
MCAO 24h v.s. SHAM	mmu_circRNA_015316	chr14	-	78651680	78653796	exonic	-2.082	8.30615	-4.78	0.0003421	0.003093	Dgkh
MCAO 24h v.s. SHAM	mmu_circRNA_45879	chrX	-	157906669	157996684	exonic	-3.048	8.2722	-4.78	0.00034624	0.003111	Cnksr2
MCAO 24h v.s. SHAM	mmu_circRNA_30683	chr17	-	58066717	58067505	antisense	2.672	6.75121	9.308	3.61E-07	5.17E-05	Cntnap5c
MCAO 24h v.s. SHAM	mmu_circRNA_39766	chr5	+	150825533	150825960	intronic	-2.219	8.34115	-4.52	0.00055377	0.0043	Gm36447
MCAO 24h v.s. SHAM	mmu_circRNA_33661	chr2	+	61720443	61785559	exonic	-2.061	5.26327	-6.6	1.58E-05	0.000393	Psmd14
MCAO 24h v.s. SHAM	mmu_circRNA_40764	chr6	-	113416788	113417411	exonic	2.875	9.96391	8.459	1.08E-06	8.06E-05	Rpusd3
MCAO 24h v.s. SHAM	mmu_circRNA_25428	chr12	+	81894982	81919380	exonic	2.325	12.5121	7.487	4.17E-06	0.00017	Pcnx
MCAO 24h v.s. SHAM	mmu_circRNA_43988	chr9	-	36923583	36987272	exonic	2.681	11.5488	4.944	0.00025588	0.002538	Pknox2
MCAO 24h v.s. SHAM	mmu_circRNA_24855	chr12	+	29866827	29867876	intronic	2.277	6.38462	8.572	9.28E-07	7.61E-05	Myt1l

MCAO 24h v.s. SHAM	mmu_circRNA_43812	chr9	-	20444258	20445897	exonic	-2.345	6.8752	-4.74	0.00037117	0.003247	Zfp26
MCAO 24h v.s. SHAM	mmu_circRNA_25974	chr13	-	8985208	8992815	exonic	-2.509	8.60964	-5.45	0.00010443	0.001398	Gtpbp4
MCAO 24h v.s. SHAM	mmu_circRNA_25973	chr13	-	8985208	8992035	exonic	-3.194	8.28853	-6.13	3.35E-05	0.000639	Gtpbp4
MCAO 24h v.s. SHAM	mmu_circRNA_27674	chr14	+	62346567	62353637	exonic	-2.149	7.92899	-3.65	0.00288145	0.014092	Rnaseh2b
MCAO 24h v.s. SHAM	mmu_circRNA_38897	chr5	-	86016201	86052241	exonic	-2.19	8.45987	-4.54	0.00052889	0.00415	Gtdc1
MCAO 24h v.s. SHAM	mmu_circRNA_35175	chr3	+	26878562	26927389	exonic	-4.304	7.13891	-5.81	5.74E-05	0.000917	Spata16
MCAO 24h v.s. SHAM	mmu_circRNA_37766	chr4	-	133535509	133541216	exonic	5.062	8.53074	8.277	1.38E-06	8.93E-05	Nudc
MCAO 24h v.s. SHAM	mmu_circRNA_26255	chr13	+	44848279	44884880	exonic	-2.09	8.52968	-7.37	4.96E-06	0.000188	Jarid2
MCAO 24h v.s. SHAM	mmu_circRNA_003371	chr6	+	134062063	134066465	intronic	-2.104	12.7065	-5.85	5.37E-05	0.000877	Etv6
MCAO 24h v.s. SHAM	mmu_circRNA_19280	chr3	-	51308049	51326037	sense overlapping	-2.845	12.3806	-7.96	2.12E-06	0.000117	Elf2
MCAO 24h v.s. SHAM	mmu_circRNA_005039	chr3	-	51308049	51326035	exonic	-2.781	12.7021	-7.76	2.84E-06	0.00014	Elf2
MCAO 24h v.s. SHAM	mmu_circRNA_43925	chr9	+	31053239	31054313	exonic	2.195	8.09254	2.819	0.01429942	0.045634	Zbtb44
MCAO 24h v.s. SHAM	mmu_circRNA_22440	chr10	-	91158747	91166069	exonic	-2.172	7.55131	-4.91	0.00027133	0.002652	Tmpo
MCAO 24h v.s. SHAM	mmu_circRNA_30189	chr17	+	5990135	5996961	exonic	3.255	6.80978	8.864	6.34E-07	6.42E-05	Synj2
MCAO 24h v.s. SHAM	mmu_circRNA_45157	chr9	+	111190167	111199835	exonic	-2.739	6.37005	-10.3	1.05E-07	4.16E-05	Lrrfip2
MCAO 24h v.s. SHAM	mmu_circRNA_29958	chr16	+	81432708	81490394	exonic	3.974	5.82206	5.745	6.36E-05	0.000979	Ncam2
MCAO 24h v.s. SHAM	mmu_circRNA_42485	chr7	-	143258341	143259384	sense overlapping	-2.058	5.74412	-5.62	7.91E-05	0.001146	Kcnq1ot1
MCAO 24h v.s. SHAM	mmu_circRNA_42485	chr7	-	143258341	143259384	sense overlapping	-2.058	5.74412	-5.62	7.91E-05	0.001146	Kcnq1
MCAO 24h v.s. SHAM	mmu_circRNA_42579	chr8	+	14305203	14305966	intronic	2.312	7.54871	5.377	0.00011918	0.001529	Dlgap2
MCAO 24h v.s. SHAM	mmu_circRNA_19033	chr11	+	87146931	87149364	intronic	-2.749	10.0048	-7.12	7.14E-06	0.000243	Trim37
MCAO 24h v.s. SHAM	mmu_circRNA_41943	chr7	+	97854435	97871612	exonic	2.716	6.21876	6.286	2.61E-05	0.000543	Pak1

MCAO 24h v.s. SHAM	mmu_circRNA_000113	chr12	-	109603917	109603990	antisense	2.05	8.53112	5.062	0.00020718	0.002196	Rian
MCAO 24h v.s. SHAM	mmu_circRNA_29699	chr16	+	38170663	38193994	exonic	-2.76	8.57752	-7.13	7.08E-06	0.000242	Gsk3b
MCAO 24h v.s. SHAM	mmu_circRNA_40985	chr6	-	136634430	136651950	exonic	-2.8	7.52008	-5.69	6.97E-05	0.001044	Plbd1
MCAO 24h v.s. SHAM	mmu_circRNA_44266	chr9	-	56653599	56653832	exonic	3.642	7.46004	10.35	1.05E-07	4.16E-05	Lingo1
MCAO 24h v.s. SHAM	mmu_circRNA_30550	chr17	-	46824449	46830996	exonic	2.372	8.09717	6.849	1.08E-05	0.000304	Bicral
MCAO 24h v.s. SHAM	mmu_circRNA_20182	chr1	-	60006233	60014018	exonic	2.112	8.74103	6.057	3.79E-05	0.000694	Ica1l
MCAO 24h v.s. SHAM	mmu_circRNA_34829	chr2	-	155708931	155726674	exonic	-2.05	12.4543	-5.28	0.00014233	0.001713	Edem2
MCAO 24h v.s. SHAM	mmu_circRNA_010337	chr3	-	153411461	153411871	exonic	-2.172	11.2999	-5.59	8.24E-05	0.001177	St6galnac3
MCAO 24h v.s. SHAM	mmu_circRNA_012978	chr17	-	24624819	24625014	intronic	-4.292	13.128	-6.58	1.63E-05	0.000402	Tsc2
MCAO 24h v.s. SHAM	mmu_circRNA_39718	chr5	+	150186772	150196735	intronic	2.9	9.76747	8.429	1.12E-06	8.21E-05	Fry
MCAO 24h v.s. SHAM	mmu_circRNA_23135	chr11	-	29831098	29906331	exonic	2.195	6.43987	8.266	1.40E-06	8.93E-05	Eml6
MCAO 24h v.s. SHAM	mmu_circRNA_30666	chr17	-	57218564	57218671	intronic	3.119	10.6724	10.73	6.79E-08	3.89E-05	C3
MCAO 24h v.s. SHAM	mmu_circRNA_016119	chr11	+	93959411	93960695	antisense	2.454	7.25485	6.407	2.15E-05	0.000473	Nme1
MCAO 24h v.s. SHAM	mmu_circRNA_29342	chr16	-	16461593	16490606	exonic	2.077	6.61347	6.248	2.77E-05	0.000564	Fgd4
MCAO 24h v.s. SHAM	mmu_circRNA_018522	chr7	+	126864319	126864661	exonic	2.149	6.66072	8.114	1.72E-06	0.000102	Hirip3
MCAO 24h v.s. SHAM	mmu_circRNA_19277	chr3	-	27620601	27643091	sense overlapping	-3.587	11.2137	-7.04	8.13E-06	0.00026	Fndc3b
MCAO 24h v.s. SHAM	mmu_circRNA_35606	chr3	-	75298105	75339532	intergenic	3.296	10.5375	7.85	2.48E-06	0.00013	Wdr49
MCAO 24h v.s. SHAM	mmu_circRNA_39338	chr5	+	123101610	123104511	exonic	2.188	11.2006	6.487	1.89E-05	0.000437	Tmem120b
MCAO 24h v.s. SHAM	mmu_circRNA_25821	chr12	-	108862802	108867777	exonic	3.896	7.83539	9.539	2.72E-07	5.03E-05	Wars
MCAO 24h v.s. SHAM	mmu_circRNA_28998	chr15	+	94638060	94714994	exonic	3.689	8.20649	4.681	0.000412	0.003495	Tmem117

MCAO 24h v.s. SHAM	mmu_circRNA_44586	chr9	-	71515068	71555737	exonic	4.474	8.69819	6.543	1.73E-05	0.000417	Myzap
MCAO 24h v.s. SHAM	mmu_circRNA_32442	chr19	-	32223508	32352023	exonic	-2.14	10.5569	-6.04	3.91E-05	0.000709	Sgms1
MCAO 24h v.s. SHAM	mmu_circRNA_23218	chr11	-	36063088	36139761	exonic	2.263	9.75593	4.155	0.00109348	0.007003	Tenm2
MCAO 24h v.s. SHAM	mmu_circRNA_25572	chr12	-	86705266	86720663	exonic	2.199	7.91941	4.052	0.00133023	0.007984	Angel1
MCAO 24h v.s. SHAM	mmu_circRNA_22930	chr11	-	20306465	20320234	exonic	3.147	12.5212	7.129	7.05E-06	0.000242	Slc1a4
MCAO 24h v.s. SHAM	mmu_circRNA_26086	chr13	+	15477999	15613884	exonic	2.559	8.12528	5.771	6.09E-05	0.000953	Gli3
MCAO 24h v.s. SHAM	mmu_circRNA_38143	chr5	-	17811180	17835671	exonic	-2.462	8.21369	-3.28	0.00581741	0.02336	Cd36
MCAO 24h v.s. SHAM	mmu_circRNA_37532	chr4	+	120463074	120508212	exonic	-2.226	10.5524	-5.13	0.00018421	0.002015	Scmh1
MCAO 24h v.s. SHAM	mmu_circRNA_015481	chr2	+	4431198	4498073	exonic	2.942	8.0864	6.433	2.06E-05	0.000464	Gm39751
MCAO 24h v.s. SHAM	mmu_circRNA_015481	chr2	+	4431198	4498073	exonic	2.942	8.0864	6.433	2.06E-05	0.000464	Frmd4a
MCAO 24h v.s. SHAM	mmu_circRNA_36149	chr3	+	130475592	130496442	exonic	3.107	7.57463	4.556	0.00051811	0.004096	Col25a1
MCAO 24h v.s. SHAM	mmu_circRNA_33280	chr2	+	32191508	32194996	exonic	2.283	8.94716	5.62	7.85E-05	0.00114	Prrc2b
MCAO 24h v.s. SHAM	mmu_circRNA_31309	chr18	-	12058925	12070131	exonic	2.249	10.647	7.577	3.66E-06	0.000157	Tmem241
MCAO 24h v.s. SHAM	mmu_circRNA_42666	chr8	-	24878720	24902884	exonic	-2.294	7.23952	-4.6	0.00047748	0.003878	Adam32
MCAO 24h v.s. SHAM	mmu_circRNA_018406	chr5	+	16264257	16267475	exonic	2.421	6.48268	5.506	9.54E-05	0.001308	Cacna2d1
MCAO 24h v.s. SHAM	mmu_circRNA_26594	chr13	+	77245200	77281152	exonic	3.409	9.87178	7.672	3.19E-06	0.000146	2210408I21Rik
MCAO 24h v.s. SHAM	mmu_circRNA_43546	chr8	+	117555974	117558116	exonic	-2.266	10.0478	-5.69	6.99E-05	0.001044	Plcg2
MCAO 24h v.s. SHAM	mmu_circRNA_35765	chr3	+	89007141	89026506	exonic	2.602	9.31604	5.549	8.87E-05	0.00125	Ash1l
MCAO 24h v.s. SHAM	mmu_circRNA_38661	chr5	+	64311058	64324609	exonic	2.683	9.55217	7.058	7.85E-06	0.000258	Tbc1d1
MCAO 24h v.s. SHAM	mmu_circRNA_24895	chr12	-	32874945	32875233	intergenic	2.558	10.8179	7.382	4.85E-06	0.000186	NA
MCAO 24h v.s. SHAM	mmu_circRNA_003119	chr2	+	104608870	104609186	exonic	-2.196	11.3148	-6.41	2.14E-05	0.000472	Cstf3

MCAO 24h v.s. SHAM	mmu_circRNA_24053	chr11	+	88049411	88050095	exonic	-2.865	10.0034	-6.19	3.03E-05	0.000603	Srsf1
MCAO 24h v.s. SHAM	mmu_circRNA_27197	chr14	+	21774962	21780181	exonic	4.622	12.5543	6.919	9.69E-06	0.000285	Samd8
MCAO 24h v.s. SHAM	mmu_circRNA_41532	chr7	-	61786069	61814190	intronic	2.575	6.94104	9.109	4.64E-07	5.38E-05	A230057D06Rik
MCAO 24h v.s. SHAM	mmu_circRNA_009934	chr5	-	8399687	8405838	exonic	-2.047	8.03429	-6.92	9.63E-06	0.000284	Dbf4
MCAO 24h v.s. SHAM	mmu_circRNA_23058	chr11	+	24652459	24723339	sense overlapping	2.877	7.94183	7.466	4.29E-06	0.000172	NA
MCAO 24h v.s. SHAM	mmu_circRNA_41104	chr6	-	148782597	148812156	exonic	2.556	6.24501	8.243	1.44E-06	9.08E-05	Ipo8
MCAO 24h v.s. SHAM	mmu_circRNA_42073	chr7	+	110052710	110054116	exonic	-2.238	7.55347	-6.01	4.06E-05	0.000729	Ipo7
MCAO 24h v.s. SHAM	mmu_circRNA_39334	chr5	-	122947726	122961588	exonic	2.07	6.03478	6.821	1.13E-05	0.000315	Kdm2b
MCAO 24h v.s. SHAM	mmu_circRNA_39334	chr5	-	122947726	122961588	exonic	2.07	6.03478	6.821	1.13E-05	0.000315	Gm33118
MCAO 24h v.s. SHAM	mmu_circRNA_30491	chr17	+	35846301	35848665	exonic	-2.312	7.16074	-3.43	0.0043662	0.019092	Mdc1
MCAO 24h v.s. SHAM	mmu_circRNA_45523	chrX	-	74035347	74080032	sense overlapping	3.841	8.21785	7.945	2.17E-06	0.000119	Mecp2
MCAO 24h v.s. SHAM	mmu_circRNA_42792	chr8	-	36850163	36938758	exonic	2.955	7.30131	6.482	1.91E-05	0.000439	Dlc1
MCAO 24h v.s. SHAM	mmu_circRNA_34737	chr2	+	146870765	146891284	exonic	2.048	9.34661	7.004	8.52E-06	0.000267	Kiz
MCAO 24h v.s. SHAM	mmu_circRNA_43073	chr8	+	76906592	77085666	sense overlapping	3.827	6.54018	6.301	2.55E-05	0.000535	Nr3c2
MCAO 24h v.s. SHAM	mmu_circRNA_000692	chr9	-	86372340	86452010	exonic	2.768	6.20701	6.663	1.44E-05	0.000371	Ube2cbp
MCAO 24h v.s. SHAM	mmu_circRNA_21880	chr10	-	41922121	41951746	exonic	3.365	10.4083	8.597	8.98E-07	7.59E-05	Armc2
MCAO 24h v.s. SHAM	mmu_circRNA_40710	chr6	+	108369010	108440687	exonic	-2.067	6.60166	-4.37	0.00072929	0.005265	Itpr1
MCAO 24h v.s. SHAM	mmu_circRNA_45251	chr9	-	119489748	119492186	exonic	2.411	8.30375	7.53	3.91E-06	0.000164	Scn5a
MCAO 24h v.s. SHAM	mmu_circRNA_006917	chr8	+	82990346	83006412	exonic	2.349	6.76574	7.984	2.06E-06	0.000115	Rnf150
MCAO 24h v.s. SHAM	mmu_circRNA_41166	chr7	-	4430393	4435098	exonic	2.211	11.0901	6.185	3.07E-05	0.000606	Rdh13

MCAO 24h v.s. SHAM	mmu_circRNA_31317	chr18	+	12684836	12698059	exonic	3.086	10.9156	7.041	8.05E-06	0.00026	Ttc39c
MCAO 24h v.s. SHAM	mmu_circRNA_009247	chr19	-	37044521	37088587	exonic	2.082	11.656	5.961	4.43E-05	0.000774	Cpeb3
MCAO 24h v.s. SHAM	mmu_circRNA_38102	chr5	-	9569918	9590184	exonic	3.12	7.74002	8.439	1.11E-06	8.16E-05	Grm3
MCAO 24h v.s. SHAM	mmu_circRNA_008308	chr2	-	93012626	93013889	exonic	2.487	6.985	4.247	0.0009209	0.00623	Prdm11
MCAO 24h v.s. SHAM	mmu_circRNA_36481	chr4	+	11593620	11605005	exonic	5.2	12.1502	7.565	3.72E-06	0.000159	Rad54b
MCAO 24h v.s. SHAM	mmu_circRNA_24344	chr11	+	107485549	107489072	exonic	5.554	9.89977	9.022	5.18E-07	5.81E-05	Psmd12
MCAO 24h v.s. SHAM	mmu_circRNA_39856	chr6	+	17654105	17662169	exonic	-3.3	11.1066	-6.49	1.89E-05	0.000437	Capza2
MCAO 24h v.s. SHAM	mmu_circRNA_38760	chr5	-	72329611	72343117	exonic	-2.589	7.15788	-7.22	6.21E-06	0.000219	Corin
MCAO 24h v.s. SHAM	mmu_circRNA_38483	chr5	+	34834260	34854791	exonic	-2.769	9.96792	-5.26	0.00014707	0.001739	Htt
MCAO 24h v.s. SHAM	mmu_circRNA_30783	chr17	-	65858972	65867830	exonic	-2.816	6.9715	-7.62	3.45E-06	0.000152	Ralbp1
MCAO 24h v.s. SHAM	mmu_circRNA_30778	chr17	+	65837683	65838024	exonic	2.113	7.59016	7.091	7.48E-06	0.000248	Ppp4r1
MCAO 24h v.s. SHAM	mmu_circRNA_39443	chr5	-	128988801	128999587	exonic	-2.018	9.21534	-4.32	0.00080337	0.005681	Stx2
MCAO 24h v.s. SHAM	mmu_circRNA_41768	chr7	-	80923100	80927886	exonic	-2.174	8.255	-5.83	5.54E-05	0.000894	Sec11a
MCAO 24h v.s. SHAM	mmu_circRNA_017159	chr3	+	133320935	133330512	exonic	2.639	9.29524	8.76	7.25E-07	6.93E-05	Ppa2
MCAO 24h v.s. SHAM	mmu_circRNA_24439	chr11	-	116545833	116546968	exonic	-2.34	11.736	-7.33	5.20E-06	0.000195	Ube2o
MCAO 24h v.s. SHAM	mmu_circRNA_000704	chr7	+	96729348	96737561	exonic	2.714	5.77833	3.197	0.00689063	0.026485	Tenm4
MCAO 24h v.s. SHAM	mmu_circRNA_43649	chr8	-	128199297	128207761	sense overlapping	2.391	6.68362	6.992	8.67E-06	0.000269	NA
MCAO 24h v.s. SHAM	mmu_circRNA_21120	chr1	+	159861209	159868285	exonic	3.004	9.45099	6.437	2.05E-05	0.000462	Tnr
MCAO 24h v.s. SHAM	mmu_circRNA_26097	chr13	-	16002457	16003880	intronic	-4.265	8.40068	-6.02	4.03E-05	0.000725	NA
MCAO 24h v.s. SHAM	mmu_circRNA_23636	chr11	-	72070302	72075035	exonic	2.254	7.53127	8.517	9.98E-07	7.81E-05	Pitpnm3
MCAO 24h v.s. SHAM	mmu_circRNA_24642	chr12	-	5137422	5140708	exonic	-2.413	11.9372	-4.37	0.00073412	0.005296	Klhl29

MCAO 24h v.s. SHAM	mmu_circRNA_43201	chr8	-	84707659	84713857	sense overlapping	3.58	7.28274	10.8	6.28E-08	3.81E-05	Nfix
MCAO 24h v.s. SHAM	mmu_circRNA_24450	chr11	+	117735971	117740009	exonic	4.25	7.00855	9.707	2.22E-07	4.58E-05	Tnrc6c
MCAO 24h v.s. SHAM	mmu_circRNA_24450	chr11	+	117735971	117740009	exonic	4.25	7.00855	9.707	2.22E-07	4.58E-05	Gm11734
MCAO 24h v.s. SHAM	mmu_circRNA_44643	chr9	+	75193938	75215259	exonic	-2.561	8.55	-5.67	7.17E-05	0.001064	Myo5a
MCAO 24h v.s. SHAM	mmu_circRNA_24798	chr12	-	21353882	21361791	exonic	2.376	8.06477	7.292	5.54E-06	0.000202	Adam17
MCAO 24h v.s. SHAM	mmu_circRNA_29599	chr16	-	34116221	34220247	exonic	2.495	8.29917	7.875	2.40E-06	0.000126	Kalrn
MCAO 24h v.s. SHAM	mmu_circRNA_014144	chr8	+	108856721	108856953	exonic	-2.216	8.59907	-4.64	0.00044076	0.003664	Zfhx3
MCAO 24h v.s. SHAM	mmu_circRNA_26563	chr13	+	76641711	76759820	exonic	-2.004	8.7906	-5.93	4.68E-05	0.000802	Mctp1
MCAO 24h v.s. SHAM	mmu_circRNA_38590	chr5	-	51472597	51474411	exonic	3.241	11.8476	8.363	1.23E-06	8.55E-05	Ppargc1a
MCAO 24h v.s. SHAM	mmu_circRNA_33119	chr2	+	23075489	23087619	exonic	-2.461	7.14529	-5.49	9.81E-05	0.001335	Acbd5
MCAO 24h v.s. SHAM	mmu_circRNA_20913	chr1	-	135532348	135537299	exonic	-2.21	6.5197	-6.22	2.89E-05	0.000581	Nav1
MCAO 24h v.s. SHAM	mmu_circRNA_29760	chr16	+	44355439	44377022	exonic	4.412	11.9177	8.111	1.73E-06	0.000102	Spice1
MCAO 24h v.s. SHAM	mmu_circRNA_42216	chr7	+	118872820	118899886	exonic	2.14	6.06002	6.751	1.25E-05	0.000338	Iqck
MCAO 24h v.s. SHAM	mmu_circRNA_23911	chr11	-	84839980	84841624	exonic	-2.433	6.428	-5.89	4.97E-05	0.000829	Ggnbp2
MCAO 24h v.s. SHAM	mmu_circRNA_31938	chr18	-	71446336	71588027	exonic	2.219	11.3606	6.866	1.05E-05	0.0003	Dcc
MCAO 24h v.s. SHAM	mmu_circRNA_44832	chr9	+	95998215	96006847	exonic	-2.339	7.54576	-4.49	0.00058623	0.004481	Xrn1
MCAO 24h v.s. SHAM	mmu_circRNA_23290	chr11	-	50301757	50308929	exonic	-2.282	10.6565	-7.03	8.14E-06	0.00026	Canx
MCAO 24h v.s. SHAM	mmu_circRNA_32945	chr2	-	6721416	6747950	intronic	2.789	7.96055	4.881	0.00028659	0.002755	Celf2
MCAO 24h v.s. SHAM	mmu_circRNA_20389	chr1	-	79713857	79715000	exonic	-2.011	6.13515	-7.04	8.10E-06	0.00026	Wdfy1
MCAO 24h v.s. SHAM	mmu_circRNA_40611	chr6	-	99162838	99669567	sense overlapping	3.391	11.8573	7.926	2.23E-06	0.000121	NA

MCAO 24h v.s. SHAM	mmu_circRNA_32069	chr18	-	82618742	82624486	exonic	-2.51	6.93891	-6.39	2.21E-05	0.000481	Zfp236
MCAO 24h v.s. SHAM	mmu_circRNA_25329	chr12	+	73926556	73937800	exonic	-3	10.6729	-3.33	0.00532311	0.022033	Hif1a
MCAO 24h v.s. SHAM	mmu_circRNA_25329	chr12	+	73926556	73937800	exonic	-3	10.6729	-3.33	0.00532311	0.022033	Gm15283
MCAO 24h v.s. SHAM	mmu_circRNA_25607	chr12	-	91213564	91299077	exonic	-2.95	9.15897	-7.37	4.94E-06	0.000188	Cep128
MCAO 24h v.s. SHAM	mmu_circRNA_43263	chr8	-	86724205	86725856	sense overlapping	2.253	13.3906	5.371	0.00012045	0.001537	Siah1a
MCAO 24h v.s. SHAM	mmu_circRNA_43998	chr9	-	37311317	37316527	exonic	-2.068	6.53499	-4.2	0.00099991	0.006588	Ccdc15
MCAO 24h v.s. SHAM	mmu_circRNA_32884	chr2	-	5354668	5362127	exonic	-3.75	11.6701	-6.97	8.99E-06	0.000273	Camk1d
MCAO 24h v.s. SHAM	mmu_circRNA_28610	chr15	+	55208730	55218273	exonic	2.373	6.0862	6.087	3.60E-05	0.00067	Deptor
MCAO 24h v.s. SHAM	mmu_circRNA_21349	chr1	-	177050037	177109738	exonic	-2.026	8.20921	-6.06	3.77E-05	0.000693	Akt3
MCAO 24h v.s. SHAM	mmu_circRNA_37971	chr4	+	150508829	150570296	exonic	2.595	7.5097	4.848	0.0003042	0.00286	Rere
MCAO 24h v.s. SHAM	mmu_circRNA_35751	chr3	-	88910313	88910810	exonic	-3.144	9.14707	-5.83	5.54E-05	0.000894	Msto1
MCAO 24h v.s. SHAM	mmu_circRNA_19193	chr17	+	57419319	57420525	sense overlapping	2.525	6.1426	6.354	2.34E-05	0.0005	Adgre1
MCAO 24h v.s. SHAM	mmu_circRNA_39827	chr6	+	8280220	8291914	intronic	3.334	6.41158	6.918	9.71E-06	0.000285	Umad1
MCAO 24h v.s. SHAM	mmu_circRNA_30884	chr17	-	73906009	73907745	exonic	5.382	9.06227	8.609	8.84E-07	7.59E-05	Xdh
MCAO 24h v.s. SHAM	mmu_circRNA_41079	chr6	+	146866736	146868264	exonic	-2.515	6.50603	-4.91	0.00027068	0.002649	Smco2
MCAO 24h v.s. SHAM	mmu_circRNA_015601	chr5	-	99240822	99243246	exonic	2.051	9.1555	5.212	0.00015907	0.001829	A930011G23Rik
MCAO 24h v.s. SHAM	mmu_circRNA_015601	chr5	-	99240822	99243246	exonic	2.051	9.1555	5.212	0.00015907	0.001829	Rasgef1b
MCAO 24h v.s. SHAM	mmu_circRNA_35962	chr3	-	107445429	107448440	exonic	3.738	12.5675	8.372	1.21E-06	8.51E-05	Kcnc4
MCAO 24h v.s. SHAM	mmu_circRNA_26316	chr13	+	49820612	49830616	intergenic	-2.672	8.79094	-4.87	0.00029359	0.002804	LOC102631805
MCAO 24h v.s. SHAM	mmu_circRNA_013699	chr10	-	9841870	9843361	exonic	-2.35	10.4659	-7.25	5.86E-06	0.000211	Stxbp5
MCAO 24h v.s. SHAM	mmu_circRNA_23961	chr11	-	86108766	86157878	exonic	2.168	8.36419	7.284	5.61E-06	0.000203	Brip1

MCAO 24h v.s. SHAM	mmu_circRNA_25530	chr12	+	85826452	85933674	exonic	2.08	9.72457	5.896	4.94E-05	0.000828	Ttll5
MCAO 24h v.s. SHAM	mmu_circRNA_43896	chr9	-	25124871	25142311	sense overlapping	-2.449	6.55179	-6.01	4.12E-05	0.000735	Herpud2
MCAO 24h v.s. SHAM	mmu_circRNA_23881	chr11	+	83321938	83336861	exonic	3.509	11.8823	7.697	3.08E-06	0.000143	Ap2b1
MCAO 24h v.s. SHAM	mmu_circRNA_43831	chr9	+	21655626	21660871	exonic	2.062	9.29955	5.063	0.00020694	0.002196	Smarca4
MCAO 24h v.s. SHAM	mmu_circRNA_010147	chr19	-	37068172	37088587	exonic	3.579	11.8228	7.791	2.70E-06	0.000137	Cpeb3
MCAO 24h v.s. SHAM	mmu_circRNA_31790	chr18	+	60546220	60556370	sense overlapping	-2.017	6.19422	-6.04	3.92E-05	0.000709	Dctn4
MCAO 24h v.s. SHAM	mmu_circRNA_44088	chr9	-	45943050	45944486	exonic	4.716	11.2441	10.03	1.51E-07	4.38E-05	Sidt2
MCAO 24h v.s. SHAM	mmu_circRNA_22088	chr10	-	62274446	62286652	exonic	3.973	9.9047	7.684	3.14E-06	0.000144	Hk1
MCAO 24h v.s. SHAM	mmu_circRNA_42738	chr8	+	31114602	31118587	sense overlapping	3.518	8.6777	7.432	4.51E-06	0.000177	Rnf122
MCAO 24h v.s. SHAM	mmu_circRNA_42273	chr7	+	122754505	122762323	exonic	5.272	9.65921	10.53	8.48E-08	3.91E-05	Cacng3
MCAO 24h v.s. SHAM	mmu_circRNA_27031	chr14	-	10729724	10751694	exonic	-3.145	6.63967	-7.6	3.53E-06	0.000154	Fhit
MCAO 24h v.s. SHAM	mmu_circRNA_27031	chr14	-	10729724	10751694	exonic	-3.145	6.63967	-7.6	3.53E-06	0.000154	Fhitos
MCAO 24h v.s. SHAM	mmu_circRNA_37727	chr4	+	130727997	130774604	exonic	-3.306	12.7307	-8.61	8.82E-07	7.59E-05	Pum1
MCAO 24h v.s. SHAM	mmu_circRNA_42362	chr7	+	129629917	129632421	exonic	4.181	10.6303	6.51	1.83E-05	0.000429	Wdr11
MCAO 24h v.s. SHAM	mmu_circRNA_41406	chr7	+	49780308	49829030	exonic	2.889	9.0492	6.153	3.23E-05	0.000621	Prmt3
MCAO 24h v.s. SHAM	mmu_circRNA_29484	chr16	-	30545125	30547469	exonic	2.329	10.6491	4.453	0.00062729	0.004703	Tmem44
MCAO 24h v.s. SHAM	mmu_circRNA_30990	chr17	-	80251231	80267639	exonic	2.067	9.2142	6.559	1.69E-05	0.000409	Dhx57
MCAO 24h v.s. SHAM	mmu_circRNA_017949	chr6	-	37029762	37067385	exonic	2.027	6.6825	3.012	0.00986069	0.034419	Dgki
MCAO 24h v.s. SHAM	mmu_circRNA_38782	chr5	+	73510885	73544155	exonic	3.756	7.02379	5.405	0.00011365	0.001483	Dcun1d4
MCAO 24h v.s. SHAM	mmu_circRNA_41054	chr6	-	145032782	145039635	exonic	-3.744	13.9357	-4.59	0.00048663	0.003928	Bcat1

MCAO 24h v.s. SHAM	mmu_circRNA_24514	chr11	-	121623431	121630867	exonic	-2.061	14.2614	-7.63	3.39E-06	0.000152	B3gntl1
MCAO 24h v.s. SHAM	mmu_circRNA_40842	chr6	-	119361827	119365380	exonic	-2.604	10.7779	-6.17	3.15E-05	0.000613	Adipor2
MCAO 24h v.s. SHAM	mmu_circRNA_31621	chr18	+	37020094	37159353	intronic	2.43	6.29889	5.442	0.00010664	0.001419	Gm38666
MCAO 24h v.s. SHAM	mmu_circRNA_31621	chr18	+	37020094	37159353	intronic	2.43	6.29889	5.442	0.00010664	0.001419	Pcdha1
MCAO 24h v.s. SHAM	mmu_circRNA_31621	chr18	+	37020094	37159353	intronic	2.43	6.29889	5.442	0.00010664	0.001419	Pcdha5
MCAO 24h v.s. SHAM	mmu_circRNA_31621	chr18	+	37020094	37159353	intronic	2.43	6.29889	5.442	0.00010664	0.001419	Pcdha7
MCAO 24h v.s. SHAM	mmu_circRNA_31621	chr18	+	37020094	37159353	intronic	2.43	6.29889	5.442	0.00010664	0.001419	Gm38667
MCAO 24h v.s. SHAM	mmu_circRNA_31621	chr18	+	37020094	37159353	intronic	2.43	6.29889	5.442	0.00010664	0.001419	Pcdha4
MCAO 24h v.s. SHAM	mmu_circRNA_31621	chr18	+	37020094	37159353	intronic	2.43	6.29889	5.442	0.00010664	0.001419	Pcdha9
MCAO 24h v.s. SHAM	mmu_circRNA_31621	chr18	+	37020094	37159353	intronic	2.43	6.29889	5.442	0.00010664	0.001419	Gm37013
MCAO 24h v.s. SHAM	mmu_circRNA_31621	chr18	+	37020094	37159353	intronic	2.43	6.29889	5.442	0.00010664	0.001419	Pcdha10
MCAO 24h v.s. SHAM	mmu_circRNA_31621	chr18	+	37020094	37159353	intronic	2.43	6.29889	5.442	0.00010664	0.001419	Pcdha6
MCAO 24h v.s. SHAM	mmu_circRNA_31621	chr18	+	37020094	37159353	intronic	2.43	6.29889	5.442	0.00010664	0.001419	Pcdha3
MCAO 24h v.s. SHAM	mmu_circRNA_31621	chr18	+	37020094	37159353	intronic	2.43	6.29889	5.442	0.00010664	0.001419	Pcdha8
MCAO 24h v.s. SHAM	mmu_circRNA_31621	chr18	+	37020094	37159353	intronic	2.43	6.29889	5.442	0.00010664	0.001419	Pcdha11
MCAO 24h v.s. SHAM	mmu_circRNA_31621	chr18	+	37020094	37159353	intronic	2.43	6.29889	5.442	0.00010664	0.001419	Pcdha2
MCAO 24h v.s. SHAM	mmu_circRNA_45613	chrX	-	94535859	94535994	sense overlapping	3.41	6.13883	5.996	4.18E-05	0.000742	Maged1
MCAO 24h v.s. SHAM	mmu_circRNA_34837	chr2	-	156096417	156103397	exonic	2.407	6.71151	9.506	2.83E-07	5.03E-05	Cpne1
MCAO 24h v.s. SHAM	mmu_circRNA_34837	chr2	-	156096417	156103397	exonic	2.407	6.71151	9.506	2.83E-07	5.03E-05	Rbm12
MCAO 24h v.s. SHAM	mmu_circRNA_29464	chr16	+	24559591	24608317	exonic	-2.065	6.90893	-5.21	0.00015994	0.001831	Lpp
MCAO 24h v.s. SHAM	mmu_circRNA_36877	chr4	-	59514273	59517755	exonic	-2.093	13.7324	-4.63	0.00044943	0.003712	Ptbp3

MCAO 24h v.s. SHAM	mmu_circRNA_43751	chr9	-	9583050	9634385	intergenic	-2.632	10.5206	-4.83	0.00031327	0.002919	LOC108167638
MCAO 24h v.s. SHAM	mmu_circRNA_34013	chr2	+	90751067	90753190	exonic	-2.033	7.04717	-5.58	8.39E-05	0.001192	Fnbp4
MCAO 24h v.s. SHAM	mmu_circRNA_38722	chr5	-	67398199	67405519	sense overlapping	-3.131	11.0972	-5.18	0.00016941	0.001895	Gm33345
MCAO 24h v.s. SHAM	mmu_circRNA_38722	chr5	-	67398199	67405519	sense overlapping	-3.131	11.0972	-5.18	0.00016941	0.001895	Bend4
MCAO 24h v.s. SHAM	mmu_circRNA_28019	chr14	+	116116298	116183082	intronic	3.238	5.33604	4.268	0.00088425	0.006074	Gpc5
MCAO 24h v.s. SHAM	mmu_circRNA_002520	chr7	-	59507338	59568724	sense overlapping	4.989	8.05372	3.114	0.00809263	0.029775	Snhg14
MCAO 24h v.s. SHAM	mmu_circRNA_40590	chr6	-	94654846	94663998	exonic	3.862	12.122	8.218	1.49E-06	9.34E-05	Lrig1
MCAO 24h v.s. SHAM	mmu_circRNA_29063	chr15	-	99278688	99285665	exonic	-2.006	9.37541	-4.35	0.00076583	0.005479	Fam186b
MCAO 24h v.s. SHAM	mmu_circRNA_33110	chr2	-	20887105	20914811	exonic	-2.38	10.0237	-5.93	4.70E-05	0.000802	Arhgap21
MCAO 24h v.s. SHAM	mmu_circRNA_013226	chr2	-	144255339	144257195	exonic	-2.326	9.21968	-7.06	7.88E-06	0.000258	Snx5
MCAO 24h v.s. SHAM	mmu_circRNA_42581	chr8	+	14305203	14314292	intronic	2.626	7.54848	6.46	1.98E-05	0.000452	Dlgap2
MCAO 24h v.s. SHAM	mmu_circRNA_22847	chr11	-	4782186	4820493	exonic	-2.727	6.87177	-5.34	0.00012782	0.001598	Nf2
MCAO 24h v.s. SHAM	mmu_circRNA_24939	chr12	-	36118986	36134955	exonic	-2.12	12.5723	-5.87	5.14E-05	0.000852	Bzw2
MCAO 24h v.s. SHAM	mmu_circRNA_44014	chr9	-	42000243	42014574	exonic	3.219	8.5422	8.741	7.44E-07	6.98E-05	Sorl1
MCAO 24h v.s. SHAM	mmu_circRNA_004260	chr6	+	17834616	17855028	exonic	-2.107	9.83718	-7	8.52E-06	0.000267	St7
MCAO 24h v.s. SHAM	mmu_circRNA_21090	chr1	-	156869440	156901890	exonic	-2.234	10.1622	-5.68	7.04E-05	0.001048	Ralgps2
MCAO 24h v.s. SHAM	mmu_circRNA_32405	chr19	-	28938231	28944454	exonic	2.663	8.13973	5.281	0.00014088	0.001697	4430402I18Rik
MCAO 24h v.s. SHAM	mmu_circRNA_41535	chr7	-	61813261	61913335	exonic	2.367	6.64059	7.092	7.46E-06	0.000248	A230057D06Rik
MCAO 24h v.s. SHAM	mmu_circRNA_42207	chr7	+	118638128	118640007	exonic	2.705	7.98536	6.95	9.25E-06	0.000277	Tmc5
MCAO 24h v.s. SHAM	mmu_circRNA_27287	chr14	+	27449945	27459851	exonic	4.866	12.2679	8.495	1.03E-06	7.91E-05	Fam208a

MCAO 24h v.s. SHAM	mmu_circRNA_21469	chr1	-	181184250	181186067	exonic	-2.095	10.7396	-4.19	0.00102907	0.006707	Wdr26
MCAO 24h v.s. SHAM	mmu_circRNA_27821	chr14	+	77268806	77441899	exonic	4.988	10.955	8.959	5.61E-07	6.03E-05	Enox1
MCAO 24h v.s. SHAM	mmu_circRNA_37824	chr4	+	138057948	138105436	exonic	2.143	12.6063	6.999	8.59E-06	0.000268	Eif4g3
MCAO 24h v.s. SHAM	mmu_circRNA_26177	chr13	-	30856653	30911250	exonic	-2.061	8.06958	-5.14	0.00018002	0.001975	Exoc2
MCAO 24h v.s. SHAM	mmu_circRNA_33806	chr2	-	70721557	70749327	exonic	2.144	8.43554	3.887	0.001819	0.010064	Tlk1
MCAO 24h v.s. SHAM	mmu_circRNA_31671	chr18	+	42288107	42305814	exonic	2.968	10.5446	6.926	9.59E-06	0.000284	Rbm27
MCAO 24h v.s. SHAM	mmu_circRNA_24944	chr12	+	36501977	36522051	exonic	2.838	10.1168	8.027	1.94E-06	0.00011	Ispd
MCAO 24h v.s. SHAM	mmu_circRNA_33003	chr2	+	14981305	14984439	exonic	-2.305	11.6408	-5.04	0.00021657	0.00227	Cacnb2
MCAO 24h v.s. SHAM	mmu_circRNA_30586	chr17	+	50711410	50718783	intergenic	4.423	10.1033	6.275	2.66E-05	0.000549	NA
MCAO 24h v.s. SHAM	mmu_circRNA_27546	chr14	-	51905612	51905724	sense overlapping	2.275	8.02883	3.497	0.0038475	0.017338	Ndrg2
MCAO 24h v.s. SHAM	mmu_circRNA_31169	chr17	-	94694281	94695119	intergenic	5.854	7.9631	9.671	2.31E-07	4.60E-05	NA
MCAO 24h v.s. SHAM	mmu_circRNA_24167	chr11	+	98257346	98267858	sense overlapping	-3.232	10.0477	-7.51	4.05E-06	0.000168	NA
MCAO 24h v.s. SHAM	mmu_circRNA_38545	chr5	+	43709043	43714601	exonic	3.74	10.5431	9.204	4.11E-07	5.26E-05	Cc2d2a
MCAO 24h v.s. SHAM	mmu_circRNA_37781	chr4	+	134934931	134936081	exonic	2.407	7.54487	6.076	3.67E-05	0.00068	Syf2
MCAO 24h v.s. SHAM	mmu_circRNA_38573	chr5	-	45735730	45795358	exonic	3.997	9.57818	6.909	9.83E-06	0.000287	Lcorl
MCAO 24h v.s. SHAM	mmu_circRNA_38350	chr5	-	29323801	29346906	exonic	2	10.9308	6.296	2.57E-05	0.000538	Lmbr1
MCAO 24h v.s. SHAM	mmu_circRNA_22663	chr10	-	116343842	116346937	antisense	4.969	9.56817	9.576	2.60E-07	4.97E-05	Ptprb
MCAO 24h v.s. SHAM	mmu_circRNA_45608	chrX	-	93800113	93814068	exonic	-2.377	9.58389	-5.16	0.00017389	0.001928	Pdk3
MCAO 24h v.s. SHAM	mmu_circRNA_38258	chr5	-	23979487	24000019	exonic	-2.139	10.2283	-4.18	0.00103464	0.006731	Fam126a
MCAO 24h v.s. SHAM	mmu_circRNA_40915	chr6	-	124436817	124438985	exonic	2.146	8.94444	5.491	9.80E-05	0.001335	Clstn3
MCAO 24h v.s. SHAM	mmu_circRNA_27264	chr14	+	26900422	26934532	sense overlapping	4.53	7.76428	8.622	8.69E-07	7.59E-05	Appl1

MCAO 24h v.s. SHAM	mmu_circRNA_003795	chr1	-	155847109	155848727	sense overlapping	-4.507	11.5818	-7.92	2.25E-06	0.000121	Cep350
MCAO 24h v.s. SHAM	mmu_circRNA_36100	chr3	+	122794259	122843108	exonic	-2.173	8.82627	-6.05	3.84E-05	0.000701	4930447N08Rik
MCAO 24h v.s. SHAM	mmu_circRNA_36100	chr3	+	122794259	122843108	exonic	-2.173	8.82627	-6.05	3.84E-05	0.000701	Pde5a
MCAO 24h v.s. SHAM	mmu_circRNA_008640	chr6	+	55460156	55461614	exonic	2.11	8.75301	2.859	0.01324814	0.043048	Adcyap1r1
MCAO 24h v.s. SHAM	mmu_circRNA_004960	chr6	-	115969875	115970248	exonic	2.851	8.8388	8.209	1.51E-06	9.37E-05	Plxnd1
MCAO 24h v.s. SHAM	mmu_circRNA_19110	chr14	-	19904158	19904277	intronic	4.006	11.1538	8.272	1.39E-06	8.93E-05	Gng2
MCAO 24h v.s. SHAM	mmu_circRNA_009044	chr7	-	67529585	67559969	exonic	2.062	8.2101	5.632	7.70E-05	0.001125	Gm34079
MCAO 24h v.s. SHAM	mmu_circRNA_009044	chr7	-	67529585	67559969	exonic	2.062	8.2101	5.632	7.70E-05	0.001125	Lrrc28
MCAO 24h v.s. SHAM	mmu_circRNA_45033	chr9	-	106239531	106241785	exonic	-2.698	12.3737	-6.43	2.08E-05	0.000465	Alas1
MCAO 24h v.s. SHAM	mmu_circRNA_34423	chr2	-	121394346	121413480	sense overlapping	2.288	8.23884	5.672	7.19E-05	0.001064	Catsper2
MCAO 24h v.s. SHAM	mmu_circRNA_28026	chr14	+	118153568	118160184	exonic	4.135	8.81652	9.123	4.55E-07	5.38E-05	Gpr180
MCAO 24h v.s. SHAM	mmu_circRNA_25460	chr12	+	82677352	82681408	intronic	-2.414	7.13749	-6.1	3.53E-05	0.00066	Rgs6
MCAO 24h v.s. SHAM	mmu_circRNA_22790	chr10	+	128207297	128208667	exonic	3.222	7.95375	5.101	0.0001936	0.002092	Gls2
MCAO 24h v.s. SHAM	mmu_circRNA_41481	chr7	-	59284025	59317973	sense overlapping	3.228	9.50828	8.666	8.20E-07	7.43E-05	Snhg14
MCAO 24h v.s. SHAM	mmu_circRNA_43465	chr8	+	110645757	110671182	exonic	2.188	8.49979	7.455	4.37E-06	0.000173	Vac14
MCAO 24h v.s. SHAM	mmu_circRNA_26040	chr13	+	13725813	13761201	exonic	-2.312	7.61351	-4.29	0.00084276	0.005887	Lyst
MCAO 24h v.s. SHAM	mmu_circRNA_41647	chr7	+	70474179	70494757	sense overlapping	2.138	6.27192	8.498	1.02E-06	7.91E-05	NA
MCAO 24h v.s. SHAM	mmu_circRNA_33249	chr2	-	30110204	30111332	exonic	-2.002	8.18039	-6.73	1.29E-05	0.000344	Zer1
MCAO 24h v.s. SHAM	mmu_circRNA_002246	chr7	-	142651460	142651527	sense overlapping	-4.67	7.52559	-7.01	8.38E-06	0.000265	Igf2
MCAO 24h v.s. SHAM	mmu_circRNA_19314	chr4	-	3080977	3084320	intergenic	4.22	10.6166	8.915	5.94E-07	6.22E-05	NA

MCAO 24h v.s. SHAM	mmu_circRNA_006741	chr6	-	119052600	119057515	exonic	3.192	8.357	9.08	4.81E-07	5.51E-05	Cacna1c
MCAO 24h v.s. SHAM	mmu_circRNA_22526	chr10	+	99402065	99402880	sense overlapping	5.507	8.75673	11.1	4.55E-08	3.37E-05	NA
MCAO 24h v.s. SHAM	mmu_circRNA_009489	chr6	+	29372580	29372670	intronic	2.199	8.22179	7.437	4.48E-06	0.000177	Calu
MCAO 24h v.s. SHAM	mmu_circRNA_38919	chr5	+	88934748	88954957	exonic	3.286	8.87298	7.778	2.75E-06	0.000137	Slc4a4
MCAO 24h v.s. SHAM	mmu_circRNA_30520	chr17	-	45642467	45642962	exonic	3.29	7.24195	3.325	0.00537598	0.022158	Capn11
MCAO 24h v.s. SHAM	mmu_circRNA_24456	chr11	-	118182219	118182913	exonic	2.105	8.78585	6.854	1.07E-05	0.000304	Cyth1
MCAO 24h v.s. SHAM	mmu_circRNA_41238	chr7	-	27541084	27542181	exonic	2.957	10.0306	7.616	3.46E-06	0.000152	Pld3
MCAO 24h v.s. SHAM	mmu_circRNA_30332	chr17	+	25145568	25155537	exonic	3.131	9.76241	9.025	5.16E-07	5.81E-05	Clcn7
MCAO 24h v.s. SHAM	mmu_circRNA_34860	chr2	+	156664798	156711261	exonic	3.16	9.01726	8.773	7.14E-07	6.91E-05	Dlgap4
MCAO 24h v.s. SHAM	mmu_circRNA_23585	chr11	+	69026069	69026579	sense overlapping	2.084	8.40521	6.393	2.20E-05	0.000479	Ctc1
MCAO 24h v.s. SHAM	mmu_circRNA_19015	chr11	+	53893644	53981397	sense overlapping	3.761	10.4419	6.055	3.80E-05	0.000695	Gm30927
MCAO 24h v.s. SHAM	mmu_circRNA_42567	chr8	+	12806649	12813155	exonic	2.595	11.152	6.065	3.74E-05	0.00069	Atp11a
MCAO 24h v.s. SHAM	mmu_circRNA_19198	chr17	+	68455690	68463021	sense overlapping	2.354	5.61195	7.241	5.97E-06	0.000213	L3mbtl4
MCAO 24h v.s. SHAM	mmu_circRNA_38255	chr5	+	23807120	23819951	sense overlapping	-2.633	9.32812	-3.36	0.0050436	0.021115	Rint1
MCAO 24h v.s. SHAM	mmu_circRNA_45137	chr9	-	110432433	110439058	exonic	2.324	10.1696	5.209	0.00015997	0.001831	Klhl18
MCAO 24h v.s. SHAM	mmu_circRNA_28632	chr15	+	62054793	62107465	intronic	2.308	10.8688	5.025	0.00022147	0.002303	Pvt1
MCAO 24h v.s. SHAM	mmu_circRNA_30869	chr17	+	71695696	71697996	exonic	2.323	6.15365	6.23	2.85E-05	0.000577	Togaram2
MCAO 24h v.s. SHAM	mmu_circRNA_36895	chr4	+	65176208	65237931	exonic	2.259	9.9772	5.941	4.58E-05	0.000792	Pappa
MCAO 24h v.s. SHAM	mmu_circRNA_23261	chr11	-	46221335	46224205	exonic	3.172	10.9546	7.476	4.23E-06	0.000171	Cyfip2
MCAO 24h v.s. SHAM	mmu_circRNA_41378	chr7	-	46743993	46750514	intergenic	3.717	9.74998	9.23	3.98E-07	5.20E-05	NA
MCAO 24h v.s. SHAM	mmu_circRNA_20836	chr1	+	128252202	128269971	exonic	2.386	7.37815	3.565	0.00337643	0.015729	Ubxn4

MCAO 24h v.s. SHAM	mmu_circRNA_39371	chr5	+	123934071	123936555	exonic	3.283	9.61083	5.369	0.00012091	0.001539	Ccdc62
MCAO 24h v.s. SHAM	mmu_circRNA_37770	chr4	-	134148009	134172880	exonic	2.674	9.51011	4.615	0.00046458	0.003801	Cep85
MCAO 24h v.s. SHAM	mmu_circRNA_20054	chr1	-	52189422	52207359	exonic	3.31	7.59221	7.499	4.09E-06	0.000169	Gls
MCAO 24h v.s. SHAM	mmu_circRNA_001874	chr9	+	120453526	120464961	exonic	2.561	9.54261	5.188	0.00016594	0.001874	Myrip
MCAO 24h v.s. SHAM	mmu_circRNA_013002	chr16	+	94403313	94468060	sense overlapping	3.127	10.6294	6.757	1.24E-05	0.000336	Ttc3
MCAO 24h v.s. SHAM	mmu_circRNA_011696	chr17	+	39844601	39844697	sense overlapping	2.404	13.7386	4.38	0.00071769	0.005203	NA
MCAO 24h v.s. SHAM	mmu_circRNA_24446	chr11	+	117700724	117723550	exonic	-3.673	11.8964	-10.5	8.52E-08	3.91E-05	Tnrc6c
MCAO 24h v.s. SHAM	mmu_circRNA_24446	chr11	+	117700724	117723550	exonic	-3.673	11.8964	-10.5	8.52E-08	3.91E-05	Gm11734
MCAO 24h v.s. SHAM	mmu_circRNA_40913	chr6	+	122828156	122833876	exonic	3.079	12.9587	9.513	2.81E-07	5.03E-05	Foxj2
MCAO 24h v.s. SHAM	mmu_circRNA_39558	chr5	-	138150606	138152969	exonic	-2.202	7.52635	-7.3	5.44E-06	0.000199	Zfp113
MCAO 24h v.s. SHAM	mmu_circRNA_011703	chr15	+	39555064	39616510	sense overlapping	2.356	6.25351	2.962	0.01085035	0.036946	Rims2
MCAO 24h v.s. SHAM	mmu_circRNA_34392	chr2	-	120929385	120955720	exonic	2.323	8.40392	3.268	0.00599631	0.023902	Ubr1
MCAO 24h v.s. SHAM	mmu_circRNA_003170	chr2	+	18101457	18126229	exonic	3.256	11.6332	6.698	1.36E-05	0.000355	Mllt10
MCAO 24h v.s. SHAM	mmu_circRNA_002314	chr13	+	8672404	8731971	exonic	3.052	8.09707	5.014	0.00022601	0.002328	Adarb2
MCAO 24h v.s. SHAM	mmu_circRNA_29156	chr16	+	5234965	5239166	exonic	3.023	11.4155	6.964	9.04E-06	0.000274	Alg1
MCAO 24h v.s. SHAM	mmu_circRNA_015541	chr2	+	37508927	37541176	exonic	3.638	9.68183	5.255	0.00014746	0.001739	Rabgap1
MCAO 24h v.s. SHAM	mmu_circRNA_015541	chr2	+	37508927	37541176	exonic	3.638	9.68183	5.255	0.00014746	0.001739	Strbp
MCAO 24h v.s. SHAM	mmu_circRNA_42495	chr7	-	143584647	143588583	exonic	2.948	6.76258	4.273	0.00087582	0.006041	Cars
MCAO 24h v.s. SHAM	mmu_circRNA_38761	chr5	-	72353869	72360758	exonic	3.005	9.91956	5.671	7.20E-05	0.001064	Corin
MCAO 24h v.s. SHAM	mmu_circRNA_013794	chr4	+	152112107	152112700	exonic	3.523	8.21143	8.282	1.37E-06	8.93E-05	Plekhg5

MCAO 24h v.s. SHAM	mmu_circRNA_19080	chr12	+	104237731	104247974	intronic	2.166	7.82583	5.029	0.00022002	0.002294	Serpina3g
MCAO 24h v.s. SHAM	mmu_circRNA_34309	chr2	+	119701117	119705643	exonic	-2.176	6.00367	-6.22	2.89E-05	0.000581	Rtf1
MCAO 24h v.s. SHAM	mmu_circRNA_27565	chr14	-	55640384	55641588	exonic	4.306	10.9835	6.539	1.74E-05	0.000419	Tm9sf1
MCAO 24h v.s. SHAM	mmu_circRNA_29807	chr16	+	52047198	52047449	exonic	-2.201	7.87873	-6.83	1.12E-05	0.000314	Cblb
MCAO 24h v.s. SHAM	mmu_circRNA_27592	chr14	+	56887794	56919342	exonic	3.265	7.43915	5.488	9.84E-05	0.001337	Zmym2
MCAO 24h v.s. SHAM	mmu_circRNA_23299	chr11	-	50910435	50912404	exonic	2.464	7.77346	4.356	0.00075085	0.005391	Zfp2
MCAO 24h v.s. SHAM	mmu_circRNA_39090	chr5	-	106600275	106666845	sense overlapping	-2.077	7.9969	-4.6	0.00047533	0.003867	Zfp644
MCAO 24h v.s. SHAM	mmu_circRNA_43037	chr8	+	72725536	72731278	exonic	3.613	6.7959	7.514	4.00E-06	0.000167	Sin3b
MCAO 24h v.s. SHAM	mmu_circRNA_24114	chr11	-	95289771	95294645	exonic	-2.07	8.19932	-5.26	0.00014731	0.001739	Kat7
MCAO 24h v.s. SHAM	mmu_circRNA_19044	chr11	-	104368678	104378827	sense overlapping	-2.494	11.2665	-8.07	1.82E-06	0.000106	Kansl1
MCAO 24h v.s. SHAM	mmu_circRNA_31098	chr17	+	86559150	86559724	sense overlapping	3.574	8.31396	5.97	4.37E-05	0.000766	Prkce
MCAO 24h v.s. SHAM	mmu_circRNA_36263	chr3	-	144705512	144709361	exonic	-3.434	10.2896	-8.27	1.38E-06	8.93E-05	NA
MCAO 24h v.s. SHAM	mmu_circRNA_40285	chr6	-	52777534	52894071	exonic	-2.092	8.5611	-3.39	0.00475541	0.020287	Jazf1
MCAO 24h v.s. SHAM	mmu_circRNA_19186	chr17	-	33977770	33997565	sense overlapping	2.122	8.2004	6.255	2.74E-05	0.000559	H2-K1
MCAO 24h v.s. SHAM	mmu_circRNA_30283	chr17	+	20355630	20360447	exonic	2.357	6.15027	9.001	5.32E-07	5.84E-05	Vmn2r107
MCAO 24h v.s. SHAM	mmu_circRNA_34106	chr2	-	101758509	101772408	exonic	-2.113	11.5585	-4.75	0.00036081	0.003188	Prr5l
MCAO 24h v.s. SHAM	mmu_circRNA_23070	chr11	+	26407547	26434500	exonic	-2.172	9.35834	-4.83	0.00031423	0.002923	Fancl
MCAO 24h v.s. SHAM	mmu_circRNA_25585	chr12	+	88850278	88853112	intronic	2.654	12.3907	5.814	5.66E-05	0.000906	Nrxn3
MCAO 24h v.s. SHAM	mmu_circRNA_39113	chr5	-	107137529	107149958	exonic	2.446	10.2656	6.954	9.19E-06	0.000277	Tgfbr3
MCAO 24h v.s. SHAM	mmu_circRNA_36559	chr4	+	24505344	24578903	exonic	-2.293	9.21446	-3.11	0.00815552	0.029932	Mms22l
MCAO 24h v.s. SHAM	mmu_circRNA_41329	chr7	-	37961387	37969697	sense overlapping	3.496	7.86328	5.014	0.00022603	0.002328	Uri1

MCAO 24h v.s. SHAM	mmu_circRNA_002563	chr18	+	22375132	22434675	exonic	2.451	11.7048	5.078	0.00020164	0.002153	Asxl3
MCAO 24h v.s. SHAM	mmu_circRNA_22776	chr10	-	127299948	127305857	exonic	2.494	7.28734	7.329	5.24E-06	0.000195	Mars
MCAO 24h v.s. SHAM	mmu_circRNA_29625	chr16	-	35422865	35449463	exonic	-2.133	14.0795	-6.28	2.63E-05	0.000546	Pdia5
MCAO 24h v.s. SHAM	mmu_circRNA_32658	chr19	+	44952068	44979229	exonic	-2.15	9.08455	-6.01	4.10E-05	0.000734	NA
MCAO 24h v.s. SHAM	mmu_circRNA_25573	chr12	+	86952743	86961903	sense overlapping	-2	10.3291	-4.87	0.00028978	0.002773	Cipc
MCAO 24h v.s. SHAM	mmu_circRNA_30337	chr17	-	25380224	25381573	exonic	2.193	9.03534	5.458	0.00010374	0.001393	Cacna1h
MCAO 24h v.s. SHAM	mmu_circRNA_38721	chr5	-	67398199	67400301	sense overlapping	-2.022	5.91948	-4.22	0.00096091	0.006399	Bend4
MCAO 24h v.s. SHAM	mmu_circRNA_36945	chr4	+	74187313	74187769	exonic	2.161	13.6928	8.271	1.39E-06	8.93E-05	Frmd3
MCAO 24h v.s. SHAM	mmu_circRNA_24544	chr12	+	3591905	3632964	exonic	-2.72	11.4848	-6.57	1.65E-05	0.000403	Dtnb
MCAO 24h v.s. SHAM	mmu_circRNA_25566	chr12	+	86253847	86256932	sense overlapping	-2.505	6.26677	-8.07	1.84E-06	0.000106	Gpatch2l
MCAO 24h v.s. SHAM	mmu_circRNA_27181	chr14	-	21476670	21483639	antisense	2.2	12.1141	4.655	0.00043191	0.003615	Gm30363
MCAO 24h v.s. SHAM	mmu_circRNA_27181	chr14	-	21476670	21483639	antisense	2.2	12.1141	4.655	0.00043191	0.003615	Kat6b
MCAO 24h v.s. SHAM	mmu_circRNA_34504	chr2	+	127237460	127238820	exonic	-3.403	10.8092	-7.79	2.70E-06	0.000137	Snrnp200
MCAO 24h v.s. SHAM	mmu_circRNA_24789	chr12	+	21230822	21247373	exonic	2.989	10.8049	6.84	1.09E-05	0.000308	Gm40855
MCAO 24h v.s. SHAM	mmu_circRNA_24789	chr12	+	21230822	21247373	exonic	2.989	10.8049	6.84	1.09E-05	0.000308	Asap2
MCAO 24h v.s. SHAM	mmu_circRNA_19387	chr5	-	124089585	124090263	sense overlapping	2.44	10.502	6.511	1.82E-05	0.000429	Abcb9
MCAO 24h v.s. SHAM	mmu_circRNA_39375	chr5	-	124089587	124090262	exonic	2.363	10.6066	6.51	1.83E-05	0.000429	Abcb9
MCAO 24h v.s. SHAM	mmu_circRNA_42048	chr7	+	105682778	105687385	exonic	2.649	12.6057	7.118	7.17E-06	0.000244	Dnhd1
MCAO 24h v.s. SHAM	mmu_circRNA_36666	chr4	+	40990792	40994180	exonic	-2.045	8.24512	-3.9	0.00178848	0.009948	Nfx1
MCAO 24h v.s. SHAM	mmu_circRNA_34777	chr2	+	152677541	152704941	sense overlapping	2.285	13.5676	5.435	0.00010784	0.00143	H13

MCAO 24h v.s. SHAM	mmu_circRNA_19180	chr17	+	15021913	15051640	sense overlapping	2.021	9.85052	4.598	0.00047995	0.003891	Ermard
MCAO 24h v.s. SHAM	mmu_circRNA_19180	chr17	+	15021913	15051640	sense overlapping	2.021	9.85052	4.598	0.00047995	0.003891	9030025P20Rik
MCAO 24h v.s. SHAM	mmu_circRNA_19180	chr17	+	15021913	15051640	sense overlapping	2.021	9.85052	4.598	0.00047995	0.003891	Gm3255
MCAO 24h v.s. SHAM	mmu_circRNA_008457	chr16	-	89877037	89885125	exonic	2.3	12.2258	6.429	2.08E-05	0.000465	Tiam1
MCAO 24h v.s. SHAM	mmu_circRNA_25839	chr12	+	109729766	109730488	sense overlapping	3.464	7.25567	8.132	1.68E-06	0.000101	Mirg
MCAO 24h v.s. SHAM	mmu_circRNA_35586	chr3	+	73269612	73341175	intronic	3.132	8.29045	6.61	1.56E-05	0.00039	Gm20754
MCAO 24h v.s. SHAM	mmu_circRNA_42423	chr7	+	133650500	133667691	exonic	3.549	11.0595	6.386	2.22E-05	0.000482	Edrf1
MCAO 24h v.s. SHAM	mmu_circRNA_32099	chr18	+	86883565	86911148	intergenic	-2.284	9.05156	-4.56	0.00051557	0.004092	NA
MCAO 24h v.s. SHAM	mmu_circRNA_36886	chr4	-	62329347	62336547	exonic	2.221	10.7924	4.183	0.00103828	0.00675	Fkbp15
MCAO 24h v.s. SHAM	mmu_circRNA_40203	chr6	+	40004144	40207533	sense overlapping	6.279	10.0608	10.16	1.29E-07	4.31E-05	Tmem178b
MCAO 24h v.s. SHAM	mmu_circRNA_013053	chr6	-	86710514	86711679	exonic	2.453	6.86638	4.717	0.00038567	0.003329	Gmcl1
MCAO 24h v.s. SHAM	mmu_circRNA_43956	chr9	+	34536915	34538449	intronic	7.462	9.26161	13.02	6.51E-09	2.24E-05	Kirrel3
MCAO 24h v.s. SHAM	mmu_circRNA_19677	chr1	-	13284813	13286025	intronic	3.49	8.44883	4.852	0.00030219	0.002851	Ncoa2
MCAO 24h v.s. SHAM	mmu_circRNA_31339	chr18	+	12976468	12988385	exonic	4.514	6.90663	6.634	1.50E-05	0.000383	Impact
MCAO 24h v.s. SHAM	mmu_circRNA_33499	chr2	-	48087107	48095756	sense overlapping	4.923	9.1881	7.211	6.24E-06	0.000219	Gm13481
MCAO 24h v.s. SHAM	mmu_circRNA_27547	chr14	+	51994879	51996407	exonic	2.912	6.47832	5.92	4.74E-05	0.000806	Arhgef40
MCAO 24h v.s. SHAM	mmu_circRNA_34968	chr2	-	166617832	166621333	exonic	4.335	11.8013	7.383	4.85E-06	0.000186	Prex1
MCAO 24h v.s. SHAM	mmu_circRNA_20613	chr1	-	105546663	105566250	exonic	3.045	8.66184	7.545	3.83E-06	0.000161	Pign
MCAO 24h v.s. SHAM	mmu_circRNA_20613	chr1	-	105546663	105566250	exonic	3.045	8.66184	7.545	3.83E-06	0.000161	Gm28403
MCAO 24h v.s. SHAM	mmu_circRNA_28615	chr15	-	55749187	55792250	exonic	-2.338	7.26177	-6.41	2.15E-05	0.000473	Sntb1
MCAO 24h v.s. SHAM	mmu_circRNA_19077	chr12	+	88455628	88456716	intronic	5.785	6.53	8.069	1.83E-06	0.000106	Adck1

MCAO 24h v.s. SHAM	mmu_circRNA_000614	chr15	-	35088387	35115645	sense overlapping	2.476	8.16913	6.268	2.69E-05	0.000553	Stk3
MCAO 24h v.s. SHAM	mmu_circRNA_27543	chr14	+	49230063	49237629	antisense	2.276	6.57241	8.264	1.40E-06	8.93E-05	1700011H14Rik
MCAO 24h v.s. SHAM	mmu_circRNA_41052	chr6	-	144155096	144625686	exonic	2.909	10.6932	7.741	2.90E-06	0.000141	NA
MCAO 24h v.s. SHAM	mmu_circRNA_19469	chr7	+	102563267	102563363	antisense	2.097	12.3503	6.375	2.26E-05	0.000488	Trim21
MCAO 24h v.s. SHAM	mmu_circRNA_19608	chr1	-	8624778	8682029	exonic	2.006	6.16824	3.119	0.00800813	0.029538	Sntg1
MCAO 24h v.s. SHAM	mmu_circRNA_45753	chrX	-	139954906	139968599	exonic	2.065	11.6766	6.441	2.04E-05	0.00046	Rbm41
MCAO 24h v.s. SHAM	mmu_circRNA_19470	chr7	-	104315409	104348404	sense overlapping	3.638	12.3217	7.298	5.49E-06	0.0002	Trim5
MCAO 24h v.s. SHAM	mmu_circRNA_31499	chr18	+	32070106	32080577	exonic	-2.017	6.71854	-3.81	0.00208876	0.011081	Iws1
MCAO 24h v.s. SHAM	mmu_circRNA_36544	chr4	+	22025119	22040595	intergenic	3.725	7.03666	7.474	4.25E-06	0.000171	Gm42260
MCAO 24h v.s. SHAM	mmu_circRNA_24051	chr11	-	88001831	88003875	sense overlapping	3.189	9.61851	9.134	4.49E-07	5.38E-05	LOC108168720
MCAO 24h v.s. SHAM	mmu_circRNA_24051	chr11	-	88001831	88003875	sense overlapping	3.189	9.61851	9.134	4.49E-07	5.38E-05	LOC105247666
MCAO 24h v.s. SHAM	mmu_circRNA_23895	chr11	+	83987912	84009550	exonic	2.032	7.03068	6.302	2.54E-05	0.000535	Synrg
MCAO 24h v.s. SHAM	mmu_circRNA_22210	chr10	+	74324357	74396479	exonic	2.59	7.4128	6.456	1.99E-05	0.000453	Pcdh15
MCAO 24h v.s. SHAM	mmu_circRNA_39830	chr6	-	8643972	8658314	exonic	3.437	7.55356	9.183	4.23E-07	5.26E-05	Ica1
MCAO 24h v.s. SHAM	mmu_circRNA_20370	chr1	-	77398493	77426889	exonic	2.031	9.75193	6.107	3.49E-05	0.000657	Epha4
MCAO 24h v.s. SHAM	mmu_circRNA_32047	chr18	-	80808611	80891923	exonic	3.112	12.7347	7.638	3.35E-06	0.000151	Atp9b
MCAO 24h v.s. SHAM	mmu_circRNA_28307	chr15	-	25027225	25364918	sense overlapping	3.573	10.2107	5.935	4.63E-05	0.000797	Gm2824
MCAO 24h v.s. SHAM	mmu_circRNA_19502	chr8	+	122305354	122307643	intronic	-2.007	8.57794	-3.72	0.00252574	0.01277	Zfpm1
MCAO 24h v.s. SHAM	mmu_circRNA_42386	chr7	+	130739811	130753661	exonic	-2.6	7.40021	-3.24	0.00629593	0.024809	Tacc2
MCAO 24h v.s. SHAM	mmu_circRNA_43907	chr9	+	28039050	28045364	intronic	4.307	11.0118	9.238	3.95E-07	5.20E-05	Opcml

MCAO 24h v.s. SHAM	mmu_circRNA_32399	chr19	-	28283651	28317413	exonic	2.146	6.18751	5.912	4.81E-05	0.000816	Glis3
MCAO 24h v.s. SHAM	mmu_circRNA_21095	chr1	-	157230038	157299264	exonic	2.925	8.53058	7.052	7.92E-06	0.000258	Rasal2
MCAO 24h v.s. SHAM	mmu_circRNA_19930	chr1	+	37035636	37063549	exonic	3.045	7.41129	9.449	3.03E-07	5.05E-05	Vwa3b
MCAO 24h v.s. SHAM	mmu_circRNA_25708	chr12	-	101043442	101049818	exonic	-2.129	9.0035	-5.72	6.61E-05	0.001007	Ppp4r3a
MCAO 24h v.s. SHAM	mmu_circRNA_33553	chr2	+	52105434	52120368	exonic	-2.02	6.04643	-4.23	0.00094772	0.006341	Rif1
MCAO 24h v.s. SHAM	mmu_circRNA_31163	chr17	-	90991378	90992144	intronic	3.856	7.0874	3.151	0.00752139	0.028329	Nrxn1
MCAO 24h v.s. SHAM	mmu_circRNA_19351	chr4	-	151830417	151835885	sense overlapping	-2.59	12.4454	-9.88	1.81E-07	4.46E-05	Camta1
MCAO 24h v.s. SHAM	mmu_circRNA_30261	chr17	+	14336546	14348778	exonic	-2.299	7.94725	-3.65	0.00288757	0.014115	Smoc2
MCAO 24h v.s. SHAM	mmu_circRNA_28060	chr14	-	121297134	121308071	exonic	-2.115	6.30441	-4.54	0.00053688	0.004202	Stk24
MCAO 24h v.s. SHAM	mmu_circRNA_19472	chr7	+	108167494	108184865	antisense	2.143	5.73533	8.404	1.16E-06	8.32E-05	Gm39066
MCAO 24h v.s. SHAM	mmu_circRNA_37176	chr4	+	95696959	95769529	exonic	3.428	10.5223	6.228	2.86E-05	0.000578	Fggy
MCAO 24h v.s. SHAM	mmu_circRNA_23788	chr11	+	78283058	78284282	exonic	-3.037	7.11824	-8.31	1.31E-06	8.92E-05	2610507B11Rik
MCAO 24h v.s. SHAM	mmu_circRNA_41355	chr7	+	45575430	45588528	exonic	2.348	6.8683	6.503	1.85E-05	0.000431	Bcat2
MCAO 24h v.s. SHAM	mmu_circRNA_44596	chr9	-	71917004	72006762	exonic	2.849	8.00539	6.814	1.14E-05	0.000318	Tcf12
MCAO 24h v.s. SHAM	mmu_circRNA_003023	chr3	-	51308049	51326032	sense overlapping	-3.336	12.2892	-9.94	1.68E-07	4.38E-05	Elf2
MCAO 24h v.s. SHAM	mmu_circRNA_40266	chr6	+	51562017	51588428	exonic	-2.646	6.35362	-6.14	3.29E-05	0.000629	Snx10
MCAO 24h v.s. SHAM	mmu_circRNA_31992	chr18	+	75894633	75900761	sense overlapping	-2.055	10.9458	-3.76	0.00230622	0.011923	Zbtb7c
MCAO 24h v.s. SHAM	mmu_circRNA_26920	chr13	-	106812207	106886757	exonic	-2.093	9.6158	-4.22	0.00096164	0.006399	Ipo11
MCAO 24h v.s. SHAM	mmu_circRNA_001009	chr19	-	4784723	4794009	sense overlapping	-2.134	11.2561	-5.12	0.00018543	0.002024	Gm21992
MCAO 24h v.s. SHAM	mmu_circRNA_001009	chr19	-	4784723	4794009	sense overlapping	-2.134	11.2561	-5.12	0.00018543	0.002024	Rbm4
MCAO 24h v.s. SHAM	mmu_circRNA_45837	chrX	+	152033698	152047968	exonic	2.799	10.4481	7.93	2.22E-06	0.000121	Smc1a

MCAO 24h v.s. SHAM	mmu_circRNA_38346	chr5	-	29287390	29363955	exonic	2.494	6.81944	7.599	3.55E-06	0.000154	Lmbr1
MCAO 24h v.s. SHAM	mmu_circRNA_19255	chr2	+	104608871	104609187	sense overlapping	-2.253	11.5541	-6.8	1.16E-05	0.000322	Cstf3
MCAO 24h v.s. SHAM	mmu_circRNA_40039	chr6	-	32892291	32968318	exonic	-2.016	6.68128	-7.21	6.26E-06	0.000219	Chchd3
MCAO 24h v.s. SHAM	mmu_circRNA_36353	chr3	+	157960983	157962382	exonic	-2.222	9.79016	-4.71	0.00039381	0.003374	Ankrd13c
MCAO 24h v.s. SHAM	mmu_circRNA_26486	chr13	+	63156545	63240331	exonic	4.655	8.743	6.617	1.55E-05	0.000389	2010111I01Rik
MCAO 24h v.s. SHAM	mmu_circRNA_30333	chr17	+	25167296	25168665	exonic	-2.259	9.46242	-4.47	0.0006101	0.004614	Ccdc154
MCAO 24h v.s. SHAM	mmu_circRNA_37263	chr4	+	102760442	102870724	exonic	2.217	8.74199	7.017	8.35E-06	0.000264	Sgip1
MCAO 24h v.s. SHAM	mmu_circRNA_000673	chr2	+	31778333	31779216	exonic	2.28	7.32182	6.109	3.48E-05	0.000657	Abl1
MCAO 24h v.s. SHAM	mmu_circRNA_22800	chr10	-	128578099	128579092	exonic	-2.241	11.4194	-4.84	0.00030624	0.002869	Erbb3
MCAO 24h v.s. SHAM	mmu_circRNA_013935	chr19	-	4784723	4785567	intronic	-2.006	11.1684	-4.36	0.00075019	0.00539	Gm21992
MCAO 24h v.s. SHAM	mmu_circRNA_013935	chr19	-	4784723	4785567	intronic	-2.006	11.1684	-4.36	0.00075019	0.00539	Rbm4
MCAO 24h v.s. SHAM	mmu_circRNA_21656	chr10	+	11289519	11291572	exonic	-2.889	9.68067	-7.03	8.22E-06	0.000261	Fbxo30
MCAO 24h v.s. SHAM	mmu_circRNA_001219	chr3	+	28532887	28542098	exonic	-2.025	10.3558	-5.15	0.00017841	0.001962	Tnik
MCAO 24h v.s. SHAM	mmu_circRNA_44888	chr9	-	96576285	96602807	exonic	-2.257	8.0109	-6.05	3.82E-05	0.000698	Rasa2
MCAO 24h v.s. SHAM	mmu_circRNA_44888	chr9	-	96576285	96602807	exonic	-2.257	8.0109	-6.05	3.82E-05	0.000698	LOC108167675
MCAO 24h v.s. SHAM	mmu_circRNA_30386	chr17	-	27596723	27596834	exonic	-2.493	7.11418	-3.79	0.00217838	0.011413	Nudt3
MCAO 24h v.s. SHAM	mmu_circRNA_42040	chr7	+	102354500	102421530	exonic	-2.493	6.90883	-5.91	4.85E-05	0.000819	Stim1
MCAO 24h v.s. SHAM	mmu_circRNA_40806	chr6	+	116313844	116315642	exonic	-2.275	7.4558	-6.22	2.92E-05	0.000585	Zfand4
MCAO 24h v.s. SHAM	mmu_circRNA_38907	chr5	+	86891227	86905024	antisense	2.307	13.7169	7.895	2.33E-06	0.000125	Ugt2b34
MCAO 24h v.s. SHAM	mmu_circRNA_24227	chr11	+	104153520	104163889	exonic	3.141	9.24502	7.211	6.25E-06	0.000219	Crhr1

MCAO 24h v.s. SHAM	mmu_circRNA_29634	chr16	-	35844727	35853758	exonic	2.314	6.98088	3.861	0.00191374	0.01044	Parp14
MCAO 24h v.s. SHAM	mmu_circRNA_30420	chr17	+	29282059	29282891	exonic	3.236	8.54857	9.793	2.00E-07	4.51E-05	BC004004
MCAO 24h v.s. SHAM	mmu_circRNA_30419	chr17	+	29277856	29282891	exonic	4.271	7.19489	13.9	2.88E-09	1.49E-05	BC004004
MCAO 24h v.s. SHAM	mmu_circRNA_42243	chr7	+	120899367	120899546	exonic	-2.244	7.39012	-5.14	0.00018183	0.001993	Eef2k
MCAO 24h v.s. SHAM	mmu_circRNA_25314	chr12	+	72783682	72786789	exonic	-2.482	6.82624	-5.43	0.000109	0.001443	Ppm1a
MCAO 24h v.s. SHAM	mmu_circRNA_29553	chr16	+	32884123	32898470	exonic	2.87	7.95559	5.17	0.00017125	0.001905	Fyttd1
MCAO 24h v.s. SHAM	mmu_circRNA_21094	chr1	-	157192746	157299264	exonic	2.173	6.70972	7.193	6.42E-06	0.000224	Rasal2
MCAO 24h v.s. SHAM	mmu_circRNA_010637	chr8	+	110632029	110637033	exonic	-2.055	7.09314	-4.29	0.00084184	0.005887	Vac14
MCAO 24h v.s. SHAM	mmu_circRNA_45396	chrX	+	20429022	20502512	exonic	3.189	8.11075	7.582	3.63E-06	0.000156	Jade3
MCAO 24h v.s. SHAM	mmu_circRNA_27645	chr14	-	59771638	59860313	exonic	2.23	7.65353	7.422	4.58E-06	0.000178	Atp8a2
MCAO 24h v.s. SHAM	mmu_circRNA_38525	chr5	+	37278084	37278931	exonic	4.446	8.0808	8.291	1.35E-06	8.93E-05	Crmp1
MCAO 24h v.s. SHAM	mmu_circRNA_33115	chr2	+	22901280	22915528	sense overlapping	-2.308	7.58996	-6.89	1.01E-05	0.000291	Pdss1
MCAO 24h v.s. SHAM	mmu_circRNA_42572	chr8	-	13605861	13631892	exonic	4.745	11.1948	7.997	2.02E-06	0.000114	Rasa3
MCAO 24h v.s. SHAM	mmu_circRNA_40598	chr6	-	97323654	97329564	exonic	-2.297	9.04445	-5.66	7.34E-05	0.001077	Frmd4b
MCAO 24h v.s. SHAM	mmu_circRNA_42191	chr7	+	117548583	117593739	exonic	2.776	8.13329	5.324	0.00013078	0.001619	Xylt1
MCAO 24h v.s. SHAM	mmu_circRNA_44389	chr9	-	61924631	61926518	exonic	2.769	11.5111	6.938	9.42E-06	0.000279	Kif23
MCAO 24h v.s. SHAM	mmu_circRNA_40047	chr6	+	33296732	33442039	exonic	-2.046	11.0256	-4.81	0.00032378	0.002975	Exoc4
MCAO 24h v.s. SHAM	mmu_circRNA_19238	chr2	-	30110202	30111334	sense overlapping	-2.461	8.70043	-7.69	3.11E-06	0.000144	Zer1
MCAO 24h v.s. SHAM	mmu_circRNA_36129	chr3	-	126976050	127023390	exonic	2.726	7.38496	6.636	1.50E-05	0.000383	Ank2
MCAO 24h v.s. SHAM	mmu_circRNA_28251	chr15	+	12150172	12156408	exonic	-2.851	6.54151	-8.57	9.33E-07	7.61E-05	Zfr
MCAO 24h v.s. SHAM	mmu_circRNA_25527	chr12	-	85320713	85336485	exonic	-2.459	9.31009	-6.77	1.22E-05	0.000332	Nek9

MCAO 24h v.s. SHAM	mmu_circRNA_39331	chr5	+	122714398	122719226	exonic	-2.454	6.64769	-4.87	0.00029453	0.00281	P2rx4
MCAO 24h v.s. SHAM	mmu_circRNA_22421	chr10	+	90510595	90644556	exonic	4.587	8.96316	6.574	1.65E-05	0.000403	Anks1b
MCAO 24h v.s. SHAM	mmu_circRNA_43389	chr8	+	105178592	105202547	exonic	-2.889	8.21995	-8.48	1.04E-06	7.91E-05	Cbfb
MCAO 24h v.s. SHAM	mmu_circRNA_22066	chr10	+	58983920	59007137	exonic	2.712	11.2729	8.671	8.15E-07	7.43E-05	Sh3rf3
MCAO 24h v.s. SHAM	mmu_circRNA_35066	chr2	-	181081454	181114729	exonic	2.294	8.86149	6.901	9.96E-06	0.00029	Kcnq2
MCAO 24h v.s. SHAM	mmu_circRNA_23643	chr11	-	72456302	72474770	exonic	-4.25	8.98581	-6.67	1.43E-05	0.000371	Spns2
MCAO 24h v.s. SHAM	mmu_circRNA_23253	chr11	+	44531275	44551819	exonic	2.71	11.7915	7.106	7.31E-06	0.000246	Rnf145
MCAO 24h v.s. SHAM	mmu_circRNA_44128	chr9	-	49543010	49569900	exonic	2.364	7.20637	6.163	3.18E-05	0.000615	Ncam1
MCAO 24h v.s. SHAM	mmu_circRNA_44128	chr9	-	49543010	49569900	exonic	2.364	7.20637	6.163	3.18E-05	0.000615	Gm11149
MCAO 24h v.s. SHAM	mmu_circRNA_39121	chr5	-	107795698	107799260	exonic	-4.347	10.891	-10.2	1.17E-07	4.18E-05	Evi5
MCAO 24h v.s. SHAM	mmu_circRNA_22574	chr10	-	107563134	107608453	exonic	2.438	7.472	7.733	2.93E-06	0.000141	Gm36177
MCAO 24h v.s. SHAM	mmu_circRNA_22574	chr10	-	107563134	107608453	exonic	2.438	7.472	7.733	2.93E-06	0.000141	Ptprq
MCAO 24h v.s. SHAM	mmu_circRNA_19729	chr1	-	21424048	21469564	exonic	-2.697	8.21758	-4.72	0.00038687	0.003334	Kcnq5
MCAO 24h v.s. SHAM	mmu_circRNA_31064	chr17	-	85065631	85076825	exonic	3.061	6.80618	7.391	4.79E-06	0.000185	Prepl
MCAO 24h v.s. SHAM	mmu_circRNA_43320	chr8	-	91299228	91310429	exonic	4.147	8.47509	6.183	3.08E-05	0.000606	Rpgrip1l
MCAO 24h v.s. SHAM	mmu_circRNA_43320	chr8	-	91299228	91310429	exonic	4.147	8.47509	6.183	3.08E-05	0.000606	Gm36163
MCAO 24h v.s. SHAM	mmu_circRNA_001832	chr6	-	82730767	82739674	exonic	3.986	12.3907	8.732	7.53E-07	7.00E-05	Hk2
MCAO 24h v.s. SHAM	mmu_circRNA_32153	chr19	-	3602234	3605494	exonic	2.162	10.6637	4.412	0.00067601	0.004985	Lrp5
MCAO 24h v.s. SHAM	mmu_circRNA_009370	chr17	+	39844697	39845062	sense overlapping	-2.35	12.5074	-3.09	0.00842264	0.030629	NA
MCAO 24h v.s. SHAM	mmu_circRNA_20530	chr1	+	91088633	91112297	exonic	2.585	9.31812	7.621	3.43E-06	0.000152	Lrrfip1

MCAO 24h v.s. SHAM	mmu_circRNA_41232	chr7	-	27174421	27176238	exonic	2.211	7.84351	8.343	1.26E-06	8.67E-05	Rab4b
MCAO 24h v.s. SHAM	mmu_circRNA_37286	chr4	+	103754180	103764967	intronic	6.789	8.22307	9.372	3.34E-07	5.15E-05	Dab1
MCAO 24h v.s. SHAM	mmu_circRNA_32308	chr19	+	21797326	21818011	exonic	2.03	10.4919	6.164	3.18E-05	0.000615	Tmem2
MCAO 24h v.s. SHAM	mmu_circRNA_005949	chr1	-	190885333	190899571	exonic	-2.25	10.5752	-5.95	4.53E-05	0.000787	Rps6kc1
MCAO 24h v.s. SHAM	mmu_circRNA_004682	chr18	-	61140790	61141790	exonic	-3.44	9.3288	-6.63	1.50E-05	0.000383	Hmgxb3
MCAO 24h v.s. SHAM	mmu_circRNA_29140	chr16	-	4417852	4419588	exonic	-2.628	7.29825	-4.83	0.00031237	0.002916	Adcy9
MCAO 24h v.s. SHAM	mmu_circRNA_20259	chr1	-	64034531	64042437	sense overlapping	3.462	10.5876	7.736	2.91E-06	0.000141	Klf7
MCAO 24h v.s. SHAM	mmu_circRNA_003057	chr18	+	11923359	11926364	exonic	3.419	9.66942	10.22	1.22E-07	4.19E-05	Cables1
MCAO 24h v.s. SHAM	mmu_circRNA_23281	chr11	+	49818579	49819254	exonic	4.772	6.99354	6.614	1.55E-05	0.000389	Gm36507
MCAO 24h v.s. SHAM	mmu_circRNA_23281	chr11	+	49818579	49819254	exonic	4.772	6.99354	6.614	1.55E-05	0.000389	Gfpt2
MCAO 24h v.s. SHAM	mmu_circRNA_26660	chr13	-	91022569	91040988	exonic	-2.236	7.40292	-4.82	0.00031786	0.002941	Atg10
MCAO 24h v.s. SHAM	mmu_circRNA_001643	chr11	+	72712156	72714483	exonic	-2.161	7.72653	-4.66	0.00042706	0.003587	Ankfy1
MCAO 24h v.s. SHAM	mmu_circRNA_19311	chr3	-	153411461	153411874	sense overlapping	-2.232	11.059	-6.06	3.76E-05	0.000693	St6galnac3
MCAO 24h v.s. SHAM	mmu_circRNA_45921	chrX	+	160523271	160533077	exonic	-4.418	12.4019	-8.48	1.05E-06	7.93E-05	Phka2
MCAO 24h v.s. SHAM	mmu_circRNA_19441	chr6	+	134062060	134066465	intronic	-2.895	11.7909	-7.71	3.05E-06	0.000142	Etv6
MCAO 24h v.s. SHAM	mmu_circRNA_38278	chr5	-	24866078	24889188	exonic	-2.457	8.14273	-5.61	7.98E-05	0.001152	Prkag2
MCAO 24h v.s. SHAM	mmu_circRNA_33594	chr2	-	58500483	58537873	exonic	2.031	12.2897	7.003	8.53E-06	0.000267	Acvr1
MCAO 24h v.s. SHAM	mmu_circRNA_002280	chr19	-	60764031	60764233	exonic	-3.018	10.7955	-7.44	4.49E-06	0.000177	Eif3a
MCAO 24h v.s. SHAM	mmu_circRNA_34709	chr2	-	144259056	144260835	exonic	-2.042	8.47027	-4.71	0.00038915	0.003344	Snx5
MCAO 24h v.s. SHAM	mmu_circRNA_45731	chrX	+	129902899	129972609	exonic	3.248	7.41611	6.767	1.22E-05	0.000332	Diaph2
MCAO 24h v.s. SHAM	mmu_circRNA_45047	chr9	+	106889019	106889196	antisense	5.586	8.9381	8.949	5.68E-07	6.05E-05	Manf

MCAO 24h v.s. SHAM	mmu_circRNA_30480	chr17	-	33658387	33677440	exonic	-2.515	7.68585	-6.45	2.01E-05	0.000455	Hnrnpm
MCAO 24h v.s. SHAM	mmu_circRNA_42655	chr8	-	24687881	24697228	exonic	-2.033	9.31941	-4.12	0.00117517	0.0073	Adam3
MCAO 24h v.s. SHAM	mmu_circRNA_23947	chr11	+	85326863	85339796	exonic	2.418	10.0317	4.393	0.00070004	0.005093	Ppm1d
MCAO 24h v.s. SHAM	mmu_circRNA_20456	chr1	+	86086029	86090190	exonic	3.453	9.70433	9.173	4.28E-07	5.26E-05	Psmd1
MCAO 24h v.s. SHAM	mmu_circRNA_32211	chr19	-	7094329	7097229	sense overlapping	2.892	7.08832	9.956	1.65E-07	4.38E-05	Macrod1
MCAO 24h v.s. SHAM	mmu_circRNA_32211	chr19	-	7094329	7097229	sense overlapping	2.892	7.08832	9.956	1.65E-07	4.38E-05	Flrt1
MCAO 24h v.s. SHAM	mmu_circRNA_015637	chr1	-	156900585	156907778	exonic	-2.169	11.7772	-6.1	3.54E-05	0.00066	Ralgps2
MCAO 24h v.s. SHAM	mmu_circRNA_002466	chr7	-	133025712	133081860	exonic	-2.059	7.19414	-5.85	5.38E-05	0.000877	Ctbp2
MCAO 24h v.s. SHAM	mmu_circRNA_40539	chr6	+	87904087	87905267	exonic	-3.204	6.6404	-5.1	0.00019372	0.002092	Copg1
MCAO 24h v.s. SHAM	mmu_circRNA_013298	chr17	+	74668758	74670382	exonic	-2.964	10.652	-7.96	2.14E-06	0.000117	Birc6
MCAO 24h v.s. SHAM	mmu_circRNA_24983	chr12	+	40626336	40710901	exonic	3.104	6.23783	6.069	3.71E-05	0.000687	Dock4
MCAO 24h v.s. SHAM	mmu_circRNA_37675	chr4	+	128393489	128419598	exonic	2.353	6.84072	6.878	1.03E-05	0.000297	Csmd2
MCAO 24h v.s. SHAM	mmu_circRNA_007649	chr14	-	52161680	52184020	sense overlapping	-2.321	9.00605	-4.06	0.0013075	0.007876	Supt16
MCAO 24h v.s. SHAM	mmu_circRNA_27348	chr14	-	30045214	30047371	exonic	-3.68	9.68138	-4.66	0.00042588	0.003586	Cacna1d
MCAO 24h v.s. SHAM	mmu_circRNA_31588	chr18	+	35571842	35588453	exonic	-2.249	9.7815	-4.78	0.00034117	0.003087	Matr3
MCAO 24h v.s. SHAM	mmu_circRNA_23083	chr11	+	29182446	29197209	exonic	-2.444	8.04744	-5.22	0.00015612	0.001805	Ppp4r3b
MCAO 24h v.s. SHAM	mmu_circRNA_22301	chr10	+	82709008	82724396	exonic	-2.42	7.89281	-5.64	7.55E-05	0.001105	Hcfc2
MCAO 24h v.s. SHAM	mmu_circRNA_22970	chr11	-	22137837	22151892	exonic	4.435	9.42401	5.707	6.78E-05	0.001025	Ehbp1
MCAO 24h v.s. SHAM	mmu_circRNA_37158	chr4	-	94575165	94584002	exonic	-3.111	7.01303	-7.32	5.30E-06	0.000197	Plaa
MCAO 24h v.s. SHAM	mmu_circRNA_26317	chr13	+	49828935	49830616	intergenic	2.725	7.87637	4.387	0.00070797	0.005144	LOC102631805

MCAO 24h v.s. SHAM	mmu_circRNA_40898	chr6	+	120402505	120428377	exonic	4.234	6.54009	6.911	9.81E-06	0.000287	Kdm5a
MCAO 24h v.s. SHAM	mmu_circRNA_21073	chr1	-	156365615	156382477	exonic	-2.495	10.8483	-4.66	0.00042691	0.003587	Axdnd1
MCAO 24h v.s. SHAM	mmu_circRNA_35735	chr3	-	88447990	88450112	exonic	2.701	8.73501	3.338	0.0052414	0.021779	Sema4a
MCAO 24h v.s. SHAM	mmu_circRNA_43789	chr9	-	15297842	15306619	sense overlapping	3.265	6.82082	7.846	2.50E-06	0.00013	4931406C07Rik
MCAO 24h v.s. SHAM	mmu_circRNA_24141	chr11	-	97240928	97267679	exonic	-2.149	7.88897	-5.99	4.25E-05	0.000751	Npepps
MCAO 24h v.s. SHAM	mmu_circRNA_36483	chr4	-	12057315	12063830	exonic	2.863	13.9213	5.873	5.13E-05	0.000851	Tmem67
MCAO 24h v.s. SHAM	mmu_circRNA_23142	chr11	-	30219578	30219772	exonic	-2.631	7.8774	-3.64	0.00290183	0.014158	Sptbn1
MCAO 24h v.s. SHAM	mmu_circRNA_23334	chr11	-	53262263	53271079	exonic	2.966	8.51314	3.136	0.00774978	0.028905	Hspa4
MCAO 24h v.s. SHAM	mmu_circRNA_23275	chr11	+	49260570	49261813	sense overlapping	2.007	6.93632	6.851	1.07E-05	0.000304	Mgat1
MCAO 24h v.s. SHAM	mmu_circRNA_006731	chr15	+	76904375	76904450	sense overlapping	-2.739	6.87196	-8.41	1.16E-06	8.32E-05	Rpl8
MCAO 24h v.s. SHAM	mmu_circRNA_37699	chr4	-	129712103	129730325	exonic	-4.294	12.3654	-5.47	0.00010082	0.001362	Khdrbs1
MCAO 24h v.s. SHAM	mmu_circRNA_32678	chr19	-	45757904	45777017	exonic	2.244	6.84811	7.046	7.99E-06	0.000259	Mgea5
MCAO 24h v.s. SHAM	mmu_circRNA_45217	chr9	-	114610644	114622962	exonic	2.817	6.80411	9.331	3.51E-07	5.17E-05	Cnot10
MCAO 24h v.s. SHAM	mmu_circRNA_36037	chr3	+	118781835	118999385	exonic	4.279	8.9245	6.394	2.19E-05	0.000479	Dpyd
MCAO 24h v.s. SHAM	mmu_circRNA_18935	chr1	-	52708163	52709758	sense overlapping	2.29	8.5999	3.4	0.00464428	0.019958	Mfsd6
MCAO 24h v.s. SHAM	mmu_circRNA_010777	chr14	-	23494538	23509900	exonic	5.3	9.39607	9.957	1.64E-07	4.38E-05	Kcnma1
MCAO 24h v.s. SHAM	mmu_circRNA_41390	chr7	+	48799828	48818720	exonic	3.155	11.2406	9.359	3.39E-07	5.15E-05	Zdhhc13
MCAO 24h v.s. SHAM	mmu_circRNA_23573	chr11	+	68011314	68020998	exonic	-2.259	6.66079	-7.82	2.60E-06	0.000134	Stx8
MCAO 24h v.s. SHAM	mmu_circRNA_22124	chr10	-	63016079	63019527	exonic	-2.351	7.1404	-5.52	9.25E-05	0.001288	Hnrnph3
MCAO 24h v.s. SHAM	mmu_circRNA_22124	chr10	-	63016079	63019527	exonic	-2.351	7.1404	-5.52	9.25E-05	0.001288	Rufy2
MCAO 24h v.s. SHAM	mmu_circRNA_39080	chr5	-	104085009	104094767	exonic	2.636	7.17418	4.311	0.00081633	0.005741	Sparcl1

MCAO 24h v.s. SHAM	mmu_circRNA_34179	chr2	+	106699287	106744881	exonic	-2.252	8.84819	-3.59	0.00323087	0.015273	Mpped2
MCAO 24h v.s. SHAM	mmu_circRNA_24038	chr11	-	87388247	87397757	exonic	-3.183	6.88192	-6.88	1.02E-05	0.000295	Rad51c
MCAO 24h v.s. SHAM	mmu_circRNA_018929	chr5	-	67729791	67751085	exonic	-2.142	7.73358	-7.05	7.93E-06	0.000258	Atp8a1
MCAO 24h v.s. SHAM	mmu_circRNA_20395	chr1	-	80290081	80323056	exonic	-2.735	9.39239	-6.18	3.11E-05	0.000609	Cul3
MCAO 24h v.s. SHAM	mmu_circRNA_33674	chr2	+	62158205	62257605	exonic	3.191	9.27946	8.283	1.37E-06	8.93E-05	Slc4a10
MCAO 24h v.s. SHAM	mmu_circRNA_33961	chr2	-	77719450	77720699	exonic	-2.472	7.80619	-4.02	0.00141326	0.008371	Zfp385b
MCAO 24h v.s. SHAM	mmu_circRNA_43608	chr8	-	124694829	124714283	exonic	2.256	8.66781	5.425	0.00010975	0.001451	Ttc13
MCAO 24h v.s. SHAM	mmu_circRNA_007853	chr10	+	14004200	14086082	sense overlapping	-3.055	12.3794	-6.42	2.10E-05	0.000465	Hivep2
MCAO 24h v.s. SHAM	mmu_circRNA_33862	chr2	-	73373614	73374014	exonic	3.393	9.08106	5.289	0.00013898	0.00168	Gpr155
MCAO 24h v.s. SHAM	mmu_circRNA_33862	chr2	-	73373614	73374014	exonic	3.393	9.08106	5.289	0.00013898	0.00168	Gm13709
MCAO 24h v.s. SHAM	mmu_circRNA_19052	chr12	+	3442471	3457211	sense overlapping	-2.605	7.86494	-6.56	1.68E-05	0.000408	Asxl2
MCAO 24h v.s. SHAM	mmu_circRNA_31462	chr18	-	25486793	25486965	exonic	-2.743	8.13496	-5.72	6.58E-05	0.001005	Celf4
MCAO 24h v.s. SHAM	mmu_circRNA_44487	chr9	-	65858245	65881060	exonic	2.277	9.42551	5.517	9.37E-05	0.001293	Trip4
MCAO 24h v.s. SHAM	mmu_circRNA_010789	chr15	-	96609855	96624169	exonic	3.126	11.0649	8.52	9.95E-07	7.81E-05	Slc38a1
MCAO 24h v.s. SHAM	mmu_circRNA_30673	chr17	+	57419321	57450101	exonic	3.339	7.19761	7.773	2.77E-06	0.000137	Adgre1
MCAO 24h v.s. SHAM	mmu_circRNA_37524	chr4	+	119872036	119872942	intronic	2.105	11.0006	6.452	2.00E-05	0.000455	Hivep3
MCAO 24h v.s. SHAM	mmu_circRNA_32230	chr19	+	8873513	8873846	exonic	-2.613	8.22412	-7.24	5.98E-06	0.000213	Ubxn1
MCAO 24h v.s. SHAM	mmu_circRNA_41526	chr7	-	61276438	61282828	intronic	3.545	9.4282	7.092	7.46E-06	0.000248	Gm31445
MCAO 24h v.s. SHAM	mmu_circRNA_41526	chr7	-	61276438	61282828	intronic	3.545	9.4282	7.092	7.46E-06	0.000248	A230006K03Rik
MCAO 24h v.s. SHAM	mmu_circRNA_33702	chr2	-	65482150	65495396	exonic	2.057	6.63896	7.318	5.33E-06	0.000197	Scn3a

MCAO 24h v.s. SHAM	mmu_circRNA_36472	chr4	-	11338721	11353463	exonic	-2.127	6.53488	-4.98	0.00023974	0.002427	Esrp1
MCAO 24h v.s. SHAM	mmu_circRNA_18974	chr10	+	40394322	40408366	intronic	-2.722	10.8707	-6.75	1.26E-05	0.000339	Cdk19
MCAO 24h v.s. SHAM	mmu_circRNA_35932	chr3	+	104455486	104456848	antisense	2.869	5.93977	7.707	3.04E-06	0.000142	Lrig2
MCAO 24h v.s. SHAM	mmu_circRNA_017685	chr16	+	5953418	5956863	intronic	3.025	11.6724	6.948	9.27E-06	0.000277	Rbfox1
MCAO 24h v.s. SHAM	mmu_circRNA_39229	chr5	+	117209553	117228079	exonic	-2.449	7.66799	-6.6	1.57E-05	0.000392	Taok3
MCAO 24h v.s. SHAM	mmu_circRNA_43010	chr8	-	70405258	70409011	exonic	2.139	10.4832	5.972	4.36E-05	0.000765	Crtc1
MCAO 24h v.s. SHAM	mmu_circRNA_002219	chr14	-	36918433	36921652	sense overlapping	-2.188	6.96671	-7.41	4.68E-06	0.000181	Ccser2
MCAO 24h v.s. SHAM	mmu_circRNA_25612	chr12	-	91266735	91348876	exonic	-2.445	7.00466	-5.92	4.72E-05	0.000805	Cep128
MCAO 24h v.s. SHAM	mmu_circRNA_20241	chr1	-	63078952	63093730	exonic	2.749	9.37306	3.663	0.00279307	0.01379	Ino80d
MCAO 24h v.s. SHAM	mmu_circRNA_39553	chr5	+	137795490	137801096	exonic	-2.557	7.97895	-3.28	0.00590651	0.023626	Zcwpw1
MCAO 24h v.s. SHAM	mmu_circRNA_22771	chr10	+	127092733	127092873	sense overlapping	3.208	11.4638	9.93	1.70E-07	4.38E-05	Agap2
MCAO 24h v.s. SHAM	mmu_circRNA_37852	chr4	-	140067136	140157573	exonic	-2.405	11.488	-7.22	6.20E-06	0.000219	Igsf21
MCAO 24h v.s. SHAM	mmu_circRNA_26826	chr13	-	98884514	98890790	exonic	3.029	10.3785	8.525	9.87E-07	7.81E-05	Kng2
MCAO 24h v.s. SHAM	mmu_circRNA_30482	chr17	+	34416466	34421987	exonic	3.025	12.0496	9.428	3.12E-07	5.11E-05	BC051142
MCAO 24h v.s. SHAM	mmu_circRNA_013924	chr4	-	136770955	136774644	exonic	-2.183	7.9129	-2.86	0.01332667	0.043198	Ephb2
MCAO 24h v.s. SHAM	mmu_circRNA_20290	chr1	-	66721804	66778108	exonic	-3.105	12.1603	-6.52	1.78E-05	0.000423	Kansl1l
MCAO 24h v.s. SHAM	mmu_circRNA_38185	chr5	-	20801901	20855201	exonic	2.276	7.96403	5.671	7.21E-05	0.001064	Phtf2
MCAO 24h v.s. SHAM	mmu_circRNA_19014	chr11	-	53891146	53969605	sense overlapping	2.369	7.83329	5.319	0.00013183	0.001626	Slc22a5
MCAO 24h v.s. SHAM	mmu_circRNA_37006	chr4	-	83260881	83271218	exonic	2.776	9.54519	5.963	4.42E-05	0.000773	Ttc39b
MCAO 24h v.s. SHAM	mmu_circRNA_38874	chr5	-	84067359	84156536	exonic	3.207	10.7494	7.618	3.45E-06	0.000152	Epha5
MCAO 24h v.s. SHAM	mmu_circRNA_36730	chr4	+	46167301	46170073	exonic	2.315	11.0736	5.884	5.04E-05	0.000838	Ncbp1

MCAO 24h v.s. SHAM	mmu_circRNA_36730	chr4	+	46167301	46170073	exonic	2.315	11.0736	5.884	5.04E-05	0.000838	Xpa
MCAO 24h v.s. SHAM	mmu_circRNA_25808	chr12	+	106042836	106062808	exonic	-2.147	8.58433	-5.7	6.87E-05	0.001035	Vrk1
MCAO 24h v.s. SHAM	mmu_circRNA_42057	chr7	+	109020516	109021029	exonic	-2.312	9.01553	-5.05	0.00021143	0.00223	Tub
MCAO 24h v.s. SHAM	mmu_circRNA_44966	chr9	+	101100570	101124015	sense overlapping	-2.19	10.7835	-4.92	0.00026832	0.002633	Ppp2r3a
MCAO 24h v.s. SHAM	mmu_circRNA_38627	chr5	+	53846200	53883694	exonic	2.081	8.10199	6.189	3.05E-05	0.000605	Tbc1d19
MCAO 24h v.s. SHAM	mmu_circRNA_44220	chr9	+	55397740	55400647	exonic	2.982	9.17902	7.082	7.57E-06	0.00025	Tmem266
MCAO 24h v.s. SHAM	mmu_circRNA_27768	chr14	+	70895771	70926072	exonic	2.028	7.55474	5.599	8.15E-05	0.001167	Gfra2
MCAO 24h v.s. SHAM	mmu_circRNA_28661	chr15	-	66156315	66161889	intronic	3.216	8.5502	7.887	2.36E-06	0.000125	Kcnq3
MCAO 24h v.s. SHAM	mmu_circRNA_25433	chr12	+	81946930	81967148	exonic	-2.964	7.14595	-4.82	0.00032043	0.002952	Pcnx
MCAO 24h v.s. SHAM	mmu_circRNA_21247	chr1	+	167830545	167832894	exonic	-2.703	7.25257	-5.2	0.00016256	0.001854	Lmx1a
MCAO 24h v.s. SHAM	mmu_circRNA_015506	chr17	+	78313123	78315746	exonic	-5.696	9.65211	-6.48	1.92E-05	0.000441	Crim1
MCAO 24h v.s. SHAM	mmu_circRNA_41878	chr7	-	92584465	92631662	exonic	-6.572	10.4762	-9.31	3.58E-07	5.17E-05	Ankrd42
MCAO 24h v.s. SHAM	mmu_circRNA_004941	chr13	-	64243633	64248499	sense overlapping	2.367	8.75373	7.029	8.21E-06	0.000261	Cdc14b
MCAO 24h v.s. SHAM	mmu_circRNA_36337	chr3	-	153411461	153509497	exonic	-3.115	8.97146	-8.54	9.71E-07	7.77E-05	St6galnac3
MCAO 24h v.s. SHAM	mmu_circRNA_32224	chr19	+	8749665	8750027	exonic	-2.422	10.1673	-5.31	0.00013503	0.001648	Stx5a
MCAO 24h v.s. SHAM	mmu_circRNA_014406	chr15	+	85359453	85365359	exonic	3.047	9.10438	5.61	7.99E-05	0.001152	Atxn10
MCAO 24h v.s. SHAM	mmu_circRNA_21750	chr10	+	21928341	21928836	sense overlapping	3.25	12.9902	7.496	4.11E-06	0.000169	Sgk1
MCAO 24h v.s. SHAM	mmu_circRNA_35996	chr3	+	109574105	109628167	exonic	-2.635	6.84934	-7.15	6.84E-06	0.000237	Vav3
MCAO 24h v.s. SHAM	mmu_circRNA_31039	chr17	-	83587656	83588379	sense overlapping	3.071	10.9519	6.6	1.59E-05	0.000393	Kcng3
MCAO 24h v.s. SHAM	mmu_circRNA_22664	chr10	+	116349965	116361307	exonic	2.14	7.27645	4.066	0.00129552	0.007812	Ptprb

MCAO 24h v.s. SHAM	mmu_circRNA_015839	chr16	-	73956487	73985712	exonic	2.267	5.96801	6.205	2.97E-05	0.000594	Robo2
MCAO 24h v.s. SHAM	mmu_circRNA_32300	chr19	-	20856674	20870488	exonic	-2.157	6.17278	-4.87	0.00029213	0.002793	Tmc1
MCAO 24h v.s. SHAM	mmu_circRNA_42683	chr8	+	25640576	25673434	exonic	-3.085	8.19164	-8.9	6.04E-07	6.23E-05	Nsd3
MCAO 24h v.s. SHAM	mmu_circRNA_20390	chr1	-	79796825	79821557	exonic	2.518	7.7575	8.793	6.95E-07	6.83E-05	Serpine2
MCAO 24h v.s. SHAM	mmu_circRNA_29357	chr16	+	17257334	17259073	sense overlapping	-2.002	9.29371	-5.01	0.00022884	0.002337	Hic2
MCAO 24h v.s. SHAM	mmu_circRNA_19780	chr1	+	24383303	24390303	antisense	3.913	6.42785	12.64	9.30E-09	2.40E-05	Col19a1
MCAO 24h v.s. SHAM	mmu_circRNA_30343	chr17	-	25841051	25841482	exonic	4.352	8.30124	11.33	3.52E-08	3.37E-05	Rhot2
MCAO 24h v.s. SHAM	mmu_circRNA_24936	chr12	-	36020489	36024072	exonic	2.188	8.25874	4.558	0.00051594	0.004092	Tspan13
MCAO 24h v.s. SHAM	mmu_circRNA_35559	chr3	+	69009483	69019325	exonic	2.047	6.57712	7.183	6.51E-06	0.000226	Smc4
MCAO 24h v.s. SHAM	mmu_circRNA_20093	chr1	-	54486469	54497627	exonic	2.387	8.6377	6.311	2.51E-05	0.000529	Pgap1
MCAO 24h v.s. SHAM	mmu_circRNA_30514	chr17	-	44551374	44555457	intronic	2.079	6.65745	6.533	1.76E-05	0.000422	Runx2
MCAO 24h v.s. SHAM	mmu_circRNA_36074	chr3	-	120797195	120799747	exonic	2.326	6.0088	6.791	1.18E-05	0.000325	6530403H02Rik
MCAO 24h v.s. SHAM	mmu_circRNA_29158	chr16	+	5952576	5956863	intronic	3.26	10.8036	7.714	3.01E-06	0.000142	Rbfox1
MCAO 24h v.s. SHAM	mmu_circRNA_23191	chr11	-	33283907	33452549	exonic	3.267	6.6821	6.398	2.18E-05	0.000478	Ranbp17
MCAO 24h v.s. SHAM	mmu_circRNA_21952	chr10	-	49355908	49523855	exonic	2.221	6.87361	6.058	3.78E-05	0.000694	Grik2
MCAO 24h v.s. SHAM	mmu_circRNA_004768	chr19	+	8874187	8874255	sense overlapping	3.176	9.4591	6.722	1.31E-05	0.000345	Ubxn1
MCAO 24h v.s. SHAM	mmu_circRNA_19355	chr5	+	3968576	3981215	sense overlapping	-3.306	8.0641	-5.35	0.00012555	0.001582	Akap9
MCAO 24h v.s. SHAM	mmu_circRNA_22867	chr11	+	5596713	5635387	exonic	-3.31	7.68831	-5.17	0.00017103	0.001905	Ankrd36
MCAO 24h v.s. SHAM	mmu_circRNA_007681	chr9	-	21757116	21757390	sense overlapping	-2.027	11.7998	-4.11	0.00119555	0.007393	Spc24
MCAO 24h v.s. SHAM	mmu_circRNA_006229	chr12	-	79083615	79085856	exonic	2.179	10.5818	6.715	1.33E-05	0.000347	Pigh
MCAO 24h v.s. SHAM	mmu_circRNA_018772	chr16	-	46436353	46438068	exonic	2.153	12.9806	7.082	7.58E-06	0.00025	Nectin3

MCAO 24h v.s. SHAM	mmu_circRNA_26004	chr13	-	10224644	10228621	sense overlapping	2.839	10.0926	7.352	5.07E-06	0.000192	Chrm3
MCAO 24h v.s. SHAM	mmu_circRNA_32779	chr19	-	56542632	56545646	exonic	2.696	6.6536	6.256	2.74E-05	0.000559	Dclre1a
MCAO 24h v.s. SHAM	mmu_circRNA_45692	chrX	+	106097389	106102846	exonic	3.964	7.42572	9.246	3.90E-07	5.20E-05	Atp7a
MCAO 24h v.s. SHAM	mmu_circRNA_41537	chr7	-	61813261	61916736	exonic	2.115	10.3586	5.701	6.85E-05	0.001034	A230057D06Rik
MCAO 24h v.s. SHAM	mmu_circRNA_39349	chr5	-	123590792	123642616	exonic	3.076	8.93705	7.598	3.55E-06	0.000154	Clip1
MCAO 24h v.s. SHAM	mmu_circRNA_32634	chr19	+	43810470	43814814	exonic	4.044	6.89627	8.441	1.10E-06	8.16E-05	Abcc2
MCAO 24h v.s. SHAM	mmu_circRNA_22146	chr10	-	66176233	66205600	antisense	2.213	9.6147	5.809	5.71E-05	0.000913	Gm31056
MCAO 24h v.s. SHAM	mmu_circRNA_22146	chr10	-	66176233	66205600	antisense	2.213	9.6147	5.809	5.71E-05	0.000913	4930407I19Rik
MCAO 24h v.s. SHAM	mmu_circRNA_21730	chr10	+	20158760	20159555	intronic	4.229	12.6282	7.717	2.99E-06	0.000142	Map7
MCAO 24h v.s. SHAM	mmu_circRNA_37893	chr4	-	145016734	145100067	exonic	-2.465	9.1335	-7.99	2.03E-06	0.000114	Vps13d
MCAO 24h v.s. SHAM	mmu_circRNA_41211	chr7	+	19368541	19368859	exonic	2.767	7.1614	8.678	8.07E-07	7.43E-05	Ppp1r13l
MCAO 24h v.s. SHAM	mmu_circRNA_22909	chr11	-	12306957	12309752	exonic	3.173	8.74306	9.413	3.17E-07	5.12E-05	Cobl
MCAO 24h v.s. SHAM	mmu_circRNA_44431	chr9	-	64149398	64153650	exonic	-2.06	10.8032	-3.02	0.00968764	0.033961	Zwilch
MCAO 24h v.s. SHAM	mmu_circRNA_31562	chr18	-	34614447	34616734	exonic	-2.477	7.18692	-6.19	3.03E-05	0.000603	Brd8
MCAO 24h v.s. SHAM	mmu_circRNA_42611	chr8	-	16346570	16501392	exonic	2.247	8.41874	5.9	4.90E-05	0.000823	Csmd1
MCAO 24h v.s. SHAM	mmu_circRNA_21761	chr10	-	25239625	25289730	exonic	-2.445	9.91409	-6.61	1.55E-05	0.000389	Akap7
MCAO 24h v.s. SHAM	mmu_circRNA_36929	chr4	-	70298819	70380301	exonic	-2.207	8.69443	-5.12	0.00018779	0.002039	Cdk5rap2
MCAO 24h v.s. SHAM	mmu_circRNA_42246	chr7	+	121032747	121057399	sense overlapping	3.9	11.8786	7.31	5.40E-06	0.000198	NA
MCAO 24h v.s. SHAM	mmu_circRNA_44758	chr9	-	86372340	86448853	exonic	2.077	9.30834	5.112	0.00018983	0.002059	Ube2cbp
MCAO 24h v.s. SHAM	mmu_circRNA_018441	chr1	+	163992593	163992680	antisense	4.033	6.37665	10.04	1.49E-07	4.38E-05	BC055324

MCAO 24h v.s. SHAM	mmu_circRNA_001686	chr6	+	61310813	61313835	exonic	2.086	13.6931	6.258	2.73E-05	0.000559	Ccser1
MCAO 24h v.s. SHAM	mmu_circRNA_29705	chr16	-	38498860	38518532	sense overlapping	-2.143	8.77763	-4.4	0.00069306	0.005075	Timmdc1
MCAO 24h v.s. SHAM	mmu_circRNA_28306	chr15	+	23436449	23460567	exonic	3.174	7.69871	4.954	0.00025125	0.002501	Cdh18
MCAO 24h v.s. SHAM	mmu_circRNA_21755	chr10	-	23138190	23159154	exonic	2.374	13.2622	5.771	6.09E-05	0.000953	Eya4
MCAO 24h v.s. SHAM	mmu_circRNA_40808	chr6	-	117702621	117702723	exonic	2.851	6.967	8.982	5.45E-07	5.92E-05	1700030F04Rik
MCAO 24h v.s. SHAM	mmu_circRNA_011784	chr8	-	77450692	77450923	exonic	-2.43	12.2097	-5.69	6.94E-05	0.001041	Arhgap10
MCAO 24h v.s. SHAM	mmu_circRNA_19073	chr12	+	81894979	81913476	sense overlapping	5.2	8.00463	11.33	3.53E-08	3.37E-05	Pcnx
MCAO 24h v.s. SHAM	mmu_circRNA_45629	chrX	-	98695998	98714211	exonic	3.604	10.5693	7.258	5.83E-06	0.00021	Ophn1
MCAO 24h v.s. SHAM	mmu_circRNA_20164	chr1	-	59066149	59076722	exonic	3.835	8.62554	9.394	3.25E-07	5.13E-05	Als2cr11
MCAO 24h v.s. SHAM	mmu_circRNA_22031	chr10	-	56064473	56129177	exonic	3.348	6.07152	9.947	1.66E-07	4.38E-05	Tbc1d32
MCAO 24h v.s. SHAM	mmu_circRNA_29261	chr16	-	13603027	13648499	exonic	-2.703	10.275	-6.15	3.24E-05	0.000621	Parn
MCAO 24h v.s. SHAM	mmu_circRNA_24681	chr12	+	8238442	8275890	exonic	2.179	6.57156	4.26	0.00089759	0.006125	Ldah
MCAO 24h v.s. SHAM	mmu_circRNA_37285	chr4	+	103754180	103755895	intronic	2.651	9.65288	4.948	0.00025396	0.002523	Dab1
MCAO 24h v.s. SHAM	mmu_circRNA_20201	chr1	+	60181462	60206416	exonic	3.063	6.89202	7.588	3.60E-06	0.000156	Nbeal1
MCAO 24h v.s. SHAM	mmu_circRNA_23477	chr11	+	62283596	62285885	exonic	2.83	9.10737	4.33	0.00078851	0.005598	Ttc19
MCAO 24h v.s. SHAM	mmu_circRNA_22246	chr10	+	75966350	75986140	exonic	3.081	10.6644	5.927	4.69E-05	0.000802	Gm5134
MCAO 24h v.s. SHAM	mmu_circRNA_29893	chr16	-	63566578	63603633	exonic	2.266	6.88765	6.626	1.52E-05	0.000386	Epha3
MCAO 24h v.s. SHAM	mmu_circRNA_43344	chr8	-	94086655	94092622	exonic	-3.988	6.19788	-6.19	3.07E-05	0.000606	Bbs2
MCAO 24h v.s. SHAM	mmu_circRNA_27734	chr14	-	66187442	66190234	exonic	4.1	9.4989	11.36	3.42E-08	3.37E-05	Ptk2b
MCAO 24h v.s. SHAM	mmu_circRNA_23240	chr11	-	41912311	41920550	sense overlapping	-2.344	8.62501	-3.79	0.00218052	0.011416	Gabrg2
MCAO 24h v.s. SHAM	mmu_circRNA_23240	chr11	-	41912311	41920550	sense overlapping	-2.344	8.62501	-3.79	0.00218052	0.011416	LOC102637814

MCAO 24h v.s. SHAM	mmu_circRNA_37352	chr4	-	108250494	108252216	exonic	2.231	9.00138	5.536	9.07E-05	0.001268	Zyg11b
MCAO 24h v.s. SHAM	mmu_circRNA_30556	chr17	-	46882732	46883949	sense overlapping	4.223	9.65751	10.54	8.40E-08	3.91E-05	NA
MCAO 24h v.s. SHAM	mmu_circRNA_42042	chr7	+	104571920	104588022	sense overlapping	-2.85	11.4005	-5.27	0.00014417	0.001723	NA
MCAO 24h v.s. SHAM	mmu_circRNA_36399	chr4	+	3589146	3598663	exonic	3.045	10.4637	4.671	0.00041922	0.003539	Tgs1
MCAO 24h v.s. SHAM	mmu_circRNA_25951	chr13	+	8559103	8570574	exonic	4.249	9.2851	8.49	1.03E-06	7.91E-05	Adarb2
MCAO 24h v.s. SHAM	mmu_circRNA_015986	chr8	-	77344639	77365160	exonic	-2.269	10.3786	-5.76	6.24E-05	0.000965	Arhgap10
MCAO 24h v.s. SHAM	mmu_circRNA_19084	chr13	+	13725811	13761203	sense overlapping	-2.292	5.71198	-4.28	0.00085744	0.005953	Lyst
MCAO 24h v.s. SHAM	mmu_circRNA_33227	chr2	-	29247827	29248878	sense overlapping	4.138	11.6832	7.649	3.30E-06	0.00015	6530402F18Rik
MCAO 24h v.s. SHAM	mmu_circRNA_37527	chr4	+	120447032	120478090	exonic	-2.148	8.27686	-5.24	0.00015173	0.001766	Scmh1
MCAO 24h v.s. SHAM	mmu_circRNA_44903	chr9	-	99101174	99122376	exonic	-3.077	7.25337	-5.83	5.47E-05	0.000888	Pik3cb
MCAO 24h v.s. SHAM	mmu_circRNA_40693	chr6	+	103700404	103706636	exonic	-2.332	8.98752	-4.38	0.00071706	0.005203	Chl1
MCAO 24h v.s. SHAM	mmu_circRNA_36755	chr4	-	48218080	48243549	exonic	2.079	12.5819	5.787	5.92E-05	0.000932	Erp44
MCAO 24h v.s. SHAM	mmu_circRNA_43615	chr8	+	125124492	125135509	exonic	-2.418	6.13057	-5.35	0.00012457	0.001576	Disc1
MCAO 24h v.s. SHAM	mmu_circRNA_25129	chr12	-	55671300	55677265	intronic	4.213	7.77317	7.332	5.22E-06	0.000195	Ralgapa1
MCAO 24h v.s. SHAM	mmu_circRNA_21394	chr1	-	178333886	178336158	exonic	-2.621	6.90231	-7.09	7.46E-06	0.000248	Hnrnpu
MCAO 24h v.s. SHAM	mmu_circRNA_36669	chr4	+	41017971	41018489	exonic	-2.249	10.0884	-3.3	0.00560116	0.022768	Nfx1
MCAO 24h v.s. SHAM	mmu_circRNA_006502	chr11	-	104368679	104378826	exonic	-2.696	10.6323	-8.37	1.21E-06	8.51E-05	Kansl1
MCAO 24h v.s. SHAM	mmu_circRNA_37478	chr4	-	116611971	116622814	exonic	2.907	10.5718	9.299	3.65E-07	5.17E-05	Nasp
MCAO 24h v.s. SHAM	mmu_circRNA_007835	chr13	-	64243633	64248466	sense overlapping	2.449	7.86489	6.976	8.88E-06	0.000273	Cdc14b
MCAO 24h v.s. SHAM	mmu_circRNA_21193	chr1	-	164439298	164441641	exonic	2.18	7.36698	4.987	0.00023719	0.002405	Atp1b1

MCAO 24h v.s. SHAM	mmu_circRNA_35381	chr3	-	55786440	56009643	exonic	-2.674	9.13842	-3.24	0.00634602	0.024921	Nbea
MCAO 24h v.s. SHAM	mmu_circRNA_37994	chr4	+	154018369	154019032	exonic	2.015	9.62179	4.76	0.00035695	0.00318	Lrrc47
MCAO 24h v.s. SHAM	mmu_circRNA_26972	chr13	+	112669586	112714247	exonic	-2.136	8.61299	-4.18	0.00104356	0.006776	Slc38a9
MCAO 24h v.s. SHAM	mmu_circRNA_29131	chr16	-	4137365	4180133	exonic	-2.307	11.6915	-4.77	0.0003483	0.003124	Crebbp
MCAO 24h v.s. SHAM	mmu_circRNA_27500	chr14	-	40907705	40934285	sense overlapping	3.28	10.731	8.397	1.17E-06	8.34E-05	Tspan14
MCAO 24h v.s. SHAM	mmu_circRNA_31359	chr18	-	16627567	16629712	exonic	2.564	11.9233	5.93	4.66E-05	0.000801	Cdh2
MCAO 24h v.s. SHAM	mmu_circRNA_30327	chr17	-	24974704	24984025	exonic	2.285	13.1057	8.77	7.17E-07	6.91E-05	Cramp1l
MCAO 24h v.s. SHAM	mmu_circRNA_34491	chr2	-	126919638	126926999	exonic	-3.557	9.25167	-6.18	3.10E-05	0.000608	Sppl2a
MCAO 24h v.s. SHAM	mmu_circRNA_32568	chr19	+	38940470	38945573	exonic	-2.56	6.48579	-3.92	0.00172081	0.009645	Hells
MCAO 24h v.s. SHAM	mmu_circRNA_36304	chr3	-	152285201	152320743	exonic	2.17	7.32744	5.262	0.00014561	0.001731	Miga1
MCAO 24h v.s. SHAM	mmu_circRNA_002959	chr8	+	85875633	85922242	exonic	-2.229	7.28717	-4.71	0.00038796	0.003341	Phkb
MCAO 24h v.s. SHAM	mmu_circRNA_21287	chr1	+	173348412	173394033	sense overlapping	-2.145	8.22131	-4.44	0.0006433	0.004799	Aim2
MCAO 24h v.s. SHAM	mmu_circRNA_43349	chr8	-	94828276	94829230	exonic	2.425	6.95634	4.081	0.0012574	0.007668	Ciapin1
MCAO 24h v.s. SHAM	mmu_circRNA_010792	chr5	+	33843111	33868878	exonic	2.191	8.71653	5.252	0.00014829	0.001742	Nsd2
MCAO 24h v.s. SHAM	mmu_circRNA_35899	chr3	+	101425450	101427440	exonic	-2.197	6.55771	-4.99	0.00023395	0.002375	Igsf3
MCAO 24h v.s. SHAM	mmu_circRNA_22174	chr10	+	69607154	69608960	intronic	2.874	10.003	10.28	1.12E-07	4.18E-05	Ank3
MCAO 24h v.s. SHAM	mmu_circRNA_31510	chr18	+	32419842	32432425	exonic	2.202	7.71473	5.211	0.00015935	0.00183	Bin1
MCAO 24h v.s. SHAM	mmu_circRNA_24243	chr11	+	104456946	104459039	antisense	2.562	11.0241	6.21	2.95E-05	0.00059	NA
MCAO 24h v.s. SHAM	mmu_circRNA_44176	chr9	+	53552249	53566832	exonic	2.165	10.6458	7.48	4.21E-06	0.00017	Npat
MCAO 24h v.s. SHAM	mmu_circRNA_26219	chr13	-	38934737	38944654	exonic	2.328	11.0949	4.619	0.00046129	0.003781	Slc35b3
MCAO 24h v.s. SHAM	mmu_circRNA_21246	chr1	+	167454466	167455198	intronic	-2.451	10.4473	-3.83	0.00201975	0.01081	Gm34207

MCAO 24h v.s. SHAM	mmu_circRNA_21246	chr1	+	167454466	167455198	intronic	-2.451	10.4473	-3.83	0.00201975	0.01081	Lrrc52
MCAO 24h v.s. SHAM	mmu_circRNA_26502	chr13	-	64104732	64113001	exonic	3.603	9.62939	9.194	4.17E-07	5.26E-05	Slc35d2
MCAO 24h v.s. SHAM	mmu_circRNA_40444	chr6	+	80947553	80953743	intergenic	4.136	5.72681	9.853	1.86E-07	4.47E-05	NA
MCAO 24h v.s. SHAM	mmu_circRNA_010803	chr6	-	115634552	115644642	exonic	-4.725	11.5318	-8.54	9.70E-07	7.77E-05	Raf1
MCAO 24h v.s. SHAM	mmu_circRNA_40795	chr6	-	116133766	116138250	exonic	-4.765	12.4267	-6.49	1.88E-05	0.000437	Tmcc1
MCAO 24h v.s. SHAM	mmu_circRNA_26526	chr13	-	68668469	68678615	exonic	3.715	12.1565	8.102	1.75E-06	0.000103	Adcy2
MCAO 24h v.s. SHAM	mmu_circRNA_35512	chr3	+	65669089	65784025	exonic	2.664	7.97222	4.058	0.00131314	0.007896	Lekr1
MCAO 24h v.s. SHAM	mmu_circRNA_20627	chr1	+	105719451	105727988	exonic	2.329	7.92264	5.718	6.66E-05	0.001014	2310035C23Rik
MCAO 24h v.s. SHAM	mmu_circRNA_41969	chr7	-	98610666	98637520	exonic	3.581	9.96169	6.969	8.98E-06	0.000273	Emsy
MCAO 24h v.s. SHAM	mmu_circRNA_34628	chr2	+	135805548	135910244	exonic	-2.026	5.98454	-6.53	1.78E-05	0.000423	Plcb4
MCAO 24h v.s. SHAM	mmu_circRNA_35656	chr3	+	83346159	83351824	intergenic	2.063	10.7672	7.558	3.76E-06	0.00016	NA
MCAO 24h v.s. SHAM	mmu_circRNA_32900	chr2	-	5914809	5923023	sense overlapping	4.465	7.94835	8.162	1.61E-06	9.72E-05	Dhtkd1
MCAO 24h v.s. SHAM	mmu_circRNA_32430	chr19	-	30781294	30993162	exonic	-2.79	6.66892	-7.71	3.04E-06	0.000142	Prkg1
MCAO 24h v.s. SHAM	mmu_circRNA_19057	chr12	+	8698087	8713939	sense overlapping	-2.561	9.4294	-5.77	6.05E-05	0.000951	Pum2
MCAO 24h v.s. SHAM	mmu_circRNA_33228	chr2	+	29679132	29689263	exonic	4.447	9.78871	6.941	9.38E-06	0.000279	Rapgef1
MCAO 24h v.s. SHAM	mmu_circRNA_39663	chr5	+	145211745	145218883	exonic	2.597	10.7123	7.131	7.04E-06	0.000242	Zkscan5
MCAO 24h v.s. SHAM	mmu_circRNA_44206	chr9	-	54423229	54447065	exonic	2.026	5.99297	7.958	2.14E-06	0.000117	Dmxl2
MCAO 24h v.s. SHAM	mmu_circRNA_008226	chr18	+	22375132	22392852	exonic	2.943	10.9977	5.862	5.23E-05	0.000863	Asxl3
MCAO 24h v.s. SHAM	mmu_circRNA_34199	chr2	+	110386528	110395382	intergenic	2.57	10.1918	8.591	9.05E-07	7.59E-05	NA
MCAO 24h v.s. SHAM	mmu_circRNA_28801	chr15	+	78044129	78047183	antisense	2.105	7.00582	6.17	3.15E-05	0.000613	LOC108168245

MCAO 24h v.s. SHAM	mmu_circRNA_28801	chr15	+	78044129	78047183	antisense	2.105	7.00582	6.17	3.15E-05	0.000613	Cacng2
MCAO 24h v.s. SHAM	mmu_circRNA_27766	chr14	-	70612667	70613420	exonic	-2.032	7.73081	-7.55	3.81E-06	0.000161	Dmtn
MCAO 24h v.s. SHAM	mmu_circRNA_33006	chr2	+	15837315	15871582	exonic	2.409	10.2742	6.623	1.53E-05	0.000387	Malrd1
MCAO 24h v.s. SHAM	mmu_circRNA_28919	chr15	+	89018149	89026636	exonic	3.324	8.81054	11.2	4.07E-08	3.37E-05	Mov10l1
MCAO 24h v.s. SHAM	mmu_circRNA_25584	chr12	+	88431020	88459203	exonic	2.951	12.9812	5.345	0.00012603	0.001585	Adck1
MCAO 24h v.s. SHAM	mmu_circRNA_40506	chr6	-	85256342	85289788	exonic	3.182	9.84178	9.386	3.28E-07	5.13E-05	Sfxn5
MCAO 24h v.s. SHAM	mmu_circRNA_011505	chr15	+	79114654	79115171	exonic	4.285	10.6939	6.473	1.94E-05	0.000444	Micall1
MCAO 24h v.s. SHAM	mmu_circRNA_20272	chr1	+	65356127	65384001	sense overlapping	2.842	7.50971	6.335	2.41E-05	0.000513	Pth2r
MCAO 24h v.s. SHAM	mmu_circRNA_27942	chr14	+	99686558	99687284	sense overlapping	2.102	6.88632	7.486	4.17E-06	0.00017	Gm34061
MCAO 24h v.s. SHAM	mmu_circRNA_31691	chr18	-	42645431	42645533	intronic	2.36	9.20963	3.809	0.00211249	0.011179	Ppp2r2b
MCAO 24h v.s. SHAM	mmu_circRNA_003739	chr14	-	121592393	121597756	sense overlapping	2.945	6.95357	8.912	5.96E-07	6.22E-05	Dock9
MCAO 24h v.s. SHAM	mmu_circRNA_26971	chr13	-	112604486	112622307	exonic	2.813	10.3028	7.708	3.03E-06	0.000142	Ddx4
MCAO 24h v.s. SHAM	mmu_circRNA_24679	chr12	-	8068097	8083726	intergenic	3.675	8.12083	9.992	1.58E-07	4.38E-05	Gm33037
MCAO 24h v.s. SHAM	mmu_circRNA_004736	chr15	-	38498668	38498768	sense overlapping	-2.054	11.6549	-6.11	3.49E-05	0.000657	Azin1
MCAO 24h v.s. SHAM	mmu_circRNA_33824	chr2	-	70786876	70795010	intronic	-2.222	11.7243	-4.82	0.00032051	0.002952	Tlk1
MCAO 24h v.s. SHAM	mmu_circRNA_19995	chr1	-	42660742	42667003	sense overlapping	2.724	7.71634	8.047	1.89E-06	0.000108	Pantr1
MCAO 24h v.s. SHAM	mmu_circRNA_42102	chr7	+	111046762	111050505	exonic	2.861	8.4655	7.836	2.53E-06	0.000131	Ctr9
MCAO 24h v.s. SHAM	mmu_circRNA_33184	chr2	-	25937945	25941469	exonic	5.079	8.60169	6.993	8.66E-06	0.000269	Camsap1
MCAO 24h v.s. SHAM	mmu_circRNA_41957	chr7	-	98230449	98259457	exonic	2.182	9.34598	4.334	0.00078274	0.005569	Acer3
MCAO 24h v.s. SHAM	mmu_circRNA_42622	chr8	+	18629545	18641665	exonic	-2.042	8.50076	-5.48	0.00010017	0.001357	Mcph1
MCAO 24h v.s. SHAM	mmu_circRNA_38427	chr5	+	33821658	33868878	exonic	2.521	8.28324	5.752	6.28E-05	0.000971	Nsd2

MCAO 24h v.s. SHAM	mmu_circRNA_29190	chr16	-	8700045	8715919	exonic	-2.197	7.1865	-4.73	0.00037664	0.003279	Usp7
MCAO 24h v.s. SHAM	mmu_circRNA_40766	chr6	-	113522313	113522807	exonic	2.265	8.05894	5.87	5.16E-05	0.000853	Emc3
MCAO 24h v.s. SHAM	mmu_circRNA_19041	chr11	-	103386956	103387349	intronic	2.675	11.6018	7.735	2.92E-06	0.000141	Plekhm1
MCAO 24h v.s. SHAM	mmu_circRNA_013131	chr4	+	24505344	24536448	exonic	2.587	8.6949	6.646	1.48E-05	0.000379	Mms22l
MCAO 24h v.s. SHAM	mmu_circRNA_009923	chr5	+	132226987	132227061	antisense	4.209	6.97919	6.295	2.57E-05	0.000538	Auts2
MCAO 24h v.s. SHAM	mmu_circRNA_20394	chr1	-	80283675	80304193	exonic	-2.158	6.96447	-5.22	0.00015691	0.001812	Cul3
MCAO 24h v.s. SHAM	mmu_circRNA_19372	chr5	-	110692047	110692115	intronic	2.397	9.74876	6.423	2.09E-05	0.000465	Ep400
MCAO 24h v.s. SHAM	mmu_circRNA_41210	chr7	+	19186101	19186371	exonic	-2.953	12.8742	-5.23	0.00015283	0.001775	Eml2
MCAO 24h v.s. SHAM	mmu_circRNA_43328	chr8	-	93104118	93108573	exonic	2.604	9.00022	8.073	1.82E-06	0.000106	Ces1c
MCAO 24h v.s. SHAM	mmu_circRNA_40341	chr6	-	60815616	60818365	exonic	-2.615	10.4939	-5.76	6.18E-05	0.000961	Snca
MCAO 24h v.s. SHAM	mmu_circRNA_37932	chr4	-	149251740	149267283	exonic	2.575	6.65868	6.729	1.30E-05	0.000344	Kif1b
MCAO 24h v.s. SHAM	mmu_circRNA_40441	chr6	+	80231810	80232745	intronic	2.001	8.76116	5.904	4.87E-05	0.000821	Lrrtm4
MCAO 24h v.s. SHAM	mmu_circRNA_19339	chr4	+	130727992	130730549	intronic	-2.384	10.5404	-5.08	0.0002014	0.002152	Pum1
MCAO 24h v.s. SHAM	mmu_circRNA_22461	chr10	-	92902014	92927839	exonic	2.005	6.25072	6.969	8.98E-06	0.000273	Cfap54

Supplementary Table S4. Parenral gene enrichment pathway analysis by GeneCoDIS3.

Groups	Pathway ID	Name	Combined Hypgeometric	Gene list
MCAO 5 min	Panther:P00047	PDGF signaling pathway	0.000206694	94190,57257,56637,110157,78514, 23871
MCAO 5 min	Kegg:04910,Panther:P00026	Insulin signaling pathway,Heterotrimeric G-protein signaling pathway-Gi alpha and Gs alpha mediated pathway	0.000548558	56637,110094,102093
MCAO 5 min	Panther:P00047,Panther:P00005,Panther:P04393	PDGF signaling pathway,Angiogenesis,Ras Pathway	0.000550474	56637,110157,23871
MCAO 5 min	Kegg:04062,Kegg:04510,Panther:P00047,Kegg:04660,Kegg:04662	Chemokine signaling pathway,Focal adhesion,PDGF signaling pathway,T cell receptor signaling pathway,B cell receptor signaling pathway	0.00062609	57257,56637,110157
MCAO 5 min	Kegg:04660	T cell receptor signaling pathway	0.000683869	21682,57257,56637,110157,228026
MCAO 5 min	Kegg:04062,Kegg:04916	Chemokine signaling pathway,Melanogenesis	0.00102507	224129,56637,110157
MCAO 5 min	Kegg:05160,Kegg:04722,Kegg:04660	Hepatitis C,Neurotrophin signaling pathway,T cell receptor signaling pathway	0.00104372	56637,110157,228026
MCAO 5 min	Kegg:04910	Insulin signaling pathway	0.00111268	56637,110094,110157,102093,22084
MCAO 5 min	Kegg:04660,Kegg:04664	T cell receptor signaling pathway,Fc epsilon RI signaling pathway	0.00197523	57257,110157,228026
MCAO 5 min	Panther:P00026	Heterotrimeric G-protein signaling pathway-Gi alpha and Gs alpha mediated pathway	0.00635568	224129,56637,110094,102093
MCAO 5 min	Kegg:04062	Chemokine signaling pathway	0.0130995	57257,224129,56637,110157
MCAO 5 min	Kegg:04020	Calcium signaling pathway	0.0130995	110094,13867,102093,20191
MCAO 5 min	Kegg:05414	Dilated cardiomyopathy	0.0138963	224129,20191,12296
MCAO 5 min	Kegg:04012	ErbB signaling pathway	0.0142372	56637,13867,110157
MCAO 5 min	Kegg:04972	Pancreatic secretion	0.0152902	224129,53869,20191
MCAO 5 min	Panther:P00034	Integrin signalling pathway	0.0287739	19248,110157,78514
MCAO 3 h	Kegg:04724	Glutamatergic synapse	0.000336807	216456,12288,14702,11514,110355,210044,105727,14660
MCAO 3 h	Kegg:04020,Kegg:04912	Calcium signaling pathway,GnRH signaling pathway	0.00144652	12288,11514,210044,19229

MCAO 3 h	Panther:P00026,Kegg:04724,Kegg:04062,Panther:P00031	Heterotrimeric G-protein signaling pathway-Gi alpha and Gs alpha mediated pathway,Glutamatergic synapse,Chemokine signaling pathway,Inflammation mediated by chemokine and cytokine signaling pathway	0.0015781	14702,110355,210044
MCAO 3 h	Kegg:04724,Kegg:05414,Kegg:04020,Kegg:04270,Kegg:04912	Glutamatergic synapse,Dilated cardiomyopathy,Calcium signaling pathway,Vascular smooth muscle contraction,GnRH signaling pathway	0.0015781	12288,11514,210044
MCAO 3 h	Kegg:04062,Kegg:04020,Kegg:04912	Chemokine signaling pathway,Calcium signaling pathway,GnRH signaling pathway	0.00181815	11514,210044,19229
MCAO 3 h	Kegg:04724,Kegg:04062	Glutamatergic synapse,Chemokine signaling pathway	0.0018236	14702,11514,110355,210044
MCAO 3 h	Kegg:04724,Panther:P04377,Panther:P04378	Glutamatergic synapse,Beta1 adrenergic receptor signaling pathway,Beta2 adrenergic receptor signaling pathway	0.00267155	12288,14702,210044
MCAO 3 h	Kegg:04972,Kegg:04970,Kegg:04270	Pancreatic secretion,Salivary secretion,Vascular smooth muscle contraction	0.00267155	16531,11514,210044
MCAO 3 h	Kegg:04062	Chemokine signaling pathway	0.00319366	14702,21844,11514,110355,210044,19229,277360
MCAO 3 h	Kegg:04020	Calcium signaling pathway	0.011213	12288,58226,110094,11514,210044,19229
MCAO 3 h	Kegg:04062,Panther:P00031	Chemokine signaling pathway,Inflammation mediated by chemokine and cytokine signaling pathway	0.0114385	14702,110355,210044,277360
MCAO 3 h	Kegg:04910	Insulin signaling pathway	0.0126031	19017,15277,110094,107746,15275
MCAO 3 h	Kegg:00500	Starch and sucrose metabolism	0.0144994	100727,15277,15275
MCAO 3 h	Kegg:05414	Dilated cardiomyopathy	0.0154781	12288,11514,210044,54376
MCAO 3 h	Kegg:04930	Type II diabetes mellitus	0.0161115	12288,15277,15275
MCAO 3 h	Kegg:04270	Vascular smooth muscle contraction	0.0268361	12288,16531,11514,210044
MCAO 3 h	Panther:P00031	Inflammation mediated by chemokine and cytokine signaling pathway	0.0379845	14702,110355,210044,18037,277360
MCAO 3 h	Panther:P00026	Heterotrimeric G-protein signaling pathway-Gi alpha and Gs alpha mediated pathway	0.0380963	14702,110094,110355,210044
MCAO 3 h	Kegg:05412	Arrhythmogenic right ventricular cardiomyopathy (ARVC)	0.0381573	12288,12558,54376
MCAO 3 h	Kegg:04976	Bile secretion	0.0383239	11514,210044,12780

MCAO 24 h	Kegg:04724,Kegg:05010	Glutamatergic synapse,Alzheimer's disease	0.0011369	12288,16438,12289,14682,18798,14812,14811
MCAO 24 h	Kegg:04020,Kegg:04912	Calcium signaling pathway,GnRH signaling pathway	0.0012582	12288,11515,16438,12289,210044,14682,19229,18798
MCAO 24 h	Kegg:04724,Kegg:05010,Kegg:04020,Kegg:04270,Kegg:04912	Glutamatergic synapse,Alzheimer's disease,Calcium signaling pathway,Vascular smooth muscle contraction,GnRH signaling pathway	0.00128962	12288,16438,12289,14682,18798
MCAO 24 h	Kegg:04972,Kegg:04971	Pancreatic secretion,Gastric acid secretion	0.00131696	11515,16438,16535,210044,14682,11931,12671,18798
MCAO 24 h	Kegg:05016,Kegg:04720	Huntington's disease,Long-term potentiation	0.0016364	12914,16438,14682,18798,14812
MCAO 24 h	Kegg:04724,Kegg:04020	Glutamatergic synapse,Calcium signaling pathway	0.00165541	12288,11515,16438,12289,210044,14682,18798,14811
MCAO 24 h	Kegg:04724,Kegg:04020,Kegg:04270,Kegg:04912	Glutamatergic synapse,Calcium signaling pathway,Vascular smooth muscle contraction,GnRH signaling pathway	0.00166159	12288,11515,16438,12289,210044,14682,18798
MCAO 24 h	Kegg:05010,Kegg:04020	Alzheimer's disease,Calcium signaling pathway	0.0017073	12288,16438,12289,14682,18798,20192,14811
MCAO 24 h	Kegg:04010,Kegg:04260,Kegg:05410,Kegg:05412,Kegg:05414	MAPK signaling pathway,Cardiac muscle contraction,Hypertrophic cardiomyopathy (HCM),Arrhythmogenic right ventricular cardiomyopathy (ARVC),Dilated cardiomyopathy	0.00177136	12288,12300,12289,54376,12296,12293
MCAO 24 h	Kegg:04972,Kegg:04970,Kegg:04020,Kegg:04971	Pancreatic secretion,Salivary secretion,Calcium signaling pathway,Gastric acid secretion	0.00177136	11515,16438,210044,14682,12671,18798
MCAO 24 h	Kegg:04724,Kegg:05010,Kegg:04020	Glutamatergic synapse,Alzheimer's disease,Calcium signaling pathway	0.00180401	12288,16438,12289,14682,18798,14811
MCAO 24 h	Panther:P00031,Panther:P00005,Kegg:04664,Kegg:04666,Panther:P00018,Panther:P00021	Inflammation mediated by chemokine and cytokine signaling pathway,Angiogenesis,Fc epsilon RI signaling pathway,Fc gamma R-mediated phagocytosis,EGF receptor signaling pathway,FGF signaling pathway	0.00180676	23797,234779,74769,110157,18754
MCAO 24 h	Kegg:04910,Kegg:05160,Kegg:04722,Kegg:05200,Kegg:04062,Kegg:04510,Kegg:05215,Panther:P00005,Panther:P00036,Kegg:05210,Kegg:05213,Kegg:04012,Kegg:04660,Panther:P04393,Kegg:04662	Insulin signaling pathway,Hepatitis C,Neurotrophin signaling pathway,Pathways in cancer,Chemokine signaling pathway,Focal adhesion,Prostate cancer,Angiogenesis,Interleukin signaling pathway,Colorectal cancer,Endometrial cancer,ErbB signaling pathway,T cell receptor signaling pathway,Ras Pathway,B cell receptor signaling pathway	0.00184384	23797,74769,56637,110157

MCAO 24 h	Kegg:04724,Kegg:05010,Kegg:05016,Kegg:04720	Glutamatergic synapse,Alzheimer's disease,Huntington's disease,Long-term potentiation	0.00184384	16438,14682,18798,14812
MCAO 24 h	Kegg:04972,Kegg:04970	Pancreatic secretion,Salivary secretion	0.00189677	11515,16438,16531,210044,14682,11931,12671,18798
MCAO 24 h	Kegg:04972,Kegg:04970,Kegg:04971	Pancreatic secretion,Salivary secretion,Gastric acid secretion	0.00191593	11515,16438,210044,14682,11931,12671,18798
MCAO 24 h	Kegg:04972,Kegg:04970,Kegg:04270	Pancreatic secretion,Salivary secretion,Vascular smooth muscle contraction	0.00192478	11515,16438,16531,210044,14682,18798
MCAO 24 h	Kegg:04724,Kegg:05010,Kegg:04720	Glutamatergic synapse,Alzheimer's disease,Long-term potentiation	0.00199415	12288,16438,14682,18798,14812,14811
MCAO 24 h	Kegg:04724	Glutamatergic synapse	0.00221888	108069,216456,12288,14702,11515,16438,12289,110355,210044,14682,105727,18798,14812,14806,14811,14660
MCAO 24 h	Kegg:04020	Calcium signaling pathway	0.002306	12288,234779,11515,16438,12289,58226,110094,13867,102093,210044,14682,12671,19229,18798,20192,18438,14811
MCAO 24 h	Kegg:04010,Kegg:04260,Kegg:05410,Kegg:05412,Kegg:05414,Panther:P04377,Panther:P04391,Panther:P04378,Panther:P00044,Panther:P04374,Panther:P00003	MAPK signaling pathway,Cardiac muscle contraction,Hypertrophic cardiomyopathy (HCM),Arrhythmogenic right ventricular cardiomyopathy (ARVC),Dilated cardiomyopathy,Beta1 adrenergic receptor signaling pathway,Oxytocin receptor mediated signaling pathway,Beta2 adrenergic receptor signaling pathway,Nicotinic acetylcholine receptor signaling pathway,5HT2 type receptor mediated signaling pathway,Alzheimer disease-amyloid secretase pathway	0.00239246	12288,12289,12296
MCAO 24 h	Kegg:04930,Panther:P04391,Panther:P04374,Kegg:04270,Panther:P00003	Type II diabetes mellitus,Oxytocin receptor mediated signaling pathway,5HT2 type receptor mediated signaling pathway,Vascular smooth muscle contraction,Alzheimer disease-amyloid secretase pathway	0.00239246	12288,12289,18754
MCAO 24 h	Kegg:04540,Kegg:04270	Gap junction,Vascular smooth muscle contraction	0.00251125	11515,16438,110157,210044,14682,19091,18798
MCAO 24 h	Kegg:04970,Kegg:04270	Salivary secretion,Vascular smooth muscle contraction	0.00251125	11515,16438,16531,210044,14682,19091,18798
MCAO 24 h	Kegg:04724,Kegg:05010,Kegg:04020,Kegg:04270,Kegg:04720,Kegg:04912	Glutamatergic synapse,Alzheimer's disease,Calcium signaling pathway,Vascular smooth muscle contraction,Long-term potentiation,GnRH signaling pathway	0.00261081	12288,16438,14682,18798

MCAO 24 h	Kegg:04970	Salivary secretion	0.00274413	11515,16438,16531,210044,14682,11931,12671,19091,18798,20192
MCAO 24 h	Kegg:04062,Kegg:04510,Kegg:04660	Chemokine signaling pathway,Focal adhesion,T cell receptor signaling pathway	0.00292113	23797,18479,74769,57257,56637,110157
MCAO 24 h	Kegg:04722,Kegg:05200,Panther:P00031,Kegg:05214,Panther:P00005,Kegg:04012,Kegg:04370,Kegg:04662,Kegg:04664,Kegg:04666,Panther:P00018,Panther:P00021,Kegg:05223	Neurotrophin signaling pathway,Pathways in cancer,Inflammation mediated by chemokine and cytokine signaling pathway,Glioma,Angiogenesis,ErbB signaling pathway,VEGF signaling pathway,B cell receptor signaling pathway,Fc epsilon RI signaling pathway,Fc gamma R-mediated phagocytosis,EGF receptor signaling pathway,FGF signaling pathway,Non-small cell lung cancer	0.0031714	23797,234779,74769,110157
MCAO 24 h	Panther:P00019,Panther:P00031	Endothelin signaling pathway,Inflammation mediated by chemokine and cytokine signaling pathway	0.00339202	23797,74769,16438,110157,210044,18754
MCAO 24 h	Kegg:04724,Kegg:04540,Kegg:04972,Kegg:04970,Kegg:04020,Kegg:04270,Kegg:04971,Kegg:04912	Glutamatergic synapse,Gap junction,Pancreatic secretion,Salivary secretion,Calcium signaling pathway,Vascular smooth muscle contraction,Gastric acid secretion,GnRH signaling pathway	0.00345573	11515,16438,210044,14682,18798
MCAO 24 h	Kegg:04722,Kegg:05200,Panther:P00005,Kegg:04012,Kegg:04662	Neurotrophin signaling pathway,Pathways in cancer,Angiogenesis,ErbB signaling pathway,B cell receptor signaling pathway	0.00359443	23797,234779,74769,56637,110157
MCAO 24 h	Kegg:04270,Kegg:04912	Vascular smooth muscle contraction,GnRH signaling pathway	0.00362859	12288,11515,16438,12289,110157,210044,14682,18798
MCAO 24 h	Panther:P00019,Panther:P00031,Panther:P00005,Kegg:04664,Kegg:04666,Panther:P00018,Panther:P00021	Endothelin signaling pathway,Inflammation mediated by chemokine and cytokine signaling pathway,Angiogenesis,Fc epsilon RI signaling pathway,Fc gamma R-mediated phagocytosis,EGF receptor signaling pathway,FGF signaling pathway	0.00366507	23797,74769,110157,18754
MCAO 24 h	Kegg:05414,Panther:P04377,Panther:P04378	Dilated cardiomyopathy,Beta1 adrenergic receptor signaling pathway,Beta2 adrenergic receptor signaling pathway	0.00366507	12288,12289,210044,12296
MCAO 24 h	Panther:P04391,Panther:P04374,Panther:P00003	Oxytocin receptor mediated signaling pathway,5HT2 type receptor mediated signaling pathway,Alzheimer disease-amyloid secretase pathway	0.00366507	12288,12289,18754,12296
MCAO 24 h	Panther:P00019	Endothelin signaling pathway	0.00367356	23797,11515,74769,16438,110157,210044,14682,18754,56529
MCAO 24 h	Kegg:04540,Kegg:04970,Kegg:04270	Gap junction,Salivary secretion,Vascular smooth muscle contraction	0.00371384	11515,16438,210044,14682,19091,18798

MCAO 24 h	Kegg:04540,Kegg:04270,Kegg:04912	Gap junction,Vascular smooth muscle contraction,GnRH signaling pathway	0.00371384	11515,16438,110157,210044,14682,18798
MCAO 24 h	Kegg:04970,Kegg:04020	Salivary secretion,Calcium signaling pathway	0.00384499	11515,16438,210044,14682,12671,18798,20192
MCAO 24 h	Kegg:04910,Kegg:05160,Kegg:04722,Kegg:05200,Kegg:04062,Kegg:05162,Kegg:04510,Kegg:05215,Panther:P00005,Panther:P00036,Kegg:05210,Kegg:05213,Kegg:04012,Kegg:04660,Panther:P04393,Kegg:04662,Panther:P00048	Insulin signaling pathway,Hepatitis C,Neurotrophin signaling pathway,Pathways in cancer,Chemokine signaling pathway,Measles,Focal adhesion,Prostate cancer,Angiogenesis,Interleukin signaling pathway,Colorectal cancer,Endometrial cancer,ErbB signaling pathway,T cell receptor signaling pathway,Ras Pathway,B cell receptor signaling pathway,PI3 kinase pathway	0.0039384	23797,74769,56637
MCAO 24 h	Panther:P00019,Panther:P00031,Panther:P00053	Endothelin signaling pathway,Inflammation mediated by chemokine and cytokine signaling pathway,T cell activation	0.00395368	23797,74769,16438,110157
MCAO 24 h	Panther:P00031,Panther:P00005,Panther:P00056,Kegg:04664,Kegg:04666,Panther:P00018,Panther:P00021	Inflammation mediated by chemokine and cytokine signaling pathway,Angiogenesis,VEGF signaling pathway,Fc epsilon RI signaling pathway,Fc gamma R-mediated phagocytosis,EGF receptor signaling pathway,FGF signaling pathway	0.00395368	234779,74769,110157,18754
MCAO 24 h	Panther:P00047,Kegg:04650,Kegg:04662,Kegg:04664,Kegg:04666,Panther:P00010	PDGF signaling pathway,Natural killer cell mediated cytotoxicity,B cell receptor signaling pathway,Fc epsilon RI signaling pathway,Fc gamma R-mediated phagocytosis,B cell activation	0.00395368	234779,74769,57257,110157
MCAO 24 h	Kegg:05010,Kegg:04970,Kegg:04020	Alzheimer's disease,Salivary secretion,Calcium signaling pathway	0.00395368	16438,14682,18798,20192
MCAO 24 h	Panther:P04391,Panther:P04374,Kegg:04270	Oxytocin receptor mediated signaling pathway,5HT2 type receptor mediated signaling pathway,Vascular smooth muscle contraction	0.00395368	12288,12289,14682,18754
MCAO 24 h	Kegg:04020,Panther:P00042	Calcium signaling pathway,Muscarinic acetylcholine receptor 1 and 3 signaling pathway	0.00395368	16438,14682,12671,14811
MCAO 24 h	Panther:P00019,Kegg:04270	Endothelin signaling pathway,Vascular smooth muscle contraction	0.00409416	11515,16438,110157,210044,14682,18754
MCAO 24 h	Kegg:04724,Kegg:05010,Kegg:04020,Kegg:04720	Glutamatergic synapse,Alzheimer's disease,Calcium signaling pathway,Long-term potentiation	0.00411172	12288,16438,14682,18798,14811
MCAO 24 h	Kegg:04722,Panther:P00031,Kegg:04662	Neurotrophin signaling pathway,Inflammation mediated by chemokine and cytokine signaling pathway,B cell receptor signaling pathway	0.00411172	23797,234779,74769,110157,18037

MCAO 24 h	Kegg:04062,Kegg:04510,Kegg:04660,Kegg:04666	Chemokine signaling pathway,Focal adhesion,T cell receptor signaling pathway,Fc gamma R-mediated phagocytosis	0.00411172	23797,18479,74769,57257,110157
MCAO 24 h	Kegg:04062,Kegg:04510,Kegg:04012,Kegg:04660	Chemokine signaling pathway,Focal adhesion,ErbB signaling pathway,T cell receptor signaling pathway	0.0043325	23797,18479,74769,56637,110157
MCAO 24 h	Kegg:04910,Kegg:05160,Kegg:04722,Kegg:05200,Kegg:04062,Kegg:04510,Kegg:05215,Panther:P00047,Panther:P00005,Panther:P00036,Kegg:05210,Kegg:05213,Kegg:04012,Kegg:04660,Panther:P04393,Kegg:04662	Insulin signaling pathway,Hepatitis C,Neurotrophin signaling pathway,Pathways in cancer,Chemokine signaling pathway,Focal adhesion,Prostate cancer,PDGF signaling pathway,Angiogenesis,Interleukin signaling pathway,Colorectal cancer,Endometrial cancer,ErbB signaling pathway,T cell receptor signaling pathway,Ras Pathway,B cell receptor signaling pathway	0.00434496	74769,56637,110157
MCAO 24 h	Panther:P00041,Kegg:04724,Kegg:05010,Kegg:04020,Kegg:04720,Panther:P00042	Metabotropic glutamate receptor group I pathway,Glutamatergic synapse,Alzheimer's disease,Calcium signaling pathway,Long-term potentiation,Muscarinic acetylcholine receptor 1 and 3 signaling pathway	0.00434496	16438,14682,14811
MCAO 24 h	Panther:P00025,Kegg:05200	Hedgehog signaling pathway,Pathways in cancer	0.00434496	12914,56637,14634
MCAO 24 h	Kegg:04724,Kegg:04540,Kegg:05010,Kegg:04972,Kegg:05016,Kegg:04970,Kegg:04020,Kegg:04270,Kegg:04971,Kegg:04720,Kegg:04730,Kegg:04912	Glutamatergic synapse,Gap junction,Alzheimer's disease,Pancreatic secretion,Huntington's disease,Salivary secretion,Calcium signaling pathway,Vascular smooth muscle contraction,Gastric acid secretion,Long-term potentiation,Long-term depression,GnRH signaling pathway	0.00434496	16438,14682,18798
MCAO 24 h	Kegg:04724,Kegg:05010,Panther:P04391,Panther:P04374,Kegg:04020,Kegg:04270,Kegg:04912	Glutamatergic synapse,Alzheimer's disease,Oxytocin receptor mediated signaling pathway,5HT2 type receptor mediated signaling pathway,Calcium signaling pathway,Vascular smooth muscle contraction,GnRH signaling pathway	0.00434496	12288,12289,14682
MCAO 24 h	Panther:P00027,Kegg:04972,Kegg:04970,Kegg:04020,Kegg:04971,Panther:P00042	Heterotrimeric G-protein signaling pathway-Gq alpha and Go alpha mediated pathway,Pancreatic secretion,Salivary secretion,Calcium signaling pathway,Gastric acid secretion,Muscarinic acetylcholine receptor 1 and 3 signaling pathway	0.00434496	16438,14682,12671
MCAO 24 h	Kegg:05016,Kegg:04720,Panther:P00057	Huntington's disease,Long-term potentiation,Wnt signaling pathway	0.00434496	12914,16438,14682
MCAO 24 h	Kegg:04910	Insulin signaling pathway	0.00439515	19017,23797,15277,74769,56637,110094,110157,102093,107746,108099,208650,22084,15275

MCAO 24 h	Kegg:04910,Kegg:05200,Kegg:05162,Kegg:04012,Kegg:04660	Insulin signaling pathway,Pathways in cancer,Measles,ErbB signaling pathway,T cell receptor signaling pathway	0.00447602	23797,74769,56637,208650
MCAO 24 h	Kegg:04724,Kegg:05414,Kegg:04020,Kegg:04270,Kegg:04912	Glutamatergic synapse,Dilated cardiomyopathy,Calcium signaling pathway,Vascular smooth muscle contraction,GnRH signaling pathway	0.00447602	12288,11515,12289,210044
MCAO 24 h	Kegg:04722,Kegg:04662	Neurotrophin signaling pathway,B cell receptor signaling pathway	0.00488223	23797,234779,74769,56637,110157,18037
MCAO 24 h	Panther:P00019,Kegg:04540,Kegg:04270,Kegg:04912	Endothelin signaling pathway,Gap junction,Vascular smooth muscle contraction,GnRH signaling pathway	0.00524124	11515,16438,110157,210044,14682
MCAO 24 h	Kegg:04062,Kegg:04510,Kegg:04660,Kegg:04662	Chemokine signaling pathway,Focal adhesion,T cell receptor signaling pathway,B cell receptor signaling pathway	0.00524124	23797,74769,57257,56637,110157
MCAO 24 h	Kegg:04540,Kegg:04270,Kegg:04730	Gap junction,Vascular smooth muscle contraction,Long-term depression	0.00524124	16438,110157,14682,19091,18798
MCAO 24 h	Panther:P00047,Panther:P00010	PDGF signaling pathway,B cell activation	0.00524124	234779,74769,16438,57257,110157
MCAO 24 h	Kegg:04270,Kegg:04720,Kegg:04912	Vascular smooth muscle contraction,Long-term potentiation,GnRH signaling pathway	0.00524124	12288,16438,110157,14682,18798
MCAO 24 h	Kegg:04664,Kegg:04666	Fc epsilon RI signaling pathway,Fc gamma R-mediated phagocytosis	0.00531586	23797,234779,74769,57257,110157,18754
MCAO 24 h	Panther:P00019,Kegg:04724,Kegg:04540,Kegg:04972,Kegg:04970,Kegg:04020,Kegg:04270,Kegg:04971,Kegg:04912	Endothelin signaling pathway,Glutamatergic synapse,Gap junction,Pancreatic secretion,Salivary secretion,Calcium signaling pathway,Vascular smooth muscle contraction,Gastric acid secretion,GnRH signaling pathway	0.0053184	11515,16438,210044,14682
MCAO 24 h	Panther:P00019,Kegg:04062,Panther:P00031,Kegg:04914	Endothelin signaling pathway,Chemokine signaling pathway,Inflammation mediated by chemokine and cytokine signaling pathway,Progesterone-mediated oocyte maturation	0.0053184	23797,74769,110157,210044
MCAO 24 h	Kegg:04930,Kegg:04973	Type II diabetes mellitus,Carbohydrate digestion and absorption	0.0053184	15277,74769,12289,15275

MCAO 24 h	Kegg:04910,Panther:P00019,Kegg:05160,Kegg:04722,Kegg:05200,Kegg:04062,Kegg:05212,Panther:P00031,Kegg:04510,Kegg:05211,Kegg:05214,Kegg:05215,Kegg:05218,Panther:P00005,Kegg:05221,Panther:P00036,Kegg:04914,Kegg:05210,Kegg:05213,Kegg:04012,Kegg:04660,Panther:P04393,Panther:P00053,Kegg:04370,Kegg:04662,Kegg:04664,Kegg:04666,Panther:P00018,Panther:P00021,Kegg:05220,Kegg:05223	Insulin signaling pathway,Endothelin signaling pathway,Hepatitis C,Neurotrophin signaling pathway,Pathways in cancer,Chemokine signaling pathway,Pancreatic cancer,Inflammation mediated by chemokine and cytokine signaling pathway,Focal adhesion,Renal cell carcinoma,Glioma,Prostate cancer,Melanoma,Angiogenesis,Acute myeloid leukemia,Interleukin signaling pathway,Progesterone-mediated oocyte maturation,Colorectal cancer,Endometrial cancer,ErbB signaling pathway,T cell receptor signaling pathway,Ras Pathway,T cell activation,VEGF signaling pathway,B cell receptor signaling pathway,Fc epsilon RI signaling pathway,Fc gamma R-mediated phagocytosis,EGF receptor signaling pathway,FGF signaling pathway,Chronic myeloid leukemia,Non-small cell lung cancer	0.00576784	23797,74769,110157
MCAO 24 h	Panther:P00026,Kegg:04972,Kegg:04970,Kegg:04020,Kegg:04971	Heterotrimeric G-protein signaling pathway-Gi alpha and Gs alpha mediated pathway,Pancreatic secretion,Salivary secretion,Calcium signaling pathway,Gastric acid secretion	0.00576784	11515,210044,12671
MCAO 24 h	Kegg:04916,Kegg:05016,Kegg:04720	Melanogenesis,Huntington's disease,Long-term potentiation	0.00576784	12914,14682,18798
MCAO 24 h	Kegg:04020,Panther:P00003	Calcium signaling pathway,Alzheimer disease-amyloid secretase pathway	0.00576784	12288,12289,12671
MCAO 24 h	Panther:P00019,Kegg:04062,Kegg:04914	Endothelin signaling pathway,Chemokine signaling pathway,Progesterone-mediated oocyte maturation	0.00589972	23797,11515,74769,110157,210044
MCAO 24 h	Panther:P00047,Kegg:04662	PDGF signaling pathway,B cell receptor signaling pathway	0.00589972	234779,74769,57257,56637,110157
MCAO 24 h	Kegg:04540,Kegg:04916,Kegg:04270,Kegg:04912	Gap junction,Melanogenesis,Vascular smooth muscle contraction,GnRH signaling pathway	0.00622278	11515,110157,210044,14682,18798
MCAO 24 h	Kegg:04650,Kegg:04666	Natural killer cell mediated cytotoxicity,Fc gamma R-mediated phagocytosis	0.00622278	18479,234779,74769,57257,110157
MCAO 24 h	Kegg:04012,Kegg:04666	ErbB signaling pathway,Fc gamma R-mediated phagocytosis	0.00622278	23797,18479,234779,74769,110157
MCAO 24 h	Kegg:04662,Kegg:04664,Kegg:04666	B cell receptor signaling pathway,Fc epsilon RI signaling pathway,Fc gamma R-mediated phagocytosis	0.00622278	23797,234779,74769,57257,110157

MCAO 24 h	Kegg:04724,Kegg:04540,Kegg:04916,Kegg:04972,Kegg:04970,Kegg:04020,Kegg:04270,Kegg:04971,Kegg:04912	Glutamatergic synapse,Gap junction,Melanogenesis,Pancreatic secretion,Salivary secretion,Calcium signaling pathway,Vascular smooth muscle contraction,Gastric acid secretion,GnRH signaling pathway	0.00625592	11515,210044,14682,18798
MCAO 24 h	Kegg:05200,Kegg:05211,Panther:P00030	Pathways in cancer,Renal cell carcinoma,Hypoxia response via HIF activation	0.00625592	23797,12914,74769,15251
MCAO 24 h	Kegg:04540,Kegg:04270,Kegg:04720,Kegg:04730,Kegg:04912	Gap junction,Vascular smooth muscle contraction,Long-term potentiation,Long-term depression,GnRH signaling pathway	0.00625592	16438,110157,14682,18798
MCAO 24 h	Kegg:04670,Panther:P00047,Kegg:04650,Kegg:04662,Kegg:04664,Kegg:04666,Panther:P00010	Leukocyte transendothelial migration,PDGF signaling pathway,Natural killer cell mediated cytotoxicity,B cell receptor signaling pathway,Fc epsilon RI signaling pathway,Fc gamma R-mediated phagocytosis,B cell activation	0.00678226	234779,74769,57257
MCAO 24 h	Kegg:04724,Kegg:05414,Panther:P04377,Panther:P04378,Kegg:04020,Kegg:04270,Kegg:04912	Glutamatergic synapse,Dilated cardiomyopathy,Beta1 adrenergic receptor signaling pathway,Beta2 adrenergic receptor signaling pathway,Calcium signaling pathway,Vascular smooth muscle contraction,GnRH signaling pathway	0.00678226	12288,12289,210044
MCAO 24 h	Kegg:04010,Kegg:04062,Kegg:04510,Kegg:05211,Kegg:04012,Kegg:04660,Kegg:04666	MAPK signaling pathway,Chemokine signaling pathway,Focal adhesion,Renal cell carcinoma,ErbB signaling pathway,T cell receptor signaling pathway,Fc gamma R-mediated phagocytosis	0.00678226	23797,18479,110157
MCAO 24 h	Kegg:04722,Kegg:05200,Panther:P00031,Kegg:05214,Panther:P00047,Panther:P00005,Kegg:04650,Kegg:04012,Panther:P00056,Kegg:04370,Kegg:04662,Kegg:04664,Kegg:04666,Panther:P00018,Panther:P00021,Panther:P00010,Kegg:05223	Neurotrophin signaling pathway,Pathways in cancer,Inflammation mediated by chemokine and cytokine signaling pathway,Glioma,PDGF signaling pathway,Angiogenesis,Natural killer cell mediated cytotoxicity,ErbB signaling pathway,VEGF signaling pathway,VEGF signaling pathway,B cell receptor signaling pathway,Fc epsilon RI signaling pathway,Fc gamma R-mediated phagocytosis,EGF receptor signaling pathway,FGF signaling pathway,B cell activation,Non-small cell lung cancer	0.00678226	234779,74769,110157
MCAO 24 h	Kegg:05200,Kegg:05152,Kegg:05211,Kegg:05215	Pathways in cancer,Tuberculosis,Renal cell carcinoma,Prostate cancer	0.00678226	23797,12914,110157
MCAO 24 h	Panther:P00031,Panther:P00047,Panther:P00010,Kegg:04070	Inflammation mediated by chemokine and cytokine signaling pathway,PDGF signaling pathway,B cell activation,Phosphatidylinositol signaling system	0.00678226	234779,74769,16438

MCAO 24 h	Kegg:04720	Long-term potentiation	0.00680694	12288,12914,16438,110157,14682,18798,14812,14811
MCAO 24 h	Kegg:04910,Kegg:04973	Insulin signaling pathway,Carbohydrate digestion and absorption	0.0068274	23797,15277,74769,15275
MCAO 24 h	Panther:P00026,Kegg:04916	Heterotrimeric G-protein signaling pathway-Gi alpha and Gs alpha mediated pathway,Melanogenesis	0.0068274	12914,11515,56637,210044
MCAO 24 h	Kegg:04062,Kegg:04510,Kegg:04810,Kegg:04650,Kegg:04660,Kegg:04666	Chemokine signaling pathway,Focal adhesion,Regulation of actin cytoskeleton,Natural killer cell mediated cytotoxicity,T cell receptor signaling pathway,Fc gamma R-mediated phagocytosis	0.0068274	18479,74769,57257,110157
MCAO 24 h	Kegg:04062,Kegg:04510,Kegg:05211,Kegg:04012,Kegg:04660,Kegg:04666	Chemokine signaling pathway,Focal adhesion,Renal cell carcinoma,ErbB signaling pathway,T cell receptor signaling pathway,Fc gamma R-mediated phagocytosis	0.0068274	23797,18479,74769,110157
MCAO 24 h	Kegg:04062,Kegg:04020,Kegg:04912	Chemokine signaling pathway,Calcium signaling pathway,GnRH signaling pathway	0.0068274	11515,210044,19229,18798
MCAO 24 h	Panther:P04391,Panther:P04374,Kegg:04020	Oxytocin receptor mediated signaling pathway,5HT2 type receptor mediated signaling pathway,Calcium signaling pathway	0.0068274	12288,234779,12289,14682
MCAO 24 h	Panther:P04391,Panther:P04374	Oxytocin receptor mediated signaling pathway,5HT2 type receptor mediated signaling pathway	0.00682776	12288,14702,234779,12289,14682,18754,12296
MCAO 24 h	Panther:P00042	Muscarinic acetylcholine receptor 1 and 3 signaling pathway	0.00682776	16536,14702,16438,14682,18754,12671,14811
MCAO 24 h	Kegg:04012,Kegg:04660	ErbB signaling pathway,T cell receptor signaling pathway	0.00684996	23797,18479,74769,56637,110157,208650
MCAO 24 h	Kegg:04270	Vascular smooth muscle contraction	0.00687392	12288,11515,16438,16531,12289,110157,210044,14682,18754,19091,18798
MCAO 24 h	Panther:P00027,Panther:P00042	Heterotrimeric G-protein signaling pathway-Gq alpha and Go alpha mediated pathway,Muscarinic acetylcholine receptor 1 and 3 signaling pathway	0.00706056	14702,16438,14682,18754,12671
MCAO 24 h	Kegg:04973	Carbohydrate digestion and absorption	0.00725085	23797,15277,74769,12289,11931,15275
MCAO 24 h	Kegg:04910,Kegg:05200,Kegg:04012,Kegg:04660	Insulin signaling pathway,Pathways in cancer,ErbB signaling pathway,T cell receptor signaling pathway	0.00727038	23797,74769,56637,110157,208650
MCAO 24 h	Kegg:04722,Kegg:05200,Panther:P00047,Panther:P00005,Kegg:04012,Kegg:04662	Neurotrophin signaling pathway,Pathways in cancer,PDGF signaling pathway,Angiogenesis,ErbB signaling pathway,B cell receptor signaling pathway	0.00763372	234779,74769,56637,110157

MCAO 24 h	Kegg:04062,Kegg:04510,Kegg:04660,Panther:P00053,Kegg:04662,Kegg:04664,Kegg:04666	Chemokine signaling pathway,Focal adhesion,T cell receptor signaling pathway,T cell activation,B cell receptor signaling pathway,Fc epsilon RI signaling pathway,Fc gamma R-mediated phagocytosis	0.00763372	23797,74769,57257,110157
MCAO 24 h	Panther:P00031,Panther:P00047,Panther:P00010	Inflammation mediated by chemokine and cytokine signaling pathway,PDGF signaling pathway,B cell activation	0.00763372	234779,74769,16438,110157
MCAO 24 h	Kegg:04540,Kegg:04970,Kegg:04270,Kegg:04730	Gap junction,Salivary secretion,Vascular smooth muscle contraction,Long-term depression	0.00763372	16438,14682,19091,18798
MCAO 24 h	Kegg:04062,Kegg:04916	Chemokine signaling pathway,Melanogenesis	0.00804764	11515,56637,110157,210044,18798
MCAO 24 h	Kegg:04972	Pancreatic secretion	0.00805769	11515,16438,16531,16535,210044,14682,11931,12671,18798,54403
MCAO 24 h	Panther:P00019,Panther:P00031,Kegg:04270	Endothelin signaling pathway,Inflammation mediated by chemokine and cytokine signaling pathway,Vascular smooth muscle contraction	0.00858646	16438,110157,210044,18754
MCAO 24 h	Kegg:04722,Panther:P00031,Kegg:04660,Kegg:04662	Neurotrophin signaling pathway,Inflammation mediated by chemokine and cytokine signaling pathway,T cell receptor signaling pathway,B cell receptor signaling pathway	0.00858646	23797,74769,110157,18037
MCAO 24 h	Kegg:04650,Kegg:04012,Kegg:04666	Natural killer cell mediated cytotoxicity,ErbB signaling pathway,Fc gamma R-mediated phagocytosis	0.00858646	18479,234779,74769,110157
MCAO 24 h	Kegg:04910,Kegg:05200,Kegg:04630,Kegg:05162,Kegg:04012,Kegg:04660,Panther:P00018,Kegg:05220	Insulin signaling pathway,Pathways in cancer,Jak-STAT signaling pathway,Measles,ErbB signaling pathway,T cell receptor signaling pathway,EGF receptor signaling pathway,Chronic myeloid leukemia	0.00863481	23797,74769,208650
MCAO 24 h	Panther:P00019,Kegg:04724,Kegg:04540,Kegg:04114,Kegg:04972,Kegg:04970,Kegg:04020,Kegg:04270,Kegg:04971,Kegg:04912	Endothelin signaling pathway,Glutamatergic synapse,Gap junction,Oocyte meiosis,Pancreatic secretion,Salivary secretion,Calcium signaling pathway,Vascular smooth muscle contraction,Gastric acid secretion,GnRH signaling pathway	0.00863481	11515,16438,210044
MCAO 24 h	Panther:P00019,Panther:P00027,Kegg:04270,Panther:P00057,Panther:P00042,Panther:P04385	Endothelin signaling pathway,Heterotrimeric G-protein signaling pathway-Gq alpha and Go alpha mediated pathway,Vascular smooth muscle contraction,Wnt signaling pathway,Muscarinic acetylcholine receptor 1 and 3 signaling pathway,Histamine H1 receptor mediated signaling pathway	0.00863481	16438,14682,18754

MCAO 24 h	Panther:P00019,Panther:P00031,Panther:P00047,Panther:P00053,Panther:P00010	Endothelin signaling pathway,Inflammation mediated by chemokine and cytokine signaling pathway,PDGF signaling pathway,T cell activation,B cell activation	0.00863481	74769,16438,110157
MCAO 24 h	Kegg:04724,Kegg:04540,Kegg:04916,Kegg:04972,Kegg:04970,Kegg:04020,Kegg:04270,Kegg:04971,Kegg:04912,Kegg:04961	Glutamatergic synapse,Gap junction,Melanogenesis,Pancreatic secretion,Salivary secretion,Calcium signaling pathway,Vascular smooth muscle contraction,Gastric acid secretion,GnRH signaling pathway,Endocrine and other factor-regulated calcium reabsorption	0.00863481	11515,14682,18798
MCAO 24 h	Kegg:04722,Kegg:05200,Kegg:04380,Panther:P00031,Kegg:05214,Panther:P00005,Kegg:04012,Kegg:04370,Kegg:04662,Kegg:04664,Kegg:04666,Panther:P00018,Panther:P00021,Kegg:05223	Neurotrophin signaling pathway,Pathways in cancer,Osteoclast differentiation,Inflammation mediated by chemokine and cytokine signaling pathway,Glioma,Angiogenesis,ErbB signaling pathway,VEGF signaling pathway,B cell receptor signaling pathway,Fc epsilon RI signaling pathway,Fc gamma R-mediated phagocytosis,EGF receptor signaling pathway,FGF signaling pathway,Non-small cell lung cancer	0.00863481	23797,234779,74769
MCAO 24 h	Kegg:04012,Panther:P00018	ErbB signaling pathway,EGF receptor signaling pathway	0.0088156	23797,234779,74769,13867,110157,208650
MCAO 24 h	Kegg:04062	Chemokine signaling pathway	0.00911687	14702,23797,18479,11515,74769,57257,56637,21844,110355,110157,210044,19229,18798,277360
MCAO 24 h	Panther:P00047	PDGF signaling pathway	0.00960617	94190,69257,50768,234779,74769,16438,57257,56637,110157,78514
MCAO 24 h	Kegg:04722,Kegg:04660,Kegg:04662	Neurotrophin signaling pathway,T cell receptor signaling pathway,B cell receptor signaling pathway	0.00968345	23797,74769,56637,110157,18037
MCAO 24 h	Kegg:05200,Kegg:04012,Panther:P00018	Pathways in cancer,ErbB signaling pathway,EGF receptor signaling pathway	0.00968345	23797,234779,74769,110157,208650
MCAO 24 h	Kegg:04062,Kegg:04912	Chemokine signaling pathway,GnRH signaling pathway	0.00968345	11515,110157,210044,19229,18798
MCAO 24 h	Panther:P00019,Kegg:04540,Kegg:04916,Kegg:04270,Kegg:04912	Endothelin signaling pathway,Gap junction,Melanogenesis,Vascular smooth muscle contraction,GnRH signaling pathway	0.00975937	11515,110157,210044,14682
MCAO 24 h	Kegg:04964	Proximal tubule bicarbonate reclamation	0.00975937	216456,11931,54403,14660
MCAO 24 h	Kegg:04062,Kegg:04510,Panther:P00047,Kegg:04660,Kegg:04662	Chemokine signaling pathway,Focal adhesion,PDGF signaling pathway,T cell receptor signaling pathway,B cell receptor signaling pathway	0.00975937	74769,57257,56637,110157
MCAO 24 h	Panther:P00047,Panther:P00053,Panther:P00010	PDGF signaling pathway,T cell activation,B cell activation	0.00975937	74769,16438,57257,110157

MCAO 24 h	Kegg:04972,Kegg:04970,Kegg:04971,Kegg:04961	Pancreatic secretion,Salivary secretion,Gastric acid secretion,Endocrine and other factor-regulated calcium reabsorption	0.00975937	11515,14682,11931,18798
MCAO 24 h	Panther:P00019,Panther:P00031,Panther:P00005,Panther:P00006,Kegg:04664,Kegg:04666,Panther:P00018,Panther:P00021	Endothelin signaling pathway,Inflammation mediated by chemokine and cytokine signaling pathway,Angiogenesis,Apoptosis signaling pathway,Fc epsilon RI signaling pathway,Fc gamma R-mediated phagocytosis,EGF receptor signaling pathway,FGF signaling pathway	0.0103323	23797,74769,18754
MCAO 24 h	Panther:P00019,Panther:P00031,Panther:P00005,Panther:P00056,Kegg:04664,Kegg:04666,Panther:P00018,Panther:P00021	Endothelin signaling pathway,Inflammation mediated by chemokine and cytokine signaling pathway,Angiogenesis,VEGF signaling pathway,Fc epsilon RI signaling pathway,Fc gamma R-mediated phagocytosis,EGF receptor signaling pathway,FGF signaling pathway	0.0103323	74769,110157,18754
MCAO 24 h	Panther:P00019,Kegg:05142,Panther:P00048	Endothelin signaling pathway,Chagas disease (American trypanosomiasis),PI3 kinase pathway	0.0103323	23797,74769,14682
MCAO 24 h	Kegg:05200,Kegg:05211,Panther:P00005,Kegg:04150,Panther:P00030	Pathways in cancer,Renal cell carcinoma,Angiogenesis,mTOR signaling pathway,Hypoxia response via HIF activation	0.0103323	23797,74769,15251
MCAO 24 h	Kegg:05010,Kegg:04916	Alzheimer's disease,Melanogenesis	0.0103323	56637,14682,18798
MCAO 24 h	Kegg:04062,Kegg:04650	Chemokine signaling pathway,Natural killer cell mediated cytotoxicity	0.0106987	18479,74769,57257,110157,19229
MCAO 24 h	Kegg:04070	Phosphatidylinositol signaling system	0.0109167	234779,74769,16438,18704,20975,380921,18798,320127
MCAO 24 h	Kegg:04910,Kegg:05200,Kegg:04012,Kegg:04660,Panther:P00018,Kegg:05220	Insulin signaling pathway,Pathways in cancer,ErbB signaling pathway,T cell receptor signaling pathway,EGF receptor signaling pathway,Chronic myeloid leukemia	0.0110913	23797,74769,110157,208650
MCAO 24 h	Kegg:05200,Panther:P00005,Panther:P00056	Pathways in cancer,Angiogenesis,VEGF signaling pathway	0.0110913	234779,74769,15251,110157
MCAO 24 h	Kegg:04910,Kegg:04722,Kegg:04510	Insulin signaling pathway,Neurotrophin signaling pathway,Focal adhesion	0.0115226	23797,74769,56637,110157,107746
MCAO 24 h	Kegg:04912	GnRH signaling pathway	0.0122924	12288,11515,16438,12289,110157,210044,14682,19229,18798
MCAO 24 h	Panther:P00026	Heterotrimeric G-protein signaling pathway-Gi alpha and Gs alpha mediated pathway	0.0127886	108069,50779,14702,12914,11515,56637,110094,110355,102093,210044,12671

MCAO 24 h	Panther:P00019,Kegg:04724,Kegg:04540,Kegg:04916,Kegg:04972,Kegg:04970,Kegg:04020,Kegg:04270,Kegg:04971,Kegg:04912	Endothelin signaling pathway,Glutamatergic synapse,Gap junction,Melanogenesis,Pancreatic secretion,Salivary secretion,Calcium signaling pathway,Vascular smooth muscle contraction,Gastric acid secretion,GnRH signaling pathway	0.0127978	11515,210044,14682
MCAO 24 h	Kegg:05200,Kegg:04630,Kegg:05211,Kegg:05215,Panther:P00059,Panther:P00030	Pathways in cancer,Jak-STAT signaling pathway,Renal cell carcinoma,Prostate cancer,p53 pathway,Hypoxia response via HIF activation	0.0127978	23797,12914,74769
MCAO 24 h	Kegg:04062,Kegg:04510,Kegg:04810,Panther:P00047,Kegg:04650,Kegg:04660,Panther:P00053,Kegg:04662,Kegg:04664,Kegg:04666,Panther:P00010	Chemokine signaling pathway,Focal adhesion,Regulation of actin cytoskeleton,PDGF signaling pathway,Natural killer cell mediated cytotoxicity,T cell receptor signaling pathway,T cell activation,B cell receptor signaling pathway,Fc epsilon RI signaling pathway,Fc gamma R-mediated phagocytosis,B cell activation	0.0127978	74769,57257,110157
MCAO 24 h	Kegg:04724,Panther:P04391,Panther:P04374	Glutamatergic synapse,Oxytocin receptor mediated signaling pathway,5HT2 type receptor mediated signaling pathway	0.0128572	12288,14702,12289,14682
MCAO 24 h	Kegg:04062,Kegg:04540,Kegg:04916,Kegg:04270,Kegg:04912	Chemokine signaling pathway,Gap junction,Melanogenesis,Vascular smooth muscle contraction,GnRH signaling pathway	0.0128572	11515,110157,210044,18798
MCAO 24 h	Panther:P00026,Kegg:04724	Heterotrimeric G-protein signaling pathway-Gi alpha and Gs alpha mediated pathway,Glutamatergic synapse	0.0140164	108069,14702,11515,110355,210044
MCAO 24 h	Panther:P00026,Kegg:04062	Heterotrimeric G-protein signaling pathway-Gi alpha and Gs alpha mediated pathway,Chemokine signaling pathway	0.0140164	14702,11515,56637,110355,210044
MCAO 24 h	Panther:P00026,Kegg:04020	Heterotrimeric G-protein signaling pathway-Gi alpha and Gs alpha mediated pathway,Calcium signaling pathway	0.0140164	11515,110094,102093,210044,12671
MCAO 24 h	Kegg:04724,Panther:P04377,Panther:P04378	Glutamatergic synapse,Beta1 adrenergic receptor signaling pathway,Beta2 adrenergic receptor signaling pathway	0.0145693	12288,14702,12289,210044
MCAO 24 h	Kegg:04910,Kegg:04930,Kegg:04973	Insulin signaling pathway,Type II diabetes mellitus,Carbohydrate digestion and absorption	0.0155572	15277,74769,15275
MCAO 24 h	Panther:P00026,Kegg:04062,Kegg:04916	Heterotrimeric G-protein signaling pathway-Gi alpha and Gs alpha mediated pathway,Chemokine signaling pathway,Melanogenesis	0.0155572	11515,56637,210044
MCAO 24 h	Kegg:04724,Kegg:04062,Kegg:04540,Kegg:04916,Kegg:04972,Kegg:04970,Kegg:04020,Kegg:04270,Kegg:04971,Kegg:04912	Glutamatergic synapse,Chemokine signaling pathway,Gap junction,Melanogenesis,Pancreatic secretion,Salivary secretion,Calcium signaling pathway,Vascular smooth muscle contraction,Gastric acid secretion,GnRH signaling pathway	0.0155572	11515,210044,18798

MCAO 24 h	Kegg:04540,Kegg:04916,Kegg:04270,Kegg:04720,Kegg:04730,Kegg:04912	Gap junction,Melanogenesis,Vascular smooth muscle contraction,Long-term potentiation,Long-term depression,GnRH signaling pathway	0.0155572	110157,14682,18798
MCAO 24 h	Kegg:04930	Type II diabetes mellitus	0.0156488	12288,15277,74769,12289,18754,15275
MCAO 24 h	Panther:P04377,Panther:P04378	Beta1 adrenergic receptor signaling pathway,Beta2 adrenergic receptor signaling pathway	0.0156732	12288,14702,12289,210044,12296
MCAO 24 h	Kegg:04910,Kegg:04722,Kegg:04510,Kegg:05211	Insulin signaling pathway,Neurotrophin signaling pathway,Focal adhesion,Renal cell carcinoma	0.0161824	23797,74769,110157,107746
MCAO 24 h	Kegg:05200,Kegg:05211,Panther:P00005	Pathways in cancer,Renal cell carcinoma,Angiogenesis	0.0161824	23797,74769,15251,110157
MCAO 24 h	Panther:P00027,Panther:P00057,Panther:P00042,Panther:P04385	Heterotrimeric G-protein signaling pathway-Gq alpha and Go alpha mediated pathway,Wnt signaling pathway,Muscarinic acetylcholine receptor 1 and 3 signaling pathway,Histamine H1 receptor mediated signaling pathway	0.0161824	14702,16438,14682,18754
MCAO 24 h	Kegg:04540	Gap junction	0.01716	11515,16438,110157,22154,210044,14682,19091,18798
MCAO 24 h	Kegg:05200,Kegg:04012	Pathways in cancer,ErbB signaling pathway	0.0179307	23797,234779,74769,56637,110157,208650
MCAO 24 h	Panther:P00019,Kegg:04062,Kegg:04540,Kegg:04914,Kegg:04916,Kegg:04270,Kegg:04912	Endothelin signaling pathway,Chemokine signaling pathway,Gap junction,Progesterone-mediated oocyte maturation,Melanogenesis,Vascular smooth muscle contraction,GnRH signaling pathway	0.0181881	11515,110157,210044
MCAO 24 h	Panther:P00019,Kegg:04540,Kegg:04270,Kegg:04720,Kegg:04730,Kegg:04912	Endothelin signaling pathway,Gap junction,Vascular smooth muscle contraction,Long-term potentiation,Long-term depression,GnRH signaling pathway	0.0181881	16438,110157,14682
MCAO 24 h	Panther:P00026,Kegg:04724,Kegg:04062,Panther:P00031	Heterotrimeric G-protein signaling pathway-Gi alpha and Gs alpha mediated pathway,Glutamatergic synapse,Chemokine signaling pathway,Inflammation mediated by chemokine and cytokine signaling pathway	0.0181881	14702,110355,210044
MCAO 24 h	Kegg:04724,Kegg:04080,Panther:P00037	Glutamatergic synapse,Neuroactive ligand-receptor interaction,Ionotropic glutamate receptor pathway	0.0181881	108069,14806,14811

MCAO 24 h	Kegg:04062,Kegg:04510,Kegg:04810,Kegg:05211,Kegg:04650,Kegg:04012,Kegg:04660,Kegg:04666	Chemokine signaling pathway,Focal adhesion,Regulation of actin cytoskeleton,Renal cell carcinoma,Natural killer cell mediated cytotoxicity,ErbB signaling pathway,T cell receptor signaling pathway,Fc gamma R-mediated phagocytosis	0.0181881	18479,74769,110157
MCAO 24 h	Kegg:05016,Kegg:04961	Huntington's disease,Endocrine and other factor-regulated calcium reabsorption	0.0181881	14682,71770,18798
MCAO 24 h	Kegg:04670,Kegg:04650	Leukocyte transendothelial migration,Natural killer cell mediated cytotoxicity	0.0184201	234779,74769,57257,19229
MCAO 24 h	Kegg:04724,Kegg:04080	Glutamatergic synapse,Neuroactive ligand-receptor interaction	0.0184201	108069,14812,14806,14811
MCAO 24 h	Kegg:04660,Kegg:04662	T cell receptor signaling pathway,B cell receptor signaling pathway	0.0186385	23797,74769,57257,56637,110157,18037
MCAO 24 h	Panther:P00026,Kegg:04724,Kegg:04062	Heterotrimeric G-protein signaling pathway-Gi alpha and Gs alpha mediated pathway,Glutamatergic synapse,Chemokine signaling pathway	0.0199814	14702,11515,110355,210044
MCAO 24 h	Kegg:04976,Kegg:04972	Bile secretion,Pancreatic secretion	0.0199814	11515,210044,11931,54403
MCAO 24 h	Panther:P00059,Panther:P04398	p53 pathway,p53 pathway feedback loops 2	0.0199814	23797,74769,20437,53892
MCAO 24 h	Panther:P00003	Alzheimer disease-amyloid secretase pathway	0.0202822	12288,12289,11491,18754,12671,12296
MCAO 24 h	Kegg:04012	ErbB signaling pathway	0.0206631	23797,18479,234779,74769,56637,13867,110157,208650
MCAO 24 h	Kegg:04062,Kegg:04810	Chemokine signaling pathway,Regulation of actin cytoskeleton	0.0211283	18479,74769,57257,21844,110157
MCAO 24 h	Panther:P04385	Histamine H1 receptor mediated signaling pathway	0.0211283	14702,234779,16438,14682,18754
MCAO 24 h	Kegg:05010,Panther:P00057	Alzheimer's disease,Wnt signaling pathway	0.0211727	16438,56637,14682
MCAO 24 h	Kegg:04260	Cardiac muscle contraction	0.021272	12288,12300,12289,11931,54376,12296,12293
MCAO 24 h	Kegg:05200,Panther:P00005	Pathways in cancer,Angiogenesis	0.0212821	23797,234779,74769,56637,15251,110157
MCAO 24 h	Kegg:04724,Panther:P00031	Glutamatergic synapse,Inflammation mediated by chemokine and cytokine signaling pathway	0.0218383	14702,16438,110355,210044
MCAO 24 h	Kegg:04724,Panther:P00042	Glutamatergic synapse,Muscarinic acetylcholine receptor 1 and 3 signaling pathway	0.0218383	14702,16438,14682,14811

MCAO 24 h	Panther:P00031,Panther:P04385	Inflammation mediated by chemokine and cytokine signaling pathway,Histamine H1 receptor mediated signaling pathway	0.0218383	14702,234779,16438,18754
MCAO 24 h	Kegg:05414	Dilated cardiomyopathy	0.0222743	12288,12300,11515,12289,210044,54376,12296,12293
MCAO 24 h	Kegg:05211	Renal cell carcinoma	0.0222759	23797,12914,18479,74769,15251,110157,107746
MCAO 24 h	Kegg:04724,Kegg:04062	Glutamatergic synapse,Chemokine signaling pathway	0.0223269	14702,11515,110355,210044,18798
MCAO 24 h	Panther:P00019,Panther:P00031,Kegg:04540,Kegg:04270,Kegg:04912	Endothelin signaling pathway,Inflammation mediated by chemokine and cytokine signaling pathway,Gap junction,Vascular smooth muscle contraction,GnRH signaling pathway	0.0240474	16438,110157,210044
MCAO 24 h	Panther:P00026,Panther:P00057	Heterotrimeric G-protein signaling pathway-Gi alpha and Gs alpha mediated pathway,Wnt signaling pathway	0.0240474	14702,12914,56637
MCAO 24 h	Kegg:04670,Kegg:04062,Kegg:04650	Leukocyte transendothelial migration,Chemokine signaling pathway,Natural killer cell mediated cytotoxicity	0.0240474	74769,57257,19229
MCAO 24 h	Kegg:05200,Kegg:05211,Panther:P00005,Panther:P00056	Pathways in cancer,Renal cell carcinoma,Angiogenesis,VEGF signaling pathway	0.0240474	74769,15251,110157
MCAO 24 h	Panther:P00027,Panther:P00031,Panther:P00057,Panther:P00042,Panther:P04385	Heterotrimeric G-protein signaling pathway-Gq alpha and Go alpha mediated pathway,Inflammation mediated by chemokine and cytokine signaling pathway,Wnt signaling pathway,Muscarinic acetylcholine receptor 1 and 3 signaling pathway,Histamine H1 receptor mediated signaling pathway	0.0240474	14702,16438,18754
MCAO 24 h	Kegg:05010,Panther:P00003	Alzheimer's disease,Alzheimer disease-amyloid secretase pathway	0.0240474	12288,12289,11491
MCAO 24 h	Panther:P04377,Panther:P04391,Panther:P04378,Panther:P04374	Beta1 adrenergic receptor signaling pathway,Oxytocin receptor mediated signaling pathway,Beta2 adrenergic receptor signaling pathway,5HT2 type receptor mediated signaling pathway	0.0243981	12288,14702,12289,12296
MCAO 24 h	Kegg:05412	Arrhythmogenic right ventricular cardiomyopathy (ARVC)	0.0261172	12288,12300,12558,12289,54376,12296,12293
MCAO 24 h	Kegg:05160,Panther:P00021	Hepatitis C,FGF signaling pathway	0.0265499	23797,74769,110157,72930
MCAO 24 h	Kegg:04510,Kegg:05211	Focal adhesion,Renal cell carcinoma	0.0275833	23797,18479,74769,110157,107746
MCAO 24 h	Kegg:04662	B cell receptor signaling pathway	0.0276651	23797,234779,74769,57257,56637,110157,18037

MCAO 24 h	Kegg:04910,Panther:P00026	Insulin signaling pathway,Heterotrimeric G-protein signaling pathway-Gi alpha and Gs alpha mediated pathway	0.0276837	56637,110094,102093
MCAO 24 h	Kegg:04724,Panther:P04377,Panther:P04391,Panther:P04378,Panther:P04374	Glutamatergic synapse,Beta1 adrenergic receptor signaling pathway,Oxytocin receptor mediated signaling pathway,Beta2 adrenergic receptor signaling pathway,5HT2 type receptor mediated signaling pathway	0.0276837	12288,14702,12289
MCAO 24 h	Kegg:05200,Kegg:05215,Kegg:04916	Pathways in cancer,Prostate cancer,Melanogenesis	0.0276837	12914,56637,110157
MCAO 24 h	Kegg:04020,Panther:P04385	Calcium signaling pathway,Histamine H1 receptor mediated signaling pathway	0.0276837	234779,16438,14682
MCAO 24 h	Kegg:00770	Pantothenate and CoA biosynthesis	0.0276837	99586,12036,12035
MCAO 24 h	Kegg:05010	Alzheimer's disease	0.0285209	12288,16438,20617,12289,56637,14682,11491,18798,14812,20192,14811
MCAO 24 h	Kegg:05200,Kegg:05211,Kegg:05215	Pathways in cancer,Renal cell carcinoma,Prostate cancer	0.0286351	23797,12914,74769,110157
MCAO 24 h	Kegg:04916,Kegg:04720	Melanogenesis,Long-term potentiation	0.0286351	12914,110157,14682,18798
MCAO 24 h	Kegg:04062,Panther:P00031	Chemokine signaling pathway,Inflammation mediated by chemokine and cytokine signaling pathway	0.030032	14702,23797,74769,110355,110157,210044,277360
MCAO 24 h	Kegg:04724,Panther:P00027,Panther:P00057,Panther:P00042,Panther:P04385	Glutamatergic synapse,Heterotrimeric G-protein signaling pathway-Gq alpha and Go alpha mediated pathway,Wnt signaling pathway,Muscarinic acetylcholine receptor 1 and 3 signaling pathway,Histamine H1 receptor mediated signaling pathway	0.0317969	14702,16438,14682
MCAO 24 h	Kegg:05200,Kegg:05210	Pathways in cancer,Colorectal cancer	0.0342221	23797,74769,56637,110157,72993,13176
MCAO 24 h	Kegg:05410	Hypertrophic cardiomyopathy (HCM)	0.0402631	12288,12300,12289,54376,108099,12296,12293
MCAO 24 h	Panther:P04394,Panther:P04391,Panther:P04374	Thyrotropin-releasing hormone receptor signaling pathway,Oxytocin receptor mediated signaling pathway,5HT2 type receptor mediated signaling pathway	0.0408669	14702,234779,14682,18754,12296
MCAO 24 h	Kegg:04530,Panther:P00021	Tight junction,FGF signaling pathway	0.0421792	23797,18754,72930
MCAO 24 h	Panther:P00005,Panther:P00056	Angiogenesis,VEGF signaling pathway	0.0438062	234779,74769,15251,110157,18754
MCAO 24 h	Kegg:04724,Panther:P00027	Glutamatergic synapse,Heterotrimeric G-protein signaling pathway-Gq alpha and Go alpha mediated pathway	0.0463942	108069,14702,16438,14682

MCAO 24 h	Kegg:05200,Kegg:04630	Pathways in cancer,Jak-STAT signaling pathway	0.0463942	23797,12914,74769,208650
MCAO 24 h	Kegg:00562,Kegg:04070	Inositol phosphate metabolism,Phosphatidylinositol signaling system	0.0466392	234779,74769,18704,20975,18798
MCAO 24 h	Panther:P00026,Panther:P05731,Kegg:04724,Kegg:04062	Heterotrimeric G-protein signaling pathway-Gi alpha and Gs alpha mediated pathway,GABA-B receptor II signaling,Glutamatergic synapse,Chemokine signaling pathway	0.0467942	14702,11515,210044
MCAO 24 h	Panther:P00027,Panther:P04394,Panther:P04391,Panther:P04374,Panther:P00057,Panther:P00042,Panther:P04385	Heterotrimeric G-protein signaling pathway-Gq alpha and Go alpha mediated pathway,Thyrotropin-releasing hormone receptor signaling pathway,Oxytocin receptor mediated signaling pathway,5HT2 type receptor mediated signaling pathway,Wnt signaling pathway,Muscarinic acetylcholine receptor 1 and 3 signaling pathway,Histamine H1 receptor mediated signaling pathway	0.0467942	14702,14682,18754
MCAO 24 h	Kegg:04976,Kegg:04972,Kegg:04970,Kegg:04971	Bile secretion,Pancreatic secretion,Salivary secretion,Gastric acid secretion	0.0467942	11515,210044,11931
MCAO 24 h	Kegg:04666	Fc gamma R-mediated phagocytosis	0.0481799	23797,18479,234779,74769,57257,110157,18754
MCAO 24 h	Kegg:04920	Adipocytokine signaling pathway	0.0482842	68465,19017,12491,23797,18037,108099
MCAO 24 h	Panther:P04394,Panther:P04391,Panther:P04374,Panther:P04385	Thyrotropin-releasing hormone receptor signaling pathway,Oxytocin receptor mediated signaling pathway,5HT2 type receptor mediated signaling pathway,Histamine H1 receptor mediated signaling pathway	0.0493443	14702,234779,14682,18754

Supplementary Table S5. miRNA binding sites of the significantly differentially expressed circRNAs.

Group	circRNA	miRNA
MCAO 5min	mmu_circRNA_29699	mmu-miR-433-3p
MCAO 5min	mmu_circRNA_013636	
MCAO 5min	mmu_circRNA_33003	mmu-miR-31-5p

MCAO 5min	mmu_circRNA_001149	mmu-miR-1247-5p
MCAO 5min	mmu_circRNA_40539	mmu-miR-1896
MCAO 5min	mmu_circRNA_32884	NA
MCAO 5min	mmu_circRNA_26948	NA
MCAO 5min	mmu_circRNA_001579	NA
MCAO 5min	mmu_circRNA_19729	mmu-miR-542-5p
MCAO 5min	mmu_circRNA_18979	NA
MCAO 5min	mmu_circRNA_004072	NA
MCAO 5min	mmu_circRNA_010803	NA
MCAO 5min	mmu_circRNA_43867	NA
MCAO 5min	mmu_circRNA_25433	mmu-miR-30c-1-3p/mmu-miR-5623-5p
MCAO 5min	mmu_circRNA_45921	mmu-miR-1962
MCAO 5min	mmu_circRNA_22898	NA
MCAO 5min	mmu_circRNA_003169	mmu-miR-470-5p
MCAO 5min	mmu_circRNA_43344	NA
MCAO 5min	mmu_circRNA_003795	NA
MCAO 5min	mmu_circRNA_34491	mmu-miR-669p-3p
MCAO 5min	mmu_circRNA_41878	mmu-miR-361-3p,mmu-miR-3058-5p,mmu-miR-743a-5p
MCAO 5min	mmu_circRNA_35065	mmu-miR-344d-3-5p， mmu-miR-344d-1-5p
MCAO 5min	mmu_circRNA_42362	NA
MCAO 5min	mmu_circRNA_011174	NA
MCAO 5min	mmu_circRNA_35587	NA
MCAO 5min	mmu_circRNA_35588	NA
MCAO 5min	mmu_circRNA_43955	NA

MCAO 5min	mmu_circRNA_26021	mmu-miR-1940
MCAO 5min	mmu_circRNA_32190	mmu-miR-3057-5p
MCAO 5min	mmu_circRNA_20066	NA
MCAO 5min	mmu_circRNA_35589	NA
MCAO 3h	mmu_circRNA_40203	NA
MCAO 3h	mmu_circRNA_36481	NA
MCAO 3h	mmu_circRNA_45876	NA
MCAO 3h	mmu_circRNA_24344	mmu-miR-3093-3p
MCAO 3h	mmu_circRNA_27197	mmu-miR-5113
MCAO 3h	mmu_circRNA_010777	mmu-miR-3089-3p
MCAO 3h	mmu_circRNA_27565	NA
MCAO 3h	mmu_circRNA_43956	NA
MCAO 3h	mmu_circRNA_19178	NA
MCAO 3h	mmu_circRNA_19178	NA
MCAO 3h	mmu_circRNA_29760	mmu-miR-484，mmu-miR-693-3p
MCAO 3h	mmu_circRNA_30586	NA
MCAO 3h	mmu_circRNA_34968	NA
MCAO 3h	mmu_circRNA_011505	NA
MCAO 3h	mmu_circRNA_19015	NA
MCAO 3h	mmu_circRNA_27287	NA
MCAO 3h	mmu_circRNA_27821	NA
MCAO 3h	mmu_circRNA_015541	NA
MCAO 3h	mmu_circRNA_015541	NA
MCAO 3h	mmu_circRNA_001832	NA

MCAO 3h	mmu_circRNA_38760	NA
MCAO 3h	mmu_circRNA_26316	NA
MCAO 3h	mmu_circRNA_38328	mmu-miR-141-5p
MCAO 3h	mmu_circRNA_23643	mmu-miR-3070b-3p，mmu-miR-5046
MCAO 3h	mmu_circRNA_43344	NA
MCAO 3h	mmu_circRNA_45921	mmu-miR-1962
MCAO 3h	mmu_circRNA_37699	NA
MCAO 3h	mmu_circRNA_002246	NA
MCAO 24h	mmu_circRNA_43956	NA
MCAO 24h	mmu_circRNA_37286	mmu-miR-149-3p,mmu-miR-199a-5p,mmu-miR-214-3p,mmu-miR-466a-3p,mmu-miR-467a-3p,mmu-miR-467a-4p,mmu-miR-467a-5p,mmu-miR-467a-6p,mmu-miR-467a-7p,mmu-miR-467a-8p,mmu-miR-467a-9p,mmu-miR-467a-10p,mmu-miR-467a-11p,mmu-miR-467a-12p,mmu-miR-467a-13p,mmu-miR-467a-14p,mmu-miR-467a-15p,mmu-miR-467a-16p,mmu-miR-467a-17p,mmu-miR-467a-20p,mmu-miR-467a-21p,mmu-miR-467a-22p,mmu-miR-467a-23p,mmu-miR-467a-24p,mmu-miR-467a-25p,mmu-miR-467a-26p,mmu-miR-467a-27p,mmu-miR-467a-28p,mmu-miR-467a-29p,mmu-miR-467a-30p,mmu-miR-467a-31p,mmu-miR-467a-32p,mmu-miR-467a-33p,mmu-miR-467a-34p,mmu-miR-467a-35p,mmu-miR-467a-36p,mmu-miR-467a-37p,mmu-miR-467a-38p,mmu-miR-467a-39p,mmu-miR-467a-40p,mmu-miR-467a-41p,mmu-miR-467a-42p,mmu-miR-467a-43p,mmu-miR-467a-44p,mmu-miR-467a-45p,mmu-miR-467a-46p,mmu-miR-467a-47p,mmu-miR-467a-48p,mmu-miR-467a-49p,mmu-miR-467a-50p,mmu-miR-467a-51p,mmu-miR-467a-52p,mmu-miR-467a-53p,mmu-miR-467a-54p,mmu-miR-467a-55p,mmu-miR-467a-56p,mmu-miR-467a-57p,mmu-miR-467a-58p,mmu-miR-467a-59p,mmu-miR-467a-60p,mmu-miR-467a-61p,mmu-miR-467a-62p,mmu-miR-467a-63p,mmu-miR-467a-64p,mmu-miR-467a-65p,mmu-miR-467a-66p,mmu-miR-467a-67p,mmu-miR-467a-68p,mmu-miR-467a-69p,mmu-miR-467a-70p,mmu-miR-467a-71p,mmu-miR-467a-72p,mmu-miR-467a-73p,mmu-miR-467a-74p,mmu-miR-467a-75p,mmu-miR-467a-76p,mmu-miR-467a-77p,mmu-miR-467a-78p,mmu-miR-467a-79p,mmu-miR-467a-80p,mmu-miR-467a-81p,mmu-miR-467a-82p,mmu-miR-467a-83p,mmu-miR-467a-84p,mmu-miR-467a-85p,mmu-miR-467a-86p,mmu-miR-467a-87p,mmu-miR-467a-88p,mmu-miR-467a-89p,mmu-miR-467a-90p,mmu-miR-467a-91p,mmu-miR-467a-92p,mmu-miR-467a-93p,mmu-miR-467a-94p,mmu-miR-467a-95p,mmu-miR-467a-96p,mmu-miR-467a-97p,mmu-miR-467a-98p,mmu-miR-467a-99p,mmu-miR-467a-100p,mmu-miR-467a-101p,mmu-miR-467a-102p,mmu-miR-467a-103p,mmu-miR-467a-104p,mmu-miR-467a-105p,mmu-miR-467a-106p,mmu-miR-467a-107p,mmu-miR-467a-108p,mmu-miR-467a-109p,mmu-miR-467a-110p,mmu-miR-467a-111p,mmu-miR-467a-112p,mmu-miR-467a-113p,mmu-miR-467a-114p,mmu-miR-467a-115p,mmu-miR-467a-116p,mmu-miR-467a-117p,mmu-miR-467a-118p,mmu-miR-467a-119p,mmu-miR-467a-120p,mmu-miR-467a-121p,mmu-miR-467a-122p,mmu-miR-467a-123p,mmu-miR-467a-124p,mmu-miR-467a-125p,mmu-miR-467a-126p,mmu-miR-467a-127p,mmu-miR-467a-128p,mmu-miR-467a-129p,mmu-miR-467a-130p,mmu-miR-467a-131p,mmu-miR-

		467a-132p,mmu-miR-467a-133p,mmu-miR-467a-134p,mmu-miR-467a-135p,mmu-miR-467a-136p,mmu-miR-467a-137p,mmu-miR-467a-138p,mmu-miR-467a-139p,mmu-miR-467a-140p,mmu-miR-467a-141p,mmu-miR-467a-142p,mmu-miR-467a-143p,mmu-miR-467a-144p,mmu-miR-467a-145p,mmu-miR-467a-146p,mmu-miR-467a-147p,mmu-miR-467a-148p,mmu-miR-467a-149p,mmu-miR-467a-149p
MCAO 24h	mmu_circRNA_40203	NA
MCAO 24h	mmu_circRNA_31169	NA
MCAO 24h	mmu_circRNA_19077	mmu-miR-669k-5p， mmu-miR-1970
MCAO 24h	mmu_circRNA_45876	NA
MCAO 24h	mmu_circRNA_45047	mmu-miR-346-5p
MCAO 24h	mmu_circRNA_24344	mmu-miR-3093-3p
MCAO 24h	mmu_circRNA_22526	NA
MCAO 24h	mmu_circRNA_30884	NA
MCAO 24h	mmu_circRNA_010777	mmu-miR-3089-3p
MCAO 24h	mmu_circRNA_42273	mmu-miR-370-3p
MCAO 24h	mmu_circRNA_19073	NA
MCAO 24h	mmu_circRNA_36481	NA

MCAO 24h	mmu_circRNA_33184	mmu-miR-346-3p，mmu-miR-365-2-5p，mmu-miR-693-3p，mmu-miR-1956，mmu-miR-3075-3p，mmu-miR-3083-5p，mmu-miR-3096-3p，mmu-miR-5102
MCAO 24h	mmu_circRNA_37766	mmu-miR-3104-3p
MCAO 24h	mmu_circRNA_002520	NA
MCAO 24h	mmu_circRNA_27821	NA
MCAO 24h	mmu_circRNA_22663	mmu-miR-669c-3p，mmu-miR-676-5p，mmu-miR-466c-5p，mmu-miR-466e-5p，mmu-miR-1951，mmu-miR-3074-5p
MCAO 24h	mmu_circRNA_33499	mmu-miR-15a-3p，mmu-miR-706，mmu-miR-709，mmu-miR-717，mmu-miR-466i-5p，mmu-miR-466i-5p，mmu-miR-1187，mmu-miR-466k，mmu-miR-1935，mmu-miR-1946a，mmu-miR-1950，mmu-miR-669n，mmu-miR-3473c
MCAO 24h	mmu_circRNA_19355	NA
MCAO 24h	mmu_circRNA_22867	mmu-miR-346-3p,mmu-miR-377-5p
MCAO 24h	mmu_circRNA_003023	NA
MCAO 24h	mmu_circRNA_016017	NA
MCAO 24h	mmu_circRNA_34504	NA
MCAO 24h	mmu_circRNA_36263	NA
MCAO 24h	mmu_circRNA_004682	NA
MCAO 24h	mmu_circRNA_34491	mmu-miR-669p-3p
MCAO 24h	mmu_circRNA_19277	NA
MCAO 24h	mmu_circRNA_24446	mmu-miR-299-5p，mmu-miR-324-3p，mmu-miR-340-3p，mmu-miR-346-3p，mmu-miR-383-3p，mmu-miR-433-3p，mmu-miR-1198-5p
MCAO 24h	mmu_circRNA_24446	mmu-miR-299-5p，mmu-miR-324-3p，mmu-miR-340-3p，mmu-miR-346-3p，mmu-miR-383-3p，mmu-miR-433-3p，mmu-miR-1198-5p
MCAO 24h	mmu_circRNA_27348	NA
MCAO 24h	mmu_circRNA_41054	NA
MCAO 24h	mmu_circRNA_32884	NA

MCAO 24h	mmu_circRNA_43344	NA
MCAO 24h	mmu_circRNA_27614	NA
MCAO 24h	mmu_circRNA_23643	mmu-miR-3070b-3p,mmu-miR-5046
MCAO 24h	mmu_circRNA_26097	NA
MCAO 24h	mmu_circRNA_012978	NA
MCAO 24h	mmu_circRNA_37699	NA
MCAO 24h	mmu_circRNA_35175	NA
MCAO 24h	mmu_circRNA_39121	NA
MCAO 24h	mmu_circRNA_45921	mmu-miR-1962
MCAO 24h	mmu_circRNA_003795	NA
MCAO 24h	mmu_circRNA_002246	NA
MCAO 24h	mmu_circRNA_010803	NA
MCAO 24h	mmu_circRNA_40795	mmu-miR-760-3p,mmu-miR-669c-3p
MCAO 24h	mmu_circRNA_015506	NA
MCAO 24h	mmu_circRNA_41878	mmu-miR-361-3p,mmu-miR-3058-5p,mmu-miR-743a-5p

Supplementary Table S6. Gene Ontology analysis of the predicted circRNA-miRNA target genes.

Group	GO category	p-value	genes	miRNAs
MCAO 5 m vs SHAM	anatomical structure development	0	332	7
MCAO 5 m vs SHAM	intracellular	0	889	8
MCAO 5 m vs SHAM	biological_process	0	1463	8
MCAO 5 m vs SHAM	cell	0	1090	10
MCAO 5 m vs SHAM	molecular_function	1.72E-14	1092	5
MCAO 5 m vs SHAM	cell differentiation	4.45E-11	183	4
MCAO 5 m vs SHAM	organelle	0.0000382	591	4
MCAO 5 m vs SHAM	embyo development	0.000173629	46	3
MCAO 5 m vs SHAM	cellular_component	0.02759663	458	3
MCAO 3 h vs SHAM	molecular_function	0	1852	11
MCAO 3 h vs SHAM	anatomical structure development	0	431	10
MCAO 3 h vs SHAM	biological_process	0	2202	14
MCAO 3 h vs SHAM	intracellular	0	1470	17
MCAO 3 h vs SHAM	cell	0	1769	20
MCAO 3 h vs SHAM	cell differentiation	4.63E-09	239	6
MCAO 3 h vs SHAM	cellular_component	0.0000904	1005	5

MCAO 3 h vs SHAM	organelle	0.000145454	486	3
MCAO 3 h vs SHAM	ion binding	0.001951403	23	1
MCAO 3 h vs SHAM	embyo development	0.002613401	65	3
MCAO 24 h vs SHAM	cell	0	1913	15
MCAO 24 h vs SHAM	intracellular	7.35E-158	1626	15
MCAO 24 h vs SHAM	biological_process	5.97E-92	2767	15
MCAO 24 h vs SHAM	anatomical structure development	8.93E-66	594	14
MCAO 24 h vs SHAM	molecular_function	4.24E-47	2701	15
MCAO 24 h vs SHAM	cell differentiation	3.28E-28	426	14
MCAO 24 h vs SHAM	cellular_component	1.68E-22	2653	15
MCAO 24 h vs SHAM	organelle	7.52E-19	1364	15
MCAO 24 h vs SHAM	embyo development	3.24E-16	156	14
MCAO 24 h vs SHAM	anatomical structure formation involved in morphogenesis	8.44E-14	146	14
MCAO 24 h vs SHAM	ion binding	1.87E-11	842	15
MCAO 24 h vs SHAM	cellular protein modification process	1.17E-09	353	15
MCAO 24 h vs SHAM	cell morphogenesis	1.99E-08	115	14
MCAO 24 h vs SHAM	chromosome organization	0.00000216	94	13
MCAO 24 h vs SHAM	homeostatic process	0.00000226	147	14

MCAO 24 h vs SHAM	nucleic acid binding transcription factor activity	0.000231647	151	14
MCAO 24 h vs SHAM	developmental maturation	0.000535648	31	9
MCAO 24 h vs SHAM	biosynthetic process	0.000797789	525	15
MCAO 24 h vs SHAM	growth	0.00178368	73	14
MCAO 24 h vs SHAM	cell motility	0.005024911	94	14
MCAO 24 h vs SHAM	cytoplasmic membrane-bounded vesicle	0.032080963	82	14
MCAO 24 h vs SHAM	chromosome	0.04118051	102	14

Supplementary Table S7. KEGG pathway analysis of circRNAs in different time points of MCAO

Group	KEGG pathway	p-value	genes	miRNAs
Upregulation circRNAs in MCAO 5 m vs SHAM	Hippo signaling pathway	6.5497E-08	Fgf1, Nf2, Ppp2ca, Trp73, Yap1, Bmp2, Gli	mmu-miR-344d-3-5p,mmu-miR-344d-1-5
Upregulation circRNAs in MCAO 5 m vs SHAM	Biosynthesis of unsaturated fatty acids	3.62879E-05	Acot2	mmu-miR-344d-3-5p,mmu-miR-344d-1-5
Upregulation circRNAs in MCAO 5 m vs SHAM	Folate biosynthesis	0.003002272	Gch1	mmu-miR-344d-3-5p,mmu-miR-344d-1-5
Upregulation circRNAs in MCAO 5 m vs SHAM	Vitamin B6 metabolism	0.030929924	Pdxk	mmu-miR-344d-3-5p,mmu-miR-344d-1-5
Downregulation circRNAs in MCAO 5 m vs SHAM	Mucin type O-Glycan biosynthesis	1.8699E-05	St6galnac1, Galnt5, Galnt7	mmu-miR-5623-5p
Downregulation circRNAs in MCAO 5 m vs SHAM	Cytokine-cytokine receptor interaction	0.00106912	Gm13304, Il1r1, Tnfrsf12a, Flt4, Gm21541, Il6st	mmu-miR-30c-1-3p,mmu-miR-1962
Downregulation circRNAs in MCAO 5 m vs SHAM	Gap junction	0.001832478	Gjd2, Mapk3, Csnk1d, Gja1, Prkg1	mmu-miR-5623-5p
Downregulation circRNAs in MCAO 5 m vs SHAM	Metabolism of xenobiotics by cytochrome P450	0.003354516	Ugt2b5, Mgst3, Gstm2, Ephx1, Ugt2b37	mmu-miR-1962,mmu-miR-361-3p
Upregulation circRNAs in MCAO 3 h vs SHAM	Thyroid hormone synthesis	0.002103258	Prkca,Tg,Plcb2	mmu-miR-3089-3p, mmu-miR-3093-3p,mmu-miR-484
Downregulation circRNAs in MCAO 3 h vs SHAM	Glycosphingolipid biosynthesis - lacto and neolacto series	2.78494E-10	Gcnt2, Fut9,Fut1	mmu-miR-199a-5p,mmu-miR-199b-5p,mmu-miR-770-3p
Downregulation circRNAs in MCAO 3 h vs SHAM	ECM-receptor interaction	8.102E-05	Col6a3, Itgb3, Lamc2, Thbs2, Hmmr, Itgav, Tnxb, Lamc1	mmu-miR-324-3p,mmu-miR-667-5p,mmu-miR-5113,mmu-miR-141-5p
Downregulation circRNAs in MCAO 3 h vs SHAM	Metabolism of xenobiotics by cytochrome P450	0.007447632	Cyp1a1, Gstm5, Ugt2b5, Mgst3, Gstm2, Ephx1, Ugt2b37	mmu-miR-361-3p,mmu-miR-770-3p,mmu-miR-1962
Upregulation circRNAs in MCAO 24 h vs SHAM	Fatty acid degradation	3.14963E-06	Hadha, Acox1, Ehhadh	mmu-miR-669c-3p,mmu-miR-669k-5p
Upregulation circRNAs in MCAO 24 h vs SHAM	Fatty acid metabolism	0.00056093	Hadha, Elovl5, Acox1, Ehhadh, Ppt1	mmu-miR-346-5p,mmu-miR-669c-3p,mmu-miR-669k-5p
Upregulation circRNAs in MCAO 24 h vs SHAM	Type II diabetes mellitus	0.000585123	Pik3r1, Pik3r3, Irs4, Ikbkb, Cacna1b, Insr, Irs1, Mapk9, Prkcd, Hkdc1, Pik3cd, Mapk8, Hk2, Adipoq	mmu-miR-1970,mmu-miR-669c-3p,mmu-miR-669b-3p,mmu-miR-297b-3p,mmu-miR-3093-3p,mmu-miR-193a-5p,mmu-miR-346-5p,mmu-miR-677-3p
Upregulation circRNAs in MCAO 24 h vs SHAM	Fatty acid elongation	0.001239343	Hadha, Elovl5, Elovl1, Ppt1	mmu-miR-346-5p,mmu-miR-669c-3p,mmu-miR-669k-5p
Upregulation circRNAs in MCAO 24 h vs SHAM	Phosphatidylinositol signaling system	0.00477227	Inpp1, Pik3r1, Pik3r3, Itpr2, Pik3c2g, Inpp5j, Pik3c3, Cds2, Prkca, Plcb1, Itpk1, Pten, Pik3cd, Plcb2	mmu-miR-669k-5p,mmu-miR-669c-3p,mmu-miR-346-5p,mmu-miR-193b-5p,mmu-miR-3093-3p,mmu-miR-1970
Upregulation circRNAs in MCAO 24 h vs SHAM	mTOR signaling pathway	0.020401333	Pik3r1, Pik3r3, Eif4e2, Ikbkb, Ulk3, Rps6ka6, Irs1, Vegfa, Prkaa1, Eif4ebp1, Prkca, Rraga, Pten, Pik3cd, Eif4e1b	mmu-miR-677-3p,mmu-miR-669c-3p,mmu-miR-193b-5p,mmu-miR-346-5p,mmu-miR-3093-3p,mmu-miR-669b-3p
Upregulation circRNAs in MCAO 24 h vs SHAM	TNF signaling pathway	0.025829681	Mapk14, Nfkbia, Pik3r1, Pik3r3, Ikbkb, Lif, Junb, Creb5, Casp3, Tnfrsf1b, Il18r1, Mapk9, Sele, Tab3,	mmu-miR-346-5p,mmu-miR-669c-3p,mmu-miR-3093-

			Tradd, Ripk1, Pik3cd, Mapk8	3p,mmu-miR-677-3p,mmu-miR-669b-3p
Upregulation circRNAs in MCAO 24 h vs SHAM	Adipocytokine signaling pathway	0.025829681	Nfkbia, Irs4, Ikbkb, Camkk2, Irs1, Tnfrsf1b, Prkcq, Prkaa1, Mapk9, Ppargc1a, Prkag2, Tradd, Pck1, Mapk8, Adipoq	mmu-miR-1970,mmu-miR-669c-3p,mmu-miR-193a-5p,mmu-miR-346-5p,mmu-miR-3093-3p,mmu-miR-677-3p,mmu-miR-669b-3p
Upregulation circRNAs in MCAO 24 h vs SHAM	Thyroid hormone synthesis	0.048874445	Itpr2, Creb5, Adcy1, Prkca, Tg, Plcb1, Plcb2	mmu-miR-3093-3p,mmu-miR-346-5p,mmu-miR-669c-3p,mmu-miR-193b-5p,mmu-miR-669k-5p,mmu-miR-677-3p
Downregulation circRNAs in MCAO 24 h vs SHAM	Systemic lupus erythematosus	1.19826E-06	Tnf, Hist3h2ba, Hist3h2a, Hist2h3c2, Hist2h3c1, Hist1h3d, Hist1h3b, Hist1h3a, Hist1h2bj, Hist1h2bg, Hist1h2bf, Hist1h2ap, Hist1h2ao, Hist1h2ag, Hist1h2ae, Hist1h2ad, Hist1h2ab, H2bfm, Cd28	mmu-miR-760-3p, mmu-miR-1962
Downregulation circRNAs in MCAO 24 h vs SHAM	Alcoholism	0.000446567	Ppp1r1b, Hist3h2ba, Hist3h2a, Hist2h3c2, Hist2h3c1, Hist1h3d, Hist1h3b, Hist1h3a, Hist1h2bj, Hist1h2bg, Hist1h2bf, Hist1h2ap, Hist1h2ao, Hist1h2ag, Hist1h2ae, Hist1h2ad, Hist1h2ab, H2bfm	mmu-miR-760-3p
Downregulation circRNAs in MCAO 24 h vs SHAM	Other types of O-glycan biosynthesis	0.000446567	Ugt2b5, Hsd17b6, Ugt2b37	mmu-miR-1962,mmu-miR-760-3p
Downregulation circRNAs in MCAO 24 h vs SHAM	Steroid hormone biosynthesis	0.008920821	Ugt2b5, Mgst3, Ugt2b37	mmu-miR-1962,mmu-miR-760-3p
Downregulation circRNAs in MCAO 24 h vs SHAM	Metabolism of xenobiotics by cytochrome P450	0.036022081	Ugt2b5, Mgst3, Ugt2b37	mmu-miR-1962
Downregulation circRNAs in MCAO 24 h vs SHAM	Inositol phosphate metabolism	0.036022081	Pi4k2a, Synj1, Plcg2, Pip5kl1, Impa2	mmu-miR-1962,mmu-miR-760-3p
Downregulation circRNAs in MCAO 24 h vs SHAM	Cytokine-cytokine receptor interaction	0.036022081	Cxcl9, Gm13304, Il2ra, Flt4, Gm21541, Il6st, Ngfr, Tnf, Flt3	mmu-miR-1962,mmu-miR-760-3p
Downregulation circRNAs in MCAO 24 h vs SHAM	Phosphatidylinositol signaling system	0.036022081	Pi4k2a, Cds2, Synj1, Plcg2, Impa2	mmu-miR-1962,mmu-miR-760-3p

Supplementary Table S8. Baseline characteristics of stroke patients and control subjects.

Demographic and clinicopathological variables	Ischemic stroke n (%)	Control subjects n (%)
Total, n	8	8
Age (Mean±SD)	55.4±12.3	54.11±10.1
Male	7(77.8%)	7(77.8%)
**Vascular risk factors, n (%)**		
Hypertension	3(33.3%)	2(22.2%)
Smoking history	4(44.4%)	2(22.2%)
Hypercholesterolemia	0(0.0%)	0(0.0%)
Diabetes mellitus	0(0.0%)	0(0.0%)
Previous TIA/stroke/MI	0(0.0%)	0(0.0%)
Atrial fibrillation	2(22.2%)	2(22.2%)
Chronic kidney disease	0(0.0%)	1(11.1%)
Family history	0(0.0%)	0(0.0%)
**Laboratory parameters, mean±SD**		
Glucose [mmol/l]	5.53±1.1	4.99±0.4
Creatinine [mg/dl]	207.9±394.5*	73.37±9.6
hs-CRP [mg/dl]	7.82±12.2*	3.29±0.9
D-dimer [ug/mL]	865.00±922.8*	289.00±70.9
Total Cholesterol [mg/dl]	9.95±10.8*	4.70±0.8
Triglycerides [mg/dl]*	1.13±0.4	1.77±0.7

LDL [mg/dl]	2.49±1.0	2.85±0.4
HDL [mg/dl]	1.19±0.6	1.62±.1.2
AST [U/l]*	22.00±6.2	18.67±6.1
ALT [U/l]	24.78±17.4	17.89±4.6
Uric acid [umol/L]	320.20±88.7	402.1±93.6
Albumin [g/L]	37.78±2.7	40.7±1.6
**Medication, n (%)**		
Statins	0(0.0%)	0(0.0%)
ACE I / ARBs	0(0.0%)	2(22.2%)
Beta-Blockers	1(11.1%)	0(0.0%)
Calcium channel blockers	2(22.2%)	0(0.0%)
Anticoagulant	0(0.0%)	0(0.0%)
Antiplatelet therapy	0(0.0%)	1(11.1%)
**Imaging findings, n (%)**		
ICA occlusion	1(11.1%)	n/a
MCA occlusion	7(77.8%)	n/a
Other occlusion	1(11.1%)	n/a
DWI lesion volume at baseline, mean±SD [mL]	32.9±36.3*	n/a
ΔT, mean±SD [minutes]	178.80±68.7	n/a
**Stroke subtype, n (%)**		n/a
CE	2(22.2%)	n/a
LAA	4(44.4%)	n/a
SVO	3(33.3%)	n/a

Other etiology	0(0.0%)	n/a
**Score, mean±SD**		n/a
NIHSS	8.67±7.0	n/a
mRS	1.89±1.85	n/a
ASPECT	7.33±2.11	n/a

* s.e.m >Mean,as for one of the patients was massive cerebral infarction, the ICA occlusion;

Abbreviation: SD, standard deviation; TIA, transient ischemic attack; MI, myocardial infarction; AST,aspartate transaminase; ALT, alanine transaminase; LDL, low-density lipoprotein; HDL, high-density lipoprotein; ACE I, angiotensin-converting enzyme inhibitor; ARBs, angiotensin II receptor blockers; ΔT, time from symptom onset until hospital arrival; n/a, not available; ASPECT,Alberta Stroke Program Early CT Score, 8of patients were obseved by MRI, one of patients was observed by CT in hyperacute phase without significant damage.

Supplementary Table S9. Ct values of RT-qPCR reactions

Figure3		β-actin	β-actin	β-actin	circ_35605	circ_35605	circ_35605
Figure3	SHAM	19.89	19.98	19.68	34.06	34.88	34.76
Figure3	SHAM	19.24	19.81	19.88	34.11	35.75	35.33
Figure3	SHAM	20.14	20.04	20.79	34.44	34.66	34.82
Figure3	MCAO 5m	20.13	20.05	20.07	34.05	34.02	34.24
Figure3	MCAO 5m	19.66	19.21	19.33	34.13	34.17	33.99
Figure3	MCAO 5m	20.66	20.38	20.5	33.72	33.6	33.79
Figure3		β-actin	β-actin	β-actin	circ 26021	circ 26021	circ 26021
Figure3	SHAM	19.28	19.33	19.21	33.01	33.68	32.58
Figure3	SHAM	19.64	19.47	19.6	32.53	32.41	32.03
Figure3	SHAM	19.23	20.08	19.63	32.02	32.84	32.91
Figure3	MCAO 5m	20.82	20.92	20.75	32.93	32.67	32.25
Figure3	MCAO 5m	20.3	20.21	20.2	32.6	32.36	32.34
Figure3	MCAO 5m	21.07	20.86	20.41	32.12	32.59	32.55
Figure3		β-actin	β-actin	β-actin	circ_32190	circ_32190	circ_32190
Figure3	SHAM	19.15	19.11	19.8	30.82	30.45	30.2
Figure3	SHAM	19.26	19.28	19.24	31.03	31.02	30.03
Figure3	SHAM	19.93	19.78	19.63	30.51	30.59	30.58
Figure3	MCAO 5m	19.8	19.91	20.08	28.74	28.94	28.78
Figure3	MCAO 5m	19.92	20.01	19.94	29.56	29.95	29.59
Figure3	MCAO 5m	20.7	20.36	20.41	29.78	29.57	29.77
Figure3		β-actin	β-actin	β-actin	circ 41878	circ 41878	circ 41878
Figure3	SHAM	19.05	19.01	19.03	28.04	28.02	28.78
Figure3	SHAM	19.16	19.18	19.14	28.56	28.95	28.5
Figure3	SHAM	19.63	19.68	19.63	29.08	29.07	28.77
Figure3	MCAO 5m	20.8	20.79	20.07	32.75	32.55	32.9
Figure3	MCAO 5m	20.02	20.11	20.04	31.49	31.02	31.21
Figure3	MCAO 5m	20.7	20.43	20.4	31.41	31.59	31.53
Figure3		β-actin	β-actin	β-actin	circ_34491	circ_34491	circ_34491
Figure3	SHAM	19.05	19.01	19.03	29.64	29.54	28.68
Figure3	SHAM	19.16	19.18	19.14	29.46	29.98	29.32
Figure3	SHAM	19.63	19.68	19.63	29.76	29.77	29.69
Figure3	MCAO 5m	20.8	20.79	20.07	32.78	32.75	32.75
Figure3	MCAO 5m	20.02	20.11	20.04	32.03	31.93	32.06
Figure3	MCAO 5m	20.7	20.43	20.4	31.54	30.92	30.88
Figure3		β-actin	β-actin	β-actin	circ 003795	circ 003795	circ 003795
Figure3	SHAM	19.28	19.33	19.21	32.93	32.67	32.25
Figure3	SHAM	19.64	19.47	19.6	32.6	32.36	32.34
Figure3	SHAM	19.23	20.08	19.63	32.12	32.59	32.55
Figure3	MCAO 5m	20.82	20.92	20.75	34.01	34.68	34.58
Figure3	MCAO 5m	20.3	20.21	20.2	34.93	34.41	34.85
Figure3	MCAO 5m	21.07	20.86	20.41	34.32	34.93	34.21
Figure3		β-actin	β-actin	β-actin	circ_40203	circ_40203	circ_40203
Figure3	SHAM	18.85	18.91	18.8	33.43	34.66	35.68
Figure3	SHAM	18.66	18.89	18.98	33.95	35.35	34.32
Figure3	SHAM	18.93	18.79	18.86	34.57	34.72	35.01
Figure3	MCAO 3h	19.38	19.9	20.01	35.01	34.86	34.94
Figure3	MCAO 3h	19.37	19.22	19.59	34.7	35.45	34.54
Figure3	MCAO 3h	20.55	19.08	19.39	35.11	34.32	34.89
Figure3		β-actin	β-actin	β-actin	circ_45876	circ_45876	circ_45876
Figure3	SHAM	19.58	19.82	19.91	32.93	33.07	32.95
Figure3	SHAM	19.65	19.74	19.92	32.6	32.36	32.34
Figure3	SHAM	20	19.83	19.83	32.05	32.48	32.39
Figure3	MCAO 3h	20.04	20.09	20.09	30.94	30.91	30.78
Figure3	MCAO 3h	19.68	19.66	19.72	30.68	31.05	31.09
Figure3	MCAO 3h	20.04	20.05	19.63	29.98	29.97	30.04
Figure3		β-actin	β-actin	β-actin	circ_24344	circ_24344	circ_24344
Figure3	SHAM	19.58	19.82	19.91	33.01	33.18	33.28
Figure3	SHAM	19.65	19.74	19.92	33.93	32.21	32.43
Figure3	SHAM	20	19.83	19.83	32.92	32.84	32.91
Figure3	MCAO 3h	20.04	20.09	20.09	32.63	32.67	32.15
Figure3	MCAO 3h	19.68	19.66	19.72	32.02	32.12	32.04
Figure3	MCAO 3h	20.04	20.05	19.63	31.38	31.99	32.18
Figure3		β-actin	β-actin	β-actin	circ_37699	circ_37699	circ_37699
Figure3	SHAM	20.63	20.01	19.55	30.11	30.01	30.68
Figure3	SHAM	20.08	19.6	19.15	30.22	30.64	30.41
Figure3	SHAM	19.54	19.72	19.63	30.44	30.99	30.82
Figure3	MCAO 3h	20.32	20.04	21.02	32.06	32.11	31.64
Figure3	MCAO 3h	20.1	20	21.03	31.29	31.73	32.08
Figure3	MCAO 3h	21.57	20.95	20.98	31.82	31.55	32.01
Figure3		β-actin	β-actin	β-actin	circ_38328	circ_38328	circ_38328
Figure3	SHAM	20.63	20.01	19.55	30.01	30.03	30.22
Figure3	SHAM	20.08	19.6	19.15	30.1	30.34	30.43
Figure3	SHAM	19.54	19.72	19.63	30.14	30.98	30.83
Figure3	MCAO 3h	20.32	20.04	21.02	32.3	32.63	32.24

Figure3	MCAO 3h	20.1	20	21.03	32.29	32.73	32.23
Figure3	MCAO 3h	21.57	20.95	20.98	32.72	32.95	32.51
Figure3		β-actin	β-actin	β-actin	circ_26316	circ_26316	circ_26316
Figure3	SHAM	20.63	20.01	19.55	30.77	30.78	30.47
Figure3	SHAM	20.08	19.6	19.15	30.83	30.33	30.76
Figure3	SHAM	19.54	19.72	19.63	30.24	30.76	30.66
Figure3	MCAO 3h	20.32	20.04	21.02	32.33	32.73	32.34
Figure3	MCAO 3h	20.1	20	21.03	32.69	32.11	32.03
Figure3	MCAO 3h	21.57	20.95	20.98	32.62	32.77	32.96
Figure3		β-actin	β-actin	β-actin	circ_43956	circ_43956	circ_43956
Figure3	SHAM	18.85	18.91	18.8	33.53	34.51	35.72
Figure3	SHAM	18.66	18.89	18.98	34.05	35.55	34.23
Figure3	SHAM	18.93	18.79	18.86	34.57	34.77	35.07
Figure3	MCAO 24h	19.38	19.9	20.01	32.05	33.45	32.01
Figure3	MCAO 24h	19.37	19.22	19.59	33.774	33.45	33.46
Figure3	MCAO 24h	20.55	19.08	19.39	33.71	33.21	33.29
Figure3		β-actin	β-actin	β-actin	circ_37286	circ_37286	circ_37286
Figure3	SHAM	19.58	19.82	19.91	31.82	31.32	31.53
Figure3	SHAM	19.65	19.74	19.92	31.95	31.43	31.3
Figure3	SHAM	20	19.83	19.83	31.98	31.69	30.84
Figure3	MCAO 24h	20.04	20.09	20.09	30.89	31.23	31.38
Figure3	MCAO 24h	19.68	19.66	19.72	31.01	30.91	31.08
Figure3	MCAO 24h	20.04	20.05	19.63	31.1	31.25	30.92
Figure3		β-actin	β-actin	β-actin	circ_40203	circ_40203	circ_40203
Figure3	SHAM	18.85	18.91	18.8	33.23	34.64	35.88
Figure3	SHAM	18.66	18.89	18.98	34.05	34.55	34.52
Figure3	SHAM	18.93	18.79	18.86	34.57	34.72	34.71
Figure3	MCAO 24h	19.38	19.9	20.01	35.01	34.98	34.75
Figure3	MCAO 24h	19.37	19.22	19.59	35.75	34.69	34.73
Figure3	MCAO 24h	20.55	19.08	19.39	35.06	34.58	34.88
Figure3		β-actin	β-actin	β-actin	circ_41878	circ_41878	circ_41878
Figure3	SHAM	18.75	19.5	19.23	28.04	28.02	28.74
Figure3	SHAM	19.26	19.28	19.34	28.46	28.85	28.82
Figure3	SHAM	19.13	19.22	19.79	29.29	28.77	28.97
Figure3	MCAO 24h	20.05	20.07	20.07	32.27	32.17	33.24
Figure3	MCAO 24h	19.95	19.89	19.94	31.93	32.01	31.89
Figure3	MCAO 24h	20.02	20.03	19.98	31.79	32.03	32.01
Figure3		β-actin	β-actin	β-actin	circ_40795	circ_40795	circ_40795
Figure3	SHAM	18.75	19.5	19.23	28.11	28.13	28.08
Figure3	SHAM	19.26	19.28	19.34	28.38	28.95	28.88
Figure3	SHAM	19.13	19.22	19.79	29.31	28.89	28.87
Figure3	MCAO 24h	20.05	20.07	20.07	31.27	31.07	30.82
Figure3	MCAO 24h	19.95	19.89	19.94	30.82	30.68	30.02
Figure3	MCAO 24h	20.02	20.03	19.98	31.02	30.13	30.42
Figure3		β-actin	β-actin	β-actin	circ_003795	circ_003795	circ_003795
Figure3	SHAM	18.75	19.5	19.23	29.09	29.45	30.08
Figure3	SHAM	19.26	19.28	19.34	29.38	29.97	29.68
Figure3	SHAM	19.13	19.22	19.79	29.49	30.13	30.34
Figure3	MCAO 24h	20.05	20.07	20.07	32.33	32.82	32.65
Figure3	MCAO 24h	19.95	19.89	19.94	32.93	32.95	32.59
Figure3	MCAO 24h	20.02	20.03	19.98	32.22	32.84	32.52
Figure 7B		β-actin	β-actin	β-actin	has_circ_0039457	has_circ_0039457	has_circ_0039457
Figure 7B	CS1	26.38	26.35	26.38	29.58	29.77	29.66
Figure 7B	IS1	24.96	24.9	24.82	30.02	29.75	29.56
Figure 7B	CS2	30.32	30.26	30.25	28.22	28.1	28.12
Figure 7B	IS2	27.5	27.75	27.62	28.67	28.67	28.74
Figure 7B	CS3	29.52	29.33	29.5	27.78	28.05	27.57
Figure 7B	IS3	28.12	27.92	28	29.51	29.38	29.33
Figure 7B	CS4	24.82	24.91	24.97	29.55	30.25	29.17
Figure 7B	IS4	26.2	26.16	26.04	30.31	30.69	31.53
Figure 7B	CS5	29.33	28.74	28.68	28.3	28.88	28.56
Figure 7B	IS5	26.41	26.37	26.25	29.36	28.97	28.93
Figure 7B	CS6	26.9	26.66	26.56	36.27	35.13	35.68
Figure 7B	IS6	24.05	24	24.03	34.92	34.06	35.7
Figure 7B	CS7	28.03	28.02	28.05	33.99	33.72	33.98
Figure 7B	IS7	29.18	29.25	29.31	33.48	34.18	33.82
Figure 7B	CS8	31.71	32.19	31.79	29.12	28.81	28.68
Figure 7B	IS8	27.56	27.75	27.64	28.86	28.84	28.75
Figure 7B		β-actin	β-actin	β-actin	has_circ_0090002	has_circ_0090002	has_circ_0090002
Figure 7B	CS1	26.38	26.35	26.38	19.84	21.01	21.44
Figure 7B	IS1	24.96	24.9	24.82	25.72	25.73	25.38
Figure 7B	CS2	30.32	30.26	30.25	20.04	19.88	19.9
Figure 7B	IS2	27.5	27.75	27.62	25.9	25.07	24.64
Figure 7B	CS3	29.52	29.33	29.5	19.18	19.9	20.34
Figure 7B	IS3	28.12	27.92	28	20.14	20.91	19.33
Figure 7B	CS4	24.82	24.91	24.97	22.86	22.8	21.99
Figure 7B	IS4	27.92	27.5	27.62	27.35	27.79	28.33

Figure 7B	CS5	29.33	28.74	28.68	23.93	23.64	23.68
Figure 7B	IS5	26.41	26.37	26.25	26.42	26.45	26
Figure 7B	CS6	26.9	26.66	26.56	35.98	35.61	34.87
Figure 7B	IS6	27.84	27.64	27.66	37.01	36.07	37.51
Figure 7B	CS7	28.03	28.02	28.05	30.63	30.51	30.5
Figure 7B	IS7	29.18	29.25	29.31	33.39	33.19	33.37
Figure 7B	CS8	31.71	32.19	31.79	24.81	25.16	25.33
Figure 7B	IS8	27.56	27.75	27.64	25.45	25.81	25.65
Supplementary Figure S3		β- actin	β- actin	β- actin	circ_42362	circ_42362	circ_42362
Supplementary Figure S3	SHAM	17.05	17.71	16.8	28.1	28.05	29.09
Supplementary Figure S3	SHAM	17.12	17.68	17.87	28.38	28.88	29.08
Supplementary Figure S3	SHAM	17.08	18.22	18.82	28.99	28.91	28.97
Supplementary Figure S3	MCAO 5m	19.05	19.89	19.97	28.39	28.13	28.41
Supplementary Figure S3	MCAO 5m	19.95	19.32	19.24	28.12	28.81	29.36
Supplementary Figure S3	MCAO 5m	18.89	18.7	19.02	29.09	28.02	28.99
Supplementary Figure S3	SHAM	18.81	17.79	18.78	30.09	29.59	30.12
Supplementary Figure S3	SHAM	18.28	18.9	18.88	30.1	30.13	30.15
Supplementary Figure S3	SHAM	19.01	18.54	18.67	30.22	30.53	30.45
Supplementary Figure S3	MCAO 3h	20.07	19.97	19.66	31.08	31.02	31.09
Supplementary Figure S3	MCAO 3h	20.02	20.1	19.94	31.25	30.48	30.18
Supplementary Figure S3	MCAO 3h	19.99	20.01	20.02	30.88	31	31.01
Supplementary Figure S3	SHAM	17.79	17.77	17.97	29.55	29.12	29.72
Supplementary Figure S3	SHAM	17.35	17.6	17.77	28.19	29.09	29.29
Supplementary Figure S3	SHAM	17.78	17.82	18.78	28.99	28.91	28.97
Supplementary Figure S3	MCAO 24h	20.01	20.23	20.11	30.83	31.02	30.35
Supplementary Figure S3	MCAO 24h	20.03	20.03	20.06	30.63	30.92	30.43
Supplementary Figure S3	MCAO 24h	20.13	20.08	20.68	30.73	30.8	30.87
Supplementary Figure S3		β- actin	β- actin	β- actin	m42362	m42362	m42362
Supplementary Figure S3	SHAM	17.05	17.71	16.8	29.13	28.99	29.55
Supplementary Figure S3	SHAM	17.12	17.68	17.87	28.17	28.45	28.12
Supplementary Figure S3	SHAM	17.08	18.22	18.82	28.03	28.01	28.04
Supplementary Figure S3	MCAO 5m	19.05	19.89	19.97	31.27	30.83	30.82
Supplementary Figure S3	MCAO 5m	19.95	19.32	19.24	31.11	31.02	29.78
Supplementary Figure S3	MCAO 5m	18.89	18.7	19.02	30.62	30.61	30.32
Supplementary Figure S3	SHAM	18.81	17.79	18.78	29.59	30.09	29.53
Supplementary Figure S3	SHAM	18.28	18.9	18.88	29.93	29.78	29.89
Supplementary Figure S3	SHAM	19.01	18.54	18.67	30.01	30.12	29.98
Supplementary Figure S3	MCAO 3h	20.07	19.97	19.66	31.13	31.02	31.11
Supplementary Figure S3	MCAO 3h	20.02	20.1	19.94	30.98	31.17	31.13
Supplementary Figure S3	MCAO 3h	19.99	20.01	20.02	31.03	31.01	31.05
Supplementary Figure S3	SHAM	17.79	17.77	17.97	28.5	28.89	28.46
Supplementary Figure S3	SHAM	17.35	17.6	17.77	29.01	29.03	29.09
Supplementary Figure S3	SHAM	17.78	17.82	18.78	28.97	28.91	28.39
Supplementary Figure S3	MCAO 24h	20.01	20.23	20.11	33.12	33.06	33.43
Supplementary Figure S3	MCAO 24h	20.03	20.03	20.06	33.63	33.48	33.89
Supplementary Figure S3	MCAO 24h	20.13	20.08	20.68	33.34	32.68	33.59
Supplementary Figure S3		β- actin	β- actin	β- actin	circ_26316	circ_26316	circ_26316
Supplementary Figure S3	SHAM	19.82	19.91	19.78	29.88	29.01	29.14
Supplementary Figure S3	SHAM	19.85	20.01	19.72	30.01	29.26	29.71
Supplementary Figure S3	SHAM	19.98	19.81	19.77	29.03	29.35	29.48
Supplementary Figure S3	MCAO 5m	20.03	19.92	19.91	31.77	31.03	31.11
Supplementary Figure S3	MCAO 5m	19.49	21	19.79	31.43	31.78	30.17
Supplementary Figure S3	MCAO 5m	20.12	20.05	20.02	31.62	31.21	31.03
Supplementary Figure S3	SHAM	19.21	19.39	19.35	NA	46.67	NA
Supplementary Figure S3	SHAM	19.33	19.8	19.72	45.9	NA	48.9
Supplementary Figure S3	SHAM	19.63	19.49	19.55	NA	NA	NA
Supplementary Figure S3	MCAO 3h	19.98	20.12	20.2	NA	NA	NA
Supplementary Figure S3	MCAO 3h	20.03	20.04	20.04	NA	47.7	NA
Supplementary Figure S3	MCAO 3h	19.77	19.93	20.02	NA	NA	NA
Supplementary Figure S3	SHAM	19.12	19.31	19.08	28.05	28.78	28.83
Supplementary Figure S3	SHAM	19.04	19.03	19.03	28.01	28.38	28.61
Supplementary Figure S3	SHAM	19.77	19.46	19.84	28.08	28.79	28.89
Supplementary Figure S3	MCAO 24h	20.01	20.23	20.11	30.02	30.06	30.61
Supplementary Figure S3	MCAO 24h	20.03	20.03	20.06	30.13	30.52	30.91
Supplementary Figure S3	MCAO 24h	20.13	20.08	20.68	30.13	30.68	30.29
Supplementary Figure S3		β- actin	β- actin	β- actin	m26316	m26316	m26316
Supplementary Figure S3	SHAM	19.82	19.91	19.78	27.24	26.6	27.02
Supplementary Figure S3	SHAM	19.85	20.01	19.72	27.23	27.45	26.67
Supplementary Figure S3	SHAM	19.98	19.81	19.77	27.04	27.05	27.19
Supplementary Figure S3	MCAO 5m	20.03	19.92	19.91	27.43	26.65	27.39
Supplementary Figure S3	MCAO 5m	19.49	21	19.79	27.11	27.02	27.24
Supplementary Figure S3	MCAO 5m	20.12	20.05	20.02	27.13	27.12	27.13
Supplementary Figure S3	SHAM	19.21	19.39	19.35	NA	NA	NA
Supplementary Figure S3	SHAM	19.33	19.8	19.72	NA	NA	47.8
Supplementary Figure S3	SHAM	19.63	19.49	19.55	NA	NA	NA
Supplementary Figure S3	MCAO 3h	19.98	20.12	20.2	NA	NA	NA
Supplementary Figure S3	MCAO 3h	20.03	20.04	20.04	NA	NA	NA
Supplementary Figure S3	MCAO 3h	19.77	19.93	20.02	NA	NA	NA
Supplementary Figure S3	SHAM	19.12	19.31	19.08	28.12	28.72	28.73
Supplementary Figure S3	SHAM	19.04	19.03	19.03	28.26	28.38	28.61
Supplementary Figure S3	SHAM	19.77	19.46	19.84	28.08	28.79	28.19

Supplementary Figure S3	MCAO 24h	20.01	20.23	20.11	29.52	29.29	29.21
Supplementary Figure S3	MCAO 24h	20.03	20.03	20.06	29.23	29.24	28.85
Supplementary Figure S3	MCAO 24h	20.13	20.08	20.68	29.35	29.68	28.72
Supplementary Figure S3		β-actin	β-actin	β-actin	circ_43344	circ_43344	circ_43344
Supplementary Figure S3	SHAM	18.54	18.08	18.34	28.21	28.67	28.23
Supplementary Figure S3	SHAM	18.11	18.67	18.73	28.07	28.51	28.61
Supplementary Figure S3	SHAM	18.09	18.66	18.76	28.11	28.09	28.06
Supplementary Figure S3	MCAO 5m	18.79	18.97	18.32	31.52	31.94	31.22
Supplementary Figure S3	MCAO 5m	18.98	18.65	18.77	30.11	30.33	31.05
Supplementary Figure S3	MCAO 5m	19.38	19.74	19.24	31.98	31.81	31.45
Supplementary Figure S3	SHAM	18.05	18.28	18.84	28.13	28.92	28.42
Supplementary Figure S3	SHAM	18.23	18.98	18.38	28.07	28.79	28.13
Supplementary Figure S3	SHAM	18.12	18.25	18.22	28.33	28.09	28.74
Supplementary Figure S3	MCAO 3h	19.05	18.23	18.17	29.77	29.77	29.48
Supplementary Figure S3	MCAO 3h	18.87	18.21	18.33	30.11	29.65	29.9
Supplementary Figure S3	MCAO 3h	18.45	18.63	18.72	29.98	29.51	29.05
Supplementary Figure S3	SHAM	18.89	18.88	18.98	28.5	28.89	28.96
Supplementary Figure S3	SHAM	18.35	18.6	18.88	28.21	29.23	29.09
Supplementary Figure S3	SHAM	18.88	18.82	18.88	28.97	28.91	28.82
Supplementary Figure S3	MCAO 24h	20.01	20.23	20.11	30.52	30.51	30.93
Supplementary Figure S3	MCAO 24h	20.03	20.03	20.06	30.83	30.48	30.91
Supplementary Figure S3	MCAO 24h	20.13	20.08	20.68	30.34	32.68	30.59
Supplementary Figure S3		β-actin	β-actin	β-actin	m43344	m43344	m43344
Supplementary Figure S3	SHAM	18.54	18.08	18.34	28.28	27.67	28.33
Supplementary Figure S3	SHAM	18.11	18.67	18.73	28.07	28.51	29.01
Supplementary Figure S3	SHAM	18.09	18.66	18.76	26.11	26.69	26.76
Supplementary Figure S3	MCAO 5m	18.79	18.97	18.32	28.13	28.01	27.66
Supplementary Figure S3	MCAO 5m	18.98	18.65	18.77	28.16	28.53	28.45
Supplementary Figure S3	MCAO 5m	19.38	19.74	19.24	28.18	27.53	27.65
Supplementary Figure S3	SHAM	18.05	18.28	18.84	28.13	28.92	28.42
Supplementary Figure S3	SHAM	18.23	18.98	18.38	27.07	27.87	27.17
Supplementary Figure S3	SHAM	18.12	18.25	18.22	28.33	28.09	28.74
Supplementary Figure S3	MCAO 3h	19.05	18.23	18.17	29.78	29.59	30.08
Supplementary Figure S3	MCAO 3h	18.87	18.21	18.33	29.11	28.75	28.19
Supplementary Figure S3	MCAO 3h	18.45	18.63	18.72	30.08	30.33	29.65
Supplementary Figure S3	SHAM	18.89	18.88	18.98	28.35	28.09	28.96
Supplementary Figure S3	SHAM	18.35	18.6	18.88	28.61	29.93	29.69
Supplementary Figure S3	SHAM	18.88	18.82	18.88	28.82	28.91	28.97
Supplementary Figure S3	MCAO 24h	20.01	20.23	20.11	30.27	30.03	30.17
Supplementary Figure S3	MCAO 24h	20.03	20.03	20.06	30.66	30.51	30.78
Supplementary Figure S3	MCAO 24h	20.13	20.08	20.68	31.44	31.22	31.31
Supplementary Figure S3		β-actin	β-actin	β-actin	circ_45921	circ_45921	circ_45921
Supplementary Figure S3	SHAM	19.34	19.08	19.34	30.21	30.37	30.13
Supplementary Figure S3	SHAM	19.11	19.67	19.43	30.07	30.51	30.71
Supplementary Figure S3	SHAM	18.29	18.54	18.46	29.78	30.02	29.88
Supplementary Figure S3	MCAO 5m	19.89	19.98	19.32	32.02	31.96	32.01
Supplementary Figure S3	MCAO 5m	19.98	19.63	19.35	31.88	32.42	32.34
Supplementary Figure S3	MCAO 5m	20.01	20.03	19.92	32.28	32.31	32.55
Supplementary Figure S3	SHAM	19.03	19.25	19.54	31.13	31.36	31.28
Supplementary Figure S3	SHAM	19.23	19.95	19.35	30.87	30.96	30.93
Supplementary Figure S3	SHAM	19.12	19.23	19.22	31.02	31.08	31.22
Supplementary Figure S3	MCAO 3h	20.04	20.25	20.44	35.77	35.77	35.58
Supplementary Figure S3	MCAO 3h	20.03	20.05	20.06	35.55	35.65	35.9
Supplementary Figure S3	MCAO 3h	20.43	20.08	20.68	35.98	35.55	35.05
Supplementary Figure S3	SHAM	19.59	19.55	19.94	29.99	29.31	30.25
Supplementary Figure S3	SHAM	19.33	19.6	19.44	29.51	29.43	30.03
Supplementary Figure S3	SHAM	19.76	19.82	19.88	30.01	30.3	30.21
Supplementary Figure S3	MCAO 24h	21.01	21.23	21.11	32.17	32.89	32.97
Supplementary Figure S3	MCAO 24h	21.14	21.02	21.06	32.83	32.28	32.91
Supplementary Figure S3	MCAO 24h	21.23	21.08	21.53	33.34	32.68	32.76
Supplementary Figure S3		β-actin	β-actin	β-actin	m45921	m45921	m45921
Supplementary Figure S3	SHAM	19.34	19.08	19.34	29.22	29.79	29.55
Supplementary Figure S3	SHAM	19.11	19.67	19.43	29.17	29.45	29.12
Supplementary Figure S3	SHAM	18.29	18.54	18.46	29.13	29.01	29.04
Supplementary Figure S3	MCAO 5m	19.89	19.98	19.32	30.64	30.76	30.88
Supplementary Figure S3	MCAO 5m	19.98	19.63	19.35	29.78	29.44	29.48
Supplementary Figure S3	MCAO 5m	20.01	20.03	19.92	30.56	30.31	30.07
Supplementary Figure S3	SHAM	19.03	19.25	19.54	28.39	28.94	28.76
Supplementary Figure S3	SHAM	19.23	19.95	19.35	28.93	28.78	28.89
Supplementary Figure S3	SHAM	19.12	19.23	19.22	29.31	29.44	29.29
Supplementary Figure S3	MCAO 3h	20.04	20.25	20.44	29.43	29.02	29.11
Supplementary Figure S3	MCAO 3h	20.03	20.05	20.06	29.01	29.34	29.54
Supplementary Figure S3	MCAO 3h	20.43	20.08	20.68	29.31	29.04	29.03
Supplementary Figure S3	SHAM	19.59	19.55	19.94	29.19	28.96	29.09
Supplementary Figure S3	SHAM	19.33	19.6	19.44	29.01	29.03	29.09
Supplementary Figure S3	SHAM	19.76	19.82	19.88	29.03	29.92	28.14
Supplementary Figure S3	MCAO 24h	21.01	21.23	21.11	30.32	30.56	30.89
Supplementary Figure S3	MCAO 24h	21.14	21.02	21.06	30.79	31.08	31.03
Supplementary Figure S3	MCAO 24h	21.23	21.08	21.53	30.64	30.87	30.97
Supplementary Figure S3		β-actin	β-actin	β-actin	m35605	m35605	m35605

Supplementary Figure S3	SHAM	18.22	18.33	19.93	27.24	27.37	27.47
Supplementary Figure S3	SHAM	18.12	18.35	18.22	27.15	27.31	27.56
Supplementary Figure S3	SHAM	18.2	18.07	18.08	27.51	27.27	27.43
Supplementary Figure S3	MCAO 5m	18.45	18.16	18.13	27.53	27.02	27.18
Supplementary Figure S3	MCAO 5m	18.47	18.39	18.45	27.4	27.62	27.17
Supplementary Figure S3	MCAO 5m	18.35	18.16	18.21	27.34	27.44	27.44
Supplementary Figure S3		β-actin	β-actin	β-actin	m26021	m26021	m26021
Supplementary Figure S3	SHAM	18.22	18.33	19.93	28.34	28.36	28.37
Supplementary Figure S3	SHAM	18.12	18.35	18.22	28.24	28.31	28.16
Supplementary Figure S3	SHAM	18.2	18.07	18.08	28.56	28.26	28.4
Supplementary Figure S3	MCAO 5m	18.45	18.16	18.13	28.92	28.45	28.24
Supplementary Figure S3	MCAO 5m	18.47	18.39	18.45	28.46	28.5	28.39
Supplementary Figure S3	MCAO 5m	18.35	18.16	18.21	28.83	28.94	28.84
Supplementary Figure S3		β-actin	β-actin	β-actin	m32190	m32190	m32190
Supplementary Figure S3	SHAM	19.14	18.08	18.24	28.32	28.44	28.02
Supplementary Figure S3	SHAM	18.45	18.67	19.06	28.83	29.18	29
Supplementary Figure S3	SHAM	18.09	18.13	18.08	28.45	28.99	28.65
Supplementary Figure S3	MCAO 5m	19.32	19.46	19.42	30.12	30.33	30.14
Supplementary Figure S3	MCAO 5m	18.97	19.04	19.29	30.26	30.54	30.09
Supplementary Figure S3	MCAO 5m	19.45	19.4	19.21	30.23	30.11	30.24
Supplementary Figure S3		β-actin	β-actin	β-actin	m41878	m41878	m41878
Supplementary Figure S3	SHAM	19.14	18.08	18.24	27.54	27.23	27.08
Supplementary Figure S3	SHAM	18.45	18.67	19.06	27.05	27.13	27.17
Supplementary Figure S3	SHAM	18.09	18.13	18.08	27.21	27.06	27.13
Supplementary Figure S3	MCAO 5m	19.32	19.46	19.42	28.08	28.11	28.21
Supplementary Figure S3	MCAO 5m	18.97	19.04	19.29	28.06	27.83	27.8
Supplementary Figure S3	MCAO 5m	19.45	19.4	19.21	27.95	27.93	28.03
Supplementary Figure S3		β-actin	β-actin	β-actin	m34491	m34491	m34491
Supplementary Figure S3	SHAM	19.05	19.61	16.8	29.02	28.15	28.1
Supplementary Figure S3	SHAM	19.12	19.68	19.86	28.48	28.78	29.07
Supplementary Figure S3	SHAM	19.08	19.32	19.82	28.99	29.03	28.98
Supplementary Figure S3	MCAO 5m	19.05	19.89	19.96	28.69	28.73	28.51
Supplementary Figure S3	MCAO 5m	19.95	19.32	19.24	31.32	31.87	31.06
Supplementary Figure S3	MCAO 5m	19.89	19.6	19.02	27.791	28.02	27.64
Supplementary Figure S3		β-actin	β-actin	β-actin	m003795	m003795	m003795
Supplementary Figure S3	SHAM	19.05	19.61	16.8	29.06	28.22	28.4
Supplementary Figure S3	SHAM	19.12	19.68	19.86	28.58	28.68	29.01
Supplementary Figure S3	SHAM	19.08	19.32	19.82	29.23	28.96	28.96
Supplementary Figure S3	MCAO 5m	19.05	19.89	19.96	29.29	29.43	29.08
Supplementary Figure S3	MCAO 5m	19.95	19.32	19.24	29.32	29.87	29.26
Supplementary Figure S3	MCAO 5m	19.89	19.6	19.02	30.09	28.72	29.64
Supplementary Figure S3		β-actin	β-actin	β-actin	m40203	m40203	m40203
Supplementary Figure S3	SHAM	17.59	17.98	17.68	26.16	26.89	26.76
Supplementary Figure S3	SHAM	17.24	17.51	17.58	26.32	26.55	26.29
Supplementary Figure S3	SHAM	18.14	18.04	18.79	26.81	26.68	26.42
Supplementary Figure S3	MCAO 3h	18.13	18.05	18.07	26.05	25.89	26.24
Supplementary Figure S3	MCAO 3h	17.66	17.21	17.33	26.23	25.77	25.38
Supplementary Figure S3	MCAO 3h	18.66	18.38	18.5	27.02	26.06	26.28
Supplementary Figure S3		β-actin	β-actin	β-actin	m45876	m45876	m45876
Supplementary Figure S3	SHAM	17.59	17.98	17.68	26.33	26.79	26.54
Supplementary Figure S3	SHAM	17.24	17.51	17.58	26.09	26.01	26.13
Supplementary Figure S3	SHAM	18.14	18.04	18.79	26.68	26.28	27.56
Supplementary Figure S3	MCAO 3h	18.13	18.05	18.07	28.05	28.89	28.24
Supplementary Figure S3	MCAO 3h	17.66	17.21	17.33	28.23	27.97	28.58
Supplementary Figure S3	MCAO 3h	18.66	18.38	18.5	27.02	27.76	27.67
Supplementary Figure S3		β-actin	β-actin	β-actin	m24344	m24344	m24344
Supplementary Figure S3	SHAM	17.39	17.45	17.23	26.56	26.48	26.97
Supplementary Figure S3	SHAM	17.53	18.01	17.79	26.97	26.89	26.68
Supplementary Figure S3	SHAM	17.29	17.66	17.58	26.58	26.28	27.56
Supplementary Figure S3	MCAO 3h	18.24	19.01	18.88	30.05	29.97	30.14
Supplementary Figure S3	MCAO 3h	19.66	19.21	19.33	29.63	30.24	29.78
Supplementary Figure S3	MCAO 3h	18.39	18.84	18.57	30.01	29.89	30.12
Supplementary Figure S3		β-actin	β-actin	β-actin	m37699	m37699	m37699
Supplementary Figure S3	SHAM	17.39	17.45	17.23	27.01	27.34	27.54
Supplementary Figure S3	SHAM	17.53	18.01	17.79	27.32	27	27.12
Supplementary Figure S3	SHAM	17.29	17.66	17.58	27.66	27.3	27.54
Supplementary Figure S3	MCAO 3h	18.24	19.01	18.88	29.07	28.44	28.85
Supplementary Figure S3	MCAO 3h	19.66	19.21	19.33	29.013	28.68	28.64
Supplementary Figure S3	MCAO 3h	18.39	18.84	18.57	28.93	28.91	28.54
Supplementary Figure S3		β-actin	β-actin	β-actin	m38328	m38328	m38328
Supplementary Figure S3	SHAM	18.82	18.78	18.85	27.11	27.22	27.03
Supplementary Figure S3	SHAM	17.92	17.91	18.12	27.31	27.55	27.62
Supplementary Figure S3	SHAM	19.2	19.56	19.83	27.21	27.69	27.66
Supplementary Figure S3	MCAO 3h	20.76	19.98	20.25	26.69	26.64	26.41
Supplementary Figure S3	MCAO 3h	20.47	20.23	20.13	26.99	26.87	26.49
Supplementary Figure S3	MCAO 3h	20.35	20.19	20.13	26.72	26.41	26.63

Supplementary Figure S3		β-actin	β-actin	β-actin	m26316	m26316	m26316
Supplementary Figure S3	SHAM	18.82	18.78	18.85	27.83	27.62	27.88
Supplementary Figure S3	SHAM	17.92	17.91	18.12	27.08	27.05	27.11
Supplementary Figure S3	SHAM	19.2	19.56	19.83	27.55	27.33	27.54
Supplementary Figure S3	MCAO 3h	20.76	19.98	20.25	28.14	28.21	28.06
Supplementary Figure S3	MCAO 3h	20.47	20.23	20.13	28.14	28.22	28.24
Supplementary Figure S3	MCAO 3h	20.35	20.19	20.13	29.42	29.37	29.39
Supplementary Figure S3		β-actin	β-actin	β-actin	m43956	m43956	m43956
Supplementary Figure S3	SHAM	18.86	18.87	18.91	25.13	25.18	25.19
Supplementary Figure S3	SHAM	18.69	18.63	18.69	25.11	25.04	25.07
Supplementary Figure S3	SHAM	18.75	18.49	18.67	25.19	25.33	25.27
Supplementary Figure S3	MCAO 24h	19.6	19.51	19.75	27.57	27.62	27.43
Supplementary Figure S3	MCAO 24h	19.58	19.52	19.55	28.42	28.57	28.4
Supplementary Figure S3	MCAO 24h	19.55	19.39	19.37	27.8	27.87	27.69
Supplementary Figure S3		β-actin	β-actin	β-actin	m37286	m37286	m37286
Supplementary Figure S3	SHAM	18.86	18.87	18.91	26.13	26.18	26.19
Supplementary Figure S3	SHAM	18.69	18.63	18.69	25.96	26.01	26.02
Supplementary Figure S3	SHAM	18.75	18.49	18.67	26.39	26.33	26.38
Supplementary Figure S3	MCAO 24h	19.6	19.51	19.75	26.84	26.88	26.76
Supplementary Figure S3	MCAO 24h	19.58	19.52	19.55	26.66	26.76	26.73
Supplementary Figure S3	MCAO 24h	19.55	19.39	19.37	27.14	27.25	27.12
Supplementary Figure S3		β-actin	β-actin	β-actin	m40203	m40203	m40203
Supplementary Figure S3	SHAM	18.78	18.63	18.71	26.15	26.1	26.28
Supplementary Figure S3	SHAM	18.78	18.53	18.75	26.52	26.46	26.51
Supplementary Figure S3	SHAM	18.38	18.31	18.11	26.43	26.41	26.34
Supplementary Figure S3	MCAO 24h	19.52	19.62	19.55	27.97	27.96	28.02
Supplementary Figure S3	MCAO 24h	19.62	19.85	19.76	28.01	28.05	27.98
Supplementary Figure S3	MCAO 24h	19.14	19.88	19.9	28.05	28.01	28.03
Supplementary Figure S3		β-actin	β-actin	β-actin	m41878	m41878	m41878
Supplementary Figure S3	SHAM	18.78	18.63	18.71	25.37	25.46	25.68
Supplementary Figure S3	SHAM	18.78	18.53	18.75	26.96	26.01	26.02
Supplementary Figure S3	SHAM	18.38	18.31	18.11	26.39	26.33	26.38
Supplementary Figure S3	MCAO 24h	19.52	19.62	19.55	28.67	28.72	28.68
Supplementary Figure S3	MCAO 24h	19.62	19.85	19.76	27.46	27.41	27.43
Supplementary Figure S3	MCAO 24h	19.14	19.88	19.9	27.64	27.75	27.92
Supplementary Figure S3		β-actin	β-actin	β-actin	m40795	m40795	m40795
Supplementary Figure S3	SHAM	17.29	17.73	17.52	28.15	28.03	28.13
Supplementary Figure S3	SHAM	17.32	17.53	17.38	27.11	27.01	27.02
Supplementary Figure S3	SHAM	17.4	17.42	17.48	27.4	27.76	27.1
Supplementary Figure S3	MCAO 24h	18.68	18.62	18.55	30.76	30.93	30.89
Supplementary Figure S3	MCAO 24h	18.62	18.85	18.69	28.45	28.72	28.53
Supplementary Figure S3	MCAO 24h	18.87	19.88	19.04	29.59	29.63	29.29
Supplementary Figure S3		β-actin	β-actin	β-actin	m003795	m003795	m003795
Supplementary Figure S3	SHAM	17.29	17.73	17.52	27.45	27.53	27.53
Supplementary Figure S3	SHAM	17.32	17.53	17.38	27.18	27.08	27.02
Supplementary Figure S3	SHAM	17.4	17.42	17.48	27.04	27.06	27.18
Supplementary Figure S3	MCAO 24h	18.68	18.62	18.55	29.37	28.53	28.49
Supplementary Figure S3	MCAO 24h	18.62	18.85	18.69	29.01	28.92	28.83
Supplementary Figure S3	MCAO 24h	18.87	19.88	19.04	28.59	28.63	29.29
Figure Supplementary 5		β-actin	β-actin	β-actin	mmu_circ_41878	mmu_circ_41878	mmu_circ_41878
Figure Supplementary 5	SHAM	19.89	19.88	19.73	34.36	35.28	34.26
Figure Supplementary 5	SHAM	19.74	19.81	19.89	34.11	35.75	35.33
Figure Supplementary 5	SHAM	20.04	19.94	19.69	35.44	34.66	34.82
Figure Supplementary 5	MCAO 5m	20.13	20.05	20.07	34.5	35.02	34.56
Figure Supplementary 5	MCAO 5m	20.17	20.11	20.23	35	35.64	34.59
Figure Supplementary 5	MCAO 5m	19.62	19.44	19.36	34.72	34.45	34.93
Figure Supplementary 5		β-actin	β-actin	β-actin	mmu_circ_35605	mmu_circ_35605	mmu_circ_35605
Figure Supplementary 5	SHAM	19.89	19.88	19.73	31.3	31.32	31.2
Figure Supplementary 5	SHAM	19.74	19.81	19.89	31.37	31.09	31.21
Figure Supplementary 5	SHAM	20.04	19.94	19.69	31.33	31.61	31.65
Figure Supplementary 5	MCAO 5m	20.13	20.05	20.07	31.09	31.25	31.46
Figure Supplementary 5	MCAO 5m	20.17	20.11	20.23	31.2	31.03	31.16
Figure Supplementary 5	MCAO 5m	19.62	19.44	19.36	30.7	30.94	30.73
Figure Supplementary 5		β-actin	β-actin	β-actin	mmu_circ_26021	mmu_circ_26021	mmu_circ_26021
Figure Supplementary 5	SHAM	19.89	19.88	19.73	31.52	31.47	31.14
Figure Supplementary 5	SHAM	19.74	19.81	19.89	31.43	31.29	31.68
Figure Supplementary 5	SHAM	20.04	19.94	19.69	31.61	31.66	31.31
Figure Supplementary 5	MCAO 5m	20.13	20.05	20.07	31.56	31.77	31.89
Figure Supplementary 5	MCAO 5m	20.17	20.11	20.23	31.34	31.65	31.46
Figure Supplementary 5	MCAO 5m	19.62	19.44	19.36	30.97	30.76	30.42
Figure Supplementary 5		β-actin	β-actin	β-actin	mmu_circ_32190	mmu_circ_32190	mmu_circ_32190
Figure Supplementary 5	SHAM	19.89	19.88	19.73	35.36	34.21	34.42
Figure Supplementary 5	SHAM	19.74	19.81	19.89	35.09	35.69	35.46
Figure Supplementary 5	SHAM	20.04	19.94	19.69	33.12	33.55	33.11
Figure Supplementary 5	MCAO 5m	20.13	20.05	20.07	35.03	35.19	35.77
Figure Supplementary 5	MCAO 5m	20.17	20.11	20.23	34.6	35.08	35.86

Figure Supplementary 5	MCAO 5m	19.62	19.44	19.36	33.74	34.33	34.53
Figure Supplementary 5		β- actin	β- actin	β- actin	mmu_circ_34491	mmu_circ_34491	mmu_circ_34491
Figure Supplementary 5	SHAM	20.28	20.33	20.21	33.01	32.68	32.48
Figure Supplementary 5	SHAM	19.74	19.74	19.8	32.63	32.41	32.83
Figure Supplementary 5	SHAM	19.93	19.78	19.63	32.82	32.34	32.31
Figure Supplementary 5	MCAO 5m	20.52	20.62	20.55	32.93	33.07	33.25
Figure Supplementary 5	MCAO 5m	20.3	20.21	20.2	32.6	32.36	33.14
Figure Supplementary 5	MCAO 5m	19.7	19.36	19.41	32.12	32.09	32.55
Figure Supplementary 5		β- actin	β- actin	β- actin	mmu_circ_003795	mmu_circ_003795	mmu_circ_003795
Figure Supplementary 5	SHAM	19.85	19.81	19.8	30.22	30.25	30.2
Figure Supplementary 5	SHAM	19.76	19.8	19.84	30.03	30.2	30.13
Figure Supplementary 5	SHAM	19.93	19.78	19.63	30.51	30.59	30.58
Figure Supplementary 5	MCAO 5m	20.1	20.24	20.08	30.24	30.34	30.38
Figure Supplementary 5	MCAO 5m	20.32	20.19	20.24	30.54	30.45	30.37
Figure Supplementary 5	MCAO 5m	19.7	19.36	19.41	29.5	29.57	29.67
Figure Supplementary 5		β- actin	β- actin	β- actin	mmu_circ_45876	mmu_circ_45876	mmu_circ_45876
Figure Supplementary 5	SHAM	19.68	19.82	19.81	31.32	31.37	31.53
Figure Supplementary 5	SHAM	19.75	19.74	19.82	31.95	31.43	31.3
Figure Supplementary 5	SHAM	20.05	19.83	19.77	30.98	30.69	31.04
Figure Supplementary 5	MCAO 3h	20.12	20.08	20.08	31.59	31.15	31.4
Figure Supplementary 5	MCAO 3h	19.68	19.66	19.72	31.08	30.91	31.04
Figure Supplementary 5	MCAO 3h	20.14	19.82	19.77	30.5	30.43	30.12
Figure Supplementary 5		β- actin	β- actin	β- actin	mmu_circ_37699	mmu_circ_37699	mmu_circ_37699
Figure Supplementary 5	SHAM	19.68	19.82	19.81	35.28	35.19	37.41
Figure Supplementary 5	SHAM	19.75	19.74	19.82	35.78	36.23	36.6
Figure Supplementary 5	SHAM	20.05	19.83	19.77	35.93	35.23	36.18
Figure Supplementary 5	MCAO 3h	20.12	20.08	20.08	37.16	37.75	36.59
Figure Supplementary 5	MCAO 3h	19.68	19.66	19.72	35.28	36.43	36.39
Figure Supplementary 5	MCAO 3h	20.14	19.82	19.77	36.29	35.82	36.24
Figure Supplementary 5		β- actin	β- actin	β- actin	mmu_circ_26316	mmu_circ_26316	mmu_circ_26316
Figure Supplementary 5	SHAM	19.68	19.82	19.81	35.45	35.56	35.1
Figure Supplementary 5	SHAM	19.75	19.74	19.82	35.3	35.67	35.35
Figure Supplementary 5	SHAM	20.05	19.83	19.77	34.84	34.67	34.58
Figure Supplementary 5	MCAO 3h	20.12	20.08	20.08	37.3	36.04	35.55
Figure Supplementary 5	MCAO 3h	19.68	19.66	19.72	35.14	35.36	35.5
Figure Supplementary 5	MCAO 3h	20.14	19.82	19.77	35.87	35.66	35.34
Figure Supplementary 5		β- actin	β- actin	β- actin	mmu_circ_41878	mmu_circ_41878	mmu_circ_41878
Figure Supplementary 5	SHAM	19.68	19.82	19.81	32.58	33.41	33.42
Figure Supplementary 5	SHAM	19.75	19.74	19.82	33.39	32.96	33.76
Figure Supplementary 5	SHAM	20.05	19.83	19.77	33.58	33.41	33.54
Figure Supplementary 5	MCAO 3h	20.12	20.08	20.08	33.32	33.39	33.38
Figure Supplementary 5	MCAO 3h	19.68	19.66	19.72	34.06	33.33	34.06
Figure Supplementary 5	MCAO 3h	20.14	19.82	19.77	33.22	33.42	33.31
Figure Supplementary 5		β- actin	β- actin	β- actin	mmu_circ_24344	mmu_circ_24344	mmu_circ_24344
Figure Supplementary 5	SHAM	20.28	20.33	20.21	35.14	33.52	34.89
Figure Supplementary 5	SHAM	20.28	20.33	20.25	35.27	35.24	35.28
Figure Supplementary 5	SHAM	20.38	20.31	20.11	32.95	33.13	33.24
Figure Supplementary 5	MCAO 3h	20.52	20.62	20.55	34.61	34.61	35.43
Figure Supplementary 5	MCAO 3h	20.62	20.85	20.76	34.59	34.67	35.16
Figure Supplementary 5	MCAO 3h	20.14	19.88	19.9	32.46	32.28	33.22
Figure Supplementary 5		β- actin	β- actin	β- actin	mmu_circ_38328	mmu_circ_38328	mmu_circ_38328
Figure Supplementary 5	SHAM	19.62	19.78	19.71	32.98	32.21	32.38
Figure Supplementary 5	SHAM	19.74	19.74	19.8	32.83	32.96	33.27
Figure Supplementary 5	SHAM	19.97	19.85	19.58	32.24	32.23	32.48
Figure Supplementary 5	MCAO 3h	19.89	19.93	20.02	33.04	32.79	33
Figure Supplementary 5	MCAO 3h	19.62	19.63	19.56	32.32	32.32	32.63
Figure Supplementary 5	MCAO 3h	20.05	19.85	19.85	32.26	32.35	32.2
Figure Supplementary 5		β- actin	β- actin	β- actin	mmu_circ_40203	mmu_circ_40203	mmu_circ_40203
Figure Supplementary 5	SHAM	19.85	19.81	19.8	35.53	34.51	37.08
Figure Supplementary 5	SHAM	19.76	19.8	19.84	36.04	35.53	34.21
Figure Supplementary 5	SHAM	19.93	19.78	19.63	34.26	33.76	34.02
Figure Supplementary 5	MCAO 3h	20.09	20.02	20.01	35.03	33.69	35.18
Figure Supplementary 5	MCAO 3h	19.66	19.68	19.73	34.8	34.42	35.49
Figure Supplementary 5	MCAO 3h	20.05	19.83	19.84	34.55	34.04	34.13
Figure Supplementary 5		β- actin	β- actin	β- actin	mmu_circ_34491	mmu_circ_34491	mmu_circ_34491
Figure Supplementary 5	SHAM	19.96	20.05	19.94	33	33.11	33.12
Figure Supplementary 5	SHAM	20.12	20.03	20.08	32.67	32.72	33.24
Figure Supplementary 5	SHAM	20.08	20.05	19.84	32.52	32.35	32.74
Figure Supplementary 5	MCAO 3h	20.7	20.85	20.74	33.28	33.52	33.82
Figure Supplementary 5	MCAO 3h	20.51	20.36	20.41	33.34	32.51	33.31
Figure Supplementary 5	MCAO 3h	20.33	20.09	20.18	32.78	32.61	32.56
Figure Supplementary 5		β- actin	β- actin	β- actin	mmu_circ_003795	mmu_circ_003795	mmu_circ_003795
Figure Supplementary 5	SHAM	19.91	19.96	19.89	30.52	30.16	30.44
Figure Supplementary 5	SHAM	20.05	19.92	20.02	30.37	30.42	30.57
Figure Supplementary 5	SHAM	19.97	19.94	19.73	30.82	30.8	31.08
Figure Supplementary 5	MCAO 3h	20.69	20.74	20.55	30.93	30.87	31.23

Figure Supplementary 5	MCAO 3h		20.33	20.26	20.29	30.06	29.88	30.52
Figure Supplementary 5	MCAO 3h		20.29	19.93	20.03	30.86	30.51	31.11
Figure Supplementary 5			β-actin	β-actin	β-actin	mmu_circ_43956	mmu_circ_43956	mmu_circ_43956
Figure Supplementary 5	SHAM		19.63	19.76	19.64	27.71	28.2	27.63
Figure Supplementary 5	SHAM		19.68	19.6	19.55	27.81	27.77	27.69
Figure Supplementary 5	SHAM		19.84	19.68	19.52	28.15	27.7	27.75
Figure Supplementary 5	MCAO 24h		21.32	21.24	21.12	28.66	28.4	28.55
Figure Supplementary 5	MCAO 24h		20.1	20.07	20.05	27.3	27.14	27.19
Figure Supplementary 5	MCAO 24h		21.37	21.15	21.18	29.32	29.11	29.07
Figure Supplementary 5			β-actin	β-actin	β-actin	mmu_circ_37286	mmu_circ_37286	mmu_circ_37286
Figure Supplementary 5	SHAM		19.63	19.76	19.64	36.08	36.8	36.19
Figure Supplementary 5	SHAM		19.68	19.6	19.55	36.05	36.14	35.97
Figure Supplementary 5	SHAM		19.84	19.68	19.52	35.22	34.68	35.18
Figure Supplementary 5	MCAO 24h		21.32	21.24	21.12	38.25	36.8	39.05
Figure Supplementary 5	MCAO 24h		20.1	20.07	20.05	36.36	36.33	36.26
Figure Supplementary 5	MCAO 24h		21.37	21.15	21.18	38.04	38.6	38.58
Figure Supplementary 5			β-actin	β-actin	β-actin	mmu_circ_40795	mmu_circ_40795	mmu_circ_40795
Figure Supplementary 5	SHAM		19.63	19.76	19.64	30.63	30.33	30.78
Figure Supplementary 5	SHAM		19.68	19.6	19.55	31.12	30.74	31.01
Figure Supplementary 5	SHAM		19.84	19.68	19.52	31.3	30.79	30.66
Figure Supplementary 5	MCAO 24h		21.32	21.24	21.12	31.26	31.41	31.47
Figure Supplementary 5	MCAO 24h		20.1	20.07	20.05	33.09	33.89	33
Figure Supplementary 5	MCAO 24h		21.37	21.15	21.18	33.12	32.95	32.1
Figure Supplementary 5			β-actin	β-actin	β-actin	mmu_circ_41878	mmu_circ_41878	mmu_circ_41878
Figure Supplementary 5	SHAM		19.63	19.76	19.64	33.16	33.53	33
Figure Supplementary 5	SHAM		19.63	19.76	19.64	33.36	33.28	32.99
Figure Supplementary 5	SHAM		19.63	19.76	19.64	32.98	33.6	32.67
Figure Supplementary 5	MCAO 24h		20.12	20.08	20.08	33.12	33.31	32.98
Figure Supplementary 5	MCAO 24h		19.68	19.66	19.72	32.92	33.12	32.99
Figure Supplementary 5	MCAO 24h		20.14	19.82	19.77	32.91	33.61	33.03
Figure Supplementary 5			β-actin	β-actin	β-actin	mmu_circ_40203	mmu_circ_40203	mmu_circ_40203
Figure Supplementary 5	SHAM		19.85	19.81	19.8	35.53	34.51	37.08
Figure Supplementary 5	SHAM		19.76	19.8	19.84	36.04	35.53	34.21
Figure Supplementary 5	SHAM		19.93	19.78	19.63	34.26	33.76	34.02
Figure Supplementary 5	MCAO 24h		21.38	21.34	21.42	34.35	35.98	35.21
Figure Supplementary 5	MCAO 24h		20.27	20.23	20.35	35.89	34.99	33.8
Figure Supplementary 5	MCAO 24h		21.55	21.34	21.38	35.81	34.94	35.27
Figure Supplementary 5			β-actin	β-actin	β-actin	mmu_circ_003795	mmu_circ_003795	mmu_circ_003795
Figure Supplementary 5	SHAM		19.97	20	19.93	30.69	30.11	30.46
Figure Supplementary 5	SHAM		19.93	20.01	20.11	30.25	30.34	30.49
Figure Supplementary 5	SHAM		20.09	20.02	19.79	30.9	31.14	31.52
Figure Supplementary 5	MCAO 24h		20.63	20.79	20.57	31.5	31.83	31.79
Figure Supplementary 5	MCAO 24h		20.39	20.3	20.34	30.31	30.27	30.25
Figure Supplementary 5	MCAO 24h		20.31	19.99	20.05	32.41	32.9	33.72
Figure Supplementary 6			β-actin	β-actin	β-actin	Gcnt2	Gcnt2	Gcnt2
Figure Supplementary 6	MCAO 3h	Sham	19.73	19.7	19.66	29.03	28.54	28.28
Figure Supplementary 6	MCAO 3h	con	20.21	20.17	20.14	29.17	29.14	28.84
Figure Supplementary 6	MCAO 3h	Infarct	20.14	20.11	20.08	29.22	29.13	28.66
Figure Supplementary 6	MCAO 3h	Peri-infarct	20.36	20.41	20.29	28.62	29.05	28.66
Figure Supplementary 6	MCAO 3h	Sham	20.18	20.17	20.2	29.05	29.25	29.12
Figure Supplementary 6	MCAO 3h	con	19.24	19.32	19.24	28.86	28.91	28.98
Figure Supplementary 6	MCAO 3h	Infarct	20.16	20.24	20.16	30.18	30.09	30.17
Figure Supplementary 6	MCAO 3h	Peri-infarct	19.26	19.35	19.3	28.03	28.19	28.12
Figure Supplementary 6	MCAO 3h	Sham	19.43	19.42	19.38	28.4	28.48	28.45
Figure Supplementary 6	MCAO 3h	con	20.22	20.34	20.35	30.02	29.8	30.25
Figure Supplementary 6	MCAO 3h	Infarct	21.14	21.31	21.33	29.61	29.95	29.96
Figure Supplementary 6	MCAO 3h	Peri-infarct	19.54	19.6	19.61	28.11	28.1	28.09
Figure Supplementary 6	MCAO 3h		β-actin	β-actin	β-actin	Fut1	Fut1	Fut1
Figure Supplementary 6	MCAO 3h	Sham	21.06	21.05	21.03	41.56	42.04	39.59
Figure Supplementary 6	MCAO 3h	con	21.61	21.63	21.5	41.41	41.57	39.01
Figure Supplementary 6	MCAO 3h	Infarct	21.63	21.77	21.45	40.58	37.92	39.39
Figure Supplementary 6	MCAO 3h	Peri-infarct	21.79	21.87	21.75	38.23	41.05	41.44
Figure Supplementary 6	MCAO 3h	Sham	21.44	21.47	21.44	36.51	40.51	40.08
Figure Supplementary 6	MCAO 3h	con	20.84	20.78	20.65	38.6	39.66	37.83

Figure Supplementary 6	MCAO 3h	Infarct	22.02	22.09	21.73	40.89	41.12	42.9
Figure Supplementary 6	MCAO 3h	Peri-infarct	20.52	20.63	20.64	41.33	41.82	39
Figure Supplementary 6	MCAO 3h	Sham	20.86	20.82	20.9	39.42	39.7	37.35
Figure Supplementary 6	MCAO 3h	con	21.83	21.94	21.96	40.84	40.95	42.13
Figure Supplementary 6	MCAO 3h	Infarct	22.62	22.97	22.94	45.23	43.13	38.21
Figure Supplementary 6	MCAO 3h	Peri-infarct	20.93	20.97	21.07	41.4	41.67	38.61
Figure Supplementary 6	MCAO 3h		β-actin	β-actin	β-actin	Fut9	Fut9	Fut9
Figure Supplementary 6	MCAO 3h	Sham	19.77	19.41	19.44	24.48	24.22	24.08
Figure Supplementary 6	MCAO 3h	con	19.97	20.06	19.94	24.87	24.64	24.3
Figure Supplementary 6	MCAO 3h	Infarct	20.17	20.02	20.03	24.55	24.4	24.41
Figure Supplementary 6	MCAO 3h	Peri-infarct	20.3	20.44	20.36	25.18	24.66	24.72
Figure Supplementary 6	MCAO 3h	Sham	19.88	20	19.91	24.62	24.8	24.62
Figure Supplementary 6	MCAO 3h	con	19.11	19.14	19.1	24.27	24.34	24.29
Figure Supplementary 6	MCAO 3h	Infarct	20.15	20.13	20.04	26.18	26.24	26.03
Figure Supplementary 6	MCAO 3h	Peri-infarct	19.18	19.24	19.23	25.05	25.09	24.99
Figure Supplementary 6	MCAO 3h	Sham	19.21	19.26	19.39	24.09	24.19	24.16
Figure Supplementary 6	MCAO 3h	con	20.07	20.12	20.29	25.03	25.4	25.26
Figure Supplementary 6	MCAO 3h	Infarct	21.02	21.3	21.26	26.53	26.56	26.81
Figure Supplementary 6	MCAO 3h	Peri-infarct	19.5	19.54	19.62	25.11	25.14	25.11
Figure Supplementary 6	MCAO 3h		β-actin	β-actin	β-actin	Acox1	Acox1	Acox1
Figure Supplementary 6	MCAO 3h	Sham	20.12	20	20.06	32.09	31.37	31.34
Figure Supplementary 6	MCAO 3h	con	19.7	19.57	19.56	31.19	31.05	30.58
Figure Supplementary 6	MCAO 3h	Infarct	22.8	22.6	22.53	33.7	33.44	33.33
Figure Supplementary 6	MCAO 3h	Peri-infarct	19.73	19.77	19.7	31.41	31.07	31.09
Figure Supplementary 6	MCAO 3h	Sham	20.53	20.5	20.45	31.76	31.6	31.85
Figure Supplementary 6	MCAO 3h	con	21.51	21.44	21.42	32.71	32.94	32.82
Figure Supplementary 6	MCAO 3h	Infarct	21.53	21.48	21.38	33.01	33.04	32.79
Figure Supplementary 6	MCAO 3h	Peri-infarct	20.17	20.21	20.18	31.68	31.75	31.5
Figure Supplementary 6	MCAO 3h	Sham	19.74	19.83	19.7	31.15	31.08	31.09
Figure Supplementary 6	MCAO 3h	con	20.13	20.24	20.35	31.41	31.69	31.51
Figure Supplementary 6	MCAO 3h	Infarct	21.77	21.76	21.83	33	32.93	32.91
Figure Supplementary 6	MCAO 3h	Peri-infarct	20.24	20.24	20.31	32.19	32.23	32.14
Figure Supplementary 6	MCAO 24h		β-actin	β-actin	β-actin	Ehhadh	Ehhadh	Ehhadh
Figure Supplementary 6	MCAO 24h	Sham	19.92	19.83	19.8	31.09	31.41	31.13
Figure Supplementary 6	MCAO 24h	con	19.5	19.4	19.36	30.9	30.86	30.72
Figure Supplementary 6	MCAO 24h	Infarct	22.62	22.45	22.4	34.21	34.27	33.75
Figure Supplementary 6	MCAO 24h	Peri-infarct	19.51	19.51	19.46	30.9	30.75	30.71
Figure Supplementary 6	MCAO 24h	Sham	19.49	19.59	19.56	30.98	31.23	31.24
Figure Supplementary 6	MCAO 24h	con	20.13	20.21	20.22	31.35	31.66	31.35
Figure Supplementary 6	MCAO 24h	Infarct	21.53	21.61	21.6	31.43	31.66	32.01
Figure Supplementary 6	MCAO 24h	Peri-infarct	20.14	20.15	20.31	31.3	31.36	31.27
Figure Supplementary 6	MCAO 24h	Sham	20.21	20.23	20.25	32	32.07	32.12
Figure Supplementary 6	MCAO 24h	con	21.3	21.31	21.16	32.13	32.21	32.46
Figure Supplementary 6	MCAO 24h	Infarct	21.32	21.26	21.23	32.33	32.3	32.23
Figure Supplementary 6	MCAO 24h	Peri-infarct	20.02	20.02	20.01	31.24	31.14	31.17
Figure Supplementary 6	MCAO 24h		β-actin	β-actin	β-actin	Elovl5	Elovl5	Elovl5
Figure Supplementary 6	MCAO 24h	Sham	19.99	20.07	20	26.8	26.9	26.83
Figure Supplementary 6	MCAO 24h	con	19.72	19.39	19.43	26.58	26.55	26.53

Figure Supplementary 6	MCAO 24h	Infarct	22.79	22.35	22.33	30.83	30.8	30.68
Figure Supplementary 6	MCAO 24h	Peri-infarct	19.6	19.72	19.38	27.5	27.11	27.13
Figure Supplementary 6	MCAO 24h	Sham	20.45	20.28	20.36	27.65	27.7	27.53
Figure Supplementary 6	MCAO 24h	con	21.22	21.35	21.26	28.79	28.77	28.64
Figure Supplementary 6	MCAO 24h	Infarct	21.47	21.35	21.31	29.27	29.14	29.15
Figure Supplementary 6	MCAO 24h	Peri-infarct	20.16	20	20.02	27.67	27.7	27.62
Figure Supplementary 6	MCAO 24h	Sham	19.62	19.61	19.64	26.63	26.6	26.74
Figure Supplementary 6	MCAO 24h	con	19.84	19.8	19.84	27.05	27.12	27.21
Figure Supplementary 6	MCAO 24h	Infarct	21.5	21.56	21.49	28.97	29.11	29.1
Figure Supplementary 6	MCAO 24h	Peri-infarct	19.76	19.55	19.47	27.54	27.41	27.44
Figure Supplementary 6	MCAO 24h		β-actin	β-actin	β-actin	Hadha	Hadha	Hadha
Figure Supplementary 6	MCAO 24h	Sham	21.23	20.97	20.84	26.57	26.42	26.24
Figure Supplementary 6	MCAO 24h	con	20.45	20.14	20.14	26.47	26.39	26.24
Figure Supplementary 6	MCAO 24h	Infarct	23.52	23.16	22.9	29.56	29.08	29.18
Figure Supplementary 6	MCAO 24h	Peri-infarct	20.68	20.29	20.11	27.16	26.84	27.03
Figure Supplementary 6	MCAO 24h	Sham	21.36	21.66	21.12	26.88	26.93	26.95
Figure Supplementary 6	MCAO 24h	con	22.11	21.74	22.18	27.87	27.88	27.68
Figure Supplementary 6	MCAO 24h	Infarct	22.01	21.78	21.72	28.17	28.28	28.13
Figure Supplementary 6	MCAO 24h	Peri-infarct	20.23	20.61	20.67	27.05	27.12	27.03
Figure Supplementary 6	MCAO 24h	Sham	21	20.18	20.54	26.15	26.12	26.23
Figure Supplementary 6	MCAO 24h	con	20.42	20.6	20.44	26.19	26.46	26.27
Figure Supplementary 6	MCAO 24h	Infarct	22.13	22.09	22.09	28.07	28.37	28.31
Figure Supplementary 6	MCAO 24h	Peri-infarct	19.8	19.54	19.7	26.7	26.59	26.59
Figure Supplementary 6	MCAO 3h		β-actin	β-actin	β-actin	TG	TG	TG
Figure Supplementary 6	MCAO 3h	SHAM	20.22	20.11	19.93	31.24	31.37	31.47
Figure Supplementary 6	MCAO 3h	SHAM	20.12	20.11	20.22	31.45	31.81	31.97
Figure Supplementary 6	MCAO 3h	SHAM	20.2	20.07	19.83	31.01	31.28	31.43
Figure Supplementary 6	MCAO 3h	MCAO	20.45	20.16	20.13	31.86	32.26	32.28
Figure Supplementary 6	MCAO 3h	MCAO	20.47	20.39	20.45	31.4	31.62	32.17
Figure Supplementary 6	MCAO 3h	MCAO	20.35	20.16	20.21	31.57	31.76	31.77
Figure Supplementary 6	MCAO 3h	Infarct	20.8	20.49	20.46	32.7	33.12	32.92
Figure Supplementary 6	MCAO 3h	Infarct	20.69	20.63	20.69	32.43	32.4	32.46
Figure Supplementary 6	MCAO 3h	Infarct	20.75	20.49	20.67	32.3	32.47	32.39
Figure Supplementary 6	MCAO 3h	peri-infarct	20.53	20.29	20.32	32.23	32.02	33.24
Figure Supplementary 6	MCAO 3h	peri-infarct	20.58	20.52	20.55	32.71	31.28	31.12
Figure Supplementary 6	MCAO 3h	peri-infarct	20.55	20.39	20.37	31.53	31.57	31.59
Figure Supplementary 6	MCAO 3h		β-actin	β-actin	β-actin	PRCKA	PRCKA	PRCKA
Figure Supplementary 6	MCAO 3h	SHAM	20.02	20.1	20.05	24.05	23.89	23.89
Figure Supplementary 6	MCAO 3h	SHAM	20.12	20.11	20.22	23.84	23.84	23.93
Figure Supplementary 6	MCAO 3h	SHAM	20.2	20.07	19.83	23.91	23.72	23.63
Figure Supplementary 6	MCAO 3h	MCAO	20.76	20.98	20.73	25.09	25.06	25.01
Figure Supplementary 6	MCAO 3h	MCAO	20.47	20.39	20.45	24.68	24.73	24.64
Figure Supplementary 6	MCAO 3h	MCAO	20.35	20.16	20.21	24.23	24.11	24.18
Figure Supplementary 6	MCAO 3h	Infarct	20.86	20.87	20.91	25.13	25.18	25.19
Figure Supplementary 6	MCAO 3h	Infarct	20.69	20.63	20.69	25.11	25.04	25.07
Figure Supplementary 6	MCAO 3h	Infarct	20.75	20.49	20.67	24.46	24.32	24.27
Figure Supplementary 6	MCAO 3h	peri-infarct	20.6	20.51	20.75	24.9	24.92	25.03
Figure Supplementary 6	MCAO 3h	peri-infarct	20.58	20.52	20.55	24.84	24.8	24.77

Figure Supplementary 6	MCAO 3h	peri-infarct	20.55	20.39	20.37	24.9	24.6	24.6
Figure Supplementary 6	MCAO 3h		β-actin	β-actin	β-actin	PLCB2	PLCB2	PLCB2
Figure Supplementary 6	MCAO 3h	SHAM	19.96	20.05	19.94	32.81	32.71	32.14
Figure Supplementary 6	MCAO 3h	SHAM	20.12	20.03	20.08	32.93	32.77	32.48
Figure Supplementary 6	MCAO 3h	SHAM	20.08	20.05	19.84	32.33	32.71	32.55
Figure Supplementary 6	MCAO 3h	MCAO	20.7	20.85	20.74	34.7	34.15	34.44
Figure Supplementary 6	MCAO 3h	MCAO	20.51	20.36	20.41	33.12	33.86	33.48
Figure Supplementary 6	MCAO 3h	MCAO	20.33	20.09	20.18	33.55	33.38	33.88
Figure Supplementary 6	MCAO 3h	Infarct	20.9	20.78	20.91	34.37	34.21	34.84
Figure Supplementary 6	MCAO 3h	Infarct	20.58	20.58	20.56	33.86	33.91	33.88
Figure Supplementary 6	MCAO 3h	Infarct	20.7	20.45	20.48	33.22	33.39	33.69
Figure Supplementary 6	MCAO 3h	peri-infarct	20.51	20.44	20.46	35.81	35.26	34.77
Figure Supplementary 6	MCAO 3h	peri-infarct	20.56	20.48	20.5	34.48	33.9	34.62
Figure Supplementary 6	MCAO 3h	peri-infarct	20.52	20.31	20.39	34.47	33.84	33.73
Figure Supplementary 7	MCAO 5m		β-actin	β-actin	β-actin	mmu_circ_43344	mmu_circ_43344	mmu_circ_43344
Figure Supplementary 7	MCAO 5m	SHAM	20.28	20.33	20.21	34.18	34.18	33.88
Figure Supplementary 7	MCAO 5m	SHAM	20.28	20.33	20.25	34.3	35.18	34.84
Figure Supplementary 7	MCAO 5m	SHAM	20.38	20.31	20.11	33.4	34.76	34.1
Figure Supplementary 7	MCAO 5m	MCAO	20.52	20.62	20.55	34.8	34.95	34.51
Figure Supplementary 7	MCAO 5m	MCAO	20.62	20.85	20.76	35.67	34.75	34.75
Figure Supplementary 7	MCAO 5m	MCAO	20.14	19.88	19.9	35.29	34.12	34.23
Figure Supplementary 7	MCAO 5m		β-actin	β-actin	β-actin	mmu_circ_45921	mmu_circ_45921	mmu_circ_45921
Figure Supplementary 7	MCAO 5m	SHAM	19.62	19.78	19.71	34.24	33.93	33.87
Figure Supplementary 7	MCAO 5m	SHAM	19.74	19.74	19.8	33.99	34.63	35.01
Figure Supplementary 7	MCAO 5m	SHAM	19.97	19.85	19.58	34.15	34.34	34.26
Figure Supplementary 7	MCAO 5m	MCAO	20.06	20.17	20.06	34.06	35.04	35.68
Figure Supplementary 7	MCAO 5m	MCAO	20.3	20.21	20.2	34.98	35.68	34.15
Figure Supplementary 7	MCAO 5m	MCAO	19.61	19.31	19.34	34.64	34.02	35.35
Figure Supplementary 7	MCAO 5m		β-actin	β-actin	β-actin	mmu_circ_43344	mmu_circ_43344	mmu_circ_43344
Figure Supplementary 7	MCAO 3h	SHAM	20.28	20.33	20.21	34.18	34.18	33.88
Figure Supplementary 7	MCAO 3h	SHAM	20.28	20.33	20.25	34.3	35.18	34.84
Figure Supplementary 7	MCAO 3h	SHAM	20.38	20.31	20.11	33.4	34.76	34.1
Figure Supplementary 7	MCAO 3h	MCAO	20.52	20.62	20.55	35.45	34.65	35.09
Figure Supplementary 7	MCAO 3h	MCAO	20.62	20.85	20.76	34.16	34.92	34.03
Figure Supplementary 7	MCAO 3h	MCAO	20.14	19.88	19.9	34.09	34.43	34.09
Figure Supplementary 7	MCAO 3h		β-actin	β-actin	β-actin	mmu_circ_45921	mmu_circ_45921	mmu_circ_45921
Figure Supplementary 7	MCAO 3h	SHAM	19.62	19.78	19.71	34.24	33.93	33.87
Figure Supplementary 7	MCAO 3h	SHAM	19.74	19.74	19.8	33.99	34.63	35.01
Figure Supplementary 7	MCAO 3h	SHAM	19.97	19.85	19.58	34.15	34.34	34.26
Figure Supplementary 7	MCAO 3h	MCAO	19.89	19.93	20.02	36.16	34.72	34.1
Figure Supplementary 7	MCAO 3h	MCAO	19.62	19.63	19.56	34.24	35.12	34.56
Figure Supplementary 7	MCAO 3h	MCAO	20.05	19.85	19.85	34.98	34.52	35.19
Figure Supplementary 7	MCAO 24h		β-actin	β-actin	β-actin	mmu_circ_43344	mmu_circ_43344	mmu_circ_43344
Figure Supplementary 7	MCAO 24h	SHAM	20.28	20.33	20.21	34.18	34.18	33.88
Figure Supplementary 7	MCAO 24h	SHAM	20.28	20.33	20.25	34.3	34.18	34.84
Figure Supplementary 7	MCAO 24h	SHAM	20.38	20.31	20.11	33.4	34.76	34.1
Figure Supplementary 7	MCAO 24h	MCAO	22.02	21.76	21.79	35.03	34.73	34.89
Figure Supplementary 7	MCAO 24h	MCAO	20.69	20.74	20.78	34.26	34.19	34.4

Figure Supplementary 7	MCAO 24h	MCAO	22.03	21.84	21.9	35.78	36.23	35.16
Figure Supplementary 7	MCAO 24h		β-actin	β-actin	β-actin	mmu_circ_45921	mmu_circ_45921	mmu_circ_45921
Figure Supplementary 7	MCAO 24h	SHAM	19.62	19.78	19.71	34.24	33.93	33.87
Figure Supplementary 7	MCAO 24h	SHAM	19.74	19.74	19.8	33.99	34.63	35.01
Figure Supplementary 7	MCAO 24h	SHAM	19.97	19.85	19.58	34.15	34.34	34.26
Figure Supplementary 7	MCAO 24h	MCAO	21.43	21.38	21.37	36.52	35.73	35.61
Figure Supplementary 7	MCAO 24h	MCAO	20.26	20.3	20.3	36.03	36.09	36.26
Figure Supplementary 7	MCAO 24h	MCAO	21.56	21.36	21.39	36.12	36.36	37
Figure Supplementary 8	MCAO 3h		β-actin	β-actin	β-actin	SH3PXD2A	SH3PXD2A	SH3PXD2A
Figure Supplementary 8	MCAO 3h	SHAM	19.72	19.93	19.84	27.47	26.46	27.09
Figure Supplementary 8	MCAO 3h	SHAM	19.86	19.9	19.86	27.11	27.1	27.06
Figure Supplementary 8	MCAO 3h	SHAM	19.91	19.8	19.67	27.16	27.08	26.92
Figure Supplementary 8	MCAO 3h	MCAO	20.58	20.6	20.44	28.89	28.35	28.29
Figure Supplementary 8	MCAO 3h	MCAO	20.23	20.2	20.23	27.79	27.77	27.74
Figure Supplementary 8	MCAO 3h	MCAO	20.17	19.85	19.86	27.43	27.41	27.42
Figure Supplementary 8	MCAO 3h	Infarct	20.6	20.59	20.7	28.58	28.19	28.24
Figure Supplementary 8	MCAO 3h	Infarct	20.4	20.39	20.39	27.96	27.87	27.86
Figure Supplementary 8	MCAO 3h	Infarct	20.51	20.31	20.32	27.85	27.8	27.62
Figure Supplementary 8	MCAO 3h	peri-infarct	20.26	20.28	20.24	28.96	28.41	28.53
Figure Supplementary 8	MCAO 3h	peri-infarct	20.35	20.24	20.33	28.24	28.22	28.2
Figure Supplementary 8	MCAO 3h	peri-infarct	20.32	20.28	20.21	28.3	28.23	28.12
Figure Supplementary 8	MCAO 3h		β-actin	β-actin	β-actin	NNT	NNT	NNT
Figure Supplementary 8	MCAO 3h	SHAM	19.72	19.93	19.84	38.01	38.1	38.5
Figure Supplementary 8	MCAO 3h	SHAM	19.86	19.9	19.86	37.72	38.26	39.32
Figure Supplementary 8	MCAO 3h	SHAM	20.22	20.11	19.93	38.21	38.7	38.11
Figure Supplementary 8	MCAO 3h	MCAO	20.58	20.6	20.44	39.78	40.4	39.98
Figure Supplementary 8	MCAO 3h	MCAO	20.23	20.2	20.23	39.38	39.26	39.1
Figure Supplementary 8	MCAO 3h	MCAO	20.45	20.16	20.13	38.35	39.23	39.3
Figure Supplementary 8	MCAO 3h	Infarct	20.6	20.59	20.7	39.12	39.61	41.58
Figure Supplementary 8	MCAO 3h	Infarct	20.4	20.39	20.39	38.89	39.07	39.13
Figure Supplementary 8	MCAO 3h	Infarct	20.8	20.49	20.46	39.88	39.26	38.66
Figure Supplementary 8	MCAO 3h	peri-infarct	20.26	20.28	20.24	39.67	40.05	40.28
Figure Supplementary 8	MCAO 3h	peri-infarct	20.35	20.24	20.33	38.93	39.17	39.58
Figure Supplementary 8	MCAO 3h	peri-infarct	20.53	20.29	20.32	40.35	38.3	39.92
Figure Supplementary 8	MCAO 3h		β-actin	β-actin	β-actin	VPS26A	VPS26A	VPS26A
Figure Supplementary 8	MCAO 3h	SHAM	20.03	20.09	20.03	28.26	27.92	27.96
Figure Supplementary 8	MCAO 3h	SHAM	20.09	20.16	20.16	27.79	27.84	27.91
Figure Supplementary 8	MCAO 3h	SHAM	20.22	20.11	19.93	28.13	28.05	27.93
Figure Supplementary 8	MCAO 3h	MCAO	20.8	20.97	20.77	29.02	28.96	28.71
Figure Supplementary 8	MCAO 3h	MCAO	20.46	20.41	20.45	28.35	28.3	28.31
Figure Supplementary 8	MCAO 3h	MCAO	20.45	20.16	20.13	28.31	28.22	28.24
Figure Supplementary 8	MCAO 3h	Infarct	20.89	20.84	20.92	29.14	29	28.94
Figure Supplementary 8	MCAO 3h	Infarct	20.66	20.61	20.7	28.55	28.68	28.52
Figure Supplementary 8	MCAO 3h	Infarct	20.8	20.49	20.46	28.72	28.6	28.41
Figure Supplementary 8	MCAO 3h	peri-infarct	20.48	20.47	20.47	30.51	29.25	29.03
Figure Supplementary 8	MCAO 3h	peri-infarct	20.6	20.55	20.45	28.96	28.9	29.01
Figure Supplementary 8	MCAO 3h	peri-infarct	20.53	20.29	20.32	28.55	28.57	28.46
Figure Supplementary 8	MCAO 3h		β-actin	β-actin	β-actin	TMUB2	TMUB2	TMUB2

Figure Supplementary 8	MCAO 3h	SHAM	19.97	20	19.93	29.03	28.98	28.91
Figure Supplementary 8	MCAO 3h	SHAM	19.93	20.01	20.11	28.88	28.71	28.74
Figure Supplementary 8	MCAO 3h	SHAM	20.09	20.02	19.79	28.91	28.93	28.71
Figure Supplementary 8	MCAO 3h	MCAO	20.63	20.79	20.57	30.37	30.3	30.19
Figure Supplementary 8	MCAO 3h	MCAO	20.39	20.3	20.34	29.52	29.4	29.43
Figure Supplementary 8	MCAO 3h	MCAO	20.31	19.99	20.05	29.75	29.57	29.67
Figure Supplementary 8	MCAO 3h	Infarct	20.78	20.74	20.78	30.05	30.01	30.22
Figure Supplementary 8	MCAO 3h	Infarct	20.53	20.52	20.52	29.6	29.51	29.55
Figure Supplementary 8	MCAO 3h	Infarct	20.61	20.4	20.4	30.04	29.9	29.8
Figure Supplementary 8	MCAO 3h	peri-infarct	20.45	20.35	20.35	30.51	30.44	30.45
Figure Supplementary 8	MCAO 3h	peri-infarct	20.6	20.48	20.54	30.45	30.19	30.36
Figure Supplementary 8	MCAO 3h	peri-infarct	20.43	20.29	20.34	30.36	30.03	30.11
Figure Supplementary 8	MCAO 3h		β-actin	β-actin	β-actin	ST8SIA2	ST8SIA2	ST8SIA2
Figure Supplementary 8	MCAO 3h	SHAM	19.91	19.96	19.89	29.41	30.14	30.07
Figure Supplementary 8	MCAO 3h	SHAM	20.05	19.92	20.02	30.06	30.29	30.15
Figure Supplementary 8	MCAO 3h	SHAM	19.97	19.94	19.73	29.91	29.67	29.9
Figure Supplementary 8	MCAO 3h	MCAO	20.69	20.74	20.55	30.94	30.98	31.13
Figure Supplementary 8	MCAO 3h	MCAO	20.33	20.26	20.29	30.15	30.07	30.13
Figure Supplementary 8	MCAO 3h	MCAO	20.29	19.93	20.03	30.48	30.59	30.23
Figure Supplementary 8	MCAO 3h	Infarct	20.68	20.69	20.75	30.87	31.22	30.92
Figure Supplementary 8	MCAO 3h	Infarct	20.48	20.51	20.47	30.53	30.3	30.41
Figure Supplementary 8	MCAO 3h	Infarct	20.64	20.38	20.4	30.79	30.71	31.26
Figure Supplementary 8	MCAO 3h	peri-infarct	20.37	20.35	20.36	30.89	30.88	30.8
Figure Supplementary 8	MCAO 3h	peri-infarct	20.46	20.39	20.4	30.95	30.86	30.97
Figure Supplementary 8	MCAO 3h	peri-infarct	20.41	20.2	20.24	30.92	30.53	30.45
Supplementary Figure S4B			β-actin	β-actin	β-actin	has_circ_0020231	has_circ_0020231	has_circ_0020231
Supplementary Figure S4B	CS1		26.38	26.35	26.38	28.23	29.35	26.31
Supplementary Figure S4B	IS1		24.96	24.9	24.82	26.12	26.12	27.25
Supplementary Figure S4B	CS2		30.32	30.26	30.25	25.22	25.14	25.08
Supplementary Figure S4B	IS2		27.5	27.75	27.62	28.62	30.14	29.55
Supplementary Figure S4B	CS3		29.52	29.33	29.5	21.99	23.74	23.45
Supplementary Figure S4B	IS3		28.12	27.92	28	29.75	30.49	29.35
Supplementary Figure S4B	CS4		24.82	24.91	24.97	24.74	24.32	26.36
Supplementary Figure S4B	IS4		27.92	27.5	27.62	28.3	32.06	31.31
Supplementary Figure S4B	CS5		29.33	28.74	28.68	27.3	26.47	27.04
Supplementary Figure S4B	IS5		26.41	26.37	26.25	27.25	27.44	27.39
Supplementary Figure S4B	CS6		26.9	26.66	26.56	35.98	35.61	34.87
Supplementary Figure S4B	IS6		27.84	27.64	27.66	37.01	36.07	37.51
Supplementary Figure S4B	CS7		28.03	28.02	28.05	29.27	29	29.46
Supplementary Figure S4B	IS7		29.18	29.25	29.31	31.04	31.05	31.06
Supplementary Figure S4B	CS8		27.04	27.05	27.17	29.95	29.01	29.51
Supplementary Figure S4B	IS8		29.08	29.12	29.11	29.14	30.98	28.84
Supplementary Figure S4B			β-actin	β-actin	β-actin	has_circ_0125476	has_circ_0125476	has_circ_0125476
Supplementary Figure S4B	CS1		26.38	26.35	26.38	43.28	38.33	37.72
Supplementary Figure S4B	IS1		24.96	24.9	24.82	37.38	37.95	38.68
Supplementary Figure S4B	CS2		30.32	30.26	30.25	40.03	37.57	43.12
Supplementary Figure S4B	IS2		27.5	27.75	27.62	38.54	36.21	38.56
Supplementary Figure S4B	CS3		29.52	29.33	29.5	37.33	38	37.665
Supplementary Figure S4B	IS3		28.12	27.92	28	36.52	36.87	36.53
Supplementary Figure S4B	CS4		24.82	24.91	24.97	38.51	38.29	37.38
Supplementary Figure S4B	IS4		27.92	27.5	27.62	36.88	37.4	37.14
Supplementary Figure S4B	CS5		29.33	28.74	28.68	35	36.32	35.21
Supplementary Figure S4B	IS5		26.41	26.37	26.25	35.59	36.03	36.87
Supplementary Figure S4B	CS6		26.89	26.69	26.74	34.46	33.93	34.25
Supplementary Figure S4B	IS6		28.43	28.42	28.38	33.95	35.09	34.32
Supplementary Figure S4B	CS7		26.84	26.76	26.75	33.66	34.04	34.03
Supplementary Figure S4B	IS7		28.27	28.1	28.05	34.1	33.92	34.49
Supplementary Figure S4B	CS8		31.71	32.19	31.79	35.17	35.3	35.07
Supplementary Figure S4B	IS8		27.56	27.75	27.64	36.03	35.65	35.23
Supplementary Figure S4B			β-actin	β-actin	β-actin	has_circ_004289	has_circ_004289	has_circ_004289
Supplementary Figure S4B	CS1		26.38	26.35	26.38	39.53	35.29	37.09
Supplementary Figure S4B	IS1		24.96	24.9	24.82	35.8	36.79	35.02
Supplementary Figure S4B	CS2		30.32	30.26	30.25	37.28	36.69	36.62

Supplementary Figure S4B	IS2	27.5	27.75	27.62	36.17	36.53	36.56
Supplementary Figure S4B	CS3	29.52	29.33	29.5	36.52	37.7	38.12
Supplementary Figure S4B	IS3	28.12	27.92	28	37.23	37.58	38.22
Supplementary Figure S4B	CS4	24.82	24.91	24.97	36.39	37.6	36.61
Supplementary Figure S4B	IS4	27.92	27.5	27.62	37.18	38.6	38.38
Supplementary Figure S4B	CS5	29.33	28.74	28.68	40.75	41.2	41.93
Supplementary Figure S4B	IS5	26.41	26.37	26.25	35.27	37.43	42.85
Supplementary Figure S4B	CS6	26.89	26.69	26.74	37.02	36.77	36.69
Supplementary Figure S4B	IS6	28.43	28.42	28.38	40.47	38.94	37.32
Supplementary Figure S4B	CS7	26.84	26.76	26.75	38.94	37.4	38.75
Supplementary Figure S4B	IS7	28.27	28.1	28.05	41.04	38.84	36.4
Supplementary Figure S4B	CS8	31.71	32.19	31.79	38.59	37.6	37.29
Supplementary Figure S4B	IS8	27.56	27.75	27.64	37.36	37.33	38.35
Supplementary Figure S4B		β-actin	β-actin	β-actin	has_circ_0094230	has_circ_0094230	has_circ_0094230
Supplementary Figure S4B	CS1	26.38	26.35	26.38	39.38	37.94	38.66
Supplementary Figure S4B	IS1	24.96	24.9	24.82	42.59	38.23	39.37
Supplementary Figure S4B	CS2	28.19	27.97	28.11	38.08	38.12	38.17
Supplementary Figure S4B	IS2	25.96	26.81	26.1	38.47	39.42	39.42
Supplementary Figure S4B	CS3	29.52	29.33	29.5	37.15	38.43	38.12
Supplementary Figure S4B	IS3	28.12	27.92	28	37.29	38.36	39.31
Supplementary Figure S4B	CS4	24.62	24.52	25.03	38.54	40.35	39.45
Supplementary Figure S4B	IS4	29.86	30.16	30.19	40.21	42.04	41.13
Supplementary Figure S4B	CS5	29.33	28.74	28.68	37.8	38.32	36.11
Supplementary Figure S4B	IS5	26.41	26.37	26.25	36.83	37.07	36.94
Supplementary Figure S4B	CS6	26.89	26.69	26.74	37.28	38.55	36.39
Supplementary Figure S4B	IS6	28.43	28.42	28.38	36.83	36.48	36.83
Supplementary Figure S4B	CS7	26.84	26.76	26.75	38.17	36.69	37.46
Supplementary Figure S4B	IS7	28.27	28.1	28.05	37.83	36.74	37.47
Supplementary Figure S4B	CS8	31.71	32.19	31.79	37.52	36.16	36.66
Supplementary Figure S4B	IS8	27.56	27.75	27.64	37.04	39.77	36.85
Supplementary Figure S4B		β-actin	β-actin	β-actin	has_circ_0053995	has_circ_0053995	has_circ_0053995
Supplementary Figure S4B	CS1	26.38	26.35	26.38	33.88	33.22	32.96
Supplementary Figure S4B	IS1	24.96	24.9	24.82	33.4	33.13	33.32
Supplementary Figure S4B	CS2	30.32	30.26	30.25	32	31.39	32.3
Supplementary Figure S4B	IS2	27.5	27.75	27.62	31.78	31.53	31.83
Supplementary Figure S4B	CS3	29.52	29.33	29.5	32.38	32.52	32.25
Supplementary Figure S4B	IS3	28.12	27.92	28	32.2	32.28	32.87
Supplementary Figure S4B	CS4	24.82	24.91	24.97	33.95	33.76	34.09
Supplementary Figure S4B	IS4	27.92	27.5	27.62	34.4	34.05	33.4
Supplementary Figure S4B	CS5	29.33	28.74	28.68	33.08	32.96	32.59
Supplementary Figure S4B	IS5	26.41	26.37	26.25	33.01	32.14	32.39
Supplementary Figure S4B	CS6	26.9	26.66	26.56	39.585	39.92	39.25
Supplementary Figure S4B	IS6	27.84	27.64	27.66	41.64	39.23	39.65
Supplementary Figure S4B	CS7	28.03	28.02	28.05	37.56	37.26	37.76
Supplementary Figure S4B	IS7	29.18	29.25	29.31	43.79	37.85	43.76
Supplementary Figure S4B	CS8	31.71	32.19	31.79	32.12	31.99	31.95
Supplementary Figure S4B	IS8	27.56	27.75	27.64	31.79	31.88	32.06

Supplementary Table S10. Predicted target genes of miR-1962 by TargetScan

Ortholog of target gene	Representative transcript	Gene name
ST8SIA2	ENST00000268164.3	ST8 alpha-N-acetyl-neuraminide alpha-2,8-sialyltransferase 2
VAC14	ENST00000261776.5	Vac14 homolog (S. cerevisiae)
TMUB2	ENST00000590235.1	transmembrane and ubiquitin-like domain containing 2
SH3PXD2A	ENST00000369774.4	SH3 and PX domains 2A
PHB2	ENST00000546111.1	prohibitin 2
RHO	ENST00000296271.3	rhodopsin
NNT	ENST00000264663.5	nicotinamide nucleotide transhydrogenase
LPP	ENST00000312675.4	LIM domain containing preferred translocation partner in lipoma
VPS26A	ENST00000395098.1	vacuolar protein sorting 26 homolog A (S. pombe)
SERTAD3	ENST00000392028.4	SERTA domain containing 3
SZT2	ENST00000562955.1	seizure threshold 2 homolog (mouse)
FRMPD3	ENST00000276185.4	FERM and PDZ domain containing 3
ZMAT2	ENST00000274712.3	zinc finger, matrin-type 2
SCN11A	ENST00000302328.3	sodium channel, voltage-gated, type XI, alpha subunit
RASGRP2	ENST00000394429.1	RAS guanyl releasing protein 2 (calcium and DAG-regulated)
FAM181B	ENST00000329203.3	family with sequence similarity 181, member B
ATOH8	ENST00000306279.3	atonal homolog 8 (Drosophila)
ARHGEF39	ENST00000343259.3	Rho guanine nucleotide exchange factor (GEF) 39
PLXNA4	ENST00000321063.4	plexin A4
RBPMS	ENST00000320203.4	RNA binding protein with multiple splicing
